# Spin-resolved topology and partial axion angles in three-dimensional insulators

**DOI:** 10.1038/s41467-024-44762-w

**Published:** 2024-01-16

**Authors:** Kuan-Sen Lin, Giandomenico Palumbo, Zhaopeng Guo, Yoonseok Hwang, Jeremy Blackburn, Daniel P. Shoemaker, Fahad Mahmood, Zhijun Wang, Gregory A. Fiete, Benjamin J. Wieder, Barry Bradlyn

**Affiliations:** 1 Department of Physics and Institute for Condensed Matter Theory, University of Illinois at Urbana-Champaign, Urbana, IL 61801 USA; 2 Kavli Institute for Theoretical Physics, University of California, Santa Barbara, CA 93106 USA; 3 School of Theoretical Physics, Dublin Institute for Advanced Studies, 10 Burlington Road, Dublin, 4 Ireland; 4 Beijing National Laboratory for Condensed Matter Physics and Institute of Physics, Chinese Academy of Sciences, 100190 Beijing, China; 5 University of Chinese Academy of Sciences, 100049 Beijing, China; 6 Department of Computer Science, State University of New York at Binghamton, Binghamton, NY 13902 USA; 7 Department of Materials Science and Engineering, University of Illinois at Urbana-Champaign, Urbana, IL 61801 USA; 8 Materials Research Laboratory, University of Illinois at Urbana-Champaign, Urbana, IL 61801 USA; 9 Department of Physics, University of Illinois at Urbana-Champaign, Urbana, IL 61801 USA; 10 Department of Physics, Northeastern University, Boston, MA 02115 USA; 11 Department of Physics, Massachusetts Institute of Technology, Cambridge, MA 02139 USA; 12 Present Address: Institut de Physique Théorique, Université Paris-Saclay, CEA, CNRS, F-91191 Gif-sur-Yvette, France

**Keywords:** Topological insulators, Spintronics, Electronic properties and materials

## Abstract

Symmetry-protected topological crystalline insulators (TCIs) have primarily been characterized by their gapless boundary states. However, in time-reversal- (${{{{{{{\mathcal{T}}}}}}}}$-) invariant (helical) 3D TCIs—termed higher-order TCIs (HOTIs)—the boundary signatures can manifest as a sample-dependent network of 1D hinge states. We here introduce nested spin-resolved Wilson loops and layer constructions as tools to characterize the intrinsic bulk topological properties of spinful 3D insulators. We discover that helical HOTIs realize one of three spin-resolved phases with distinct responses that are quantitatively robust to large deformations of the bulk spin-orbital texture: 3D quantum spin Hall insulators (QSHIs), “spin-Weyl” semimetals, and ${{{{{{{\mathcal{T}}}}}}}}$-doubled axion insulator (T-DAXI) states with nontrivial partial axion angles indicative of a 3D spin-magnetoelectric bulk response and half-quantized 2D TI surface states originating from a partial parity anomaly. Using ab-initio calculations, we demonstrate that *β*-MoTe_2_ realizes a spin-Weyl state and that *α*-BiBr hosts both 3D QSHI and T-DAXI regimes.

## Abstract (web summary)

3D higher-order topological insulators (HOTIs) exhibit 1D hinge states depending on extrinsic sample details, while intrinsic features of HOTIs remain unknown. Here, K.S. Lin et al. introduce the framework of spin-resolved topology to show that helical HOTIs can realize a doubled axion insulator phase with nontrivial partial axion angles.

## Introduction

In recent years, the study of topological phases of matter in solid-state materials has largely focused on their anomalous gapless boundary states^1^. 2D and 3D topological insulators (TIs), for example, exhibit time-reversal- (${{{{{{{\mathcal{T}}}}}}}}$-) symmetry-protected 1D helical modes and 2D Dirac cones on their boundaries^2–6^, respectively. While this focus on gapless boundary states has been validated by remarkable transport and spectroscopy experiments^7–9^, and has revealed promising avenues for chemical applications^10,11^ and interface spintronics^12,13^, it also has drawbacks.

**Fig. 1:**
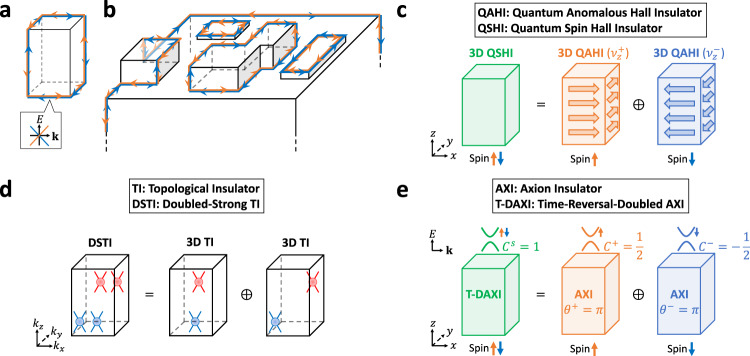
Spin-resolving helical higher-order topological crystalline insulators. **a** A helical higher-order topological crystalline insulator (HOTI) cut into a finite geometry with perfect spatial inversion (${{{{{{{\mathcal{I}}}}}}}}$) symmetry. In **a**, the configuration of intrinsic 1D helical hinge modes indicates the bulk topology^19–22^. **b** A helical HOTI in a more realistic sample geometry^20^ featuring irregular surfaces and broken global ${{{{{{{\mathcal{I}}}}}}}}$ symmetry. The hinge modes in **b** originate from extrinsic sample details and surface physics, and are indistinguishable from the extrinsic helical modes of bulk-trivial materials^45,52^. By computing the gauge-invariant spin-spectrum^55^ (see Fig. 2 and SN 2B), the bulk electronic structure of a helical HOTI with ${{{{{{{\mathcal{I}}}}}}}}$ and time-reversal (${{{{{{{\mathcal{T}}}}}}}}$) symmetries can be further classified into one of three spin-stable phases (SN 4D). **c** The 3D QSHI regime of a helical HOTI, which is constructed by layering 2D TI (QSHI) states with the same spin Hall response^37^. The 3D QSHI state in **c** hence exhibits an extensive bulk spin Hall conductivity per unit cell (see refs. ^16,44,60^ and SN 4D). In each half of the spin spectrum, a 3D QSHI carries the topology of a 3D QAHI, as indicated by the partial weak Chern numbers ${\nu }_{z}^{\pm }$ in **c** [see SN 4C3]. **d** The spin-Weyl-semimetal [DSTI^36^] regime of a helical HOTI, which is constructed by superposing two 3D TIs with gapless bulk spin spectra featuring chirally-charged nodal degeneracies that we term spin-Weyl points [red and blue circles in **d**, see SN 2E]. e The T-DAXI regime of a helical HOTI, which is constructed by superposing time-reversed spin-polarized magnetic AXIs. Each half of the spin spectrum in **e** is topologically equivalent to a magnetic AXI with an ${{{{{{{\mathcal{I}}}}}}}}$-quantized partial axion angle *θ*^±^ = *π*, implying a topological bulk spin-magnetoelectric response. The gapped surfaces of T-DAXIs bind anomalous halves of ${{{{{{{\mathcal{T}}}}}}}}$-invariant 2D TIs with odd spin Chern numbers *C*^*s*^—formed from summing anomalous surface states with half-integer partial Chern numbers *C*^±^ and ${{{{{{{\mathcal{T}}}}}}}}$-related spin-orbital textures—as a consequence of a novel 2D surface partial parity anomaly (SN 5E).

In particular, in 3D symmetry-protected topological crystalline insulators (TCIs), gapless boundary states only appear on 2D surfaces that preserve specific crystal symmetries, and the surface states on the remaining crystal facets are generically gapped^14–17^. The limitations of anomalous gapless boundary states as experimental signatures of bulk topological phases are further compounded in the class of 3D TCIs that have become known as higher-order TCIs (HOTIs), in which most—if not all—of the 2D surface states are gapped, and the 1D hinges (edges) between gapped surfaces bind gapless chiral or helical modes^18–22^. In HOTIs, the specific configuration of hinge states can provide an indicator of the bulk topology, but only in system geometries with unrealistically high symmetry (i.e. where the entire crystallite exhibits perfect point group symmetry)^23,24^ [see Fig. 1(a,b)]. For the subset of ${{{{{{{\mathcal{T}}}}}}}}$-broken HOTI phases with chiral hinge modes and relevant SOC, this issue has been overcome by recognizing that spinful chiral HOTIs are magnetic axion insulators (AXIs)^19,23,25,26^. Magnetic AXIs, like 3D TIs, are characterized by a bulk topological axion angle *θ* = *π* (where *θ* is defined modulo 2*π*)^27–30^, leading to a quantized **E** ⋅ **B** bulk magnetoelectric response and anomalous quantum Hall states with half-integer Chern numbers on gapped surfaces as a consequence of the 2D surface parity anomaly. Importantly, the quantized bulk magnetoelectric response of AXIs can be experimentally measured without invoking gapless boundary states. For example, the quantized value *θ* = *π* was measured (in the units of the fine-structure constant) through optical experiments performed on 3D TIs with magnetically gapped surface Dirac cones^31,32^.

Using the theoretical methods of Topological Quantum Chemistry^25,33,34^ and symmetry-based indicators (SIs)^35–37^, researchers have performed high-throughput^38–42^ and exhaustive^43^ searches for 3D topological materials, yielding thousands of candidate TIs and TCIs. These computational investigations have revealed candidate helical HOTI phases in readily accessible materials, including rhombohedral bismuth^20^, *α*-BiBr^44,45^, and the transition-metal dichalcogenides MoTe_2_ and WTe_2_^46^, in turn motivating experimental efforts to observe nontrivial topology in these materials^47–51^. However in HOTIs, the bulk topological spectral flow cannot be inferred by observing gapless surface states in photoemission experiments, because the surface states are gapped on most (if not all) 2D surfaces of 3D HOTI phases^18–22^. Experimental investigations of helical HOTIs have therefore instead largely focused on scanning-tunneling microscopy (STM) and ballistic supercurrent signatures of 1D helical “hinge” channels. Unfortunately, the configuration of hinge states in a given material is highly dependent on sample details [Fig. 1(b)], and hinge-state-like 1D gapless modes can also originate from crystal defects, or even manifest in materials that are topologically trivial in the bulk^45,52^. To better understand the existing experimental data and provide a road map for real-world applications of the topological materials identified in refs. ^38–43^, it is crucial to elucidate the geometry-independent bulk signatures of newly discovered helical TCI and HOTI phases, analogous to the characterization of chiral HOTIs as AXIs. Bulk topological invariants like the axion angle *θ* are typically robust to boundary details and disorder^53,54^, and hence predictive under more realistic material conditions.

In this work, we perform extensive numerical investigations to, for the first time, unravel the bulk and surface theories of helical HOTIs, and their connection to boundary-insensitive physical observables. Unlike 2D Chern insulators and 3D TIs, we find that the electromagnetic response of helical HOTIs does not depend solely on the electronic band topology, but also depends on additional details of the spin-orbital texture of the occupied bands. We start from the projected spin operator *PsP* introduced by Prodan in ref. ^55^, where *P* projects onto a set of occupied bands, and *s* is a choice of spin direction. Building on ref. ^55^ and the crystallographic splitting theorem of ref. ^56^, we show that topologically nontrivial ${{{{{{{\mathcal{T}}}}}}}}$-invariant insulators with relevant spin-orbit coupling (SOC) have topologically nontrivial spin-resolved bands. We theoretically introduce and numerically implement a gauge-invariant (nested) Wilson loop (non-Abelian Berry phase) method^16,17,23,57^ for computing crystal-symmetry-protected, spin-resolved band topology in ${{{{{{{\mathcal{T}}}}}}}}$-invariant insulators [see Sections 3 and 4 of the Supplementary Notes (SN 3 and 4)]. The extensive toy-model and real-material spin-resolved and nested Wilson loop calculations in this work (see SN 3, 4, 5, 8, 9, and 10) were performed using the freely accessible Python package NESTED_AND_SPIN_RESOLVED_WILSON_LOOP^58^, which was previously implemented and utilized for the preparation of refs. ^23,59^, and was then greatly refined and extended to spin-resolved calculations for the present work. In SN 2C, we crucially demonstrate that gaps in the spectrum of *PsP*, termed the spin spectrum, are perturbatively robust to deformations of the spin-orbital texture of the occupied bands, leading to a controlled notion of “spin-stable” band topology in which spin-resolved bulk topological invariants remain quantized under symmetric deformations that neither close an energy gap nor a spin gap in the *PsP* spectrum. To provide position-space physical intuition for our spin-resolved Wilson loop calculations, we introduce a spin-resolved layer construction method^25,34,37^ for enumerating and classifying 3D symmetry-protected, spin-gapped topological states. In SN 3H, we also introduce a formulation of a spin-resolved entanglement spectrum, which we prove to be homotopic to the spectrum of the spin-resolved Wilson loop.

Through our numerical calculations, we find that helical HOTIs necessarily fall into one of three regimes of spin-stable topology, each of which is characterized by a distinct spin-electromagnetic response. Previous studies have recognized that helical TCI phases may realize layered quantum spin Hall states in which each unit cell contributes a nonzero spin Hall conductivity^16,44,60^, and we accordingly find that some helical HOTIs for particular spin resolution directions *s* [such as *α*-BiBr for *s*_*z*_ spins, see SN 10B] realize these 3D quantum spin Hall insulator (QSHI) states [Fig. 1(c)]. However, we also discover two additional spin-stable regimes of ${{{{{{{\mathcal{I}}}}}}}}$- and ${{{{{{{\mathcal{T}}}}}}}}$-protected helical HOTIs, which are both physically distinguishable from each other, and from 3D QSHIs (SN 4D).

First, we find that helical HOTIs may exhibit (for some or all spin resolution directions) a gapless *PsP* spectrum in which the spin-gap closing points form “spin-Weyl fermions” that act as monopoles of spin-resolved partial Berry curvature^61^, a quantity that derives from the partial polarization (Berry phase) introduced by Fu and Kane in ref. ^62^ [see Fig. 1(d) and SN 2E, 3E, and 3F]. In SN 3E and 3F, we show that all 3D TI phases realize spin-resolved spin-Weyl states, and in SN 2G, we demonstrate that spin-Weyl points in the *PsP* spectrum can be converted into Weyl points in the energy spectrum by a strong Zeeman field, leading to the presence of topological Fermi-arc surface states. Through ab-initio calculations, we demonstrate in SN 9 that for all choices of *s* in *PsP*, the candidate HOTI *β*-MoTe_2_ realizes a spin-Weyl semimetal state. We further show that in the representative case of *s* ∝ *s*_*x*_ + *s*_*z*_, the spin gap of *β*-MoTe_2_ closes at only 8 spin-Weyl points, which give rise to Fermi arcs on the experimentally accessible (001)-surface under a strong $(\hat{{{{{{{{\bf{x}}}}}}}}}+\hat{{{{{{{{\bf{z}}}}}}}}})$-directed Zeeman field (see ref. ^46^ and SN 9C).

Most intriguingly, we discover a final regime for helical HOTIs in which the bands within each sector of *PsP* exhibit the same topology as a magnetic AXI. By applying nested Wilson-loop and hybrid-Wannier methods for computing *θ*^23,26^ to the spin spectrum of helical HOTIs, we specifically uncover the existence of a previously unrecognized ${{{{{{{\mathcal{T}}}}}}}}$-doubled AXI (T-DAXI) state characterized by bulk nontrivial partial axion angles *θ*^±^ = *π* [Fig. 1(e) and SN 4D and 4E]. We implement a spin-resolved variant of the local Chern marker^63,64^ to demonstrate that the gapped surfaces of T-DAXIs bind anomalous halves of 2D TI states as a consequence of a novel ${{{{{{{\mathcal{T}}}}}}}}$-invariant partial parity anomaly (SN 5E). The T-DAXI regime hence provides the first theoretical description of a helical HOTI that is free from the requirement of perfect global crystal symmetry: in T-DAXIs, ${{{{{{{\mathcal{I}}}}}}}}$ symmetry pins *θ*^±^ = *π* deep in the bulk leading to a topological contribution to the bulk spin-magnetoelectric response, and 1D helical modes appear on surface (and hinge) domain walls between gapped facets hosting topologically distinct halves of 2D TI states. Crucially, while the spin-Weyl semimetal, QSHI, and T-DAXI spin-resolutions of a helical HOTI can be deformed into each other by closing a spin gap without closing an energy gap, we will show below that they cannot be deformed into insulators with trivial spin-stable topology without closing an energy gap.

Lastly, we remarkably discover that the T-DAXI state—as well as the aforementioned 3D QSHI state—are both realized in the quasi-1D candidate HOTI *α*-BiBr. Through ab-initio calculations detailed in SN 10, we specifically find that *α*-BiBr hosts a spin gap for nearly all spin resolution directions, which interpolates between a wide 3D QSHI regime centered around *s*_*z*_ and a narrower (but still significant and numerically stable, see SN 10B) T-DAXI regime centered around *s*_*x*_. To provide physical signatures of the spin-gapped states in *α*-BiBr, we then in SN 10C use a Wannier-based tight-binding model to compute the bulk intrinsic contribution to the (non-quantized) spin Hall conductivity of *α*-BiBr (per unit cell) within its 3D QSHI and T-DAXI regimes. Our calculations reveal a highly anisotropic bulk spin Hall response in *α*-BiBr that is nearly quantized within the 3D QSHI regime (*s*_*z*_ spins) and nearly vanishing within the T-DAXI regime (*s*_*x*_ spins), in close agreement with the bulk spin-resolved topology (partial Chern numbers).

## Results

### The spin spectrum and spin-stable topology

**Fig. 2:**
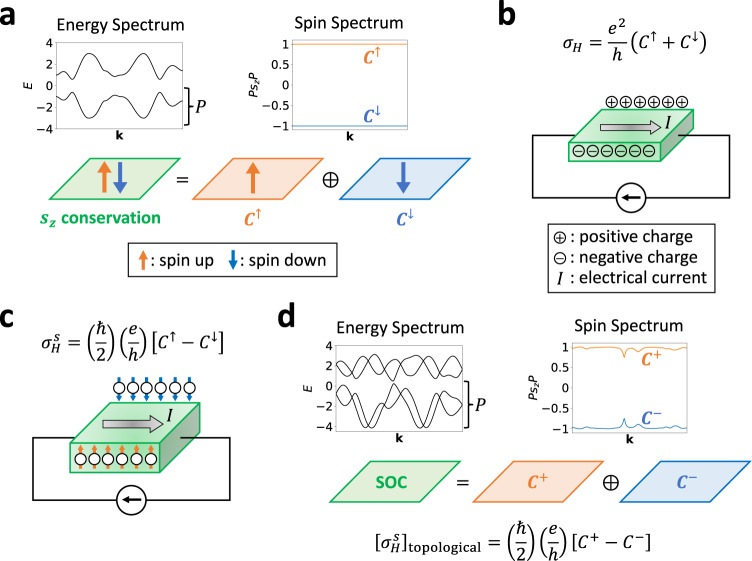
Spin-resolved band topology. **a** A 2D insulator with strong *s*_*z*_-preserving (e.g. “Ising”^66^ or “Kane-Mele-like”^2^) spin-orbit coupling (SOC). In **a**, separate Chern numbers *C*^*↑*,*↓*^ can be defined for the *s*_*z*_ = *↑*, *↓* occupied states. The sum *C*^*↑*^ + *C*^*↓*^ indicates the topological coefficient of **b** the Hall response [Eq. (1)], whereas the difference *C*^*↑*^ − *C*^*↓*^ indicates the topological coefficient of **c** the spin Hall response [Eq. (3)]^67,69,70^. **d** A 2D insulator with *s*_*z*_-breaking (e.g. Rashba^3^) SOC. Though the *s*_*z*_ spin Hall conductivity is no longer quantized in **d**, the existence of a topological contribution ${[{\sigma }_{H}^{s}]}_{{{{{{{{\rm{topological}}}}}}}}}$ to the (non-quantized) bulk *s*_*z*_ spin Hall response can still be inferred from the quantized partial Chern numbers *C*^±^ of spectrally isolated groupings of bands in the spin spectrum of the matrix *PsP* with *s* = *s*_*z*_^55^ [see Eq. (5) and SN 3C]. Crucially, perturbative deformations to the system correspond to perturbative deformations of the spin spectrum (SN 2C). This facilitates introducing a finer notion of spin-stable topological phases in which the spin-resolved band topology of the *PsP* spectrum indicates the existence of bulk topological contributions to (non-quantized) spin-electromagnetic response effects, which cannot be removed without closing gaps in the energy or spin spectra. For example, because the *s*_*z*_-nonconserving *Ps*_*z*_*P* spin spectrum in **d** is adiabatically related to the *s*_*z*_-conserving *Ps*_*z*_*P* spectrum in **a** without closing an energy or spin gap, then *C*^*↑*^ = *C*^+^ and *C*^*↓*^ = *C*^−^.

Spin-resolved band topology and its relationship to spin-electromagnetic response effects can most straightforwardly be understood in 2D insulators. To begin, we consider a 2D spinful (fermionic), noninteracting insulator lying in the *xy*-plane with *s* = *s*_*z*_ spin-rotation symmetry (i.e., U(1) spin symmetry, in addition to the U(1) charge conservation symmetry^65^). We emphasize that the simultaneous requirements of charge and *s*_*z*_ spin conservation symmetries do not require SOC to vanish, or to even be small. Instead, *s*_*z*_ symmetry only enforces that *s*_*z*_-nonconserving (e.g. Rashba^3^) contributions to the SOC vanish, whereas *s*_*z*_-conserving (e.g., “Ising”^66^ or “Kane-Mele-like”^2^) SOC may be arbitrarily large. In the band structure of the 2D insulator, each occupied Bloch eigenstate is an eigenstate of *s*_*z*_, allowing separate Berry connections, curvatures, and Chern numbers *C*^*↑*,*↓*^ to be defined for the occupied states [Fig. 2(a)]. The total (charge) Hall conductivity, which characterizes the transverse voltage generated under an applied current [Fig. 2(b)], is given by:

1 $${\sigma }_{H}=\frac{{e}^{2}}{h}C,$$

where *C* is the total Chern number:

2 $$C={C}^{\uparrow }+{C}^{\downarrow }.$$

Similarly, for each spin direction *s*, a separate spin Hall conductivity ${\sigma }_{H}^{s}$^67^ can be defined to characterize the transverse *s*-polarized spin separation generated under an applied current [Fig. 2(c)]. For the above example of an insulator with *s*_*z*_-conservation symmetry, the *s* = *s*_*z*_ spin Hall conductivity is given by:

3 $${\sigma }_{H}^{s}=\left(\frac{\hslash }{2}\right)\left(\frac{e}{h}\right)\left[{C}^{\uparrow }-{C}^{\downarrow }\right],$$

motivating the definition of a spin Chern number:

4 $${C}^{s}={C}^{\uparrow }-{C}^{\downarrow }.$$

2D insulators with *C* ≠ 0 are termed quantum Hall states^68^, and insulators with *C* = 0, *C*^*s*^ ≠ 0 are termed quantum spin Hall states^69,70^.

As crucially emphasized by Kane and Mele^2,3^, *s*_*z*_ symmetry is typically broken in real materials by the presence of multiple microscopic (e.g. simultaneous Ising and Rashba) contributions to SOC, because crystal and ${{{{{{{\mathcal{T}}}}}}}}$ symmetries alone cannot enforce a U(1) spin conservation symmetry, such as *s*_*z*_. Without *s*_*z*_ symmetry, the spin Hall conductivity is no longer quantized and cannot be computed through Eq. (3), because states can no longer by labeled by the spin eigenvalues *s*_*z*_ = *↑*, *↓*. However, it is known that as perturbatively weak *s*_*z*_-nonconserving SOC is introduced, the intrinsic bulk contribution to the spin Hall response does not instantaneously vanish, but instead remains perturbatively close to the value given by Eq. (3)^71^.

To deduce the existence of a bulk topological contribution to ${\sigma }_{H}^{s}$ for each spin direction $s={{{{{{{\bf{s}}}}}}}}\cdot \hat{{{{{{{{\bf{n}}}}}}}}}$, Prodan introduced the projected spin operator *PsP*^55^, which in this work represents a shorthand expression for the matrix:

5 $$PsP\equiv P({{{{{{{\bf{k}}}}}}}})\left({{{{{{{\bf{s}}}}}}}}\cdot \hat{{{{{{{{\bf{n}}}}}}}}}\right)P({{{{{{{\bf{k}}}}}}}}),$$

where *P*(**k**) is the projector onto an energetically isolated (typically occupied) set of electronic states at the crystal momentum **k** (see SN 2B). The eigenvalues of *PsP* are gauge invariant and as functions of **k** form the spin spectrum, a physically meaningful characterization of the occupied states that is complementary to the electronic structure [Fig. 2(d)]. When a given *s* is a conserved symmetry (whether or not *s*-preserving SOC is present), the eigenvalues of *PsP* in the occupied subspace are pinned to ± 1, and in insulators with compensated numbers of *s* = *↑*, *↓* electrons and negligible *s*-nonconserving SOC, the eigenvalues of *PsP* are separated at all **k** points by a spin gap Δ_*s*_ ≈ 2. Importantly, as *s*-nonconserving SOC is introduced and *s*-rotation [e.g., *s*_*z*_] symmetry relaxed, the eigenvalues of *PsP* do not fluctuate wildly, but instead, as shown in SN 2C, perturbatively deviate from ± 1. This can be contrasted with a similar quantity, the non-Abelian Wilson loop (holonomy) matrix computed in the direction of a reciprocal lattice vector **G**^16,17,23,57^:

6 $${{{{{{{{\mathcal{W}}}}}}}}}_{1,{{{{{{{\bf{k}}}}}}}},{{{{{{{\bf{G}}}}}}}}}=\mathop{\prod }\limits_{{{{{{{{\bf{q}}}}}}}}}^{{{{{{{{\bf{k}}}}}}}}+{{{{{{{\bf{G}}}}}}}}\leftarrow {{{{{{{\bf{k}}}}}}}}}P({{{{{{{\bf{q}}}}}}}}),$$

for which the (hybrid Wannier) spectrum need not adiabatically change under small perturbations to the system Hamiltonian, because ${{{{{{{{\mathcal{W}}}}}}}}}_{1,{{{{{{{\bf{k}}}}}}}},{{{{{{{\bf{G}}}}}}}}}$ is non-local in **k** (see SN 2C).

It was previously recognized in ref. ^55^ that even in a system without *s*_*z*_ symmetry, the Chern numbers of spectrally isolated bands in the *Ps*_*z*_*P* spin spectrum remain gauge-invariant quantities, and can be numerically computed. In particular, if there exists a spin gap between the spin bands with *Ps*_*z*_*P* eigenvalues closer to ± 1 [Fig. 2(d)], then one may compute the gauge-invariant, spin-resolved partial Chern numbers *C*^±^ of the bands within each half of the spin spectrum. The partial Chern numbers *C*^±^ importantly allow one to define the *s*_*z*_ spin Chern number^72^—even when *s*_*z*_ is no longer conserved—by generalizing Eq. (4) to the spin spectrum band topology:

7 $${C}^{s}={C}^{+}-{C}^{-}.$$

Though Eq. (7) no longer indicates the coefficient of a quantized spin Hall response away from the limit of *s*_*z*_ symmetry, *C*^*s*^ ≠ 0 still indicates the existence of a bulk topological contribution to ${\sigma }_{H}^{s}$ (for *s*_*z*_ spins). Furthermore, the intrinsic bulk spin Hall conductivity ${\sigma }_{H}^{s}$ may even lie close to the quantized topological value given by Eq. (3) if *s*_*z*_-breaking SOC is relatively weak (see SN 3C and 7B).

In an isolated 2D insulator with spinful ${{{{{{{\mathcal{T}}}}}}}}$ symmetry, *C*^+^ = − *C*^−^, such that *C* = 0, and ${C}^{s}\,{{{{{{{\rm{mod}}}}}}}}\,2=0$ for all choices of spin direction *s*. While *C*^*s*^ can be changed by 2 through spin band inversions between the upper and lower spin bands at a single **k** point in the spin spectrum, ${{{{{{{\mathcal{T}}}}}}}}$ symmetry enforces that spin band inversions occur in pairs at ± **k** or in crossings with quadratic dispersion at time-reversal-invariant **k** (TRIM) points, such that ${C}^{s}\,{{{{{{{\rm{mod}}}}}}}}\,4$ cannot change without closing a gap in the energy spectrum (see SN 3C and ref. ^55^). The spin spectrum hence also facilitates an alternative definition of the 2D ${{\mathbb{Z}}}_{2}$ invariant in ${{{{{{{\mathcal{T}}}}}}}}$-symmetric insulators:

8 $${z}_{2}=\left(\frac{{C}^{s}}{2}\right)\,{{{{{{{\rm{mod}}}}}}}}\,2,$$

for all *s* for which *PsP* exhibits a spin gap such that *C*^±^ (and hence *C*^*s*^) are well defined. Eq. (8) is consistent with the crystallographic splitting theorem of ref. ^56^, and further implies that *C*^*s*^ can still be nonzero in a ${{{{{{{\mathcal{T}}}}}}}}$-invariant insulator with *z*_2_ = 0. More generally, in SN 3G we show that a “fragile” TCI, which has a less robust form of topology than stable topological phases like 2D ${{\mathbb{Z}}}_{2}$ TIs^23,73^, can still carry a nonzero *C*^*s*^, and hence have a nonvanishing bulk topological contribution to its (non-quantized) spin Hall response. Our calculations in SN 4E also imply that spin bands in the *PsP* spectrum can exhibit a novel form of spin-resolved fragile topology. Lastly, unlike the 2D ${{\mathbb{Z}}}_{2}$ invariant^3^, *C*^*s*^ remains well-defined when ${{{{{{{\mathcal{T}}}}}}}}$ symmetry is broken (SN 3C). We will shortly exploit the robustness of *C*^*s*^ under ${{{{{{{\mathcal{T}}}}}}}}$-breaking potentials to analyze the topology of 3D insulators, in which **k**-space surfaces away from TRIM points can be treated as 2D systems with broken ${{{{{{{\mathcal{T}}}}}}}}$ symmetry^59^.

In this work, we more generally recognize the partial Chern numbers *C*^±^ to be members of a larger class of spin-resolved topological invariants that are stable to deformations that close neither an energy gap nor a spin gap. Given a 3D insulator that respects the symmetries of a nonmagnetic space group *G*, the spin bands specifically respect the symmetries of, and can carry topological invariants protected by, the magnetic space subgroup *M* ⊂ *G* for which each element *m* ∈ *M* commutes with the spin operator *s* in *PsP*. Building off of tremendous recent progress enumerating SIs and Wilson-loop indicators for spinful magnetic topological phases^23,25,34^, we resolve the spin-stable topology of 3D TIs and helical HOTIs by applying the existing magnetic topological classification to the spin bands of *PsP*. To compute spin-resolved topological invariants, we theoretically introduce and numerically implement spin-resolved generalizations of the (nested) Wilson loop matrix [Eq. (6), see SN 3B and 4B, as well as ref. ^58^]. We further introduce in SN 3H a spin-resolved generalization of the entanglement spectrum^57^, which we show to be homotopic to the spin-resolved Wilson spectrum. Using *PsP* and spin-resolved Wilson loops, we discover several previously unrecognized, experimentally detectable features of well-studied 3D insulators, including spin-Weyl points in 3D TIs and nontrivial partial axion angles in helical HOTIs, which we will explore in detail below.

### Spin-Weyl fermions in 3D TIs

3D TIs have previously been linked to spin-orbital textures through their anomalous Dirac-cone surface states. Previous experimental investigations have specifically shown that the surface states of 3D TIs exhibit helical spin textures^8,9^ and can efficiently convert charge current to magnetic spin torque^12,13^. In this work, we find that 3D TIs additionally exhibit unremovable bulk spin textures, which are revealed by analyzing the connectivity and topology of the spin bands in *PsP*. We specifically find that 3D TIs must carry gapless spin spectra for all choices of *s* in *PsP*. As we will show below, absent additional symmetries, the *PsP* spectrum of a 3D TI generically exhibits an odd number of Weyl-fermion-like touching points between the ± -sector spin bands in each half of the 3D BZ, where each 3D nodal point acts as a source or sink of partial Berry curvature (SN 2E, 3E, and 3F).

**Fig. 3:**
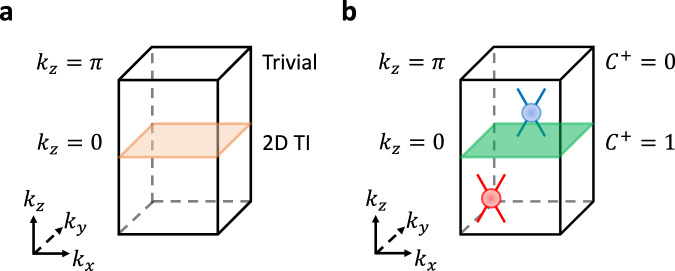
Spin-Weyl fermions in spin-resolved 3D topological insulators. **a** A ${{{{{{{\mathcal{T}}}}}}}}$-invariant 3D strong topological insulator (TI) in crystal momentum (**k**) space. The 3D TI in **a** can be re-expressed as a helical Thouless pump between a 2D TI (orange plane) and a trivial insulator^5,6,23,28^. Because a 3D TI is a strong, isotropic topological phase, then we may choose the pumping parameter in **a** to be *k*_*z*_ without loss of generality. **b** The *PsP* spin spectrum [Eq. (5)] of the 3D TI in **a** is gapless for all choices of $s={{{{{{{\bf{s}}}}}}}}\cdot \hat{{{{{{{{\bf{n}}}}}}}}}$ (e.g. *s*_*z*_). In each half of the 3D Brillouin zone (BZ) in **b**, the spin spectrum specifically exhibits Weyl-like^61^ nodal degeneracies with a net-odd partial chiral charge, which we term “spin-Weyl fermions” (see SN 2E, 3E, and 3F). In **b**, we show the simplest schematic example of a spin-resolved 3D TI [spin-Weyl state] with one positively (red) and one negatively (blue) charged spin-Weyl point in each half of the 3D BZ. The green plane in **b** indicates that the positive *PsP* bands at *k*_*z*_ = 0 carry a nontrivial odd partial Chern number *C*^+^ = 1 [originating from spin-resolving the 2D TI bands at *k*_*z*_ = 0 in **a**], which stands in contrast to the trivial partial Chern number *C*^+^ = 0 at *k*_*z*_ = *π*. The $\Delta {C}^{+}\,{{{{{{{\rm{mod}}}}}}}}\,2=1$ difference in partial Chern numbers between *k*_*z*_ = 0, *π* in **b**, combined with the continued validity of the *PsP* calculation in BZ planes without ${{{{{{{\mathcal{T}}}}}}}}$ symmetry away from *k*_*z*_ = 0, *π*^55^, indicates the presence of an odd number of integer-charge spin-Weyl fermions per half BZ. Spin-Weyl states like those in 3D TIs exhibit topological surface Fermi arcs under strong Zeeman fields (SN 2G and 9C), and display arc-like states along the entanglement cut in the spin-resolved entanglement spectrum (SN 3H). Further theoretical and numerical calculations demonstrating unremovable spin-Weyl points in 3D TIs are provided in SN 2E, 3E, and 3F.

To see that 3D TIs for all *s* must exhibit gapless *PsP* spectra featuring nodal degeneracies with nontrivial chiral charge—which we term “spin-Weyl” points—we first note that the momentum-space band structure of a 3D TI can be re-expressed as a helical Thouless pump of a 2D TI^5,6,23,28^. Taking *k*_*z*_ to be equivalent to the Thouless pumping parameter, the occupied bands in one ${{{{{{{\mathcal{T}}}}}}}}$-invariant BZ plane must be equivalent to a 2D TI [*k*_*z*_ = 0 in Fig. 3(a)], and must be equivalent to a 2D trivial insulator in the other ${{{{{{{\mathcal{T}}}}}}}}$-invariant, *k*_*z*_-indexed BZ plane [*k*_*z*_ = *π* in Fig. 3(a)]. Through Eqs. (7) and (8) and the constraint from ${{{{{{{\mathcal{T}}}}}}}}$ symmetry that *C*^+^ = − *C*^−^ (SN 3C), this implies that for the occupied bands of the 3D TI in Fig. 3:

9 $${C}^{\pm }\,{{{{{{{\rm{mod}}}}}}}}\,2=1\,{{{{{{{\rm{at}}}}}}}}\,{k}_{z}=0,$$

and:

10 $${C}^{\pm }\,{{{{{{{\rm{mod}}}}}}}}\,2=0\,{{{{{{{\rm{at}}}}}}}}\,{k}_{z}=\pi,$$

for all choices of *s* in *PsP*.

Crucially, unlike the ${{\mathbb{Z}}}_{2}$ invariant for 2D TIs, the partial Chern numbers *C*^±^ remain well-defined when ${{{{{{{\mathcal{T}}}}}}}}$ is broken in 2D BZ planes away from *k*_*z*_ = 0, *π*^55^. Eqs. (9) and (10) hence imply that *C*^±^ must each change by odd numbers across each half of the 3D BZ, which can only occur if the ± -sector spin bands in the spin spectrum meet in nodal degeneracies with nontrivial (partial) chiral charges [Fig. 3(b)]. Absent additional symmetries, nodal degeneracies with nontrivial chiral charge manifest as 3D conventional Weyl fermions with charge ± 1^1,61^. We therefore, in this work, refer to nodal points in the *PsP* spectrum with nontrivial partial chiral charges as spin-Weyl fermions, such that a spin-resolved 3D TI realizes a spin-Weyl semimetal phase.

Because a maximally spin-gapped *PsP* spectrum indicates the absence of *s*-nonconserving spin texture in the occupied bands (SN 2B), then the existence of unavoidable spin-Weyl points in 3D TIs implies that the occupied bands exhibit an unremovable spin texture. Like a Weyl semimetal state, a spin-Weyl semimetal state also exhibits forms of topological surface Fermi arcs. In the spin-Weyl state, the spin Fermi arcs either manifest as arc-like states along the entanglement cut in the spin-resolved entanglement spectrum (SN 3H), or as topological surface Fermi arcs in the energy spectrum under a large external Zeeman field, which we will explore in greater detail in the Experimental Signatures and Discussion section. Lastly, because the occupied energy bands in a portion of the BZ in a spin-Weyl state must necessarily exhibit *C*^*s*^ ≠ 0, then a finite sample of a spin-Weyl state, such as a 3D TI, may exhibit an extensive (though non-quantized) spin Hall conductivity.

### Partial axion angles in helical HOTIs

Having deduced the spin-resolved topology of 2D and 3D TIs, we will next analyze the spin-resolved topology and response of helical HOTIs. In studies to date, there exist three competing theoretical constructions of an ${{{{{{{\mathcal{I}}}}}}}}$- and ${{{{{{{\mathcal{T}}}}}}}}$-protected helical HOTI state:

1. Orbital-double (superpose two identical copies of) an ${{{{{{{\mathcal{I}}}}}}}}$- and ${{{{{{{\mathcal{T}}}}}}}}$-symmetric 3D TI to form a so-called “doubled-strong TI” (DSTI)^36^.
2. Stack ${{{{{{{\mathcal{I}}}}}}}}$- and ${{{{{{{\mathcal{T}}}}}}}}$-symmetric 2D TIs with the same spin-orbital textures to form a layer construction with two identical 2D TIs per cell separated by a half-lattice translation^25,34,37^.
3. ${{{{{{{\mathcal{T}}}}}}}}$-double (superpose two time-reversed copies of) an ${{{{{{{\mathcal{I}}}}}}}}$-symmetric magnetic AXI^23,46^.

As we will show below, the three constructions of a helical HOTI in fact represent families of spin-resolved states with distinct spin-stable topology and distinct physical signatures. In order, the three constructions above correspond to a spin-Weyl semimetal with an even number of spin-Weyl points per half BZ [Fig. 1(d)], a 3D QSHI state [Fig. 1(c)], and a T-DAXI state [Fig. 1(e)].

This result can most succinctly be understood through the language of SIs. An ${{{{{{{\mathcal{I}}}}}}}}$- and ${{{{{{{\mathcal{T}}}}}}}}$-symmetric HOTI is characterized by vanishing weak SIs and a nonvanishing ${{\mathbb{Z}}}_{4}$-valued strong SI *z*_4_ = 2, where *z*_4_ is defined by promoting the ${{\mathbb{Z}}}_{2}$-valued strong Fu-Kane parity (${{{{{{{\mathcal{I}}}}}}}}$) criterion for 3D TIs to a ${{\mathbb{Z}}}_{4}$ invariant that further distinguishes between uninverted and doubly inverted bands^6,22,25,34,36,37,46^:

11 $${z}_{4}=\frac{1}{4}\mathop{\sum}\limits_{{{{{{{{{\bf{k}}}}}}}}}_{a}\in {{{{{{{\rm{TRIMs}}}}}}}}}\left({n}_{+}^{a}-{n}_{-}^{a}\right)\,{{{{{{{\rm{mod}}}}}}}}\,4,$$

where ${n}_{+}^{a}$ (${n}_{-}^{a}$) is the number of occupied Bloch states at the TRIM point **k**_*a*_ with positive (negative) parity eigenvalues. Eq. (11) was originally obtained by performing combinatorics on the elementary (trivial) bands allowed in the nonmagnetic Shubnikov space group (SSG) $P\bar{1}{1}^{{\prime} }$ (# 2.5), which is generated by ${{{{{{{\mathcal{I}}}}}}}}$, ${{{{{{{\mathcal{T}}}}}}}}$, and 3D lattice translation symmetries^22,25,34,36,37,46^.

Importantly, when spin-resolving an insulator with the symmetries of SSG $P\bar{1}{1}^{{\prime} }$ (# 2.5), for any choice of spin direction *s* in *PsP* [Eq. (5)], the spin bands will respect the symmetries of magnetic SSG $P\bar{1}$ (# 2.4), which is the subgroup of SSG $P\bar{1}{1}^{{\prime} }$ (# 2.5) generated by breaking ${{{{{{{\mathcal{T}}}}}}}}$ while preserving ${{{{{{{\mathcal{I}}}}}}}}$ and 3D lattice translations^25,34^. This can be seen by recognizing that $\{s,{{{{{{{\mathcal{T}}}}}}}}\}=0$ and $[s,{{{{{{{\mathcal{I}}}}}}}}]=0$ for all possible spin resolution directions *s*. Because *PsP* splits the occupied bands in a ${{{{{{{\mathcal{T}}}}}}}}$-invariant insulator into halves, then the spin bands in each of the ± spin sectors will therefore each inherit half of the parity eigenvalues of the occupied bands of the original ${{{{{{{\mathcal{T}}}}}}}}$-invariant insulator (see SN 4D).

In magnetic SSG $P\bar{1}$ (# 2.4) there is also a ${{\mathbb{Z}}}_{4}$-valued strong SI:

12 $${\tilde{z}}_{4}=\frac{1}{2}\mathop{\sum}\limits_{{{{{{{{{\bf{k}}}}}}}}}_{a}\in {{{{{{{\rm{TRIMs}}}}}}}}}\left({n}_{+}^{a}-{n}_{-}^{a}\right)\,{{{{{{{\rm{mod}}}}}}}}\,4,$$

in which the prefactor of 1/2 differs from the prefactor of 1/4 in Eq. (11) because spinful ${{{{{{{\mathcal{T}}}}}}}}$ symmetry forces states to form Kramers pairs at the TRIM points in nonmagnetic SSG $P\bar{1}{1}^{{\prime} }$ (# 2.5). For a helical HOTI with *z*_4_ = 2, the spin bands in each ± sector will therefore carry the partial SI ${\tilde{z}}_{4}=2$. In magnetic SSG $P\bar{1}$ (# 2.4) (see SN 4D and refs. ^25,34^), ${\tilde{z}}_{4}=2$ can indicate Weyl-semimetal states with even numbers of Weyl points in each half BZ, 3D quantum anomalous Hall states, and AXI states—exactly in correspondence with the possible spin-resolved topological states of a helical HOTI [Fig. 1(c-e)]. Importantly, it is possible for *s*-nonconserving SOC to drive spin band inversions and change the spin-stable topology without closing an energy gap. However, because the ± -sector spin bands are related by ${{{{{{{\mathcal{T}}}}}}}}$ (SN 2B), and because $[{{{{{{{\mathcal{T}}}}}}}},{{{{{{{\mathcal{I}}}}}}}}]=0$, then a spin band inversion unaccompanied by an energy band inversion cannot change the value of ${\tilde{z}}_{4}$, and therefore cannot trivialize the spin-resolved topology of a helical HOTI. This represents the 3D generalization of the statement that the spin Chern number *C*^*s*^ of a 2D TI can be changed without closing an energy gap, but cannot go to zero without closing an energy gap or breaking ${{{{{{{\mathcal{T}}}}}}}}$ symmetry [i.e.$({C}^{s}/2)\,{{{{{{{\rm{mod}}}}}}}}\,2=1$ for all *s* in a 2D TI state, see ref. ^55^ and the text preceding Eq. (8)].

Having established the spin-resolved partial SIs of a helical HOTI, we will now more closely analyze each family of spin-stable topological states in its spin resolution. Earlier, we showed that a spin-resolved 3D TI for all *s* necessarily has an odd number of spin-Weyl points in each half of the BZ, absent symmetries beyond ${{{{{{{\mathcal{I}}}}}}}}$ and ${{{{{{{\mathcal{T}}}}}}}}$ [Fig. 3(b) and SN 3E and 3F]. Building on this result, because the DSTI construction of a helical HOTI consists of superposing (orbital-doubling) two identical 3D TIs^36^, it follows that a spin-resolved DSTI realizes for all *s* a spin-Weyl semimetal state with an even number of spin-Weyl points per half BZ [Fig. 1(d)]. Like an energy-band Weyl semimetal state, a spin-Weyl state also exhibits topological Fermi arcs, which can be detected in the spin-resolved entanglement spectrum (SN 3H), or in the surface energy spectrum in the presence of a large Zeeman field (SN 2G). We will shortly demonstrate in the Experimental Signatures and Discussion section that the candidate helical HOTI *β*-MoTe_2_^41,46^ realizes a spin-Weyl semimetal state with an even number of spin-Weyl points per half BZ for all choices of spin direction *s* in *PsP*, and hence lies in the DSTI regime of a helical HOTI (see SN 9B for further calculation details).

**Fig. 4:**
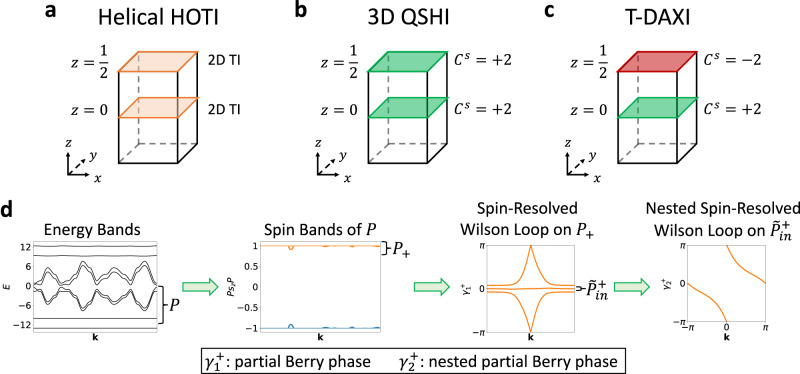
Spin-resolved layer constructions and bulk partial axion angles in ${{{{{{{\mathcal{T}}}}}}}}$-doubled AXIs. **a** The layer construction of an ${{{{{{{\mathcal{I}}}}}}}}$- and ${{{{{{{\mathcal{T}}}}}}}}$-symmetric helical HOTI^25,34,37^. The HOTI in **a** is theoretically constructed by placing ${{{{{{{\mathcal{I}}}}}}}}$- and ${{{{{{{\mathcal{T}}}}}}}}$-symmetric 2D TIs (orange rectangles) in the ${{{{{{{\mathcal{I}}}}}}}}$-invariant *z* = 0 and *z* = 1/2 real-space planes in each unit cell. **b**, **c** Spin-resolved layer constructions with electronic bands that are topologically equivalent to the HOTI in **a**. Specifically if each 2D TI in **a** carries a bulk *s*_*z*_ spin gap [where we have chosen *s* = *s*_*z*_ for concreteness, see Fig. 2d], there are two ways to spin-resolve the helical HOTI layer construction in **a** while keeping a spin gap open. If both 2D TI layers have the same partial Chern numbers, then **b** through Eq. (7), each 2D TI layer carries the same even-integer *s*_*z*_ spin Chern number ${C}^{s}\,{{{{{{{\rm{mod}}}}}}}}\,4=2$ [Eq. (8)], resulting in a 3D QSHI state with a non-quantized (but generically nonvanishing) *s*_*z*_ spin Hall conductivity per bulk unit cell. However if the 2D TI layers in **a** have oppositely signed partial Chern numbers that are identical in magnitude, **c** the system instead realizes a T-DAXI state with a vanishing bulk *s*_*z*_ spin Chern number and ${{{{{{{\mathcal{I}}}}}}}}$-quantized nontrivial partial axion angles *θ*^±^ = *π* (SN 4E). By closing and reopening the *Ps*_*z*_*P* spin gap, the QSHI insulator in **b** can be deformed into the T-DAXI in **c** via an intermediate spin-Weyl semimetal regime. Crucially, this deformation—which changes the bulk topological contribution to the spin-electromagnetic response for *s*_*z*_ spins—must close an *s*_*z*_ spin gap, but need not close an energy gap. **d** Numerical workflow employed in this study to compute *θ*^±^. We specifically extract *θ*^±^ by theoretically elucidating and numerically implementing a spin-resolved generalization of the nested Wilson loop method for computing *θ* that was previously introduced in ref. ^23^. Documentation and details for accessing our freely available (spin-resolved) nested Wilson loop code are provided in SN 4E and 10B and ref. ^58^.

We will next consider two cases of spin-stable resolutions of helical HOTIs that can be formally expressed using a spin-resolved variant of the layer construction method for enumerating and analyzing symmetry-protected topological states^25,34,37^. Given an SSG, a symmetry-protected topological state is considered to be layer-constructable if its momentum-space band topology can be completely captured in a system composed of flat, parallel layers of lower-dimensional topological states that are placed a manner in which their boundary states are pairwise gapped while preserving all system symmetries. For ${{{{{{{\mathcal{T}}}}}}}}$-invariant 3D TCI phases, the building blocks of layer constructions are 2D TIs and mirror TCIs^25,34,37^. In nonmagnetic SSG $P\bar{1}{1}^{{\prime} }$ (# 2.5) the layer construction of a helical HOTI consists of one ${{{{{{{\mathcal{I}}}}}}}}$-symmetric 2D TI at the origin of the unit cell [the ${{{{{{{\mathcal{I}}}}}}}}$-invariant *z* = 0 plane in Fig. 4(a)] and one ${{{{{{{\mathcal{I}}}}}}}}$-symmetric 2D TI in a real-space plane separated by a half-lattice translation from the origin [the ${{{{{{{\mathcal{I}}}}}}}}$-invariant *z* = 1/2 plane in Fig. 4(a)].

In this work, we introduce a finer distinction for layer constructions in which the spin-orbital textures of the layers, and hence their spin-resolved topology, become additional knobs in the layer construction method. To formulate these spin-resolved layer constructions, we begin by considering a 3D SSG that additionally carries at least one conserved spin direction *s* at all points in space (e.g., *s* = *s*_*z*_ symmetry). Formally, the full symmetry group of SSG symmetries and at least U(1) spin symmetry is isomorphic to a (nonmagnetic) “spin space group”^74^. The spin space groups are generally suitable for classifying the symmetry and topology of spin-wave excitations (magnons), for which minimal models represent a realistic approximation. However, the spin space groups are largely unsuitable for characterizing the electronic structure of solid-state materials, in which perfect spin-rotation symmetries are broken by phenomenologically distinct, symmetry-allowed contributions to the SOC, such as Ising and Rashba potentials^3,25,34,66^. With this in mind, we next introduce *s*-nonconserving SOC to break the conserved spin symmetry, but not in a manner strong enough to close a spin gap within any of the system layers. Hence, we may still classify the layer construction using the partial Chern numbers *C*^±^ of the occupied bands within each layer.

The simplest spin-resolved layer construction of a helical HOTI is one in which each 2D TI layer is spin-gapped for a spin direction *s* and carries the same spin-orbital texture, such that the ± -sector spin bands within each layer carry the same partial Chern numbers [Fig. 4(b)]. Through the definition of the 2D ${{\mathbb{Z}}}_{2}$ invariant in Eq. (8), this implies that each layer carries the same spin Chern number satisfying ${C}^{s}\,{{{{{{{\rm{mod}}}}}}}}\,4=2$. Because each unit cell carries a non-vanishing spin Chern number, then a helical HOTI constructed from identical 2D TI layers realizes a 3D QSHI state with an intrinsic bulk spin Hall response per unit cell that is nonvanishing (though also generically non-quantized due to the presence of *s*-nonconserving SOC). 3D QSHI states were previously predicted in the hourglass TCI KHgSb^16^, distorted square-net compounds^60^, and in the helical HOTI *α*-BiBr^44^. Indeed, our spin-resolved topological analysis of *α*-BiBr, detailed below and in SN 10, reveals that *α*-BiBr realizes a 3D QSHI state with a large bulk spin gap over a wide range of spin resolution directions.

However in this work, we recognize the existence of a second possible spin-resolved layer construction of an ${{{{{{{\mathcal{I}}}}}}}}$- and ${{{{{{{\mathcal{T}}}}}}}}$-symmetric helical HOTI. Instead of placing spin-gapped (for a spin direction *s*) 2D TI layers with the same partial Chern numbers in each ${{{{{{{\mathcal{I}}}}}}}}$-invariant plane, we alternatingly place layers with oppositely signed odd partial Chern numbers that are identical in magnitude [Fig. 4(c)]. In this case, the total spin Chern number within each unit cell vanishes. However, this does not imply a trivial spin-electromagnetic response. Instead we recognize that per ± spin sector, the layer construction in Fig. 4(c) is identical to that of an ${{{{{{{\mathcal{I}}}}}}}}$-protected magnetic AXI (see Supplementary Figure 19 and refs. ^25,26,34^). This implies that taken per ± sector (which reduce to the *↑*, *↓* spin sectors in the limit of perfect *s* spin-rotation symmetry), the system carries an ${{{{{{{\mathcal{I}}}}}}}}$-quantized partial axion angle *θ*^±^ = *π*, even though the total (charge) axion angle is trivial $\theta \,{{{{{{{\rm{mod}}}}}}}}\,2\pi=0$. Unlike the standard axion angle *θ*, which can be quantized by either ${{{{{{{\mathcal{I}}}}}}}}$ or ${{{{{{{\mathcal{T}}}}}}}}$, the partial axion angles *θ*^±^ are quantized by ${{{{{{{\mathcal{I}}}}}}}}$ and exchanged (with a relative sign) by the action of ${{{{{{{\mathcal{T}}}}}}}}$ (*θ*^±^ → − *θ*^∓^ under ${{{{{{{\mathcal{T}}}}}}}}$, see SN 4D). In the same sense that the standard axion angle *θ* represents the 3D generalization of the 1D Berry phase (charge polarization)^28–30^, the partial axion angles *θ*^±^ therefore represent the 3D generalizations of the 1D partial Berry phases (polarization) introduced by Fu and Kane in ref. ^62^. We term the new spin-stable state characterized by *θ*^±^ = *π* the T-DAXI regime of a helical HOTI. To numerically verify the existence of ${{{{{{{\mathcal{I}}}}}}}}$-quantized partial axion angles in the T-DAXI state, we applied the nested Wilson loop method for computing *θ* previously introduced in ref. ^23^ to the spin spectrum of a modified (*s*_*z*_-nonconserving) implementation of the helical HOTI model formulated in ref. ^46^ (see also ref. ^58^). As shown in Fig. 4d and documented in SN 4E, the ${{{{{{{\mathcal{I}}}}}}}}$-symmetric nested spin-resolved Wilson spectrum exhibits odd chiral winding, indicating that *θ*^±^ = *π*. Through extensive nested spin-resolved Wilson loop calculations (see SN 10B), we find that the candidate helical HOTI *α*-BiBr realizes not only the aforementioned 3D QSHI state, but also the T-DAXI state introduced in this work.

In 3D AXIs, the bulk axion angle *θ* = *π* also has a deep relation to the physics and response of 2D surfaces. Specifically in isolated 2D systems, the parity anomaly dictates that there cannot exist an odd number of symmetry-stabilized twofold Dirac cones^17,28,68^. However the parity anomaly is circumvented on 2D interfaces (domain walls) between 3D insulators with *θ* = *π* (e.g. 3D TIs) and insulators with *θ* = 0 (typically the vacuum). Under the preservation of specific surface (interface) symmetries (such as ${{{{{{{\mathcal{T}}}}}}}}$), this leads to an odd number of symmetry-stabilized surface Dirac cones^5,6,23,26^.

**Fig. 5:**
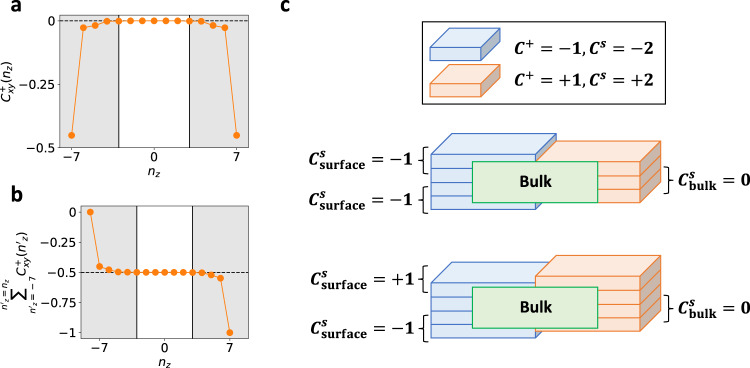
Surface partial parity anomaly in ${{{{{{{\mathcal{T}}}}}}}}$-doubled axion insulators. **a** The layer-resolved position-space partial Chern number ${C}_{xy}^{+}({n}_{z})$ for *s* = *s*_*z*_ spins of an ${{{{{{{\mathcal{I}}}}}}}}$-symmetric finite slab of the *s*_*z*_-nonconserving T-DAXI model from Fig. 4(d) [adapted from ref. ^46^, see SN 5E], plotted as a function of the *z*-direction slab layer index *n*_*z*_. **b** The cumulative (summed) values of ${C}_{xy}^{+}({n}_{z})$ in **a**. In a T-DAXI, ${C}_{xy}^{+}({n}_{z})$ is zero in the bulk of the system [white region in **a**, **b**] and nonvanishing on gapped surfaces [shaded regions in **a**, **b**]. However on each T-DAXI surface, we observe a cumulative half-integer partial Chern number [specifically *C*^+^ = − 0.5 in **a**,**b**]. Because isolated ${{{{{{{\mathcal{T}}}}}}}}$-invariant noninteracting 2D insulators can only carry even spin Chern numbers (and hence integer partial Chern numbers via *C*^*s*^ = *C*^+^ − *C*^−^ = 2*C*^+^)^75^ and because ${C}^{+}\,{{{{{{{\rm{mod}}}}}}}}\,2=1$ in 2D TIs^55^, the data in **a**,**b** indicate that the ${{{{{{{\mathcal{T}}}}}}}}$-invariant gapped surfaces of T-DAXIs are not trivial, but rather carry anomalous halves of 2D TI states in a realization of a novel partial parity anomaly (SN 4D3). Importantly, perfect global ${{{{{{{\mathcal{I}}}}}}}}$ symmetry is not required to quantize *θ*^±^ = *π* in the bulk and realize anomalous surface halves of 2D TI states. To illustrate this, in **c** we show schematic layer constructions of a finite T-DAXI slab. [**c**, upper schematic] An ${{{{{{{\mathcal{I}}}}}}}}$-symmetric slab corresponding to the partial Chern number distribution in **a**, **b**. [**c**, lower schematic] The T-DAXI slab from the upper panel in **c**. Adding an extra (non-anomalous) layer with *C*^*s*^ = 2 (*C*^+^ = 1) to the top surface of the system breaks global ${{{{{{{\mathcal{I}}}}}}}}$ symmetry, yielding a slab with a vanishing total spin Chern number. However because each surface still carries an anomalous half of a 2D TI, each surface under an applied magnetic field still exhibits an intrinsic (non-quantized) spin Hall response unaccompanied by a bulk response, resulting overall in a 3D spin-magnetoelectric effect (see SN 7C and refs. ^24,80^).

However, if the 2D surface does not preserve enough symmetries, then it becomes gapped. Crucially, this does not imply that the 2D surface is trivial. As the low-energy 2D surface theory of a 3D *θ* = *π* phase originates from an unpaired, parity-anomaly-violating twofold Dirac cone (integer quantum Hall critical point), then the 2D surface of a 3D TI or AXI, when gapped, realizes an anomalous (noninteracting) half quantum Hall state^6,28^. In helical HOTIs, the gapped 2D surfaces are ${{{{{{{\mathcal{T}}}}}}}}$-invariant, and hence have vanishing Hall conductivities [see the text preceding Eq. (8)]. One might therefore believe that the gapped 2D surfaces of helical HOTIs are trivial, or possibly carry integer 2D TI states, because a portion of the edges (hinges) between gapped HOTI surfaces exhibit 1D helical modes [Fig. 1(a,b)]. However, our discovery of ${{{{{{{\mathcal{I}}}}}}}}$-quantized bulk partial axion angles *θ*^±^ = *π* in the T-DAXI state suggests that instead, each partial axion angle contributes a half-integer partial Chern number to each gapped 2D surface. This implies that each 2D surface of a T-DAXI (with *θ*^±^ = *π* obtained for a fixed spin direction *s*) hosts a ${{{{{{{\mathcal{T}}}}}}}}$-invariant gapped state with an odd spin Chern number (${C}^{s}\,{{{{{{{\rm{mod}}}}}}}}\,2=1$), a value that cannot be realized in an isolated ${{{{{{{\mathcal{T}}}}}}}}$-invariant noninteracting insulator with a spin gap (see ref. ^75^ and SN 3C). Each gapped surface of a T-DAXI is hence equivalent to an anomalous half of an isolated 2D TI as a consequence of a novel partial parity anomaly. To numerically verify the existence of a surface partial parity anomaly in T-DAXIs, we implemented a spin-resolved partial variant of the position-space layer-resolved Chern number^29,30,76^ (see SN 5 for calculation details). As shown in Fig. 5a, the layer-resolved partial Chern number vanishes on the average in the bulk of a T-DAXI, and indeed saturates at anomalous half-integer values on its gapped surfaces.

We can draw several connections between the anomalous surfaces of T-DAXIs and previous works. First, anomalous halves of 2D TI states were previously predicted to occur on the top and bottom surfaces of weak TIs^77^; here, we recognize anomalous half 2D TI states to be more general features of helical HOTIs in the T-DAXI regime. Though T-DAXIs and globally ${{{{{{{\mathcal{I}}}}}}}}$-symmetric models of spin-gapped weak TIs (with odd total numbers of 2D TI layers) both exhibit anomalous surface half 2D TI states, they are still distinguishable via bulk spin Hall measurements, provided that the intrinsic spin Hall response is dominated by the bulk topological contribution. Specifically, in weak TIs with a gap in *PsP* for a spin direction *s*, the topological contribution to the spin Hall conductance of a finite sample (for *s*-polarized spins) is extensive and carries a nonvanishing weight in each bulk unit cell. Conversely in a T-DAXI with a *PsP* gap for a spin direction *s*, the topological contribution to the spin Hall conductivity vanishes in the bulk and only manifests (anomalously) on 2D surfaces, and is hence independent of sample thickness [Fig. 5(a,b)]. A magnetic field applied to a T-DAXI will therefore induce a (non-quantized) spin Hall response (for *s*-polarized spins) on spatially separated (opposing) surfaces. If the spin Hall responses on the opposing surfaces are oppositely signed [which necessarily breaks global ${{{{{{{\mathcal{I}}}}}}}}$ symmetry, see Fig. 5(c) and ref. ^31^], the magnetic field will generate a spin separation with both a transverse and a parallel component with respect to the field. We term the novel response originating from the field-parallel spin separation the 3D spin-magnetoelectric effect. As we have only demonstrated the existence of the spin-magnetoelectric effect through layering and Thouless-pump arguments (SN 3G and 4D), a linear-response formulation of the spin-magnetoelectric effect in the presence of *s*-nonconserving SOC remains an exciting and urgent direction for future study.

The anomalous odd spin Chern number of the gapped surfaces of the T-DAXI state is also reminiscent of 3D bosonic TIs, for which each 2D surface carries an odd Chern number, a value that is anomalous because isolated 2D bosonic systems without topological order are required to have even Chern numbers^78,79^. Additionally, 3D symmetry-protected topological phases with anomalous 2D quantum spin Hall surface responses have been proposed in field-theoretic investigations, but were not previously associated to helical HOTIs^80^. Lastly, the gapless surface theories of other ${{{{{{{\mathcal{T}}}}}}}}$-invariant 3D TCI phases, like twofold-rotation-anomaly TCIs (two twofold Dirac cones)^21,25,34^ and the nonsymmorphic Dirac insulator (one fourfold Dirac cone)^17^ can be deformed into the gapped surface theory of a helical HOTI by lowering the surface crystal symmetry without breaking ${{{{{{{\mathcal{T}}}}}}}}$. This suggests that the symmetry-enhanced fermion doubling theorems circumvented in these TCI phases, which were deduced from crystal-symmetry constraints on band connectivity, may be expressible in the language of quantum field theory through the partial parity anomaly identified in this work.

## Discussion

**Fig. 6:**
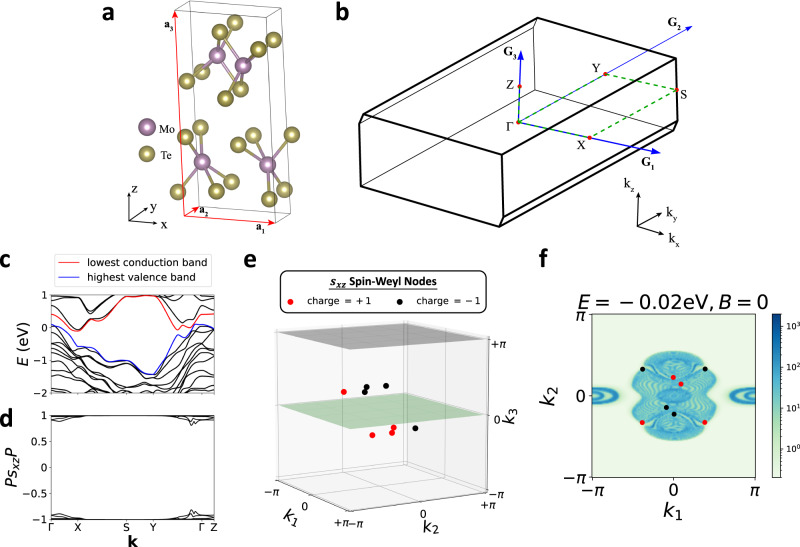
Spin-Weyl points in *β*-MoTe_2_. **a** Crystal structure of the candidate helical HOTI *β*-MoTe_2_^41,46^, which respects the symmetries of Shubnikov space group (SSG) $P{2}_{1}/m{1}^{{\prime} }$ (# 11.51, see SN 9A). The red arrows in **a** indicate the primitive lattice vectors **a**_1,2,3_. **b** The bulk BZ of *β*-MoTe_2_. The blue arrows in **b** indicate the primitive reciprocal lattice vectors **G**_1,2,3_. **c** Band structure of a first-principles- (DFT-) obtained, symmetric, Wannier-based tight-binding model of *β*-MoTe_2_ (details provided in SN 9A), plotted along the dashed green high-symmetry **k**-path in **b**. In **c**, we label the highest valence [lowest conduction] doubly-degenerate bands in blue [red]. **d** The *Ps*_*xz*_*P* spin spectrum of the occupied bands of *β*-MoTe_2_ [choosing all states to be individually occupied up to and including the blue bands in **c** at each **k** point, see SN 9A]. Though the spin spectrum in **d** appears gapped, closer examination of the spin gap away from high-symmetry BZ lines reveals the presence of spin-Weyl points in the BZ interior. We further find that for all choices of spin direction *s* in *PsP* [Eq. (5)], *β*-MoTe_2_ realizes a spin-Weyl state with an even number of spin-Weyl nodes per half BZ (SN 9B). **e** The distribution of spin-Weyl nodes for the *s* = *s*_*xz*_ [Eq. (13)] spin spectrum in **d**. In **e**, there are eight spin-Weyl nodes in the BZ interior with a total partial chiral charge of ∣2∣ per *k*_3_-indexed half BZ, which we have confirmed through spin-resolved Wilson loop calculations (SN 9B). **f** The (001)-surface spectral function of *β*-MoTe_2_ with the projected locations and partial chiral charges of the bulk *s*_*xz*_ spin-Weyl points from **e** labeled with red and black circles.

We conclude by discussing experimental signatures of spin-resolved band topology and avenues for future study. First, in SN 2G, we show that the spin bands of a spin-Weyl semimetal state computed for a spin direction $s={{{{{{{\bf{s}}}}}}}}\cdot \hat{{{{{{{{\bf{n}}}}}}}}}$ [Figs. 1(d) and 3] exhibit connectivity and topology related to that of the energy bands (in each spin sector) when a large Zeeman field **B** is applied parallel to *s* (${{{{{{{\bf{B}}}}}}}}\parallel \hat{{{{{{{{\bf{n}}}}}}}}}$). To explore the relationship between the energy and spin spectrum in a realistic spin-Weyl state, we performed ab-initio calculations on the layered transition-metal dichalcogenide *β*-MoTe_2_ [Fig. 6(a,c), see SN 9A for calculation details]. Previous theoretical works have predicted that *β*-MoTe_2_ realizes an ${{{{{{{\mathcal{I}}}}}}}}$- and ${{{{{{{\mathcal{T}}}}}}}}$-protected helical HOTI phase^41,46^, and previous experimental works have observed signatures of hinge-state-like 1D gapless channels in STM^47^ and in supercurrent oscillation^48^ probes of MoTe_2_. Through extensive spin-gap minimization calculations detailed in SN 9B, we find that for all choices of spin direction *s*, *β*-MoTe_2_ realizes a spin-Weyl semimetal state with an even number of spin-Weyl nodes in each half of the BZ. For the particularly simple case in which *s* is chosen to be:

13 $${s}_{xz}=\frac{1}{\sqrt{2}}\left({s}_{x}+{s}_{z}\right),$$

we specifically find that the spin spectrum is gapped along all high-symmetry lines [Fig. 6(b,d)], and that in the *k*_3_ > 0 half of the 3D BZ, there are three spin-Weyl points with positive charge and one spin-Weyl point with negative charge [Fig. 6(e,f)]. Because pairs of oppositely charged spin-Weyl points lie close together, and because the total spin-Weyl partial chiral charge in each *k*_3_-indexed half of the BZ is ∣2∣, then we conclude that overall, *β*-MoTe_2_ lies in the DSTI regime of a helical HOTI state (see SN 3E, 4D, and 9B).

**Fig. 7:**
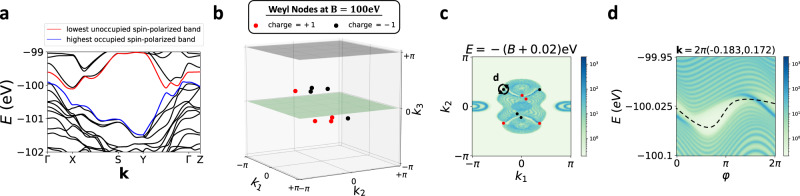
Converting spin-Weyl fermions to Weyl fermions in *β*-MoTe_2_ with an applied Zeeman field. **a** The electronic band structure of the Wannier-based tight-binding model of *β*-MoTe_2_ [Fig. 6(c)] in the presence of an ($\hat{{{{{{{{\bf{x}}}}}}}}}+\hat{{{{{{{{\bf{z}}}}}}}}}$)-directed *B* = ∣**B**∣ = 100eV (spin-) Zeeman field. We note that the band structure in **a** within each *s*_*xz*_ spin sector (here close to *E* ~ −*B* ~ −100eV) exhibits nearly the same band ordering and features as the field-free band ordering at *E*_*F*_ in Fig. 6(c). Though the blue and red singly-degenerate [nearly spin-polarized] bands in **a** appear gapped along high-symmetry BZ lines [Fig. 6(b)], the blue and red bands in fact cross in the BZ interior to form Weyl fermions. **b** The eight Weyl points connecting the blue and red bands in **a**. Remarkably, the locations and chiral charges of the Zeeman-induced Weyl nodes in **b** are nearly identical to the locations and partial chiral charges of the spin-Weyl nodes in Fig. 6(e). **c** The (001)-surface spectral function of *β*-MoTe_2_ at *E* = −100eV with the projected locations and partial chiral charges of the Zeeman-induced bulk Weyl points from **b** labeled with red and black circles. **d** The (001)-surface spectral function at *E* = −100eV computed on a small, counterclockwise path encircling the (001)-surface projection of the bulk Weyl point circled in **c**. The bulk Weyl points in **b**, **c** give rise to topological Fermi-arc surface states crossing the bulk (indirect) gap, as shown in **d** and SN 9C.

When we theoretically apply a (very) large Zeeman field **B**∥*s*_*xz*_ (∣**B**∣ = 100eV) to our Wannier-based tight-binding model of *β*-MoTe_2_, we observe that the spin-Weyl nodes continuously evolve into bulk Weyl nodes at energies *E* ≈ ± ∣**B**∣, as shown in Fig. 7(a,b) for energies close to −∣**B**∣. The presence of Weyl nodes in the energy spectrum implies that the surface spectrum computed at the energy of the Weyl nodes should exhibit topological surface Fermi arcs. Focusing on the experimentally accessible (001)-surface of *β*-MoTe_2_ (see ref. ^46^ and SN 9C), we compute the surface Green’s function in the presence of a strong ($\hat{{{{{{{{\bf{x}}}}}}}}}+\hat{{{{{{{{\bf{z}}}}}}}}}$)-directed Zeeman field [Fig. 7(c)]. Consistent with our predictions, we observe topological surface Fermi arcs crossing the bulk (indirect) gap [Fig. 7d].

**Fig. 8:**
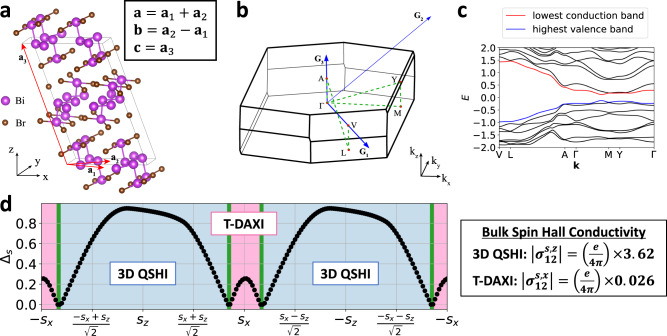
3D quantum spin Hall and ${{{{{{{\mathcal{T}}}}}}}}$-doubled axion insulator states in *α*-BiBr. **a** Crystal structure of the candidate helical HOTI *α*-BiBr^44,45^, which respects the symmetries of SSG $C2/m{1}^{{\prime} }$ (*#* 12.59, see SN 10A). The red arrows in **a** indicate the primitive lattice vectors **a**_1,2,3_, which are related to the conventional lattice vectors **a,** **b,** **c** through the equations in the inset box. **b** The bulk BZ of *α*-BiBr. The blue arrows in **b** indicate the primitive reciprocal lattice vectors **G**_1,2,3_. **c** Band structure of a DFT-obtained, symmetric, Wannier-based tight-binding model of *α*-BiBr (details provided in SN 10A), plotted along the dashed green high-symmetry **k**-path in **b**. In **c**, we label the highest valence [lowest conduction] doubly-degenerate bands in blue [red]. **d** The spin gap Δ_*s*_ and spin-resolved topology of *α*-BiBr plotted as a function of *s* rotated in the *xz*-plane (see SN 10B). For nearly every spin resolution direction in **d**, *α*-BiBr is spin-gapped, with the largest spin gap [Δ_*s*_ ≈ 0.95, ≈ 47% of its maximal value Δ_*s*_ = 2] surprisingly lying within 3 degrees of the **a**_3_∥**c** axis in **a** [see SN 10B]. The large **c**-axis-directed spin gap indicates that the bulk spin-orbital texture in *α*-BiBr is dominated by contributions almost entirely polarized along the **c** axis. Through (nested) spin-resolved Wilson loop calculations [see Fig. 4d and SN 10B], we obtain the spin-resolved topological phase diagram of *α*-BiBr shown in **d**, in which the ± *s*_*z*_-centered blue regions host 3D QSHI states, and the ± *s*_*x*_-centered pink regions host the spin-stable T-DAXI state introduced in this work. The inset box in **d** shows the non-quantized bulk spin Hall conductivity per unit cell of *α*-BiBr for the *s*_*z*_ and *s*_*x*_ spin directions (see SN 7 and 10C for calculation details). For both the 3D QSHI (*s*_*z*_) and T-DAXI (*s*_*x*_) regimes of *α*-BiBr, the bulk intrinsic spin Hall conductivity of *α*-BiBr lies close to the quantized topological contribution from its nontrivial spin-resolved bulk topology.

To observe and characterize spin-gapped phases under real-material conditions, we next perform a detailed analysis of the (spin-resolved) topology and spin-electromagnetic response of the quasi-1D candidate HOTI *α*-BiBr [Fig. 8(a,b,c), see SN 10 calculation details]^44,45^, for which angle-resolved photoemission spectroscopy (ARPES) and STM experiments have revealed signatures of 1D helical hinge states that persist to room temperature^50,51^. Prior to computing the spin-resolved topology of *α*-BiBr, we first probe its (hybrid) Wannier spectrum by computing the ${{{{{{{\mathcal{I}}}}}}}}$- and ${{{{{{{\mathcal{T}}}}}}}}$-symmetric nested Wilson loop of the occupied bands (SN 10B). Our nested Wilson loop calculations on *α*-BiBr reveal the characteristic higher-order (nested Wilson) spectral flow of a helical HOTI. This finding itself represents a significant result, as nested Wilson loop calculations on ab-initio-derived electronic structures remain exceedingly rare, with a noteworthy previous example being the identification of a non-symmetry-indicated helical HOTI state in noncentrosymmetric (${{{{{{{\mathcal{I}}}}}}}}$-broken) *γ*-MoTe_2_ via a pattern of helical nested Wilson loop flow similar to that in *α*-BiBr (but protected by distinct symmetries, as *α*-BiBr is centrosymmetric)^46^.

We next compute the spin gap for *α*-BiBr over the complete range of spin resolution directions *s*. Unlike previously for *β*-MoTe_2_ (SN 9B), we find that *α*-BiBr is spin-gapped for nearly all spin resolution directions (SN 10B). In particular, when restricting *s* to lie in the *xz*-plane [perpendicular to the *y*-directed chains in its crystal structure, see Fig. 8(a)], we observe that the spin gap in *α*-BiBr only closes in four extremely narrow spin-gapless (spin-Weyl) regions, which are indicated in green in Fig. 8(d). We observe that the *s*_*z*_ spin gap in *α*-BiBr is large (${\Delta }_{{s}_{z}}\approx 0.93$, ≈ 46% of the maximal value Δ_*s*_ = 2), and is much larger than the *s*_*x*_ spin gap (${\Delta }_{{s}_{x}}\approx 0.26$). This is consistent with earlier first-principles investigations of *α*-BiBr, which found the spin-electromagnetic (Rashba-Edelstein) response of its (010)-surface states to be strongly polarized in the *z*-direction relative to the *x*-direction^44^. We further find that overall, the global spin gap in *α*-BiBr peaks at a similarly large value (Δ_*s*_ ≈ 0.95) and lies within ≈ 3 degrees of the **a**_3_∥**c** lattice vector [Fig. 8(a)], indicating that the bulk spin-orbital texture in *α*-BiBr is dominated by contributions that are almost entirely polarized along the *c*-axis. As discussed earlier, similar SOC textures that are polarized along a high-symmetry (out-of-plane) crystallographic axis in 2D materials have been termed Ising SOC^66^. The appearance of a large bulk spin gap nearly locked to a crystallographic axis in *α*-BiBr [Fig. 8(d)] suggests that it would be intriguing to investigate the microscopic mechanism of the SOC in *α*-BiBr in future theoretical studies, and to study the spin-resolved response of *α*-BiBr in future photoemission and transport experiments, which may exhibit an unusually high degree of spin polarization relative to other strongly spin-orbit-coupled 3D materials.

Through (nested) spin-resolved Wilson loop calculations detailed in SN 10B, we find that the four spin-gapped regions in the spin-resolved topological phase diagram of *α*-BiBr [Fig. 8(d)] respectively correspond to two wide ${\nu }_{z}^{\pm }=\mp 2$ 3D QSHI regions [see Fig. 1(c)] with large spin gaps centered around *s* = ± *s*_*z*_, and two narrower ${\nu }_{x,y,z}^{\pm }=0$, *θ*^±^ = *π* T-DAXI regions with relatively smaller spin gaps centered around *s* = ± *s*_*x*_. For completeness, we note that because spins lying in the *xz*-plane are left invariant under the **E** ⋅ **B**-odd ${C}_{2y}\times {{{{{{{\mathcal{T}}}}}}}}$ antiunitary rotation symmetry of *α*-BiBr (see SN 10A), then the nontrivial partial axion angles *θ*^±^ = *π* in the T-DAXI regime of *α*-BiBr in Fig. 8(d) could alternatively be interpreted as quantized by the “rotation-anomaly” symmetry ${C}_{2y}\times {{{{{{{\mathcal{T}}}}}}}}$, rather than ${{{{{{{\mathcal{I}}}}}}}}$^21,23^.

To demonstrate physical signatures of nontrivial spin-resolved topology in *α*-BiBr, we next compute the intrinsic bulk spin Hall conductivity (per unit cell) in the 3D QSHI (*s* = *s*_*z*_) and T-DAXI (*s* = *s*_*x*_) regimes. Formally, our calculations were performed by applying a numerical implementation of the spin-nonconserving spin Hall conductivity derived from linear response through the Kubo formula (SN 7) to a DFT-obtained, Wannier-based model of *α*-BiBr (SN 10C). Even though both the *s*_*z*_ and *s*_*x*_ spin gaps in *α*-BiBr lie at less than half the maximal value of Δ_*s*_ = 2 [Fig. 8(d)], we find that both the 3D QSHI and T-DAXI regimes of *α*-BiBr exhibit bulk (nonquantized) spin Hall conductivities that lie close to the quantized topological contribution given by ${\sigma }_{12}^{s}=[e/(4\pi )]\times 2{\nu }_{z}^{+}$. Specifically, in the $| {\nu }_{z}^{+}|=2$ 3D QSHI region (*s* = *s*_*z*_), the spin Hall conductivity is nearly quantized $| {\sigma }_{12}^{{s}_{z}}|=(e/4\pi )\times 3.62$, whereas in the T-DAXI region (*s* = *s*_*x*_), the spin Hall conductivity is nearly vanishing $| {\sigma }_{12}^{{s}_{x}}|=(e/4\pi )\times 0.026$ [see Fig. 8(d) and SN 10C]. This result suggests a highly anisotropic spin Hall response in *α*-BiBr that interpolates between a large, extensive bulk contribution for *s*_*z*_ spin transport to a small, surface-dominated contribution for *s*_*x*_ spin transport. Given that *α*-BiBr is readily synthesizable^50,51^, the anisotropic spin-electromagnetic response predicted in this work should be accessible through straightforward (inverse) spin Hall measurements that are achievable within a short timeframe.

In addition to the spin Hall response of QSHI states [Fig. 2(c)] and Zeeman-induced surface Fermi arcs in spin-Weyl states (Fig. 6), spin-resolved topology may be experimentally accessed through terahertz measurements of the spin-magnetoelectric response of helical HOTIs in the T-DAXI regime, such as *α*-BiBr for *s* ≈ *s*_*x*_-polarized spins [see Fig. 5(b,c), Fig. 8(d), and SN 10B]. A spin imbalance at the surface of a T-DAXI will yield a non-quantized charge (electromagnetic) Hall response due to a lack of compensation between states with opposite partial Chern numbers. As depicted in Fig. 1(e), when *s*-nonconserving SOC is weak, we can describe the surface of a helical HOTI in the T-DAXI regime in terms of a massive fourfold Dirac cone (two bands per spin) with weak spin mixing^17,46^. In this scenario, the spin-up surface bands hence carry an anomalous Chern number 1/2 + *n*, and the spin-down surface bands correspondingly carry an anomalous Chern number − 1/2 − *n*, where $n\in {\mathbb{Z}}$. Selectively depopulating one spin species will hence yield a surface Fermi surface with a nonvanishing anomalous Hall conductivity^30^. By analogy with previous optical experiments performed on 3D TIs with magnetically gapped surface states^31,32^, a surface anomalous Hall conductivity can be measured through the Kerr and Faraday rotation of a terahertz probe.

A spin imbalance on the gapped surface of a T-DAXI can either be realized through selective excitation across the surface band gap using circularly polarized light (similar to optical experiments performed on monolayer transition-metal dichalcogenides and associated heterostructures^81^), or by spin injection using an adjoining magnetic transducer layer^82^. We expect the measured Faraday and Kerr rotation to vary linearly with the induced spin imbalance. Given that both the surface and the bulk are gapped, a spin-magnetoelectric response controlled by a spin imbalance could enable electric-field-tunable terahertz and infrared polarization modulators with nearly perfect transmission.

Furthermore, we note that the spin spectrum itself could be directly probed through generalizations of spin-ARPES (S-ARPES). Typical S-ARPES resolves the spin polarizations of individual bands, which are related to diagonal matrix elements of the *PsP* operator. S-ARPES experiments have previously been performed on the candidate spin-Weyl semimetal *β*-MoTe_2_ identified in this work^83^, and should be revisited in the context of spin-resolved topology. To fully resolve the spin spectrum experimentally, the off-diagonal matrix elements of *PsP* between occupied states must also be measured. However, this would require measuring the spin-dependent transition probability between pairs of occupied states. While such a measurement is currently beyond existing photoemission methods, recent proposals on double and pair photoemission spectroscopy^84,85^ may provide a promising and exciting path forward, provided that they can be developed with Mott- or very low-energy electron-diffraction-based spin detection.

Further material candidates in the T-DAXI, spin-Weyl semimetal, or 3D QSHI regimes may be identifiable among centrosymmetric, exfoliable materials with narrow band gaps and the SIs of a helical HOTI [see SN 4D and the text surrounding Eq. (11)], such as ZrTe_2_^43^. Dimensional reduction through exfoliation, as well as substitutional doping (i.e. Br/I, Zr/Hf, and Se/Te) to tune SOC can be explored to open a gap at the Fermi level in metallic material candidates. Surface passivation can also be explored to drive insulating behavior in metallic candidate materials, similar to the case of Al on Bi_2_Te_3_^86^. Though we have largely focused on solid-state realizations of spin-resolved topology, cold atoms have recently been employed to mimic 2D QSHI phases^87^, and hence may also serve as promising platforms for engineering the 3D T-DAXI states identified in this work.

Finally, our findings suggest several intriguing future directions. We have introduced a predictive framework for linking novel low-energy response theories to gauge-invariant quantities obtained from real-material calculations. Despite our progress unraveling the bulk and surface theories of ${{{{{{{\mathcal{I}}}}}}}}$- and ${{{{{{{\mathcal{T}}}}}}}}$-symmetric helical HOTIs, and despite other promising early efforts^88,89^, there remain numerous other noninteracting TCI phases—such as SU(2)-doubled magnetic AXIs^24,46^ and fourfold-rotation-anomaly TCIs like SnTe^19,21^—for which the bulk response theories are largely unknown. Additionally, while we focused on resolving band topology through the spin degree of freedom, the methods introduced in this work can straightforwardly be extended to sublattice (pseudospin), orbital, and layer degrees of freedom to predict new valleytronic and layertronic effects, such as valley- and spin-resolved generalizations of the layer Hall response recently observed in the antiferromagnetic AXI MnBi_2_Te_4_^90^. The (inverse) spin Hall effect is also measurable in magnetic systems^67^, and is arguably richer in magnets because it can be coupled to switchable magnetic order^91^. Therefore, it stands as an exciting future direction to determine whether there exist 3D magnetic materials that exhibit the axionic (inverse) spin-magnetoelectric responses introduced in this work, as well as to determine how the spin-magnetoelectric responses of such magnetic materials relate to recently introduced theories of magnetoelectric multipoles^92,93^. Furthermore, using the position-space formulations of the partial Chern numbers and partial axion angles (via layered partial Chern numbers), one can straightforwardly extend the spin-resolved topological quantities introduced in this work to the interacting setting using twisted spin boundary conditions^55,72^. Lastly, the spin-resolved generalizations of the axion angle and parity anomaly numerically identified in this work may also admit analytic descriptions in the languages of Berry connections and quantum field theory, which we hope to explore in future studies.

## Methods

We here summarize the properties of the projected spin operator and the construction of spin-resolved and nested spin-resolved Wilson loops. We further provide a brief summary of the computation of layer-resolved partial Chern numbers and summarize our implementation of the Kubo formula for computing the spin-Hall conductivity. Lastly, we review the methods used for our ab-initio calculations of the electronic and spin spectrum of *β*-MoTe_2_ and *α*-BiBr. Complete details of our research methodology can be found in the extensive Supplementary Notes.

### Summary of properties of the projected spin operator

Consider a 2*N* × 2*N* matrix Bloch Hamiltonian *H*(**k**). *H*(**k**) acts on a Hilbert space consisting of *N* spin-degenerate orbitals per unit cell (see SN 2A). Letting the Pauli matrices *σ*_*i*_ act on the spin degrees of freedom, we can define the spin operators

14 $${s}_{i}\equiv {\sigma }_{i}\otimes {{\mathbb{I}}}_{N},$$

where ${{\mathbb{I}}}_{N}$ is the *N* × *N* identity matrix acting in the orbital subspace of the entire Hilbert space (including both occupied and unoccupied states). Letting *P*(**k**) represent the projector onto a set of “occupied” energy eigenstates at **k**, we can then form the projected spin operator

15 $$PsP\equiv P({{{{{{{\bf{k}}}}}}}})\hat{{{{{{{{\bf{n}}}}}}}}}\cdot {{{{{{{\bf{s}}}}}}}}P({{{{{{{\bf{k}}}}}}}}),$$

for any choice of unit vector $\hat{{{{{{{{\bf{n}}}}}}}}}$ (see SN 2B). For notational convenience, we have frequently throughout this work suppressed the **k**-dependence of *PsP* when our discussion applies to both finite and infinite systems. When we are considering translationally-invariant systems, the projection operator *P* is taken to be a 2*N* × 2*N ***k**-dependent matrix where 2*N* is the number of spinful orbitals within each unit cell. The spin operator *s*_*i*_ is hence a **k**-independent 2*N* × 2*N* matrix. When we are considering finite systems with open boundary conditions, the projection operator *P* and the spin operator *s*_*i*_ are both taken to be 2*N* × 2*N ***k**-independent matrices, where 2*N* is the number of spinful orbitals in the entire finite system.

In SN 2 we prove that the spectrum of the projected spin operator *PsP* is gauge-invariant and changes continuously under perturbations of the Hamiltonian. This implies that the spectrum of *PsP* is a well-defined and perturbatively robust physical object in an insulator or for energetically isolated bands. For either the occupied bands of an insulator, or more generally a set of energetically isolated bands, we can write the projector *P*(**k**) as

16 $$P({{{{{{{\bf{k}}}}}}}})={P}_{+}({{{{{{{\bf{k}}}}}}}})+{P}_{-}({{{{{{{\bf{k}}}}}}}}),$$

where *P*_+_(**k**) is the projection operator onto a subset of *PsP* eigenstates with largest eigenvalue, and *P*_−_(**k**) is the projector onto the remaining *PsP* eigenstates. For the spin-compensated systems considered in this work, we have typically taken the rank of *P*_+_(**k**) to be equal to the rank of *P*_−_(**k**), such that the decomposition in Eq. (16) partitions the occupied states into two equal sets. We then define a spin gap to exist when, for every **k**, the smallest *PsP* eigenvalue for states in the image of *P*_+_(**k**) is distinct from the largest *PsP* eigenvalue for states in the image of *P*_−_(**k**).

### Summary of the spin-resolved and nested spin-resolved Wilson loop methods

For systems with a spin gap, we can use the projection operators *P*_+_(**k**) and *P*_−_(**k**) to define Wilson loops. Specifically, we can write the matrix of *P*_±_(**k**) in the tight-binding Hilbert space in a basis of eigenstates $|{u}_{n,{{{{{{{\bf{k}}}}}}}}}^{\pm }\rangle$ of *PsP* as

17 $$[{P}_{\pm }({{{{{{{\bf{k}}}}}}}})]=\mathop{\sum }\limits_{n=1}^{{N}_{{{{{{{{\rm{occ}}}}}}}}}^{\pm }}\big|{u}_{n,{{{{{{{\bf{k}}}}}}}}}^{\pm }\big\rangle \big\langle {u}_{n,{{{{{{{\bf{k}}}}}}}}}^{\pm }\big|,$$

where the square brackets indicate that [*P*_±_(**k**)] is a 2*N* × 2*N* matrix. The occupied-space matrix projector [*P*(**k**)] is then equal to [*P*_+_(**k**)] + [*P*_−_(**k**)] where [*P*_+_(**k**)][*P*_−_(**k**)] = 0. The corresponding holonomy matrix for [*P*_±_(**k**)] starting at a base point **k** and continuing along a straight-line path to **k + G** (where **G** is a primitive reciprocal lattice vector)—which we term the *P*_±_-Wilson loop matrix (or the spin-resolved Wilson loop matrix, see SN 3B)—is then given by the path-ordered product

18 $${[{{{{{{{{\mathcal{W}}}}}}}}}_{1,{{{{{{{\bf{k}}}}}}}},{{{{{{{\bf{G}}}}}}}}}^{\pm }]}_{m,n}=\left\langle {u}_{m,{{{{{{{\bf{k}}}}}}}}+{{{{{{{\bf{G}}}}}}}}}^{\pm }\right|\left(\mathop{\prod }\limits_{{{{{{{{\bf{q}}}}}}}}}^{{{{{{{{\bf{k}}}}}}}}+{{{{{{{\bf{G}}}}}}}}\leftarrow {{{{{{{\bf{k}}}}}}}}}[{P}_{\pm }({{{{{{{\bf{q}}}}}}}})]\right)\left|{u}_{n,{{{{{{{\bf{k}}}}}}}}}^{\pm }\right\rangle .$$

In SN 3 we show that $[{{{{{{{{\mathcal{W}}}}}}}}}_{1,{{{{{{{\bf{k}}}}}}}},{{{{{{{\bf{G}}}}}}}}}^{\pm }]$ is a unitary matrix with eigenvalues ${e}^{i{({\gamma }_{1}^{\pm })}_{j,{{{{{{{\bf{k,G}}}}}}}}}}$. From this, we define the partial Chern numbers *C*^±^ to respectively be equal to the winding numbers of ${\sum }_{j}{({\gamma }_{1}^{\pm })}_{j,{{{{{{{\bf{k,G}}}}}}}}}$ as functions of momenta perpendicular to **G** (see SN 3C for further details).

Going further, we can write the eigenvectors of $[{{{{{{{{\mathcal{W}}}}}}}}}_{1,{{{{{{{\bf{k}}}}}}}},{{{{{{{\bf{G}}}}}}}}}^{\pm }]$ as ${[{\nu }_{j,{{{{{{{\bf{k}}}}}}}},{{{{{{{\bf{G}}}}}}}}}^{\pm }]}_{m}$, which satisfy

19 $$[{{{{{{{{\mathcal{W}}}}}}}}}_{1,{{{{{{{\bf{k}}}}}}}},{{{{{{{\bf{G}}}}}}}}}^{\pm }][{\nu }_{j,{{{{{{{\bf{k}}}}}}}},{{{{{{{\bf{G}}}}}}}}}^{\pm }]={e}^{i{({\gamma }_{1}^{\pm })}_{j,{{{{{{{\bf{k,G}}}}}}}}}}[{\nu }_{j,{{{{{{{\bf{k}}}}}}}},{{{{{{{\bf{G}}}}}}}}}^{\pm }].$$

In the Bloch basis, we can then express the *P*_±_-Wannier band eigenstates as

20 $$\left|{w}_{j,{{{{{{{\bf{k}}}}}}}},{{{{{{{\bf{G}}}}}}}}}^{\pm }\right\rangle=\mathop{\sum }\limits_{m=1}^{{N}_{{{{{{{{\rm{occ}}}}}}}}}^{\pm }}{[{\nu }_{j,{{{{{{{\bf{k}}}}}}}},{{{{{{{\bf{G}}}}}}}}}^{\pm }]}_{m}\left|{u}_{m,{{{{{{{\bf{k}}}}}}}}}^{\pm }\right\rangle .$$

If there is a gap between the eigenvalues ${e}^{i{({\gamma }_{1}^{\pm })}_{j,{{{{{{{\bf{k,G}}}}}}}}}}$, we can choose a subset $j=1,\ldots {N}_{W}^{\pm }$ of Wilson loop eigenstates on which to form the *P*_±_-Wannier band projector

21 $$[{\widetilde{P}}_{{{{{{{{\bf{G}}}}}}}}}^{\pm }({{{{{{{\bf{k}}}}}}}})]=\mathop{\sum }\limits_{j=1}^{{N}_{W}^{\pm }}\left|{w}_{j,{{{{{{{\bf{k}}}}}}}},{{{{{{{\bf{G}}}}}}}}}^{\pm }\right\rangle \left\langle {w}_{j,{{{{{{{\bf{k}}}}}}}},{{{{{{{\bf{G}}}}}}}}}^{\pm }\right|.$$

Lastly from Eq. (21), we then define the nested spin-resolved Wilson loops as the holonomy matrices that correspond to the *P*_±_-Wannier band projectors $[{\widetilde{P}}_{{{{{{{{\bf{G}}}}}}}}}^{\pm }({{{{{{{\bf{k}}}}}}}})]$. Concretely, the nested spin-resolved Wilson loop matrix $[{{{{{{{{\mathcal{W}}}}}}}}}_{2,{{{{{{{\bf{k}}}}}}}},{{{{{{{\bf{G}}}}}}}},{{{{{{{{\bf{G}}}}}}}}}^{{\prime} }}^{\pm }]$ (see SN 4B) is given by

22 $${[{{{{{{{{\mathcal{W}}}}}}}}}_{2,{{{{{{{\bf{k}}}}}}}},{{{{{{{\bf{G}}}}}}}},{{{{{{{{\bf{G}}}}}}}}}^{{\prime} }}^{\pm }]}_{i,\, j}=\left\langle {w}_{i,{{{{{{{\bf{k}}}}}}}}+{{{{{{{{\bf{G}}}}}}}}}^{{\prime} },{{{{{{{\bf{G}}}}}}}}}^{\pm }\right|\left(\mathop{\prod }\limits_{{{{{{{{\bf{q}}}}}}}}}^{{{{{{{{\bf{k}}}}}}}}+{{{{{{{{\bf{G}}}}}}}}}^{{\prime} }\leftarrow {{{{{{{\bf{k}}}}}}}}}[{\widetilde{P}}_{{{{{{{{\bf{G}}}}}}}}}^{\pm }({{{{{{{\bf{q}}}}}}}})]\right)\left|{w}_{j,{{{{{{{\bf{k}}}}}}}},{{{{{{{\bf{G}}}}}}}}}^{\pm }\right\rangle .$$

In SN 4C, we further show that the spectrum of the nested spin-resolved Wilson loop defines the nested partial Chern numbers, by analogy to the partial Chern numbers defined above in the text following Eq. (18).

### Summary of the layer-resolved partial Chern number calculation method

We begin by considering a 3D system with the primitive Bravais lattice vectors {**a**_*j*_, **a**_*l*_, **a**_*i*_}. We next cut our system into a slab geometry with *N*_*i*_ unit cells (slab layers) along the (now-finite) **a**_*i*_ direction, while keeping the system infinite along **a**_*j*_ and **a**_*l*_. We take there to be *N*_sta_ = 2*N*_orb_ tight-binding basis states per unit cell, where the factor of 2 accounts for the on-site (internal) spin-1/2 degree of freedom. In this basis, the spin operator oriented in a spin direction $\hat{{{{{{{{\bf{n}}}}}}}}}$ is defined as $s\equiv \hat{{{{{{{{\bf{n}}}}}}}}}\cdot {{{{{{{\boldsymbol{\sigma }}}}}}}}\otimes {{\mathbb{I}}}_{{N}_{{{{{{{{\rm{orb}}}}}}}}}}\otimes {{\mathbb{I}}}_{{N}_{i}}$, where the Pauli matrices ***σ*** act on the spin-1/2 degree of freedom, and where ${{\mathbb{I}}}_{{N}_{{{{{{{{\rm{orb}}}}}}}}}}$ and ${{\mathbb{I}}}_{{N}_{i}}$ are identity matrices that respectively act on the orbital and unit cell (layer) degrees of freedom. We next denote the projection operator onto the occupied energy bands of the finite slab at the 2D crystal momentum **k** = (*k*_*j*_, *k*_*l*_) as *P*(**k**). The projector *P*(**k**) can then be decomposed using the projected spin operator *PsP* via Eq. (16).

Using each projector *P*_±_(**k**), we next obtain the partial Chern number of the finite 2D slab through (see SN 3C)

23 $${C}_{jl}^{\pm }=\frac{-i}{2\pi }\int\,d{{{{{{{\bf{k}}}}}}}}\,{{{{{{{\rm{Tr}}}}}}}}\left({P}_{\pm }({{{{{{{\bf{k}}}}}}}})\left[\frac{\partial {P}_{\pm }({{{{{{{\bf{k}}}}}}}})}{\partial {k}_{j}},\frac{\partial {P}_{\pm }({{{{{{{\bf{k}}}}}}}})}{\partial {k}_{l}}\right]\right),$$

where the integral in **k** is performed over the 2D BZ of the slab, and where the matrix trace (${{{{{{{\rm{Tr}}}}}}}}$) is performed over both the *N*_*i*_ unit cells (layers) and the 2*N*_orb_ tight-binding basis states per unit cell. Using Eq. (23), we may further define a layer-resolved partial Chern number ${C}_{jl}^{\pm }({n}_{i})$ by expanding the matrix trace in the tight-binding basis and then re-summing (tracing) only over the degrees of freedom within each layer (see SN 5D for further details). The layer-resolved partial Chern number ${C}_{jl}^{\pm }({n}_{i})$ specifically quantifies how the partial Chern number of a 2D slab is distributed over the *N*_*i*_ unit cells (layers) in the finite slab, and can be viewed as the spin-resolved generalization of the well-established position-space (layer-resolved) Chern number^29,30,76^.

### Spin-Hall conductivity

As detailed in SN 7, the spin conductivity tensor ${\sigma }_{\mu \nu }^{s,i}$ parametrizes the linear response of the spin current **J**^*s*,*i*^ to an applied DC electric field **E** via

24 $$\langle {\,J}_{\mu }^{s,i}\rangle=\mathop{\sum}\limits_{\nu }{\sigma }_{\mu \nu }^{s,i}{E}_{\nu }.$$

Here *μ* and *ν* index spatial coordinates, and *i* = *x*, *y*, *z* indexes the spin direction. The spin conductivity can then be evaluated using the standard Kubo formula

25 $${\sigma }_{\mu \nu }^{s,i}=\mathop{\lim }\limits_{\epsilon \to 0}\int\nolimits_{0}^{\infty }dt\left\langle \left[{J}_{\mu }^{s,i}(t),\, {X}_{\nu }(0)\right]\right\rangle {e}^{-\epsilon t},$$

where *X*_*ν*_ is the *ν* component of the position operator (which couples to the external electric field in the Hamiltonian), the time-dependence of operators is evaluated in the Heisenberg picture using the unperturbed (**E** = **0**) Hamiltonian *H*_0_, and the average is computed with respect to the unperturbed ground state. We next define the spin current operator to be

26 $${J}_{\mu }^{s,i}=\frac{\partial }{\partial t}\left({X}_{\mu }{s}^{i}\right)=i\left[{H}_{0},\, {X}_{\mu }{s}^{i}\right].$$

From Eqs. (25) and (26), we then define the spin Hall conductivity to be the antisymmetric part of the spin conductivity tensor. To numerically evaluate Eq. (25) for a tight-binding model, we work in a hybrid Wannier basis following the approach of ref. ^94^. For a semi-infinite 3D system consisting of a finite number of 2D layers, this also allows us to define a layer-resolved spin Hall conductivity as the integrand of Eq. (25) before taking the sum over layers (see SN 7C).

### Ab-initio calculation details

We here detail our first-principles (DFT) calculations for *β*-MoTe_2_ and *α*-BiBr. First, as detailed in SN 9, our first-principles calculations for *β*-MoTe_2_ were performed within the DFT framework using the projector-augmented wave (PAW) method^95,96^ as implemented in the Vienna ab-initio simulation package (VASP)^97,98^. In our DFT calculations for *β*-MoTe_2_, we adopted the Perdew-Burke-Ernzerhof (PBE) generalized gradient approximation exchange-correlation functional^99^, and SOC was incorporated self-consistently. The cutoff energy for the plane-wave expansion was 400 eV, and 0.03 × 2*π* Å^−1 ^**k**-point sampling grids were used in the self-consistent process.

To analyze the spin-resolved band topology, we constructed a symmetric, Wannier-based tight-binding model fit to the electronic structure of *β*-MoTe_2_ obtained from our DFT calculations. We constructed symmetric Wannier functions for the bands near the Fermi energy *E*_*F*_ in *β*-MoTe_2_ by using the Wannier90 package^100^ for the Mo 4*d* and the Te 5*p* orbitals, and then performing a subsequent SG symmetrization using WannierTools^101^. We denote the Hamiltonian of the Wannier-based tight-binding model as $[{H}_{{{{{{{{{\rm{MoTe}}}}}}}}}_{2}}]$. The single-particle Hilbert space of $[{H}_{{{{{{{{{\rm{MoTe}}}}}}}}}_{2}}]$ consists of 44 spinful Wannier functions per unit cell; the Bloch Hamiltonian $[{H}_{{{{{{{{{\rm{MoTe}}}}}}}}}_{2}}({{{{{{{\bf{k}}}}}}}})]$ is therefore an 88 × 88 matrix. To reduce the computational resources required for our spin-resolved and Wilson loop calculations, we next truncated $[{H}_{{{{{{{{{\rm{MoTe}}}}}}}}}_{{{{{{{{\rm{2}}}}}}}}}}]$ to only contain hopping terms with an absolute magnitude greater than or equal to 0.001eV. We have confirmed that this truncation affects neither the band ordering nor the qualitative features of the band structure near the Fermi energy in *β*-MoTe_2_ (see SN 9A for complete calculation details).

Next, as detailed in SN 10, our first-principles calculations for *α*-BiBr were also performed within the DFT framework using the PAW method^95,96^ as implemented in VASP^97,98^. In our DFT calculations for *α*-BiBr, we similarly adopted the PBE generalized gradient approximation exchange-correlations functional^99^, and SOC was also incorporated self-consistently. The cutoff energy for the plane-wave expansion was 400eV, and 0.03 × 2*π* Å^−1 ^**k**-point sampling grids were used in the self-consistent process.

To analyze the spin-resolved band topology of *α*-BiBr, we next constructed a symmetric, Wannier-based tight-binding model fit to the electronic structure of *α*-BiBr obtained from our DFT calculations. We specifically constructed symmetric Wannier functions for the bands near *E*_*F*_ in *α*-BiBr by using the Wannier90 package^100^ for the Bi 6*p* and the Br 4*p* orbitals, and then performing a subsequent SG symmetrization using WannierTools^101^. We denote the tight-binding Hamiltonian of the Wannier-based tight-binding model as *H*_BiBr_. The single-particle Hilbert space of *H*_BiBr_ consists of 48 spinful Wannier functions per primitive (unit) cell; the Bloch Hamiltonian [*H*_BiBr_(**k**)] is therefore a 96 × 96 matrix, To reduce the computational resources required for our spin-resolved and Wilson loop tight-binding calculations, we then truncated [*H*_BiBr_(**k**)] to only contain hopping terms with an absolute magnitude greater than or equal to 0.001eV. We have confirmed that the truncated Wannier-based tight-binding model exhibits the same band ordering and qualitative features as the first-principles electronic structure of *α*-BiBr (see SN 10A for complete calculation details).

### Supplementary information

Supplementary Information

## Data Availability

The data supporting the theoretical findings of this study are available within the paper and as code examples in the NESTED_AND_SPIN_RESOLVED_WILSON_LOOP^58^ repository. All first-principles calculations were performed using CIF structure files with the experimental lattice parameters, which can be obtained from the Inorganic Crystal Structure Database (ICSD)^107^ using the accession numbers provided in SN 8.

## Code availability

The spin-resolved tight-binding and (nested) Wilson loop calculations in this work were performed using the freely available Python package NESTED_AND_SPIN_RESOLVED_WILSON_LOOP^58^, which represents an extension of the PythTB open-source Python tight-binding package^108^ that was implemented and utilized for the preparation of refs. ^23,59^, and was then greatly expanded for the present work.
