# Supplementary material for: Spin-resolved topology and partial axion angles in three-dimensional insulators
Source: Nat Commun. 2024 Jan 16;15:550. doi: 10.1038/s41467-024-44762-w (PMC10791639; doi:10.1038/s41467-024-44762-w)
Supplement: Supplementary file 1 — Supplementary Information [file 41467_2024_44762_MOESM1_ESM.pdf]

Supplementary Information for "Spin-Resolved Topology and Partial Axion Angles in Three-Dimensional Insulators"

Kuan-Sen Lin,[1,2,*] Giandomenico Palumbo,[3] Zhaopeng Guo,[4,5] Yoonseok Hwang,[1]
Jeremy Blackburn,[6] Daniel P. Shoemaker,[7,8] Fahad Mahmood,[8,9] Zhijun Wang,[4,5]
Gregory A. Fiete,[10,11,†] Benjamin J. Wieder,[10,11,12,‡] and Barry Bradlyn[1,§]

[1]*Department of Physics and Institute for Condensed Matter Theory, University of Illinois at Urbana-Champaign, Urbana, IL 61801, USA*
[2]*Kavli Institute for Theoretical Physics, University of California, Santa Barbara, CA 93106, USA*
[3]*School of Theoretical Physics, Dublin Institute for Advanced Studies, 10 Burlington Road, Dublin 4, Ireland*
[4]*Beijing National Laboratory for Condensed Matter Physics and Institute of Physics, Chinese Academy of Sciences, Beijing 100190, China*
[5]*University of Chinese Academy of Sciences, Beijing 100049, China*
[6]*Department of Computer Science, State University of New York at Binghamton, Binghamton, NY 13902, USA*
[7]*Department of Materials Science and Engineering, University of Illinois at Urbana-Champaign, Urbana, IL 61801, USA*
[8]*Materials Research Laboratory, University of Illinois at Urbana-Champaign, Urbana, IL 61801, USA*
[9]*Department of Physics, University of Illinois at Urbana-Champaign, Urbana, IL 61801, USA*
[10]*Department of Physics, Northeastern University, Boston, MA 02115, USA*
[11]*Department of Physics, Massachusetts Institute of Technology, Cambridge, MA 02139, USA*
[12]*Institut de Physique Théorique, Université Paris-Saclay, CEA, CNRS, F-91191 Gif-sur-Yvette, France*
(Dated: December 15, 2023)

* Corresponding author: kuansen2@illinois.edu
† Corresponding author: g.fiete@northeastern.edu
‡ Primary address for B. J. W.: Institut de Physique Théorique, Université Paris-Saclay, CEA, CNRS, F-91191 Gif-sur-Yvette, France. Corresponding author: benjamin.wieder@ipht.fr
§ Corresponding author: bbradlyn@illinois.edu

CONTENTS

Supplementary Notes 4

1. Introduction to the Supplementary Notes 4

2. Projected Spin Operators, Spin Spectra, and Spin Gaps 7
   A. Tight-Binding Notation 8
   B. Properties of the Projected Spin Operator 9
   C. Effects of Perturbations on the Spin Gap 14
   D. A Physical Interpretation of the Spin Gap 17
   E. Explicit Calculations for a 3D Topological Insulator Model 18
   F. Effects of Spin-Orbital Entanglement on the Spin Spectrum 20
   G. (Spin) Band Structure in a Strong Zeeman Field 21

3. Spin-Resolved Wilson Loops 24
   A. P-Wilson Loop 24
   B. $P_{\pm}$-Wilson Loop 25
   C. Spectral Flow of the $P_{\pm}$-Wilson Loop 27
   D. 2D Spinful Time-Reversal-Invariant Systems 30
   E. 3D Spinful Time-Reversal-Invariant Systems with Inversion Symmetry 33
   F. 3D TIs With and Without Inversion Symmetry 36
   G. Spin-Stable Topology in a 2D Fragile Topological Insulator 37
   H. Spin-Resolved Wilson Loops and the Spin Entanglement Spectrum 45

4. Nested Spin-Resolved Wilson Loops 47
   A. Nested P-Wilson Loop 48
   B. Nested $P_{\pm}$-Wilson Loop 51
   C. General Properties of the Nested $P_{\pm}$-Wilson Loop Spectra 54
      1. Nested P- and $P_{\pm}$-Wilson Loops When $\mathbf{G} = \mathbf{G}'$ 55
      2. The Nested P- and $P_{\pm}$-Wilson Loops Wind Along at Most One Primitive Reciprocal Lattice Direction 56
      3. Relation Between Nested P- and $P_{\pm}$-Wilson Loops and (Partial) Weak Chern Numbers 57
      4. $\mathbb{Z}_2$-Stable Spectral Flow in the Nested $P_{\pm}$-Wilson Loop Eigenphases in the Presence of Bulk $\mathcal{I}$ and Spinful $\mathcal{T}$ Symmetries, and the Corresponding Spin-Stable Bulk Topology 61
   D. Comparison Between Spin-Stable Topology and Symmetry-Indicated Topology in $\mathcal{I}$- and Spinful $\mathcal{T}$-Symmetric 3D Insulators 64
      1. Spin-Resolved Layer Constructions 65
      2. The Quantum Spin Hall Insulator and the $\mathcal{T}$-Doubled Axion Insulator 70
      3. Responses of 3D QSHIs and T-DAXIs, and the Deduction of an Intermediate Spin-Weyl Regime 74
   E. Numerical Calculations for Nested $P_{\pm}$-Wilson Loops of an $\mathcal{I}$- and $\mathcal{T}$-Symmetric Helical HOTI 76

5. Position-Space Signatures of Spin-Resolved Topology 84
   A. Chern Marker 84
   B. The Partial Chern Marker and the Spin Chern Marker 85
   C. Layer-Resolved Chern Number 86
   D. Layer-Resolved Partial Chern Numbers and Layer-Resolved Spin Chern Number 87
   E. Layer-Resolved Spin Chern Number of a Helical HOTI 89

6. Symmetry Constraints on Wilson Loop Spectra 93
   A. Unitary $\mathcal{I}$ Constraint on the P-Wilson Loop 94
   B. Unitary $\mathcal{I}$ Constraint on the Nested P-Wilson Loop 96
   C. Antiunitary $\mathcal{T}$ Constraint on the P-Wilson Loop 99
   D. Antiunitary $\mathcal{T}$ Constraint on the Nested P-Wilson Loop 100
   E. Unitary $\mathcal{I}$ Constraint on the $P_{\pm}$-Wilson Loop 102
   F. Unitary $\mathcal{I}$ Constraint on the Nested $P_{\pm}$-Wilson Loop 103
   G. Antiunitary $\mathcal{T}$ Constraint on the $P_{\pm}$-Wilson Loop 104
   H. Antiunitary $\mathcal{T}$ Constraint on the Nested $P_{\pm}$-Wilson Loop 105
   I. A Summary of Symmetry Constraints on P-, $P_{\pm}$-, Nested P-, and Nested $P_{\pm}$-Wilson Loop 106
   J. Eigenvalues and Eigenvectors of Wilson Loop Operators and Their Hermitian Conjugates 107

K. Transformation of the $[V(\mathbf{G})]$ Matrix Under Unitary $\mathcal{I}$ 107
L. Transformation of the $[V(\mathbf{G})]$ Matrix Under Antiunitary $\mathcal{T}$ 109

7. Bulk Spin Hall Conductivity 110
A. Computational Details 110
B. Spin Hall Conductivity of the 2D Spin-Stable Quantum Spin Hall Insulator 111
C. Layer-Resolved Spin Hall Conductivity of T-DAXIs in 3D 111

8. Spin-Resolved Topology in Real Materials 114

9. First-Principles Analysis of β-$MoTe_2$ 115
A. Details of Density Functional Theory Calculations on β-$MoTe_2$ 115
B. Spin-Resolved Topology of β-$MoTe_2$ 118
1. Searching for a Spin Gap in β-$MoTe_2$ at Generic Spin Resolution Directions 121
C. Physical Observables of the Spin-Weyl State in β-$MoTe_2$: Surface Fermi Arcs in a Strong Zeeman Field 123

10. First-Principles Analysis of α-BiBr 124
A. Details of Density Functional Theory Calculations on α-BiBr 124
B. Spin-Resolved Topology of α-BiBr 126
C. Physical Signatures of Spin-Gapped States in α-BiBr: Bulk Spin Hall Conductivity 136

Supplementary References 137

SUPPLEMENTARY NOTES

1. INTRODUCTION TO THE SUPPLEMENTARY NOTES

In this section, we provide a guide to the Supplementary Notes, and summarize the main results of this work. We begin in Supplementary Note (SN) 2 with a discussion of the general properties of projected spin operators, building on the formalism introduced by Prodan in Supplementary Reference (SRef.) [1]. In SN 2 A, we first review the notation that we will use for states and operators in tight-binding models. The tight-binding models that we consider in this work include both physically motivated toy models as well as systematically constructed, Wannier-based tight-binding truncations that reproduce a set of bands in a real material. Next, in SN 2 B, we introduce the projected spin operator PsP. We show how the projected spin operator can be defined in terms of a projector onto a set of "occupied" (Bloch) states and a choice of spin direction $s = \mathbf{s}\cdot\hat{\mathbf{n}}$. For periodic systems, we introduce the spin band structure (spin spectrum), which we define through the gauge-invariant eigenvalues of the projected spin operator. We examine constraints imposed on the spin operator and the spin spectrum by time-reversal and crystal symmetries, and provide a precise definition of a *spin gap* in the spin spectrum. In SN 2 C, we show how to compute changes to the spin band structure order by order in perturbations to the Hamiltonian. This expansion establishes the spin band structure as a well-defined, perturbatively-robust physical object in an insulator given a choice of spin direction. In particular in SN 2 C, we show that perturbations to the Hamiltonian induce smooth and bounded changes to the spin band structure. Building from this result, we then show in SN 2 D that the smallest nontrivial eigenvalue of the projected spin operator—which determines the spin gap in the presence of bulk inversion and time-reversal symmetries—places a bound on the relaxation time for spin-flip excitations in an insulating material.

Exploiting the fact that the energy and spin gaps are robust to perturbations, we continue in SN 2 E by defining the concept of *spin-resolved band topology*, beginning with an explicit calculation of the spin band structure for a simple model of a three-dimensional (3D) inversion- and time-reversal-symmetric topological insulator (TI). We review the notion of spin-resolved partial Berry curvature as a 2D extension of the partial polarization introduced in SRefs. [1, 2]. This allows us to introduce in SN 2 E a formulation of partial Chern numbers, defined as the Chern numbers computed separately for the spin bands in each half of a gapped spin spectrum. We next argue that in a 3D insulator, there can exist linearly-dispersing, twofold degeneracies in the spin spectrum—which we term spin-Weyl nodes—that act as monopole sources of partial Chern number. Spin-Weyl nodes are hence analogous to (energy) Weyl nodes, which act as monopole sources of ordinary (total, *i.e.* charge) Chern number. We then prove that 3D TIs must generically have an odd number of spin-Weyl nodes in their spin band structures in each half of the Brillouin zone (BZ).

In SN 2 F, we next explore how entanglement between spin and orbital degrees of freedom can affect the spin band structure, focusing on the effect of the choice of spin direction s in PsP. Then, in SN 2 G, we derive a relationship between the spin band structure and the (energy) band structure in the presence of a strong Zeeman field. We rigorously show that the band structure for a spectrally flattened Hamiltonian in the presence of a strong Zeeman field is adiabatically deformable to the spin band structure. Going further, we argue that in many cases, we can extend the correspondence and link the spin band structure to the energy spectrum *without* spectral flattening, allowing us to relate the topology and connectivity of bands in the spin spectrum to the topology and connectivity of bands in the energy system for an insulator in the presence of a strong Zeeman field. Using this result, we argue in SN 2 G that a 3D TI (spin-Weyl semimetal) in a strong Zeeman field will generically have low-energy Weyl nodes in the *energy* spectrum, which can be adiabatically connected to spin-Weyl nodes in the spin band structure.

In SN 3 we continue our study of the topology of spin bands by introducing a general formalism for computing spin-resolved topological invariants. We begin in SN 3 A with a short review of the ordinary Wilson loop (non-Abelian Berry phase) for a set of occupied states [3–5]. Then, in SN 3 B we use the spin band structure to define a spin-resolved Wilson loop for systems with a spin gap, where the "occupied" states are taken to be the spin bands in one half of the spin spectrum of an insulator with a spin gap. Crucially, our formalism for spin-resolved topology *does not* require the system to carry a conserved spin direction (*i.e.* s_z symmetry); the notion of spin-resolved band topology introduced in this work remains valid when spin-conservation symmetry is broken by spin-orbit coupling (SOC), which typically cannot be neglected in real materials [6]. In SN 3 C we show how the partial Chern numbers first introduced in SN 2 E can be computed from the eigenphases of the spin-resolved Wilson loop. Making contact with prior work, we then show how the partial Chern numbers computed in this work can be used to compute the spin Chern numbers introduced in SRefs. [1, 2, 7]. By applying our definition of the partial Chern number to the Kubo formula for spin Hall conductivity, we further show that nontrivial partial Chern numbers indicate the presence of an intrinsic, topological contribution to the spin Hall conductivity, even in systems where spin conservation is (weakly) broken by SOC.

Having established a formal definition for the spin-resolved Wilson loop, we next consider several illustrative examples of band topology identifiable from spectral flow in the (first) spin-resolved Wilson loop. In SN 3 D, we begin by applying our spin-resolved Wilson loop formalism to the case of 2D time-reversal-invariant TIs. We specifically review

the results of SRef. [1], in which it was shown that the partial Chern numbers of the occupied bands of an insulator can only change by even integers when a spin gap closes, noting that spin gap closures are typically unaccompanied by energy gap closures. Using this result, we demonstrate that in the presence of time-reversal symmetry, the parity of the partial Chern number cannot change without closing an energy gap, and hence provides an alternative definition of the Kane-Mele $\mathbb{Z}_2$ index for 2D insulators with spinful time-reversal symmetry. In SN 3 E and 3 F we next compute the spin-resolved Wilson loops for models of a time-reversal-invariant 3D TI both with and without spatially inversion symmetry. We numerically confirm the presence of an odd number of spin-Weyl nodes in each half of the BZ, consistent with the theoretical result established in SN 2 E. We additionally numerically demonstrate that the spin-Weyl nodes act as sources and sinks of partial Berry curvature. In SN 3 G, we next examine the spin-resolved Wilson loops of the model of a 2D fourfold-rotation- and time-reversal-symmetric fragile topological insulator first introduced in SRef. [8]. We demonstrate that the topologically fragile model has a nonzero even partial Chern number, which we find to be stable to the addition of trivial bands to PsP (bands with vanishing partial Chern numbers), provided that the spin gap remains open as the trivial bands are coupled to the system.

This motivates introducing a refined notion of band topology for bands within the spin spectrum, which we term "spin-stable" topology. Systems with inequivalent spin-stable topology have spin band structures that cannot be adiabatically deformed into each other without breaking a symmetry or closing either an energy gap or a spin gap. As we show in SN 3 G, this definition is physically motivated, as systems with distinct spin-stable topology exhibit different spin-electromagnetic responses, *even if they share the same electronic band topology* without spin resolution. In SN 3 H, we then formulate a notion of a spin-resolved entanglement spectrum by restricting the projector onto the lower (or upper) spin bands to one half of a system in position space. We show that this particular formulation of the spin entanglement spectrum is homotopic to the spectrum of the spin-resolved Wilson loop taken in the direction perpendicular to the position-space bipartition. We demonstrate this concretely by computing the spin entanglement spectrum for a 2D TI (which features chiral modes in the entanglement spectrum), a 3D TI (which features Fermi arcs in the entanglement spectrum emanating from spin-Weyl nodes), and the spin-stable 2D fragile model from SRef. [8] (which features a pair of chiral modes signifying an even partial Chern number). Our results are also relevant to ongoing experimental efforts exploring the interplay between spin and topology in quantum materials [9–12].

In SN 4, we use the projected spin operator to study crystal-symmetry-protected, spin-stable topology in 3D insulators, which we accomplish by computing *nested* Wilson loops. We begin in SN 4 A by reviewing the nested Wilson loop formalism of SRefs. [13–15]. We review how the Wilson loop matrix from SN 3 A defines a set of Wannier bands and show that if there is a gap in the Wilson loop spectrum, we can compute a second (nested) Wilson loop by projecting onto a subset of the Wannier bands. We pay particular attention to the numerical subtleties involved in calculating the projector onto subsets of Wannier bands, and introduce a robust method for computing the nested Wilson loop in tight-binding models. In SN 4 B we define a nested spin-resolved Wilson loop by first identifying the eigenstates of the spin-resolved Wilson loop as spin-resolved Wannier bands (*i.e.* the Wannier bands of the Wilson loop computed using the spin bands of PsP). We then define the nested spin-resolved Wilson loop as the Wilson loop computed for a projector onto a subset of the spin-resolved Wannier bands.

We continue in SN 4 C by discussing general properties of nested (spin-resolved) Wilson loops and the relationship between the nested (spin-resolved) Wilson spectrum and (spin-stable) bulk topology. In SN 4 C 1 and 4 C 2, we establish that in systems with an energy gap and a (spin-resolved) Wannier gap, the nested (partial) Wilson loop eigenvalues can have nonzero winding numbers as functions of at most one crystal momentum. This result, which was implicit in SRefs. [15, 16], allows us to define a nested (partial) Chern number independent of the base point used to define the nested Wilson loop. Using this result, in SN 4 C 3 we show that the sum of the nested (partial) Chern numbers over all sets of (spin-resolved) Wannier bands gives the weak (partial) Chern number of the occupied bands. In SN 4 C 4 we specialize to systems with both inversion and time-reversal symmetry. We show that for insulators with these symmetries, the nested partial Chern number can only change by an even number when a gap in the spin-resolved Wannier spectrum closes and reopens (provided that the spin gap and energy gap remain open). We then relate this result to established topological phases, most notably the topological crystalline insulating states that have become known as higher-order TIs (HOTIs) [14–41].

We next consider the position-space implications of our nested (spin-resolved) Wilson loop calculations. In SN 4 D we formulate a precise relationship between the nested spin-resolved Wilson loop and a novel notion of *spin-resolved layer constructions* of 3D spin-stable topological phases, generalizing the approach of SRefs. [33, 42–44]. We specifically show that the layer constructions for spin-stable topological phases with inversion and time-reversal symmetry can be obtained from the layer constructions for magnetic topological insulators with inversion symmetry, which were previously discussed in SRefs. [43–46]. We show how spin-stable topology refines the standard classification of topological phases by focusing on the representative example of a helical HOTI, a phase of matter whose boundary-independent bulk characterization and response have eluded earlier investigations [14–41, 47–50]. A boundary-independent characterization of helical HOTIs is urgently needed to better understand ongoing experimental and theoretical studies [19, 51–74], whose results prior to this study could only be interpreted through the language of 1D helical hinge modes, which

manifest in configurations dependent on sample details and surface physics. In SN 4 D, we show that there exist two *physically distinguishable* spin-stable, spin-resolved layer-constructable topological phases that can both be adiabatically connected to the layer construction of a helical HOTI. The first spin-stable, spin-gapped layer-constructable state is a 3D quantum spin Hall insulator (QSHI) [75], which has an extensively large topological contribution to the spin Hall conductivity in a finite sample. Previous investigations have recognized the possibility of 3D QSHI states in time-reversal-invariant 3D topological crystalline phases [75, 76].

However, there also exists a second, previously unrecognized spin-stable and spin-gapped helical HOTI formed from a time-reversal-doubled magnetic axion insulator. The time-reversal-doubled axion insulator (T-DAXI) state can specifically be viewed as a superposition of two magnetic axion insulators, where each magnetic axion insulator originates from bands within each half of the spin spectrum (*i.e.* two superposed magnetic axion insulators with time-reversed spin-orbital textures). We relate this result to the case of a 3D TI or magnetic axion insulator, which are both characterized by quantized magnetoelectric theta angles $\theta = \pi$ (where θ is defined modulo 2π) [15, 16, 31, 34, 36–39, 77–96]. In particular, θ transforms like $\mathbf{E}\cdot\mathbf{B}$, where $\mathbf{E}$ is the electric field and $\mathbf{B}$ is the magnetic field, and is hence quantized by any symmetry, such as inversion or time-reversal, that takes $\mathbf{E}\cdot\mathbf{B} \rightarrow -\mathbf{E}\cdot\mathbf{B}$. Using this well-established classification of axionic insulators, we demonstrate in SN 4 D that the spin bands in each half of the spin spectrum in a T-DAXI are characterized by a novel topological quantity: a nontrivial *partial* axion angle $\theta^{\pm} = \pi$. In the case of the T-DAXI regime of an inversion- and time-reversal-protected helical HOTI, we find that $\theta^{\pm}$ are individually quantized by inversion symmetry, and related by time-reversal. We conclude SN 4 D by showing that the adiabatic deformation between a T-DAXI and a 3D QSHI involves an intermediate spin-stable (but spin-gapless) phase with an even number of spin-Weyl points in each half of the BZ; we show that this "spin-Weyl semimetal" regime is equivalent to the "doubled strong TI" (DSTI) construction of a helical HOTI introduced in SRef. [17]. In SN 4 E, we confirm our theoretical analysis by numerically computing the nested spin-resolved Wilson loops for the model of a 3D helical HOTI introduced in SRef. [15]. We first show that the simple eight-band model exhibits a novel form of "spin-fragile" Wilson loop winding, which implies the existence of 2D insulators with fragile spin-resolved band topology. We then add trivial bands to the model and explicitly compute the partial axion angles $\theta^{\pm}$ using a spin-resolved generalization of the nested Wilson loop indicator for θ introduced in SRef. [15]. We find that the 3D helical HOTI model introduced in SRef. [31] indeed resides in the T-DAXI regime, and demonstrate that its bulk-quantized partial axion angles remain nontrivial in the presence of spin-nonconserving SOC. The calculations detailed in SN 4 E – as well as the other extensive toy-model and real-material spin-resolved and nested Wilson loop calculations in this work – were performed using the freely accessible Python package NESTED_AND_SPIN_RESOLVED_WILSON_LOOP [97], which was previously implemented and utilized for the preparation of SRefs. [8, 15], and was then greatly refined and extended to spin-resolved calculations for the present work.

In SN 5, we next return to position space to further explore the surfaces of the T-DAXI state uncovered in this work. We begin in SN 5 A by reviewing the local marker formulation of the Chern number first introduced in SRefs. [98, 99]. The Chern marker is specifically a position-space density for the Chern number in two dimensions, and gives a local contribution to the Hall conductivity. In SN 5 B we use the projected spin operator to define a *partial* Chern marker by evaluating the Chern marker for the bands in half of the spin spectrum in an insulator with a spin gap [1, 100–104]. The partial Chern marker gives the local contribution to the topological part of the spin Hall conductivity, building on the discussion in SN 3 D. For 3D systems, we then review in SN 5 C the construction of the layer-resolved Chern number introduced in SRefs. [36, 105]. For a quasi-2D slab of an insulating material, it was specifically shown in SRefs. [36, 105] that the layer-resolved Chern number gives the contribution of each layer of the slab to the total Chern number of the slab. For 3D quantum anomalous Hall insulators the layer-resolved Chern number is a nonzero (integer) constant in each layer, while for magnetic axion insulators it is zero (on average) in the bulk of the slab and quantized to half integers on the top and bottom surfaces; this reflects the half-quantized Hall conductivity at a boundary where the magnetoelectric θ angle changes by π, and represents a manifestation of the parity anomaly [36, 77, 79, 105, 106].

Building on established results for magnetic axion insulators, in SN 5 D, we then formulate a layer-resolved *partial* Chern number for insulators with a spin gap. For a quasi-2D slab of an insulating material with a spin gap, the layer-resolved partial Chern number gives the contribution of each layer of the slab to the partial Chern number of the full slab. In SN 5 E, we next numerically demonstrate that for a helical HOTI in the T-DAXI regime, the layer-resolved partial Chern number is zero (on average) in the bulk of the slab and quantized to half integers on the top and bottom surfaces. This is consistent with the fact that the partial axion angles $\theta^{\pm}$ introduced in SN 4 D each change by π at the boundary between a T-DAXI and the vacuum. This implies that each 2D surface of a 3D T-DAXI carries an anomalous half of the partial Chern number (and half the topological contribution to the spin Hall conductivity) of an isolated (lattice-regularized) 2D TI, representing a manifestation of a novel spin-resolved "partial" parity anomaly. The partial parity anomaly identified in this work is closely related to, and provides a deeper theoretical understanding of, the symmetry-enhanced fermion doubling theorems for topological crystalline insulator surface states formulated in SRefs. [20, 43, 44, 107] using the constraints imposed by crystal symmetry on noninteracting 2D lattice (tight-binding) models. Specifically, the gapless surface theories for the nonsymmorphic

Dirac insulator introduced in SRef. [107] and the "rotation-anomaly" topological crystalline insulators introduced in SRefs. [20, 43, 44] can be deformed to the gapped surface theories of inversion- and time-reversal-symmetric helical HOTIs by relaxing surface crystal symmetries [31], indicating a close relationship between the symmetry-enhanced fermion doubling theorems introduced in those works and the partial parity anomaly introduced in this work.

In SN 6, we next provide detailed proofs of the action of symmetries on the spin-resolved Wilson spectrum. We specifically provide a comprehensive discussion of the action of inversion and time-reversal symmetry on (spin-resolved) Wilson loops and nested (spin-resolved) Wilson loops.

Next, in SN 7 we review the theory of intrinsic spin Hall conductivity, and draw a connection between spin-resolved topology and the intrinsic bulk and surface spin Hall conductivity in spin-stable topological phases. First in SN 7 A we review the computational implementation of the Kubo formula for the intrinsic spin Hall conductivity, building on the formalism of SRef. [108]. With the general formalism developed, in SN 7 B we apply our tools to compute the intrinsic spin Hall conductivity of the spin-stable 2D fragile model from SRef. [8] (analyzed in SN 3 G). We show numerically that the intrinsic spin-s_z Hall conductivity is given to leading order by the spin Chern number, with corrections that grow perturbatively in the strength of spin-s_z nonconserving SOC. Finally, in SN 7 C we define a layer-resolved spin Hall conductivity for three-dimensional systems. We compute the layer-resolved spin Hall conductivity for the model of a T-DAXI analyzed in SN 5, where we show that the anomalous half-integer partial Chern numbers at the surface of a T-DAXI imply an anomalously (approximately) half-quantized surface spin Hall conductivity. We thus connect spin-resolved topology to experimentally-relevant transport coefficients in spin-gapped states.

Crucially, the spin-resolved topological machinery introduced in this work can be applied to ab-initio calculations of real materials, where we provide a brief summary in SN 8. To demonstrate this, in SN 9, we first analyze the spin-resolved topology of β-MoTe$_2$, which was identified in SRefs. [31, 50] as a candidate helical HOTI. Starting with a symmetric, Wannier-based tight-binding model obtained from ab-initio calculations (SN 9 A), we compute the spin band structure and spin-resolved Wilson loops for β-MoTe$_2$. We specifically in SN 9 B compute the spin spectrum for all possible spin orientations $s = \mathbf{s}\cdot\hat{\mathbf{n}}$ in the projected spin operator PsP. We find that for all choices of s, β-MoTe$_2$ is spin-gapless with an even number of spin-Weyl nodes in each half of the BZ. To elucidate physical signatures of the gapless spin spectrum in β-MoTe$_2$, we next compute the bulk *energy* spectrum for β-MoTe$_2$ in the presence of a strong Zeeman field directed parallel to the spin direction $s \propto s_x + s_z$, for which β-MoTe$_2$ exhibits a particularly simple spin spectrum. We observe that in the presence of a large $(\hat{\mathbf{x}} + \hat{\mathbf{z}})$-directed Zeeman field, β-MoTe$_2$ exhibits Weyl nodes in the energy spectrum that lie at almost the same locations, and carry the same chiral charges, as the spin-Weyl nodes in the spin spectrum in the absence of a magnetic field, providing further support for the analysis in SN 2 G. Lastly, we compute the surface spectral function for a finite slab of β-MoTe$_2$ in the presence of a strong Zeeman field to verify the presence of topological Fermi-arc surface states originating from the field-induced Weyl nodes, which hence serve as physical signatures of the bulk spin-Weyl semimetal state.

Finally, in addition to β-MoTe$_2$, we also in SN 10 analyze the spin-resolved topology and physical observables of α-BiBr. Previous theoretical studies [50, 52, 53] have identified α-BiBr as a rare bulk-insulating candidate helical HOTI (*i.e.* one without bulk electron and hole pockets, in contrast to β-MoTe$_2$). α-BiBr is also readily experimentally synthesized, and has exhibited signatures of 1D helical hinge modes in numerous spectroscopic and transport experiments [64–66, 68, 72, 109–113]. We use first-principles calculations to construct a symmetric, Wannier-based tight-binding model of α-BiBr as detailed in SN 10 A. We then in SN 10 B use our Wannier-based tight-binding model of α-BiBr to compute the spin gap over the full range of spin resolution directions $\hat{\mathbf{n}}$. Unlike β-MoTe$_2$, we discover that α-BiBr is *spin-gapped* over a large range of spin-resolution directions $\hat{\mathbf{n}}$. Continuing in SN 10 B, we then compute the spin-resolved Wilson loops, nested Wilson loops, and nested spin-resolved Wilson loops for α-BiBr within the spin-gapped regions of its PsP spectra. We remarkably find that α-BiBr realizes *all three* spin-resolved regimes of a helical HOTI, specifically interpolating as a function of $\hat{\mathbf{n}}$ between 3D QSHI states and the spin-Weyl and T-DAXI spin-stable states introduced in this work. α-BiBr hence represents the first known realization of a material with nontrivial, gauge-invariant partial axion angles $\theta^{\pm} = \pi$. To provide physical signatures of the spin-resolved topology in α-BiBr, we lastly in SN 10 C compute the bulk spin Hall conductivities of α-BiBr in its 3D QSHI and T-DAXI regimes. We find that the intrinsic bulk contribution to the spin Hall conductivity exhibits remarkably good agreement with the spin-resolved topology in α-BiBr, and specifically takes on nearly quantized (nearly vanishing) values in its spin-stable 3D QSHI (T-DAXI) states.

2. PROJECTED SPIN OPERATORS, SPIN SPECTRA, AND SPIN GAPS

In this section, we will review the properties of the projected spin operator that are necessary to analyze spin resolved (higher-order) topology. First, in SN 2 A we will introduce the tight-binding notation used throughout this work. In SN 2 B we will review general properties of the projected spin operator. In SN 2 C we will prove that the spectrum of the spin operator changes continuously under perturbations to the Hamiltonian. In SN 2 D we will show

how the eigenvalues of the projected spin operator impact physical observables. Moving to topology, in SN 2 E we will analytically compute the spin spectrum for a simple model of a 3D topological insulator. In SN 2 F we will show how entanglement between spin and orbital degrees of freedom can impact the spin spectrum. Finally, in SN 2 G we will show how the spin spectrum is related to the band structure (electronic energy spectrum) for a system in a strong Zeeman field.

A. Tight-Binding Notation

In this section we provide the notation for our tight-binding models. The second-quantized Fourier-transformed Hamiltonian for an (infinite or periodic) system with discrete translation symmetry is given by

$$H = \sum_{\mathbf{k},\alpha,\beta} c^\dagger_{\mathbf{k},\alpha}[H(\mathbf{k})]_{\alpha,\beta}c_{\mathbf{k},\beta}, \tag{2.1}$$

where $[H(\mathbf{k})]$ is the first-quantized Bloch Hamiltonian matrix, $\mathbf{k}$ is the crystal momentum, the summation over $\mathbf{k}$ is within the first BZ of the crystal, and

$$c^\dagger_{\mathbf{k},\alpha} = \frac{1}{\sqrt{N}}\sum_{\mathbf{R}} e^{i\mathbf{k}\cdot(\mathbf{R}+\mathbf{r}_\alpha)}c^\dagger_{\mathbf{R},\alpha}, \tag{2.2}$$

$$c_{\mathbf{k},\alpha} = \frac{1}{\sqrt{N}}\sum_{\mathbf{R}} e^{-i\mathbf{k}\cdot(\mathbf{R}+\mathbf{r}_\alpha)}c_{\mathbf{R},\alpha}, \tag{2.3}$$

where N is the number of unit cells, $c^\dagger_{\mathbf{R},\alpha}$ and $c_{\mathbf{R},\alpha}$ are the creation and annihilation operators of the (spinful) orbital labeled by α in the unit cell $\mathbf{R}$, and $\mathbf{r}_\alpha$ is the position of the (spinful) orbital labeled by α within unit cell $\mathbf{R}$. The actual position of the (spinful) orbital created by $c^\dagger_{\mathbf{R},\alpha}$ is thus $\mathbf{R}+\mathbf{r}_\alpha$. Discrete translation symmetry implies that the Hamiltonian in Supplementary Equation (SEq.) (2.1) is invariant if we shift the summation over $\mathbf{k}$ by a reciprocal lattice vector $\mathbf{G}$. In particular, we have

$$H = \sum_{\mathbf{k},\alpha,\beta} c^\dagger_{\mathbf{k}+\mathbf{G},\alpha}[H(\mathbf{k}+\mathbf{G})]_{\alpha,\beta}c_{\mathbf{k}+\mathbf{G},\beta} \tag{2.4}$$

$$= \sum_{\mathbf{k},\alpha,\beta} c^\dagger_{\mathbf{k},\alpha}e^{i\mathbf{G}\cdot\mathbf{r}_\alpha}[H(\mathbf{k}+\mathbf{G})]_{\alpha,\beta}e^{-i\mathbf{G}\cdot\mathbf{r}_\beta}c_{\mathbf{k},\beta} \tag{2.5}$$

$$= \sum_{\mathbf{k},\alpha,\beta} c^\dagger_{\mathbf{k},\alpha}[H(\mathbf{k})]_{\alpha,\beta}c_{\mathbf{k},\beta}, \tag{2.6}$$

which implies that

$$e^{i\mathbf{G}\cdot\mathbf{r}_\alpha}[H(\mathbf{k}+\mathbf{G})]_{\alpha,\beta}e^{-i\mathbf{G}\cdot\mathbf{r}_\beta} = [H(\mathbf{k})]_{\alpha,\beta}. \tag{2.7}$$

Defining the unitary matrix $[V(\mathbf{G})]$ with matrix elements

$$[V(\mathbf{G})]_{\alpha,\beta} \equiv \delta_{\alpha\beta}e^{i\mathbf{G}\cdot\mathbf{r}_\alpha}, \tag{2.8}$$

we see that the electron operators $c^\dagger_{\mathbf{k},\alpha}$ satisfy the boundary condition $c^\dagger_{\alpha,\mathbf{k}+\mathbf{G}} = c^\dagger_{\beta,\mathbf{k}}[V(\mathbf{G})]_{\beta\alpha}$. Similarly, the Bloch Hamiltonian matrix $[H(\mathbf{k})]$ satisfies

$$[V(\mathbf{G})][H(\mathbf{k}+\mathbf{G})][V(\mathbf{G})]^{-1} = [H(\mathbf{k})]. \tag{2.9}$$

Therefore, upon a shift of $\mathbf{k}\to\mathbf{k}+\mathbf{G}$, $[H(\mathbf{k})]$ transforms according to the following boundary condition

$$[H(\mathbf{k}+\mathbf{G})] = [V(\mathbf{G})]^{-1}[H(\mathbf{k})][V(\mathbf{G})], \tag{2.10}$$

which is due to the gauge choices of the electron operators in SEqs. (2.2)–(2.3). Suppose the eigenvectors of $[H(\mathbf{k})]$ are denoted as $|u_{n,\mathbf{k}}\rangle$ where n is the band index, we have the eigenvalue equation

$$[H(\mathbf{k})]\,|u_{n,\mathbf{k}}\rangle = E_{n,\mathbf{k}}\,|u_{n,\mathbf{k}}\rangle\,, \tag{2.11}$$

where $E_{n,\mathbf{k}}$ is the dispersion of the nth energy band. The boundary condition in SEq. (2.10) then implies that we can choose the boundary condition for $|u_{n,\mathbf{k}}\rangle$ as

$$|u_{n,\mathbf{k}+\mathbf{G}}\rangle = [V(\mathbf{G})]^{-1}\,|u_{n,\mathbf{k}}\rangle\,. \tag{2.12}$$

As shown in SRefs. [4, 14], such boundary conditions lead to the identification of the phases of the Wilson loop eigenvalues as the actual localized positions of (hybrid) Wannier functions within a unit cell.

B. Properties of the Projected Spin Operator

In this section, we derive several useful properties of the projected spin operator. Consider a $2N\times 2N$ matrix Bloch Hamiltonian $H(\mathbf{k})$. $H(\mathbf{k})$ acts on a Hilbert space consisting of N spin-degenerate orbitals per unit cell. Letting the Pauli matrices σ_i act on the spin degrees of freedom, we can define the spin operators

$$s_i \equiv \sigma_i \otimes \mathbb{I}_N, \tag{2.13}$$

where $\mathbb{I}_N$ is the $N\times N$ identity matrix acting in the orbital subspace of the entire Hilbert space (including both occupied and unoccupied states). In this work we will be considering the band topology of the occupied states in spinful insulators; following SRefs. [1, 7, 114] we will show that the eigenstates of the spin operator projected to the occupied bands provide a refinement of the usual notion of band topology. To formulate this precisely, let $P(\mathbf{k})$ represent the projector onto a set of "occupied" energy eigenstates at $\mathbf{k}$. We can then form the projected spin operator

$$PsP \equiv P(\mathbf{k})\hat{\mathbf{n}}\cdot\mathbf{s}P(\mathbf{k}), \tag{2.14}$$

for any choice of unit vector $\hat{\mathbf{n}}$. SEq. (2.14) defines the projected spin operator for both infinite periodic and finite systems, where for finite systems $\mathbf{k}$ indexes only the periodic directions in the BZ (*i.e.* for a system finite in three spatial directions with open boundary conditions, there is no $\mathbf{k}$ dependence). For notational convenience, we will suppress the $\mathbf{k}$-dependence of PsP when our discussion applies to both finite and infinite systems. When we are considering translationally-invariant systems, the projection operator P will be a $2N\times 2N$ $\mathbf{k}$-dependent matrix where $2N$ is the number of spinful orbitals within the unit cell. The spin operator s_i will be a $\mathbf{k}$-independent $2N\times 2N$ matrix. When we are considering finite systems with open boundary conditions, the projection operator P and the spin operator s_i will both be $2N\times 2N$ $\mathbf{k}$-independent matrices, where $2N$ is the number of spinful orbitals in the entire finite system.

For periodic systems, the spectrum of PsP forms a set of well-defined bands; from SEq. (2.12) we have that

$$P(\mathbf{k}+\mathbf{G})sP(\mathbf{k}+\mathbf{G}) = V^{-1}(\mathbf{G})P(\mathbf{k})[V(\mathbf{G})sV^{-1}(\mathbf{G})]P(\mathbf{k})V(\mathbf{G}). \tag{2.15}$$

For the spectrum of PsP to be periodic, we must have

$$[V(\mathbf{G}), s] = 0. \tag{2.16}$$

Physically, this means that basis orbitals come in time-reversed pairs with opposite spins. This is consistent with the fact that the Hilbert space of a solid derives from pairs of orbitals at the same position with opposite spins (*i.e.* atomic orbitals), and so the periodicity constraint in SEq. (2.16) does not place any unreasonable constraints on the classes of systems we consider in this work. In particular, SEq. (3.11) must hold for time-reversal invariant systems. We can thus compute the PsP spectrum for any approximate model of a material [*i.e.* from a density functional theory (DFT) calculation] provided the Hilbert space of the model is consistent with SEq. (2.16).

We will now derive several properties of the spectrum of PsP that will be useful for the remainder of this work. Because $(\hat{\mathbf{n}}\cdot\mathbf{s})^2 = \mathbb{I}_{2N}$, we know that the eigenvalues of $\hat{\mathbf{n}}\cdot\mathbf{s}$ are all ± 1. This places several constraints on the eigenvalues of PsP. First, consider an eigenstate $|\psi\rangle$ of PsP with eigenvalue λ, such that

$$P|\psi\rangle = |\psi\rangle, \tag{2.17}$$

$$\langle\psi|\psi\rangle = 1, \tag{2.18}$$

$$PsP|\psi\rangle = \lambda|\psi\rangle. \tag{2.19}$$

SEqs. (2.17)–(2.19) imply that

$$s|\psi\rangle = \lambda|\psi\rangle + |\tilde{\phi}\rangle, \tag{2.20}$$

where

$$Q|\tilde{\phi}\rangle \equiv (\mathbb{I}_{2N} - P)|\tilde{\phi}\rangle = |\tilde{\phi}\rangle, \tag{2.21}$$

$$\langle\tilde{\phi}|\tilde{\phi}\rangle = \langle\psi|\, sQs\, |\psi\rangle \equiv |\alpha|^2. \tag{2.22}$$

We then have

$$\begin{aligned} 1 &= \langle\psi|\psi\rangle \\ &= \langle\psi|s^2|\psi\rangle \\ &= (\lambda\langle\psi| + \langle\tilde{\phi}|)(\lambda|\psi\rangle + |\tilde{\phi}\rangle) \\ &= |\lambda|^2 + |\alpha|^2. \end{aligned} \tag{2.23}$$

From SEq. (2.23) we deduce immediately that $|\lambda| \leq 1$. Furthermore, $\lambda = 1$ if and only if $|\psi\rangle$ is an eigenstate of s. Going further, if $|\lambda| < 1$, then we can define a normalized state

$$|\phi\rangle \equiv \frac{1}{|\alpha|}|\tilde{\phi}\rangle \tag{2.24}$$

such that

$$s|\psi\rangle = \lambda|\psi\rangle + |\alpha||\phi\rangle. \tag{2.25}$$

Acting with s again we see that

$$\begin{aligned} s^2|\psi\rangle = |\psi\rangle &= \lambda s|\psi\rangle + |\alpha|s|\phi\rangle \\ &= |\lambda|^2|\psi\rangle + \lambda|\alpha||\phi\rangle + |\alpha|s|\phi\rangle. \end{aligned} \tag{2.26}$$

In order to satisfy the equality in SEq. (2.26), we must have that

$$s|\phi\rangle = |\alpha||\psi\rangle - \lambda|\phi\rangle. \tag{2.27}$$

From which we deduce that

$$QsQ|\phi\rangle \equiv Q(\mathbf{k})\hat{\mathbf{n}}\cdot\mathbf{s}Q(\mathbf{k})|\phi\rangle = -\lambda|\phi\rangle, \tag{2.28}$$

i.e. for each eigenstate of PsP within the image of P with eigenvalue λ with $|\lambda| < 1$, there exists an eigenstate of QsQ within the image of Q with eigenvalue $-\lambda$, and vice versa.

SEq. (2.28) shows that given $|\psi\rangle$ in the image of P with $PsP|\psi\rangle = \lambda|\psi\rangle$ where $|\lambda| < 1$, there exists a state $|\phi\rangle$ in the image of Q satisfying $QsQ|\phi\rangle = -\lambda|\phi\rangle$ where $Q = 1 - P$. In particular, $|\phi\rangle$ satisfies $s|\phi\rangle = |\alpha||\psi\rangle - \lambda|\phi\rangle$, where $|\alpha| = \sqrt{1-\lambda^2}$. Next, we will show that if we have $|\phi_1\rangle$ and $|\phi_2\rangle$—both in the image of Q—that are constructed from two orthogonal states $|\psi_1\rangle$ and $|\psi_2\rangle$ in the image of P with non-unit PsP eigenvalues, then $|\phi_1\rangle$ and $|\phi_2\rangle$ are orthogonal. To see this, we can rewrite the overlap $\langle\phi_1|\phi_2\rangle$ as

$$\langle\phi_1|\phi_2\rangle = \langle\phi_1|s^2|\phi_2\rangle = (\langle\phi_1|s)\,(s|\phi_2\rangle) \tag{2.29}$$

$$= (|\alpha_1|\langle\psi_1| - \lambda_1\langle\phi_1|)\,(|\alpha_2||\psi_2\rangle - \lambda_2|\phi_2\rangle) \tag{2.30}$$

$$= |\alpha_1||\alpha_2|\langle\psi_1|\psi_2\rangle - |\alpha_1|\lambda_2\langle\psi_1|\phi_2\rangle - \lambda_1|\alpha_2|\langle\phi_1|\psi_2\rangle + \lambda_1\lambda_2\langle\phi_1|\phi_2\rangle \tag{2.31}$$

$$= \lambda_1\lambda_2\langle\phi_1|\phi_2\rangle, \tag{2.32}$$

where we have used $\langle\psi_1|\psi_2\rangle = 0$, $\langle\psi_1|\phi_2\rangle = 0$, and $\langle\phi_1|\psi_2\rangle = 0$. This then implies that

$$(1 - \lambda_1\lambda_2)\,\langle\phi_1|\phi_2\rangle = 0. \tag{2.33}$$

Since both λ_1 and λ_2 are non-unit PsP eigenvalues, we have $|\lambda_1\lambda_2| = |\lambda_1||\lambda_2| < 1$ such that $1 - \lambda_1\lambda_2 \neq 0$. This then implies that

$$\langle\phi_1|\phi_2\rangle = 0, \tag{2.34}$$

which is the desired result.

It will often be helpful to consider the matrix elements of PsP between the N_{occ} states in the image of P. Choosing a basis $\{|n\rangle\,|n = 1\ldots N_{\text{occ}}\}$ for the image of P, we can introduce the $N_{\text{occ}} \times N_{\text{occ}}$ *reduced spin matrix*

$$[s_{\text{reduced}}]_{m,n} = \langle m|s|n\rangle, \tag{2.35}$$

where N_{occ} is the number of states in the image of P, which in many realistic calculations corresponds to the number of occupied valence bands (ignoring core states). The nonzero eigenvalues of $[s_{\text{reduced}}]$ are in one-to-one correspondence with the nonzero eigenvalues of PsP. However, PsP will have more zero eigenvalues than $[s_{\text{reduced}}]$, because the zero eigenspace of PsP includes all states in the image of Q. Since these additional zero eigenvalues provide no new information, we will see in SN 3 and 4 that it is often advantageous to diagonalize $[s_{\text{reduced}}]$ to determine the spin spectrum. Throughout this work we will use interchangeably the terms "reduced s eigenvalues", namely the eigenvalues of the matrix $[s_{\text{reduced}}]$, "PsP eigenvalues" where we will ignore the zero PsP eigenvalues arising from states in the image of Q, and "(projected) spin eigenvalues". In addition, for a translationally invariant system, we will call the PsP eigenvalues as a function of crystal momentum $\mathbf{k}$ the "spin (s) band structure".

Going further, for each eigenvalue λ of PsP with $|\lambda| < 1$, we can explicitly reconstruct two eigenstates of s. This will be important when we consider systems with SOC. In particular, combining SEqs. (2.20) and (2.27), we can write

$$s(a|\psi\rangle + b|\phi\rangle) = (|\psi\rangle, |\phi\rangle)\begin{pmatrix} \lambda & |\alpha| \\ |\alpha| & -\lambda \end{pmatrix}\begin{pmatrix} a \\ b \end{pmatrix} \equiv (|\psi\rangle, |\phi\rangle)\mathbf{M}\begin{pmatrix} a \\ b \end{pmatrix}. \tag{2.36}$$

We can see that SEq. (2.36) is a 2×2 matrix equation, where the matrix $\mathbf{M}$ has trace $\mathrm{tr}(\mathbf{M}) = 0$ and determinant $\det \mathbf{M} = -1$. By diagonalizing $\mathbf{M}$, we obtain two eigenstates of s with eigenvalues ± 1 given by $|\xi\rangle = (2 - 2\xi\lambda)^{-1/2}(|\alpha|\,|\psi\rangle + (-\lambda + \xi)\,|\phi\rangle)$ where $\xi = \pm 1$. This shows concretely how in the presence of SOC, spin eigenstates can be reconstructed from linear combinations of occupied and unoccupied states. A particularly interesting case is when $|\psi\rangle$ is a null-eigenstate of $P(\mathbf{k})sP(\mathbf{k})$, so that $\lambda = 0, |\alpha| = 1$. In this case, $|\pm\rangle = \frac{1}{\sqrt{2}}(|\psi\rangle \pm |\phi\rangle)$ is an eigenstate of s with eigenvalue ± 1. Similarly, if we have eigenstates $|\pm\rangle$ of $PsQ + QsP$ with eigenvalue ± 1, we can see from the block structure of SEq. (2.36) that $\frac{1}{\sqrt{2}}(|+\rangle \pm |-\rangle)$ are null eigenstates of PsP and QsQ. We see then that $[s_{\text{reduced}}]$ has a null eigenvalue if and only if $PsQ + QsP$ has a pair of eigenvalues of modulus 1.

For the bulk of this work, we will be primarily interested in time-reversal invariant systems, with time-reversal operator $\mathcal{T}$ satisfying $\mathcal{T}^2 = -\mathbb{I}_{2N}$. Since $\mathcal{T} s_i \mathcal{T}^{-1} = -s_i$, time-reversal symmetry requires that $\mathcal{T}P(\mathbf{k})(\hat{\mathbf{n}}\cdot\mathbf{s})P(\mathbf{k})\mathcal{T}^{-1} = -P(-\mathbf{k})sP(-\mathbf{k})$. In particular, if $P(\mathbf{k})sP(\mathbf{k})|\psi\rangle = \lambda|\psi\rangle$, then $P(-\mathbf{k})sP(-\mathbf{k})\mathcal{T}|\psi\rangle = -\lambda\mathcal{T}|\psi\rangle$. An example of spin bands with $\mathcal{T}$ symmetry is shown in Supplementary Figure (SFig.) 1(b). If we additionally have inversion symmetry $\mathcal{I}$ satisfying $\mathcal{I}^2 = \mathbb{I}_{2N}$, then $\mathcal{I}P(\mathbf{k})sP(\mathbf{k})\mathcal{I}^{-1} = P(-\mathbf{k})sP(-\mathbf{k})$. Combining the constraints on PsP from $\mathcal{I}$ and $\mathcal{T}$ then implies that for a system with inversion and (spinful) time-reversal symmetry, the eigenvalues of $P(\mathbf{k})sP(\mathbf{k})$ come in $\pm\lambda$ pairs. In other words, the spectrum of PsP has an effective "chiral" symmetry if the bulk has $\mathcal{IT}$ symmetry, which is demonstrated in SFig. 1(c).

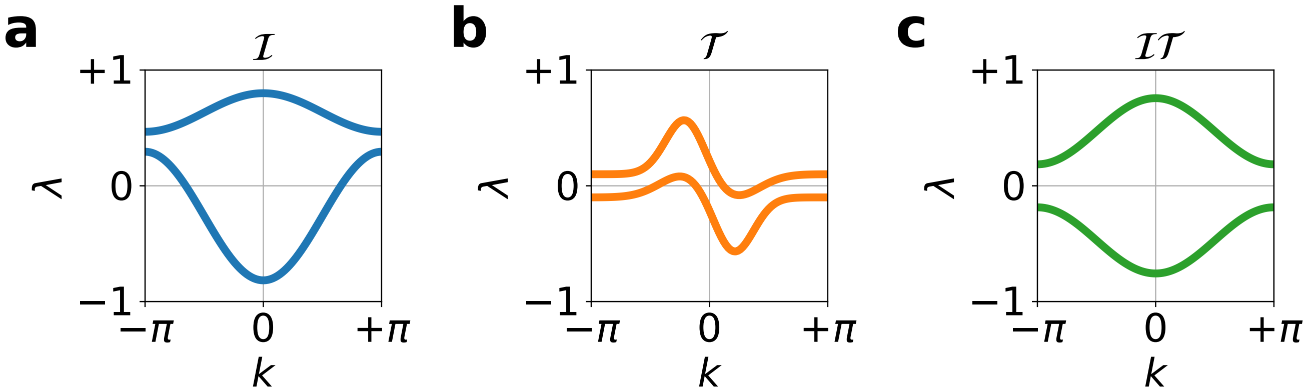

Supplementary Figure 1. Example of spin band structures for 1D systems with (a) inversion ($\mathcal{I}$), (b) time-reversal ($\mathcal{T}$), and (c) $\mathcal{IT}$ symmetry. The number of occupied energy bands is assumed to be 2, hence all of (a), (b), and (c) contain two spin bands. We denote the set of PsP eigenvalues at crystal momentum k as $\{\lambda_n(k)\}$ where $n = 1, 2$. In (a) the PsP eigenvalues satisfy $\{\lambda_n(k)\} = \{\lambda_n(-k)\}$ due to $\mathcal{I}$ symmetry. In (b) the PsP eigenvalues satisfy $\{\lambda_n(k)\} = \{-\lambda_n(-k)\}$ due to $\mathcal{T}$ symmetry. Finally, in (c) the PsP eigenvalues satisfy $\{\lambda_n(k)\} = \{-\lambda_n(k)\}$ due to the combination of $\mathcal{I}$ and $\mathcal{T}$ symmetry. In all cases, there is a gap at every $\mathbf{k}$ between the two spin bands, and so the spin gap is open in (a), (b), and (c). In particular, (b) shows that for a system with time-reversal symmetry only, the spin gap can be open throughout the BZ even though the spin bands cross zero, as long as there is no degeneracy between the $\frac{1}{2}\mathrm{rank}[P(k)]$ spin bands with largest spin eigenvalue, and the $\frac{1}{2}\mathrm{rank}[P(k)]$ spin bands with smallest spin eigenvalue. In contrast, for a system with $\mathcal{IT}$ symmetry, the spin gap is open if and only if there is no degeneracy of spin bands at zero PsP eigenvalues, as shown in (c), such that $\mathrm{rank}[P_+(k)] = \mathrm{rank}[P_-(k)] = \frac{1}{2}\mathrm{rank}[P(k)]$. $P_+(k)$ and $P_-(k)$ in (c) are defined to be the projectors onto the positive and negative eigenspace of $P(k)sP(k)$, respectively. In addition, since we have $\{\lambda_n(k)\} = \{-\lambda_n(k)\}$ in (c), the corresponding spin band structure has an effective "chiral" symmetry.

To use PsP to define partial topological invariants, *i.e.* topological invariants for spectrally isolated PsP eigenstates [2], we would like to divide the occupied states into two groups based on their PsP eigenvalues. Inversion and time-reversal symmetry give us two natural ways to do this. For systems with time-reversal and inversion symmetry, we can define the spin gap at each crystal momentum $\mathbf{k}$ as $\Delta_s(\mathbf{k}) \equiv \min_{|\psi\rangle\in\mathrm{Image}[P(\mathbf{k})]} |\langle\psi|P(\mathbf{k})sP(\mathbf{k})|\psi\rangle|$. Using the Rayleigh characterization of eigenvalues [115], we see that $\Delta_s(\mathbf{k}) \equiv |\lambda_{\min}(\mathbf{k})|$, where $\lambda_{\min}(\mathbf{k})$ is the eigenvalues of $P(\mathbf{k})sP(\mathbf{k})$ with the smallest absolute value. When the spin gap is open, $\Delta_s(\mathbf{k}) > 0$ and we can partition $P(\mathbf{k}) = P_+(\mathbf{k}) + P_-(\mathbf{k})$ for all momenta $\mathbf{k}$, where $P_\pm(\mathbf{k})$ projects respectively onto the positive (negative) eigenspace of $P(\mathbf{k})sP(\mathbf{k})$. We remark that this process of decomposing the occupied space into spin-resolved sectors through

$P(\mathbf{k}) = P_+(\mathbf{k}) + P_-(\mathbf{k})$ is closely related to the sublattice or spin sector resolution employed in SRefs. [116–118] to analyze "hidden" spin-orbital textures. For systems with time-reversal and inversion symmetry, we have immediately that $\mathrm{rank}[P_+(\mathbf{k})] = \mathrm{rank}[P_-(\mathbf{k})] = \frac{1}{2}\mathrm{rank}[P(\mathbf{k})]$ when the spin gap is open. This definition of the spin gap corresponds to placing the "spin Fermi energy" globally at zero, and demanding that no spin bands cross it, see for instance SFig. 1(c). Even without inversion symmetry, we have that for an insulating $\mathcal{T}$-invariant system there are an even number of spin bands. We can for such systems define P_+ to be the projector onto the $\mathrm{rank}(P)/2$ spin bands with the largest spin eigenvalues, and P_- to be the projector onto the $\mathrm{rank}(P)/2$ spin bands with the smallest spin eigenvalues. We then say that a spin gap is open when there is no degeneracy between states in $\mathrm{Image}(P_+)$ and states in $\mathrm{Image}(P_-)$ at any $\mathbf{k}$. Note that for systems with both inversion and time-reversal symmetry, this construction of P_+ and P_- coincides with that of the previous paragraph. Analogous to direct gaps in band structures, it is possible that with $\mathcal{T}$ symmetry alone a spin gap can be opened even if a spin band crosses zero, see for instance SFig. 1(b). From this construction, it follows that

$$\mathcal{T}P_\pm(\mathbf{k})\mathcal{T}^{-1} = P_\mp(-\mathbf{k}). \tag{2.37}$$

For simple models with additional emergent symmetries at low energies (for example the approximate chiral symmetry in graphene [119] and polyacetylene [120], or valley conservation in twisted bilayer systems [121]) there are several additional properties of PsP that we can exploit. First, consider insulating models with a unitary chiral symmetry $\Pi H(\mathbf{k})\Pi^{-1} = -H(\mathbf{k})$. In this case $P(\mathbf{k})$ projects onto the set of eigenstates with negative energies. In such a system, we have that $P(\mathbf{k})sP(\mathbf{k}) = \Pi Q(\mathbf{k})\Pi^{-1}s\Pi Q(\mathbf{k})\Pi^{-1}$. If, furthermore, $[\Pi, s] = 0$, then this implies that $P(\mathbf{k})sP(\mathbf{k})$ and $Q(\mathbf{k})sQ(\mathbf{k})$ are isospectral. To be more specific, if $|\psi\rangle \in \mathrm{Image}[P(\mathbf{k})]$ is an eigenstate of $P(\mathbf{k})sP(\mathbf{k})$ with eigenvalue λ, then $\Pi|\psi\rangle \in \mathrm{Image}[Q(\mathbf{k})]$ is an eigenstate of $Q(\mathbf{k})sQ(\mathbf{k})$ with the same eigenvalue λ. Combining this with SEq. (2.28), we see that chiral symmetry forces every non-unit eigenvalue λ of $P(\mathbf{k})sP(\mathbf{k})$ to have a partner $-\lambda$. The spin gap in a chiral-symmetric system with $[\Pi, s] = 0$ can then only close when a pair of $P(\mathbf{k})sP(\mathbf{k})$ eigenvalues crosses 0. This implies that $\mathrm{rank}[P_+(\mathbf{k})] = \mathrm{rank}[P_-(\mathbf{k})]$ at $\mathbf{k}$ points that do not have $P(\mathbf{k})sP(\mathbf{k})$ eigenvalues with modulus 1 for a model of an insulator with chiral symmetry.

On the other hand, since

$$0 = \mathrm{Tr}[s] = \mathrm{Tr}[(P+Q)s(P+Q)] \tag{2.38}$$
$$= \mathrm{Tr}[PsP] + \mathrm{Tr}[QsQ], \tag{2.39}$$

where we have used $PQ = 0$ and the cyclic property of trace, we can obtain

$$\mathrm{Tr}[PsP] = -\,\mathrm{Tr}[QsQ]. \tag{2.40}$$

In other words, the summations of all the eigenvalues of PsP and QsQ are opposite to each other. Combining this with the fact that if $|\psi\rangle \in \mathrm{Image}(P)$ is an eigenstate of PsP with an non-unit eigenvalue λ, namely $|\lambda| < 1$, there is a corresponding eigenstate $|\phi\rangle \in \mathrm{Image}(Q)$ of QsQ with eigenvalue $-\lambda$, we deduce that the summations of eigenvalues with modulus 1, namely $|\lambda| = 1$, of PsP and QsQ are also opposite to each other. Denoting $n_\pm^{P/Q}$ as the number of $+1/-1$ eigenvalues in the PsP/QsQ spectrum, this means that

$$n_+^Q - n_-^Q = -\left(n_+^P - n_-^P\right). \tag{2.41}$$

Furthermore, if we denote $\mathrm{rank}\,(P)$ and $\mathrm{rank}\,(Q)$ as r_P and r_Q respectively, using the fact that there is a one-to-one correspondence between the eigenstates of PsP and QsP with non-unit eigenvalues, it can be shown that

$$\left(n_+^Q + n_-^Q\right) - \left(n_+^P + n_-^P\right) = r_Q - r_P. \tag{2.42}$$

In other words, the total number of eigenvalues of PsP and QsQ with modulus 1 will be differ by $\mathrm{rank}(P) - \mathrm{rank}(Q)$. Combining SEqs. (2.41)–(2.42), we can show that

$$n_+^Q = \frac{1}{2}\left(r_Q - r_P\right) + n_-^P, \tag{2.43}$$
$$n_-^Q = \frac{1}{2}\left(r_Q - r_P\right) + n_+^P. \tag{2.44}$$

Crucially, SEqs. (2.43)–(2.44) are generic statements *independent of the symmetries of the system.* Notice that, unlike the case for non-unit eigenvalues, there is no one-to-one correspondence between the eigenstates of PsP and QsQ with eigenvalues of modulus 1. This can be understood from the fact that $n_+^Q + n_-^Q$ is not necessarily equal to $n_+^P + n_-^P$,

as is demonstrated in SEq. (2.42). For an insulator with a unitary chiral symmetry Π satisfying $[\Pi, s] = 0$, if we define P and Q as the projectors to all the negative and positive energy eigenstates respectively, we necessarily have $\mathrm{rank}\,(P) = \mathrm{rank}\,(Q)$ such that according to SEqs. (2.43)–(2.44) we obtain

$$n_+^Q = n_-^P, \tag{2.45}$$

$$n_-^Q = n_+^P. \tag{2.46}$$

From the perspective of non-unit $P(\mathbf{k})sP(\mathbf{k})$ eigenvalues, chiral (Π) and $\mathcal{IT}$ symmetry act in the same way provided $[\Pi, s] = 0$: they both map one state $|\psi\rangle \in \mathrm{Image}[P(\mathbf{k})]$ with eigenvalue λ to another state $|\tilde{\psi}\rangle \in \mathrm{Image}[P(\mathbf{k})]$ with eigenvalue $-\lambda$. However, Π and $\mathcal{IT}$ are fundamentally different symmetries: Π is a unitary symmetry while $\mathcal{IT}$ is an antiunitary symmetry. For example, a nearest-neighbor model of graphene in the presence of a Zeeman field without SOC [122] has a Π symmetry with $[\Pi, s] = 0$, while the Kane-Mele model [6] has $\mathcal{I}$, $\mathcal{T}$, and thus $\mathcal{IT}$ symmetries. Our discussion about how unitary chiral symmetry constrains the PsP spectrum is not just applicable to model systems. Recently, the method of topological quantum chemistry [123] has been used to identify stoichiometric crystalline materials with (nearly) flat bands [124–130]. These materials usually have a bipartite lattice, and have exact or approximate chiral symmetry. Although the application of our method to extract out spin-resolved topology in such materials is beyond the scope of this paper, we expect that the study of spin-resolved topology in spin-orbit coupled stoichiometric solid state materials with (nearly) flat bands, as well as generalizations to sublattice-resolved topology, will be a fruitful direction to explore. Specifically, our approach can be generalized to any operator which, like s, has eigenvalues ± 1 only. As such, our methods can be applied to analyze orbital- or sublattice-resolved topology as well.

If the Bloch Hamiltonian $H(\mathbf{k})$ can be written in "Dirac form"

$$H(\mathbf{k}) = \epsilon(\mathbf{k}) \sum_i \hat{d}_i(\mathbf{k})\Gamma_i \tag{2.47}$$

with $\{\Gamma_i, \Gamma_j\} = 2\delta_{ij}$ and $\sum_i [\hat{d}_i(\mathbf{k})]^2 = 1$, then we can oftentimes analytically compute the spectrum of PsP. For a Dirac model [SEq. (2.47)], the projector $P(\mathbf{k})$ onto the valence bands has the explicit form

$$P(\mathbf{k}) = \frac{1}{2}(\mathbb{I}_{2N} - \sum_i \hat{d}_i(\mathbf{k})\Gamma_i) \tag{2.48}$$

such that

$$P(\mathbf{k})sP(\mathbf{k}) = \frac{1}{4}\left(s - \sum_i \{s, d_i(\mathbf{k})\Gamma_i\} + \sum_{i,j} d_i(\mathbf{k})d_j(\mathbf{k})\Gamma_i s \Gamma_j \right). \tag{2.49}$$

While SEq. (2.49) can be efficiently computed for models with small Hilbert spaces, if we are only interested in the non-unit ($|\lambda| \neq 1$) eigenspace of $P(\mathbf{k})sP(\mathbf{k})$ we can simplify the computation even further by considering

$$P(\mathbf{k})sP(\mathbf{k}) - Q(\mathbf{k})sQ(\mathbf{k}) = -\frac{1}{2}\sum_i d_i(\mathbf{k})\{s, \Gamma_i\}. \tag{2.50}$$

From SEq. (2.28), we see that eigenvalues of $P(\mathbf{k})sP(\mathbf{k}) - Q(\mathbf{k})sQ(\mathbf{k})$ with $|\lambda| < 1$ are doubly degenerate, and correspond to the non-unit eigenvalues of $P(\mathbf{k})sP(\mathbf{k})$.

Crucially, the utility of considering $PsP - QsQ$ as a proxy for PsP extends beyond Dirac models and to the analysis of real materials with larger single-particle Hilbert spaces. This is because $PsP - QsQ$ eigenstates are all s eigenstates. To see this, let us evaluate

$$\begin{aligned}
[s, PsP - QsQ] &= sPsP - PsPs - sQsQ + QsQs, \\
&= (P+Q)sPsP - PsPs(P+Q), \\
&- (P+Q)sQsQ + QsQs(P+Q), \\
&= QsPsP - PsPsQ - PsQsQ + QsQsP, \\
&= Qs(P+Q)sP - Ps(P+Q)sQ, \\
&= Qs^2P - Ps^2Q = 0,
\end{aligned} \tag{2.51}$$

and so we can simultaneously diagonalize $PsP - QsQ$ and s. Since the non-unit spectrum of PsP coincides with that of $PsP - QsQ$, we can identify points at which the spin gap closes by diagonalizing $PsP - QsQ$ within the basis of

spin eigenstates. This is consistent with what we showed in SEqs. (2.28) and (2.36), since we can take eigenstates of PsP and $-QsQ$ both with eigenvalue λ and reconstruct s eigenstates. Moreover, to check whether the spin gap is closed in PsP, we *only* need to diagonalize $PsP - QsQ$ within the basis of spin s eigenstates with either $+1$ or -1 eigenvalues.

Interestingly, $PsP - QsQ$ can also be related to the (spectrally flattened) Hamiltonian projected into a single spin sector. To see this, first note that

$$\begin{aligned} PsP - QsQ &= PsP - (1-P)s(1-P), \\ &= s + \{s, P\}, \\ &= \{s, \frac{1}{2} + P\}. \end{aligned} \tag{2.52}$$

Let us now introduce

$$P_\uparrow = \frac{1}{2}(1+s), \tag{2.53}$$

$$P_\downarrow = \frac{1}{2}(1-s), \tag{2.54}$$

which project onto spin-up and spin-down eigenstates, respectively. Inserting $s = P_\uparrow - P_\downarrow$ into SEq. (2.52), we find

$$PsP - QsQ = \{P_\uparrow - P_\downarrow, 1/2 + P\} \tag{2.55}$$

$$= P_\downarrow(1-2P)P_\downarrow - P_\uparrow(1-2P)P_\uparrow, \tag{2.56}$$

where we have used the fact that $1 = P_\uparrow + P_\downarrow$. Let us introduce the spin-projected correlation matrices

$$C_\uparrow = P_\uparrow P P_\uparrow, \tag{2.57}$$

$$C_\downarrow = P_\downarrow P P_\downarrow. \tag{2.58}$$

Then we see that

$$PsP - QsQ = P_\downarrow(1-2C_\downarrow)P_\downarrow - P_\uparrow(1-2C_\uparrow)P_\uparrow. \tag{2.59}$$

The quantity $C_\uparrow$ was previously introduced in SRefs. [131–133] to study "spin entanglement cuts" in topological insulators. Here we see that $C_\uparrow$ is directly related to the projected spin operator; in SN 2 G we will show that PsP and $P_\uparrow - 2C_\uparrow$ are in fact isospectral.

C. Effects of Perturbations on the Spin Gap

In this section, we examine the effects of perturbations $\delta H(\mathbf{k})$ on the spectrum of $P(\mathbf{k})sP(\mathbf{k})$. It is crucial to establish the perturbative stability of the $P(\mathbf{k})sP(\mathbf{k})$ spectrum in order to define a robust notion of (partial) topology of the $P(\mathbf{k})sP(\mathbf{k})$. In particular, we will establish in this section that the spin spectrum changes smoothly and continuously in response to perturbations of the Hamiltonian (*e.g.* in response to an external magnetic field or perturbations to the form of SOC). To do so, we first need to understand the effect of a perturbation $\delta H(\mathbf{k})$ on the projector $P(\mathbf{k})$. Let us denote $H(\mathbf{k}) = H_0 + \delta H$, and $P(\mathbf{k}) = P_0 + \delta P$. Assume also that the states in the image of P_0 are separated by an energy gap Δ from states in the image of Q_0, and that the perturbation δH is sufficiently weak that no new degeneracies are created between PsP bands. In this case, we can express the projector P in terms of a contour integral

$$P = -\frac{1}{2\pi i}\oint_\Gamma \frac{dE}{H_0 + \delta H - E} \equiv -\frac{1}{2\pi i}\oint_\Gamma G(E)dE, \tag{2.60}$$

where the integral is taken around a contour Γ in the complex plane that encloses all of energies E_i^- of the states in the image of P_0, and none of the energies E_i^+ of the states in the image of Q_0. Notice that E_i^- and E_i^+ are all of the eigenvalues of the unperturbed Hamiltonian H_0. The resolvent (single-particle Green's function) $G(E)$ satisfies the Dyson equation

$$\delta H = G^{-1}(E) - G_0^{-1}(E), \tag{2.61}$$

where the unperturbed resolvent is

$$G_0(E) = \frac{1}{H_0 - E}. \tag{2.62}$$

Solving the Dyson equation, we find

$$G(E) = G_0(1 - \delta H G) = G_0(1 + \delta H G_0)^{-1} = G_0 \sum_{n=0}^{\infty} (-1)^n (\delta H G_0)^n. \tag{2.63}$$

This series expansion gives us a perturbation series for the projection operator

$$P = \sum_{n=0}^{\infty} P_n, \tag{2.64}$$

$$P_n = (-1)^{n+1} \frac{1}{2\pi i} \oint dE G_0(E)[\delta H G_0(E)]^n. \tag{2.65}$$

By inserting a complete set of states, each term P_n can be computed by elementary contour integration. For instance, the first-order correction P_1 takes the form

$$\begin{aligned} P_1 &= \frac{1}{2\pi i} \oint dE \frac{1}{H_0 - E} \delta H \frac{1}{H_0 - E} \\ &= \frac{1}{2\pi i} \oint dE \sum_{nm} \frac{|\psi_n\rangle\langle\psi_n|\delta H|\psi_m\rangle\langle\psi_m|}{(E_n - E)(E_m - E)}, \end{aligned} \tag{2.66}$$

where the sum runs over all eigenstates of H_0 whose eigenvalues are denoted as E_n. We can evaluate the contour integral in SEq. (2.66) by first noting that if $|\psi_n\rangle$ and $|\psi_m\rangle$ are both unoccupied states or both occupied states, then the contour integral vanishes. In the former case, $P_0 |\psi_n\rangle = P_0 |\psi_m\rangle = 0$ and all poles lie outside the contour of integration. In the latter case both poles are inside the integration contour, but have residue equal in magnitude and opposite in sign. Also, in the latter case, even though we will encounter $E_n = E_m$ when $n = m$ and when there are degenerate energy eigenvalues, the function $1/(E_n - E)^2$ has a second order pole and so its residue still vanishes. With this restriction, we can use the residue theorem to evaluate the contour integral to find

$$P_1 = -\sum_{nm} \frac{|\psi_n\rangle\langle\psi_n| (Q_0 \delta H P_0 - P_0 \delta H Q_0) |\psi_m\rangle\langle\psi_m|}{E_n - E_m}. \tag{2.67}$$

Given an M-th order approximation $\overline{P} = \sum_{n=0}^{M} P_n$ to P, we can construct the M-th order perturbed projected spin operator $\overline{PsP} = \sum_{n=0}^{M} \sum_{m=n}^{M} P_n s P_{M-m}$. We can then use standard perturbation theory to look at corrections to the eigenvalues $\lambda = \lambda_0 + \lambda_1 + \cdots + \lambda_M$ of $\overline{PsP}$.

As a concrete example, let us consider the first-order correction to a non-degenerate eigenvalue λ of PsP. Let $|\psi\rangle$ denote the corresponding unperturbed normalized eigenstate of $P_0 s P_0$ satisfying $P_0 s P_0 |\psi\rangle = \lambda_0 |\psi\rangle$ and $P_0|\psi\rangle = |\psi\rangle$. Then, from the non-degenerate perturbation theory, the first-order correction λ_1 to λ_0 is $\langle\psi| (P_1 s P_0 + P_0 s P_1) |\psi\rangle$, such that

$$\lambda_1 = \langle\psi|P_1 s|\psi\rangle + \langle\psi|s P_1|\psi\rangle \tag{2.68}$$

$$= |\alpha|(\langle\psi|P_1|\phi\rangle + \langle\phi|P_1|\psi\rangle), \tag{2.69}$$

where $|\alpha| = \sqrt{1 - |\lambda_0|^2}$, $|\phi\rangle$ is constructed using SEqs. (2.20) and (2.24), and we have used the fact that P_1 is block-off-diagonal [see SEq. (2.67)]. Simplifying further, we find

$$\lambda_1 = -|\alpha| \sum_{\substack{n \in \text{unocc} \\ m \in \text{occ}}} \frac{\langle\phi|\psi_n\rangle\langle\psi_n|\delta H|\psi_m\rangle\langle\psi_m|\psi\rangle + \langle\psi|\psi_m\rangle\langle\psi_m|\delta H|\psi_n\rangle\langle\psi_n|\phi\rangle}{E_n - E_m}. \tag{2.70}$$

In particular, it can be shown that if $|\lambda_0| = 1$, the first-order correction vanishes, namely $\lambda_1 = 0$. From this viewpoint, a completely spin-polarized state $|\psi\rangle$ satisfying $P_0|\psi\rangle = |\psi\rangle$, $P_0 s P_0|\psi\rangle = \pm|\psi\rangle$, and in fact $s|\psi\rangle = \pm 1|\psi\rangle$, is more robust against perturbation compared with those states $|\psi\rangle$ with $|\lambda_0| < 1$. Let us specialize to a mathematically

interesting case where $\delta H = g\hat{\mathbf{n}} \cdot \mathbf{s} = gs$ for some small coupling constant g. In this case, we can apply the Cauchy-Schwarz inequality to derive the bound

$$|\lambda_1| \leq 2\frac{|g|}{\Delta}(1 - \Delta_s^2), \tag{2.71}$$

where Δ is the smallest energy gap, Δ_s is the eigenvalue of $P_0 s P_0$ with smallest absolute value, and we have made use of the fact that $|\alpha| \leq \sqrt{1 - \Delta_s^2}$. In particular, SEq. (2.71) shows that a Zeeman field produces perturbatively controllable changes to projected spin eigenvalues. Notice that we have assumed in the above derivation that we are considering states at a specific momentum $\mathbf{k}$. Therefore, the above $\delta H = gs$ can come from a $\mathcal{T}$-breaking $\mathbf{k}$-independent Zeeman field interaction such as $\delta H(\mathbf{k}) = gs$, or from a $\mathcal{T}$-invariant $\mathbf{k}$-dependent SOC such as $\delta H(\mathbf{k}) = g \sin(k_x) s$. This shows that small changes to the SOC strength in a system produce parametrically small changes to the spin band structure. Physically, this means that any (topological) quantity computed from the spin band structure will be a continuous function of the SOC strength. We note that the $\mathbf{k}$-local perturbative stability of the PsP spectrum stands in contrast to the effect of perturbations on the Wilson loop (non Abelian Berry phase) spectrum. Recall (as we will review in SN 3 A) that the Wilson loop can be expressed as a product of projectors along a closed $\mathbf{k}$-path. From our perturbative series [SEq. (2.65)] for the changes to the projector, we then have that the eigenvalues of the Wilson loop depend on the perturbations to the Hamiltonian at *every* $\mathbf{k}$ along the path. This significantly complicates the study of stability of gaps in the Wilson loop spectrum.

For completeness, here we prove SEq. (2.71) which states that for a perturbation $\delta H = gs$ at a given $\mathbf{k}$ point, the first-order correction to the spectrum of $P_0 s P_0$ has a controlled upper bound. In other words, the $P_0 s P_0$ spectrum will be stable against spin-dependent perturbations to the Hamiltonian. Assuming that there is an energy gap $\Delta > 0$ between the unoccupied and occupied states such that $|E_n - E_m| \geq \Delta$ for $n \in$ unocc and $m \in$ occ, we have, from SEq. (2.70), that

$$|\lambda_1| \leq \frac{|\alpha||g|}{\Delta} \left| \sum_{\substack{n\in\text{unocc}\\ m\in\text{occ}}} \left(\langle\phi|\psi_n\rangle\langle\psi_n|s|\psi_m\rangle\langle\psi_m|\psi\rangle + \langle\psi|\psi_m\rangle\langle\psi_m|s|\psi_n\rangle\langle\psi_n|\phi\rangle \right) \right|, \tag{2.72}$$

where we have substituted $\delta H = gs$. Using the triangle inequality and the definitions of $|\psi\rangle$ and $|\phi\rangle$ which are related to each other through SEq. (2.25) and are eigenstates of $P_0 s P_0$ and $Q_0 s Q_0$ respectively, we have

$$|\lambda_1| \leq \frac{2|\alpha||g|}{\Delta} \left|\langle\phi|\, s \,|\psi\rangle\right|. \tag{2.73}$$

Using our expression in SEq. (2.25) for the action of s on $|\psi\rangle$, we yield

$$|\lambda_1| \leq 2\frac{|\alpha|^2|g|}{\Delta} = 2\frac{|g|}{\Delta} \cdot (1 - |\lambda_0|^2), \tag{2.74}$$

where we have also used $|\alpha| = \sqrt{1 - |\lambda_0|^2}$. Finally, letting $\Delta_s \geq 0$ be the absolute value of the smallest-magnitude eigenvalue of the unperturbed $P_0 s P_0$ spectrum (such that $|\lambda_0| \geq \Delta_s$), we have

$$|\lambda_1| \leq 2\frac{|g|}{\Delta}(1 - \Delta_s^2). \tag{2.75}$$

We then see that all of the perturbation strength $|g|$, energy gap Δ, and the absolute value of the smallest-magnitude unperturbed projected spin eigenvalue Δ_s contribute to the upper bound of $|\lambda_1|$. We note that Δ_s only coincides with the spin gap at a given $\mathbf{k}$ point when the system has both inversion and time-reversal symmetry. Importantly, the upper bound of $|\lambda_1|$ is linearly dependent on $|g|$, which means that one can make $|\lambda_1|$ as small as possible by decreasing $|g|$. In other words, the correction to the $P_0 s P_0$ spectrum is controllable and there is no instability where a small value of $|g|$ will induce a dramatic change of $|\lambda_1|$. In practice, this means that if a spin gap is open—*i.e.* if it is possible to divide the spin bands into two disconnected sets—then the spin gap cannot close under infinitesimally small perturbations to the Hamiltonian. In conclusion, the spin gap is stable against perturbations.

Before moving on, let us note that we can reformulate our perturbation theory for PsP in an illuminating way. In particular, we see that corrections to the projected spin eigenvalues emerge from perturbative corrections to the projection operator. An alternative way of developing the perturbative expansion of the projection operator is in terms of the Schrieffer-Wolff transformation [134–136]. Typically, the Schrieffer-Wolff transformation is used to find a canonical transformation S that block diagonalizes the Hamiltonian, *i.e.* such that

$$e^S(H_0 + \delta H)e^{-S} = P_0 H_{eff} P_0 + Q_0 H_{eff} Q_0. \tag{2.76}$$

The transformation S can be found perturbatively by expanding the left hand side of SEq. (2.76), and recursively eliminating off-diagonal terms. As a consequence of our definition in SEq. (2.60) for the projection operator, we also have that

$$e^{-S}P_0e^S = P. \tag{2.77}$$

Expanding both sides of SEq. (2.77), we recover our perturbative expansion of the projection operator. In particular, we can rewrite SEq. (2.67) as

$$P_1 = [P_0, S_1], \tag{2.78}$$

where

$$S_1 = -\sum_{nm} \frac{|\psi_n\rangle\langle\psi_n|Q_0\delta H P_0 + P_0\delta H Q_0|\psi_m\rangle\langle\psi_m|}{E_m - E_n} \tag{2.79}$$

is the standard form of the leading order term in the Schrieffer-Wolff transformation [135].

While the Schrieffer-Wolff computation of corrections to P is equivalent to our perturbative expansion in SEq. (2.65), it allows us to recast the computation of the perturbed spin spectrum in terms of computing modifications to PsP itself. In particular, so long as we are concerned only with finding the eigenvalues of PsP, we can note that

$$PsP = e^{-S}P_0e^Sse^{-S}P_0e^S, \tag{2.80}$$

which implies that PsP is isospectral to the operator $P_0e^Sse^{-S}P_0$. This allows us to compute corrections to the PsP spectrum without computing corrections to the energy eigenstates directly. The Schrieffer-Wolff approach is particularly well suited to analyzing perturbations to spin-conserving models that do not modify the energy spectrum, such as the changes to the spin quantization axis considered in SRef. [1] to demonstrate that the spin Chern number is a $\mathbb{Z}_2$ invariant (we will review this argument in detail in SN 3 C). Here we describe two examples upon which the above reformulation based on Schrieffer-Wolff transformation can be useful. Recall that the spin quantization axis of a system can be fixed by several effects. For example, a strong external Zeeman field can (nearly) fix the spin quantization axis to be aligned with the field. If the Zeeman field undergoes a slow precession, the spin quantization axis will also rotate. In addition, the spin quantization axis can also be fixed due to crystalline anisotropy. For example, recent experiments on WTe_2 have identified a fixed spin quantization axis parallel to a high-symmetry crystallographic direction lying in a mirror plane [137–139]. Therefore, for such systems, a structural distortion can change the spin quantization axis. Finally, we note that there exists a diagrammatic method [136] for computing S to arbitrary orders in perturbation theory, both for single-particle and many-body Hamiltonians. These diagrammatic methods can be leveraged for the analysis of PsP, though we leave this as a task for future work.

D. A Physical Interpretation of the Spin Gap

In this section, we provide a physical interpretation of the spin gap for systems with $\mathcal{IT}$ symmetry. Let us consider a situation where we have an insulating, inversion and time-reversal invariant system. At $t = 0$ we perform a quench by turning on a weak Zeeman field, $\delta H = B\hat{\mathbf{n}}\cdot\mathbf{s}\delta(t)$. This will create some density of excited states, and this density can be measured either through angle-resolved photoemission spectroscopy (ARPES), or by allowing the system to relax back to the ground state $|\Psi_0\rangle$ and collecting the photons and phonons that are emitted in the process. The density of excited states will be proportional to the transition rate out of the ground state, which is given to lowest order in perturbation theory by

$$\Gamma_{ex} = \sum_{n>0} B^2|\langle\Psi_n|s|\Psi_0\rangle|^2, \tag{2.81}$$

where $|\Psi_n\rangle$ are the many-body eigenstates of the system. For a non-interacting system with discrete translation symmetry whose eigenstate $|\Psi_n\rangle$ can be written as a Slater determinant of single-particle states, we can rewrite SEq. (2.81) as

$$\Gamma_{ex} = B^2\sum_{\mathbf{k}} \mathrm{Tr}\left(P(\mathbf{k})sQ(\mathbf{k})sP(\mathbf{k})\right), \tag{2.82}$$

where the summation of $\mathbf{k}$ is within the BZ. Note that the trace in SEq. (2.82) is bounded by the maximum and minimum values of $|\alpha|^2 = 1 - |\lambda|^2$, defined in SEq. (2.23). Dividing by volume to obtain an intensive quantity, we have

$$1 - \lambda_{max}^2 \le \frac{1}{B^2 N_e}\Gamma_{ex} \le 1 - \lambda_{min}^2, \tag{2.83}$$

where λ_{max} and λ_{min} are respectively the largest and smallest absolute values of all the $[s_{\text{reduced}}]$ [as defined in SEq. (2.35)] eigenvalues in the BZ, and N_e is the number of electrons in the system. We note that when obtaining λ_{max} and λ_{min} in SEq. (2.83) only the eigenstates of $P(\mathbf{k})sP(\mathbf{k})$ that are in the image of $P(\mathbf{k})$ are considered. Although there may be technical challenges in disentangling the spin Zeeman interaction used here from an orbital Zeeman interaction (which could lead to the formation of Landau levels), we still see that the spin gap $\Delta_s = \lambda_{min}$ controls the upper bound on the creation of excited states. This shows that the spin gap—and more generally the smallest-magnitude projected spin eigenvalue—place bounds on experimental observables, and are thus measurable in principle.

E. Explicit Calculations for a 3D Topological Insulator Model

In this section, we will compute the spectrum of $P(\mathbf{k})sP(\mathbf{k})$ for the Bernevig-Hughes-Zhang (BHZ) model of a 3D TI [79, 140–142]. We will show analytically that the spin gap generically closes at isolated degeneracies with a linear dispersion reminiscent of Weyl fermions. In SN 3 we will show that these "spin-Weyl nodes" are a general feature in the PsP spectrum of 3D topological insulators.

Let us start with the Bernevig-Hughes-Zhang (BHZ) model in three dimensions,

$$H_0(\mathbf{k}) = \tau_x \sum_i \sigma_i \sin k_i + (3 - m - \sum_i \cos k_i)\tau_z, \tag{2.84}$$

where τ_i are Pauli matrices acting on the orbital degrees of freedom. This model has $\mathcal{I}$ symmetry, $\mathcal{T}$ symmetry, and octahedral symmetries generated by fourfold rotations C_{4i} about the three Cartesian axes and a threefold rotation C_{31} about the $\hat{\mathbf{x}} + \hat{\mathbf{y}} + \hat{\mathbf{z}}$ cubic diagonal. These symmetries are represented by

$$[\mathcal{I}] = \tau_x, \tag{2.85}$$

$$[\mathcal{T}] = i\sigma_y \mathcal{K}, \tag{2.86}$$

$$[C_{4i}] = \exp\left(-i\frac{\pi}{4}\sigma_i\right), \tag{2.87}$$

$$[C_{31}] = \exp\left[-i\frac{\pi}{3\sqrt{3}}(\sigma_x + \sigma_y + \sigma_z)\right], \tag{2.88}$$

where $\mathcal{K}$ is the complex conjugation operation. The Hamiltonian $H_0(\mathbf{k})$ satisfies

$$[\mathcal{I}]H_0(\mathbf{k})[\mathcal{I}]^{-1} = H_0(-\mathbf{k}), \tag{2.89}$$

$$[\mathcal{T}]H_0(\mathbf{k})[\mathcal{T}]^{-1} = H_0(-\mathbf{k}), \tag{2.90}$$

$$[C_{4i}]H_0(\mathbf{k})[C_{4i}]^{-1} = H_0(C_{4i}\mathbf{k}), \tag{2.91}$$

$$[C_{31}]H_0(\mathbf{k})[C_{31}]^{-1} = H_0(C_{31}\mathbf{k}) \tag{2.92}$$

where $C_{4i}\mathbf{k}$ denotes the vector $\mathbf{k}$ rotated by $\pi/4$ radians about k_i, and $C_{31}(k_x, k_y, k_z) = (k_y, k_z, k_x)$. For $0 < m < 2$ this model represents a strong topological insulator, while for $m < 0$ it represents a trivial insulator.

Because H_0 is written in Dirac form [SEq. (2.47)] [79], we can directly compute the non-unit spectrum of PsP via SEq. (2.50). We find

$$PsP - QsQ = -\frac{1}{\epsilon(\mathbf{k})}\left(\sum_i \tau_x \hat{n}_i \sin k_i + (3 - m - \sum_i \cos k_i)\hat{\mathbf{n}} \cdot \boldsymbol{\sigma}\tau_z\right), \tag{2.93}$$

where we have introduced the band energy

$$\epsilon(\mathbf{k}) = \sqrt{\sum_i \sin^2 k_i + (3 - m - \sum_i \cos k_i)^2}. \tag{2.94}$$

We would like to identify points at which the spin gap closes. Because SEq. (2.93) determining the spectrum of PsP consists of anticommuting matrices, we see that the spin gap closes when the following two conditions are simultaneously satisfied:

$$\sum_i \hat{n}_i \sin k_i = 0, \tag{2.95}$$

$$(3 - m - \sum_i \cos k_i) = 0. \tag{2.96}$$

Note that in the topologically trivial phase, $m < 0$ and so the second equation is never satisfied. Thus, in the topologically trivial phase the BHZ model has a spin gap for all $\mathbf{k}$. In the topologically nontrivial phase, we see that the first constraint [SEq. (2.95)] defines an open surface containing origin and extending to the boundaries of the Brillouin zone, while the second constraint [SEq. (2.96)] defines a closed surface surrounding the origin. These surfaces will intersect on a closed one-dimensional curve, along which the spin gap closes. For sufficiently small m, we can expand the constraint equations to lowest order in k to find that the spin gap closes on a circle of radius $\sqrt{2m}$ centered at the origin and oriented normal to the vector $\hat{\mathbf{n}}$. As we can see from SEq. (2.93), the null eigenvectors of $PsP - QsQ$ are fourfold degenerate, implying that the null eigenvectors of PsP are twofold degenerate along this curve.

Note, however, that these twofold degenerate zeros of PsP are not perturbatively stable; the spin gap closes along a closed curve due to the residual symmetries of the model. To break these symmetries while preserving inversion and time-reversal symmetry, we can add to H_0 the perturbation

$$H_1 = \eta \sum_i \hat{m}_i \sin k_i \tau_y, \tag{2.97}$$

where $\hat{\mathbf{m}}$ is a unit vector such that $\hat{\mathbf{m}} \times \hat{\mathbf{n}} \neq 0$. The combined Hamiltonian $H_0 + H_1$ is still of Dirac form, but with a modified band energy

$$\tilde{\epsilon}(\mathbf{k}) = \sqrt{\sum_i \sin^2 k_i + \eta^2 (\sum_i \hat{m}_i \sin k_i)^2 + (3 - m - \sum_i \cos k_i)^2}. \tag{2.98}$$

Recomputing $PsP - QsQ = -1/(2\tilde{\epsilon})\{s, H_0 + H_1\}$, we find

$$\begin{aligned} PsP - QsQ = & -\frac{1}{\tilde{\epsilon}(\mathbf{k})} \sum_i \tau_x \hat{n}_i \sin k_i \\ & - \frac{\eta}{\tilde{\epsilon}(\mathbf{k})} \sum_i \tau_y (\hat{\mathbf{n}} \cdot \boldsymbol{\sigma}) \hat{m}_i \sin k_i \\ & - \frac{1}{\tilde{\epsilon}(\mathbf{k})} (3 - m - \sum_i \cos k_i) \hat{\mathbf{n}} \cdot \boldsymbol{\sigma} \tau_z . \end{aligned} \tag{2.99}$$

Since this is still given as the sum of three anticommuting terms, we see that the spin gap closes only when each of the three terms is individually equal to zero. In addition to the constraints in SEqs. (2.95) and (2.96), we have the additional constraint that

$$\sum_i \hat{m}_i \sin k_i = 0. \tag{2.100}$$

This defines a third surface in the Brillouin zone, which is open and contains the origin. Furthermore, the restriction $\hat{\mathbf{n}} \times \hat{\mathbf{m}} \neq 0$ ensures that this surface is independent from the one defined in SEq. (2.95). These three surfaces will generically intersect at a set of isolated points. For small m, we can again expand our constraint equations to find that the spin gap closes at two points, which lie at the intersection of a sphere of radius $\sqrt{2m}$, and the line through the origin parallel to $\hat{\mathbf{n}} \times \hat{\mathbf{m}}$. These are perturbatively stable fourfold degeneracies in the spectrum of $PsP - QsQ$, and hence correspond to twofold-degenerate Weyl nodes in the spectrum of PsP. Just like Weyl nodes in the energy spectrum of a Hamiltonian, these "spin-Weyl nodes" are stable to perturbations of the Hamiltonian (by virtue of our results in SN 2 C establishing that the PsP spectrum varies continuously when the Hamiltonian is perturbed).

The fact that PsP has at least one Weyl node in each half of the BZ for the $\mathcal{I}$- and $\mathcal{T}$-symmetric 3D TI can also be established on topological grounds. Consider two parallel $\mathcal{T}$-invariant planes in the BZ, defined as planes with constant $k_i = 0$ and $k_i = \pi$ for one choice of $i = 1, 2, 3$. Furthermore, assume that PsP is gapped on both

planes. Then we can compute the partial Chern numbers $C^-(k_i=0)$ and $C^-(k_i=\pi)$ of the projector $P_-(\mathbf{k})$ onto the states with negative PsP eigenvalue in each of these planes. As shown in SRefs. [1, 7] (and as we will review in SN 3), $C^-(k_i=0,\pi) \mod 2$ gives the value of the Kane-Mele invariant $\nu_{2d}(k_i=0,\pi)$ in each plane. Since our entire system is a 3D TI, we must have that $|\nu_{2d}(k_i=0)-\nu_{2d}(k_i=\pi)|=1$. This implies that the partial Chern numbers $C^-(k_i=0)$ and $C^-(k_i=\pi)$ must differ by an odd integer. Since we can define the partial Chern numbers $C^-(k_i)$ for any constant-k_i plane in the 3D BZ provided that the spin gap in the 2D plane is opened, and since $C^-(k_i)$ is an integer, this means that for some non-$\mathcal{T}$-invariant plane the partial Chern number must cease to be well-defined, which can only happen when the spin gap closes at a spin-Weyl point. Furthermore, the integer change in partial Chern number of the $N_{\text{occ}}/2$ lower spin bands corresponds to the chiral charge of the spin-Weyl point. We will later demonstrate this numerically in SN 3 E for a 3D TI with inversion symmetry, and in SN 3 F for a 3D TI without inversion symmetry, using our spin-resolved Wilson loop formalism.

F. Effects of Spin-Orbital Entanglement on the Spin Spectrum

In elucidating the properties of the projected spin operator PsP developed in SN 2 B, we did not need to make a specific choice for the spin direction $\hat{\mathbf{n}}$ in $s=\hat{\mathbf{n}}\cdot\mathbf{s}$. We can then ask the following question: for a system with a spin gap for a given $\hat{\mathbf{n}}$, must there be a spin gap for other spin directions? In this section we will answer this question in the negative by giving explicit examples. We will see shortly that although the spin operator s acts as the identity in the orbital subspace, the *orbital components* of the occupied wave functions are also important when determining the PsP spectrum. In particular, entanglement between the spin and orbital degrees of freedom in the wave function can force the spin gap to close for certain choices of $\hat{\mathbf{n}}$. Let us consider the following two 4×4 Hamiltonians

$$H_1 = \tau_z\sigma_0, \tag{2.101}$$
$$H_2 = \tau_y\sigma_z, \tag{2.102}$$

where the Pauli matrices τ_μ and σ_ν act on orbital and spin degrees of freedom, respectively. τ_0 and σ_0 are both 2×2 identity matrices. Both H_1 and H_2 have energies $(-1,-1,+1,+1)$, and both are invariant under spinful $\mathcal{T}$ symmetry represented by

$$[\mathcal{T}]H_1[\mathcal{T}]^{-1} = \sigma_y H_1^*\sigma_y = H_1, \tag{2.103}$$
$$[\mathcal{T}]H_2[\mathcal{T}]^{-1} = \sigma_y H_2^*\sigma_y = H_2. \tag{2.104}$$

We now compute the reduced spin matrix defined in SEq. (2.35) for both H_1 and H_2. We take for our occupied states the two states with negative energy in both cases. To be explicit, the two states with energy eigenvalues -1 are given by

$$|1\rangle = \begin{bmatrix}0\\1\end{bmatrix}\otimes\begin{bmatrix}1\\0\end{bmatrix},\ |2\rangle = \begin{bmatrix}0\\1\end{bmatrix}\otimes\begin{bmatrix}0\\1\end{bmatrix} \text{ for } H_1, \tag{2.105}$$
$$|1\rangle = \frac{1}{\sqrt{2}}\begin{bmatrix}1\\-i\end{bmatrix}\otimes\begin{bmatrix}1\\0\end{bmatrix},\ |2\rangle = \frac{1}{\sqrt{2}}\begin{bmatrix}1\\i\end{bmatrix}\otimes\begin{bmatrix}0\\1\end{bmatrix} \text{ for } H_2. \tag{2.106}$$

The first vector in each tensor product corresponds to the orbital (τ) degree of freedom, and the second vector in each tensor product corresponds to the spin (σ) degree of freedom. Notice that the negative energy eigenstates of H_1 and H_2 in SEqs. (2.105) and (2.106) are all s_z eigenstates. In addition, the *orbital components* of the eigenstates of H_1 in SEq. (2.105) are *identical.* Because of this, by taking linear combinations of $|1\rangle$ and $|2\rangle$ in SEq. (2.105), the occupied eigenvectors for H_1 can be chosen as eigenstates of $\hat{\mathbf{n}}\cdot\mathbf{s}$ for any choice of $\hat{\mathbf{n}}$. On the other hand, the orbital components of the eigenstates of H_2 in SEq. (2.106) are orthogonal. This means that for the eigenstates of H_2 we cannot create $\hat{\mathbf{n}}\cdot\mathbf{s}$ eigenstates for arbitrary $\hat{\mathbf{n}}$ by taking linear combinations of $|1\rangle$ and $|2\rangle$ in SEq. (2.106).

This difference in the orbital part of the eigenfunctions is crucial in determining the spin spectrum. To see this, recall that the spin operator s is defined as $s\equiv\tau_0\sigma$, where $\sigma=\boldsymbol{\sigma}\cdot\hat{\mathbf{n}}$ and $\hat{\mathbf{n}}$ is a unit vector in 3D [see SEq. (2.13)]. The reduced spin matrix from SEq. (2.35), in the two-dimensional space of states with -1 energy eigenvalues, is then given by

$$[s_{\text{reduced}}]_{2\times2} = \begin{bmatrix}\langle1|s|1\rangle & \langle1|s|2\rangle\\ \langle2|s|1\rangle & \langle2|s|2\rangle\end{bmatrix} = \begin{bmatrix}\langle1|\tau_0\sigma|1\rangle & \langle1|\tau_0\sigma|2\rangle\\ \langle2|\tau_0\sigma|1\rangle & \langle2|\tau_0\sigma|2\rangle\end{bmatrix} = \begin{bmatrix}\langle1_\tau|\tau_0|1_\tau\rangle\langle1_\sigma|\sigma|1_\sigma\rangle & \langle1_\tau|\tau_0|2_\tau\rangle\langle1_\sigma|\sigma|2_\sigma\rangle\\ \langle2_\tau|\tau_0|1_\tau\rangle\langle2_\sigma|\sigma|1_\sigma\rangle & \langle2_\tau|\tau_0|2_\tau\rangle\langle2_\sigma|\sigma|2_\sigma\rangle\end{bmatrix} \tag{2.107}$$

where $|i_\tau\rangle$ and $|i_\sigma\rangle$ are the orbital and spin components of the ith eigenstate with energy eigenvalue -1 from

SEqs. (2.105) or (2.106) such that $|i\rangle = |i_\tau\rangle \otimes |i_\sigma\rangle$. Using SEq. (2.107), we find the following reduced spin matrices

$$[s]_{2\times 2} = \begin{bmatrix} \sigma_{11} & \sigma_{12} \\ \sigma_{21} & \sigma_{22} \end{bmatrix} = \sigma \text{ for } H_1, \tag{2.108}$$

$$[s]_{2\times 2} = \begin{bmatrix} \sigma_{11} & 0 \\ 0 & \sigma_{22} \end{bmatrix} \text{ for } H_2, \tag{2.109}$$

where σ_{ij} are the matrix elements of the 2×2 matrix $\sigma = \boldsymbol{\sigma} \cdot \hat{\mathbf{n}}$. As we can see in SEq. (2.109), the reduced spin matrix for H_2 is diagonal, which follows from the fact that the *orbital components* of $|1\rangle$ and $|2\rangle$ in SEq. (2.106) are orthogonal for the occupied eigenstates of H_2. We can then compute the two eigenvalues of the reduced spin matrices with $\hat{\mathbf{n}} = \hat{\mathbf{x}}$, $\hat{\mathbf{y}}$, and $\hat{\mathbf{z}}$; the results are summarized in Supplementary Table 1. We see that the spin gap is open for all of $\hat{\mathbf{n}} = \hat{\mathbf{x}}$, $\hat{\mathbf{y}}$, and $\hat{\mathbf{z}}$ for the eigenvectors in SEq. (2.105) of H_1. In fact, since the reduced spin matrix for H_1 is $\sigma = \boldsymbol{\sigma} \cdot \hat{\mathbf{n}}$, as shown in SEq. (2.108), no matter what direction $\hat{\mathbf{n}}$ we choose, the occupied subspace of H_1 has a nonzero spin gap. However, in the case of H_2, we find that although the spin gap is open for $\hat{\mathbf{n}} = \hat{\mathbf{z}}$, the spin gap is closed for $\hat{\mathbf{n}} = \hat{\mathbf{x}}$ and $\hat{\mathbf{y}}$. This is because when $\hat{\mathbf{n}} = \hat{\mathbf{x}}$ and $\hat{\mathbf{y}}$, the reduced spin matrices in SEq. (2.109) are zero matrices, and thus the two eigenvalues of the reduced spin operator are degenerate and equal to 0. From the above example, we have demonstrated that although the spin gap in the occupied space is open for one choice of $\hat{\mathbf{n}}$, it is not guaranteed that the spin gap will be open for other choices of $\hat{\mathbf{n}}$. In particular we have seen that entanglement between spin and orbital degrees of freedom plays a crucial role in determining the spin gap.

	H_1	H_2
$s = \tau_0\sigma_x$	$(-1, +1)$	$(0, 0)$
$s = \tau_0\sigma_y$	$(-1, +1)$	$(0, 0)$
$s = \tau_0\sigma_z$	$(-1, +1)$	$(-1, +1)$

Supplementary Table 1. The two eigenvalues of the reduced spin matrices in SEqs. (2.108)–(2.109) with $\hat{\mathbf{n}} = \hat{\mathbf{x}}$, $\hat{\mathbf{y}}$, and $\hat{\mathbf{z}}$ in the occupied (negative energy) subspace of eigenstates of H_1 and H_2.

We thus see that whether or not there is a spin gap for a particular choice of direction $\hat{\mathbf{n}}$ depends on the microscopic details of the spin-orbit interaction. Concretely, H_2 from SEq. (2.102) can be viewed as the spin-orbit contribution to a Bloch Hamiltonian at a time-reversal-invariant crystal momentum (TRIM). We see that the entanglement between spin and orbital degrees of freedom in the eigenstates [SEq. (2.106)] results in a preferred choice of direction $\hat{\mathbf{n}} = \hat{\mathbf{z}}$ along which the spin gap is maximal. We will revisit this discussion for more realistic spin and orbital textures in SN 9 and 10, in which we compute the spin spectrum of the candidate higher-order topological insulators (HOTIs) β-MoTe$_2$ [31, 50] and α-BiBr [50, 52, 53]. In SN 9 and 10, we will specifically respectively demonstrate that while β-MoTe$_2$ does not exhibit a spin gap for any choice of spin direction, α-BiBr exhibits a spin gap for multiple choices of $\hat{\mathbf{n}}$, including a large ($\Delta_s \sim 1$ in unit of $\hbar/2$) spin gap for $\hat{\mathbf{n}} = \hat{\mathbf{z}}$.

G. (Spin) Band Structure in a Strong Zeeman Field

In this section, we will show that the partial band topology topology of the spin band structure is intimately connected to the electronic band topology of an insulator in a strong Zeeman field. To see this, let us consider a spinful, noninteracting electron system with the Hamiltonian

$$H = H_0 - g\mu_B|\mathbf{B}|s + V_0, \tag{2.110}$$

where H_0 determines the band structure in the absence of external perturbations, $\mathbf{B}$ is the external magnetic field, g is the spin g-factor (where we take $g > 0$ for simplicity), μ_B is the Bohr magneton, $s = \hat{\mathbf{B}} \cdot \mathbf{s}$ is the spin component along the magnetic field direction, and V_0 is a scalar potential which we will use to manipulate the Fermi level. If we take

$$V_0 = g\mu_B|\mathbf{B}|, \tag{2.111}$$

then we can rewrite SEq. (2.110) as

$$H = H_0 + g\mu_B|\mathbf{B}|P_\downarrow, \tag{2.112}$$

$$\equiv H_0 + \delta H \tag{2.113}$$

where $P_\downarrow$ is the projection operator onto the negative eigenspace of s from SEq. (2.54). For this choice of V_0, the combined effects of the Zeeman and scalar potentials are then to energetically penalize spin-down electrons, while leaving spin-up electrons unaffected.

This has particularly stark consequences in the limit $g\mu_B|\mathbf{B}| \to \infty$. In this limit, we can view δH as the unperturbed Hamiltonian, and treat H_0 as the perturbation. Since δH pushes spin-down electrons up to negative energy, the projector onto the low energy subspace of δH is given by SEq. (2.53). We then have that to first order in perturbation theory

$$H \approx \delta H + P_\uparrow H_0 P_\uparrow. \tag{2.114}$$

In particular, since $\delta H P_\uparrow = 0$, energies in the low-energy $P_\uparrow$ subspace are completely determined by

$$H_{\text{low}} = P_\uparrow H_0 P_\uparrow. \tag{2.115}$$

This means that the effect of δH as $g\mu_B|\mathbf{B}| \to \infty$ is to project H_0 onto the spin-up subspace.

Let us now focus on the low-energy $P_\uparrow$ subspace. Introducing a set of eigenstates $|n\mathbf{k}\rangle$ and energies $\epsilon_{n\mathbf{k}}$ for H_0, we can re-express SEq. (2.115) as

$$H_{\text{low}} = \sum_{n\mathbf{k}} \epsilon_{n\mathbf{k}} P_\uparrow |n\mathbf{k}\rangle \langle n\mathbf{k}| P_\uparrow. \tag{2.116}$$

To make further progress, let us assume that H_0 has a gapped spectrum. By an appropriate shift of the zero of energy at each $\mathbf{k}$, we can ensure for convenience that the gap is centered at zero, such that states with $\epsilon_{n\mathbf{k}} > 0$ are above the gap (unoccupied), and states with $\epsilon_{n\mathbf{k}} < 0$ are below the gap (occupied). Before turning on a Zeeman field, we can spectrally flatten H_0 to have the form

$$H_0(\mathbf{k}) \to Q(\mathbf{k}) - P(\mathbf{k}) \tag{2.117}$$

where we have introduced the projectors $P(\mathbf{k})$ and $Q(\mathbf{k})$ onto the occupied and unoccupied space of H_0 at each $\mathbf{k}$ respectively. In this case, H_{low} becomes

$$H_{\text{low}} \to \sum_{n\in\text{unocc}} P_\uparrow |n\mathbf{k}\rangle \langle n\mathbf{k}| P_\uparrow - \sum_{n\in\text{occ}} P_\uparrow |n\mathbf{k}\rangle \langle n\mathbf{k}| P_\uparrow \tag{2.118}$$

$$= \sum_{\mathbf{k}} P_\uparrow Q(\mathbf{k}) P_\uparrow - P_\uparrow P(\mathbf{k}) P_\uparrow, \tag{2.119}$$

Note that $P_\uparrow P(\mathbf{k}) P_\uparrow$ is exactly the spin-projected correlation matrix of SRef. [131], and $P_\uparrow Q(\mathbf{k}) P_\uparrow$ is the analogous operator for the unoccupied states.

Next, we can express P and Q in terms of eigenstates of PsP and QsQ. In particular, using the results of SN 2 B, we can write

$$P(\mathbf{k}) = \sum_{|\lambda_\mathbf{k}|\neq 1} |\lambda_\mathbf{k}\rangle \langle \lambda_\mathbf{k}| + \sum_i |{+i\mathbf{k}}\rangle \langle {+i\mathbf{k}}| + |{-i\mathbf{k}}\rangle \langle {-i\mathbf{k}}|, \tag{2.120}$$

where $|\lambda_\mathbf{k}\rangle$ are the eigenstates of PsP with eigenvalue $\lambda_\mathbf{k} \neq \pm 1$, and $|\pm i\mathbf{k}\rangle$ are the eigenstates of PsP with eigenvalue ± 1. As shown in SN 2 B, the eigenstates $|\lambda_\mathbf{k}\rangle$ are in one-to-one correspondence with eigenstates

$$|\phi(\lambda_\mathbf{k})\rangle = \frac{Qs|\lambda_\mathbf{k}\rangle}{\sqrt{\langle\lambda_\mathbf{k}| sQs |\lambda_\mathbf{k}\rangle}} = \frac{Qs|\lambda_\mathbf{k}\rangle}{\sqrt{1-\lambda_k^2}} \tag{2.121}$$

of QsQ with eigenvalue $-\lambda$. This means we can also write

$$Q(\mathbf{k}) = \sum_{|\lambda_\mathbf{k}|\neq 1} |\phi(\lambda_\mathbf{k})\rangle \langle \phi(\lambda_\mathbf{k})| + \sum_{\tilde{i}} |{+\tilde{i}\mathbf{k}}\rangle \langle {+\tilde{i}\mathbf{k}}| + |{-\tilde{i}\mathbf{k}}\rangle \langle {-\tilde{i}\mathbf{k}}|, \tag{2.122}$$

where $|\pm\tilde{i}\mathbf{k}\rangle$ are the eigenstates of QsQ with eigenvalue ± 1. Using the definitions of $|\lambda_\mathbf{k}\rangle, |\phi(\lambda_\mathbf{k})\rangle, |\pm i\mathbf{k}\rangle$ and $|\pm\tilde{i}\mathbf{k}\rangle$ along with SEqs. (2.20)–(2.27) we have

$$P_\uparrow |\lambda_\mathbf{k}\rangle = \frac{1+\lambda_\mathbf{k}}{2} |\lambda_\mathbf{k}\rangle + \frac{\sqrt{1-|\lambda_\mathbf{k}|^2}}{2} |\phi(\lambda_\mathbf{k})\rangle \tag{2.123}$$

$$P_\uparrow |\phi(\lambda_\mathbf{k})\rangle = \frac{1-\lambda_\mathbf{k}}{2} |\phi(\lambda_\mathbf{k})\rangle + \frac{\sqrt{1-|\lambda_\mathbf{k}|^2}}{2} |\lambda_\mathbf{k}\rangle \tag{2.124}$$

$$P_\uparrow |{+i\mathbf{k}}\rangle = |{+i\mathbf{k}}\rangle \tag{2.125}$$

$$P_\uparrow |{+\tilde{i}\mathbf{k}}\rangle = |{+\tilde{i}\mathbf{k}}\rangle \tag{2.126}$$

$$P_\uparrow |{-i\mathbf{k}}\rangle = P_\uparrow |{-\tilde{i}\mathbf{k}}\rangle = 0. \tag{2.127}$$

We can now combine SEqs. (2.120) and (2.122) with the definition in SEq. (2.116). Using SEqs. (2.123)–(2.127) we find

$$P_\uparrow[Q(\mathbf{k})-P(\mathbf{k})]P_\uparrow = \sum_{|\lambda_\mathbf{k}|\neq 1} -\lambda_\mathbf{k}\left(\sqrt{\frac{1+\lambda_\mathbf{k}}{2}}\left|\lambda_\mathbf{k}\right\rangle + \sqrt{\frac{1-\lambda_\mathbf{k}}{2}}\left|\phi(\lambda_\mathbf{k})\right\rangle\right)\left(\sqrt{\frac{1+\lambda_\mathbf{k}}{2}}\left\langle\lambda_\mathbf{k}\right| + \sqrt{\frac{1-\lambda_\mathbf{k}}{2}}\left\langle\phi(\lambda_\mathbf{k})\right|\right)$$
$$+\sum_{\tilde{i}}\left|+\tilde{i}\mathbf{k}\right\rangle\left\langle+\tilde{i}\mathbf{k}\right| - \sum_i \left|+i\mathbf{k}\right\rangle\left\langle+i\mathbf{k}\right|. \tag{2.128}$$

Note that we can define $|\phi(+1)\rangle \equiv 0$ and $|\phi(-1_\mathbf{k})\rangle \equiv \left|+\tilde{i}\mathbf{k}\right\rangle$, and that this is consistent with SEqs. (2.20)–(2.27), which allows us to write

$$P_\uparrow[Q(\mathbf{k})-P(\mathbf{k})]P_\uparrow = \sum_{\lambda_\mathbf{k}} -\lambda_\mathbf{k}\left(\sqrt{\frac{1+\lambda_\mathbf{k}}{2}}\left|\lambda_\mathbf{k}\right\rangle + \sqrt{\frac{1-\lambda_\mathbf{k}}{2}}\left|\phi(\lambda_\mathbf{k})\right\rangle\right)\left(\sqrt{\frac{1+\lambda_\mathbf{k}}{2}}\left\langle\lambda_\mathbf{k}\right| + \sqrt{\frac{1-\lambda_\mathbf{k}}{2}}\left\langle\phi(\lambda_\mathbf{k})\right|\right) \tag{2.129}$$

$$\equiv \sum_{\lambda_\mathbf{k}} -\lambda_\mathbf{k}\left|\psi_\mathbf{k}\right\rangle\left\langle\psi_\mathbf{k}\right|. \tag{2.130}$$

Since the states $|\psi_\mathbf{k}\rangle$ are orthonormal, we see that the spectrum of $P_\uparrow[Q(\mathbf{k})-P(\mathbf{k})]P_\uparrow$ coincides with the spectrum $\{\lambda_\mathbf{k}\}$ of the reduced spin operator [SEq. (2.35)], modulo the number of $+1$ eigenvalues corresponding to exact spin-up eigenstates of Q, and also modulo the number of -1 eigenvalues corresponding to exact spin-down eigenstates of P. Furthermore, recall that the spectrum of $P(\mathbf{k})sP(\mathbf{k})$ restricted to the occupied states coincides with the spectrum of the reduced spin operator. This means that the low energy spectrum of a system with spectrally flat H_0 [SEq. (2.117)] in a strong Zeeman field coincides with the nontrivial spectrum of $P(\mathbf{k})sP(\mathbf{k})$. As a side-effect, also note that $P_\uparrow[Q(\mathbf{k})-P(\mathbf{k})]P_\uparrow = P_\uparrow[1-2P(\mathbf{k})]P_\uparrow = P_\uparrow - 2C_\uparrow$ is directly related to the spin-projected correlation matrix of SRefs. [131–133]; SEq. (2.130) hence shows then that there is a one-to-one correspondence between PsP eigenvalues and $C_\uparrow$ eigenvalues (modulo the number of states at the accumulation point $\lambda_\mathbf{k} = -1$).

We can go further to relate the topology of PsP bands and $C_\uparrow$ bands if there is a spin gap. In this case, we can spectrally flatten SEq. (2.129) by spectrally flattening $P(\mathbf{k})sP(\mathbf{k})$, taking $\lambda_\mathbf{k}\to -1$ continuously for the bands below the spin gap and $\lambda_\mathbf{k}\to +1$ continuously for states above the spin gap, while leaving the eigenstates $|\lambda_\mathbf{k}\rangle$ and $|\phi(\lambda_\mathbf{k})\rangle$ unchanged. The states $|\psi_\mathbf{k}\rangle$ change continuously under this deformation, yielding

$$P_\uparrow[Q(\mathbf{k})-P(\mathbf{k})]P_\uparrow \sim \sum_{\lambda_\mathbf{k}}^{\text{below}}\left|\phi(\lambda_\mathbf{k})\right\rangle\left\langle\phi(\lambda_\mathbf{k})\right| - \sum_{\lambda_\mathbf{k}}^{\text{above}}\left|\lambda_\mathbf{k}\right\rangle\left\langle\lambda_\mathbf{k}\right|, \tag{2.131}$$

where $\sum_{\lambda_\mathbf{k}}^{\text{above}}$ is a sum is over $\lambda_\mathbf{k}$ in the upper spin bands, and $\sum_{\lambda_\mathbf{k}}^{\text{below}}$ is a sum over $\lambda_\mathbf{k}$ in the lower spin bands. Finally, we see that

$$\sum_{\lambda_\mathbf{k}}^{\text{above}}\left|\lambda_\mathbf{k}\right\rangle\left\langle\lambda_\mathbf{k}\right| = P_+, \tag{2.132}$$

$$\sum_{\lambda_\mathbf{k}}^{\text{below}}\left|\phi(\lambda_\mathbf{k})\right\rangle\left\langle\phi(\lambda_\mathbf{k})\right| = Q_+, \tag{2.133}$$

where P_+ is the projector onto the upper spin bands in the occupied subspace, and Q_+ is the projector onto the upper spin bands in the unoccupied subspace. This means that for systems with a spin gap, the topological properties of $P_\uparrow[Q(\mathbf{k})-P(\mathbf{k})]P_\uparrow$ bands coincide with the topological properties of the spin band structure.

Finally, we would like to determine the extent to which we can relate the topological properties of $P_\uparrow[Q(\mathbf{k})-P(\mathbf{k})]P_\uparrow$ bands to the topological properties of H_{low} directly. In general, we cannot do this: the process of projecting H into the $P_\uparrow$ subspace does not commute with the spectral flattening process—gaps in the spectrum can close in H_{low} as we spectrally flatten H. However, for inversion symmetric systems, the theory of symmetry indicators can provide more information. In particular, as long as inversion eigenvalues of the occupied bands are not exchanged with inversion eigenvalues of the unoccupied bands at TRIM points as we deform $H_{\text{low}}\to P_\uparrow[Q(\mathbf{k})-P(\mathbf{k})]P_\uparrow$, then we know that the symmetry-indicated topology of H_{low} and $P_\uparrow[Q(\mathbf{k})-P(\mathbf{k})]P_\uparrow$ will coincide. We expect that for any inversion-symmetric topological system derived from a band-inverted semiconductor with weak spin orbit coupling, there will be a spin gap for some spin direction at all TRIM points. In these cases, applying a Zeeman field along or near that

given spin direction will not invert the lowest spin band inversion eigenvalues, and hence will allow us to deduce the H_{low} band topology from the PsP band topology. Note, furthermore, that if H_0 is a $\mathcal{T}$-invariant insulator, then both H_{low} and PsP respect the symmetries of a magnetic space group. Since the symmetry indicators of the magnetic space groups are fully enumerated [43–46, 143], we can extend this logic to say that the magnetic symmetry indicated topology of H_{low} and PsP will coincide.

As an example, we can apply this logic to 3D strong topological insulators. As discussed in SN 2 E, the PsP spectrum for a 3D TI has an odd number of spin-Weyl points per half BZ. This implies that under the influence of a strong Zeeman field, the low energy Hamiltonian H_{low} will have an odd number of Weyl points per half BZ in its spectrum. This means that for a semi-infinite slab of an inversion-symmetric 3D TI under the influence of a strong Zeeman field, we expect to find Fermi arcs connecting the surface projections of these Weyl points. Although we can only rigorously establish the existence of Weyl points in H_{low} for inversion-symmetric systems with a spin gap, we expect that these results will hold even without inversion symmetry, due to the topological stability of Weyl points (*i.e.*, the improbability of Weyl points annihilating as we adiabatically deform the spectrum of H_{low} to that of PsP). We will show that this intuition is justified in SN 9 by considering the candidate higher-order topological insulator β-$MoTe_2$ [31, 50] in the presence of a strong Zeeman field. We will specifically show that β-$MoTe_2$ is a spin-Weyl semimetal for all choices of spin direction, with detectable Fermi arcs on the (001) surface in the presence of a strong Zeeman field. Furthermore, we also show that α-BiBr is a spin-Weyl semimetal for a neighborhood of spin directions surrounding s_y in SN 10.

3. SPIN-RESOLVED WILSON LOOPS

In this section, we will show how the projected spin operator can be used to formulate a refined notion of band topology. We will start in SN 3 A by reviewing the definition of the Wilson loop (non-Abelian Berry phase), and its application to computing topological invariants of bands in the image of a projector $P(\mathbf{k})$. Next, in SN 3 B we will show that for systems with a spin gap, we can extend the definition of the Wilson loop to compute Berry phases for PsP eigenstates, which we term spin-resolved Wilson loop. This will allow us to introduce spin-resolved topological invariants, which classify bands that cannot be deformed into each other without closing *either* an energy gap or a spin gap. We then study some generic properties of the spin-resolved Wilson loops, and relate their spectral flows to the 2D Kane-Mele $\mathbb{Z}_2$ invariant and the topological contribution to the spin Hall conductivity, in SN 3 C. We will then present examples of spin-resolved topology in two (SN 3 D) and three (SN 3 E and 3 F) dimensions. We will explore the connection between spin-resolved and fragile topology in SN 3 G. Finally, in SN 3 H we will generalize the correspondence between Wilson loops and the entanglement spectrum to spin-resolved Wilson loops.

A. P-Wilson Loop

We denote $[P(\mathbf{k})]$ as the matrix projector to a set of N_{occ} occupied states of the Bloch Hamiltonian matrix $[H(\mathbf{k})]$, namely

$$[P(\mathbf{k})] = \sum_{n=1}^{N_{\text{occ}}} |u_{n,\mathbf{k}}\rangle \langle u_{n,\mathbf{k}}| , \tag{3.1}$$

where $|u_{n,\mathbf{k}}\rangle$ is defined in SEq. (2.11), and n denotes the band index. Notice that we can choose any set of occupied bands as long as there is a finite gap between the energy $E_{n,\mathbf{k}}$ of the occupied bands from the others for all $\mathbf{k}$. We work in a truncated tight-binding Hilbert space with $N_{\text{sta}} = 2N_{\text{orb}}$ states per unit cell, where 2 accounts for the spin degrees of freedom and N_{orb} is the number of orbitals (for example s and p orbitals). Such a truncation can always be obtained for a real material through, *e.g.* Wannierization of a topologically trivial subset of electronic states in an ab-initio calculation [144]. We denote by $[P(\mathbf{k})]$ the $2N_{\text{orb}} \times 2N_{\text{orb}}$ matrix representation of $P(\mathbf{k})$. Similarly, our Bloch eigenstates $|u_{n,\mathbf{k}}\rangle$ are $2N_{\text{orb}}$-component vectors in the tight-binding basis states. The P-Wilson loop matrix for the holonomy starting at base point $\mathbf{k}$ and going along a straight path to $\mathbf{k}+\mathbf{G}$ where $\mathbf{G}$ is a primitive reciprocal lattice vector is given by [3, 4, 145]

$$\begin{aligned}[\mathcal{W}_{1,\mathbf{k},\mathbf{G}}]_{m,n} &= \langle u_{m,\mathbf{k}+\mathbf{G}}| \left(\prod_{\mathbf{q}}^{\mathbf{k}+\mathbf{G}\leftarrow\mathbf{k}} [P(\mathbf{q})] \right) |u_{n,\mathbf{k}}\rangle \\ &= \langle u_{m,\mathbf{k}}| \, [V(\mathbf{G})] \left(\prod_{\mathbf{q}}^{\mathbf{k}+\mathbf{G}\leftarrow\mathbf{k}} [P(\mathbf{q})] \right) |u_{n,\mathbf{k}}\rangle , \end{aligned} \tag{3.2}$$

where both m and n are the indices of occupied eigenvectors, the $[V(\mathbf{G})]$ matrix is defined in SN 2 A, and

$$\left(\prod_{\mathbf{q}}^{\mathbf{k}+\mathbf{G}\leftarrow\mathbf{k}}[P(\mathbf{q})]\right) \tag{3.3}$$

$$\equiv \lim_{N\to\infty}[P(\mathbf{k}+\mathbf{G})][P(\mathbf{k}+\frac{N-1}{N}\mathbf{G})]\ldots[P(\mathbf{k}+\frac{1}{N}\mathbf{G})][P(\mathbf{k})]. \tag{3.4}$$

The eigenvalues of the P-Wilson loop matrix $[\mathcal{W}_{1,\mathbf{k},\mathbf{G}}]$ [SEq. (3.2)], which is an $N_{\text{occ}}\times N_{\text{occ}}$ matrix, are of the unimodular form $\exp\{i(\gamma_1)_{j,\mathbf{k},\mathbf{G}}\}$ where $j=1\ldots N_{\text{occ}}$ index the N_{occ} eigenvalues. We call the set $\{(\gamma_1)_{j,\mathbf{k},\mathbf{G}}\}$ as *P-Wannier bands* (or simply *Wannier bands*) because $\{(\gamma_1)_{j,\mathbf{k},\mathbf{G}}\}$ tells us the positions of the hybrid Wannier functions, which are localized along the lattice vector $\mathbf{a}$ dual to $\mathbf{G}$; to be specific the hybrid Wannier functions are localized at $\mathbf{a}\cdot(\gamma_1)_{j,\mathbf{k},\mathbf{G}}/2\pi$. The P-Wilson loop eigenphases $\{(\gamma_1)_{j,\mathbf{k},\mathbf{G}}\}$ are the Berry phases of the N_{occ} occupied states for the closed loop in $\mathbf{k}$-space parallel to $\mathbf{G}$. If we write the base point $\mathbf{k}=\sum_{j=1}^{d}\frac{k_j}{2\pi}\mathbf{G}_j$ where d is the spatial dimension of the system, and if we choose the closed loop holonomy to be along $\mathbf{G}_i$ with base point $\mathbf{k}$, then $\{(\gamma_1)_{j,\mathbf{k},\mathbf{G}_i}\}$ will be independent of the component $k_i=\mathbf{k}\cdot\mathbf{a}_i$ where $\{\mathbf{a}_i\}$ is the set of position-space lattice vectors dual to the reciprocal lattice vectors $\{\mathbf{G}_j\}$ such that $\mathbf{a}_i\cdot\mathbf{G}_j=2\pi\delta_{ij}$ [146]. In addition, to directly specify the holonomy in SEq. (3.2), we will sometimes call $\{(\gamma_1)_{j,\mathbf{k},\mathbf{G}}\}$ as a function of $\mathbf{k}$ the $\widehat{\mathbf{G}}$*-directed P-Wannier bands* (or simply $\widehat{\mathbf{G}}$*-directed Wannier bands*), where j labels the band index. The symmetry-protected spectral flow of the eigenphases $\{(\gamma_1)_{j,\mathbf{k},\mathbf{G}}\}$ is a powerful tool for diagnosing the nontrivial topology of energy bands [3–5, 76, 107, 145, 147–153]. In SN 6 A and 6 C we prove the constraints on the values of the P-Wilson loop eigenphases at different $\mathbf{k}$ points due to inversion and time-reversal symmetries, respectively.

B. $P_{\pm}$-Wilson Loop

We would now like to formulate a definition of a Wilson loop applicable to spin bands. Recall from SEq. (2.35) that we can introduce the reduced spin matrix

$$[s_{\text{reduced}}(\mathbf{k})]_{m,n}=\langle u_{m,\mathbf{k}}|\,s\,|u_{n,\mathbf{k}}\rangle=\langle u_{m,\mathbf{k}}|\,\hat{\mathbf{n}}\cdot\mathbf{s}\,|u_{n,\mathbf{k}}\rangle \tag{3.5}$$

where $\hat{\mathbf{n}}$ is a unit vector, $\mathbf{s}$ is the spin operator from SEq. (2.13), m and n index the occupied states (*i.e.* states in the image of occupied-space projector $[P(\mathbf{k})]$), and where we have made the $\mathbf{k}$ dependence explicit. Suppose that a spin gap is open (see SN 2 B), such that we can divide the eigenvalues of $[s_{\text{reduced}}(\mathbf{k})]$ into two disjoint groups labeled by $\pm$,

$$[s_{\text{reduced}}(\mathbf{k})]\left|\tilde{u}^{\pm}_{n,\mathbf{k}}\right\rangle=\lambda^{\pm}_{n,\mathbf{k}}\left|\tilde{u}^{\pm}_{n,\mathbf{k}}\right\rangle, \tag{3.6}$$

where we have for all $\mathbf{k}$, n and m that $\lambda^{+}_{n,\mathbf{k}}>\lambda^{-}_{m,\mathbf{k}}$. We refer to $\{\lambda^{\pm}_{n,\mathbf{k}}\}$ as the upper and lower PsP bands, respectively. We denote by N^{+}_{occ} the number of upper spin bands, and by N^{-}_{occ} the number of lower spin bands. We necessarily have $N^{+}_{\text{occ}}+N^{-}_{\text{occ}}=N_{\text{occ}}$. Furthermore, since a spin gap is open, N^{+}_{occ} and N^{-}_{occ} are independent of $\mathbf{k}$. Recall from SN 2 B that in a system with $\mathcal{T}$ symmetry, it is possible to choose the upper and lower spin bands such that $N^{+}_{\text{occ}}=N^{-}_{\text{occ}}=N_{\text{occ}}/2$. Additionally, in a system with both $\mathcal{I}$ and $\mathcal{T}$ symmetry, we can choose the upper and lower spin bands to satisfy $\lambda^{+}_{n,\mathbf{k}}>0$ and $\lambda^{-}_{m,\mathbf{k}}<0$, respectively.

In terms of spinful orbital basis states, we can re-express $\left|\tilde{u}^{\pm}_{n,\mathbf{k}}\right\rangle$ as

$$\left|u^{\pm}_{n,\mathbf{k}}\right\rangle=\sum_{m=1}^{N_{\text{occ}}}[\tilde{u}^{\pm}_{n,\mathbf{k}}]_m\,|u_{m,\mathbf{k}}\rangle, \tag{3.7}$$

where $[\tilde{u}^{\pm}_{n,\mathbf{k}}]_m$ is the mth component of the nth N_{occ}-component eigenvector $\left|\tilde{u}^{\pm}_{n,\mathbf{k}}\right\rangle$ and $|u_{m,\mathbf{k}}\rangle$ is the $2N_{\text{orb}}$-component eigenvectors for the occupied bands of $[H(\mathbf{k})]$. We can also derive the boundary condition for $\left|u^{\pm}_{n,\mathbf{k}}\right\rangle$ as follows. Upon a shift $\mathbf{k}\to\mathbf{k}+\mathbf{G}$, we have

$$[s_{\text{reduced}}(\mathbf{k}+\mathbf{G})]_{m,n} \tag{3.8}$$

$$=\langle u_{m,\mathbf{k}+\mathbf{G}}|\,s\,|u_{n,\mathbf{k}+\mathbf{G}}\rangle \tag{3.9}$$

$$=\langle u_{m,\mathbf{k}}|\,[V(\mathbf{G})]s[V(\mathbf{G})]^{-1}\,|u_{n,\mathbf{k}}\rangle, \tag{3.10}$$

where the $[V(\mathbf{G})]$ matrix is defined in SN 2 A. If we assume that our orbital truncation is such that time-reversed pairs of orbitals are located at the same position (this is always possible, as it is true of atomic orbitals), we can factorize $[V(\mathbf{G})]$ into $[V(\mathbf{G})] = \sigma_0 \otimes [\widetilde{V}(\mathbf{G})]$ where $[\widetilde{V}(\mathbf{G})]$ is a unitary $N_{\text{orb}} \times N_{\text{orb}}$ matrix (recall that $2N_{\text{orb}}$ is the dimension of the Hilbert space of $[H(\mathbf{k})]$) with matrix element $[\widetilde{V}(\mathbf{G})]_{\widetilde{\alpha},\widetilde{\beta}} = \delta_{\widetilde{\alpha}\widetilde{\beta}} e^{i\mathbf{G}\cdot\mathbf{r}_{\widetilde{\alpha}}}$. Notice that the indices $\widetilde{\alpha}$ and $\widetilde{\beta}$ both range from $1 \ldots N_{\text{orb}}$ and $\mathbf{r}_{\widetilde{\alpha}}$ is the position of the orbital labeled by $\widetilde{\alpha}$. From this we can show that the matrix spin operator s is invariant under a unitary transformation of $[V(\mathbf{G})]$

$$[V(\mathbf{G})]s[V(\mathbf{G})]^{-1} \tag{3.11}$$

$$= \left(\sigma_0 \otimes [\widetilde{V}(\mathbf{G})]\right) (\hat{\mathbf{n}} \cdot \boldsymbol{\sigma} \otimes \mathbb{I}_{N_{\text{orb}}}) \left(\sigma_0 \otimes [\widetilde{V}(\mathbf{G})]^{-1}\right) \tag{3.12}$$

$$= \hat{\mathbf{n}} \cdot \boldsymbol{\sigma} \otimes \mathbb{I}_{N_{\text{orb}}} = s. \tag{3.13}$$

This implies

$$[s_{\text{reduced}}(\mathbf{k}+\mathbf{G})]_{m,n} = [s_{\text{reduced}}(\mathbf{k})]_{m,n}, \tag{3.14}$$

and the boundary condition for $\left|\tilde{u}^{\pm}_{n,\mathbf{k}}\right\rangle$ can be chosen as

$$\left|\tilde{u}^{\pm}_{n,\mathbf{k}+\mathbf{G}}\right\rangle = \left|\tilde{u}^{\pm}_{n,\mathbf{k}}\right\rangle, \tag{3.15}$$

such that the component $[\tilde{u}^{\pm}_{n,\mathbf{k}}]_m$ in SEq. (3.7) has the same boundary condition $[\tilde{u}^{\pm}_{n,\mathbf{k}+\mathbf{G}}]_m = [\tilde{u}^{\pm}_{n,\mathbf{k}}]_m$. We now define the projectors onto the upper and lower spin bands as

$$[P_{\pm}(\mathbf{k})] = \sum_{n=1}^{N^{\pm}_{\text{occ}}} \left|u^{\pm}_{n,\mathbf{k}}\right\rangle \left\langle u^{\pm}_{n,\mathbf{k}}\right|. \tag{3.16}$$

The occupied space matrix projector $[P(\mathbf{k})]$ is then equal to $[P_+(\mathbf{k})] + [P_-(\mathbf{k})]$ where $[P_+(\mathbf{k})][P_-(\mathbf{k})] = 0$. The corresponding holonomy matrix starting at base point $\mathbf{k}$ and going along a straight line path to $\mathbf{k}+\mathbf{G}$ for $[P_{\pm}(\mathbf{k})]$, which we term the $P_{\pm}$-Wilson loop matrix (or the spin-resolved Wilson loop matrix), is given by

$$[\mathcal{W}^{\pm}_{1,\mathbf{k},\mathbf{G}}]_{m,n} = \left\langle u^{\pm}_{m,\mathbf{k}+\mathbf{G}}\right| \left(\prod_{\mathbf{q}}^{\mathbf{k}+\mathbf{G}\leftarrow\mathbf{k}} [P_{\pm}(\mathbf{q})]\right) \left|u^{\pm}_{n,\mathbf{k}}\right\rangle$$

$$= \left\langle u^{\pm}_{m,\mathbf{k}}\right| [V(\mathbf{G})] \left(\prod_{\mathbf{q}}^{\mathbf{k}+\mathbf{G}\leftarrow\mathbf{k}} [P_{\pm}(\mathbf{q})]\right) \left|u^{\pm}_{n,\mathbf{k}}\right\rangle, \tag{3.17}$$

where

$$\left(\prod_{\mathbf{q}}^{\mathbf{k}+\mathbf{G}\leftarrow\mathbf{k}} [P_{\pm}(\mathbf{q})]\right) \tag{3.18}$$

$$\equiv \lim_{N\to\infty} [P_{\pm}(\mathbf{k}+\mathbf{G})][P_{\pm}(\mathbf{k}+\frac{N-1}{N}\mathbf{G})]\ldots[P_{\pm}(\mathbf{k}+\frac{1}{N}\mathbf{G})][P_{\pm}(\mathbf{k})], \tag{3.19}$$

and we have used the fact that, since $|u_{n,\mathbf{k}+\mathbf{G}}\rangle = [V(\mathbf{G})]^{-1}|u_{n,\mathbf{k}}\rangle$ (see SN 2 A) and $[\tilde{u}^{\pm}_{n,\mathbf{k}+\mathbf{G}}]_m = [\tilde{u}^{\pm}_{n,\mathbf{k}}]_m$, we have the boundary condition for $|u^{\pm}_{n,\mathbf{k}}\rangle$ as

$$\left|u^{\pm}_{n,\mathbf{k}+\mathbf{G}}\right\rangle = \sum_{m=1}^{N_{\text{occ}}} [\tilde{u}^{\pm}_{n,\mathbf{k}+\mathbf{G}}]_m |u_{m,\mathbf{k}+\mathbf{G}}\rangle \tag{3.20}$$

$$= \sum_{m=1}^{N_{\text{occ}}} [\tilde{u}^{\pm}_{n,\mathbf{k}}]_m [V(\mathbf{G})]^{-1} |u_{m,\mathbf{k}}\rangle \tag{3.21}$$

$$= [V(\mathbf{G})]^{-1} \sum_{m=1}^{N_{\text{occ}}} [\tilde{u}^{\pm}_{n,\mathbf{k}}]_m |u_{m,\mathbf{k}}\rangle \tag{3.22}$$

$$= [V(\mathbf{G})]^{-1} \left|u^{\pm}_{n,\mathbf{k}}\right\rangle. \tag{3.23}$$

The $P_\pm$-Wilson loop matrices $[\mathcal{W}^\pm_{1,\mathbf{k},\mathbf{G}}]$ [SEq. (3.17)] are $N^+_{\text{occ}} \times N^+_{\text{occ}}$ and $N^-_{\text{occ}} \times N^-_{\text{occ}}$ unitary matrices respectively. We can write the eigenvalues of $[\mathcal{W}^\pm_{1,\mathbf{k},\mathbf{G}}]$ as $\exp\{i(\gamma_1^\pm)_{j,\mathbf{k},\mathbf{G}}\}$ where $j = 1 \ldots N^\pm_{\text{occ}}$ index the $N^\pm_{\text{occ}}$ eigenvalues. We call the set $\{(\gamma_1^\pm)_{j,\mathbf{k},\mathbf{G}}\}$ as $P_\pm$-*Wannier bands* (or *spin-resolved Wannier bands*) because $\{(\gamma_1^\pm)_{j,\mathbf{k},\mathbf{G}}\}$ tells us the positions of spin-resolved hybrid Wannier functions formed from the subspace of upper (+) or lower (−) spin bands; the spin-resolved hybrid Wannier functions are localized along the lattice vector dual to $\mathbf{G}$. Similar to the eigenphases of the ordinary Wilson loops $[\mathcal{W}_{1,\mathbf{k},\mathbf{G}}]$, the eigenphases $\{(\gamma_1^\pm)_{j,\mathbf{k},\mathbf{G}}\}$ are invariant with respect to gauge transformations that do not change the boundary conditions on the Bloch wave functions. This follows from SEq. (2.16) as well as the definition in SEq. (3.7) of the PsP eigenstates. The eigenphases $\{(\gamma_1^\pm)_{j,\mathbf{k},\mathbf{G}}\}$ of the $P_\pm$-Wilson loop matrix are the Berry phases of the $P_\pm$-subspace of the N_{occ} occupied states for the closed loop in $\mathbf{k}$-space parallel to $\mathbf{G}$. Again, if we write the base point $\mathbf{k} = \sum_{j=1}^d \frac{k_j}{2\pi}\mathbf{G}_j$ where d is the spatial dimension of the system, and if we choose the closed loop holonomy to be along $\mathbf{G}_i$ with base point $\mathbf{k}$, then $\{(\gamma_1^\pm)_{j,\mathbf{k},\mathbf{G}_i}\}$ will be independent of the component $k_i = \mathbf{k}\cdot\mathbf{a}_i$ where $\{\mathbf{a}_i\}$ is the set of position-space lattice vectors dual to the reciprocal lattice vectors $\{\mathbf{G}_j\}$ such that $\mathbf{a}_i \cdot \mathbf{G}_j = 2\pi\delta_{ij}$ [146]. In addition, to directly specify the holonomy in SEq. (3.17), we will throughout this work refer to $\{(\gamma_1^\pm)_{j,\mathbf{k},\mathbf{G}}\}$ as a function of $\mathbf{k}$ as the $\widehat{\mathbf{G}}$-*directed* $P_\pm$-*Wannier bands* (or $\widehat{\mathbf{G}}$-*directed spin-resolved Wannier bands*), where j labels the band index. Similar to the case of $\widehat{\mathbf{G}}$-*directed* P-*Wannier bands* we may deduce the topological properties of the system by computing the spectral flow of $\{(\gamma_1^\pm)_{j,\mathbf{k},\mathbf{G}}\}$. In SN 6 E and 6 G we prove the constraints on the values of the $P_\pm$-Wilson loop eigenphases at different $\mathbf{k}$ points due to inversion and time-reversal symmetries, respectively. In the next SN 3 C we will discuss some general properties of spectral flow in the $P_\pm$-Wilson loop.

C. Spectral Flow of the $P_\pm$-Wilson Loop

In this section we discuss some general properties of the spectral flow of the $P_\pm$-Wilson loop eigenphases $\gamma_1^\pm$. To simplify the discussion, we will in this section specify to 2D spinful systems. We will not assume any symmetries to begin (including time-reversal symmetry) except for discrete translations. We take the primitive Bravais lattice vectors to be $\{\mathbf{a}_1, \mathbf{a}_2\}$, and the corresponding primitive reciprocal lattice vectors to be $\{\mathbf{G}_1, \mathbf{G}_2\}$ satisfying $\mathbf{a}_i \cdot \mathbf{G}_j = 2\pi\delta_{ij}$. The crystal momentum $\mathbf{k}$ can then be expanded using $\mathbf{k} = \sum_{i=1}^2 \frac{k_i}{2\pi}\mathbf{G}_i$ where $k_i = \mathbf{k}\cdot\mathbf{a}_i$. The BZ is then defined by the region with $k_i = [-\pi, \pi)$. Toward the end of this section we will additionally specialize to time-reversal ($\mathcal{T}$) invariant systems. We will also generalize to 3D systems in SN 3 E and 3 F.

We will suppose that both the energy and the spin gaps are open. This implies that the occupied-space projector $P(\mathbf{k})$ is well-defined and is a smooth function of $\mathbf{k}$, and that the eigenvectors of $P(\mathbf{k})sP(\mathbf{k})$ in Image$[P(\mathbf{k})]$ can be separated into upper and lower eigenspaces with projectors $P_\pm(\mathbf{k})$. The projectors $P_\pm(\mathbf{k})$ are similarly well-defined and smooth over the BZ. Let us denote the $\widehat{\mathbf{G}}_2$-directed $P_\pm$-Wilson loop eigenphase as $\{\gamma^\pm_{1,j}(k_1)\}$ where j is the $P_\pm$-Wannier band indices. Notice that, to facilitate the discussion in this subsection 3 C, unlike the most general notation $\{(\gamma_1^\pm)_{j,\mathbf{k},\mathbf{G}}\}$ introduced in SN 3 B, we have suppressed the subscripts for both $\mathbf{k}$ denoting the base point and $\mathbf{G}$ characterizing the holonomy such that only the k_1-dependence remains explicit in the expressions below. This is because the eigenphases are independent of k_2 for $\widehat{\mathbf{G}}_2$-directed $P_\pm$-Wilson loops. We can define the partial Chern numbers $C^\pm_{\gamma_1}$ as the *negative* winding number of $\operatorname{Im}\log\det\left[\mathcal{W}^\pm_{1,\mathbf{k},\mathbf{G}_2}\right]$ as a function of k_1, or equivalently the *positive* winding number of $\operatorname{Im}\log\det\left[\mathcal{W}^\pm_{1,\mathbf{k},\mathbf{G}_1}\right]$ as a function of k_2. In terms of the eigenphases $\{\gamma^\pm_{1,j}(k_1)\}$, we can write the partial Chern numbers as [1, 7]

$$C^\pm_{\gamma_1} = -\frac{1}{2\pi}\sum_{j=1}^{N^\pm_{\text{occ}}}\int_{-\pi}^{\pi} dk_1 \frac{\partial\gamma^\pm_{1,j}(k_1)}{\partial k_1} = -\frac{i}{2\pi}\int d^2k \operatorname{Tr}\left(P_\pm(\mathbf{k})\left[\frac{\partial P_\pm(\mathbf{k})}{\partial k_1}, \frac{\partial P_\pm(\mathbf{k})}{\partial k_2}\right]\right). \tag{3.24}$$

We see that the partial Chern numbers $C^\pm_{\gamma_1}$ are respectively equal to the Chern numbers of the subspaces of states in the images of $P_\pm(\mathbf{k})$. Denoting the Chern number of the occupied energy bands as C_{γ_1}, we can use $P_+(\mathbf{k}) + P_-(\mathbf{k}) = P(\mathbf{k})$ and $P_+(\mathbf{k})P_-(\mathbf{k}) = 0$ in conjunction with the second equality of SEq. (3.24) to show that (see for example SRef. [154])

$$C^+_{\gamma_1} + C^-_{\gamma_1} = C_{\gamma_1}. \tag{3.25}$$

As argued in SRef. [1], the partial Chern numbers $C^\pm_{\gamma_1}$ are topological invariants, in the sense that two systems with different partial Chern numbers cannot be adiabatically deformed into each other without closing either an energy gap or a spin gap. As shown in SN 2 C, this is a physically motivated constraint since both the energy gap and the spin gap are robust to external perturbations and hence physically meaningful. During a deformation of the Hamiltonian, although C_{γ_1} and $C^\pm_{\gamma_1}$ can change due to energy and spin gap closing respectively, they need to satisfy the exact relation in SEq. (3.25).

Following SRef. [1], we may also define a *spin Chern number*

$$C^s_{\gamma_1} \equiv C^+_{\gamma_1} - C^-_{\gamma_1}, \tag{3.26}$$

which can be viewed as the *relative winding number* of the Wilson loop eigenphases of the upper and lower $P(\mathbf{k})sP(\mathbf{k})$ eigenspaces. We have normalized $C^s_{\gamma_1}$ such that, for a system with conserved spin component $\hat{\mathbf{n}} \cdot \mathbf{s}$, the spin Hall conductivity is given by [6, 7, 140, 155–158]

$$\sigma^s_H = \left(\frac{\hbar}{2}\right)\left(\frac{e}{h}\right) C^s_{\gamma_1}, \tag{3.27}$$

where e is the (negative) electron charge.

SEq. (3.25) places constraints on how $C^s_{\gamma_1}$ can change when a spin gap closes. Suppose we deform the Hamiltonian in a way that the energy gap remains open while the spin spectrum undergoes a spin band inversion (*i.e.* the spin gap between the upper and lower spin bands closes and reopens). C_{γ_1} will remain invariant since the energy gap is open throughout the deformation. According to SEq. (3.25), $C^+_{\gamma_1}$ and $C^-_{\gamma_1}$ can only be changed by opposite integers in order to keep C_{γ_1} invariant. For example, before and after the spin band inversion, we can have

$$C_{\gamma_1} \to C_{\gamma_1}, \tag{3.28}$$

$$C^\pm_{\gamma_1} \to C^\pm_{\gamma_1} \pm n, \tag{3.29}$$

where $n \in \mathbb{Z}$. Therefore, as long as the energy gap remains open, $C^+_{\gamma_1}$ and $C^-_{\gamma_1}$ cannot change independently. However, absent additional symmetries, the values of $C^+_{\gamma_1}$ and $C^-_{\gamma_1}$ need not to be related. From the definition of $C^s_{\gamma_1}$ in SEq. (3.26), when $C^\pm_{\gamma_1} \to C^\pm_{\gamma_1} \pm n$, we have $C^s_{\gamma_1} \to C^s_{\gamma_1} + 2n$. Therefore, without an energy gap closing, it is possible to deform the Hamiltonian such that $C^s_{\gamma_1}$ is changed by an even integer $2n$ when the spin gap closes and reopens. Therefore, $(C^s_{\gamma_1})$ mod 2 is a $\mathbb{Z}_2$-stable topological invariant that cannot change even when the spin gap closes and reopens. This invariant specifically characterizes whether there is an even- or odd-integer difference between $C^+_{\gamma_1}$ and $C^-_{\gamma_1}$. Note that systems with $(C^s_{\gamma_1})$ mod $2 = 1$ cannot be time-reversal symmetric, since the Chern number $C_{\gamma_1} = C^+_{\gamma_1} + C^-_{\gamma_1} \neq 0$ if $C^+_{\gamma_1} - C^-_{\gamma_1}$ is odd.

As an example, let us consider a toy model of a Chern insulator with two spinful orbitals per unit cell. We suppose that $\hat{\mathbf{n}} \cdot \mathbf{s}$ commutes with the Hamiltonian, so that spin $\hat{\mathbf{n}} \cdot \mathbf{s}$ is a good quantum number. This means that we can compute the partial Chern numbers for the spin-up (along $+\hat{\mathbf{n}}$) and spin-down (along $-\hat{\mathbf{n}}$) occupied states separately. Suppose that the total Chern number $C_{\gamma_1} = 1$, the partial Chern number $C^+_{\gamma_1} = 1$ for the spin-up electrons, and the partial Chern number $C^-_{\gamma_1} = 0$ for the spin-down electrons. In this case the spin Chern number $C^s_{\gamma_1} = 1$. If we deform the Hamiltonian in a way that the energy gap does not close, but the spin gap can close and reopen, then there can be a transfer of partial Chern number between the upper and lower spin bands. Absent any (crystallographic or time-reversal) symmetry constraints, we can have, for instance, that

$$C^+_{\gamma_1} = 1 \to 2,\ C^-_{\gamma_1} = 0 \to -1, \tag{3.30}$$

such that $C_{\gamma_1} = C^+_{\gamma_1} + C^-_{\gamma_1}$ is still 1. The spin Chern number $C^s_{\gamma_1}$ has changed from 1 to 3. In this example, before and after the spin gap closing, $(C^s_{\gamma_1})$ mod 2 is always equal to 1. Furthermore, the energy gap has remained open throughout the deformation. Note that if the system has conserved spin component $\hat{\mathbf{n}} \cdot \mathbf{s}$ before and after the deformation, then the spin Hall conductivity [SEq. (3.27)] must have changed by $e/(2\pi)$ times an integer.

For non-interacting systems with time-reversal symmetry, we have that $C^s_{\gamma_1}$ must be even, and

$$C^s_{\gamma_1} = 2C^+_{\gamma_1} = -2C^-_{\gamma_1}. \tag{3.31}$$

SEq. (3.31) follows from inserting SEq. (2.37) in the definition [SEqs. (3.24) and (3.26)] of the spin Chern number. SEq. (3.31) means that for time-reversal-symmetric systems, the partial Chern number $C^+_{\gamma_1}$ determines the spin Chern number $C^s_{\gamma_1}$. In fact, it was established in SRefs. [1, 2, 159] that for time-reversal-symmetric systems $C^s_{\gamma_1}$ mod 4 (or alternatively $C^\pm_{\gamma_1}$ mod 2) can be directly related to the two-dimensional strong $\mathbb{Z}_2$-valued index ν_{2d} for 2D topological insulators introduced by Kane and Mele [6, 160]. To see this, let us consider a deformation of the Hamiltonian that preserves $\mathcal{T}$ symmetry and does not close an energy gap, while allowing the spin gap to close and reopen. Due to $\mathcal{T}$ symmetry, if the spin gap closes at $\mathbf{k}$, then the spin gap will also close at $-\mathbf{k}$. This means that $C^\pm_{\gamma_1}$ can in general change only by even integers, such that

$$C^\pm_{\gamma_1} \to C^\pm_{\gamma_1} \pm 2n, \tag{3.32}$$

where $n \in \mathbb{Z}$. Although the states at the spin gap closing points $\mathbf{k}$ and $-\mathbf{k}$ are related by time-reversal, the same quantity of partial Chern number is transferred from the lower spin bands to the upper spin bands at each point. This is because unlike for energy bands, time-reversal symmetry interchanges the upper and lower spin bands. Furthermore, if the spin gap closes between two spin bands at a TRIM, $\mathcal{T}$ symmetry requires that the spin band dispersion near the gap closing point is quadratic; hence $C^{\pm}_{\gamma_1}$ can also only change by an even integer [161]. SEq. (3.32) implies that $C^s_{\gamma_1}$ can only change according to

$$C^s_{\gamma_1} \to C^s_{\gamma_1} + 4n. \tag{3.33}$$

We then see that with $\mathcal{T}$ symmetry, $1/2(C^s_{\gamma_1} \mod 4)$ is a stable $\mathbb{Z}_2$ invariant as first shown by Prodan [1]. This is consistent the crystallographic splitting theorem of SRef. [162].

To further establish that $1/2(C^s_{\gamma_1} \mod 4)$ coincides with the Kane-Mele $\mathbb{Z}_2$ invariant, let us consider a $\mathcal{T}$-invariant system with $C^{\pm}_{\gamma_1} = \pm 1$ such that $C^s_{\gamma_1} = 2$. Recall that $\{\gamma^{\pm}_{1,j}(k_1)\}$ are the localized positions along $\mathbf{a}_2$ of the spin-resolved hybrid Wannier functions in the upper and lower PsP eigenspaces, and the partial Chern numbers $C^{\pm}_{\gamma_1}$ are the corresponding *negative* net winding numbers of the spin-resolved hybrid Wannier centers when $k_1 \to k_1 + 2\pi$. Since $\mathcal{T}P_{\pm}(\mathbf{k})\mathcal{T}^{-1} = P_{\mp}(-\mathbf{k})$, the P_+- and P_--hybrid Wannier functions are related to each other by time-reversal symmetry. $C^{\pm}_{\gamma_1} = \pm 1$ then means that we cannot form exponentially-localized Wannier functions using only states in the image of $P_{\pm}$, since the spin-resolved hybrid Wannier functions have a discontinuity when $k_1 \to k_1 + 2\pi$ [163]. Thus, we see that $1/2(C^s_{\gamma_1} \mod 4) = 1$ quantifies the obstruction to forming exponentially-localized Wannier functions related to each other by time-reversal symmetry, and so coincides with ν_{2d} [163]. Hence an odd winding in the $P_{\pm}$-Wilson loops implies *helical winding* protected by $\mathcal{T}$ symmetry in the centers of the ordinary hybrid Wannier functions [3, 147] computed using the P-Wilson loop formalism (SN 3 A). We will numerically demonstrate this in SN 3 D.

Note also that a nonzero spin Chern number $C^s_{\gamma_1}$ implies an intrinsic contribution to the spin Hall conductivity. For systems with conserved component $\hat{\mathbf{n}} \cdot \mathbf{s}$ of the spin, we have that the spin Hall conductivity is directly proportional to the spin Chern number via SEq. (3.27); this follows from Thouless-Komohto-Nightingale-den Nijs expression for the Kubo formula for the Hall conductivity [164] applied separately to spin-up and spin-down states [7]. When spin is not conserved, the Kubo formula for the spin Hall conductivity takes the more complicated form

$$\sigma^s_H = \frac{e}{4\pi}(\sigma^s_I + \sigma^s_{II}), \tag{3.34}$$

where (using $\mathrm{Tr}(\mathcal{O})$ to denote the trace of operator $\mathcal{O}$)

$$\sigma^s_I = -\frac{i}{2\pi}\int d^2k \mathrm{Tr}\left(sP\left[\frac{\partial P}{\partial k_1}, \frac{\partial P}{\partial k_2}\right]\right) \tag{3.35}$$

describes spin transport, and σ^s_{II} quantifies the rate of change of the spin density due to spin nonconservation in the Hamiltonian [108, 165–167]. Our definition of σ^s_{II} differs slightly from that in SRef. [108] in that we have chosen to include in σ^s_{II} *all* contributions to the spin Hall conductivity that vanish when spin is conserved. The spin Chern number gives a topological contribution to σ^s_I even when spin is not conserved. Writing $P(\mathbf{k}) = P_+(\mathbf{k}) + P_-(\mathbf{k})$ and inserting a complete set $\left|u^{\pm}_{n\mathbf{k}}\right\rangle$ of states in the image of $P(\mathbf{k})$, we can write

$$\sigma^s_I = C^s_{\gamma_1} + \frac{i}{2\pi}\int d^2k \left[2\mathrm{Tr}\left(P_+\left[\frac{\partial P_-}{\partial k_1}, \frac{\partial P_-}{\partial k_2}\right]\right) - \sum_{\alpha=\pm}\sum_n \beta^{\alpha}_{n\mathbf{k}} \left\langle u^{\alpha}_{n\mathbf{k}}\right| P\left[\frac{\partial P}{\partial k_1}, \frac{\partial P}{\partial k_2}\right]\left|u^{\alpha}_{n\mathbf{k}}\right\rangle\right], \tag{3.36}$$

where we have introduced $\beta^{\pm}_{n\mathbf{k}} = \pm 1 - \lambda^{\pm}_{n\mathbf{k}}$. The first term in SEq. (3.36) is the spin Chern number [SEq. (3.26)]. The second term quantifies the fact that if spin is not conserved, the lower PsP eigenstates at $\mathbf{k}$ will have nonzero overlap with upper PsP eigenstates at neighboring $\mathbf{k} + \delta\mathbf{k}$. Finally, the third term in SEq. (3.36) gives the integral of the diagonal matrix elements of the ordinary Berry curvature between PsP eigenstates, weighted by the deviation of their PsP eigenvalue from ± 1. Thus, $\sigma^s_I - C^s_{\gamma_1}$ will be small when $\hat{\mathbf{n}} \cdot \mathbf{s}$ non-conserving SOC is weak. Additionally, it was established in SRefs. [108, 168] that σ^s_{II} is also small when $\hat{\mathbf{n}} \cdot \mathbf{s}$ non-conserving SOC is weak. Hence, we have shown that the spin Chern number indicates the leading intrinsic (topological) contribution to the spin Hall conductivity for weak $\hat{\mathbf{n}} \cdot \mathbf{s}$ non-conservation. In SN 10 C, we will validate this result under realistic conditions by computing the bulk spin Hall conductivity for several choices of $\hat{\mathbf{n}}$ for the candidate helical HOTI α-BiBr [50, 52, 53], which we find to have two independent spin-gapped regimes. Remarkably, we find that in both independent spin-gapped regimes, the intrinsic bulk contribution to the spin Hall conductivity lies in close agreement with a quantized value proportional to the spin Chern number, even when the spin gap is as small as $\Delta_s \sim 0.25$.

Finally, the relation $\nu_{2d} = (1/2)C^s_{\gamma_1} \mod 2$ in 2D allows us to deduce some general features of the spin spectrum for 3D topological insulators. Recall that for a 3D strong topological insulator, if we evaluate the 2D strong $\mathbb{Z}_2$ invariant

$\nu_{2d}(k_i)$ over two parallel time-reversal invariant planes with $k_i = 0$ and $k_i = \pi$ $(i = 1, 2, 3)$, then $|\nu_{2d}(0) - \nu_{2d}(\pi)| = 1$. This implies that the spin Chern numbers evaluated on these planes satisfy $|C^s_{\gamma_1}(0) - C^s_{\gamma_1}(\pi)| = 2 + 4n$ for some integer n. Since the spin Chern number $C^s_{\gamma_1}(k_i)$ can be defined over any constant-k_i plane over which the spin gap is open, the only way for the $C^s_{\gamma_1}$ to change is if the spin gap closes for some k_i. As such, the spin spectrum must have an odd number of gap closing points between the $k_i = 0$ and $k_i = \pi$ planes [169]. Just like for energy bands, the Wigner-von Newmann theorem tells us that, absent additional crystal symmetries, the spin gap closing points at generic $\mathbf{k}$ will occur as isolated twofold degeneracies with linearly dispersing spin bands in all directions [170]. Therefore, a 3D strong topological insulator must have a *gapless* spin spectrum. For an analytic demonstration, we refer the readers to SN 2 E where we perform an explicit computation using a model of 3D strong topological insulator. We will come back to this statement later in SN 3 E and 3 F when we numerically demonstrate the application of our $P_\pm$-Wilson loop formalism to 3D strong topological insulators.

D. 2D Spinful Time-Reversal-Invariant Systems

In this section, we will numerically demonstrate the application of the $P_\pm$-Wilson loop formalism in SN 3 B to resolve the nontrivial topology in the spin spectrum by considering a 2D strong TI with spinful time-reversal ($\mathcal{T}$) symmetry. We will also numerically confirm the symmetry constraints for the $P_\pm$-Wilson loop spectra we derived in SN 6 G. In the following discussion, we will refer to the eigenvalues of the reduced spin matrix introduced in SEq. (3.5) with $\hat{\mathbf{n}}$ a unit vector along the ith Cartesian coordinate direction as the "s_i spectrum" or the "s_i eigenvalues." The 2D strong TI model we consider is a four-band lattice model with orbital (s and p) and spin ($\uparrow$ and $\downarrow$) degrees of freedom represented by Pauli matrices τ_μ and σ_ν, respectively. The corresponding four-band (momentum-space) Bloch Hamiltonian $[H(\mathbf{k})]$ is given by [79, 140, 142]

$$\begin{aligned}[H(\mathbf{k})] = {} & [\epsilon - t_{1,x}\cos(k_x) - t_{1,y}\cos(k_y)]\tau_z\sigma_0 + t_{2,x}\sin(k_x)\tau_y\sigma_0 + t_{2,y}\sin(k_y)\tau_x\sigma_z \\ & + t_R(\sin(k_y)\tau_0\sigma_x - \sin(k_x)\tau_0\sigma_y) + [t_{PH,x}\cos(k_x) + t_{PH,y}\cos(k_y)]\tau_0\sigma_0 \\ & + t_{M_x}\cos(k_x)\tau_y\sigma_x + t_{M_y}\cos(k_y)\tau_y\sigma_y + t_{\mathcal{I}}\cos(k_x)\tau_x\sigma_0, \end{aligned} \tag{3.37}$$

Both τ_0 and σ_0 are 2×2 identity matrices. SEq. (3.37) was formulated by adding additional terms to the Bernevig-Hughes-Zhang (BHZ) model of a 2D strong TI [79, 140, 142] to break rotation and mirror symmetries of that model. We will choose the following values for the parameters of the model:

$$\epsilon = 1.0, t_{1,x} = 0.8, t_{1,y} = 1.2, t_{2,x} = 1.3, t_{2,y} = 0.9, t_R = 0.8, t_{PH,x} = 0.3, t_{PH,y} = 0.4, t_{M_x} = 0.3, t_{M_y} = 0.2, t_{\mathcal{I}} = 1.0. \tag{3.38}$$

The model in SEq. (3.37) is $\mathcal{T}$-symmetric, where $\mathcal{T}$ symmetry is represented as $[\mathcal{T}] = i\sigma_y\mathcal{K}$, and $\mathcal{K}$ represents complex conjugation. For the parameter values given in SEq. (3.38), the model does not have mirror or twofold rotation symmetries. The spectrum and s_z spin spectrum for this model are shown in SFig. 2, where we see that there is both an energy gap and a spin gap.

Instead of using $\{(\gamma_1)_{j,\mathbf{k},\mathbf{G}}\}$ and $\{(\gamma_1^\pm)_{j,\mathbf{k},\mathbf{G}}\}$ to denote the set of eigenphases of the P- and $P_\pm$-Wilson loop matrices, in this section we will denote them as $\{\gamma_{1,j}(k_\perp)\}$ and $\{\gamma^\pm_{1,j}(k_\perp)\}$ to emphasize that the eigenphases depend only on $k_\perp$. For example, in a 2D orthorhombic system, which is the case we will consider below, if we choose $\mathbf{G} = 2\pi\hat{\mathbf{x}}$ for the holonomy, the corresponding eigenphases for the k_x-directed P- and $P_\pm$-Wilson loop matrices will be denoted as $\{\gamma_{1,j}(k_y)\}$ and $\{\gamma^\pm_{1,j}(k_y)\}$, respectively.

In addition to helical edge states, another characteristic of a 2D strong TI is the presence of an odd helical winding in the P-Wilson loop spectrum [3]. In SFig. 3(a), we show the eigenphases γ_1 of the k_x-directed P-Wilson loop matrix [SEq. (3.2)] as a function of k_y, where we have chosen $\mathbf{G} = 2\pi\hat{\mathbf{x}}$ in SN 3 A. The two-fold degeneracies at $k_y = 0$ and $k_y = \pi$ in SFig. 3(a) are due to Kramers theorem, and thus the helical winding, if it exists, is protected as long as the $\mathcal{T}$ symmetry is preserved [3, 147]. Since the model [SEq. (3.37)] with parameters in SEq. (3.38) has only translation and time-reversal symmetries, the eigenphases $\{\gamma_{1,j}(k_y)\}$ take generic values at $k_y = 0, \pi$, as shown in SFig. 3(a); there are no improper rotation symmetries (such as mirror symmetries) to quantize $\{\gamma_{1,j}(k_y = 0, \pi)\}$[107]. For a detailed discussion of how crystalline symmetries can quantize the eigenphases, we refer the readers to SN 6 A where we specifically consider the effect of inversion symmetry on the P-Wilson loop eigenphases.

To systematically resolve the topology in the spin spectrum, we first verify that there is a gap in the s_z spectrum computed in the occupied two-band valence space throughout the 2D BZ, as shown in SFig. 2(c). Note that the Ps_zP eigenvalues deviate from unity, indicating that s_z is not conserved. This will enable us to decompose the occupied space into two parts and compute the $P_\pm$-Wilson loop eigenphases in the occupied space. We will again choose $\mathbf{G} = 2\pi\hat{\mathbf{x}}$ in SN 3 B. As shown in SFig. 3(b,c), such k_x-directed P_+- and P_--Wilson loop eigenphases as a function of k_y are related to each other by

$$\{\gamma^\pm_{1,j}(k_y)\} \bmod 2\pi = \{\gamma^\mp_{1,j}(-k_y)\} \bmod 2\pi \tag{3.39}$$

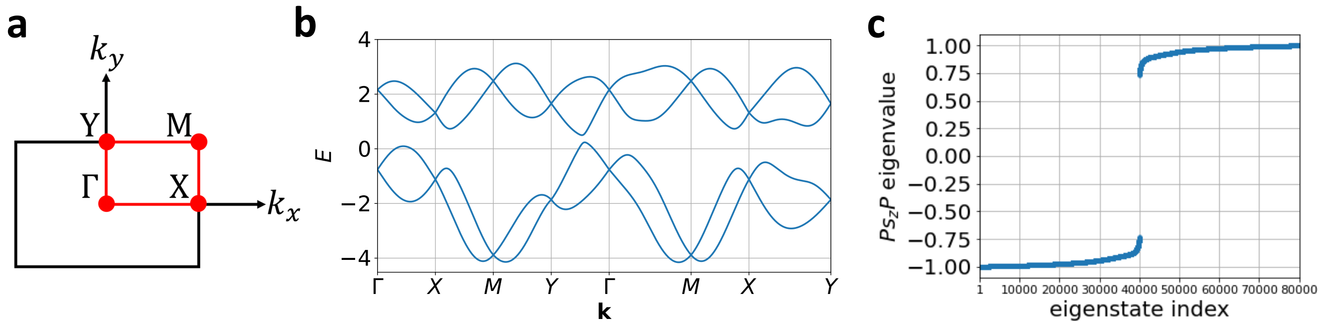

Supplementary Figure 2. Brillouin zone, energy spectrum, and s_z spin spectrum for the 2D TI model in SEqs. (3.37) and (3.38). (a) shows the first BZ of a 2D orthorhombic lattice. (b) shows the bulk energy bands of $[H(\mathbf{k})]$ [SEq. (3.37)] with tight-binding parameters in SEq. (3.38). (c) shows the Ps_zP eigenvalues for the lowest two bands in (b) evaluated on a uniformly sampled grid of $\mathbf{k}$ points in the 2D BZ. The (square) grid size used in the sampling is $(\Delta k_x, \Delta k_y) = (0.01\pi, 0.01\pi)$ such that the number of $\mathbf{k}$ points is 200×200. The calculations detailed in this figure were performed using the freely available Python package NESTED_AND_SPIN_RESOLVED_WILSON_LOOP [97], which represents an extension of the PythTB open-source Python tight-binding package [171] that was implemented and utilized for the preparation of SRefs. [8, 15] and the present work.

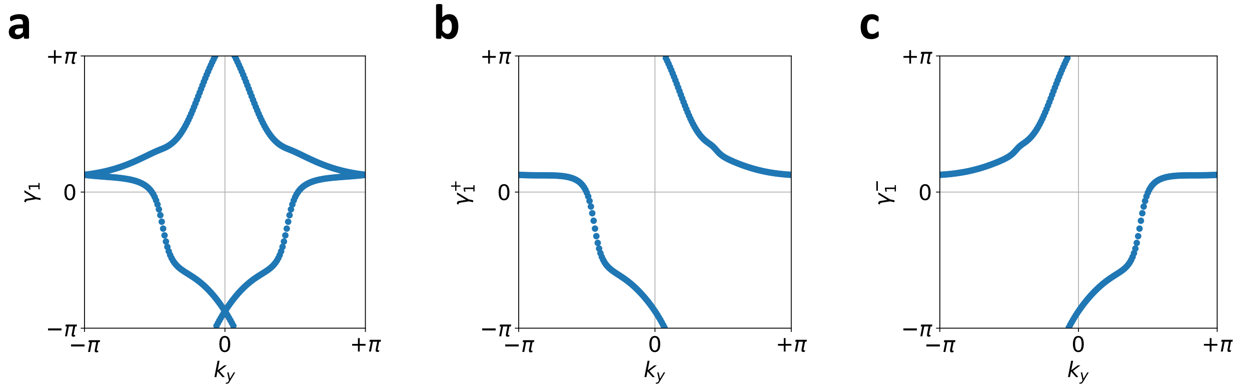

Supplementary Figure 3. Wilson loops and spin-resolved Wilson loops for a 2D strong TI with $\mathcal{T}$ symmetry. The Bloch Hamiltonian is given by SEq. (3.37) with tight-binding parameters in SEq. (3.38). And the occupied energy bands are chosen as the lowest two energy bands. (a) shows the eigenphases $\{\gamma_{1,j}(k_y)\}$ of the k_x-directed P-Wilson loop matrix [SEq. (3.2)] as a function of k_y. There are two bands that demonstrate an odd helical winding. (b) and (c) show the eigenphases $\{\gamma^{\pm}_{1,j}(k_y)\}$ of the k_x-directed $P_\pm$-Wilson loop matrix [SEq. (3.17)] as a function of k_y. There is one band for each of (b) and (c). The spin-resolved Wilson loop spectra in (b) and (c) demonstrate nonzero net winding. For the spectrum in (b), as $k_y \to k_y + 2\pi$ we have winding number -1 corresponding to the partial Chern number $C^+_{\gamma_1} = -1$ [using the sign convention introduced in SEq. (3.24)] while for the spectrum in (c) we have winding number $+1$ corresponding to the partial Chern number $C^-_{\gamma_1} = +1$. The calculations detailed in this figure were performed using the freely available Python package NESTED_AND_SPIN_RESOLVED_WILSON_LOOP [97], which represents an extension of the PythTB open-source Python tight-binding package [171] that was implemented and utilized for the preparation of SRefs. [8, 15] and the present work.

where $\{\gamma^{\pm}_{1,j}(k_y)\}$ is the set of the eigenphases of the k_x-directed $P_\pm$-Wilson loop matrix [SEq. (3.17)], and j is the corresponding band indices. This is the 2D counterpart of the $\mathcal{T}$ constraints on the $P_\pm$-Wilson loops that we will derive in SN 6 G. Indeed, SEq. (3.39) follows directly from the definition of the $P_\pm$-Wilson loop matrix, combined with SEq. (2.37). This constrains the winding numbers of $\{\gamma^{+}_{1,j}(k_y)\}$ and $\{\gamma^{-}_{1,j}(k_y)\}$ as $k_y \to k_y + 2\pi$ to be opposite. In our specific examples, we see that the winding numbers of the k_x-directed $P_\pm$-Wilson loop spectra are given by ∓ 1. From SEq. (3.24), the partial Chern numbers are then given by $C^{\pm}_{\gamma_1} = \mp 1$, where we recall that the partial Chern numbers are given by the winding number of $\{\gamma^{\pm}_{1,j}(k_y)\}$ as $k_y \to k_y + 2\pi$ [78, 148, 172]. This is consistent with the fact that the subspace of upper and lower spin bands are not invariant under $\mathcal{T}$. However, $\mathcal{T}$ symmetry enforces the constraint that $C^+_{\gamma_1} = -C^-_{\gamma_1}$ as we have stated in SEq. (3.31).

From our definition in SEq. (3.26) we have that the spin Chern number of this model is given by

$$C^s_{\gamma_1} = -2. \tag{3.40}$$

As mentioned in SN 3C, for a system with $\mathcal{T}$ symmetry, $1/2(C^s_{\gamma_1} \mod 4)$ gives the 2D $\mathbb{Z}_2$ Kane-Mele invariant ν_{2d} [1, 114]. Therefore, SEq. (3.40) means that our 2D model in SEq. (3.37) with parameters in SEq. (3.38) is indeed a 2D strong TI with nontrivial strong $\mathbb{Z}_2$ invariant $\nu_{2d} = 1$.

As discussed in the text surrounding SEq. (3.33), the reason that $2\nu_{2d} = C^s_{\gamma_1} \mod 4$ is because $C^s_{\gamma_1}$ can change by a multiple of 4 under $\mathcal{T}$-symmetric adiabatic deformations of the Hamiltonian that close a spin gap but not an energy gap. To see this concretely, we follow the logic of SRef. [1] and construct a deformation of the Hamiltonian $[H(\mathbf{k})]$ in SEq. (3.37) through a unitary transformation

$$[U(\phi)] = \exp\left\{\frac{-i\phi\sigma_x}{2}\right\} = \sigma_0 \cos\frac{\phi}{2} - i\sigma_x \sin\frac{\phi}{2}, \tag{3.41}$$

which corresponds to a rotation of the spin vector about the x-axis by an angle ϕ. The transformed Hamiltonian as a function of ϕ is denoted as $[H(\mathbf{k},\phi)] = [U(\phi)][H(\mathbf{k})][U(\phi)]^\dagger$. Since $\mathcal{T}$ is represented as

$$[\mathcal{T}] = i\sigma_y\mathcal{K} \tag{3.42}$$

with $\mathcal{K}$ denoting the complex conjugation operator, it follows that

$$[\mathcal{T}][H(\mathbf{k},\phi)][\mathcal{T}]^{-1} = [H(-\mathbf{k},\phi)] \tag{3.43}$$

This means that $[H(\mathbf{k},\phi)]$ is still $\mathcal{T}$-invariant. For simplicity, we will turn off all the tight-binding parameters in SEq. (3.38) except for ϵ, $t_{1,x}$, $t_{1,y}$, $t_{2,x}$, and $t_{2,y}$. We will further choose $\epsilon = 1.0$, $t_{1,x} = t_{1,y} = t_{2,x} = t_{2,y} = 1.0$, which does not close the energy gap between the conduction and valence bands, and does not change the winding numbers of the $P_\pm$-Wilson loop eigenphases from their values in SFig. 3(b,c). In other words, when $\phi = 0$, we have $C^\pm_{\gamma_1} = \mp 1$. Since $[U(\pi)]^\dagger\sigma_z[U(\pi)] = -\sigma_z$, it can then be deduced that when $\phi = \pi$, the same eigenstates of the reduced s_z matrix that we originally labeled as having positive [negative] Ps_zP eigenvalues will at $\phi = \pi$ be labeled as having negative [positive] Ps_zP eigenvalues. Therefore, when $\phi = \pi$, we have instead $C^\pm_{\gamma_1} = \pm 1$. This means that the spin Chern number will change from $C^s_{\gamma_1} = -2$ to $C^s_{\gamma_1} = +2$ when we deform the Hamiltonian from $\phi = 0$ to $\phi = \pi$. As the partial Chern numbers $C^\pm_{\gamma_1}$ give the Chern numbers for the upper and lower Ps_zP bands, respectively, the spin gap must close and reopen throughout the deformation from $\phi = 0$ to $\phi = \pi$ in order to transfer -2 partial Chern number from the upper to lower spin bands such that $C^\pm_{\gamma_1}$ change from ∓ 1 to ± 1. In fact, with the simpler tight-binding parameter choice we mentioned above, it can be shown analytically that the gap in the Ps_zP spectrum closes when $\phi = \pi/2$. To see this, note that since $[U(\pi/2)]^\dagger\sigma_z[U(\pi/2)] = \sigma_y$, the Ps_zP eigenvalues of the unitary-transformed model with $\phi = \pi/2$ are the same as the Ps_yP eigenvalues of the model before the transformation. Before the deformation ($\phi = 0$), with the simpler tight-binding parameter choice, we have at $(k_x, k_y) = (0, \pm\pi/2)$ the Bloch Hamiltonian

$$[H(0,\pm\pi/2)] = \pm\tau_x\sigma_z. \tag{3.44}$$

Similar to the analysis in SN 2F, we first obtain the corresponding two valence eigenvectors

$$|1_\pm\rangle = \frac{1}{\sqrt{2}}\begin{bmatrix}1\\ \mp 1\end{bmatrix} \otimes \begin{bmatrix}1\\0\end{bmatrix}, \tag{3.45}$$

$$|2_\pm\rangle = \frac{1}{\sqrt{2}}\begin{bmatrix}1\\ \pm 1\end{bmatrix} \otimes \begin{bmatrix}0\\1\end{bmatrix}, \tag{3.46}$$

where $|1_\pm\rangle$ and $|2_\pm\rangle$ denote the first and second eigenvectors of $[H(0,\pm\pi/2)]$ with eigenvalue -1. It can be then checked that the reduced s_y spin matrix $[s_{y,\text{reduced}}(\mathbf{k})]$ defined in SEq. (3.5) is a 2×2 matrix of zeros at both $(k_x, k_y) = (0, \pm\pi/2)$. This means that the Ps_yP eigenvalues for both $[H(0,\pm\pi/2)]$ are zeros with two-fold degeneracy. In other words, the gap in the Ps_yP spectrum is closed at $(k_x, k_y) = (0, \pm\pi/2)$ for the Bloch Hamiltonian before the deformation ($\phi = 0$). Therefore, the gap in the Ps_zP spectrum of the unitary-transformed model $[U(\pi/2)][H(\mathbf{k})][U(\pi/2)]^\dagger$ is closed. Notice that $\mathcal{T}$ symmetry requires that if the spin gap is closed at $\mathbf{k}$, there must be another spin gap closing point at $-\mathbf{k}$. This is because the projected spin operator $s(\mathbf{k}) = P(\mathbf{k})sP(\mathbf{k})$ satisfies

$$[\mathcal{T}]s(\mathbf{k})[\mathcal{T}]^{-1} = -s(-\mathbf{k}). \tag{3.47}$$

Also note that since we are performing a unitary transformation [SEq. (3.41)] on the Hamiltonian, the energy spectrum is unchanged throughout the deformation. However, we have changed $C^s_{\gamma_1}$ by $+4$. The upshot is that, with

$\mathcal{T}$ symmetry, if we only maintain the (Hamiltonian) energy gap, then through a closing of the spin gap we are able to change the spin Chern number [SEq. (3.26)] by a multiple of 4 [1, 114]. This, correspondingly, results in a measurable change of the (topological contribution to the) spin Hall conductivity (in our example, it interchanges spin-up and spin-down electrons, which reverses the sign of the spin Hall conductivity).

We can interpret SFig. 3(b,c) in terms of the spectral flow of hybrid Wannier centers for hybrid Wannier functions composed of upper and lower Ps_zP eigenstates. In particular, we see that as k_y is adiabatically varied from $-\pi$ to π, the centers of hybrid Wannier functions formed from P_+ states shift one unit cell to the left along the x direction. Similarly, the centers of hybrid Wannier functions formed from P_- states—which are the time-reversed partners of the P_+ hybrid Wannier functions—shift one unit cell to the right along the x direction. Notice that such a behavior can also be seen in the ordinary P-Wilson loop calculation, as shown in SFig. 3(a). If we trace the individual bands in SFig. 3(a) in a smooth and continuous way, we will also obtain two distinct windings that are related to each other by time-reversal symmetry [such as the reflection with respect to the $k_y = 0$ axis in SFig. 3(b,c)], with opposite winding numbers. We emphasize that, in this section, we have demonstrated the procedure for spin-resolving the Wilson loop spectrum by first decomposing the occupied space into two parts that are related to each other through a spinful $\mathcal{T}$ operation, and then performing the Wilson loop calculation within each of the two separate subspaces. Although it may seem from the simple example of a 2D TI considered in this section that the $P_\pm$-Wilson loop eigenvalues and general features [SFig. 3(b,c)] can be obtained via a straightforward decomposition of the P-Wilson loop spectrum [SFig. 3(a)], in general this is not the case. In SN 3 G, we will analyze a more complicated model of a 2D fragile topological insulator with more pronounced differences between the $P_\pm$-Wilson loops and naive "halves" of the P-Wilson loop spectrum. In subsequent sections we will further show how the spin-resolved Wilson loop can be employed to analyze 3D strong TIs (SN 3 E and 3 F), 2D fragile TIs (SN 3 G), and higher-order topological phases (SN 4 E).

We conclude by summarizing the properties of 2D strong TIs shown in this section:

1. The P-Wilson loop eigenphases have spectral flow with odd helical winding protected by $\mathcal{T}$ symmetry [SFig. 3(a)].
2. The $P_\pm$-Wilson loop eigenphases have spectral flow with opposite odd winding numbers [SFig. 3(b,c)].
3. The spin Chern number $C^s_{\gamma_1}$ defined in SEq. (3.26) indicates the 2D strong $\mathbb{Z}_2$ invariant $\nu_{2d} = 1/2(C^s_{\gamma_1} \mod 4)$, such that for a 2D strong TI, $C^s_{\gamma_1} = 2 + 4n$ where $n \in \mathbb{Z}$.

E. 3D Spinful Time-Reversal-Invariant Systems with Inversion Symmetry

In this section, we will demonstrate that the $P_\pm$-Wilson loops can also be used to detect a 3D TI with spinful $\mathcal{T}$ symmetry. We will also verify that spin gap closing points in a 3D system play the role of Berry curvature monopoles for the partial Chern numbers $C^\pm_{\gamma_1}$, defined in SEq. (3.24) as the winding numbers of $P_\pm$-Wilson loop eigenphases. We term such spin gap closing points with nonzero partial Berry flux as *spin-Weyl nodes.*

We begin by introducing a 4-band spinful $\mathcal{T}$ and $\mathcal{I}$ symmetric 3D TI with an orthorhombic lattice, where at the origin of each unit cell we place a spinful s and a spinful p orbital. The corresponding four-band (momentum-space) Bloch Hamiltonian matrix is given by [15]

$$[H(\mathbf{k})] = m\tau_z\sigma_0 + \sum_{i=x,y,z} (t_{1,i}\cos(k_i)\tau_z\sigma_0 + t_{PH,i}\cos(k_i)\tau_0\sigma_0 + t_{2,i}\sin(k_i)\tau_y\sigma_0 + v_{1,i}\sin(k_i)\tau_x\sigma_i)$$
$$+ v_{2,xy}[\sin(k_x) + \sin(k_y)]\tau_x\sigma_z + v_{2,z}\sin(k_z)\tau_x\sigma_x. \quad (3.48)$$

where again τ_μ and σ_ν are Pauli matrices describing the orbital (s and p) and spin ($\uparrow$ and $\downarrow$) degrees of freedom respectively, and both τ_0 and σ_0 are 2×2 identity matrices. The Bloch Hamiltonian [SEq. (3.48)] is formulated by adding additional terms to the BHZ model of a 3D strong TI to break symmetries of that model. We will choose the following parameter values:

$$m = -5,\ t_{1,x} = 2.3,\ t_{1,y} = 2.5,\ t_{1,z} = 3,\ t_{PH,x} = t_{PH,y} = 0.3,\ t_{PH,z} = 0,\ t_{2,x} = 0.9,\ t_{2,y} = t_{2,z} = 0,$$
$$v_{1,x} = v_{1,y} = 3.2,\ v_{1,z} = 2.4,\ v_{2,xy} = 1.5,\ v_{2,z} = 0.4. \quad (3.49)$$

$[H(\mathbf{k})]$ in SEq. (3.48) with the parameter values specified in SEq. (3.49) is invariant under 3D lattice translation, inversion, and spinful time-reversal symmetries. In particular, inversion and time-reversal symmetries are represented as

$$[\mathcal{I}][H(\mathbf{k})][\mathcal{I}]^{-1} = \tau_z\sigma_0[H(\mathbf{k})]\tau_z\sigma_0 = [H(-\mathbf{k})], \quad (3.50)$$
$$[\mathcal{T}][H(\mathbf{k})][\mathcal{T}]^{-1} = \tau_0\sigma_y[H(\mathbf{k})]^*\tau_0\sigma_y = [H(-\mathbf{k})], \quad (3.51)$$

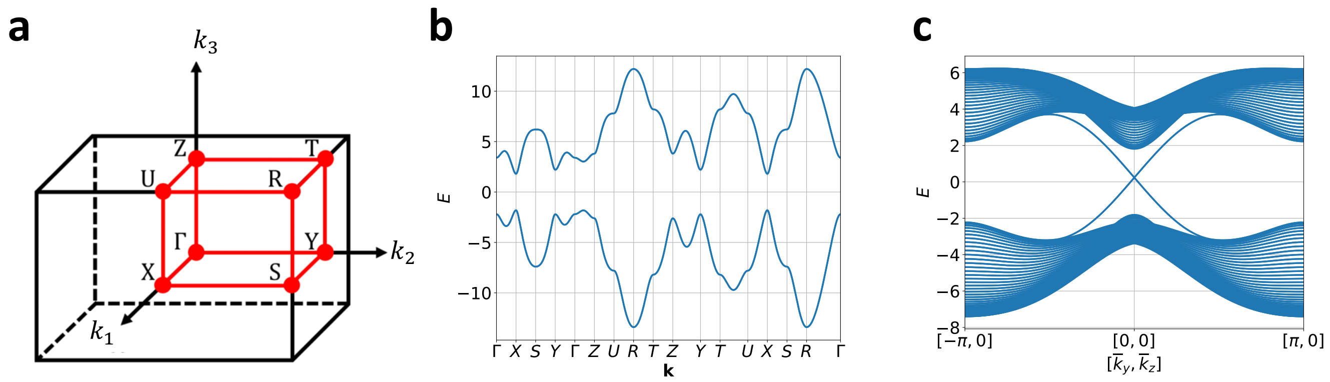

Supplementary Figure 4. Spectrum for the 3D $\mathcal{I}$- and $\mathcal{T}$-symmetric 3D TI given in SEqs. (3.48) and (3.49). (a) shows the 3D Brillouin zone with high symmetry points labeled. (b) shows the 3D bulk band structure of $[H(\mathbf{k})]$ in SEq. (3.48) with tight-binding parameters in SEq. (3.49). (c) shows the 2D band structure of a slab infinite along $\mathbf{a}_2 \parallel \hat{\mathbf{y}}$ and $\mathbf{a}_3 \parallel \hat{\mathbf{z}}$ while finite along $\mathbf{a}_1 \parallel \hat{\mathbf{x}}$ with open boundary condition and 41 unit cells. We see a twofold degenerate surface Dirac cone on each surface.

respectively. The 3D Brillouin zone, energy spectrum, and finite-sized slab spectrum for this model are shown in SFig. 4.

In SFig. 5(a), we show the k_x-directed P-Wilson loop eigenphases $\{\gamma_{1,j}(k_y, k_z)\}$ $(j = 1 \sim 2)$ of the two occupied bands as a function of k_y at different constant-k_z planes. At the $k_z = 0$ plane there is an odd helical winding of $\{\gamma_{1,j}(k_y, 0)\}$ as $k_y \to k_y + 2\pi$ while at the $k_z = \pi$ plane there is no helical winding of $\{\gamma_{1,j}(k_y, \pi)\}$ as $k_y \to k_y + 2\pi$. In SFig. 5(a), the helical winding at the $k_z = 0$ plane is protected by spinful $\mathcal{T}$ symmetry, while at $k_z = \pi$ we have a trivial winding of $\{\gamma_{1,j}(k_y, k_z)\}$ as $k_y \to k_y + 2\pi$. This means that the Hamiltonian [SEq. (3.48)] restricted to the $k_z = 0$ plane is topologically equivalent to a two-dimensional Hamiltonian describing a 2D strong TI, while the Hamiltonian [SEq. (3.48)] restricted to the $k_z = \pi$ plane is topologically equivalent to a two-dimensional Hamiltonian describing a trivial insulator. SFig. 5(a) then demonstrates that our lattice model is indeed a 3D TI.

We next numerically determine the locations of the gap closing points in the Ps_zP bands. Since our system has both $\mathcal{I}$ and $\mathcal{T}$ symmetry, we know from SN 2 B and SFig. 1 that we can define the lower spin bands as those states with $[s_{z,\text{reduced}}(\mathbf{k})]$ eigenvalue $\lambda^-_{n\mathbf{k}} < 0$, and similarly the upper spin bands can be defined as those states with $[s_{z,\text{reduced}}(\mathbf{k})]$ eigenvalue $\lambda^+_{n\mathbf{k}} > 0$. Spin gap closing points can only occur at points $\mathbf{k}_*$ such that $\lambda^+_{n\mathbf{k}_*} = \lambda^-_{n\mathbf{k}_*} = 0$. We can exploit this fact to efficiently find the spin-Weyl nodes numerically by searching for points $\mathbf{k}_*$ where $\det[s_{z,\text{reduced}}(\mathbf{k}_*)] = 0$ (which can only occur when the spin gap closes). We do this by applying the iterative numerical minimization algorithm to a grid of initial points in the BZ. In SFig. 5(b) we show the two obtained spin s_z gap closing points $\mathbf{k}_1 = -\mathbf{k}_2 = (0, 0.456\pi, -0.212\pi)$ related to each other by $\mathcal{T}$ and $\mathcal{I}$ symmetries.

We next compute the k_x-directed $P_\pm$-Wilson loop spectrum $\{\gamma^\pm_{1,j}(k_y, k_z)\}$ as a function of k_y for different constant-k_z planes, shown in SFig. 5(c,d). Since our model has only two occupied bands, there is only one band in each of the positive and negative Ps_zP eigenspace, and hence there is only one band $\{\gamma^\pm_{1,j}(k_y, k_z)\}$ $(j = 1$ only) for each of the $P_\pm$-Wilson loops. As long as $k_z \neq \pm 0.212\pi$, the s_z spin gap is open, such that the $P_\pm$-Wilson loop is well-defined. We first notice that in the $\mathcal{T}$-invariant $k_z = 0$ plane, the $\{\gamma^+_{1,j}(k_y, k_z)\}$ and $\{\gamma^-_{1,j}(k_y, k_z)\}$ as a function of k_y exhibit net winding numbers equal to $+1$ and -1, respectively. This implies that the occupied bands in the $k_z = 0$ plane have the partial Chern numbers $C^\pm_{\gamma_1} = \pm 1$ and the relative winding number (s_z spin Chern number) $C^s_{\gamma_1} = +2$ defined in SEq. (3.26). The Hamiltonian of our model restricted to the $k_z = 0$ plane is thus topologically equivalent to a model of a 2D strong topological insulator. Since the partial Chern numbers cannot change unless the spin gap closes, we expect that $C^\pm_{\gamma_1}(k_z) = \pm 1$ for all $|k_z| < 0.212\pi$. Indeed we see in SFig. 5(c,d) that $C^\pm_{\gamma_1}(k_z = 0.16\pi) = \pm 1$, consistent with our expectation. Thus, away from $k_z = 0$, the Hamiltonian of our model restricted to 2D planes with $|k_z| < 0.212\pi$ are topologically equivalent (in the sense that they can be deformed without closing an energy gap *or* a spin gap) to a model of a 2D magnetic insulator with partial Chern numbers $C^\pm_{\gamma_1} = \pm 1$.

On the other hand, in the $\mathcal{T}$-invariant $k_z = \pi$ plane, both $\{\gamma^+_{1,j}(k_y, k_z)\}$ and $\{\gamma^-_{1,j}(k_y, k_z)\}$ exhibit zero winding as a function of k_y, which implies that the occupied bands in the $k_z = \pi$ plane have vanishing partial Chern numbers. This indicates that the Hamiltonian of our model restricted to the $k_z = \pi$ plane is topologically equivalent to a model of a 2D trivial insulator. Going further, since the partial Chern numbers can be defined for all BZ planes in which the spin gap is open, away from $k_z = \pi$, the occupied bands of our model restricted to 2D BZ planes with $\pi \geq |k_z| > 0.212\pi$ are topologically equivalent (in the sense that they can be deformed without closing an energy gap *or* a spin gap) to those of a 2D magnetic insulator with partial Chern numbers $C^\pm_{\gamma_1} = 0$. We numerically verify this by computing the

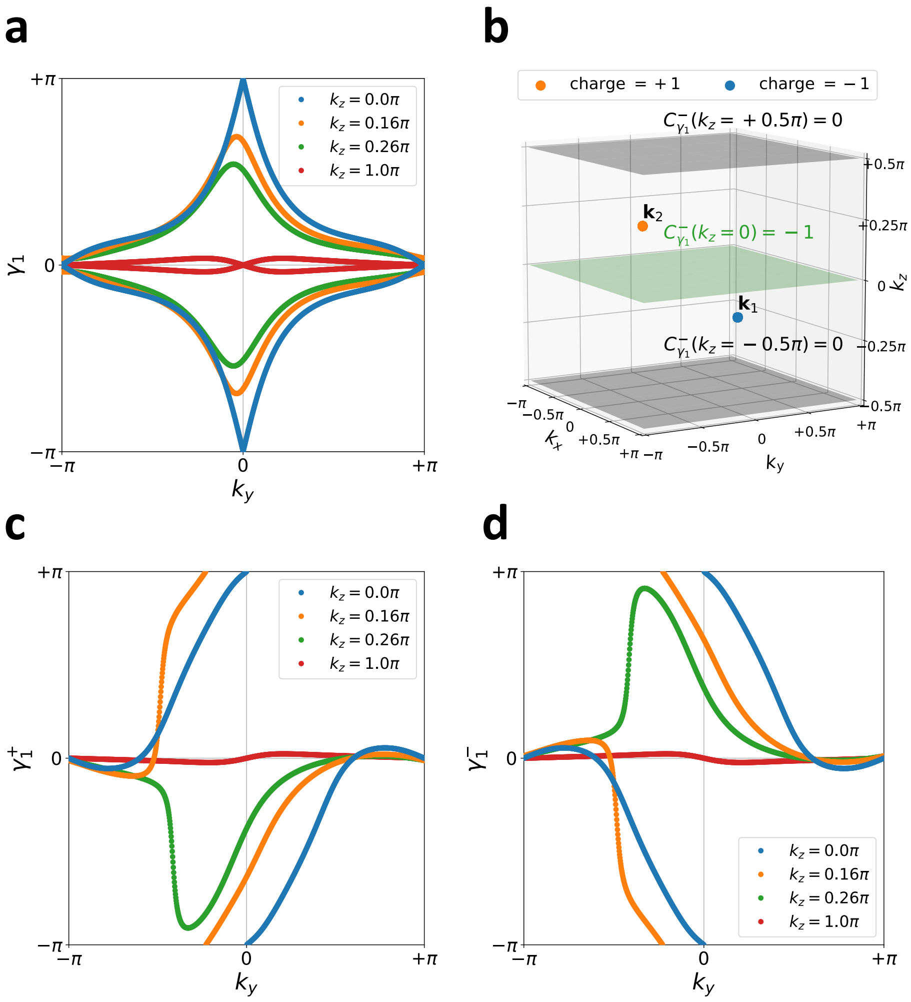

Supplementary Figure 5. Wilson loops for the 3D strong TI with both spinful time-reversal and inversion symmetry, with Hamiltonian given by SEqs. (3.48) and (3.49). (a) shows the k_x-directed P-Wilson loop eigenphases $\{\gamma_{1,j}(k_y, k_z)\}$ $(j = 1 \sim 2)$ as a function of k_y in constant-k_z plane. (b) shows the two Ps_zP gap closing points (spin-Weyl nodes) at $\mathbf{k}_1 = -\mathbf{k}_2 = (0, 0.456\pi, -0.212\pi)$ with their individual charges, namely the partial Berry flux, of the lower spin band indicated. The winding numbers $C^-_{\gamma_1}$ of the P_--Wilson loop spectra at constant-k_z planes separated by these spin-Weyl nodes will differ by the amount of the topological charges, as will be demonstrated in (d). (c)&(d) show the k_x-directed $P_\pm$-Wilson loop eigenphases $\{\gamma^\pm_{1,j=1}(k_y, k_z)\}$ as a function of k_y in constant-k_z plane. The projectors $[P_\pm(\mathbf{k})]$ are defined using s_z as the s operator in SEq. (3.5), see SN 3 B for further details. All of the k_x-directed P- and $P_\pm$-Wilson loop are computed by discretizing the path $\mathbf{k} + \mathbf{G} \leftarrow \mathbf{k}$ of the projector product in SEqs. (3.4) and (3.19) with 101 $\mathbf{k}$ points. The calculations detailed in this figure were performed using the freely available Python package NESTED_AND_SPIN_RESOLVED_WILSON_LOOP [97], which represents an extension of the PythTB open-source Python tight-binding package [171] that was implemented and utilized for the preparation of SRefs. [8, 15] and the present work.

$P_\pm$-Wilson loops at $k_z = 0.26\pi$ and $k_z = \pi$ [SFig. 5(c,d)], which exhibit zero winding, indicating that the spin bands carry zero partial Chern numbers.

Since the k_z coordinate of the spin s_z gap closing points are $\pm 0.212\pi$, we expect that both $\{\gamma^+_{1,j}(k_y, k_z)\}$ and $\{\gamma^-_{1,j}(k_y, k_z)\}$ will have a discontinuous change of winding when the constant-k_z plane passes through $k_z = \pm 0.212\pi$. As demonstrated in SFig. 5(c) [5(d)], in the $k_z = 0.16\pi$ plane, the k_x-directed $\{\gamma^+_{1,j}(k_y, k_z)\}$ $[\{\gamma^-_{1,j}(k_y, k_z)\}]$ has a net $+1$ $[-1]$ winding while in the $k_z = 0.26\pi$ plane it has zero winding. Since the partial Chern number $C^+_{\gamma_1}$ $[C^-_{\gamma_1}]$ changes by -1 $[+1]$ between planes just below and just above the spin gap closing point with $k_z = 0.212\pi$, we deduce that this spin gap closing point is a monopole source of partial Berry flux with charge -1 $[+1]$ for the upper [lower] spin

bands. We indicate this in SFig. 5(b). This justifies our identification of the spin gap closing points as spin-Weyl nodes. Consistent with our analysis in SN 2 E, 3 C and 3 H, we see that our 3D TI model has an odd number of spin-Weyl nodes in each half of the BZ.

F. 3D TIs With and Without Inversion Symmetry

In this section, we will examine the spin spectra and topology of 3D strong topological insulators with only time-reversal symmetry. As noted in SN 2 B, for systems with $\mathcal{T}$ but without $\mathcal{I}$ symmetry, we can divide the spin band structure into an equal number of lower and upper spin bands. For N_{occ} total occupied bands, we take the projector onto the upper spin bands $P_+(\mathbf{k})$ to project onto the $N_{\text{occ}}/2$ eigenstates of $[s_{\text{reduced}}(\mathbf{k})]$ with largest eigenvalues; we denote the reduced spin eigenvalues for these states as $\lambda^+_{n\mathbf{k}}$, where $n = 1 \ldots N_{\text{occ}}/2$. Similarly we take the projector onto the lower spin bands $P_-(\mathbf{k})$ to project onto the $N_{\text{occ}}/2$ eigenstates of $[s_{\text{reduced}}(\mathbf{k})]$ with smallest eigenvalues; we denote the reduced spin eigenvalues for these states as $\lambda^-_{n\mathbf{k}}$, where $n = 1 \ldots N_{\text{occ}}/2$. The spin gap is open provided $\lambda^+_{n\mathbf{k}} \neq \lambda^-_{m\mathbf{k}}$ for all m, n and $\mathbf{k}$.

Just as in SN 3 E, the spin gap with $\mathcal{T}$ symmetry will generically close at isolated points $\mathbf{k}_*$, at which for a given choice of n, we have that $\lambda^+_{n\mathbf{k}_*} = \lambda^-_{n\mathbf{k}_*}$. However, with only $\mathcal{T}$ symmetry, $\lambda^+_{n\mathbf{k}_*} = \lambda^-_{n\mathbf{k}_*} \neq 0$ generically at the spin gap closing points. Specifically, with only $\mathcal{T}$ symmetry, the spin gap for a 3D insulator will generically close at a set of spin-Weyl nodes, but those spin-Weyl nodes need not have vanishing PsP eigenvalues (*i.e.* the spin-Weyl nodes can appear at nonzero PsP eigenvalues in the spin spectrum). We also have from SN 2 B that if there exists a spin-Weyl node at $\mathbf{k}_*$ with $\lambda^+_{n\mathbf{k}_*} = \lambda^-_{n\mathbf{k}_*} = \lambda$, then by $\mathcal{T}$ symmetry, there will also be a spin-Weyl node at $-\mathbf{k}_*$ with $\lambda^+_{n-\mathbf{k}_*} = \lambda^-_{n-\mathbf{k}_*} = -\lambda$; this is illustrated in SFig. 6(d). As in the $\mathcal{I}$- and $\mathcal{T}$-symmetric case considered in SN 3 E, spin-Weyl nodes in systems with only $\mathcal{T}$ symmetry are also monopole sources of partial Chern numbers, even though they generically carry nonzero PsP eigenvalues. Specifically, if we consider the occupied bands in two BZ planes on either side of a spin-Weyl node in a noncentrosymmetric, $\mathcal{T}$-invariant insulator, then the partial Chern numbers $C^{\pm}_{\gamma_1}$ each change by ± 1 (or ∓ 1) as $\mathbf{k}$ crosses the spin-Weyl node.

We can now relate spin spectrum degeneracies to the strong topological index in systems with only time-reversal symmetry. Consider a system with a spin band structure that has an odd number of spin-Weyl points in half the BZ, none of which occur on a $\mathcal{T}$-invariant plane. We then have that the partial Chern numbers on the two $\mathcal{T}$-invariant planes bounding the half of the BZ must differ by 1 (or more generally an odd integer). On $\mathcal{T}$-invariant planes, 2D $\mathbb{Z}_2$ Kane-Mele invariant ν_{2d} is given by the parity of the partial Chern numbers as shown in the argument following SEq. (3.33). This lets us deduce that ν_{2d} differs between these two $\mathcal{T}$-invariant planes. This implies immediately that the system is a strong topological insulator as defined in SRef. [141]. It is crucial to recall in this argument that while the 2D $\mathbb{Z}_2$ invariant is only defined on $\mathcal{T}$-invariant planes, the partial Chern numbers are defined at all planes in the BZ in which the spin gap is open. In particular, the value of ν_{2d} for the occupied bands on a $\mathcal{T}$-invariant plane M fixes parity of half the spin Chern number $C^s_{\gamma_1}/2$ [defined in SEq. (3.26)] for the occupied bands on any non-$\mathcal{T}$-invariant plane that can be reached by deforming M without crossing a spin-Weyl node. Thus, we deduce that for a gapped Hamiltonian H with spinful $\mathcal{T}$ symmetry, if the spectrum of PsP has a number of Weyl nodes $n_w \mod 4 = 2$, then the system must be a 3D strong topological insulator. In particular, if there is a spin-Weyl node at $\mathbf{k}$ with PsP eigenvalue λ, by time-reversal symmetry there will be another spin-Weyl node at $-\mathbf{k}$ with PsP eigenvalue $-\lambda$. Crucially, the Berry flux around a spin-Weyl node reverses sign under $\mathcal{T}$ symmetry: two spin-Weyl nodes related by $\mathcal{T}$ have opposite partial Chern numbers (chiral charges). This might seem counter-intuitive, as we know that Weyl nodes in the energy spectrum that are related by $\mathcal{T}$ symmetry carry the same Berry flux. However, recall that for spin bands, time-reversal not only flips the momentum but also flips the sign of the spin. Therefore, under $\mathcal{T}$ the upper spin band of a spin-Weyl node at momentum $\mathbf{k}$ maps to the lower spin band of the image spin-Weyl node at $-\mathbf{k}$. Since the upper and lower bands of a Weyl node carry opposite Chern numbers *provided that the occupied energy bands are separated from the unoccupied energy bands by a finite energy gap*, we deduce that the spin-Weyl nodes that are related to each other by $\mathcal{T}$ symmetry will carry opposite partial Berry flux. On the other hand, for a system with both $\mathcal{I}$ and $\mathcal{T}$ symmetries (see SN 3 E), the spin-Weyl nodes with $\lambda = 0$ at $\mathbf{k}$ and $-\mathbf{k}$ are related by either $\mathcal{T}$ or $\mathcal{I}$. Importantly, spin-Weyl nodes related by $\mathcal{I}$ symmetry will also carry opposite Berry flux. This is because $\mathcal{I}$ acts on the PsP eigenstates in the same way as the energy eigenstates, and $\mathcal{I}$ does not flip the sign of the spin. Therefore, each of spin-Weyl nodes carries nonzero partial Berry flux (even though the system has both $\mathcal{I}$ and $\mathcal{T}$ symmetry) while Weyl nodes in the energy spectrum are forbidden in systems with both $\mathcal{I}$ and $\mathcal{T}$ symmetry [173]. As a corollary, this also constrains the perturbations to the spin band structure that can occur without closing the spectral gap of the Hamiltonian H. In particular, note that in order to have, or induce, a total number of spin-Weyl nodes $n_w \mod 4 = 2$, we must invert spin bands at a TRIM point (a spin band inversion at a generic point would create two pairs of Weyl points related by $\mathcal{T}$-symmetry). Since this spin band inversion would change the strong $\mathbb{Z}_2$ invariant of the occupied states in a $\mathcal{T}$-invariant plane, it cannot occur without closing the gap in the Hamiltonian.

This is consistent with the analysis of SRef. [169].

Let us now numerically verify these points. We will consider a model of a 3D TI with only $\mathcal{T}$ symmetry and demonstrate the application of the $P_{\pm}$-Wilson loop formalism previously described in SN 3 B to diagnose a 3D $\mathcal{T}$-invariant TI without inversion symmetry. To begin, we take the model of a 3D TI described in SEq. (3.48) with parameters in SEq. (3.49) and add additional terms

$$H_{\mathcal{I}\text{-breaking}}(\mathbf{k}) = \sum_{i,j=1}^{3} f_{0ij} \sin k_j \tau_0 \sigma_i, \;\; f_{0ij} \in \mathbb{R}, \tag{3.52}$$

that break $\mathcal{I}$ while preserving $\mathcal{T}$ symmetry:

$$\mathcal{I} : (\tau_z\sigma_0) f_{0ij} \sin k_j \tau_0 \sigma_i (\tau_z \sigma_0) = f_{0ij} \sin k_j \tau_0 \sigma_i \neq f_{0ij} \sin(-k_j) \tau_0 \sigma_i, \tag{3.53}$$

$$\mathcal{T} : (\tau_0\sigma_y) f_{0ij} \sin k_j \tau_0 (\sigma_i)^* (\tau_0 \sigma_y) = f_{0ij} \sin(-k_j) \tau_0 \sigma_i. \tag{3.54}$$

In SFig. 6(a,b) we show the 3D bulk band structures with $f_{0ij} = 0.0$ and $f_{0ij} = 1.0$ for all $i, j = 1 \sim 3$, respectively. As we can see in SFig. 6(b), since the $\mathcal{I}$ symmetry is broken, the energy bands are no longer doubly degenerate at generic (non-time-reversal-invariant) $\mathbf{k}$ points. Since there is no bulk gap closing when we turn on nonzero values of f_{0ij}, the two valence bands of SFig. 6(b) still describe a 3D TI with a nontrivial strong $\mathbb{Z}_2$ invariant $\nu_{2d} = 1$. This can be seen by computing the band structure of a 2D slab finite along z, which is shown in SFig. 6(c) exhibits one surface Dirac cone at the surface Γ point of each surface. As mentioned above, if we denote $\{s_n(\mathbf{k})\}$ as the set of eigenvalues of the reduced spin matrix [SEq. (3.5)], $\mathcal{T}$ symmetry alone only constrains $\{s_n(\mathbf{k})\} = \{-s_n(-\mathbf{k})\}$. Therefore, generically the spin-Weyl nodes do not appear at zero PsP eigenvalues. To demonstrate this, we obtain the Ps_zP band structure of the two valence bands of SFig. 6(b), which contains two spin-Weyl nodes at momenta $\pm\mathbf{k}_*$ with *nonzero* Ps_zP *eigenvalues*, as shown in SFig. 6(d). In SFig. 6(d), the spin-resolved projectors $P_+(\mathbf{k})$ and $P_-(\mathbf{k})$ are the projectors onto the upper (orange) and lower (blue) spin bands. We then compute the k_x-directed P- and $P_{\pm}$-Wilson loop spectra as a function of k_y at various $k_z =$ constant planes. As shown in SFig. 6(e), at $k_z = 0$ and $k_z = \pi$ we have helical winding and trivial winding, respectively. This indicates that the two valence bands still describe a 3D TI. In addition, in SFig. 6(f,g) we have spectral flows with winding numbers ± 1 and 0 in the $P_{\pm}$-Wilson loop eigenphases at $k_z = 0$ and $k_z = \pi$ planes, respectively. This again demonstrates that the Hamiltonian restricted to $k_z = 0$ is topologically equivalent to a 2D $\mathcal{T}$-invariant TI. Similarly, the Hamiltonian restricted to the $k_z = \pi$ plane is topologically equivalent to a trivial 2D insulator. In particular, there is a discontinuous change of the spectral flow of the $P_{\pm}$-Wilson loops between $k_z < (\mathbf{k}_*)_z$ and $k_z > (\mathbf{k}_*)_z$, as shown in SFig. 6(f,g). This is a clear indication that the spin-Weyl nodes at $\pm\mathbf{k}_*$ play the role of *partial Chern number monopoles*.

The above discussion reiterates the central idea first recognized in SRef. [6] that to diagnose a helical topological phase, one could divide the occupied space into two parts that are related to each other by the $\mathcal{T}$ operation. Once we have constructed $P_{\pm}(\mathbf{k})$ as a smooth function of $\mathbf{k}$ (except at the spin-Weyl nodes) without a discontinuous jump of rank$[P_{\pm}(\mathbf{k})]$, we can use one of the $P_{\pm}(\mathbf{k})$ to diagnose the helical topological phase by examining its Wilson loop spectrum. In SN 4 we will extend this perspective to demonstrate the existence of previously unrecognized quantized spin-resolved invariants in helical HOTIs.

G. Spin-Stable Topology in a 2D Fragile Topological Insulator

In this section, we will demonstrate that in a 2D fragile TI there can exist *spin-stable topology*, which is robust to perturbations provided that both the *energy* and *spin* gaps remain open. We will argue that such spin-stable topological phases can exhibit robust responses to external fields and fluxes. Thus, we will show that even though 2D fragile topological phases have the same boundary signatures as obstructed atomic insulators [8, 15, 31, 174–177], they can differ in their bulk signatures due to spin-stable topology.

Without the addition of appropriately chosen trivial bands, the occupied bands of a fragile TI cannot form exponentially localized Wannier functions that respect all of the crystal symmetries. For example, let us consider the 2D fragile topological model introduced in SRef. [8] as a time-reversal invariant generalization of the 2D quadrupole insulator [13, 14]. This model has a square lattice with Bloch Hamiltonian

$$\begin{aligned}[H_F(\mathbf{k})] = {} & t_1[\cos(k_x) + \cos(k_y)]\tau_z\sigma_0 \\ & + t_2[\cos(k_x) - \cos(k_y)]\tau_x\sigma_0 + v_m\tau_z\sigma_0 \\ & + t_{PH}[\cos(k_x) + \cos(k_y)]\tau_0\sigma_0 \\ & + v_s \sin(k_x)\sin(k_y)\tau_y\sigma_z \\ & + v_{M_z}[\sin(k_x)\tau_z\sigma_y - \sin(k_y)\tau_z\sigma_x], \end{aligned} \tag{3.55}$$

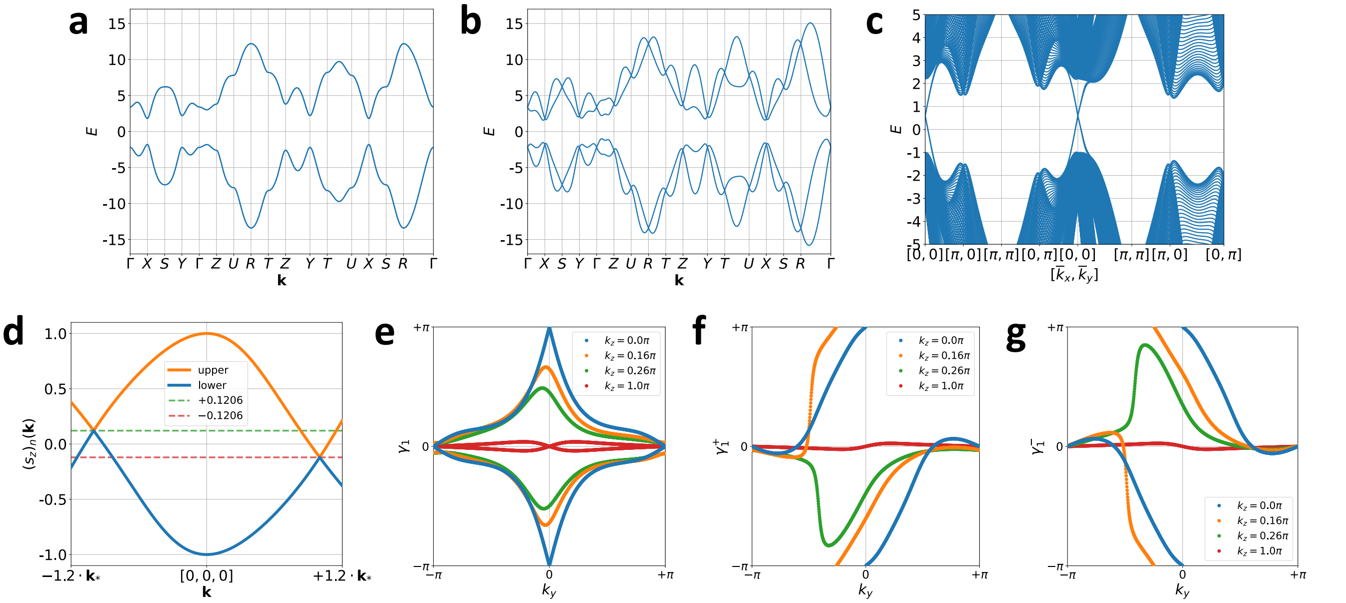

Supplementary Figure 6. Spectrum and Wilson loops for an inversion-breaking 3D TI. (a) shows the 3D bulk energy band structure of a 3D TI described by SEq. (3.48) with parameters in SEq. (3.49), where the labels of the high-symmetry $\mathbf{k}$ points can be found in SFig. 4(a). The energy bands are doubly degenerate at all $\mathbf{k}$ points because of the coexistence of inversion ($\mathcal{I}$) and time-reversal ($\mathcal{T}$) symmetries. (b) shows the 3D bulk energy band structure of a 3D TI described by SEq. (3.48) with additional inversion-breaking terms in SEq. (3.52). The energy bands are no longer doubly degenerate at generic $\mathbf{k}$ points except for TRIMs. (c) shows the 2D energy band structure of a slab finite along z with 41 unit cells for the inversion-breaking 3D TI with the same model parameters in (b). We can see that there are 2D, twofold degenerate surface Dirac cones with linear dispersion at the $\bar{\Gamma}$ point ($\bar{k}_x = \bar{k}_y = 0$) of the surface BZ. (d) shows the eigenvalues $(s_z)_n(\mathbf{k})$ (n = band index) of the reduced s_z matrix from SEq. (3.5) as a function of the crystal momenta $\mathbf{k}$, which we call the Ps_zP band structure, for the two valence bands in (b). The Ps_zP band structure is plotted from $-1.2\mathbf{k}_*$ to $+1.2\mathbf{k}_*$ along a straight line. And $\pm\mathbf{k}_* \approx \pm(0, 0.46\pi, -0.21\pi)$ are the positions of the spin-Weyl nodes which have *nonzero* Ps_zP *eigenvalues* ∓ 0.1206. (e) shows the k_x-directed P-Wilson loop eigenphases of the two lowest occupied bands of the inversion-breaking 3D TI plotted as a function of k_y at different k_z = constant planes. (f) & (g) show the k_x-directed $P_\pm$-Wilson loop eigenphases of the two lowest occupied bands of the inversion-breaking 3D TI plotted as a function of k_y at different k_z = constant planes. We can see that there is a discontinuous change of the winding numbers of the spectral flow at the planes with $k_z < (\mathbf{k}_*)_z$ and $k_z > (\mathbf{k}_*)_z$. As an example, we can see that the spectral flow has a $+1$ [0] winding number at the $k_z = 0.16\pi$ [$k_z = 0.26\pi$] plane in (f). And the spectral flow has a -1 [0] winding number at the $k_z = 0.16\pi$ [$k_z = 0.26\pi$] plane in (g). The calculations detailed in this figure were performed using the freely available Python package NESTED_AND_SPIN_RESOLVED_WILSON_LOOP [97], which represents an extension of the PythTB open-source Python tight-binding package [171] that was implemented and utilized for the preparation of SRefs. [8, 15] and the present work.

where Pauli matrices τ_i (σ_i) denotes the s and d orbital (spin) degrees of freedom that are placed at the $1a$ ($(x, y) = (0, 0)$) Wyckoff position of the primitive square unit cell. Both τ_0 and σ_0 are 2×2 identity matrices. t_1 and t_2 are the nearest-neighbor hopping energies between the same and different orbitals, v_m induces an on-site orbital energy splitting, t_{PH} is a spin- and orbital-independent nearest-neighbor hopping that breaks the particle-hole symmetry of the energy spectrum, and v_s represents a next-nearest-neighbor spin-orbit coupling (SOC). In addition, v_{M_z} is an SOC term breaking the 3D layer group [178] mirror reflection M_z (represented as σ_z) and inversion $\mathcal{I}$ (represented as identity) symmetries.

The Bloch Hamiltonian [SEq. (3.55)] respects the symmetries of wallpaper group $p4m1'$, which is generated by mirror reflection M_x, the four-fold rotation C_{4z}, time-reversal $\mathcal{T}$, and 2D lattice translations (for further information regarding wallpaper groups and their relationship to topological phases, see SRefs. [8, 107, 178–181]). The symmetries are represented by

$$[M_x][H_F(\mathbf{k})][M_x]^{-1} = \sigma_x[H_F(\mathbf{k})]\sigma_x = [H_F(M_x\mathbf{k})] \tag{3.56}$$

$$[C_{4z}][H_F(\mathbf{k})][C_{4z}]^{-1} = \tau_z e^{-i\pi/4\sigma_z}[H_F(\mathbf{k})]\tau_z e^{i\pi/4\sigma_z} = [H_F(C_{4z}\mathbf{k})] \tag{3.57}$$

$$[\mathcal{T}][H_F(\mathbf{k})][T]^{-1} = (i\sigma_y)[H_F(\mathbf{k})]^*(-i\sigma_y) = [H_F(-\mathbf{k})] \tag{3.58}$$

The 2D bulk energy bands of SEq. (3.55) correspond to the images of the projectors P_2 and Q_2 shown in SFig. 7.

This model has no gapless edge states in the energy gaps between P_2 and Q_2 when placed in a ribbon geometry, but has four Kramers pairs of corner modes when placed on a finite-sized square [8]. When the bulk is half-filled, the corner mode filling is generically 3/4.

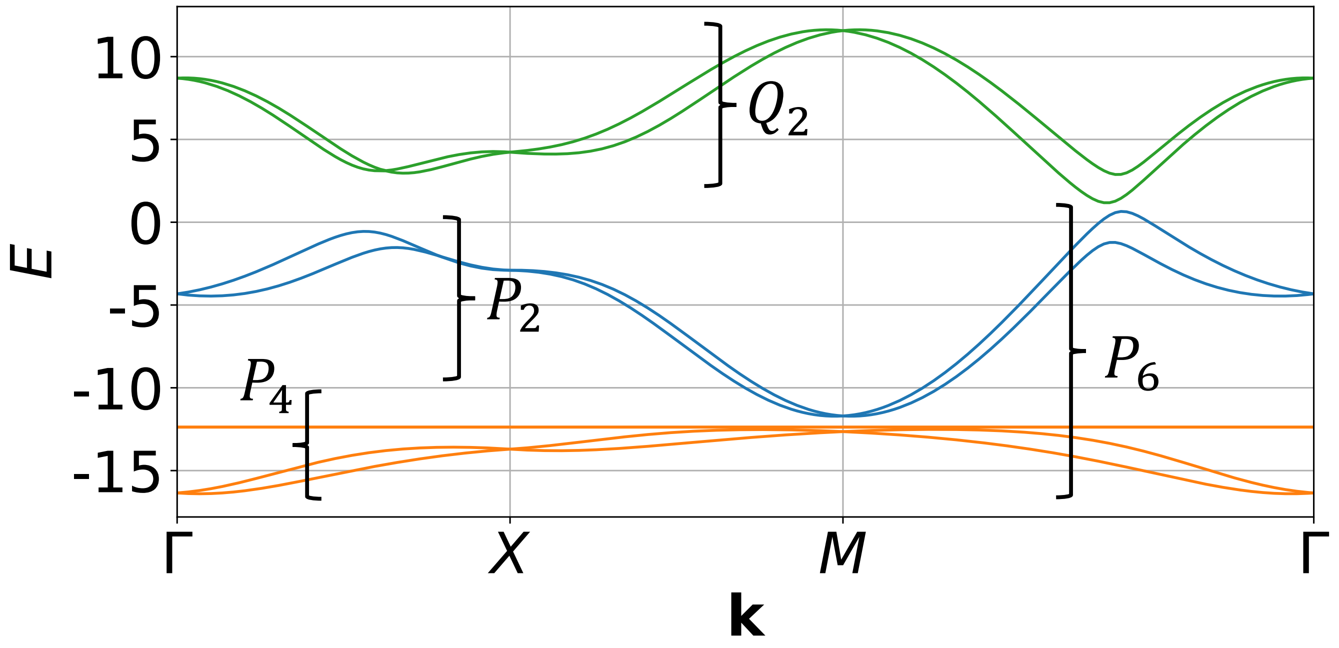

Supplementary Figure 7. Energy bands of a 2D fragile TI in SEq. (3.55) on a square lattice where P_2 and Q_2 project respectively onto the two valence and two conduction bands. The high-symmetry $\mathbf{k}$ points Γ, X and M correspond to $(k_x, k_y) = (0,0)$, $(\pi, 0)$, and (π,π), respectively. P_4 is the projector onto four additional energy bands induced from trivial atomic orbitals that are coupled to the 2D fragile TI [SEq. (3.55)] through SEq. (3.59) in a way that respects the symmetries of $p4m1'$ wallpaper group (see also SRef. [8], where this model was originally introduced). The energy bands are computed from Bloch Hamiltonian $[H_F(\mathbf{k})] + [V_C(\mathbf{k})]$ in SEqs. (3.55) and (3.59) with tight-binding parameters $t_1 = 5.0$, $t_2 = 1.5$, $v_m = -1.5$, $t_{PH} = 0.1$, $v_s = 1.3$, $v_{M_z} = 0.4$, $v_\mu = 8.25 \times v_m$, $v_C = 4.0$, and $v_{CS} = 0.45 \times v_C$. The numbers of energy bands in the image of Q_2, P_2, and P_4 are 2, 2, and 4, respectively.

The energy bands in the image of the projector P_2 have fragile topology [150–152] that can be diagnosed from symmetry indicators and the Wilson loop spectrum [8]. Shown in SFig. 8(a) are the k_y-directed P-Wilson loop eigenphases $\{\gamma_{1,j}(k_x)\}$ as a function of k_x computed in the P_2 occupied space where j is the P-Wannier band index. In particular, this P-Wilson loop spectrum has several two-band crossings at $\gamma_1 = \pi$ and generic momenta demonstrated in SFig. 8(b), that are protected by C_{2z} and $\mathcal{T}$ symmetries [8, 87, 153, 182, 183]. To demonstrate that the two-band crossings in SFig. 8(b) arise from bulk fragile topology, we introduce *four* additional tight-binding basis orbitals to the Hilbert space of the system consisting of spinful s orbitals at the $2c$ Wyckoff positions ($(x, y) = (1/2, 0)$ and $(0, 1/2)$) that are coupled to $[H_F(\mathbf{k})]$ in SEq. (3.55) through

$$\begin{aligned}[V_C(\mathbf{k})] &= v_\mu [P_{\mu^s}] \\ &+ v_C \left[\mu_{1,x} \cos\left(\frac{k_x}{2}\right) + \mu_{2,x} \cos\left(\frac{k_y}{2}\right)\right] \\ &+ v_{CS} \left[-\mu_{1,x}\sigma_y \sin\left(\frac{k_x}{2}\right) + \mu_{2,x}\sigma_x \sin\left(\frac{k_y}{2}\right)\right]. \end{aligned} \tag{3.59}$$

$[P_{\mu^s}]$ is the projection matrix onto the spinful s orbitals at $2c$ Wyckoff positions. $\mu_{1,x}$ $[\mu_{2,x}]$ implements the hopping matrix with nonzero coefficients being 1 that connects the spinful d orbitals at $1a$ and s orbitals at $(x, y) = (1/2, 0)$ $[(x, y) = (0, 1/2)]$. v_μ denotes the on-site energy (or effectively the chemical potential, up to a sign) of the spinful s orbitals at $2c$. v_C (v_{CS}) represents the (spin-orbit) coupling between the spinful d orbitals at $1a$ and s orbitals at $2c$. Notice that both of v_C and v_{CS} contain inter- and intra-cell hopping [8]. Crucially, the symmetry group of the combined Hamiltonian $[H(\mathbf{k})] + [V_C(\mathbf{k})]$ remains the wallpaper group $p4m1'$, such that changes to the spectral flow of the Wilson loop cannot be attributed to symmetry breaking.

The additional four bands whose coupling to the original Hamiltonian [SEq. (3.55)] is described by $[V_C(\mathbf{k})]$ [SEq. (3.59)] lie in the image of the projector denoted as P_4 in SFig. 7. The bands in the image of P_4 carry an elementary band representation of $p4m1'$ induced from spinful s orbitals at the $2c$ Wyckoff position. As analyzed in SRef. [8], although the bands in the image of P_2 are fragile topological, we can include the trivial bands in the image of P_4 and consider the combined projection operator $P_2 \oplus P_4 = P_6$. And the straight P_6-Wilson loop does not wind for any choice of direction in the 2D BZ. This is demonstrated in SFig. 10(b) where the two-band crossings at $\gamma_1 = \pi$ in SFig. 8(b) are removed, and the six-band Wilson loop spectrum shown in SFig. 10(a) form two groups of bands that are spectrally separated from each other–one group consists of two bands around $\gamma_1 = \pi$ mod 2π and the other group consists of four bands centered around $\gamma_1 = 0$. We have hence demonstrated that the two-band crossings [see SFig. 8(b)] of the P-Wilson loop spectrum in occupied space P_2 can be trivialized by adding trivial bands [see SFig. 10(b)], confirming the previous calculations in SRef. [8].

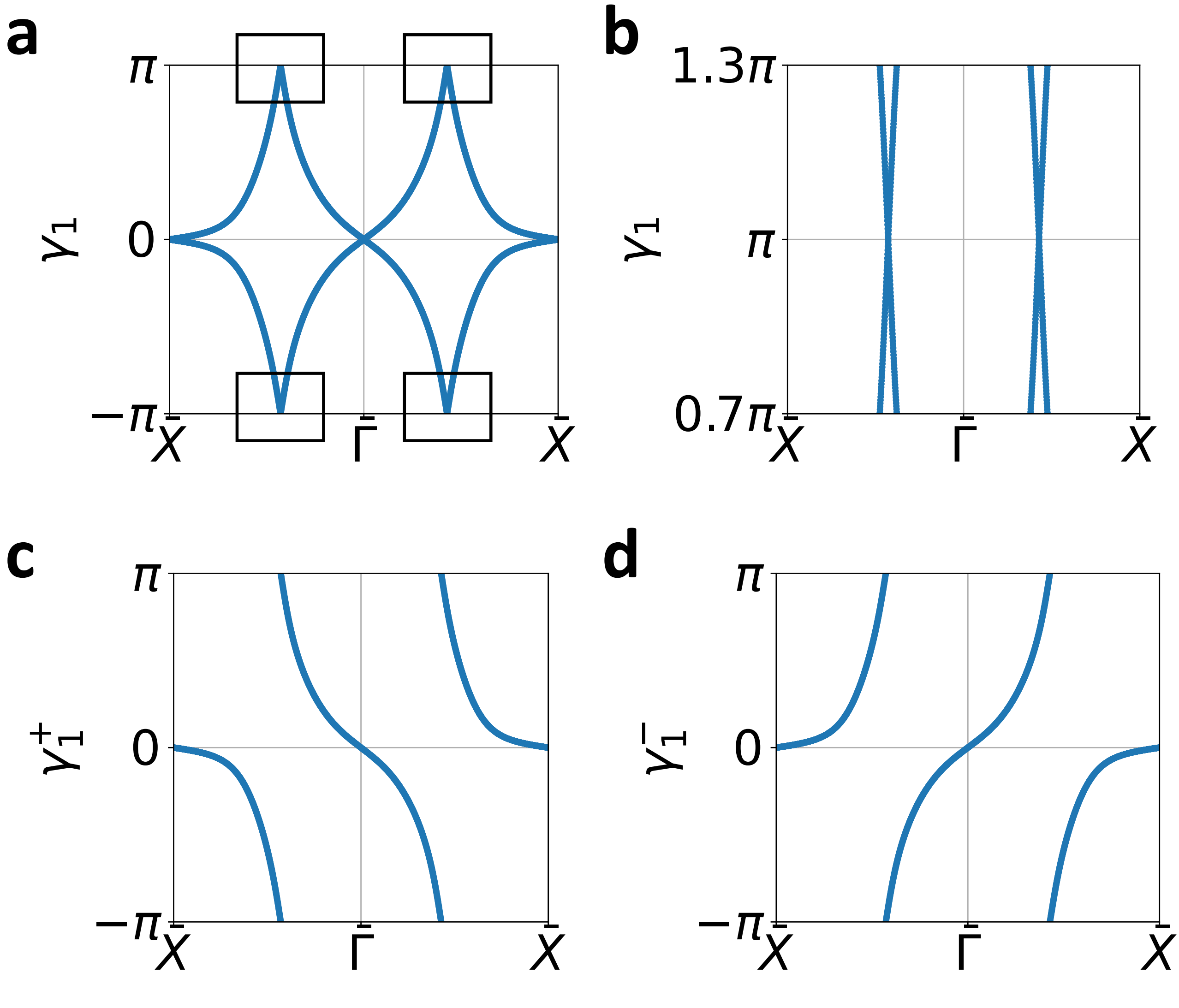

Supplementary Figure 8. k_y-directed Wilson loop eigenphases as a function of k_x in the (a) occupied space P_2 of SFig. 7, (c) positive (+), and (d) negative (−) eigenspace of $P_2 s_z P_2$ where s_z is the z-component of the spin vector $\mathbf{s}$, namely $s_z = \hat{\mathbf{z}} \cdot \mathbf{s}$. (b) is an enlarged view of the region in (a) around $\gamma_1 = \pi$, demonstrating the two-band crossings protected by C_{2z} and $\mathcal{T}$. The numbers of bands in (a), (c), and (d) are 2, 1, and 1, respectively. The calculations detailed in this figure were performed using the freely available Python package NESTED_AND_SPIN_RESOLVED_WILSON_LOOP [97], which represents an extension of the PythTB open-source Python tight-binding package [171] that was implemented and utilized for the preparation of SRefs. [8, 15] and the present work.

Although this system has a fragile topology in the occupied space P_2, we will now demonstrate that it also carries

a *nontrivial spin-stable topology*. We can spin-resolve the occupied space according to its $P_2 s_z P_2$ eigenvalues. As shown in SFig. 8(c,d), the k_y-directed $P_\pm$-Wilson loop eigenphases $\{\gamma^{\pm}_{1,j}(k_x)\}$ have net ∓ 2 winding as $k_x \to k_x + 2\pi$. From the winding numbers in SFig. 8(c,d) we can determine the partial Chern numbers of the occupied space P_2, using the sign convention introduced in SEq. (3.24). To be precise, the partial Chern numbers $C^{\pm}_{\gamma_1}$ in this case are determined by the negative winding numbers of the k_y-directed $P_\pm$-Wilson loop eigenphases as $k_x \to k_x + 2\pi$. This means that the partial Chern numbers of the $\pm$ eigenspace of $P_2 s_z P_2$ are $C^{\pm}_{\gamma_1} = \pm 2$, such that the *spin Chern number* [SEq. (3.26)] is given by $C^s_{\gamma_1} = +4$. As long as both the energy and $P_2 s_z P_2$ gaps remain open, this $C^s_{\gamma_1} = +4$ is robust [1] and can have several physical consequences when we apply external electromagnetic fields.

We now discuss how this spin-stable topology with $|C^s_{\gamma_1}| = 4$ manifests in the response of the system to external electromagnetic fields. We begin with the limit where $v_{M_z} = 0$ [see SEq. (3.55)]. In this case, our model in SEq. (3.55) retains mirror reflection M_z symmetry and also s_z-conservation. Hence in this limit the Hamiltonian characterizes a stable mirror topological crystalline insulator (TCI) and quantum spin Hall phase with mirror Chern number 2 and $|C^s_{\gamma_1}| = 4$ [140, 156]. This implies the presence of two pairs of fully-s_z-polarized helical edge states [8]. Here, "fully-s_z-polarized" means that the energy eigenstates are also eigenstates of s_z. A schematic depiction of the helical states at one edge is shown in SFig. 9(b) if we regard Φ in the x-axis as the crystal momentum parallel to the edge. This will lead to a quantized spin Hall conductivity $|\sigma^s_H| = |C^s_{\gamma_1}| \times |e|/4\pi = |e|/\pi$ when an external electric field is applied parallel to this 2D system [7], consistent with SEq. (3.27).

In addition to an in-plane electric field, we may also adiabatically thread a $U(1)$ magnetic flux $\Phi(t)$ as a function of time through the 2D system to probe the nontrivial bulk topology, see the setup as schematically depicted in SFig. 9(a). When $\Phi = 0$ and $\Phi = \pi$, the system has time-reversal symmetry while time-reversal is broken at generic values of $\Phi \neq 0$ and $\Phi \neq \pi$. The corresponding energy spectrum of the 2D sample as a function of Φ when s_z is conserved is shown in SFig. 9(b). Due to $|C^s_{\gamma_1}| = 4$, there are four gap-crossing modes, corresponding to single-particle bound states around the magnetic flux. Two of the modes have positive slopes and the other two have negative slopes. Modes with opposite slopes will have opposite s_z eigenvalues as a consequence of time-reversal symmetry. Note that although time-reversal symmetry is broken when the $U(1)$ magnetic flux $\Phi \neq 0$ and $\Phi \neq \pi$, for a model with s_z conservation when $\Phi = 0$, the Hamiltonian continues to commute with the s_z operator when $\Phi \neq 0$. Therefore, all the energy eigenstates in SFig. 9(b) are eigenstates of s_z.

Importantly, the crossings between the bands at generic Φ indicated by the red arrows in SFig. 9(b) are protected by s_z conservation, and the crossings at $\Phi = \pi$ indicated by black arrows are protected by $\mathcal{T}$. As we gradually increase the flux $\Phi(t)$, there will be a tangential electric field induced along the azimuthal direction around the flux $\Phi(t)$. Since we have s_z conservation, $|C^s_{\gamma_1}| = 4$ indicates that the magnetic flux induces radial currents of spin-up and spin-down electrons with same magnitudes but opposite signs. Applying the same computation performed in SRef. [184] at $\Phi = \pi$, the many-body ground state in the vicinity of the inserted flux may be described as a spinon bound state with total electric charge $Q = 0$ and total spin $|S_z| = 2(\hbar/2)$ [79, 185–194]. Note that the accumulated spin in the vicinity of the flux (measured in the standard units of $\hbar/2$) is equal to *half* the spin Chern number, consistent with the fact that $\Phi = \pi$ corresponds to half of a flux quantum. We also note that, correspondingly, if we were to instead insert a (fictitious or pseudo-) magnetic field that takes opposite signs in opposite spin sectors (*i.e.* a spin flux), the many-body ground state in the vicinity of the flux would instead be a chargeon with zero total spin and charge $|Q| = 2$ [184] (measured in the units of electron charge e).

This can also be understood as follows. We start from the ground state with all states in the valence continuum filled, and we fix the electron number so that the infinite system has total charge $Q = 0$ (including the underlying positive ions). If we now increase the flux Φ from 0 to π, two states will appear at the mid-gap through the two gap-crossing modes with positive slopes. Notice that these two states will have s_z eigenvalues both equal to $+\hbar/2$ or $-\hbar/2$, depending on the sign of $C^s_{\gamma_1}$ (recall that we assume $|C^s_{\gamma_1}| = 4$). At $\Phi = \pi$, one state of the doubly degenerate Kramers pair at higher mid-gap energy will be filled, and there will be another filled state of the doubly degenerate Kramers pair at lower mid-gap energy. And both of the filled states have the same s_z eigenvalues, which implies that the total spin of these two filled states will be $|S_z| = \hbar$. Due to the time-reversal symmetry at $\Phi = \pi$, the valence continuum will have a total spin equal to zero. Therefore, the spinon bound state around the magnetic flux when $\Phi = \pi$ will have total electric charge $Q = 0$ and total spin $|S_z| = \hbar$. We then conclude that when s_z is conserved, we expect to see quantized spin response. In response to an external electric field parallel to the 2D system we have quantized spin Hall conductivity $|\sigma^s_H| = |e|/\pi$ [SEq. (3.27)]. By threading a $U(1)$ magnetic flux Φ we will obtain a spinon bound state around the flux with total electric charge $Q = 0$ and total spin $|S_z| = \hbar$ when $\Phi = \pi$. We can then ask how this response effect changes when s_z is no longer conserved.

When s_z is not conserved, which in our 2D fragile topological insulator model [SEq. (3.55)] occurs when $v_{M_z} \neq 0$, the metallic edge states will in general be gapped due to the hybridization between fully-s_z-polarized edge states with opposite s_z eigenvalues and opposite Fermi velocities. For example, a schematic 1D band structure is shown in SFig. 9(c) if we regard the x-axis as the momentum parallel to the edge. In such a case, the entire system is an insulator in a ribbon geometry, including both the bulk and edge. In other words, when an external electric field is applied

parallel to the 2D system, there will be no charge flows that could simultaneously carry spin currents. Although there can be other mechanisms [108], for example the local spin flipping process, that can induce spin currents without charge currents, we expect that the spin Hall conductivity $|\sigma^s_H|$ will no longer be quantized, as discussed in SN 3 C. Note that if the modes have sufficiently large velocities (slopes $|\frac{dE}{d\mathbf{k}}|$ in the energy-momentum dispersion relation), then it is possible that there are no crossings between bands with positive slope and bands with negative slope within the bulk gap. In this case the spectrum would be insensitive to small nonzero values of v_{M_z}. We do not consider this case here, as it can always be deformed to the situation in SFig. 9(c) without closing an energy gap or a spin gap.

On the other hand, the spin-stable topology may still be explored by threading a $U(1)$ magnetic flux Φ through the system [see SFig. 9(a)]. As above, we consider the case where the velocity (slope $|\frac{dE}{d\Phi}|$ of the energy-Φ relation) of modes is low enough that there are crossings within the bulk gap as shown in SFig. 9(b). Since s_z is not conserved, the crossing between the counterpropagating modes indicated by the red arrows in SFig. 9(b) will in general be gapped, with a gap size proportional to $|v_{M_z}|$ [see SFig. 9(c)]. However, the doubly-degenerate localized states around the $U(1)$ flux at $\Phi = \pi$ indicated by the black arrows in SFig. 9(b) will still be doubly-degenerate, as indicated by the black arrows in SFig. 9(c). This is because when $\Phi = \pi$, the system has time-reversal symmetry and the doubly-degenerate states at $\Phi = \pi$ is protected due to Kramers theorem. If $|v_{M_z}|$ is small enough, we expect that the doubly-degenerate states, both at higher and lower energies, will consist of one state with a z-component spin angular momentum $\langle s_z \rangle \lesssim \hbar/2$ and another state with $\langle s_z \rangle \gtrsim -\hbar/2$.

Let us now consider threading a time-dependent flux $\Phi(t) = 2\pi t/T$. The system is now described by three energy scales (with $\hbar = 1$):

1. Δ, the bulk energy gap,

2. $|v_{M_z}|$, strength of s_z-conservation-breaking, which by our analysis in SN 2 C controls the size of the spin gap,

3. $\dot{\Phi} = d\Phi/dt = 2\pi/T$, the rate of flux insertion.

In the following discussion, we will assume that $|v_{M_z}| \ll \Delta$ and $\dot{\Phi} \ll \Delta$ such that the flux insertion analysis can be carried out using SFig. 9(c) where the bulk energy gap remains open, and the value of the bulk energy gap with $v_{M_z} \neq 0$ is close to its value with $v_{M_z} = 0$. We will start from the ground state with all states in the valence continuum filled at $\Phi = 0$, and we fix the electron number so that the infinite system has total charge $Q = 0$ (including the underlying positive ions). If $\dot{\Phi} = 2\pi/T \ll |v_{M_z}|$, when $t = T/2$ such that $\Phi = \pi$, we would have both the states in the doubly degenerate Kramers pair at the lower mid-gap energy filled. Since $\Phi = \pi$ preserves time-reversal symmetry, the filled valence continuum will have total spin equal to zero. Together with the filled doubly degenerate states, which also have total spin equal to zero as the degenerate states are related to each other by a spinful time-reversal symmetry, the many-body ground state in the vicinity of the flux at $\Phi = \pi$ may be described as a bound state that carries a total charge $Q = 0$ and a total spin $S_z = 0$.

Now suppose that $\dot{\Phi} = 2\pi/T \gg |v_{M_z}|$. In this case, a Landau-Zener transition [195, 196] from the filled state to the excited state [see SFig. 9(c)] may occur when the filled state approaches the avoided crossing. The probability of such a transition is given by [197]

$$P \sim \exp\left\{-2\pi \frac{1}{\hbar\,|2\frac{dE}{d\Phi}|} \frac{|v_{M_z}|^2}{\dot{\Phi}}\right\} = \exp\left\{-\frac{T}{2\hbar} \frac{|v_{M_z}|^2}{|\frac{dE}{d\Phi}|}\right\}, \tag{3.60}$$

where we have restored factors of $\hbar$, and where $\frac{dE}{d\Phi}$ is the slope of the gap-crossing mode when $v_{M_z} = 0$ indicated in SFig. 9(b). Since we have assumed $\dot{\Phi} = 2\pi/T \gg |v_{M_z}|$, the probability in SEq. (3.60) will be $P \lesssim 1$, and in such a case we in general expect that the Landau-Zener transition will occur. Therefore, at $t = T/2$ ($\Phi = \pi$), there will be one state filled in the doubly degenerate Kramers pair at the higher mid-gap energy and another state filled in the doubly degenerate Kramers pair at the lower mid-gap energy. Importantly, the spin expectation values $\langle s_z \rangle$ of these two filled mid-gap states will have the same signs and will both individually satisfy $|\langle s_z \rangle| \lesssim \hbar/2$. Again, at $\Phi = \pi$ time-reversal symmetry requires the valence continuum to have total spin equal to zero. Therefore, if we thread the $U(1)$ magnetic flux $\Phi(t)$ with $\dot{\Phi} = 2\pi/T \gg |v_{M_z}|$, the many-body ground state at $\Phi = \pi$ may be described by a spinon bound state with total charge $Q = 0$ and total spin $|S_z| \lesssim \hbar$ (*i.e.* less than or equal to 2 in the units of $\hbar/2$).

We hence conclude that when s_z is not conserved, although the spin Hall conductivity may in principle be decreased to zero, if we thread a $U(1)$ magnetic flux $\Phi(t)$ at a rate much greater than the strength of s_z-conservation-breaking, we still expect to observe a spinon bound state with nearly spin-$\hbar$ when $\Phi = \pi$.

For completeness, we note that if in the s_z-conserving limit the slopes $\left|\frac{dE}{d\Phi}\right|$ of the midgap modes are large enough, then it can be the case that there are no crossing points between modes with opposite slopes within the bulk gap. In this case, if we weakly break the s_z-conservation, there will still be two chiral midgap modes with positive slopes and two chiral midgap modes with negative slopes. If we then insert a magnetic flux Φ, there will in general be no bound

states at $\Phi = \pi$. Instead, we will observe robust counterpropagating edge states circulating around the small hole as a function of Φ.

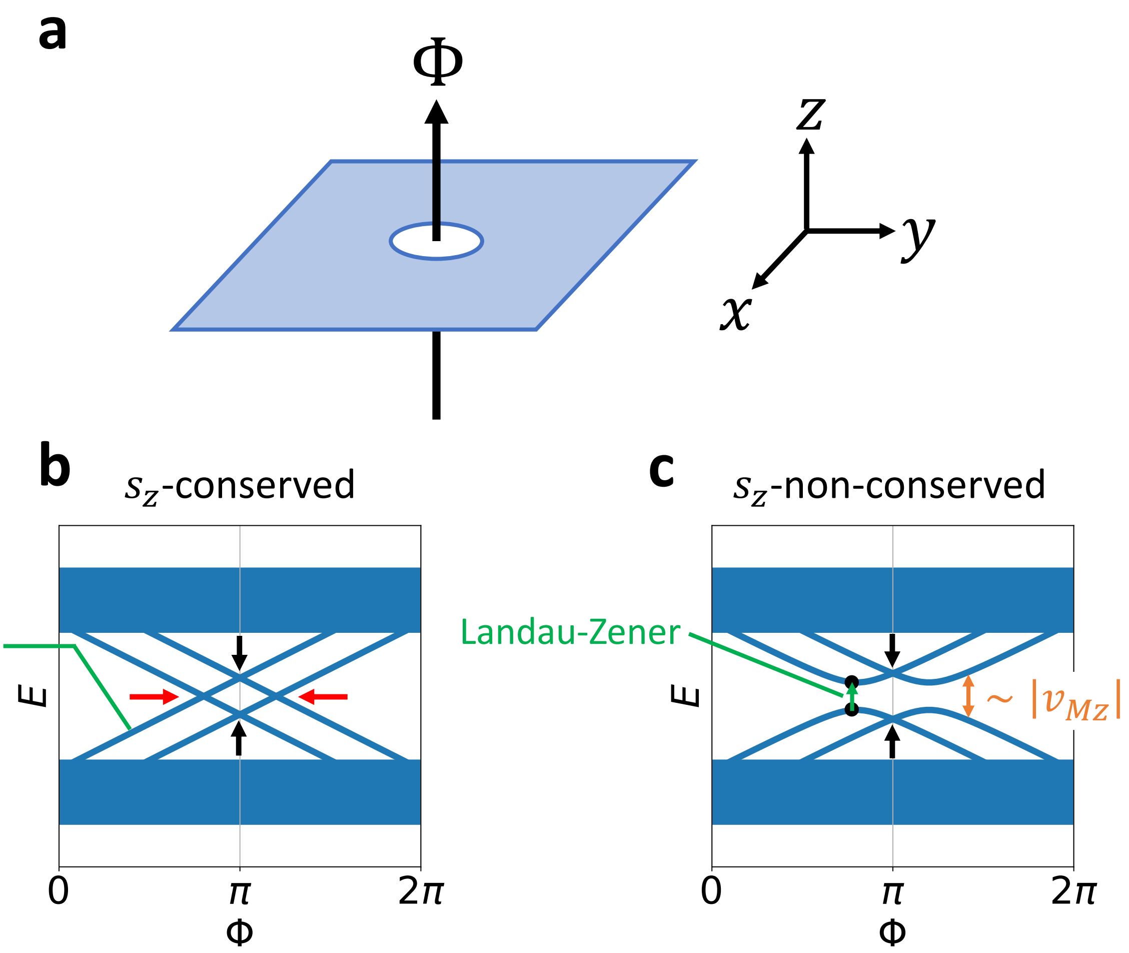

Supplementary Figure 9. Flux insertion for a spin-stable topological phase with spin Chern number $|C^s_{\gamma_1}| = 4$. (a) shows a schematic of a perpendicular $U(1)$ flux Φ threading through a small hole of a 2D system. When $\Phi = 0$ and $\Phi = \pi$ the system has time-reversal symmetry while if $\Phi \neq 0$ or $\Phi \neq \pi$ the time-reversal symmetry is broken [184]. (b) shows a schematic of the energy spectrum of the 2D sample in (a) with infinite size along x and y and a small hole as a function of Φ in the limit of s_z-conservation, which in our 2D fragile topological model [SEq. (3.55)] corresponds to $v_{M_z} = 0$. There is spectral flow as a function of Φ, with two bands crossing the bulk gap with positive slope, and two bands crossing the bulk gap with negative slope. For small values of the mode velocity $\left|\frac{dE}{d\Phi}\right|$, there will generically be four crossing points between modes with positive and negative slope. The two crossings indicated by the red arrows at generic values of Φ are protected by s_z-conservation, and the two indicated by the black arrows at $\Phi = \pi$ are protected by time-reversal symmetry due to Kramers theorem. The slope $\left|\frac{dE}{d\Phi}\right|$ of the gap-crossing modes is also indicated. (c) shows a schematic of the energy spectrum of the 2D sample in (a) with infinite size along x and y and a small hole as a function of Φ when s_z is not conserved, which in our 2D fragile topological model [SEq. (3.55)] corresponds to $v_{M_z} \neq 0$. There are two crossings indicated by the black arrows at $\Phi = \pi$ that are protected by $\mathcal{T}$ symmetry. In other words, the mid-gap localized states at $\Phi = \pi$ remain doubly degenerate due to Kramers theorem. In contrast, the crossings previously indicated by the red arrows in (b) are now gapped since s_z is not conserved due to a nonzero v_{M_z} in the Hamiltonian [SEq. (3.55)]. In particular, the gap is proportional to $|v_{M_z}|$ for small $|v_{M_z}|$. A Landau-Zener transition [195, 196] from the low energy filled state to an excited state is also indicated, whose probability is described in SEq. (3.60).

As such, we have established that the robust spin-stable topology indicated in SFig. 8 can have a physical consequence. In fact, such a robustness persists in the presence of a coupling to trivial atomic orbitals such as those in SEq. (3.59). This is demonstrated in SFig. 10(c,d), in which the ∓ 2 winding number in the k_y-directed $P_\pm$-Wilson loop eigenphases $\{\gamma^\pm_{1,j}(k_x)\}$ persists in the P_6 occupied space. According to the sign convention introduced in SEq. (3.24), this indicates that the partial Chern numbers of the occupied space still remain $C^\pm_{\gamma_1} = \pm 2$ after the addition of trivial

atomic orbitals, under which the occupied space has been enlarged from the bands in P_2 to those in P_6. We note that unlike previously for the 2D TI model analyzed in SFig. 3, the $P_\pm$-Wilson loop spectra for the fragile phase studied in this section [SFig. 10(c,d)] is not a straightforward decomposition of the P-Wilson loop spectrum [SFig. 10(a)]. This occurs precisely because the P-Wilson loop spectrum need not always wind when the $P_\pm$-Wilson loops wind, which is particularly well exemplified by the trivialized fragile phase analyzed in this section.

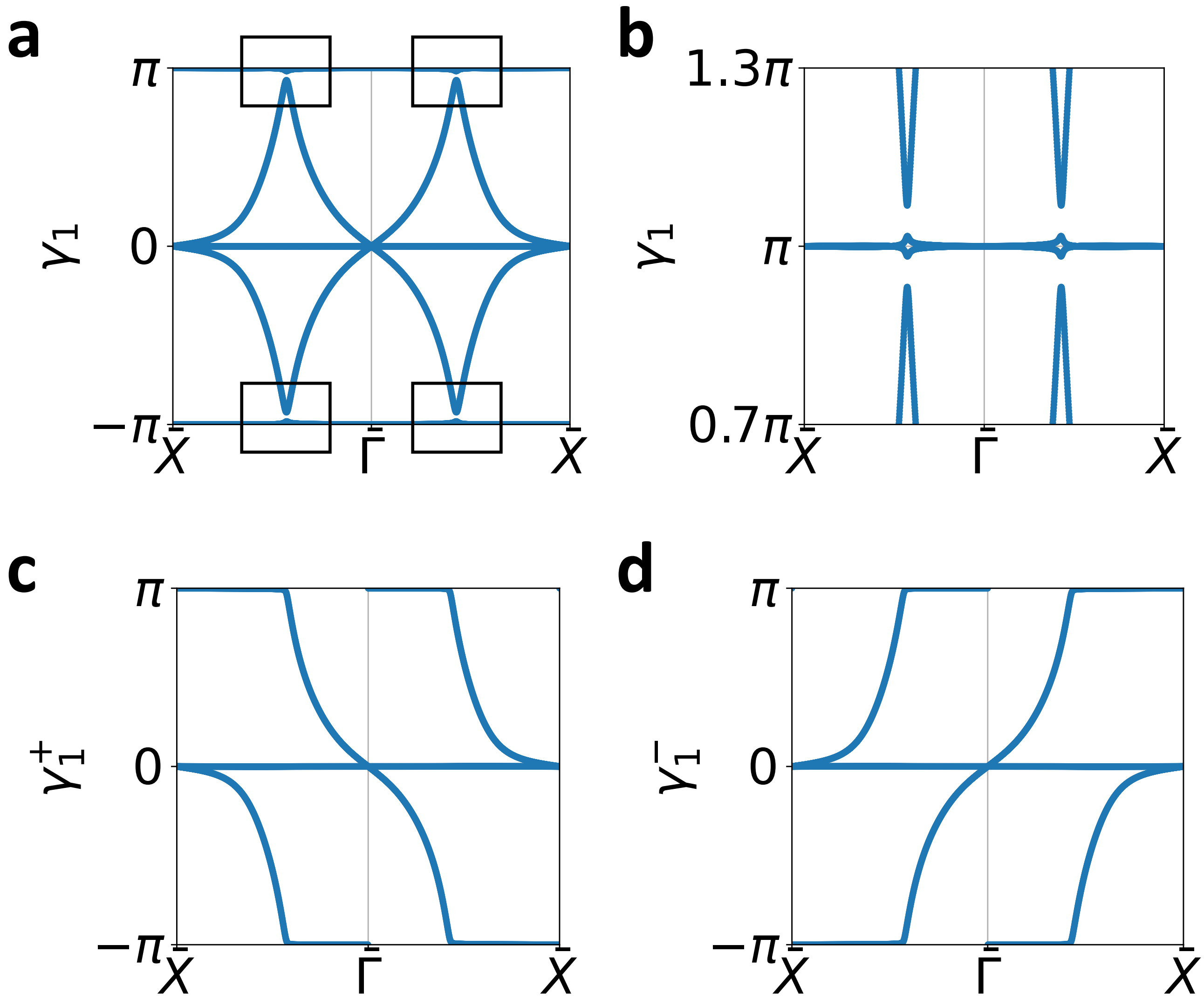

Supplementary Figure 10. k_y-directed Wilson loop eigenphases as a function of k_x in the (a) occupied space P_6 of SFig. 7, (c) positive (+), and (d) negative (−) eigenspace of $P_6 s_z P_6$ where s_z is the z-component of the spin vector $\mathbf{s}$, namely $s_z = \hat{\mathbf{z}} \cdot \mathbf{s}$. (b) shows an enlarged view of (a) around $\gamma_1 = \pi$ to demonstrate that the two-band crossings in SFig. 8(b) are removed. The numbers of bands in (a), (c), and (d) are 6, 3, and 3, respectively. The calculations detailed in this figure were performed using the freely available Python package NESTED_AND_SPIN_RESOLVED_WILSON_LOOP [97], which represents an extension of the PythTB open-source Python tight-binding package [171] that was implemented and utilized for the preparation of SRefs. [8, 15] and the present work.

To sum up, although the occupied space of a fragile TI becomes trivial after including bands induced from trivial atomic orbitals, it is possible that each of the spin-resolved ± eigenspaces of PsP have stable nontrivial *spin-resolved* topology. Importantly, such a *spin-resolved stable nontrivial topology* can contribute to experimentally measurable quantities such as spin Hall conductivity [7] and bound states carrying nonzero total spin around a $U(1)$ magnetic π flux [184]. This provides a crucial example demonstrating that, beyond the simplest 2D Chern and 3D axion insulating phases [155, 158, 198], stable electronic band topology *does not* in general uniquely determine the spin-electromagnetic response of an insulating system. Instead, a finer treatment is required, such as the gauge-invariant

spin-resolved methods introduced in this work.

H. Spin-Resolved Wilson Loops and the Spin Entanglement Spectrum

Recall that for the usual P-Wilson loop, there is a correspondence between the P-Wilson loop spectrum and the bipartite entanglement spectrum. In particular, SRefs. [5, 145] argued that in a band insulator the spectrum of the P-Wilson loop in the $\widehat{\mathbf{G}}_i$ direction is the same as the spectrum of the projected position operator $P\mathbf{x}\cdot\mathbf{G}_iP$, and that this can be continuously deformed to the entanglement spectrum defined as the spectrum of the single-particle correlation matrix $\Theta(\mathbf{x}\cdot\mathbf{G}_i)P\Theta(\mathbf{x}\cdot\mathbf{G}_i)$, where $\Theta(\mathbf{x}\cdot\mathbf{G}_i)$ is zero for states with $\mathbf{x}\cdot\mathbf{G}_i < 0$, and 1 otherwise.

The argument of SRef. [145], relying only on the geometric properties of the projection operator P, applies straightforwardly to our spin projection operators $P_\pm$ onto the upper/lower-half of the spin spectrum provided a spin gap is open. Concretely, the $P_\pm$-Wilson loops in the $\widehat{\mathbf{G}}$ direction are isospectral to the *spin-projected* position operators $P_\pm\mathbf{x}\cdot\mathbf{G}P_\pm$. Following the homotopy arguments of SRef. [145], we can adiabatically deform $P_\pm\mathbf{x}\cdot\mathbf{G}P_\pm$ to the *spin entanglement spectrum* given by the spectrum of $\Theta(\mathbf{x}\cdot\mathbf{G})P_\pm\Theta(\mathbf{x}\cdot\mathbf{G})$. As a consequence, nontrivial spectral flow in the $P_\pm$-Wilson loop implies nontrivial spectral flow in the spin entanglement spectrum, provided that the entanglement cut $\mathbf{x}\cdot\mathbf{G} > 0$ does not break any symmetry that protects the spectral flow. This represents a generalization of the bulk-boundary correspondence to the spin spectrum and the spin entanglement spectrum.

As a concrete example, let us consider first the model for a two-dimensional topological insulator presented in SN 3 D. The spectra of the P_+- and P_--Wilson loops for this model were discussed in SN 3 D and shown in SFig. 3. This model has spin Chern number $C^s_{\gamma_1}$ equal to -2, which is reflected in the $P_\pm$-Wilson loop eigenphases (SFig. 3). In particular, we demonstrated that the P_+-Wilson loop spectrum winds one time with negative slope as a function of k_x, while the P_--Wilson loop spectrum winds one time with positive slope as a function of k_x. We thus expect to see spectral flow in the $P_\pm$ entanglement spectrum, with one mode of negative chirality in the P_+ entanglement spectrum, and one mode of positive chirality in the P_- entanglement spectrum at each boundary of the entanglement cut. To verify this, we numerically compute the spectrum of $\Theta(\mathbf{x}\cdot\mathbf{G})P_\pm\Theta(\mathbf{x}\cdot\mathbf{G})$ on a cylinder with circumference $N = 60$ unit cells in the y-direction. We partition the cylinder into two regions: region A has $0 \le y < 30$, and region B has $30 \le y \le 59$. We then diagonalize $\Theta(y\in A)P_\pm\Theta(y\in A)$, where $\Theta(y\in A)$ is 1 if $y \in A$, and 0 otherwise. We show the results in SFig. 11. Since region A has two boundaries, we see a pair of modes crossing the gap in each of the P_+ and P_- entanglement spectra. Modes with opposite slope correspond to states localized at opposite boundaries of the entanglement region. Each entanglement spectrum has a spectral flow of one mode per boundary, consistent with the winding of the $P_\pm$-Wilson loops.

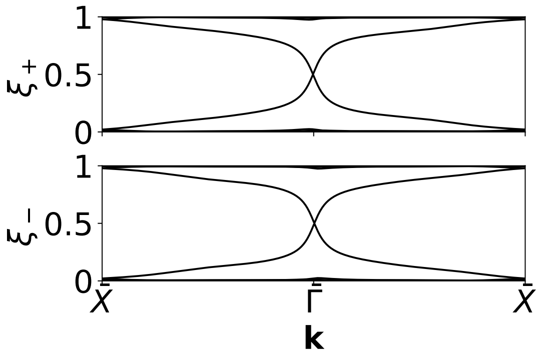

Supplementary Figure 11. Spin entanglement spectrum $\xi_\pm$ for the 2D strong topological insulator considered in SN 3 D. The P_+ entanglement spectrum is shown on the top panel, and the P_- entanglement spectrum is shown on the bottom panel. Each entanglement spectrum shows a single gap-traversing chiral mode per boundary of the entanglement region, consistent with the fact that this model has partial Chern numbers $C^\pm_{\gamma_1}$ equal to ∓ 1.

As a more interesting example, let us consider the model introduced in SRef. [8] and analyzed in SN 3 G for a two-dimensional insulator with partial Chern numbers $C^\pm_{\gamma_1} = \pm 2$ and hence spin Chern number $C^s_{\gamma_1} = +4$. This model is a fragile topological insulator, and hence has no stable winding in the P-Wilson loop spectrum, and no gapless surface states. In SFig. 12 we also verify that the ordinary entanglement spectrum computed from $\Theta(\mathbf{x}\in A)P\Theta(\mathbf{x}\in A)$ (with the region A defined as above) has no spectral flow. Turning to the spin entanglement spectrum, however, we see in SFig. 13 that the spectra of $\Theta(\mathbf{x}\in A)P_\pm\Theta(\mathbf{x}\in A)$ each feature two chiral modes per boundary of the entanglement region, consistent with the winding of the $P_\pm$-Wilson loop eigenphases in SFig. 10. We thus see that

by the bulk-boundary correspondence for the spin spectrum, $C^{\pm}_{\gamma_1} = \pm 2$ implies that the $P_{\pm}$-Wilson loops wind twice, which implies the existence of two chiral modes per boundary in the spin entanglement spectrum.

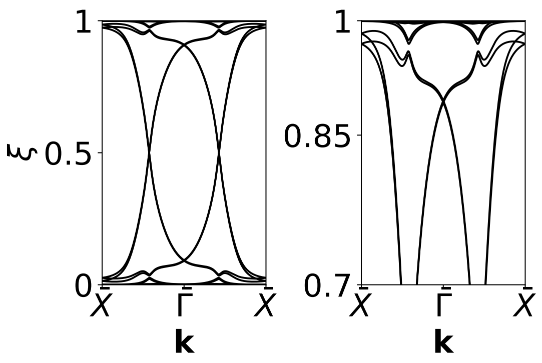

Supplementary Figure 12. Entanglement spectrum ξ for the 2D fragile topological insulator considered in SN 3 G. The left panel shows the full range of the entanglement spectrum. On the right, we zoom in on the range $0.7 \leq \xi \leq 1$. We see that there is no spectral flow in the entanglement spectrum, consistent with the fact that the P-Wilson loop does not wind.

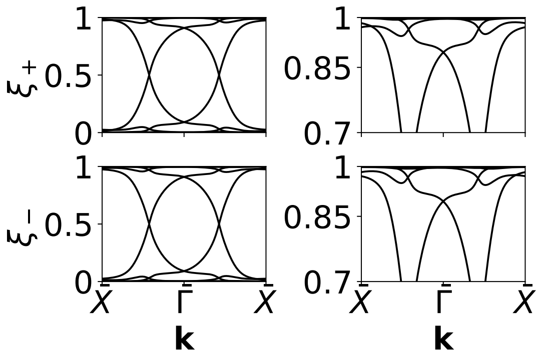

Supplementary Figure 13. Spin entanglement spectrum $\xi_{\pm}$ for the 2D fragile topological insulator considered in SN 3 G. The P_+ entanglement spectrum is shown on the top, and the P_- entanglement spectrum is shown on the bottom. On the right, we zoom in on the range $0.7 \leq \xi_{\pm} \leq 1$. Each entanglement spectrum shows two gap-traversing chiral modes per boundary of the entanglement region, consistent with the fact that this model has partial Chern numbers $C^{\pm}_{\gamma_1}$ equal to ± 2.

As a final example, let us consider the spin entanglement spectrum for a 3D strong topological insulator. We consider the model for a 3D TI with broken inversion symmetry given by SEq. (3.48) with the additional inversion-symmetry breaking term [SEq. (3.52)]. The bulk, slab, and spin band structures, as well as the P- and $P_{\pm}$-Wilson loop eigenphases for this model were shown in SFig. 6. The helical winding of the P-Wilson loop in the $k_z = 0$ plane coupled with the trivial winding of the P-Wilson loop in every other k_z plane implies that the ordinary entanglement spectrum will feature a protected Dirac crossing at $\bar{\Gamma}$ with $\xi = 0.5$ on each entanglement boundary, with nontrivial helical spectral flow. This is indeed confirmed in SFig. 14, where we see that the spectrum $\{\xi\}$ of $\Theta(y)P\Theta(y)$ is homotopic to the slab spectrum in SFig. 6(c).

This result can be interestingly contrasted with the spin entanglement spectrum of a 3D TI. Since the occupied energy bands in every constant-k_z plane between the spin-Weyl nodes at $\pm\mathbf{k}_* = \pm(0, 0.46\pi, -0.21\pi)$ have spin Chern number $C^s_{\gamma_1} = 2$, we expect the $\Theta(y)P_{\pm}\Theta(y)$ entanglement spectrum to be isospectral to the boundary states (and Wilson loop) of a Weyl semimetal. In particular, we expect to find a set of "spin Fermi arcs" in the spectrum of $\Theta(y)P_{\pm}\Theta(y)$ between the projections $\pm\bar{\mathbf{k}}_* = \pm(0, -0.21\pi)$ of the spin-Weyl nodes onto the Brillouin zone of the entanglement cut. In SFig. 15 we show the spectrum $\xi_{\pm}$ of $\Theta(y)P_{\pm}\Theta(y)$ for the 3D TI model, where a Fermi arc state can be seen in the line of $\xi_{\pm} = 0.5$ eigenvalues starting at $\bar{\Gamma}$ and proceeding along the $\bar{\Gamma} - \bar{Z}$ line. To make this clearer, we show in SFig. 16 the P_+ spin entanglement spectrum along a straight path between $-1.2\bar{\mathbf{k}}_*$ and $1.2\bar{\mathbf{k}}_*$, corresponding to the surface projection of the path between spin-Weyl nodes shown in SFig. 6(d), which shows the spin Fermi arc clearly. This thus demonstrates that, according to the generalized bulk-boundary correspondence, the spin entanglement spectrum for a 3D system with spin-Weyl nodes displays spin Fermi arcs connecting the surface projections of the spin-Weyl points.

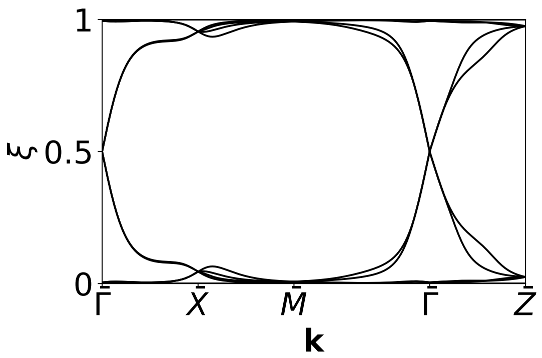

Supplementary Figure 14. Eigenvalues ξ of the entanglement spectrum for the 3D TI model with broken inversion symmetry discussed in SN 3 F and in SFig. 6. The entanglement spectrum shows a protected Dirac crossing at $\bar{\Gamma}$, with nontrivial spectral flow, in correspondence with the gapless surface states of a 3D TI.

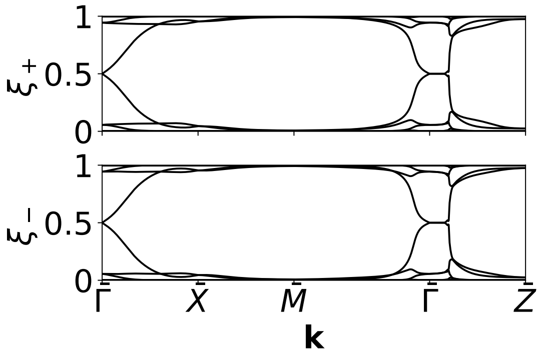

Supplementary Figure 15. Eigenvalues $\xi_\pm$ of the spin entanglement spectrum for the 3D TI model with broken inversion symmetry discussed in SN 3 F and in SFig. 6. Each entanglement spectrum shows a "spin Fermi arc" originating at $\bar{\Gamma}$ and extending along the $\bar{\Gamma}-\bar{Z}$ line, in correspondence with the gapless surface states of a 3D Weyl semimetal.

To conclude, we have shown that the $P_\pm$-Wilson loop spectrum is homotopic to the spin entanglement spectrum, defined as the eigenvalues of the restriction of $P_\pm$ to half the system in position space. Our construction is similar in spirit to the symmetry-resolved entanglement spectrum studied for strongly interacting symmetry-protected topological phases in, *e.g.* SRefs. [199–201]. It should be noted that the spectrum of $\Theta(\mathbf{x}\cdot\mathbf{G})P_\pm\Theta(\mathbf{x}\cdot\mathbf{G})$ differs in important respects from the spin-resolved entanglement spectrum introduced in SRefs. [131–133]. The authors of those works considered an entanglement bipartition not in position space, but in spin space. That is, they considered the (nonzero) spectrum of the matrix $C_\uparrow = P_\uparrow P P_\uparrow$, with no position space restriction [recall we discussed $C_\uparrow$ matrix in SEq. (2.57)]. As $C_\uparrow$ respects the translation symmetry of Hamiltonian, there is no bulk-boundary correspondence for the notion of spin entanglement spectrum in SRefs. [131–133]. By contrast, we have shown here that the spectrum of $\Theta(\mathbf{x}\cdot\mathbf{G})P_\pm\Theta(\mathbf{x}\cdot\mathbf{G})$ is homotopic to the spin-resolved Wilson loop spectrum.

4. NESTED SPIN-RESOLVED WILSON LOOPS

In this section, we will apply our formalism of spin-resolved topology to higher-order topological phases [13–15, 17–19, 21, 33]. To do so, in SN 4 A we will start by reviewing the nested Wilson loop introduced in SRefs. [13, 15, 36] as a diagnostic of bulk higher-order topology. Next, in SN 4 B we will define a nested spin-resolved Wilson loop, which we also call nested $P_\pm$-Wilson loop and can be understood as the nested Wilson loop computed in the subsets of the PsP [SEq. (2.14)] spectrum. In SN 4 C we will discuss general properties of the nested $P_\pm$-Wilson loop, and show how its spectrum can be related to higher-order helical topological invariants described by a set of *spin-stable* invariants that are protected by an energy and a spin gap. In particular, we derive that a 3D spin-gapped crystalline insulator with inversion and spinful time-reversal symmetry has a $\mathbb{Z}_2\times\mathbb{Z}$ spin-resolved topology. In SN 4 D we show how the spin-resolved topology refines the classification of topological crystalline insulators and revealing the existence of a previously unrecognized bulk invariant, the *partial* axion angle, which can be extracted from the nested

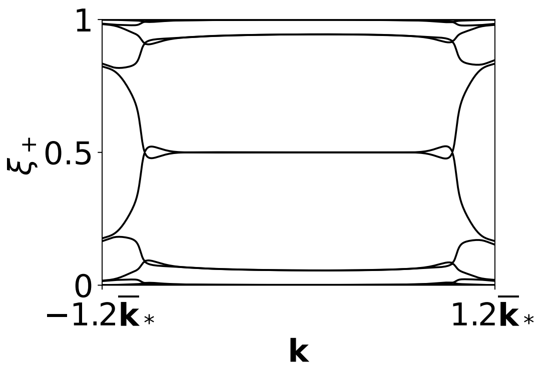

Supplementary Figure 16. Eigenvalues ξ_+ of the spin entanglement spectrum for the 3D TI model with broken inversion symmetry, plotted along a line connecting the surface projection of the spin-Weyl points. The flat spin Fermi arcs at $\xi_+ = 0.5$ are visible over the majority of the plotted range. Later, using the example of β-MoTe$_2$ in SN 9 we will show that spin Fermi arcs manifest as topological surface Fermi arcs in the physical energy spectrum of a spin-Weyl semimetal in a strong Zeeman field.

spin-resolved Wilson loop spectrum. In particular, we will introduce the spin-resolved layer construction which can be deduced from the spin-stable invariants and used to enumerate spinful crystalline insulators, from which we find three different spin-resolved regimes of a helical higher-order topological insulator (HOTI). Finally, in SN 4 E we will apply our formalism to a model of a helical HOTI, paying particular attention to practical considerations that arise in the computation.

A. Nested P-Wilson Loop

Before we develop the formalism of a spin-resolved version of nested Wilson loops in SN 4 B, let us review the ordinary nested P-Wilson loop formalism [8, 13–15, 31, 202]. We consider a 2D or 3D lattice model with discrete translation symmetries and denote the projector to the N_{occ} occupied energy eigenstates at crystal momentum $\mathbf{k}$ by

$$[P(\mathbf{k})] = \sum_{n=1}^{N_{\text{occ}}} |u_{n,\mathbf{k}}\rangle \langle u_{n,\mathbf{k}}| \,. \tag{4.1}$$

We will assume that in the following construction, the energy eigenvalues of the occupied eigenvectors of the Bloch Hamiltonian $[H(\mathbf{k})]$ are spectrally separate from the unoccupied ones such that $[P(\mathbf{k})]$ is well-defined and smooth as a function of $\mathbf{k}$. To form the nested P-Wilson loop, we must first calculate the P-Wilson loop along direction $\mathbf{G}$; in this section and what follows we take $\mathbf{G}$ to be a primitive reciprocal lattice vector, although the formalism applies for more general reciprocal lattice vectors as well. We can then find the eigenvectors of the Wilson loop, allowing us to form a basis of (Fourier-transformed) hybrid Wannier functions from the occupied states. We then will construct a projector $[\tilde{P}_{\mathbf{G}}(\mathbf{k})]$ onto a subset of these hybrid Wannier bands, and compute the eigenvalues for a Wilson loop formed as a product of the $[\tilde{P}_{\mathbf{G}}(\mathbf{k})]$.

To formalize this process, we begin with the $N_{\text{occ}} \times N_{\text{occ}}$ P-Wilson loop matrix $[\mathcal{W}_{1,\mathbf{k},\mathbf{G}}]$ for a holonomy starting at base point $\mathbf{k}$ and going along a straight path to $\mathbf{k}+\mathbf{G}$ where $\mathbf{G}$ is a primitive reciprocal lattice vector [see SEq. (3.2) and surrounding text]. To find the eigenvectors of $[\mathcal{W}_{1,\mathbf{k},\mathbf{G}}]$, we must solve

$$[\mathcal{W}_{1,\mathbf{k},\mathbf{G}}]\, |\nu_{j,\mathbf{k},\mathbf{G}}\rangle = e^{i(\gamma_1)_{j,\mathbf{k},\mathbf{G}}}\, |\nu_{j,\mathbf{k},\mathbf{G}}\rangle \,, \tag{4.2}$$

where $\{|\nu_{j,\mathbf{k},\mathbf{G}}\rangle | j = 1 \ldots N_{\text{occ}}\}$ is a set of N_{occ}-component orthonormal eigenvectors such that $\langle \nu_{i,\mathbf{k},\mathbf{G}} | \nu_{j,\mathbf{k},\mathbf{G}}\rangle = \delta_{ij}$. Recall from SN 3 A that the eigenvalues $(\gamma_1)_{j,\mathbf{k},\mathbf{G}}$ are invariant under shifts $\mathbf{k} \to \mathbf{k}+\Delta\mathbf{k}$ for $\Delta\mathbf{k} \parallel \mathbf{G}$. This means that the eigenvectors $|\nu_{j,\mathbf{k}+\Delta\mathbf{k},\mathbf{G}}\rangle$ are related to the eigenvectors $|\nu_{j,\mathbf{k},\mathbf{G}}\rangle$ by a unitary transformation known as parallel transport [4, 78]. If we define the P-Wilson line matrix

$$[\mathcal{W}_{1,\mathbf{k}'\leftarrow\mathbf{k}}]_{m,n} \equiv \langle u_{m,\mathbf{k}'}| \left(\prod_{\mathbf{q}}^{\mathbf{k}'\leftarrow\mathbf{k}} [P(\mathbf{q})] \right) |u_{n,\mathbf{k}}\rangle, \tag{4.3}$$

where both m and n are the indices of occupied energy eigenvectors, and

$$\left(\prod_{\mathbf{q}}^{\mathbf{k}'\leftarrow\mathbf{k}}[P(\mathbf{q})]\right) = \lim_{N\to\infty}[P(\mathbf{k}')][P(\mathbf{k}+\frac{N-1}{N}(\mathbf{k}'-\mathbf{k}))]\cdots[P(\mathbf{k}+\frac{1}{N}(\mathbf{k}'-\mathbf{k}))][P(\mathbf{k})], \tag{4.4}$$

then the eigenvectors $|\nu_{j,\mathbf{k},\mathbf{G}}\rangle$ satisfy [14]

$$|\nu_{j,\mathbf{k}+\Delta\mathbf{k},\mathbf{G}}\rangle = \exp\left\{-i\frac{(\gamma_1)_{j,\mathbf{k},\mathbf{G}}}{2\pi}(\Delta\mathbf{k})\cdot\mathbf{a}\right\}[\mathcal{W}_{1,\mathbf{k}+\Delta\mathbf{k}\leftarrow\mathbf{k}}]|\nu_{j,\mathbf{k},\mathbf{G}}\rangle. \tag{4.5}$$

Here $\mathbf{a}$ is the real-space primitive lattice vector dual to the primitive reciprocal lattice vector $\mathbf{G}$ such that $\mathbf{a}\cdot\mathbf{G} = 2\pi$. We also note that by definition $[\mathcal{W}_{1,\mathbf{k}+\mathbf{G}\leftarrow\mathbf{k}}]$ [SEq. (4.3)] is equal to $[\mathcal{W}_{1,\mathbf{k},\mathbf{G}}]$ [SEq. (3.2)].

We emphasize that in practice the computation of the eigenvectors $|\nu_{j,\mathbf{k},\mathbf{G}}\rangle$ requires a bit of care. For a given base point we can compute the P-Wilson loop matrix $[\mathcal{W}_{1,\mathbf{k},\mathbf{G}}]$ on a discretized $\mathbf{k}$-mesh using SEq. (3.2). To account for numerical error introduced by the finite $\mathbf{k}$-mesh spacing, we then perform a singular value decomposition (SVD) on $[\mathcal{W}_{1,\mathbf{k},\mathbf{G}}]$ to obtain $[\mathcal{W}_{1,\mathbf{k},\mathbf{G}}] = USV^\dagger$ where U and V are unitary matrices while S is a real and diagonal matrix with non-negative diagonal elements; we then redefine $[\mathcal{W}_{1,\mathbf{k},\mathbf{G}}]$ to be the unitary part $UV^\dagger$ of the decomposition [78]. Being a unitary matrix, we can then diagonalize $[\mathcal{W}_{1,\mathbf{k},\mathbf{G}}]$ to obtain SEq. (4.2) where the eigenvalues are of the unimodular form $e^{i(\gamma_1)_{j,\mathbf{k},\mathbf{G}}}$ with $(\gamma_1)_{j,\mathbf{k},\mathbf{G}}\in\mathbb{R}$. In practice, we use the Schur decomposition $[\mathcal{W}_{1,\mathbf{k},\mathbf{G}}] = ZTZ^\dagger$ to ensure that we obtain orthonormal eigenvectors for the bands of $[\mathcal{W}_{1,\mathbf{k},\mathbf{G}}]$, where Z is a unitary matrix and T is an upper-triangular matrix [203]. Since $[\mathcal{W}_{1,\mathbf{k},\mathbf{G}}]$ is unitary, T is diagonal. Therefore, we can identify the diagonal elements of T as $e^{i(\gamma_1)_{j,\mathbf{k},\mathbf{G}}}$ and the corresponding columns of Z as $|\nu_{j,\mathbf{k},\mathbf{G}}\rangle$ in SEq. (4.2).

We next use the $|\nu_{j,\mathbf{k},\mathbf{G}}\rangle$ to form a set of "hybrid Wannier states" as vectors in our N_{sta}-dimensional Hilbert space as [13–16, 36] (recall that N_{sta} denotes the number of basis states per unit cell in the Hilbert space of our truncated tight-binding Hamiltonian)

$$|w_{j,\mathbf{k},\mathbf{G}}\rangle = \sum_{m=1}^{N_{\text{occ}}}[\nu_{j,\mathbf{k},\mathbf{G}}]_m\,|u_{m,\mathbf{k}}\rangle\,, \tag{4.6}$$

where $[\nu_{j,\mathbf{k},\mathbf{G}}]_m$ is the mth $(m=1,\ldots,N_{\text{occ}})$ component of $|\nu_{j,\mathbf{k},\mathbf{G}}\rangle$. Notice that $|w_{j,\mathbf{k},\mathbf{G}}\rangle$ is an N_{sta}-component vector.

To proceed, we now derive the boundary conditions satisfied by $|w_{j,\mathbf{k},\mathbf{G}}\rangle$. Notice that, upon a shift of the crystal momentum $\mathbf{k}\to\mathbf{k}+\mathbf{G}'$ where $\mathbf{G}'$ is a reciprocal lattice vector, the matrix projector $[P(\mathbf{k})]$ [SEq. (4.1)] to the occupied energy bands transforms as

$$[P(\mathbf{k}+\mathbf{G}')] = \sum_{n\in\text{occ}}|u_{n,\mathbf{k}+\mathbf{G}'}\rangle\langle u_{n,\mathbf{k}+\mathbf{G}'}| \tag{4.7}$$

$$= \sum_{n\in\text{occ}}[V(\mathbf{G}')]^{-1}|u_{n,\mathbf{k}}\rangle\langle u_{n,\mathbf{k}}|[V(\mathbf{G}')] \tag{4.8}$$

$$= [V(\mathbf{G}')]^{-1}[P(\mathbf{k})][V(\mathbf{G}')], \tag{4.9}$$

where we have used $|u_{n,\mathbf{k}+\mathbf{G}'}\rangle = [V(\mathbf{G}')]^{-1}|u_{n,\mathbf{k}}\rangle$ [SEq. (2.12)]. Going further, we can use SEq. (4.9) to show that the product of projectors along a loop starting at the base point $\mathbf{k}+\mathbf{G}'$ and going along a straight path to $\mathbf{k}+\mathbf{G}'+\mathbf{G}$ can be rewritten as

$$\left(\prod_{\mathbf{q}}^{\mathbf{k}+\mathbf{G}'+\mathbf{G}\leftarrow\mathbf{k}+\mathbf{G}'}[P(\mathbf{q})]\right) = \lim_{N\to\infty}[P(\mathbf{k}+\mathbf{G}'+\mathbf{G})][P(\mathbf{k}+\mathbf{G}'+\frac{N-1}{N}\mathbf{G})]\ldots[P(\mathbf{k}+\mathbf{G}'+\frac{1}{N}\mathbf{G})][P(\mathbf{k}+\mathbf{G}')] \tag{4.10}$$

$$= \lim_{N\to\infty}[V(\mathbf{G}')]^{-1}[P(\mathbf{k}+\mathbf{G})][P(\mathbf{k}+\frac{N-1}{N}\mathbf{G})]\ldots[P(\mathbf{k}+\frac{1}{N}\mathbf{G})][P(\mathbf{k})][V(\mathbf{G}')] \tag{4.11}$$

$$= [V(\mathbf{G}')]^{-1}\left(\prod_{\mathbf{q}}^{\mathbf{k}+\mathbf{G}\leftarrow\mathbf{k}}[P(\mathbf{q})]\right)[V(\mathbf{G}')]. \tag{4.12}$$

Using SEqs. (2.12) and (4.12), it follows that upon a shift of the crystal momentum $\mathbf{k}\to\mathbf{k}+\mathbf{G}'$, the matrix elements

of the P-Wilson loop $[\mathcal{W}_{1,\mathbf{k},\mathbf{G}}]$ [SEq. (3.2)] are invariant:

$$[\mathcal{W}_{1,\mathbf{k}+\mathbf{G}',\mathbf{G}}]_{m,n} = \langle u_{m,\mathbf{k}+\mathbf{G}'}| [V(\mathbf{G})] \left(\prod_{\mathbf{q}}^{\mathbf{k}+\mathbf{G}'+\mathbf{G}\leftarrow\mathbf{k}+\mathbf{G}'} [P(\mathbf{q})] \right) |u_{n,\mathbf{k}+\mathbf{G}'}\rangle \tag{4.13}$$

$$= \langle u_{m,\mathbf{k}}| [V(\mathbf{G}')][V(\mathbf{G})][V(\mathbf{G}')]^{-1} \left(\prod_{\mathbf{q}}^{\mathbf{k}+\mathbf{G}\leftarrow\mathbf{k}} [P(\mathbf{q})] \right) [V(\mathbf{G}')][V(\mathbf{G}')]^{-1} |u_{n,\mathbf{k}}\rangle \tag{4.14}$$

$$= \langle u_{m,\mathbf{k}}| [V(\mathbf{G})][V(\mathbf{G}')][V(\mathbf{G}')]^{-1} \left(\prod_{\mathbf{q}}^{\mathbf{k}+\mathbf{G}\leftarrow\mathbf{k}} [P(\mathbf{q})] \right) |u_{n,\mathbf{k}}\rangle \tag{4.15}$$

$$= \langle u_{m,\mathbf{k}}| [V(\mathbf{G})] \left(\prod_{\mathbf{q}}^{\mathbf{k}+\mathbf{G}\leftarrow\mathbf{k}} [P(\mathbf{q})] \right) |u_{n,\mathbf{k}}\rangle \tag{4.16}$$

$$= [\mathcal{W}_{1,\mathbf{k},\mathbf{G}}]_{m,n}. \tag{4.17}$$

In deriving SEq. (4.17) we have used the fact that both $[V(\mathbf{G})]$ and $[V(\mathbf{G}')]$ are diagonal matrices and hence they commute, *i.e.*

$$[V(\mathbf{G})][V(\mathbf{G}')] = [V(\mathbf{G}')][V(\mathbf{G})]. \tag{4.18}$$

Since the $N_{\text{occ}} \times N_{\text{occ}}$ matrix $[\mathcal{W}_{1,\mathbf{k},\mathbf{G}}]$ [SEq. (3.2)] is *invariant* upon a shift of $\mathbf{k} \to \mathbf{k} + \mathbf{G}'$, this implies that we can choose

$$|\nu_{j,\mathbf{k}+\mathbf{G}',\mathbf{G}}\rangle = |\nu_{j,\mathbf{k},\mathbf{G}}\rangle\,. \tag{4.19}$$

for the eigenvectors in SEq. (4.2). Notice that the boundary condition in SEq. (4.19) is consistent with the eigenvalue equation in SEq. (4.2) and the parallel transport condition in SEq. (4.5) if we choose $\mathbf{G}' = \mathbf{G}$. Due to the boundary condition in SEq. (4.19), we then have that the $|w_{j,\mathbf{k},\mathbf{G}}\rangle$ in SEq. (4.6) satisfy

$$|w_{j,\mathbf{k}+\mathbf{G}',\mathbf{G}}\rangle = \sum_{m=1}^{N_{\text{occ}}} [\nu_{j,\mathbf{k}+\mathbf{G}',\mathbf{G}}]_m |u_{m,\mathbf{k}+\mathbf{G}'}\rangle \tag{4.20}$$

$$= \sum_{m=1}^{N_{\text{occ}}} [\nu_{j,\mathbf{k},\mathbf{G}}]_m [V(\mathbf{G}')]^{-1} |u_{m,\mathbf{k}}\rangle \tag{4.21}$$

$$= [V(\mathbf{G}')]^{-1} |w_{j,\mathbf{k},\mathbf{G}}\rangle\,, \tag{4.22}$$

where we have again used SEq. (2.12). We then see that $|w_{j,\mathbf{k},\mathbf{G}}\rangle$ satisfies the same boundary conditions as the eigenstates of the Bloch Hamiltonian [SEq. (2.12)], and so can be regarded as physical states. In this work, we will call the $|w_{j,\mathbf{k},\mathbf{G}}\rangle$ the $\widehat{\mathbf{G}}$*-directed* P*-Wannier band basis* [13–15], or simply the P*-Wannier basis.*

The $|w_{j,\mathbf{k},\mathbf{G}}\rangle$ are the single-particle eigenstate of the P-Wilson loop matrix $[\mathcal{W}_{1,\mathbf{k},\mathbf{G}}]$ with eigenphase $(\gamma_1)_{j,\mathbf{k},\mathbf{G}}$ [SEq. (4.2)], expressed in terms of the tight-binding basis states. As long as there is a spectral gap in the P-Wannier bands, namely the eigenphase dispersion $\{(\gamma_1)_{j,\mathbf{k},\mathbf{G}} | j = 1 \ldots N_{\text{occ}}\}$ as a function of $\mathbf{k}$, we can further choose a subspace containing N_W P-Wannier bands to form the matrix projector

$$[\widetilde{P}_{\mathbf{G}}(\mathbf{k})] = \sum_{j=1}^{N_W} |w_{j,\mathbf{k},\mathbf{G}}\rangle \langle w_{j,\mathbf{k},\mathbf{G}}|\,, \tag{4.23}$$

where $N_W \leq N_{\text{occ}}$, and the subscript $\mathbf{G}$ of $[\widetilde{P}_{\mathbf{G}}(\mathbf{k})]$ indicates that the P-Wannier basis states $|w_{j,\mathbf{k},\mathbf{G}}\rangle$ are obtained by diagonalizing the P-Wilson loop matrix [SEq. (3.2)] for a loop taken parallel to $\mathbf{G}$. We will refer to the states in the image of $[\widetilde{P}_{\mathbf{G}}]$ as the "occupied" P-Wannier bands, by analogy to the occupied energy eigenstates in the image of $[P]$ [SEq. (4.1)]. Being a projector onto a subspace that is spectrally isolated, we can construct the $N_W \times N_W$ $\tilde{P}_{\mathbf{G}}$-Wilson loop matrix along a loop $\mathbf{G}'$ with the matrix elements

$$[\mathcal{W}_{2,\mathbf{k},\mathbf{G},\mathbf{G}'}]_{i,j} = \langle w_{i,\mathbf{k}+\mathbf{G}',\mathbf{G}}| \left(\prod_{\mathbf{q}}^{\mathbf{k}+\mathbf{G}'\leftarrow\mathbf{k}} [\widetilde{P}_{\mathbf{G}}(\mathbf{q})] \right) |w_{j,\mathbf{k},\mathbf{G}}\rangle = \langle w_{i,\mathbf{k},\mathbf{G}}| [V(\mathbf{G}')] \left(\prod_{\mathbf{q}}^{\mathbf{k}+\mathbf{G}'\leftarrow\mathbf{k}} [\widetilde{P}_{\mathbf{G}}(\mathbf{q})] \right) |w_{j,\mathbf{k},\mathbf{G}}\rangle\,, \tag{4.24}$$

where i and j range from $1 \dots N_W$, and where we have used the boundary condition [SEq. (4.22)] for $|w_{j,\mathbf{k},\mathbf{G}}\rangle$. We term $[\mathcal{W}_{2,\mathbf{k},\mathbf{G},\mathbf{G}'}]$ the *nested P-Wilson loop matrix.* From SEq. (4.24), we see that by definition, the nested P-Wilson loop matrix is the *$\tilde{P}_{\mathbf{G}}$-Wilson loop matrix for the "occupied" Wannier bands.* We will then denote the unimodular eigenvalues of the nested P-Wilson loop matrix $[\mathcal{W}_{2,\mathbf{k},\mathbf{G},\mathbf{G}'}]$ as $\exp\{i(\gamma_2)_{j,\mathbf{k},\mathbf{G},\mathbf{G}'}\}$, where $j = 1, \dots, N_W$. We call the set $\{(\gamma_2)_{j,\mathbf{k},\mathbf{G},\mathbf{G}'} | j = 1 \dots N_W\}$ as a function of $\mathbf{k}$ the "*nested* P-Wannier bands," where j is the band index. The eigenphases $\{(\gamma_2)_{j,\mathbf{k},\mathbf{G},\mathbf{G}'} | j = 1 \dots N_W\}$ give the positions of hybrid Wannier functions formed from the N_W "occupied" P-Wannier bands and localized along the lattice vector $\mathbf{a}'$ dual to the reciprocal lattice vector $\mathbf{G}'$ [13–15]. The eigenphases $(\gamma_2)_{j,\mathbf{k},\mathbf{G},\mathbf{G}'}$ of the nested P-Wilson loop matrix [SEq. (4.24)] are also the non-Abelian Berry phases of the N_W "occupied "P-Wannier bands for the closed loop in $\mathbf{k}$-space parallel to $\mathbf{G}'$. In particular, the eigenphases $(\gamma_2)_{j,\mathbf{k},\mathbf{G},\mathbf{G}'}$ are independent of the momentum component $\mathbf{k} \cdot \mathbf{a}'$. Note, however, that $(\gamma_2)_{j,\mathbf{k},\mathbf{G},\mathbf{G}'}$ in general depend on the momentum component $\mathbf{k} \cdot \mathbf{a}$ where $\mathbf{a}$ is the primitive real-space lattice vector dual to the primitive reciprocal lattice vector $\mathbf{G}$. In SN 6 B and 6 D we prove the constraints on the values of the nested P-Wilson loop eigenphases at different $\mathbf{k}$ points due to inversion and time-reversal symmetries.

We emphasize that in defining the nested Wilson loop in SEq. (4.24) it was crucial to have the eigenvectors $|\nu_{j,\mathbf{k},\mathbf{G}}\rangle$ be orthonormal in order for $[\widetilde{P}_{\mathbf{G}}(\mathbf{k})]$ defined in SEq. (4.23) to be a projection matrix. Numerically, this was guaranteed by our use of the Schur decomposition. Importantly, the eigenvectors $|\nu_{j,\mathbf{k},\mathbf{G}}\rangle$ obtained numerically via the Schur decomposition only satisfy the parallel transport equation [SEq. (4.5)] up to an arbitrary complex phase (or more generally up to an arbitrary unitary transformation between states with equal $(\gamma_1)_{j,\mathbf{k},\mathbf{G}}$. Nevertheless, since our formalism for computing the non-Abelian (nested) Berry phases makes use of only the matrix projection operators, this numerical ambiguity will not affect our computation of the eigenphases $(\gamma_1)_{j,\mathbf{k},\mathbf{G}}$ and $(\gamma_2)_{j,\mathbf{k},\mathbf{G},\mathbf{G}'}$. However it should be noted that in order to correctly construct the (nested) hybrid Wannier functions, one needs to numerically compute the eigenvector of the Wilson loop matrix [SEq. (3.2)] and nested Wilson loop matrix [SEq. (4.24)] at a given base point $\mathbf{k}$ and obtain the eigenvectors at the other momenta via parallel transport equations such as SEq. (4.5) [14, 16].

Prior to this work, the nested P-Wilson loop formalism described in this section has been applied to identify signatures of several nontrivial insulating phases of matter, including quantized electric multipole insulators [13, 14], magnetic axion insulators [15], and higher-order topology in transition metal dichalcogenides [31]. For example, magnetic axion insulators with 3D spatial inversion symmetry have a nontrivial $\mathbb{Z}_2$-stable spectral flow [15] in the eigenphases $(\gamma_2)_{j,\mathbf{k},\mathbf{G},\mathbf{G}'}$. In the next section (SN 4 B), we will develop a spin-resolved version of nested P-Wilson loop formalism with an aim in the application to identify signatures of spin-stable higher-order topology.

B. Nested $P_\pm$-Wilson Loop

We will now move to one of the larger theoretical and numerical methods introduced in this work: we will apply the formalism of the previous SN 4 A to the spin-resolved projectors $P_\pm$ in order to define a nested $P_\pm$-Wilson loop. As in SN 3 B, we define the spin operator along $\hat{\mathbf{n}}$ to be $s \equiv (\hat{\mathbf{n}} \cdot \boldsymbol{\sigma}) \otimes \mathbb{I}_{N_{\text{orb}}}$, and denote $N_{\text{sta}} = 2N_{\text{orb}}$. The spin operator s satisfies $[V(\mathbf{G})]s[V(\mathbf{G})]^{-1} = s$, as previously proved in SEqs. (3.11)–(3.13), such that the reduced spin matrix $[s_{\text{reduced}}(\mathbf{k})]$ [SEq. (3.5)] satisfies the periodic boundary condition [SEq. (3.14)]. We will suppose that the energy and spin gap are both open for every $\mathbf{k}$ point such that $[P(\mathbf{k})]$, the matrix projector to the occupied energy bands in SEq. (3.1), and $[P_\pm(\mathbf{k})]$, the matrix projector to the upper/lower spin bands in SEq. (3.16), are always well-defined and smooth over the BZ.

To begin, we consider the $N^\pm_{\text{occ}} \times N^\pm_{\text{occ}}$ $P_\pm$-Wilson loop matrix $[\mathcal{W}^\pm_{1,\mathbf{k},\mathbf{G}}]$ defined in SEq. (3.17). This is the holonomy for a loop starting at base point $\mathbf{k}$ and going along a straight path to $\mathbf{k} + \mathbf{G}$, where $\mathbf{G}$ is a primitive reciprocal lattice vector, and $N^+_{\text{occ}} + N^-_{\text{occ}} = N_{\text{occ}}$ is the total number of occupied energy bands of the Bloch Hamiltonian $[H(\mathbf{k})]$.

As in SN 4 A, our goal will be to construct a projector onto a subset of $P_\pm$-Wannier bands. To do this, we must first find the eigenvectors of $[\mathcal{W}^\pm_{1,\mathbf{k},\mathbf{G}}]$, which satisfy

$$[\mathcal{W}^\pm_{1,\mathbf{k},\mathbf{G}}] \left|\nu^\pm_{j,\mathbf{k},\mathbf{G}}\right\rangle = e^{i(\gamma^\pm_1)_{j,\mathbf{k},\mathbf{G}}} \left|\nu^\pm_{j,\mathbf{k},\mathbf{G}}\right\rangle, \tag{4.25}$$

where the $N^\pm_{\text{occ}}$-component eigenvectors $|\nu^\pm_{j,\mathbf{k},\mathbf{G}}\rangle$ $(j = 1, \dots, N^\pm_{\text{occ}})$ are orthonormal such that

$$\left\langle \nu^\pm_{i,\mathbf{k},\mathbf{G}} \middle| \nu^\pm_{j,\mathbf{k},\mathbf{G}} \right\rangle = \delta_{ij}. \tag{4.26}$$

The eigenvectors $\left|\nu^\pm_{j,\mathbf{k},\mathbf{G}}\right\rangle$ satisfy a parallel transport condition by analogy with SEq. (4.5): Defining the $P_\pm$-Wilson

line matrix

$$[\mathcal{W}^{\pm}_{1,\mathbf{k}'\leftarrow\mathbf{k}}]_{m,n} \equiv \langle u^{\pm}_{m,\mathbf{k}'}| \left(\prod_{\mathbf{q}}^{\mathbf{k}'\leftarrow\mathbf{k}} [P_{\pm}(\mathbf{q})]\right) |u^{\pm}_{n,\mathbf{k}}\rangle, \tag{4.27}$$

where $m, n = 1 \dots N^{\pm}_{\text{occ}}$ index the upper/lower spin bands, and

$$\left(\prod_{\mathbf{q}}^{\mathbf{k}'\leftarrow\mathbf{k}} [P_{\pm}(\mathbf{q})]\right) = \lim_{N\to\infty} [P_{\pm}(\mathbf{k}')][P_{\pm}(\mathbf{k} + \frac{N-1}{N}(\mathbf{k}' - \mathbf{k}))] \cdots [P_{\pm}(\mathbf{k} + \frac{1}{N}(\mathbf{k}' - \mathbf{k}))][P_{\pm}(\mathbf{k})], \tag{4.28}$$

the eigenvector $\left|\nu^{\pm}_{j,\mathbf{k},\mathbf{G}}\right\rangle$ satisfies the parallel transport condition [14]

$$|\nu^{\pm}_{j,\mathbf{k}+\Delta\mathbf{k},\mathbf{G}}\rangle = \exp\left\{-i\frac{(\gamma^{\pm}_{1})_{j,\mathbf{k},\mathbf{G}}}{2\pi}(\Delta\mathbf{k}) \cdot \mathbf{a}\right\}[\mathcal{W}^{\pm}_{1,\mathbf{k}+\Delta\mathbf{k}\leftarrow\mathbf{k}}]|\nu^{\pm}_{j,\mathbf{k},\mathbf{G}}\rangle \tag{4.29}$$

for $\Delta\mathbf{k} \parallel \mathbf{G}$, where $\mathbf{a}$ is the real-space primitive lattice vector dual to the primitive reciprocal lattice vector $\mathbf{G}$ such that $\mathbf{a} \cdot \mathbf{G} = 2\pi$. Also notice that by definition $[\mathcal{W}^{\pm}_{1,\mathbf{k}+\mathbf{G}\leftarrow\mathbf{k}}]$ [SEq. (4.27)] is equal to $[\mathcal{W}^{\pm}_{1,\mathbf{k},\mathbf{G}}]$ [SEq. (3.17)]. Note that we have not imposed time-reversal symmetry, and therefore N^{+}_{occ} can be different from N^{-}_{occ}. Consequently, the spin-resolved Wilson loop matrices $[\mathcal{W}^{+}_{1,\mathbf{k},\mathbf{G}}]$ and $[\mathcal{W}^{-}_{1,\mathbf{k},\mathbf{G}}]$ need not generally be matrices of the same size. As described in SN 3 B, the eigenphases $(\gamma^{\pm}_{1})_{j,\mathbf{k},\mathbf{G}}$ are the non-Abelian Berry phases for the subset of the occupied states in the image of $P_{\pm}$. Denoting $\mathbf{a}$ as the real-space primitive lattice vector dual to $\mathbf{G}$, we have that the eigenphases $(\gamma^{\pm}_{1})_{j,\mathbf{k},\mathbf{G}}/(2\pi)$ correspond to the localized positions (along and measured in unit of $\mathbf{a}$) of *spin-resolved* hybrid Wannier states formed from linear combinations of states in the image of $P_{\pm}$. The numerical methods that we employ in this work to find the eigenvectors $\left|\nu^{\pm}_{j,\mathbf{k},\mathbf{G}}\right\rangle$ are the same as those introduced in SN 4 A. We first evaluate $[\mathcal{W}^{\pm}_{1,\mathbf{k},\mathbf{G}}]$ using the projector product in SEq. (3.17). We then employ a singular value decomposition to extract the unitary part of $[\mathcal{W}^{+}_{1,\mathbf{k},\mathbf{G}}]$. A further Schur decomposition of the result then gives us the orthonormal eigenvectors $\left|\nu^{\pm}_{j,\mathbf{k},\mathbf{G}}\right\rangle$ [SEq. (4.25)].

Analogous to SEq. (4.6), we next use the $\left|\nu^{\pm}_{j,\mathbf{k},\mathbf{G}}\right\rangle$ to form a set of spin-resolved "hybrid Wannier states" as vectors in our N_{sta}-dimensional Hilbert space as [13–15]

$$\left|w^{\pm}_{j,\mathbf{k},\mathbf{G}}\right\rangle = \sum_{m=1}^{N^{\pm}_{\text{occ}}} [\nu^{\pm}_{j,\mathbf{k},\mathbf{G}}]_m \left|u^{\pm}_{m,\mathbf{k}}\right\rangle, \tag{4.30}$$

where $[\nu^{\pm}_{j,\mathbf{k},\mathbf{G}}]_m$ is the mth ($m = 1, \dots, N^{\pm}_{\text{occ}}$) component of $\left|\nu^{\pm}_{j,\mathbf{k},\mathbf{G}}\right\rangle$. Notice that $\left|w^{\pm}_{j,\mathbf{k},\mathbf{G}}\right\rangle$ is an $N_{\text{sta}} = 2N_{\text{orb}}$-component vector.

To proceed, we now derive the boundary conditions satisfied by $\left|\nu^{\pm}_{j,\mathbf{k},\mathbf{G}}\right\rangle$ and $\left|w^{\pm}_{j,\mathbf{k},\mathbf{G}}\right\rangle$. First, we note that as previously proved in SEq. (3.23), the eigenstates $\left|u^{\pm}_{m,\mathbf{k}}\right\rangle$ of the projected spin operator $[P(\mathbf{k})]s[P(\mathbf{k})]$ satisfy the boundary condition $\left|u^{\pm}_{m,\mathbf{k}+\mathbf{G}'}\right\rangle = [V(\mathbf{G}')]^{-1}\left|u^{\pm}_{m,\mathbf{k}}\right\rangle$ under a shift of the crystal momentum from $\mathbf{k}$ to $\mathbf{k} + \mathbf{G}'$, where $\mathbf{G}'$ is any reciprocal lattice vector. Hence the projection operators $[P_{\pm}(\mathbf{k})]$ onto the upper/lower spin bands satisfy

$$[P_{\pm}(\mathbf{k} + \mathbf{G}')] = \sum_{n=1}^{N^{\pm}_{\text{occ}}} \left|u^{\pm}_{n,\mathbf{k}+\mathbf{G}'}\right\rangle \left\langle u^{\pm}_{n,\mathbf{k}+\mathbf{G}'}\right| \tag{4.31}$$

$$= \sum_{n=1}^{N^{\pm}_{\text{occ}}} [V(\mathbf{G}')]^{-1} \left|u^{\pm}_{n,\mathbf{k}}\right\rangle \left\langle u^{\pm}_{n,\mathbf{k}}\right| [V(\mathbf{G}')] \tag{4.32}$$

$$= [V(\mathbf{G}')]^{-1}[P_{\pm}(\mathbf{k})][V(\mathbf{G}')], \tag{4.33}$$

where we have used $[V(\mathbf{G}')]^\dagger = [V(\mathbf{G}')]^{-1}$. Second, the product of matrix projectors $[P_\pm(\mathbf{k})]$ satisfies

$$\left(\prod_{\mathbf{q}}^{\mathbf{k}+\mathbf{G}'+\mathbf{G}\leftarrow\mathbf{k}+\mathbf{G}'}[P_\pm(\mathbf{q})]\right) = \lim_{N\to\infty}[P_\pm(\mathbf{k}+\mathbf{G}'+\mathbf{G})][P_\pm(\mathbf{k}+\mathbf{G}'+\frac{N-1}{N}\mathbf{G})]\dots[P_\pm(\mathbf{k}+\mathbf{G}'+\frac{1}{N}\mathbf{G})][P_\pm(\mathbf{k}+\mathbf{G}')] \tag{4.34}$$

$$= \lim_{N\to\infty}[V(\mathbf{G}')]^{-1}[P_\pm(\mathbf{k}+\mathbf{G})][P_\pm(\mathbf{k}+\frac{N-1}{N}\mathbf{G})]\dots[P_\pm(\mathbf{k}+\frac{1}{N}\mathbf{G})][P_\pm(\mathbf{k})][V(\mathbf{G}')] \tag{4.35}$$

$$= [V(\mathbf{G}')]^{-1}\left(\prod_{\mathbf{q}}^{\mathbf{k}+\mathbf{G}\leftarrow\mathbf{k}}[P_\pm(\mathbf{q})]\right)[V(\mathbf{G}')]. \tag{4.36}$$

Putting together SEqs. (4.33) and (4.36), we can then show that, under a shift of the crystal momentum from $\mathbf{k}$ to $\mathbf{k}+\mathbf{G}'$, the $N^\pm_{\text{occ}}\times N^\pm_{\text{occ}}$ $P_\pm$-Wilson loop matrix $[\mathcal{W}^\pm_{1,\mathbf{k},\mathbf{G}}]$ in SEq. (3.17) is invariant:

$$[\mathcal{W}^\pm_{1,\mathbf{k}+\mathbf{G}',\mathbf{G}}]_{m,n} = \left\langle u^\pm_{m,\mathbf{k}+\mathbf{G}'}\right|[V(\mathbf{G})]\left(\prod_{\mathbf{q}}^{\mathbf{k}+\mathbf{G}'+\mathbf{G}\leftarrow\mathbf{k}+\mathbf{G}'}[P_\pm(\mathbf{q})]\right)\left|u^\pm_{n,\mathbf{k}+\mathbf{G}'}\right\rangle \tag{4.37}$$

$$= \left\langle u^\pm_{m,\mathbf{k}}\right|[V(\mathbf{G}')][V(\mathbf{G})][V(\mathbf{G}')]^{-1}\left(\prod_{\mathbf{q}}^{\mathbf{k}+\mathbf{G}\leftarrow\mathbf{k}}[P_\pm(\mathbf{q})]\right)[V(\mathbf{G}')][V(\mathbf{G}')]^{-1}\left|u^\pm_{n,\mathbf{k}}\right\rangle \tag{4.38}$$

$$= \left\langle u^\pm_{m,\mathbf{k}}\right|[V(\mathbf{G})][V(\mathbf{G}')][V(\mathbf{G}')]^{-1}\left(\prod_{\mathbf{q}}^{\mathbf{k}+\mathbf{G}\leftarrow\mathbf{k}}[P_\pm(\mathbf{q})]\right)\left|u^\pm_{n,\mathbf{k}}\right\rangle \tag{4.39}$$

$$= \left\langle u^\pm_{m,\mathbf{k}}\right|[V(\mathbf{G})]\left(\prod_{\mathbf{q}}^{\mathbf{k}+\mathbf{G}\leftarrow\mathbf{k}}[P_\pm(\mathbf{q})]\right)\left|u^\pm_{n,\mathbf{k}}\right\rangle \tag{4.40}$$

$$= [\mathcal{W}^\pm_{1,\mathbf{k},\mathbf{G}}]_{m,n}. \tag{4.41}$$

As in SN 4 A, we have used the fact that $[V(\mathbf{G})]$ and $[V(\mathbf{G}')]$ commute, as shown in SEq. (4.18). Since the $N^\pm_{\text{occ}}\times N^\pm_{\text{occ}}$ matrix $[\mathcal{W}^\pm_{1,\mathbf{k},\mathbf{G}}]$ [SEq. (3.17)] is *invariant* upon a shift of crystal momentum from $\mathbf{k}$ to $\mathbf{k}+\mathbf{G}'$, we can choose without loss of generality

$$\left|\nu^\pm_{j,\mathbf{k}+\mathbf{G}',\mathbf{G}}\right\rangle = \left|\nu^\pm_{j,\mathbf{k},\mathbf{G}}\right\rangle. \tag{4.42}$$

for the eigenvectors in SEq. (4.25). Notice that the boundary condition in SEq. (4.42) is consistent with the eigenvalue equation in SEq. (4.25) and the parallel transport condition in SEq. (4.29) if we choose $\mathbf{G}'=\mathbf{G}$. With the boundary conditions in SEqs. (3.23) and (4.42), we then have that the $|w^\pm_{j,\mathbf{k},\mathbf{G}}\rangle$ in SEq. (4.30) satisfy

$$\left|w^\pm_{j,\mathbf{k}+\mathbf{G}',\mathbf{G}}\right\rangle = \sum_{m=1}^{N^\pm_{\text{occ}}}[\nu^\pm_{j,\mathbf{k}+\mathbf{G}',\mathbf{G}}]_m\left|u^\pm_{m,\mathbf{k}+\mathbf{G}'}\right\rangle \tag{4.43}$$

$$= \sum_{m=1}^{N^\pm_{\text{occ}}}[\nu^\pm_{j,\mathbf{k},\mathbf{G}}]_m[V(\mathbf{G}')]^{-1}\left|u^\pm_{m,\mathbf{k}}\right\rangle \tag{4.44}$$

$$= [V(\mathbf{G}')]^{-1}\left|w^\pm_{j,\mathbf{k},\mathbf{G}}\right\rangle. \tag{4.45}$$

We see that $\left|w^\pm_{j,\mathbf{k},\mathbf{G}}\right\rangle$ satisfies the same boundary conditions as both the eigenstates of the Bloch Hamiltonian [SEq. (2.12)] and also the eigenstates $|u^\pm_{m,\mathbf{k}}\rangle$ of the projected spin operator $[P(\mathbf{k})]s[P(\mathbf{k})]$ [SEq. (3.23)]. We can thus regard the $\left|w^\pm_{j,\mathbf{k},\mathbf{G}}\right\rangle$ as physical states. We will call the $\left|w^\pm_{j,\mathbf{k},\mathbf{G}}\right\rangle$ the $\hat{\mathbf{G}}$-*directed* $P_\pm$-*Wannier band basis* [13–15], or interchangeably the $P_\pm$-*Wannier basis*.

The $\left|w^\pm_{j,\mathbf{k},\mathbf{G}}\right\rangle$ are the single-particle eigenstate of the $P_\pm$-Wilson loop matrix $[\mathcal{W}^\pm_{1,\mathbf{k},\mathbf{G}}]$ with eigenphase $(\gamma^\pm_1)_{j,\mathbf{k},\mathbf{G}}$, expressed in terms of the spinful tight-binding basis states. As long as there is a spectral gap in the $P_\pm$-Wannier

bands, namely the eigenphase dispersion $\{(\gamma_1^{\pm})_{j,\mathbf{k},\mathbf{G}}|j=1\ldots N_{\text{occ}}^{\pm}\}$ as a function of $\mathbf{k}$ (*i.e.* as long as there is a gap in the spin-resolved Wilson loop spectrum), we can further choose a subspace containing $N_W^{\pm}$ $P_{\pm}$-Wannier bands to form the matrix projector

$$[\widetilde{P}_{\mathbf{G}}^{\pm}(\mathbf{k})]=\sum_{j=1}^{N_W^{\pm}}\left|w_{j,\mathbf{k},\mathbf{G}}^{\pm}\right\rangle\left\langle w_{j,\mathbf{k},\mathbf{G}}^{\pm}\right|, \tag{4.46}$$

where $N_W^{\pm}\leq N_{\text{occ}}^{\pm}$, and the subscript $\mathbf{G}$ of $[\widetilde{P}_{\mathbf{G}}^{\pm}(\mathbf{k})]$ indicates that the $P_{\pm}$-Wannier basis is obtained by diagonalizing the $P_{\pm}$-Wilson loop matrix [SEq. (3.17)] for a loop taken parallel to $\mathbf{G}$. Again, being a projector onto a subspace that is spectrally isolated, we can construct the $N_W^{\pm}\times N_W^{\pm}$ $\tilde{P}_{\mathbf{G}}^{\pm}$-Wilson loop matrix along a loop parallel to $\mathbf{k}+\mathbf{G}'$ with matrix elements

$$[\mathcal{W}_{2,\mathbf{k},\mathbf{G},\mathbf{G}'}^{\pm}]_{i,j}=\left\langle w_{i,\mathbf{k}+\mathbf{G}',\mathbf{G}}^{\pm}\right|\left(\prod_{\mathbf{q}}^{\mathbf{k}+\mathbf{G}'\leftarrow\mathbf{k}}[\widetilde{P}_{\mathbf{G}}^{\pm}(\mathbf{q})]\right)\left|w_{j,\mathbf{k},\mathbf{G}}^{\pm}\right\rangle=\left\langle w_{i,\mathbf{k},\mathbf{G}}^{\pm}\right|[V(\mathbf{G}')]\left(\prod_{\mathbf{q}}^{\mathbf{k}+\mathbf{G}'\leftarrow\mathbf{k}}[\widetilde{P}_{\mathbf{G}}^{\pm}(\mathbf{q})]\right)\left|w_{j,\mathbf{k},\mathbf{G}}^{\pm}\right\rangle, \tag{4.47}$$

where i and j range from $1,\ldots,N_W^{\pm}$, and where we have used the boundary condition in SEq. (4.45) for $\left|w_{j,\mathbf{k},\mathbf{G}}^{\pm}\right\rangle$. We term $[\mathcal{W}_{2,\mathbf{k},\mathbf{G},\mathbf{G}'}^{\pm}]$ the *nested $P_{\pm}$-Wilson loop matrix.* From SEq. (4.47), we see that by definition, the nested $P_{\pm}$-Wilson loop matrix is the *$\tilde{P}_{\mathbf{G}}^{\pm}$-Wilson loop matrix for the "occupied" $P_{\pm}$-Wannier bands.* We then denote the unimodular eigenvalues of the $N_W^{\pm}\times N_W^{\pm}$ nested $P_{\pm}$-Wilson loop matrix $[\mathcal{W}_{2,\mathbf{k},\mathbf{G},\mathbf{G}'}^{\pm}]$ as $\exp\{i(\gamma_2^{\pm})_{j,\mathbf{k},\mathbf{G},\mathbf{G}'}\}$, where $j=1,\ldots,N_W^{\pm}$. We then term the set $\{(\gamma_2^{\pm})_{j,\mathbf{k},\mathbf{G},\mathbf{G}'}|j=1\ldots N_W^{\pm}\}$ as a function of $\mathbf{k}$ the "nested $P_{\pm}$-*Wannier* bands," where j is the band index. The eigenphases $\{(\gamma_2^{\pm})_{j,\mathbf{k},\mathbf{G},\mathbf{G}'}|j=1\ldots N_W^{\pm}\}$ give the positions of spin-resolved hybrid Wannier functions formed from the $N_W^{\pm}$ "occupied" $P_{\pm}$-Wannier bands and localized along the lattice vector $\mathbf{a}'$ dual to the reciprocal lattice vector $\mathbf{G}'$ [13, 14]. Notice that in general N_W^{+} can be different from N_W^{-} if there are no constraints from symmetries such as time-reversal or $SU(2)$ spin rotation symmetry. The eigenphases $(\gamma_2^{\pm})_{j,\mathbf{k},\mathbf{G},\mathbf{G}'}$ of the nested $P_{\pm}$-Wilson loop matrix [SEq. (4.47)] are also the non-Abelian Berry phases of the $N_W^{\pm}$ "occupied" $P_{\pm}$-Wannier bands for the closed loop in $\mathbf{k}$-space parallel to $\mathbf{G}'$. In particular, the eigenphases $(\gamma_2^{\pm})_{j,\mathbf{k},\mathbf{G},\mathbf{G}'}$ are independent of the momentum component $\mathbf{k}\cdot\mathbf{a}'$. In SN 6 F and 6 H we elucidate the constraints on the values of nested $P_{\pm}$-Wilson loop eigenphases due to inversion and time-reversal symmetries.

Similar to the case of nested P-Wilson loop in SN 4 A, we may deduce the topological properties of a spinful system by computing the spectral flow of $\{(\gamma_2^{\pm})_{j,\mathbf{k},\mathbf{G},\mathbf{G}'}|j=1\ldots N_W^{\pm}\}$. We will derive general properties of the spectral flow in SN 4 C and apply these to the classification and study of spin-resolved topology in HOTIs in SN 4 D. In SN 4 E we will numerically evaluate the nested $P_{\pm}$-Wilson loops for a tight-binding model of a helical HOTI, revealing the existence of previously unrecognized spin-stable partial axion angles.

C. General Properties of the Nested $P_{\pm}$-Wilson Loop Spectra

In this section, we will derive several general properties of the nested P- and $P_{\pm}$-Wilson loop [SEqs. (4.24) and (4.47)] eigenphases (nested non-Abelian Berry phases) and their associated spectral flow. These will allow us to develop a classification of spin-resolved topology in systems with inversion and time-reversal symmetry. Throughout this section, we will use "($\pm$)", to indicate that a derivation applies to both the nested P- and $P_{\pm}$-Wilson loops. Unless otherwise specified, we will assume that both the energy and PsP spectrum are gapped at every $\mathbf{k}$ throughout the BZ. We will consider 3D systems with primitive real-space lattice vectors $\{\mathbf{a}_1,\mathbf{a}_2,\mathbf{a}_3\}$ and the dual primitive reciprocal lattice vectors $\{\mathbf{G}_1,\mathbf{G}_2,\mathbf{G}_3\}$ such that $\mathbf{a}_i\cdot\mathbf{G}_j=2\pi\delta_{i,j}$ $(i,j=1\ldots3)$. The crystal momentum $\mathbf{k}$ can then be expanded using $\mathbf{k}=\sum_{i=1}^{3}\frac{k_i}{2\pi}\mathbf{G}_i$ where the momentum component $k_i=\mathbf{k}\cdot\mathbf{a}_i$. The BZ is then defined by the region with $k_i=[-\pi,\pi)$.

We begin in SN 4 C 1, by showing that the eigenphases of the nested $P_{(\pm)}$-Wilson loops [SEqs. (4.24) and (4.47)] coincide with a subset of the eigenphases of the $P_{(\pm)}$-Wilson loops [SEqs. (3.2) and (3.17)] when the directions of the first and second holonomy are parallel. Using this result, we will establish in SN 4 C 2 that although the *nested* Wilson loop eigenphases depend on two momentum components of the base point $\mathbf{k}$, the eigenphases can have a nontrivial spectral flow with a nonzero winding number along at most one momentum direction. In SN 4 C 3, we will derive an exact relation between the spectral flow of the *nested* (spin-resolved) Wilson loop eigenphases and the (partial) weak Chern numbers, which are the 3D generalizations of the (partial) Chern numbers from SEq. (3.24). While we will specialize to cases where at most one (partial) weak Chern number is nonzero, we will in SN 4 C 3 show how our results extend to more general situations. In SN 4 C 4 we will show that for inversion- and time-reversal-symmetric systems the nested $P_{\pm}$-Wilson loops exhibit a novel variant of $\mathbb{Z}_2$-stable spectral flow, from which

we classify spin-stable topology in these systems. Then, in the next SN 4 D, based on the layer-construction of helical and magnetic (higher-order) topological insulators [33, 42–44], we show how spin-stable topology refines and enriches the notion of symmetry-indicated stable band topology, and specifically reveals the existence of previously unrecognized spin-resolved (partial) axion angles in 3D insulators.

1. *Nested P- and $P_\pm$-Wilson Loops When* $\mathbf{G}=\mathbf{G}'$

In the computation of the *nested* Wilson loop eigenphases, we were required to choose two primitive reciprocal lattice vectors $\mathbf{G}$ and $\mathbf{G}'$ in SEqs. (4.24) and (4.47). $\mathbf{G}$ fixes the direction of the first Wilson loop, and so determines the localization direction of the Wannier band eigenstates; $\mathbf{G}'$ gives the direction of the second (nested) Wilson loop, computed as a product of projectors onto the $\widehat{\mathbf{G}}$-directed Wannier bands. And we refer to $\mathbf{G}$ and $\mathbf{G}'$ as the directions of the first and second closed loop holonomy. In this section, we consider the case where $\mathbf{G}'=\mathbf{G}$ and then prove that the resulting nested (spin-resolved or ordinary) Wilson loop eigenphases are the same as the centers of the (spin-resolved or ordinary) $\widehat{\mathbf{G}}$-directed hybrid Wannier functions.

We begin by denoting the projector onto the single-particle energy or PsP eigenstates as $P_{(\pm)}$, such that

$$\langle\alpha,\mathbf{k}|P_{(\pm)}|\beta,\mathbf{q}\rangle=\langle 0|c_{\alpha,\mathbf{k}}P_{(\pm)}c^\dagger_{\beta,\mathbf{q}}|0\rangle=\delta_{\mathbf{k},\mathbf{q}}[P_{(\pm)}(\mathbf{k})]_{\alpha,\beta}, \tag{4.48}$$

where $c^\dagger_{\alpha,\mathbf{k}}$ and $c_{\alpha,\mathbf{k}}$ are the Fourier-transformed creation and annihilation operators of the αth orbital defined in SEqs. (2.2) and (2.3). The matrix projector $[P_{(\pm)}(\mathbf{k})]$ in SEq. (4.48) is

$$[P_{(\pm)}(\mathbf{k})]=\sum_{n=1}^{N^{(\pm)}_{\text{occ}}}|u^{(\pm)}_{n,\mathbf{k}}\rangle\langle u^{(\pm)}_{n,\mathbf{k}}|, \tag{4.49}$$

where $|u^{(\pm)}_{n,\mathbf{k}}\rangle$ is the occupied energy eigenvector of the Bloch Hamiltonian matrix $[H(\mathbf{k})]$ [SEq. (2.11)] or the eigenvector of the reduced spin matrix $[s_{\text{reduced}}(\mathbf{k})]$ [SEq. (3.5)] in the upper/lower PsP eigenspace written in terms of the tight-binding basis states [SEq. (3.7)]. From SRefs. [4, 14], the eigenvalues of the projected position operator $P_{(\pm)}\widehat{\mathbf{x}}\cdot\mathbf{G}P_{(\pm)}$ are the $\widehat{\mathbf{G}}$-directed $P_{(\pm)}$-Wannier bands $\{(\gamma^{(\pm)}_1)_{j,\mathbf{k},\mathbf{G}}|j=1\ldots N^{(\pm)}_{\text{occ}}\}$ [SEqs. (4.2) and (4.25)], and the corresponding eigenstates are (spin-resolved or ordinary) hybrid Wannier functions in the image of $P_{(\pm)}$ that are localized along the lattice vector $\mathbf{a}$ dual to the reciprocal lattice vector $\mathbf{G}$. Provided that we have disjoint groups of $P_{(\pm)}$-Wannier bands (*i.e.*, provided there is a gap in the $P_{(\pm)}$-Wannier bands), we can form the projector $\widetilde{P}^{(\pm)}_{\mathbf{G}}$ onto a group of $N^{(\pm)}_W$ $P_{(\pm)}$-Wannier bands using the corresponding single-particle hybrid Wannier functions, such that

$$\langle\alpha,\mathbf{k}|\widetilde{P}^{(\pm)}_{\mathbf{G}}|\beta,\mathbf{q}\rangle=\langle 0|c_{\alpha,\mathbf{k}}\widetilde{P}^{(\pm)}_{\mathbf{G}}c^\dagger_{\beta,\mathbf{q}}|0\rangle=\delta_{\mathbf{k},\mathbf{q}}[\widetilde{P}^{(\pm)}_{\mathbf{G}}(\mathbf{k})]_{\alpha,\beta}. \tag{4.50}$$

The matrix projector $[\widetilde{P}^{(\pm)}_{\mathbf{G}}(\mathbf{k})]$ in SEq. (4.50) is [according to SEqs. (4.23) and (4.46)]

$$[\widetilde{P}^{(\pm)}_{\mathbf{G}}(\mathbf{k})]=\sum_{j=1}^{N^{(\pm)}_W}|w^{(\pm)}_{j,\mathbf{k},\mathbf{G}}\rangle\langle w^{(\pm)}_{j,\mathbf{k},\mathbf{G}}|, \tag{4.51}$$

where $|w^{(\pm)}_{j,\mathbf{k},\mathbf{G}}\rangle$ [SEqs. (4.6) and (4.30)] is the $\widehat{\mathbf{G}}$-directed $P_{(\pm)}$-Wannier band basis corresponding to the eigenvectors of the $\widehat{\mathbf{G}}$-directed $P_{(\pm)}$-Wilson loop matrix [SEqs. (3.2) and (3.17)] with eigenphases $\{(\gamma^{(\pm)}_1)_{j,\mathbf{k},\mathbf{G}}|j=1\ldots N^{(\pm)}_W\}$ where j indexes the "occupied" $N^{(\pm)}_W$ $P_{(\pm)}$-Wannier bands spectrally separated from the other "unoccupied" $N^{(\pm)}_{\text{occ}}-N^{(\pm)}_W$ $P_{(\pm)}$-Wannier bands.

Next, we consider the projected position operator $\widetilde{P}^{(\pm)}_{\mathbf{G}}\widehat{\mathbf{x}}\cdot\mathbf{G}\widetilde{P}^{(\pm)}_{\mathbf{G}}$, whose eigenvalues are the eigenphases of the nested $P_{(\pm)}$-Wilson loop with $\mathbf{G}'=\mathbf{G}$ [4, 14] denoted by $\{(\gamma^{(\pm)}_2)_{j,\mathbf{k},\mathbf{G},\mathbf{G}}|j=1\ldots N^{(\pm)}_W\}$ (SN 4 A and 4 B). Since $P_{(\pm)}=\widetilde{P}^{(\pm)}_{\mathbf{G}}$ on states in the image of $\widetilde{P}^{(\pm)}_{\mathbf{G}}$, and since the image of $\widetilde{P}^{(\pm)}_{\mathbf{G}}$ is spanned by a set of eigenstates of $P_{(\pm)}\widehat{\mathbf{x}}\cdot\mathbf{G}P_{(\pm)}$, we have that eigenstates of $\widetilde{P}^{(\pm)}_{\mathbf{G}}\widehat{\mathbf{x}}\cdot\mathbf{G}\widetilde{P}^{(\pm)}_{\mathbf{G}}$ are eigenstates of $P_{(\pm)}\widehat{\mathbf{x}}\cdot\mathbf{G}P_{(\pm)}$ with the same eigenvalues. In other words, we have established that $\{(\gamma^{(\pm)}_2)_{j,\mathbf{k},\mathbf{G},\mathbf{G}}|j=1\ldots N^{(\pm)}_W\}$ is the same as $\{(\gamma^{(\pm)}_1)_{j,\mathbf{k},\mathbf{G}}|j=1\ldots N^{(\pm)}_W\}$ where $j=1\ldots N^{(\pm)}_W$ index the $P_{(\pm)}$-Wannier bands considered in the computation of the nested $P_{(\pm)}$-Wilson loops.

In conclusion, we have proved that when the first and second closed-loop holonomy are in the same direction, the resulting nested $P_{(\pm)}$-Wilson loop eigenphases are the same as the corresponding set of $\widehat{\mathbf{G}}$-directed $P_{(\pm)}$-Wannier centers.

2. The Nested P- and $P_{\pm}$-Wilson Loops Wind Along at Most One Primitive Reciprocal Lattice Direction

In this section we will further analyze the winding of the nested $P_{(\pm)}$-Wilson loop eigenphases $\{(\gamma_2^{(\pm)})_{n,\mathbf{k},\mathbf{G}_i,\mathbf{G}_j}|n = 1\ldots N_W^{(\pm)}\}$ [SN 4 A and 4 B] where the first and second closed loop holonomy are parallel to $\mathbf{G}_i$ and $\mathbf{G}_j$ respectively, and we will assume that $i \neq j$ $(i,j \in (1,2,3))$ for now, as we have discussed the case of $i = j$ in SN 4 C 1. The one remaining linearly-independent primitive reciprocal lattice vector is denoted as $\mathbf{G}_l$. The total nested Berry phase $(\gamma_2^{(\pm)})_{\mathbf{k},\mathbf{G}_i,\mathbf{G}_j}$ is defined by summing over the nested $P_{(\pm)}$-Wilson loop eigenphases

$$(\gamma_2^{(\pm)})_{\mathbf{k},\mathbf{G}_i,\mathbf{G}_j} \equiv \sum_{n=1}^{N_W^{(\pm)}} (\gamma_2^{(\pm)})_{n,\mathbf{k},\mathbf{G}_i,\mathbf{G}_j} \bmod 2\pi = \mathrm{Im}\left(\log\left(\det[\mathcal{W}^{(\pm)}_{2,\mathbf{k},\mathbf{G}_i,\mathbf{G}_j}]\right)\right). \tag{4.52}$$

$(\gamma_2^{(\pm)})_{\mathbf{k},\mathbf{G}_i,\mathbf{G}_j}$ is a function of the momentum components k_i and k_l, and $[\mathcal{W}^{(\pm)}_{2,\mathbf{k},\mathbf{G}_i,\mathbf{G}_j}]$ is the nested $P_{(\pm)}$-Wilson loop matrix [SEqs. (4.24) and (4.47)]. From $(\gamma_2^{(\pm)})_{\mathbf{k},\mathbf{G}_i,\mathbf{G}_j}$, we can define two winding numbers

$$C^{(\pm)}_{\gamma_2,i,j}(k_l;i) \equiv \int_{BZ} dk_i \frac{\partial(\gamma_2^{(\pm)})_{\mathbf{k},\mathbf{G}_i,\mathbf{G}_j}}{\partial k_i} = \int_{BZ} dk_i \frac{\partial \mathrm{Im}\left(\log\left(\det[\mathcal{W}^{(\pm)}_{2,\mathbf{k},\mathbf{G}_i,\mathbf{G}_j}]\right)\right)}{\partial k_i}, \tag{4.53}$$

$$C^{(\pm)}_{\gamma_2,i,j}(k_i;l) \equiv \int_{BZ} dk_l \frac{\partial(\gamma_2^{(\pm)})_{\mathbf{k},\mathbf{G}_i,\mathbf{G}_j}}{\partial k_l} = \int_{BZ} dk_l \frac{\partial \mathrm{Im}\left(\log\left(\det[\mathcal{W}^{(\pm)}_{2,\mathbf{k},\mathbf{G}_i,\mathbf{G}_j}]\right)\right)}{\partial k_l}. \tag{4.54}$$

$C^{(\pm)}_{\gamma_2,i,j}(k_l;i)$ [SEq. (4.53)] is the winding number of the nested Berry phase $(\gamma_2^{(\pm)})_{\mathbf{k},\mathbf{G}_i,\mathbf{G}_j}$ [SEq. (4.52)] when we fix k_l and let $k_i \to k_i + 2\pi$, while $C^{(\pm)}_{\gamma_2,i,j}(k_i;l)$ [SEq. (4.54)] is the winding number of the nested Berry phase $(\gamma_2^{(\pm)})_{\mathbf{k},\mathbf{G}_i,\mathbf{G}_j}$ [SEq. (4.52)] when we fix k_i and let $k_l \to k_l + 2\pi$.

We will now prove that $C^{(\pm)}_{\gamma_2,i,j}(k_l;i)$ [SEq. (4.53)] must be zero provided that the $\widehat{\mathbf{G}}_i$-directed $P_{(\pm)}$-Wannier bands can be decomposed into disjoint sets without band touching points, which is the requirement for the nested Berry phases $(\gamma_2^{(\pm)})_{\mathbf{k},\mathbf{G}_i,\mathbf{G}_j}$ [SEq. (4.52)] to be well-defined at all (k_i,k_l).

To begin, let us denote the $\widehat{\mathbf{G}}_i$-directed $P_{(\pm)}$-Wannier bands (non-Abelian Berry phases) as $\{(\gamma_1^{(\pm)})_{n,\mathbf{k},\mathbf{G}_i}|n = 1\ldots N_{\mathrm{occ}}^{(\pm)}\}$ [SEqs. (4.2) and (4.25)]. Since $(\gamma_1^{(\pm)})_{n,\mathbf{k},\mathbf{G}_i}$ is independent of the momentum component k_i, we may view $\{(\gamma_1^{(\pm)})_{n,\mathbf{k},\mathbf{G}_i}|j = 1\ldots N_{\mathrm{occ}}^{(\pm)}\}$ as a 3D band structure that is flat as a function of k_i, with the caveat that the eigenvectors of the $P_{(\pm)}$-Wilson loop matrix must satisfy the parallel transport conditions [SEqs. (4.5) and (4.29)]. We will also assume that $\{(\gamma_1^{(\pm)})_{n,\mathbf{k},\mathbf{G}_i}|n = 1\ldots N_{\mathrm{occ}}^{(\pm)}\}$ consists of spectrally separated groups of bands so that the computation of $(\gamma_2^{(\pm)})_{\mathbf{k},\mathbf{G}_i,\mathbf{G}_j}$ is well-defined over the 2D BZ spanned by $\mathbf{G}_i$ and $\mathbf{G}_l$. Using the sign convention in SEq. (3.24) that relates the Chern number and the Berry phase winding number, if we choose (j,i,l) to index a right-handed coordinate system, $C^{(\pm)}_{\gamma_2,i,j}(k_l;i)$ indicates the *Chern number* in the k_l-constant BZ plane for the $N_W^{(\pm)}$ $\widehat{\mathbf{G}}_i$-directed, spectrally separated $P_{(\pm)}$-Wannier bands. On the other hand, the same Chern number can also be obtained by first computing the nested Berry phases

$$(\gamma_2^{(\pm)})_{\mathbf{k},\mathbf{G}_i,\mathbf{G}_i} \equiv \sum_{n=1}^{N_W^{(\pm)}} (\gamma_2^{(\pm)})_{n,\mathbf{k},\mathbf{G}_i,\mathbf{G}_i} \bmod 2\pi = \mathrm{Im}\left(\log\left(\det[\mathcal{W}^{(\pm)}_{2,\mathbf{k},\mathbf{G}_i,\mathbf{G}_i}]\right)\right), \tag{4.55}$$

which depend on the momentum components k_j and k_l. Notice that on the right-hand side of SEq. (4.55), both the first and second closed-loop holonomy are parallel to $\mathbf{G}_i$. We next obtain the corresponding winding number

$$C^{(\pm)}_{\gamma_2,i,i}(k_l;j) = \int_{BZ} dk_j \frac{\partial(\gamma_2^{(\pm)})_{\mathbf{k},\mathbf{G}_i,\mathbf{G}_i}}{\partial k_j} = \int_{BZ} dk_j \frac{\partial \mathrm{Im}\left(\log\left(\det[\mathcal{W}^{(\pm)}_{2,\mathbf{k},\mathbf{G}_i,\mathbf{G}_i}]\right)\right)}{\partial k_j}., \tag{4.56}$$

in which $C^{(\pm)}_{\gamma_2,i,i}(k_l;j)$ is the winding number of the nested Berry phase $(\gamma_2^{(\pm)})_{\mathbf{k},\mathbf{G}_i,\mathbf{G}_i}$ [SEq. (4.55)] for a fixed value of k_l and taking $k_j \to k_j + 2\pi$. Notice that using the sign convention in SEq. (3.24) that relates the Chern number and the Berry phase winding number, if we choose (i,j,l) to index a right-handed coordinate system, $C^{(\pm)}_{\gamma_2,i,i}(k_l;j)$ also indicates the *Chern number* of the $\widehat{\mathbf{G}}_i$-directed $P_{(\pm)}$ Wannier bands in a constant-k_l BZ plane. In particular,

$C^{(\pm)}_{\gamma_2,i,i}(k_l;j)$ [SEq. (4.56)] and $C^{(\pm)}_{\gamma_2,i,j}(k_l;i)$ [SEq. (4.53)] satisfy

$$C^{(\pm)}_{\gamma_2,i,j}(k_l;i) = -C^{(\pm)}_{\gamma_2,i,i}(k_l;j). \tag{4.57}$$

Next, from the discussion in SN 4 C 1, we can deduce that the nested Berry phase $(\gamma_2^{(\pm)})_{\mathbf{k},\mathbf{G}_i,\mathbf{G}_i}$ [SEq. (4.55)] is equal to $\sum_{n=1}^{N_W^{(\pm)}}(\gamma_1^{(\pm)})_{n,\mathbf{k},\mathbf{G}_i}$ mod 2π, where $n = 1\ldots N_W^{(\pm)}$ indexes the $N_W^{(\pm)}$ Wannier bands. Since we have assumed that the $N_W^{(\pm)}$ Wannier bands are spectrally separated from the other Wannier bands, the winding number of $\sum_{n=1}^{N_W^{(\pm)}}(\gamma_1^{(\pm)})_{n,\mathbf{k},\mathbf{G}_i}$ mod 2π must be zero. Since $(\gamma_2^{(\pm)})_{\mathbf{k},\mathbf{G}_i,\mathbf{G}_i} = \sum_{n=1}^{N_W^{(\pm)}}(\gamma_1^{(\pm)})_{n,\mathbf{k},\mathbf{G}_i}$ mod 2π, this then implies the winding number $C^{(\pm)}_{\gamma_2,i,i}(k_l;j)$ [SEq. (4.56)] is also zero. From the relation $C^{(\pm)}_{\gamma_2,i,j}(k_l;i) = -C^{(\pm)}_{\gamma_2,i,i}(k_l;j)$ [SEq. (4.57)], we then conclude that $C^{(\pm)}_{\gamma_2,i,j}(k_l;i)$ [SEq. (4.53)] satisfies

$$C^{(\pm)}_{\gamma_2,i,j}(k_l;i) = 0. \tag{4.58}$$

Therefore, the winding number of the nested Berry phase $(\gamma_2^{(\pm)})_{\mathbf{k},\mathbf{G}_i,\mathbf{G}_j}$ [SEq. (4.52)] must be zero if we fix k_l and let $k_i \to k_i + 2\pi$.

We emphasize that, employing the sign convention in SEq. (3.24) and choosing (i,j,l) to index a right-handed coordinate system, since the winding number $C^{(\pm)}_{\gamma_2,i,j}(k_i;l)$ [SEq. (4.54)] is equal to the *Chern number* of the $N_W^{(\pm)}$ spectrally separated $\widehat{\mathbf{G}}_i$-directed $P_{(\pm)}$-Wannier bands in a constant-k_i BZ plane, we must have that $C^{(\pm)}_{\gamma_2,i,j}(k_i;l)$ [SEq. (4.54)] is a constant independent of k_i. Therefore, the nested Berry phase $(\gamma_2^{(\pm)})_{\mathbf{k},\mathbf{G}_i,\mathbf{G}_j}$ [SEq. (4.52)] can have at most one nonzero winding number $C^{(\pm)}_{\gamma_2,i,j}(k_i;l)$ [SEq. (4.54)], and its value is independent of k_i.

To conclude, if we compute the nested Berry phase $(\gamma_2^{(\pm)})_{\mathbf{k},\mathbf{G}_i,\mathbf{G}_j}$ through SEq. (4.52) with $\mathbf{G}_i \neq \mathbf{G}_j$, even though the eigenphases depend on k_i and k_l (noting that $\mathbf{G}_i$, $\mathbf{G}_j$, $\mathbf{G}_l$ are linearly-independent), there can be no net winding as a function of k_i. As such, nonzero winding in the spectrum of $(\gamma_2^{(\pm)})_{\mathbf{k},\mathbf{G}_i,\mathbf{G}_j}$ [SEq. (4.52)] can occur only as a function of k_l, and in addition such a winding number must be independent of k_i provided that there exists a Wannier gap. Therefore, without loss of generality, when we refer to the winding number of the nested Berry phase $(\gamma_2^{(\pm)})_{\mathbf{k},\mathbf{G}_i,\mathbf{G}_j}$ [SEq. (4.52)], we exclusively are referring to the winding number $C^{(\pm)}_{\gamma_2,i,j}(k_i;l)$ [SEq. (4.54)]. As such, in later sections we will introduce a simpler notation for $C^{(\pm)}_{\gamma_2,i,j}(k_i;l)$. Lastly, we note that $\mathbf{G}_l$ is the only primitive reciprocal lattice vector that we have not yet used either for the first or the second closed-loop holonomy in the computation of the nested Berry phases $(\gamma_2^{(\pm)})_{\mathbf{k},\mathbf{G}_i,\mathbf{G}_j}$ [SEq. (4.52)].

The winding number of the nested P-Wilson loop has been shown in SRefs. [15, 16] to be $\mathbb{Z}_2$-stable, and is nontrivial for 3D strong magnetic axion insulators (*i.e.* magnetic insulators with bulk quantized Chern-Simons axion angles $\theta = \pi$ and vanishing weak Chern numbers). In particular, the winding number modulo 2 has been shown to be an indicator of θ [77, 79]. In the next section (SN 4 C 3), we will generalize this observation and derive a relationship between the winding number of the nested $P_{(\pm)}$-Wilson loop spectrum and the (partial) weak Chern number, which we will then in SN 4 D use to extract a novel spin-resolved (partial) variant of the axion angle.

3. *Relation Between Nested P- and $P_\pm$-Wilson Loops and (Partial) Weak Chern Numbers*

In this section, we will derive exact relations between the winding number of the nested (spin-resolved) Wilson loop eigenphases and the (partial) weak Chern numbers, which are the 3D generalization of the (partial) Chern numbers defined in SEq. (3.24). To be specific, we here consider the nested Berry phases $(\gamma_2^{(\pm)})_{\mathbf{k},\mathbf{G}_i,\mathbf{G}_j}$ in SEq. (4.52), where the first and second holonomy are parallel to the linearly-independent primitive reciprocal lattice vectors $\mathbf{G}_i$ and $\mathbf{G}_j$, respectively, and the one remaining linearly-independent primitive reciprocal lattice vector is denoted as $\mathbf{G}_l$. In order for $(\gamma_2^{(\pm)})_{\mathbf{k},\mathbf{G}_i,\mathbf{G}_j}$ to be well-defined over the 2D BZ spanned by $\mathbf{G}_i$ and $\mathbf{G}_l$, we also assume that the $\widehat{\mathbf{G}}_i$-directed $P_{(\pm)}$-Wannier bands described by $\{(\gamma_1^{(\pm)})_{j,\mathbf{k},\mathbf{G}_i} | j = 1\ldots N_{\text{occ}}^{(\pm)}\}$ contain spectrally separated $N_{\text{group}}^{(\pm)}$ groups of bands. As previously proved in SN 4 C 2, the winding number of the nested Berry phase $(\gamma_2^{(\pm)})_{\mathbf{k},\mathbf{G}_i,\mathbf{G}_j}$ for a given group of $P_{(\pm)}$-Wannier bands can only be nonzero if computed as as a function of k_l. We will hence in this section specifically focus on the winding number in SEq. (4.54) of the nested Berry phase $(\gamma_2^{(\pm)})_{\mathbf{k},\mathbf{G}_i,\mathbf{G}_j}$ computed as a function of k_l for a given group of $P_{(\pm)}$-Wannier bands.

To begin, we can decompose the matrix projector $[P_{(\pm)}(\mathbf{k})]$ onto the occupied energy bands or the upper/lower spin bands into

$$[P_{(\pm)}(\mathbf{k})] = \sum_{n=1}^{N^{(\pm)}_{\text{group}}} [\widetilde{P}^{(\pm)}_{\mathbf{G}_i,n}(\mathbf{k})], \tag{4.59}$$

where

$$[\widetilde{P}^{(\pm)}_{\mathbf{G}_i,n}(\mathbf{k})] = \sum_{j\in n^{\text{th}}\text{ group}} |w^{(\pm)}_{j,\mathbf{k},\mathbf{G}_i}\rangle\langle w^{(\pm)}_{j,\mathbf{k},\mathbf{G}_i}| \tag{4.60}$$

is the matrix projector [SEqs. (4.23) and (4.46)] formed from the $\widehat{\mathbf{G}}_i$-directed $P_{(\pm)}$-Wannier band basis $|w^{(\pm)}_{j,\mathbf{k},\mathbf{G}_i}\rangle$ [SEqs. (4.6) and (4.30)] in the nth ($n = 1\ldots N^{(\pm)}_{\text{group}}$) group of $\widehat{\mathbf{G}}_i$-directed $P_{(\pm)}$-Wannier bands. In addition, we also have the orthogonality conditions

$$[\widetilde{P}_{\mathbf{G}_i,n_1}(\mathbf{k})][\widetilde{P}_{\mathbf{G}_i,n_2}(\mathbf{k})] = \delta_{n_1,n_2}[\widetilde{P}_{\mathbf{G}_i,n_1}(\mathbf{k})] \tag{4.61}$$

for the projectors onto the subspaces within the image of $[P(\mathbf{k})]$, and

$$[\widetilde{P}^{\sigma_1}_{\mathbf{G}_i,n_1}(\mathbf{k})][\widetilde{P}^{\sigma_2}_{\mathbf{G}_i,n_2}(\mathbf{k})] = \delta_{\sigma_1,\sigma_2}\delta_{n_1,n_2}[\widetilde{P}^{\sigma_1}_{\mathbf{G}_i,n_1}(\mathbf{k})] \tag{4.62}$$

for the projectors onto the subspaces within the image of $[P_\pm(\mathbf{k})]$ where σ_1, $\sigma_2 = \pm$ and n_1, n_2 denotes the group.

Since we have assumed that the energy and PsP spectrum are gapped throughout the 3D BZ, we can define (partial) weak Chern numbers $\nu_1^{(\pm)}$, $\nu_2^{(\pm)}$, and $\nu_3^{(\pm)}$. $\{\nu_1,\nu_2,\nu_3\}$ specifically denote the weak Chern numbers of the occupied energy bands, while $\{\nu_1^\pm,\nu_2^\pm,\nu_3^\pm\}$ are the partial weak Chern numbers of the upper $(+)$ and lower $(-)$ spin bands. To be precise, $\nu_1^{(\pm)}$, $\nu_2^{(\pm)}$, and $\nu_3^{(\pm)}$ are the Chern numbers of the single-particle states in the image of $[P_{(\pm)}(\mathbf{k})]$ in BZ planes of constant-k_1, constant-k_2, and constant-k_3, respectively. We note that in the literature, the term "weak indices" is generally used to refer to topological invariants computed in the $k_i = \pi$ BZ planes [33, 43, 44, 141, 204, 205]. However in systems with an energy and a spin gap, the (partial) Chern numbers cannot change as functions of k_i (which would require energy or spin gap-closing points), and hence the (partial) Chern numbers are constant and independent of k_i. Since we require the $\widehat{\mathbf{G}}_i$-directed $P_{(\pm)}$-Wannier bands to consist of disjoint groups that are spectrally separated, we must have that $\nu_j^{(\pm)} = \nu_l^{(\pm)} = 0$. Conversely, $\nu_i^{(\pm)}$ can take any integral values. The partial weak Chern numbers ν_i^+ and ν_i^- are generically independent, but can be related to each other by symmetries that reverse the spin direction, as we will see below. Recall that throughout this section, we are employing a notation in which i, j, l to denote the three linearly-independent primitive reciprocal lattice vectors $\mathbf{G}_i$, $\mathbf{G}_j$, and $\mathbf{G}_l$. We can express the (partial) weak Chern number $\nu_i^{(\pm)}$ from $[P_{(\pm)}(\mathbf{k})]$ [SEq. (4.59)] computed in a BZ plane of constant-k_i as

$$\nu_i^{(\pm)} = -\frac{i}{2\pi}\int_{\substack{2D\ BZ\\ k_i=const}} dk_j dk_l \,\mathrm{Tr}\left([P_{(\pm)}(\mathbf{k})]\left[\frac{\partial[P_{(\pm)}(\mathbf{k})]}{\partial k_j},\frac{\partial[P_{(\pm)}(\mathbf{k})]}{\partial k_l}\right]\right). \tag{4.63}$$

Similarly, the (partial) Chern number of the nth group of the $\widehat{\mathbf{G}}_i$-directed $P_{(\pm)}$-Wannier bands in a constant-k_i BZ plane can be obtained through

$$C^{(\pm)}_{\gamma_2,n} = -\frac{i}{2\pi}\int_{\substack{2D\ BZ\\ k_i=const}} dk_j dk_l \,\mathrm{Tr}\left([\widetilde{P}^{(\pm)}_{\mathbf{G}_i,n}(\mathbf{k})]\left[\frac{\partial[\widetilde{P}^{(\pm)}_{\mathbf{G}_i,n}(\mathbf{k})]}{\partial k_j},\frac{\partial[\widetilde{P}^{(\pm)}_{\mathbf{G}_i,n}(\mathbf{k})]}{\partial k_l}\right]\right), \tag{4.64}$$

in which we have chosen (j,l,i) to index a right-handed coordinate system. $C^{(\pm)}_{\gamma_2,n}$ in SEq. (4.64) is identical to the winding number $C^{(\pm)}_{\gamma_2,i,j}(k_i;l)$ defined in SEq. (4.54) provided that, as specified above, (j,l,i) align with the right-handed coordinate system in SEq. (4.54). The fact that different groups of $\widehat{\mathbf{G}}_i$-directed $P_{(\pm)}$-Wannier bands are spectrally separated allows us to deduce that $C^{(\pm)}_{\gamma_2,n}$ in SEq. (4.64) does not depend on the choice of constant-k_i BZ plane in which we perform the calculation on the right-hand side of SEq. (4.64), as is discussed in SN 4 C 2.

Combining SEqs. (4.59), (4.61), (4.62), (4.63), and (4.64), it follows in analogy with SEq. (3.25) that [154]

$$\nu_i^{(\pm)} = \sum_{n=1}^{N^{(\pm)}_{\text{group}}} C^{(\pm)}_{\gamma_2,n}. \tag{4.65}$$

This implies that the sum of the nested (partial) Chern numbers $C^{(\pm)}_{\gamma_2,n}$ [SEq. (4.64)] over all of the $N^{(\pm)}_{\text{group}}$ groups of $P_{(\pm)}$-Wannier bands gives the (partial) weak Chern number of the occupied energy bands (or the upper/lower spin bands). In addition, from $[P(\mathbf{k})] = [P_+(\mathbf{k})] + [P_-(\mathbf{k})]$ where $[P_+(\mathbf{k})][P_-(\mathbf{k})] = 0$, it also follows that

$$\nu_i = \nu_i^+ + \nu_i^-, \tag{4.66}$$

such that the total weak Chern number of the occupied bands is the sum of the partial weak Chern numbers of the upper and lower spin bands. Before we apply SEq. (4.65) to specific cases, we pause to briefly restate the essential assumptions made in the above derivations:

1. The energy and spin gaps are open throughout the 3D BZ.
2. The $\widehat{\mathbf{G}}_i$-directed $P_{(\pm)}$-Wannier bands can be separated into disjoint groups such that the decomposition in SEq. (4.59) is well-defined and smooth over the 3D BZ, which implies that two of the (partial) weak Chern numbers are zero, namely $\nu_j^{(\pm)} = \nu_l^{(\pm)} = 0$. However, we note that $\nu_j^{(\pm)} = \nu_l^{(\pm)} = 0$ does not imply that the $\widehat{\mathbf{G}}_i$-directed $P_{(\pm)}$-Wannier bands can be separated into disjoint groups.

Let us now consider some specific implications of SEq. (4.65). First, consider a spinful system with time-reversal symmetry, and gaps in both the energy and PsP spectrum at every $\mathbf{k}$ point throughout the BZ. The weak Chern number of the occupied bands at any constant-k_i plane must be zero, which implies that $\nu_i = 0$. From SEq. (4.66), this further implies that

$$\nu_i^+ = -\nu_i^- \tag{4.67}$$

in the presence of spinful time-reversal symmetry. Then, if we have $\nu_i^{\pm} = \pm m \neq 0$ where $m \in \mathbb{Z}$, we can deduce that

$$\sum_{n=1}^{N^{\pm}_{\text{group}}} C^{\pm}_{\gamma_2,n} = \pm m. \tag{4.68}$$

This case occurs, for example, in the 3D quantum spin Hall insulator considered in SRef. [75], in which both the energy and spin gaps are open, and the partial weak Chern numbers are $\nu_1^{\pm} = \pm 2$, $\nu_2^{\pm} = \nu_3^{\pm} = 0$.

Next, consider a 3D insulator with an energy gap and $\nu_i = 0$, or a 3D insulator with energy and spin gaps and $\nu_i^{\pm} = 0$. The former can be realized, for instance, by a 3D magnetic axion insulator [15], and the latter can be realized by a time-reversal-symmetric helical HOTI, as we will see later in SN 4 D. The vanishing (partial) weak Chern number in either case implies that

$$\sum_{n=1}^{N^{(\pm)}_{\text{group}}} C^{(\pm)}_{\gamma_2,n} = 0. \tag{4.69}$$

If we then choose to decompose the $\widehat{\mathbf{G}}_i$-directed $P_{(\pm)}$-Wannier bands into two groups (which is a convenient choice if the system has inversion symmetry, as will be discussed in detail in SN 4 C 4), we have that the winding numbers $C^{(\pm)}_{\gamma_2,1^{\text{st}}\text{ group}}$ and $C^{(\pm)}_{\gamma_2,2^{\text{nd}}\text{ group}}$ are opposite to each other:

$$C^{(\pm)}_{\gamma_2,1^{\text{st}}\text{ group}} = -C^{(\pm)}_{\gamma_2,2^{\text{nd}}\text{ group}}. \tag{4.70}$$

Now that we have enumerated useful properties of nested $P_{(\pm)}$-Wilson loops, we can proceed to use them as tools for diagnosing (spin-resolved) band topology. In particular, in the next section we will show that in inversion- and time-reversal symmetric systems, the winding number of the nested $P_{\pm}$-Wilson loop is a $\mathbb{Z}_2$-valued invariant that characterizes properties of a system that are robust as long as neither an energy gap nor a spin gap closes (termed "spin stability" in this work). This will allow us to deduce the existence of nonzero spin-electromagnetic response coefficients in 3D helical HOTIs from their spin-resolved electronic band topology.

As a final remark, we will now generalize our analysis to cases where all of the (partial) weak Chern numbers can be nonzero, provided that the $P_{(\pm)}$-Wannier bands can be divided into disjoint groupings. Using the (partial) weak Chern numbers $\{\nu_1^{(\pm)}, \nu_2^{(\pm)}, \nu_3^{(\pm)}\}$ and the primitive reciprocal lattice vectors $\{\mathbf{G}_1, \mathbf{G}_2, \mathbf{G}_3\}$, we can construct a (partial) Chern vector [92, 206–208]

$$\boldsymbol{\nu}^{(\pm)} = \nu_1^{(\pm)}\mathbf{G}_1 + \nu_2^{(\pm)}\mathbf{G}_2 + \nu_3^{(\pm)}\mathbf{G}_3 \tag{4.71}$$

which is related (for weak Chern numbers in the energy spectrum) to the Hall conductivity via

$$\sigma_{ij}^{H} = \frac{e^2}{h}(\boldsymbol{\nu})_k \epsilon_{kij}, \tag{4.72}$$

or (for partial weak Chern numbers in the PsP spectrum) to the topological contribution to the spin Hall conductivity [$C^{s}_{\gamma_1}$ in SEq. (3.36)] via

$$\sigma_{ij,\mathrm{top}}^{s} = \frac{e}{4\pi}(\boldsymbol{\nu}^{+} - \boldsymbol{\nu}^{-})_k \epsilon_{kij}. \tag{4.73}$$

In SEqs. (4.72) and (4.73), $i,j = x,y,z$ index *Cartesian* directions, and ϵ_{ijk} is the antisymmetric Levi-Civita symbol. From the primitive position-space lattice vectors $\{\mathbf{a}_1, \mathbf{a}_2, \mathbf{a}_3\}$, we can form a (non-unique) set of supercell position-space lattice vectors $\{\mathbf{a}_1^{sc}, \mathbf{a}_2^{sc}, \mathbf{a}_3^{sc}\}$ where

$$\mathbf{a}_1^{sc} = \nu_1^{(\pm)}\mathbf{a}_3 - \nu_3^{(\pm)}\mathbf{a}_1, \tag{4.74}$$

$$\mathbf{a}_2^{sc} = \nu_2^{(\pm)}\mathbf{a}_3 - \nu_3^{(\pm)}\mathbf{a}_2, \tag{4.75}$$

$$\mathbf{a}_3^{sc} = \mathbf{a}_3, \tag{4.76}$$

are all linear combinations of primitive lattice vectors $\{\mathbf{a}_1, \mathbf{a}_2, \mathbf{a}_3\}$ with integral coefficients. A semi-infinite slab formed from the supercell with finite thickness along $\mathbf{a}_3^{sc}$ has a surface normal vector parallel to the (partial) Chern vector $\boldsymbol{\nu}^{(\pm)}$ since [78]

$$\mathbf{a}_1^{sc} \cdot \boldsymbol{\nu}^{(\pm)} = \mathbf{a}_2^{sc} \cdot \boldsymbol{\nu}^{(\pm)} = 0. \tag{4.77}$$

Assuming without loss of generality that the (partial) weak Chern number $\nu_3^{(\pm)} \neq 0$ [if not we can always permute our coordinates to facilitate this provided that $\boldsymbol{\nu}^{(\pm)} \neq \mathbf{0}$ in SEq. (4.71)], the dual supercell primitive reciprocal lattice vectors are $\{\mathbf{G}_1^{sc}, \mathbf{G}_2^{sc}, \mathbf{G}_3^{sc}\}$ where

$$\mathbf{G}_1^{sc} = -\frac{1}{\nu_3^{(\pm)}}\mathbf{G}_1, \tag{4.78}$$

$$\mathbf{G}_2^{sc} = -\frac{1}{\nu_3^{(\pm)}}\mathbf{G}_2, \tag{4.79}$$

$$\mathbf{G}_3^{sc} = \frac{\nu_1^{(\pm)}}{\nu_3^{(\pm)}}\mathbf{G}_1 + \frac{\nu_2^{(\pm)}}{\nu_3^{(\pm)}}\mathbf{G}_2 + \mathbf{G}_3. \tag{4.80}$$

Recall that the (partial) Chern vector is a property of a set of occupied states in a 3D translationally-invariant system, which is independent of the description whether we use the primitive lattice vectors $\{\mathbf{a}_1, \mathbf{a}_2, \mathbf{a}_3\}$ or the supercell lattice vectors $\{\mathbf{a}_1^{sc}, \mathbf{a}_2^{sc}, \mathbf{a}_3^{sc}\}$. Therefore, the (partial) Chern vector of the 3D translationally-invariant system described by the supercell lattice vectors $\{\mathbf{a}_1^{sc}, \mathbf{a}_2^{sc}, \mathbf{a}_3^{sc}\}$ is the same as SEq. (4.71). To obtain the (partial) weak Chern numbers of the supercell system, we rewrite the (partial) Chern vector $\boldsymbol{\nu}^{(\pm)}$ [SEq. (4.71)] in terms of $\{\mathbf{G}_1^{sc}, \mathbf{G}_2^{sc}, \mathbf{G}_3^{sc}\}$ and obtain

$$\begin{aligned}\boldsymbol{\nu}^{(\pm)} &= \nu_1^{(\pm),sc}\mathbf{G}_1^{sc} + \nu_2^{(\pm),sc}\mathbf{G}_2^{sc} + \nu_3^{(\pm),sc}\mathbf{G}_3^{sc} \\ &= \nu_3^{(\pm)}\mathbf{G}_3^{sc}.\end{aligned} \tag{4.81}$$

SEq. (4.81) implies that the supercell (partial) weak Chern numbers $\{\nu_1^{(\pm),sc}, \nu_2^{(\pm),sc}, \nu_3^{(\pm),sc}\}$ of the 3D translationally-invariant system whose Bravais lattice vectors are $\{\mathbf{a}_1^{sc}, \mathbf{a}_2^{sc}, \mathbf{a}_3^{sc}\}$ are given by

$$\nu_1^{(\pm),sc} = 0, \quad \nu_2^{(\pm),sc} = 0, \quad \nu_3^{(\pm),sc} = \nu_3^{(\pm)}. \tag{4.82}$$

Therefore, provided that the (partial) Chern vector $\boldsymbol{\nu}^{(\pm)}$ in SEq. (4.71) is a nonzero vector, we can choose a supercell spanned by $\{\mathbf{a}_1^{sc}, \mathbf{a}_2^{sc}, \mathbf{a}_3^{sc}\}$ such that only one of the supercell (partial) weak Chern numbers is nonzero . As such, the results derived in this section can be directly generalized and applied to the supercell system to obtain the relation between the supercell (partial) weak Chern numbers $\nu_3^{(\pm),sc}$ and the winding numbers of the nested $P_{(\pm)}$-Wilson loop spectra, provided that the $\widehat{\mathbf{G}}_3^{sc}$-directed $P_{(\pm)}$-Wannier bands of the supercell system contain disjoint groupings. We emphasize again that different choices of the non-unique supercell Bravais lattice vectors can lead to distinct supercell

(partial) weak Chern numbers. However, physical observables like the (spin) Hall conductivity [SEqs. (4.72) and (4.73)] only depend on the invariant (partial) Chern vector [SEq. (4.71)]. As long as one can obtain a set of supercell lattice vectors for which the $P_{(\pm)}$-Wannier bands of the supercell system computed along one of the supercell reciprocal lattice vectors can be separated into disjoint groupings such that two of the supercell (partial) weak Chern numbers are zero, the results derived in this section can be directly extended to the relation between the supercell (partial) weak Chern numbers and the winding numbers of the nested $P_{(\pm)}$-Wilson loop spectra of the supercell system.

4. $\mathbb{Z}_2$-Stable Spectral Flow in the Nested $P_\pm$-Wilson Loop Eigenphases in the Presence of Bulk $\mathcal{I}$ and Spinful $\mathcal{T}$ Symmetries, and the Corresponding Spin-Stable Bulk Topology

In this section, we will show that with inversion ($\mathcal{I}$) and time-reversal ($\mathcal{T}$) symmetry, the spectral flow of the nested Berry phases in the positive and negative PsP eigenspaces is $\mathbb{Z}_2$-stable to deformations of the spin-resolved Wannier band structure, provided that both the energy and spin gap remain open throughout the deformation. To be specific, as in SN 4 C 3, we consider the nested Berry phases in SEq. (4.52) where the first and second closed-loop holonomy in the computation of the nested Berry phases are parallel to $\mathbf{G}_i$ and $\mathbf{G}_j$ ($i \neq j$), respectively. We again denote the one remaining linearly-independent primitive reciprocal lattice vector as $\mathbf{G}_l$. As was previously proved in SN 4 C 2, the winding numbers of the nested Berry phases [SEq. (4.52)] for a given group of Wannier bands can only have nonzero winding as a function of k_l. We will thus in this section exclusively consider the winding numbers of the nested Berry phases [SEq. (4.52)] for a given group of Wannier bands as a function of k_l.

To begin, we consider 3D insulators with $\mathcal{I}$ and $\mathcal{T}$ symmetry whose energy and spin spectra are both gapped. As shown in SN 2 B, in the presence of $\mathcal{I}\mathcal{T}$ symmetry, the upper (+) and lower (−) eigenspace of PsP are the positive and negative eigenspace of PsP. In the presence of $\mathcal{I}$ symmetry, the $\widehat{\mathbf{G}}_i$-directed $P_\pm$-Wannier band eigenphases $(\gamma_1^\pm)_{j,\mathbf{k},\mathbf{G}_i}$ satisfy

$$\{(\gamma_1^\pm)_{j,\mathbf{k},\mathbf{G}_i} | j = 1 \ldots N_{\text{occ}}^\pm\} = \{-(\gamma_1^\pm)_{j,-\mathbf{k},\mathbf{G}_i} | j = 1 \ldots N_{\text{occ}}^\pm\}, \tag{4.83}$$

and in the presence of $\mathcal{T}$ symmetry, we have

$$\{(\gamma_1^\pm)_{j,\mathbf{k},\mathbf{G}_i} | j = 1 \ldots N_{\text{occ}}^\pm\} = \{(\gamma_1^\mp)_{j,-\mathbf{k},\mathbf{G}_i} | j = 1 \ldots N_{\text{occ}}^\pm\}, \tag{4.84}$$

where $(\gamma_1^\pm)_{j,\mathbf{k},\mathbf{G}_i}$ is defined modulo 2π. Note that $(\gamma_1^\pm)_{j,\mathbf{k},\mathbf{G}_i}$ depends only on the momentum components k_j and k_l. We refer the readers to SN 6 E and 6 G for the detailed proof of SEqs. (4.83) and (4.84), respectively.

Assuming that there a gap in the $P_\pm$-Wannier bands, the inversion symmetry constraint in SEq. (4.83) allows us to divide the $P_\pm$-Wannier bands into two disjoint groups—one centered around $\gamma_1^\pm = 0$, which we term the "inner set", and another around $\gamma_1^\pm = \pi$ (or equivalently $-\pi$), which we term the "outer set" [13–16, 18, 19, 31, 174]. A schematic example is shown in SFig. 17(a,e). The inner and outer sets of spin-resolved ($P_\pm$-) Wannier bands are effectively inversion-symmetric in the sense that they individually satisfy SEq. (4.83). In the following, we will use interchangeably the "winding number of the nested Barry phase $(\gamma_2^\pm)_{\mathbf{k},\mathbf{G}_i,\mathbf{G}_j}$ [SEq. (4.52)]", and the term "nested partial Chern number [SEq. (4.64)]", where the sign convention in SEq. (3.24) has been implicitly applied. From SEqs. (4.65) and (4.68) in SN 4 C 3, we have that the nested partial Chern numbers for a system with time-reversal symmetry satisfy

$$C_{\gamma_2,in}^\pm + C_{\gamma_2,out}^\pm = \nu_i^\pm = \pm m, \tag{4.85}$$

where $\nu_i^\pm$ is the partial weak Chern number (defined in SN 4 C 3) of the positive/negative spin bands at constant-k_i planes, $m \in \mathbb{Z}$, and we have used "in" and "out" to denote the disjoint inner and outer groups of spin-resolved Wannier bands. As is discussed in SN 4 C 3, by construction, we are here specializing (without loss of generality) to systems in which the other two partial weak Chern numbers $\nu_j^\pm$ and $\nu_l^\pm$ are zero due to the fact that the $\widehat{\mathbf{G}}_i$-directed $P_\pm$-Wannier bands contain disjoint groups.

In the presence of $\mathcal{T}$ symmetry, since the nested Berry phases $(\gamma_2^\pm)_{\mathbf{k},\mathbf{G}_i,\mathbf{G}_j}$ for a given group of $P_\pm$-Wannier bands satisfy $(\gamma_2^\pm)_{\mathbf{k},\mathbf{G}_i,\mathbf{G}_j} \bmod 2\pi = (\gamma_2^\mp)_{-\mathbf{k},\mathbf{G}_i,\mathbf{G}_j} \bmod 2\pi$ (we refer the readers to SN 6 H for a detailed proof), we can deduce that the nested partial Chern numbers [SEq. (4.64)] must satisfy

$$C_{\gamma_2,in}^\pm = -C_{\gamma_2,in}^\mp, \tag{4.86}$$

$$C_{\gamma_2,out}^\pm = -C_{\gamma_2,out}^\mp. \tag{4.87}$$

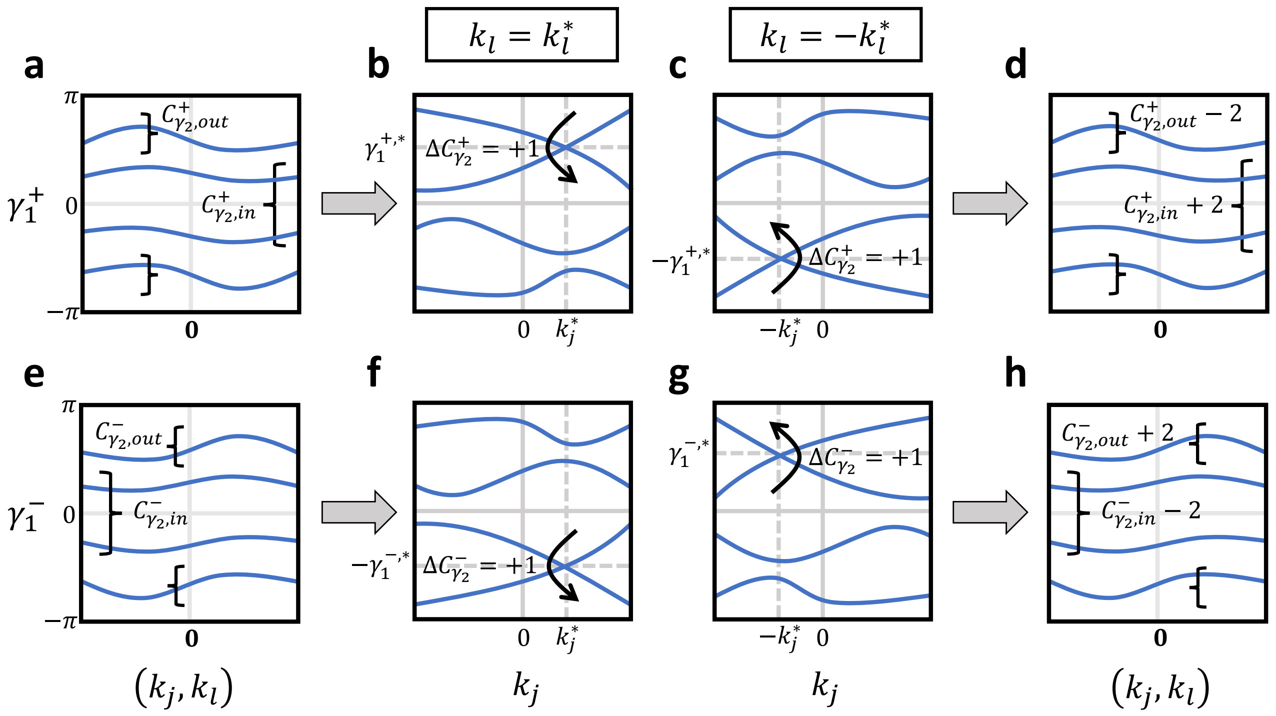

Supplementary Figure 17. Effect of Wannier gap closings on the nested $P_\pm$-Wilson loop spectrum in the presence of spinful time-reversal and 3D inversion symmetries. (a)–(d) schematically show a deformation of the Hamiltonian that induces P_+-Wannier band gap closings between the inner and outer set of P_+-Wannier bands. The outer set of P_+-Wannier bands transfers $+2$ partial Chern number to the inner set of P_+-Wannier bands via the two P_+-Wannier band gap closings in (b) and (c) related by inversion symmetry. After the deformation, the winding numbers of the nested Berry phases in the P_+-eigenphase change according to $C^+_{\gamma_2,in} \to C^+_{\gamma_2,in} + 2$ and $C^+_{\gamma_2,out} \to C^+_{\gamma_2,out} - 2$, as indicated in (a) and (d). (e)–(h) show the corresponding deformation of the P_--Wannier bands related to (a)–(d) by the operation of time-reversal. In particular, (a) and (e), (b) and (g), (c) and (f), (d) and (h) are related to each other by time-reversal. The inner set of P_--Wannier bands transfers $+2$ partial Chern number to the outer set of P_--Wannier bands via the two P_--Wannier band gap closings in (f) and (g) related by inversion symmetry. After the deformation, the winding numbers of the nested Berry phases in the P_--eigenspace change according to $C^-_{\gamma_2,in} \to C^-_{\gamma_2,in} - 2$ and $C^-_{\gamma_2,out} \to C^-_{\gamma_2,out} + 2$, as indicated in (e) and (h). Both (b) and (f) are taken in the $k_l = k_l^*$ plane, and both (c) and (g) are in the $k_l = -k_l^*$ plane, in which the $P_\pm$-Wannier band gap closes during the deformation.

Similar to SN 3 C, we may then define the *relative winding numbers* of the nested Berry phases between the positive and negative PsP eigenspaces as

$$C^s_{\gamma_2,in} \equiv C^+_{\gamma_2,in} - C^-_{\gamma_2,in}, \tag{4.88}$$

$$C^s_{\gamma_2,out} \equiv C^+_{\gamma_2,out} - C^-_{\gamma_2,out}. \tag{4.89}$$

We will also call $C^s_{\gamma_2,in}$ and $C^s_{\gamma_2,out}$ as the *nested spin Chern numbers*. From SEqs. (4.86) and (4.87), in the presence of $\mathcal{T}$ symmetry, we have

$$C^s_{\gamma_2,in} = 2C^+_{\gamma_2,in} = -2C^-_{\gamma_2,in}, \tag{4.90}$$

$$C^s_{\gamma_2,out} = 2C^+_{\gamma_2,out} = -2C^-_{\gamma_2,out}. \tag{4.91}$$

In addition, since the partial weak Chern number $\nu_i^+ = -\nu_i^-$ in the presence of $\mathcal{T}$ symmetry, SEqs. (4.85), (4.90), and (4.91) also imply that

$$C^s_{\gamma_2,in} + C^s_{\gamma_2,out} = 2\nu_i^+ = -2\nu_i^-. \tag{4.92}$$

From SEqs. (4.85), (4.86), and (4.87), we see that the four winding numbers $C^\pm_{\gamma_2,in}$ and $C^\pm_{\gamma_2,out}$ of the nested Berry phases are fully specified given any of the four winding numbers and the partial weak Chern number

$$\nu_i^\pm = \pm m \in \mathbb{Z}. \tag{4.93}$$

To be explicit, using SEqs. (4.85), (4.86), (4.87), and (4.93), it can be shown that if

$$C^{+}_{\gamma_2,in} = C \in \mathbb{Z}, \tag{4.94}$$

then

$$C^{+}_{\gamma_2,out} = -C + m, \tag{4.95}$$

$$C^{-}_{\gamma_2,in} = -C, \tag{4.96}$$

$$C^{-}_{\gamma_2,out} = C - m, \tag{4.97}$$

which from SEqs. (4.90) and (4.91) also imply that the nested spin Chern numbers are

$$C^{s}_{\gamma_2,in} = 2C, \tag{4.98}$$

$$C^{s}_{\gamma_2,out} = -2C + 2m. \tag{4.99}$$

As an example, we can consider an inversion-symmetric 3D quantum spin Hall insulator with s_z conservation and $\nu_i^{\pm} = \pm 2$ [75], as will be detailed in SN 4 D. If there is a Wannier gap, then such a system can have $C^{+}_{\gamma_2,in} = +1$, from which we can deduce that $C^{+}_{\gamma_2,out} = +1$, $C^{-}_{\gamma_2,in} = -1$, $C^{-}_{\gamma_2,out} = -1$, $C^{s}_{\gamma_2,in} = +2$, and $C^{s}_{\gamma_2,out} = +2$. We will show in SN 4 D that this configuration of nested partial Chern numbers can be realized through a layer construction with two 2D quantum spin Hall insulators in each unit cell. For introductions to and further discussions of the layer-construction method for enumerating and analyzing symmetry-protected topological states, interested readers may consult SRefs. [20, 33, 42–44, 209]. Another example of a system that has an open spin gap and an open energy gap is a 3D helical higher-order topological insulator (HOTI) [31] with $\nu_i^{\pm} = 0$ and $C^{+}_{\gamma_2,in} = +1$, which can also be obtained from a layer construction (detailed in SN 4 D and SRefs. [33, 210]) and will be the main numerical focus in SN 4 E. $\nu_i^{\pm} = 0$ and $C^{+}_{\gamma_2,in} = +1$ implies that for this helical HOTI $C^{+}_{\gamma_2,out} = -1$, $C^{-}_{\gamma_2,in} = -1$, $C^{-}_{\gamma_2,out} = +1$, $C^{s}_{\gamma_2,in} = +2$, and $C^{s}_{\gamma_2,out} = -2$. We will show in SN 4 D that $C^{\pm}_{\gamma_2,out} = \mp 1$ and $C^{\pm}_{\gamma_2,in} = \pm 1$ can be realized via a layer construction by placing one $\mathcal{I}$- and spinful $\mathcal{T}$-symmetric 2D TI with partial Chern numbers $C^{\pm}_{\gamma_1} = \pm 1$ in the $\mathcal{I}$-symmetric planes containing the origin of each unit cell, along with placing one $\mathcal{I}$- and spinful $\mathcal{T}$-symmetric 2D TI with partial Chern numbers $C^{\pm}_{\gamma_1} = \mp 1$ in the inversion-symmetric planes at the boundary of each unit cell (defined to be a half-lattice translation from the origin) [18, 19, 31]. We will explicitly demonstrate this in SN 4 E through a numerical example implementing our nested spin-resolved Wilson loop formalism.

Recall from SEq. (4.64) that the winding numbers of the nested Berry phases in the $P_{\pm}$-eigenspaces are identical to the partial Chern numbers of the $P_{\pm}$-Wannier bands. Let us now consider a deformation of the Hamiltonian that preserves $\mathcal{I}$ and spinful $\mathcal{T}$ symmetries and keeps the energy and spin gaps open. Focusing on the P_{+}-Wannier bands, from SEq. (4.83), if there is a Wannier gap closing between the inner and outer set of bands at $\gamma_1^{+,*} = \gamma$ and crystal momentum (k_j^*, k_l^*) [see SFig. 17(b)], there must be another gap closing at $-\gamma_1^{+,*} = -\gamma$ and crystal momentum $(-k_j^*, -k_l^*)$ [see SFig. 17(c)] related by inversion symmetry. These two gap closings transfer the same partial Chern numbers between the inner and outer set of P_{+}-Wannier bands, such that after the deformation, the winding numbers can change according to

$$C^{+}_{\gamma_2,in} \to C^{+}_{\gamma_2,in} + 2n, \tag{4.100}$$

$$C^{+}_{\gamma_2,out} \to C^{+}_{\gamma_2,out} - 2n, \tag{4.101}$$

where $n \in \mathbb{Z}$. As an example, in SFig. 17(a–d) we schematically show the case of SEqs. (4.100) and (4.101) with $n = 1$.

On the other hand, due to $\mathcal{T}$ symmetry, SEq. (4.84) implies that simultaneously there will be also be a pair of gap closings at $\pm\gamma_1^{-,*} = \pm\gamma$ and crystal momentum $(\mp k_j^*, \mp k_l^*)$ between the inner and outer set of P_{-}-Wannier bands, see for instance SFig. 17(f,g). Since this pair of band crossings in the P_{-}-Wannier bands are related to the previous pair of band crossings in the P_{+}-Wannier bands by time-reversal, we deduce that after the deformation, we have

$$C^{-}_{\gamma_2,in} \to C^{-}_{\gamma_2,in} - 2n, \tag{4.102}$$

$$C^{-}_{\gamma_2,out} \to C^{-}_{\gamma_2,out} + 2n. \tag{4.103}$$

As an example, in SFig. 17(e–h) we have schematically shown the case of SEqs. (4.102) and (4.103) with $n = 1$.

From SEqs. (4.100), (4.101), (4.102), and (4.103), the relative winding numbers $C^{s}_{\gamma_2,in/out}$, which are even numbers from SEqs. (4.90) and (4.91), will also change according to

$$C^{s}_{\gamma_2,in} \to C^{s}_{\gamma_2,in} + 4n, \tag{4.104}$$

$$C^{s}_{\gamma_2,out} \to C^{s}_{\gamma_2,out} - 4n. \tag{4.105}$$

In particular SEqs. (4.100)–(4.105) ensure that the nested partial Chern numbers satisfy SEqs. (4.85)–(4.87), and (4.92) both before and after the deformation. Therefore, we can deduce that the quantities $C^{\pm}_{\gamma_2,in}$, $C^{\pm}_{\gamma_2,out}$, $\frac{1}{2}C^{s}_{\gamma_2,in}$, and $\frac{1}{2}C^{s}_{\gamma_2,out}$ are all $\mathbb{Z}_2$-stable when a spin-resolved Wannier gap closes and reopens. In fact all of $C^{\pm}_{\gamma_2,in}$, $C^{\pm}_{\gamma_2,out}$, $\frac{1}{2}C^{s}_{\gamma_2,in}$, and $\frac{1}{2}C^{s}_{\gamma_2,out}$ are uniquely specified given $(C^{+}_{\gamma_2,in},\nu^{+}_{i})$. In all the following discussion, we refer to $(C^{+}_{\gamma_2,in} \mod 2, \nu^{+}_{i})$ as the *spin-stable invariant* for a 3D inversion- and spinful time-reversal-invariant system with an energy gap, a spin gap, and $\nu^{+}_{j}=\nu^{+}_{l}=0$.

If both the energy gap and the gap between the positive and negative PsP eigenspaces are required to be opened during a deformation of the bulk that preserves $\mathcal{I}$ and $\mathcal{T}$ symmetry, $\nu^{+}_{i}\in\mathbb{Z}$ cannot change. Therefore, we have that

$$(C^{+}_{\gamma_2,in} \mod 2, \nu^{+}_{i}) \in \mathbb{Z}_2\times\mathbb{Z} \tag{4.106}$$

classifies the spin-stable topology of a 3D insulator with $\mathcal{I}$ and spinful $\mathcal{T}$ symmetry and $\nu^{\pm}_{j}=\nu^{\pm}_{l}=0$ (recalling that we have taken $\nu^{\pm}_{j}$ and $\nu^{\pm}_{l}$ to be zero in order to guarantee the possibility of spectrally separated $\widehat{\mathbf{G}}_i$-directed $P_{\pm}$-Wannier bands). In SN 4 D we will compare this $\mathbb{Z}_2\times\mathbb{Z}$ spin-stable topology with symmetry-indicated (energy) band topology. We will then show that a symmetry-indicated helical HOTI can be further resolved into (at least) two families of spin-stable (spin-gapped) phases with different spin-response effects, which includes the experimentally observable spin-magnetoelectric response and the topological contribution to the bulk spin Hall conductivity. After we introduce the notion of *spin-resolved elementary layer construction* in SN 4 D, we will also discuss how to systematically enumerate and analyze various families of 3D spin-stable (spin-gapped) phases with inversion and spinful time-reversal symmetries by stacking 2D insulators in 3D. The stacking approach has the merit of making the spin-response effects physically manifest.

D. Comparison Between Spin-Stable Topology and Symmetry-Indicated Topology in $\mathcal{I}$- and Spinful $\mathcal{T}$-Symmetric 3D Insulators

In this section, we will show that the spin-stable topology (SN 4 C 4) characterized by the spin-resolved Wannier band configurations $(C^{+}_{\gamma_2,in},\nu^{+}_{i})$ [SEq. (4.106)] provides a finer topological classification than the usual stable (symmetry-indicated) (energy) band topology [17, 21, 31, 33, 143]. We will introduce a formal notion of spin-resolved layer constructions and the corresponding symmetry indicators (SIs) to enumerate and analyze insulators in the nonmagnetic space group $P\bar{1}1'$ (# 2.5) [generated by 3D translations, inversion ($\mathcal{I}$), and spinful time-reversal ($\mathcal{T}$)] with a spin gap, which can include the $\mathcal{I}$- and $\mathcal{T}$-protected helical higher-order topological insulators (HOTIs) studied in this work. We will then discuss the different spin-electromagnetic responses of two families of spin-stable (spin-gapped) insulators that are both symmetry-indicated helical HOTIs in the nonmagnetic space group $P\bar{1}1'$ (# 2.5).

In this section, we consider 3D insulators in the nonmagnetic space group $P\bar{1}1'$ (# 2.5) with a spin gap. Provided that the spin gap is open, we can spin-resolve the occupied electronic bands into positive (+) and negative (−) spin bands, as the system has both $\mathcal{I}$ and spinful $\mathcal{T}$ symmetries [see also SN 2 B and SFig. 1(c)]. We denote the 3D partial weak Chern numbers as $\{\nu^{\pm}_{1},\nu^{\pm}_{2},\nu^{\pm}_{3}\}$ [SEq. (4.63)]. Following the discussion in SN 4 C 3 and 4 C 4, we assume that in the presence of $\mathcal{I}$ symmetry, without loss of generality, the $\widehat{\mathbf{G}}_3$-directed $P_{\pm}$-Wannier bands can be separated into inner and outer groups centered around the partial Berry phase values $\gamma^{\pm}_{1}=0$ and $\gamma^{\pm}_{1}=\pi$ (or equivalently $-\pi$) respectively, which by construction implies that the partial weak Chern numbers

$$\nu^{\pm}_{1}=\nu^{\pm}_{2}=0. \tag{4.107}$$

Recall from SN 4 C 3 that we can always choose a supercell such that SEq. (4.107) holds. The nested partial Chern numbers, namely the partial Chern numbers of the inner and outer groups of $\widehat{\mathbf{G}}_3$-directed $P_{\pm}$-Wannier bands, satisfy $C^{\pm}_{\gamma_2,in}+C^{\pm}_{\gamma_2,out}=\nu^{\pm}_{3}$ from SEq. (4.65). Spinful $\mathcal{T}$ symmetry implies from SEqs. (4.67), (4.86), and (4.87) that $C^{+}_{\gamma_2,in}=-C^{-}_{\gamma_2,in}$, $C^{+}_{\gamma_2,out}=-C^{-}_{\gamma_2,out}$, and $\nu^{+}_{3}=-\nu^{-}_{3}$. We then have that the pair $(C^{+}_{\gamma_2,in},\nu^{+}_{3})$ determines the remaining nested partial Chern numbers as

$$C^{\pm}_{\gamma_2,in}=\pm C^{+}_{\gamma_2,in}, \tag{4.108}$$

$$C^{\pm}_{\gamma_2,out}=\pm\left(-C^{+}_{\gamma_2,in}+\nu^{+}_{3}\right). \tag{4.109}$$

We next consider deforming the Hamiltonian in a manner that respects the symmetries of the nonmagnetic space group $P\bar{1}1'$ (# 2.5) and does not close either the energy gap or the spin gap. Under such a deformation, two spin-resolved Wannier band configurations can be deformed into each other provided that

$$(C^{+}_{\gamma_2,in},\nu^{+}_{3})_{\text{configuration }1}-(C^{+}_{\gamma_2,in},\nu^{+}_{3})_{\text{configuration }2}=(2n,0), \tag{4.110}$$

where $n \in \mathbb{Z}$. The deformation proceeds via pairs of gap closings and reopenings related by $\mathcal{I}$ between the inner and outer groups of the $\widehat{\mathbf{G}}_3$-directed $P_\pm$-Wannier bands, as described in SFig. 17. For example, the trivial spin-resolved Wannier band configuration $(C^+_{\gamma_2,in}, \nu^+_3) = (0,0)$ can be deformed into $(C^+_{\gamma_2,in}, \nu^+_3) = (2,0)$ as depicted in SFig. 17, where the $P_\pm$-Wannier bands transfer ± 2 partial Chern number from the outer to inner $P_\pm$-Wannier bands. Hence $(C^+_{\gamma_2,in}, \nu^+_3) = (2,0)$ is equivalent to the trivial $(C^+_{\gamma_2,in}, \nu^+_3) = (0,0)$ in the $\mathbb{Z}_2 \times \mathbb{Z}$ classification of the spin-stable topological phases with vanishing partial weak Chern numbers $\nu^\pm_1 = \nu^\pm_2 = 0$, as per SEq. (4.106). On the other hand, the trivial spin-resolved Wannier band configuration $(C^+_{\gamma_2,in}, \nu^+_3) = (0,0)$ cannot be deformed into $(C^+_{\gamma_2,in}, \nu^+_3) = (1,1)$ or $(0,1)$ since the difference between their $\mathbb{Z}_2 \times \mathbb{Z}$ spin-stable invariants violates SEq. (4.110). In fact, the spin-resolved Wannier band configuration with $(C^+_{\gamma_2,in}, \nu^+_3) = (1,1)$ corresponds to a 3D weak topological insulator (WTI) [141, 142]. This is shown schematically in SFig. 20(a). Similarly, the configuration $(C^+_{\gamma_2,in}, \nu^+_3) = (0,1)$ corresponds to a 3D "obstructed" weak topological insulator (oWTI), which is related to WTI by a half-lattice translation [92]. This is shown schematically in SFig. 20(b). Both $(C^+_{\gamma_2,in}, \nu^+_3) = (1,1)$ and $(0,1)$ are of particular importance in this work, as they serve as the building blocks of spin-stable topological crystalline phases in the nonmagnetic space group $P\bar{1}1'$ (# 2.5) with a spin gap and partial weak Chern numbers $\nu^\pm_1 = \nu^\pm_2 = 0$. We will shortly discuss these two cases in greater detail.

In Supplementary Table 2, we summarize values of nested partial Chern numbers $C^\pm_{\gamma_2,in}$ [SEq. (4.108)] and $C^\pm_{\gamma_2,out}$ [SEq. (4.109)] consistent (up to the addition of a trivial spin-resolved Wannier band configuration) with the $\mathbb{Z}_2 \times \mathbb{Z}$ spin-stable invariants $(C^+_{\gamma_2,in} \mod 2, \nu^+_3)$ in SEq. (4.106). A spin-resolved Wannier band configuration has trivial invariant $(C^+_{\gamma_2,in} \mod 2, \nu^+_3) = (0,0)$ if the inner and outer groups of spin-resolved hybrid Wannier functions have nested partial Chern numbers $C^\pm_{\gamma_2,in} = \pm 2n$ and $C^\pm_{\gamma_2,out} = \mp 2n$ where $n \in \mathbb{Z}$, respectively [as shown in SEq. (4.110)]. From Supplementary Table 2, a spin-resolved Wannier band configuration has $(C^+_{\gamma_2,in} \mod 2, \nu^+_3) = (1,1)$ if the inner and outer groups of the spin-resolved hybrid Wannier functions have nested partial Chern numbers $C^\pm_{\gamma_2,in} = \pm 1$ and $C^\pm_{\gamma_2,out} = 0$, respectively, modulo the addition of a trivial spin-resolved Wannier band configuration. On the other hand, up to the addition of a trivial configuration $(C^+_{\gamma_2,in} \mod 2, \nu^+_3) = (0,0)$, a spin-resolved Wannier band configuration has $(C^+_{\gamma_2,in} \mod 2, \nu^+_3) = (0,1)$ if the inner and outer groups of the spin-resolved hybrid Wannier functions have nested partial Chern numbers $C^\pm_{\gamma_2,in} = 0$ and $C^\pm_{\gamma_2,out} = \pm 1$, respectively. Importantly, $(C^+_{\gamma_2,in} \mod 2, \nu^+_3) = (1,1)$ and $(0,1)$ can be taken as the generators of the $\mathbb{Z}_2 \times \mathbb{Z}$ spin-stable invariants in nonmagnetic space group $P\bar{1}1'$ (# 2.5) with a spin gap and partial weak Chern numbers $\nu^\pm_1 = \nu^\pm_2 = 0$. Any spin-resolved Wannier band configurations can be derived from linear combinations of these two generators where a "negative" generator is obtained by reversing the signs of $(C^+_{\gamma_2,in}, \nu^+_3)$, which according to SEqs. (4.108) and (4.109) amounts to reversing the signs of the corresponding (nested) partial Chern numbers. Several examples of spin-stable (spin-gapped) phases in the nonmagnetic space group $P\bar{1}1'$ (# 2.5) with $\nu^\pm_1 = \nu^\pm_2 = 0$ built using the configurations $(C^+_{\gamma_2,in}, \nu^+_3) = (1,1)$ and $(0,1)$ are shown and discussed in Supplementary Table 2.

1. *Spin-Resolved Layer Constructions*

The enumeration of spin-stable phases based on their spin-resolved Wannier band configurations characterized by $(C^+_{\gamma_2,in}, \nu^+_3)$, or equivalently $C^\pm_{\gamma_2,in}$ and $C^\pm_{\gamma_2,out}$ (Supplementary Table 2), allows us to formulate position-space *layer constructions* of spin-stable (spin-gapped) topological crystalline phases with $\nu^\pm_1 = \nu^\pm_2 = 0$ in the nonmagnetic space group $P\bar{1}1'$ (# 2.5) generated by 3D lattice translations, $\mathcal{I}$, and spinful $\mathcal{T}$. Layer constructions were introduced in SRefs. [33, 42–44, 209] to build and classify magnetic and nonmagnetic topological phases protected by crystal symmetries. Given a 3D (magnetic) space group, the layer construction approach builds a topological crystalline phase by tessellating space with flat layers of 2D topological crystalline phases placed in high-symmetry planes within the unit cell in a manner consistent with the crystal symmetries, and coupled such that the bulk remains gapped. Two layer constructions correspond to the same topological phase if they can be deformed into each other by sliding layers in a manner consistent with the crystal symmetries. As a representative example, let us consider the 3D magnetic space group $P\bar{1}$ (# 2.4), generated by $\mathcal{I}$ and 3D lattice translations with Bravais lattice vectors $\{\mathbf{a}_1, \mathbf{a}_2, \mathbf{a}_3\}$. There are six $\mathcal{I}$-invariant planes in the unit cell perpendicular to one of the primitive reciprocal lattice vectors $\mathbf{G}_i$: they are the constant $r_i = 0, 1/2$ planes (where $\mathbf{r} = r_1\mathbf{a}_1 + r_2\mathbf{a}_2 + r_3\mathbf{a}_3$). It was argued in SRefs. [43, 44] that all gapped, noninteracting topological crystalline phases in magnetic space group $P\bar{1}$ (# 2.4) can be generated from four elementary configurations of layers, termed *elementary layer constructions* (eLCs). In SFig. 18(a,b) we show two of the elementary layer constructions for topological crystalline phases in magnetic space group $P\bar{1}$ (# 2.4) with weak Chern numbers $\nu_1 = \nu_2 = 0$, where we tile 3D space by $C_{\gamma_1} = 1$ inversion-symmetric Chern insulators in the $r_3 = 0$ or $r_3 = 1/2$ planes respectively. These two elementary layer constructions correspond to $\nu_3 = 1$ 3D quantum

	$(C^{+}_{\gamma_2,in} \mod 2, \nu_3^{+}) \in \mathbb{Z}_2 \times \mathbb{Z}$ Spin-Stable Invariants					
	$(0,0)$	$(1,1)$	$(0,1)$	$(0,2)$	$(1,2)$	$(1,0)$
$C^{\pm}_{\gamma_2,in}$	$\pm 2n$	± 1	0	0	± 1	± 1
$C^{\pm}_{\gamma_2,out}$	$\mp 2n$	0	± 1	± 2	± 1	∓ 1

Supplementary Table 2. Values of the nested partial Chern numbers (spin-resolved Wannier band configurations) $C^{\pm}_{\gamma_2,in}$ and $C^{\pm}_{\gamma_2,out}$ consistent with the given $\mathbb{Z}_2 \times \mathbb{Z}$ spin-stable invariants $(C^{+}_{\gamma_2,in} \mod 2, \nu_3^{+})$, assuming that the partial weak Chern numbers $\nu_1^{\pm} = \nu_2^{\pm} = 0$ as in SEq. (4.107) (which is required to admit the possibility of a gap in the $\hat{\mathbf{G}}_3$-directed $P_{\pm}$-Wilson loop spectrum). The first column indicates that the trivial configuration $(0,0)$ is consistent with even-integer nested partial Chern numbers $C^{\pm}_{\gamma_2,in} = \pm 2n$ for the inner $P_{\pm}$-Wanner bands, and opposite even-integer nested partial Chern numbers $C^{\pm}_{\gamma_2,out} = \mp 2n$ for the outer $P_{\pm}$-Wannier bands. The configurations $(1,1)$ and $(0,1)$ can be taken as the generators of the $\mathbb{Z}_2 \times \mathbb{Z}$ group of spin-stable (spin-gapped) phases in the nonmagnetic space group $P\bar{1}1'$ (# 2.5) with $\nu_1^{\pm} = \nu_2^{\pm} = 0$, where we define the negative of the generators by reversing the signs of the nested partial Chern numbers in the table. In particular, $(1,1)$ corresponds to the $\nu_3^{\pm} = \pm 1$ weak topological insulator [141, 142] depicted in SFig. 20(a), and $(0,1)$ corresponds to the $\nu_3^{\pm} = \pm 1$ obstructed weak topological insulator [92] depicted in SFig. 20(b). After the double-vertical line in the table, we give three examples of spin-stable phases with spin-resolved Wannier band configurations derived from linear combinations of the generators. To add two configurations we add their inner and outer nested partial Chern numbers, or equivalently add their $(C^{+}_{\gamma_2,in}, \nu_3^{+})$. The $(0,2)$ phase can be realized with the configuration $C^{\pm}_{\gamma_2,in} = 0$ and $C^{\pm}_{\gamma_2,out} = \pm 2$, corresponding to the linear combination $(0,2) = (0,1) \oplus (0,1)$. The $(1,2)$ spin-stable phase can be realized with the configuration $C^{\pm}_{\gamma_2,in} = \pm 1$ and $C^{\pm}_{\gamma_2,out} = \pm 1$, corresponding to the linear combination $(1,2) = (0,1) \oplus (1,1)$. This corresponds to the $\nu_3^{\pm} = \pm 2$ quantum spin Hall insulator (QSHI), depicted in SFig. 22(a). The $(1,0)$ spin-stable phase can be realized with the configuration $C^{+}_{\gamma_2,in} = \pm 1$ and $C^{+}_{\gamma_2,out} = \mp 1$, corresponding to the linear combination $(1,0) = (1,1) \ominus (0,1)$. This phase corresponds to the $\mathcal{T}$-doubled axion insulator (T-DAXI) depicted in SFig. 22(b). We note that we can add a configuration in the trivial phase $(0,0)$ for any integer n to any of these representative configurations without changing the spin-stable phase. This corresponds to the fact that, as shown in SEq. (4.106) and the surrounding text, $C^{+}_{\gamma_2,in}$ is only stable modulo 2.

anomalous Hall insulator (QAHI) and $\nu_3 = 1$ 3D obstructed quantum anomalous Hall insulator (oQAHI), where the $\nu_3 = 1$ oQAHI in SFig. 18(b) is related to the $\nu_3 = 1$ QAHI in SFig. 18(a) by a half-lattice translation along $\mathbf{a}_3$ [92]. For completeness, we note that the two remaining elementary layer constructions are obtained by placing an inversion-symmetric Chern insulator with Chern number $C_{\gamma_1} = 1$ in either the $r_1 = 1/2$ or $r_2 = 1/2$ plane. They have, respectively, $(\nu_1, \nu_2, \nu_3) = (1,0,0)$ and $(\nu_1, \nu_2, \nu_3) = (0,1,0)$. There are four eLCs rather than 3 to account for the fact that the "Chern number polarization" relative to the origin is a strong $\mathbb{Z}_2$ invariant protected by inversion symmetry [16, 33, 43, 46, 92].

Let us now consider the topological crystalline phases built from the two $(\nu_1, \nu_2, \nu_3) = (0,0,1)$ elementary layer constructions in magnetic space group $P\bar{1}$ (# 2.4) discussed above. Focusing on topological configurations with weak Chern numbers $\nu_1 = \nu_2 = 0$, in SFig. 19 we show the layer construction of two representative phases. In SFig. 19(a) we show how a 3D QAHI with weak Chern number $\nu_3 = 2$ in magnetic space group $P\bar{1}$ (# 2.4) can be constructed by tiling space with a $C_{\gamma_1} = 1$ Chern insulator placed in the $r_3 = 0$ plane in each unit cell and a $C_{\gamma_1} = 1$ Chern insulator placed in the $r_3 = 1/2$ plane in each unit cell. This corresponds to the sum of the two elementary layer constructions depicted in SFig. 18. In SFig. 19(b) we show how an $\mathcal{I}$-protected 3D magnetic axion insulator (AXI) in magnetic space group $P\bar{1}$ (# 2.4), which is characterized by zero weak Chern numbers $\nu_1 = \nu_2 = \nu_3 = 0$ and a quantized bulk axion angle $\theta = \pi$ [77, 79], can be constructed by tiling space with a $C_{\gamma_1} = 1$ inversion-symmetric Chern insulator placed in the $r_3 = 0$ plane in each unit cell and a $C_{\gamma_1} = -1$ inversion-symmetric Chern insulator placed in the $r_3 = 1/2$ plane in each unit cell. This corresponds to the *difference* of the two elementary layer constructions depicted in SFig. 18, where the negative of an elementary layer construction is obtained by reversing the signs of the Chern numbers of the 2D layers that tile 3D space. We emphasize that both adding and subtracting elementary layer constructions increases the number of occupied bands in the system.

The layer construction method allows us to efficiently identify symmetry-protected topological phases that span the SI group of a given (magnetic or nonmagnetic) space group. Recall that the magnetic space group $P\bar{1}$ (# 2.4) has the SIs $(\tilde{z}_4, \tilde{z}_{2,1}, \tilde{z}_{2,2}, \tilde{z}_{2,3})$ which can be computed from the inversion eigenvalues of the occupied energy bands at the high-symmetry points in the BZ [43–46, 143]. The strong $\mathbb{Z}_4$ index $\tilde{z}_4$ is specifically determined by the half of the difference between the number of occupied energy bands of positive (n_+^a) and negative (n_-^a) parity (inversion eigenvalues), modulo 4 at all 8 TRIMs $\mathbf{k}_a$,

$$\tilde{z}_4 \equiv \frac{1}{2} \sum_{\mathbf{k}_a \in \text{TRIMs}} \left(n_+^a - n_-^a \right) \mod 4. \tag{4.111}$$

The three weak $\mathbb{Z}_2$ indices are determined by the half of the difference between the number of occupied energy bands

of positive and negative parity at all 4 TRIMs in the $\mathbf{k}_a \cdot \mathbf{a}_i = \pi$ planes, modulo 2,

$$\tilde{z}_{2,i} \equiv \frac{1}{2} \sum_{\substack{\mathbf{k}_a \in \text{TRIMs} \\ \mathbf{k}_a \cdot \mathbf{a}_i = \pi}} \left(n_+^a - n_-^a \right) \bmod 2, \tag{4.112}$$

where $i = 1, 2, 3$. We see in SFig. 18 that the 3D QAHI with $\nu_3 = 1$ and the 3D oQAHI with $\nu_3 = 1$ have the SIs $(\tilde{z}_4, \tilde{z}_{2,1}, \tilde{z}_{2,2}, \tilde{z}_{2,3}) = (2, 0, 0, 1)$ and $(0, 0, 0, 1)$, respectively. Similarly, we see in SFig. 19 that the 3D QAHI with $\nu_3 = 2$ and the 3D AXI have the same SIs $(\tilde{z}_4, \tilde{z}_{2,1}, \tilde{z}_{2,2}, \tilde{z}_{2,3}) = (2, 0, 0, 0)$.

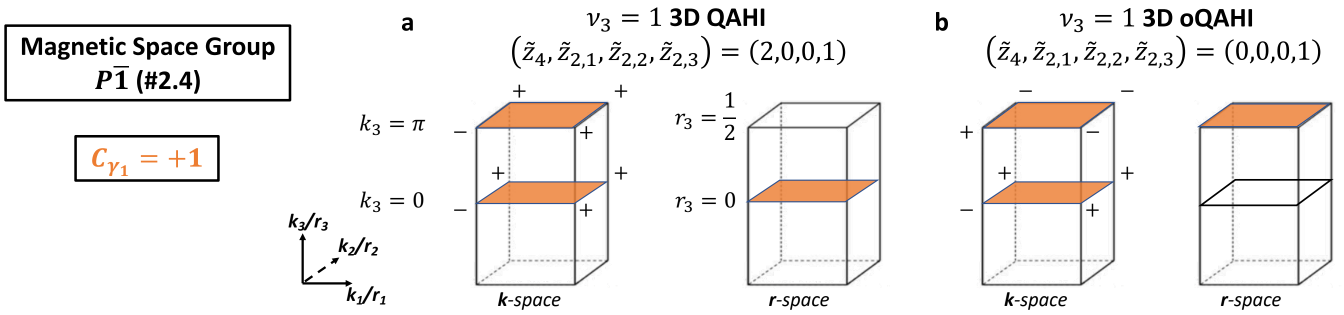

Supplementary Figure 18. Elementary layer constructions for topological crystalline phases in the magnetic space group $P\bar{1}$ (# 2.4) with weak Chern numbers $\nu_1 = \nu_2 = 0$. The coordinates k_i and r_i $(i = 1, 2, 3)$ in momentum($\mathbf{k}$)-space and position($\mathbf{r}$)-space are given by $\mathbf{k} = \sum_{i=1}^{3} \frac{k_i}{2\pi} \mathbf{G}_i$ and $\mathbf{r} = \sum_{i=1}^{3} r_i \mathbf{a}_i$ respectively. $\{\mathbf{a}_1, \mathbf{a}_2, \mathbf{a}_3\}$ are the $\mathbf{r}$-space primitive lattice vectors and $\{\mathbf{G}_1, \mathbf{G}_2, \mathbf{G}_3\}$ are the dual $\mathbf{k}$-space primitive reciprocal lattice vectors such that $\mathbf{a}_i \cdot \mathbf{G}_j = 2\pi\delta_{ij}$ $(i, j = 1, 2, 3)$. The $\mathcal{I}$-invariant constant-r_3 planes contain $r_3 = 0$ and $r_3 = 1/2$, which correspond to the center and the boundary of the primitive unit cell along $\mathbf{a}_3$. Similarly, the $\mathcal{I}$-symmetric constant-k_3 planes contain $k_3 = 0$ and $k_3 = \pi$, which correspond to the center and the boundary of the BZ along $\mathbf{G}_3$. (a) shows the layer construction of a $\nu_3 = 1$ 3D quantum anomalous Hall insulator (QAHI) in both $\mathbf{r}$-space (right) and $\mathbf{k}$-space (left). In $\mathbf{r}$-space, the $\nu_3 = 1$ 3D QAHI can be constructed by tiling 3D space with 2D $\mathcal{I}$-symmetric Chern insulators with Chern number $C_{\gamma_1} = 1$ placed in the $\mathcal{I}$-symmetric $r_3 = 0$ plane within each unit cell, as shown on the right-hand side of (a). The occupied energy bands in momentum space for this layer construction each have Chern number $C_{\gamma_1} = 1$ in both the $\mathcal{I}$-symmetric $k_3 = 0$ and $k_3 = \pi$ planes. (b) shows the layer construction of a $\nu_3 = 1$ 3D obstructed quantum anomalous Hall insulator (oQAHI) in both $\mathbf{r}$-space (right) and $\mathbf{k}$-space (left) [92]. In $\mathbf{r}$-space, the $\nu_3 = 1$ 3D oQAHI can be constructed by tiling 3D space with 2D $\mathcal{I}$-symmetric Chern insulators with Chern number $C_{\gamma_1} = 1$ placed in the $\mathcal{I}$-symmetric $r_3 = 1/2$ plane within each unit cell, as shown on the right-hand side of (b). The occupied energy bands in momentum space for this layer construction have Chern number $C_{\gamma_1} = 1$ in both the $\mathcal{I}$-symmetric $k_3 = 0$ and $k_3 = \pi$ planes. The 3D oQAHI in (b) and 3D QAHI in (a) are related to each other by a half-lattice translation along $\mathbf{a}_3$. The inversion eigenvalues at the eight TRIMs compatible with the $\mathbf{r}$-space layer construction for (a) and (b) with one occupied electronic energy band are also shown individually on the left-hand side of each panel, from which the $\mathbb{Z}_4 \times (\mathbb{Z}_2)^3$ SIs $(\tilde{z}_4, \tilde{z}_{2,1}, \tilde{z}_{2,2}, \tilde{z}_{2,3})$ [SEqs. (4.111) and (4.112)] are determined to be $(2, 0, 0, 1)$ for (a) and $(0, 0, 0, 1)$ for (b). Any topological crystalline phases in the magnetic space group $P\bar{1}$ (# 2.4) with weak Chern numbers $\nu_1 = \nu_2 = 0$ can be derived from linear combinations of (a) and (b), where the negative of a layer construction is obtained by reversing the signs of the Chern numbers of the 2D layers (as opposed to subtracting bands from the occupied subspace) [43, 44].

The layer construction of topological crystalline insulators has a direct connection to the nested (partial) Berry phase classifications that we introduced in SN 4 A and 4 B. This is because the inner nested (partial) Chern number $C_{\gamma_2,in}^{(\pm)}$ gives the (partial) Chern number for the (spin-resolved) hybrid Wannier bands centered at an $\mathcal{I}$-symmetric plane at the origin of the unit cell. Similarly, the outer nested (partial) Chern number $C_{\gamma_2,out}^{(\pm)}$ gives the (partial) Chern number for the (spin-resolved) hybrid Wannier bands centered at an $\mathcal{I}$-symmetric plane at the boundary of the unit cell. In particular, as we have shown in SEq. (4.65) of SN 4 C 3, the summation of $C_{\gamma_2,in}^{(\pm)}$ and $C_{\gamma_2,out}^{(\pm)}$ is the (partial) weak Chern number along the direction normal to the layers (in a layer construction with parallel layers). This allows us to associate each layer construction to a configuration of $C_{\gamma_2,in}^{(\pm)}$ and $C_{\gamma_2,out}^{(\pm)}$, where $C_{\gamma_2,in}^{(\pm)}$ gives the (partial) Chern number of the layer at the origin of the unit cell, and $C_{\gamma_2,out}^{(\pm)}$ gives the (partial) Chern number at the boundary of the unit cell.

We can now adapt these observations to formulate a *spin-resolved* layer construction for the spin-stable (spin-gapped) topological crystalline phases with $\nu_1^{\pm} = \nu_2^{\pm} = 0$ in the nonmagnetic space group $P\bar{1}1'$ (# 2.5). First, although the projector $P(\mathbf{k})$ onto the occupied electronic energy bands is invariant under the nonmagnetic space group $P\bar{1}1'$ (# 2.5), the projectors $P_{\pm}(\mathbf{k})$ onto the positive/negative spin bands are invariant only under the magnetic subgroup $P\bar{1}$ (# 2.4) of symmetries that commute with PsP. From this it follows that the spin-resolved topology

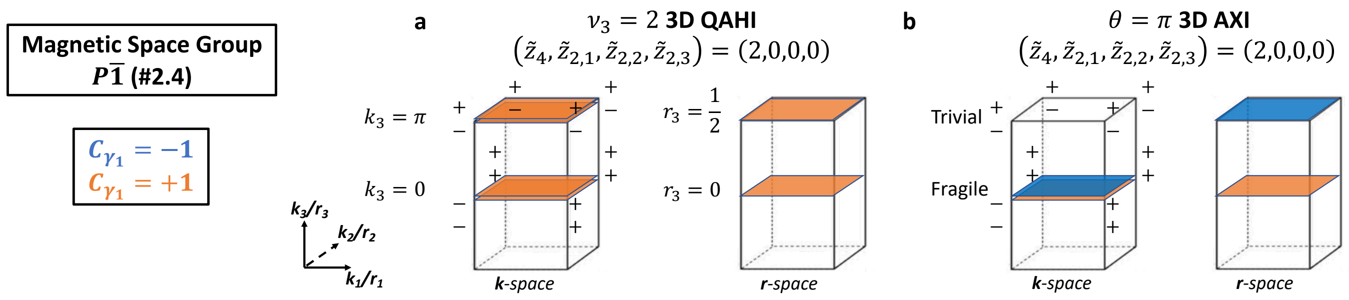

Supplementary Figure 19. Layer constructions for (a) a 3D quantum anomalous Hall insulator (QAHI) with weak Chern numbers $\nu_1 = \nu_2 = 0$ and $\nu_3 = 2$, and (b) an $\mathcal{I}$-protected 3D axion insulator (AXI) characterized by $\nu_1 = \nu_2 = \nu_3 = 0$ and an $\mathcal{I}$-quantized bulk axion angle $\theta = \pi$ [15, 33, 42–44, 209]. Both (a) and (b) are topological phases in the magnetic space group $P\bar{1}$ (# 2.4) with $\nu_1 = \nu_2 = 0$. The coordinates k_i and r_i $(i = 1, 2, 3)$ in momentum($\mathbf{k}$)-space and position($\mathbf{r}$)-space are given by $\mathbf{k} = \sum_{i=1}^{3} \frac{k_i}{2\pi}\mathbf{G}_i$ and $\mathbf{r} = \sum_{i=1}^{3} r_i\mathbf{a}_i$ respectively. $\{\mathbf{a}_1, \mathbf{a}_2, \mathbf{a}_3\}$ are the $\mathbf{r}$-space primitive lattice vectors and $\{\mathbf{G}_1, \mathbf{G}_2, \mathbf{G}_3\}$ are the dual $\mathbf{k}$-space primitive reciprocal lattice vectors such that $\mathbf{a}_i \cdot \mathbf{G}_j = 2\pi\delta_{ij}$ $(i, j = 1, 2, 3)$. The $\mathcal{I}$-invariant constant-r_3 planes contain $r_3 = 0$ and $r_3 = 1/2$, which correspond to the center and the boundary of the primitive unit cell along $\mathbf{a}_3$. Similarly, the $\mathcal{I}$-symmetric constant-k_3 planes contain $k_3 = 0$ and $k_3 = \pi$, which correspond to the center and the boundary of the BZ along $\mathbf{G}_3$. The right-hand side of (a) shows that to layer-construct a 3D QAHI with $\nu_3 = 2$, we place $\mathcal{I}$-symmetric Chern insulators with Chern numbers $C_{\gamma_1} = +1$ in both the $r_3 = 0$ and $r_3 = 1/2$ planes within each unit cell in the $\mathbf{r}$-space. The left-hand side of (a) shows that the occupied energy bands in $\mathbf{k}$-space for this layer construction have Chern number $C_{\gamma_1} = +2$ in both the $k_3 = 0$ and $k_3 = \pi$ planes. The right-hand side of (b) shows that to layer-construct a 3D AXI, we place an $\mathcal{I}$-symmetric Chern insulator with a Chern number $C_{\gamma_1} = +1$ in the $r_3 = 0$ plane of each unit cell, and a $\mathcal{I}$-symmetric Chern insulator with a Chern number $C_{\gamma_1} = -1$ in the $r_3 = 1/2$ plane of each unit cell in $\mathbf{r}$-space. The left-hand side of (b) shows that the two occupied energy bands in $\mathbf{k}$-space for this layer construction individually carry the Chern numbers $C_{\gamma_1} = \pm 1$ in the $k_3 = 0$ plane, such that the total Chern number in the $k_3 = 0$ plane vanishes. Along with the vanishing Chern number $C_{\gamma_1} = 0$ in the $k_3 = \pi$ plane, this is consistent with the fragile-phase (instead of strong 2D-phase) pumping picture of an AXI introduced in SRef. [15]. The inversion eigenvalues at the eight TRIMs compatible with the layer construction for (a) and (b) with two occupied electronic energy bands are also shown on left-hand side of each panel. The $\mathbb{Z}_4 \times (\mathbb{Z}_2)^3$ SIs $(\tilde{z}_4, \tilde{z}_{2,1}, \tilde{z}_{2,2}, \tilde{z}_{2,3})$ [SEqs. (4.111) and (4.112)] are $(2, 0, 0, 0)$ for both (a) and (b). In particular, on the left-hand side of (b), the two occupied energy bands in the $k_3 = 0$ plane for a 3D AXI have 2D fragile topology in the magnetic space group $P\bar{1}$ (# 2.4), though the 3D (strong axionic) topology is stable [15].

of the projectors $P_\pm(\mathbf{k})$ correspond to the topology of bands in the magnetic subgroup $P\bar{1}$ (# 2.4) of $P\bar{1}1'$ (# 2.5). (We note that in systems with conserved spin, spin-resolved band topology can also be analyzed using spin space groups [43, 211–219]).

Spinful $\mathcal{T}$ symmetry only constrains that the states in the image of $P_+(\mathbf{k})$ are related to the states in the image of $P_-(\mathbf{k})$ by spinful $\mathcal{T}$, such that states in the image of $P(\mathbf{k}) = P_+(\mathbf{k}) + P_-(\mathbf{k})$ are symmetric under the symmetries of $P\bar{1}1'$ (# 2.5). Recall that the four elementary layer constructions for the magnetic subgroup $P\bar{1}$ (# 2.4) correspond to tiling 3D space with $\mathcal{I}$-symmetric Chern insulators with Chern number $C_{\gamma_1} = 1$ in $\mathcal{I}$-invariant planes within the unit cell. We then deduce that the four elementary spin-resolved layer constructions for spin-stable phases in nonmagnetic group $P\bar{1}1'$ (# 2.5) correspond to tiling 3D space with 2D spin-gapped, translation-, $\mathcal{I}$-, and spinful $\mathcal{T}$-invariant insulators with partial Chern numbers $C^{\pm}_{\gamma_1} = \pm 1$ in $\mathcal{I}$-invariant planes within the unit cell. Restricting to the cases with $\nu_1^\pm = \nu_2^\pm = 0$, this yields two elementary spin-resolved layer constructions L_1 and L_2, as shown in Supplementary Table 3; two other elementary spin-resolved layer constructions can be obtained by permuting the crystalline axes in L_2 such that $\nu_1^\pm = \nu_3^\pm = 0$ or $\nu_2^\pm = \nu_3^\pm = 0$. L_1 [L_2] are constructed by tiling 3D space with 2D $\mathcal{I}$- and $\mathcal{T}$-symmetric spin-gapped insulators with partial Chern numbers $C^{\pm}_{\gamma_1} = \pm 1$ in the $\mathcal{I}$-invariant plane $r_3 = 0$ [$r_3 = 1/2$] within each unit cell, as shown in SFig. 20(a) [SFig. 20(b)]. Recall from SN 3 C that a $\mathcal{T}$-symmetric spin-gapped 2D system with odd partial Chern numbers is a 2D strong topological insulator. By construction, L_1 is then a WTI [141, 142] while L_2 is an oWTI [92] related to L_1 by a half-lattice translation along $\mathbf{a}_3$. L_1 and L_2 are *elementary* in the sense that any spin-resolved layer constructions with spin-resolved Wannier band configurations described by nested partial Chern numbers in SEqs. (4.108) and (4.109) can be derived from linear combinations of L_1 and L_2, where the negative of a spin-resolved layer construction can be obtained by reversing the signs of all the partial Chern numbers $C^{\pm}_{\gamma_1}$ of the 2D layers. In particular, as indicated in Supplementary Table 3, we have that L_1 and L_2 have spin-stable invariants $(C^{+}_{\gamma_2,in} \mod 2, \nu_3^+) = (1, 1)$ and $(0, 1)$ respectively, which are the generators of the $\mathbb{Z}_2 \times \mathbb{Z}$ group of spin-stable topology of nonmagnetic space group $P\bar{1}1'$ (# 2.5) with $\nu_1^\pm = \nu_2^\pm = 0$.

From the two elementary spin-resolved layer constructions L_1 and L_2 in Supplementary Table 3 and SFig. 20, we

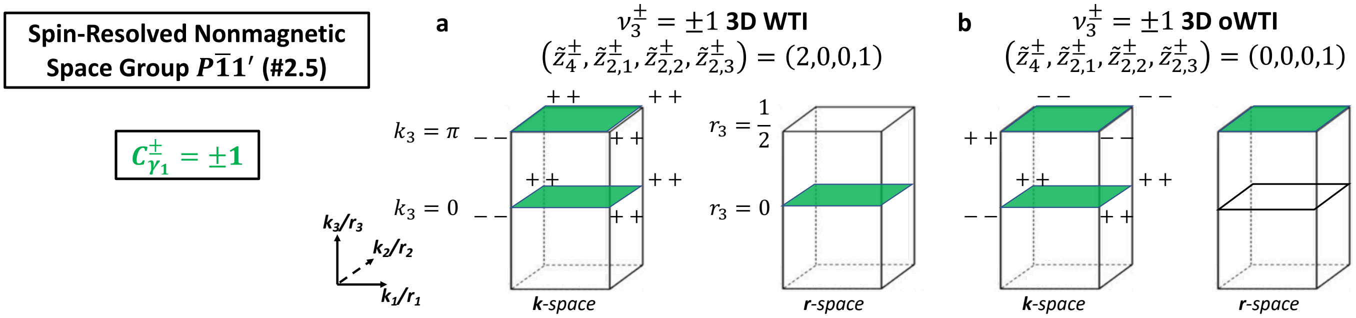

Supplementary Figure 20. Elementary spin-resolved layer constructions for topological crystalline phases in the nonmagnetic space group $P\bar{1}1'$ (# 2.5) with a spin gap and partial weak Chern numbers $\nu_1^{\pm} = \nu_2^{\pm} = 0$. The coordinates k_i and r_i $(i = 1, 2, 3)$ in momentum($\mathbf{k}$)-space and position($\mathbf{r}$)-space are given by $\mathbf{k} = \sum_{i=1}^{3} \frac{k_i}{2\pi}\mathbf{G}_i$ and $\mathbf{r} = \sum_{i=1}^{3} r_i \mathbf{a}_i$ respectively. $\{\mathbf{a}_1, \mathbf{a}_2, \mathbf{a}_3\}$ are the $\mathbf{r}$-space primitive lattice vectors and $\{\mathbf{G}_1, \mathbf{G}_2, \mathbf{G}_3\}$ are the dual $\mathbf{k}$-space primitive reciprocal lattice vectors such that $\mathbf{a}_i \cdot \mathbf{G}_j = 2\pi\delta_{ij}$ $(i, j = 1, 2, 3)$. The $\mathcal{I}$-invariant constant-r_3 planes contain $r_3 = 0$ and $r_3 = 1/2$, which correspond to the center and the boundary of the primitive unit cell along $\mathbf{a}_3$. Similarly, the $\mathcal{I}$-symmetric constant-k_3 planes contain $k_3 = 0$ and $k_3 = \pi$, which correspond to the center and the boundary of the BZ along $\mathbf{G}_3$. (a) shows the layer construction of a $\nu_3^{\pm} = \pm 1$ 3D weak topological insulator (WTI) in both $\mathbf{r}$-space (right) and $\mathbf{k}$-space (left). In $\mathbf{r}$-space, the $\nu_3^{\pm} = \pm 1$ 3D WTI can be constructed by tiling 3D space by 2D translation-, $\mathcal{I}$-, and spinful $\mathcal{T}$-symmetric layers with partial Chern numbers $C^{\pm}_{\gamma_1} = \pm 1$ in the $\mathcal{I}$-symmetric $r_3 = 0$ plane within each unit cell, as shown on the right-hand side of (a). As we showed in SN 3 C, a 2D spinful $\mathcal{T}$-invariant system with partial Chern numbers $C^{\pm}_{\gamma_1} = \pm 1$ is a 2D strong topological insulator, hence by construction (a) is a WTI [141, 142]. The occupied energy bands for the layer construction of this 3D WTI have partial Chern numbers $C^{\pm}_{\gamma_1} = \pm 1$ in both the $\mathcal{I}$-symmetric $k_3 = 0$ and $k_3 = \pi$ planes, as shown in the left of panel (a). (b) shows the layer construction of a $\nu_3^{\pm} = \pm 1$ 3D obstructed weak topological insulator (oWTI) in both $\mathbf{r}$-space (right) and $\mathbf{k}$-space (left) [92]. In $\mathbf{r}$-space, the $\nu_3^{\pm} = \pm 1$ 3D oWTI can be constructed by tiling 3D space by 2D layers with partial Chern numbers $C^{\pm}_{\gamma_1} = \pm 1$ in the $\mathcal{I}$-symmetric $r_3 = 1/2$ plane within each unit cell, as shown on the right-hand side of (b). The occupied energy bands for the layer construction of 3D oWTI have partial Chern numbers $C^{\pm}_{\gamma_1} = \pm 1$ in both the $\mathcal{I}$-symmetric $k_3 = 0$ and $k_3 = \pi$ planes, as shown in the left of panel (b). The 3D oWTI in (b) and 3D WTI in (a) are related to each other by a half-lattice translation along $\mathbf{a}_3$ [92]. The inversion eigenvalues at the eight TRIMs compatible with the $\mathbf{r}$-space layer construction for (a) and (b) with two occupied electronic energy band are also shown on the left-hand side of each panel. The $\mathbb{Z}_4 \times (\mathbb{Z}_2)^3$ partial SIs (defined in SN 4 D 2) $(\tilde{z}_4^{\pm}, \tilde{z}_{2,1}^{\pm}, \tilde{z}_{2,2}^{\pm}, \tilde{z}_{2,3}^{\pm})$ are $(2, 0, 0, 1)$ for (a) and $(0, 0, 0, 1)$ for (b). Any topological crystalline phases in the nonmagnetic space group $P\bar{1}1'$ (# 2.5) with a spin gap and partial weak Chern numbers $\nu_1^{\pm} = \nu_2^{\pm} = 0$ can be derived from linear combinations of (a) and (b), where the negative of a layer construction is obtained by reversing the signs of the partial Chern numbers of 2D layers tiling 3D space in the layer construction [33, 210]. In particular, (a) and (b) correspond to the L_1 and L_2 elementary spin-resolved layer constructions in Supplementary Table 3, respectively.

can build the trivial $\mathbb{Z}_2 \times \mathbb{Z}$ spin-stable phases with $(C^{+}_{\gamma_2,in} \mod 2, \nu_3^{+}) = (0, 0)$ by forming the linear combinations $2nL_1 \ominus 2nL_2$, since

$$2n(1 \mod 2, 1) - 2n(0 \mod 2, 1) = (0, 0). \tag{4.113}$$

Therefore, any spin-resolved layer construction with trivial $\mathbb{Z}_2 \times \mathbb{Z}$ invariants $(C^{+}_{\gamma_2,in} \mod 2, \nu_3^{+}) = (0, 0)$ corresponds to having 2D layers with partial Chern numbers $C^{\pm}_{\gamma_1} = \pm 2n$ and $C^{\pm}_{\gamma_1} = \mp 2n$ in the $r_3 = 0$ and $r_3 = 1/2$ planes, respectively, consistent with Supplementary Table 2. We can always add this trivial layer construction to any spin-stable phase without changing the spin-stable topology. We can make contact with SN 4 C 4 by noting that the trivial spin-resolved layer constructions $2nL_1 \ominus 2nL_2$ can also be obtained by tiling 3D space by 2D layers with *in total* zero partial Chern numbers at generic constant-r_3 planes related by $\mathcal{I}$. These layers can be moved to inversion-symmetric constant-r_3 planes in a manner respecting the nonmagnetic space group symmetry $P\bar{1}1'$ (# 2.5); such a *spin-resolved bubbling process* [46, 210] is demonstrated in SFig. 21. Two spin-resolved layer constructions in the nonmagnetic space group $P\bar{1}1'$ (# 2.5) describe the same spin-stable phases if they can be related by such a spin-resolved bubbling process. We term such an equivalence as the *spin-resolved bubble equivalence* [210]. The spin-resolved bubbling process requires a gap closing and reopening in the spin-resolved Wannier spectrum, consistent with the change in nested partial Chern numbers [SEqs. (4.100) and (4.101)].

	L_1	L_2	built from elementary spin-resolved layer constructions				
$(C^+_{\gamma_2,in}, \nu_3^+)$	$(1,1)$	$(0,1)$	$(0,0)$	$(0,2)$	$(0,2)$	$(1,2)$	$(1,0)$
$C^+_{\gamma_1}$ in $r_3 = 0$	1	0	$2n$	0	2	1	1
$C^+_{\gamma_1}$ in $r_3 = 1/2$	0	1	$-2n$	2	0	1	-1

Supplementary Table 3. Spin-resolved layer constructions for the spin-stable phases in the nonmagnetic space group $P\bar{1}1'$ (# 2.5) with a spin gap and partial weak Chern numbers $\nu_1^\pm = \nu_2^\pm = 0$ in Supplementary Table 2. Since $C^-_{\gamma_1} = -C^+_{\gamma_1}$ by time-reversal symmetry, we only list the values of partial Chern number $C^+_{\gamma_1}$ for the 2D layers. All the spin-stable phases in the nonmagnetic space group $P\bar{1}1'$ (# 2.5) with a spin gap and $\nu_1^\pm = \nu_2^\pm = 0$ in Supplementary Table 2 can be derived from linear combinations of the elementary spin-resolved layer constructions L_1 and L_2, where we define the negative of a spin-resolved layer construction by reversing the signs of the partial Chern numbers of all the 2D layers. The trivial spin-stable phase is realized by any linear combination of the form $2nL_1 \ominus 2nL_2$ where $n \in \mathbb{Z}$. The spin-resolved layer construction with $\mathbb{Z}_2 \times \mathbb{Z}$ invariant $(C^+_{\gamma_2,in} \mod 2, \nu_3^+) = (0,2)$ can be obtained both from $2L_1$ or $2L_2 = 2L_1 \oplus (2L_2 \ominus 2L_1)$. This is because $2L_1$ corresponds to $(C^+_{\gamma_2,in} \mod 2, \nu_3^+) = 2 \times (1 \mod 2, 1) = (0,2)$, and $2L_2$ corresponds to $(C^+_{\gamma_2,in} \mod 2, \nu_3^+) = 2 \times (0 \mod 2, 1) = (0,2)$. $2L_1$ and $2L_2$ can be constructed by placing a 2D layer with partial Chern numbers $C^\pm_{\gamma_1} = \pm 2$ in the $r_3 = 0$ or $r_3 = 1/2$ plane, respectively. Such a 2D layer with $C^\pm_{\gamma_1} = \pm 2$ and two occupied energy bands can be realized, for example, using the model of a fragile topological insulator introduced in SRef. [8] (and analyzed in detail in SN 3 G) in the s_z-conserved limit [$v_{Mz} = 0$ in SEq. (3.55)], or also in the presence of s_z-non-conserving SOC provided that the spin gap is open [$v_{Mz} \neq 0$ in SEq. (3.55)]. The 3D quantum spin Hall insulator with $\nu_3^\pm = \pm 2$ in SFig. 22(a), which has the spin-stable invariants $(C^+_{\gamma_2,in} \mod 2, \nu_3^+) = (1,2)$, can be constructed as $L_1 \oplus L_2$. The 3D $\mathcal{T}$-doubled axion insulator with $\nu_3^\pm = 0$ and quantized bulk partial axion angles $\theta^\pm = \pi$ [SEq. (4.123)] in SFig. 22(b), which has the spin-stable invariants $(C^+_{\gamma_2,in} \mod 2, \nu_3^+) = (1,0)$, can be constructed as $L_1 \ominus L_2$.

2. *The Quantum Spin Hall Insulator and the $\mathcal{T}$-Doubled Axion Insulator*

Using the two elementary spin-resolved layer constructions L_1 and L_2 in Supplementary Table 3, we will now build two representative families of spin-stable (spin-gapped) topological crystalline phases in nonmagnetic space group $P\bar{1}1'$ (# 2.5) with $\nu_1^\pm = \nu_2^\pm = 0$. In SFig. 22(a), we show that a 3D quantum spin Hall insulator (QSHI) with the partial weak Chern numbers $\nu_3^\pm = \pm 2$ can be constructed by tiling 3D space with $C^\pm_{\gamma_1} = \pm 1$ 2D layers in the $\mathcal{I}$-symmetric $r_3 = 0$ plane within each unit cell, and $C^\pm_{\gamma_1} = \pm 1$ 2D layers in the $\mathcal{I}$-symmetric $r_3 = 1/2$ plane within each unit cell. SFig. 22(a) corresponds to the sum $L_1 \oplus L_2$ of the two elementary spin-resolved layer constructions defined in Supplementary Table 3 and SFig. 20. In SFig. 22(b) we show that a 3D phase in nonmagnetic space group $P\bar{1}1'$ (# 2.5) that we term in this work the $\mathcal{T}$-doubled magnetic axion insulator (T-DAXI) can be constructed by tiling 3D space with $C^\pm_{\gamma_1} = \pm 1$ 2D layers in the $r_3 = 0$ plane within each unit cell, and $C^\pm_{\gamma_1} = \mp 1$ 2D layers in the $r_3 = 1/2$ plane within each unit cell. This corresponds to the difference $L_1 \ominus L_2$ of the two elementary spin-resolved layer constructions (Supplementary Table 3 and SFig. 20). Recall that the elementary spin-resolved layer constructions in the nonmagnetic space group $P\bar{1}1'$ (# 2.5) are obtained from time-reversed pairs of the elementary layer construction in the magnetic subgroup $P\bar{1}$ (# 2.4). The QSHI with $\nu_3^\pm = \pm 2$ in SFig. 22(a) can be spin-resolved into QAHI states [SFig. 19(a)] in both the positive and negative PsP eigenspaces that are related to each other by spinful $\mathcal{T}$. On the other hand, the T-DAXI in SFig. 22(b) can be spin-resolved into AXI states [SFig. 19(b)] in both the positive and negative PsP eigenspaces that are related to each other by spinful $\mathcal{T}$. This justifies our choice to term this spin-stable phase as the $\mathcal{T}$-*doubled* AXI. The T-DAXI is characterized by zero partial weak Chern numbers $\nu_1^\pm = \nu_2^\pm = \nu_3^\pm = 0$ and, as we will show in SEq. (4.123) and the surrounding text, $\mathcal{I}$-quantized bulk *partial* axion angles $\theta^\pm = \pi$.

Note that the projectors $P_\pm(\mathbf{k})$ onto the positive/negative spin bands are symmetric under the action of the magnetic subgroup $P\bar{1}$ (# 2.4) of $P\bar{1}1'$ (# 2.5). This implies that we can assign parity eigenvalues to the spin bands at TRIMs, which are inherited from the parity eigenvalues of the occupied bands. We can then compute the corresponding SIs [SEqs. (4.111) and (4.112)] within each of the positive and negative PsP eigenspaces. This allows us to define SIs within the positive/negative PsP eigenspace, which we term the *partial* SIs. To be specific, we define the partial SIs $(\tilde{z}_4^\pm, \tilde{z}_{2,1}^\pm, \tilde{z}_{2,2}^\pm, \tilde{z}_{2,3}^\pm)$ in the same way as SEqs. (4.111) and (4.112) except that the numbers of the $+1$ and -1 inversion eigenvalues at TRIMs are taken from PsP eigenstates in the positive $(+)$ and negative $(-)$ spin bands. We can compute the partial SIs for each spin-stable (spin-gapped) phase in space group $P\bar{1}1'$ (# 2.5) with $\nu_1^\pm = \nu_2^\pm = 0$ using our spin-resolved layer constructions.

Let us first consider the partial SIs for the elementary spin-resolved layer constructions (Supplementary Table 3 and SFig. 20). The elementary spin-resolved layer construction L_1 (WTI) in SFig. 20(a) consists of two copies of the magnetic elementary layer construction for a 3D QAHI with $\nu_3 = 1$ shown in SFig. 18(a), one in each spin subspace, related by time-reversal symmetry. Since the magnetic 3D QAHI with $\nu_3 = 1$ has the magnetic SIs $(\tilde{z}_4, \tilde{z}_{2,1}, \tilde{z}_{2,2}, \tilde{z}_{2,3}) = (2,0,0,1)$, we deduce that the elementary spin-resolved layer construction L_1 has the partial SIs $(\tilde{z}_4^\pm, \tilde{z}_{2,1}^\pm, \tilde{z}_{2,2}^\pm, \tilde{z}_{2,3}^\pm) = (2,0,0,1)$. On the other hand, the elementary spin-resolved layer construction L_2 (oWTI) in

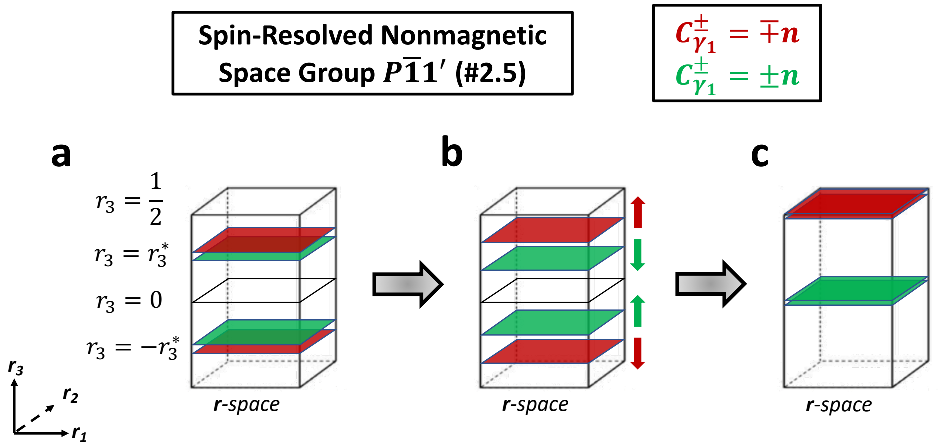

Supplementary Figure 21. Schematic demonstration of the *spin-resolved bubbling process* [210] in position- ($\mathbf{r}$-)space for spin-resolved layer constructions in the nonmagnetic space group $P\bar{1}1'$ (# 2.5) with a spin gap and partial weak Chern numbers $\nu_1^{\pm} = \nu_2^{\pm} = 0$. For any spin-resolved layer construction with $\nu_1^{\pm} = \nu_2^{\pm} = 0$, we can always add two layers within each unit cell in a $\mathcal{I}$-symmetric manner, one with partial Chern number $C_{\gamma_1}^{\pm} = \pm n$ and the other with $C_{\gamma_1}^{\pm} = \mp n$ where $n \in \mathbb{Z}$, to each of the generic $\mathcal{I}$-related $r_3 = r_3^*$ and $r_3 = -r_3^*$ planes as shown in (a). Notice that the slight offset between the two layers with $C_{\gamma_1}^{\pm} = \pm n$ and $C_{\gamma_1}^{\pm} = \mp n$ is a guide to the eye; the two layers should be taken to be in the same $r_3 = r_3^*$ (or $-r_3^*$) plane. In (a), in each of the $r_3 = r_3^*$ and $r_3 = -r_3^*$ planes, we thus have 2D layers with in total zero (trivial) partial Chern numbers. For any spin-resolved layer construction of spin-stable (spin-gapped) phases with $\nu_1^{\pm} = \nu_2^{\pm} = 0$ in the nonmagnetic space group $P\bar{1}1'$ (# 2.5), we can always insert 2D layers in the way depicted in (a) without changing the spin-stable topology by closing and reopening a set of spin-resolved Wannier gaps. Next, we can slide the 2D layers in a manner that respects the symmetries (namely $\mathcal{I}$ and $\mathcal{T}$) of $P\bar{1}1'$ (# 2.5), by sliding the 2D layers with $C_{\gamma_1}^{\pm} = \pm n$ and $C_{\gamma_1}^{\pm} = \mp n$ toward the $r_3 = 0$ and $r_3 = 1/2$ plane, respectively. This is shown schematically in (b). Finally, in (c), we have moved the two 2D layers with $C_{\gamma_1}^{\pm} = \pm n$ to $\mathcal{I}$-invariant $r_3 = 0$ plane, while also moving the two layers with $C_{\gamma_1}^{\pm} = \mp n$ to the $\mathcal{I}$-invariant $r_3 = 1/2$ plane. In terms of the spin-resolved Wannier band configuration, (c) corresponds to $C_{\gamma_2,in}^{\pm} = \pm 2n$ and $C_{\gamma_2,out}^{\pm} = \mp 2n$, which as we have shown in Supplementary Table 2 is consistent with the trivial $\mathbb{Z}_2 \times \mathbb{Z}$ spin-stable invariants $(C_{\gamma_2,in}^{+}, \nu_3^{+}) = (0,0)$. We term the process described by $(a) \to (b) \to (c)$ the *spin-resolved bubbling process* [46, 210]. In particular, inserting 2D layers with (in total) trivial spin-resolved topology in generic $r_3 = r_3^*$ and $r_3 = -r_3^*$ planes in a manner respecting the symmetries of $P\bar{1}1'$ (# 2.5) cannot change the spin-resolved topology, and therefore corresponds to the trivial spin-resolved Wannier band configuration with $C_{\gamma_2,in}^{\pm} = \pm 2n$ and $C_{\gamma_2,out}^{\pm} = \mp 2n$. We refer to spin-stable phases with spin-resolved layer constructions related by the spin-resolved bubbling process as *spin-resolved bubble equivalent* [46, 210].

SFig. 20(b) consists of two copies of the magnetic elementary layer construction for a 3D oQAHI with $\nu_3 = 1$ shown in SFig. 18(b), one in each spin subspace, related by time-reversal symmetry. Since the magnetic 3D oQAHI with $\nu_3 = 1$ has the magnetic SIs $(\tilde{z}_4, \tilde{z}_{2,1}, \tilde{z}_{2,2}, \tilde{z}_{2,3}) = (0,0,0,1)$, we deduce that the elementary spin-resolved layer construction L_1 has the partial SIs $(\tilde{z}_4^{\pm}, \tilde{z}_{2,1}^{\pm}, \tilde{z}_{2,2}^{\pm}, \tilde{z}_{2,3}^{\pm}) = (0,0,0,1)$.

Next, let us consider the two families (SFig. 22) of spin-stable phases built from these two elementary spin-resolved layer constructions L_1 and L_2. The 3D QSHI with $\nu_3^{\pm} = \pm 2$ in SFig. 22(a), which corresponds to $L_1 \oplus L_2$, has partial SIs $(\tilde{z}_4^{\pm}, \tilde{z}_{2,1}^{\pm}, \tilde{z}_{2,2}^{\pm}, \tilde{z}_{2,3}^{\pm}) = (2,0,0,0)$ as the bands in the positive and negative PsP eigenspaces are equivalent to a time-reversed pair of QAHIs with $\nu_3 = \pm 2$ depicted in SFig. 19(a) with magnetic symmetry indicators $(\tilde{z}_4, \tilde{z}_{2,1}, \tilde{z}_{2,2}, \tilde{z}_{2,3}) = (2,0,0,0)$. Similarly, the 3D T-DAXI in SFig. 22(b), which corresponds to $L_1 \ominus L_2$, also has the partial SIs $(\tilde{z}_4^{\pm}, \tilde{z}_{2,1}^{\pm}, \tilde{z}_{2,2}^{\pm}, \tilde{z}_{2,3}^{\pm}) = (2,0,0,0)$ as the bands in the positive and negative PsP eigenspaces are equivalent to the time-reversed pair of AXIs depicted in SFig. 19(b), with the magnetic symmetry indicators $(\tilde{z}_4, \tilde{z}_{2,1}, \tilde{z}_{2,2}, \tilde{z}_{2,3}) = (2,0,0,0)$.

We now investigate the relationship between the spin-stable phases and the stable symmetry-indicated electronic band topology. For topological crystalline phases in the nonmagnetic space group $P\bar{1}1'$ (# 2.5) with a spin gap, we can obtain the $\mathbb{Z}_4 \times (\mathbb{Z}_2)^3$ SIs $(z_4, z_{2,1}, z_{2,2}, z_{2,3})$ for electronic band topology based on the partial SIs $(\tilde{z}_4^{\pm}, \tilde{z}_{2,1}^{\pm}, \tilde{z}_{2,2}^{\pm}, \tilde{z}_{2,3}^{\pm})$ within the positive/negative PsP eigenspaces. The strong $\mathbb{Z}_4$ index for the nonmagnetic space group $P\bar{1}1'$ (# 2.5) is given by [17, 21, 33]

$$z_4 \equiv \frac{1}{4} \sum_{\mathbf{k}_a \in \text{TRIMs}} \left(n_+^a - n_-^a \right) \mod 4, \tag{4.114}$$

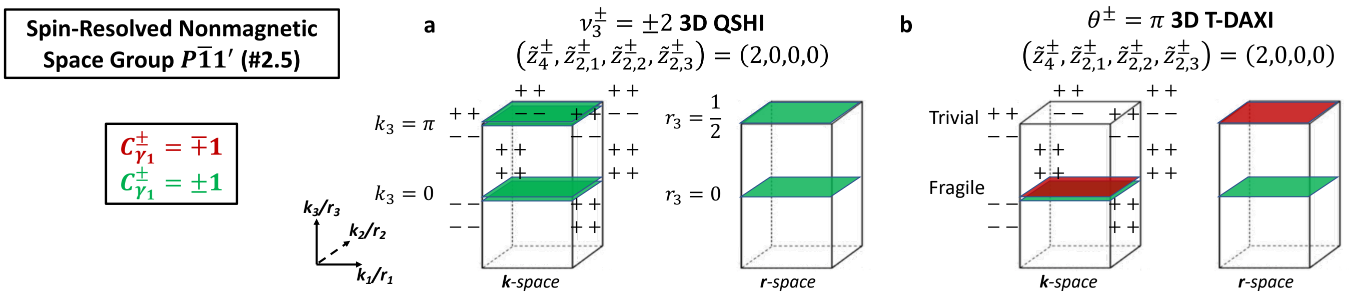

Supplementary Figure 22. Spin-resolved layer constructions for (a) a 3D quantum spin Hall insulator (QSHI) with partial weak Chern numbers [SEq. (4.63)] $\nu_1^{\pm} = \nu_2^{\pm} = 0$ and $\nu_3^{\pm} = \pm 2$, and (b) a 3D $\mathcal{T}$-doubled axion insulator (T-DAXI) characterized by $\nu_1^{\pm} = \nu_2^{\pm} = \nu_3^{\pm} = 0$ and quantized bulk partial axion angles $\theta^{\pm} = \pi$ [SEq. (4.123)]. Both (a) and (b) are in the nonmagnetic space group $P\bar{1}1'$ (# 2.5) with $\nu_1^{\pm} = \nu_2^{\pm} = 0$ and a spin gap. The coordinates k_i and r_i $(i = 1, 2, 3)$ in momentum($\mathbf{k}$)-space and position($\mathbf{r}$)-space are given by $\mathbf{k} = \sum_{i=1}^{3} \frac{k_i}{2\pi} \mathbf{G}_i$ and $\mathbf{r} = \sum_{i=1}^{3} r_i \mathbf{a}_i$ respectively. $\{\mathbf{a}_1, \mathbf{a}_2, \mathbf{a}_3\}$ are the $\mathbf{r}$-space primitive lattice vectors and $\{\mathbf{G}_1, \mathbf{G}_2, \mathbf{G}_3\}$ are the dual $\mathbf{k}$-space primitive reciprocal lattice vectors such that $\mathbf{a}_i \cdot \mathbf{G}_j = 2\pi\delta_{ij}$ $(i, j = 1, 2, 3)$. The $\mathcal{I}$-invariant constant-r_3 planes contain $r_3 = 0$ and $r_3 = 1/2$, which correspond to the center and the boundary of the primitive unit cell along $\mathbf{a}_3$. Similarly, the $\mathcal{I}$-symmetric constant-k_3 planes contain $k_3 = 0$ and $k_3 = \pi$, which correspond to the center and the boundary of the BZ along $\mathbf{G}_3$. The right-hand side of (a) shows that to layer-construct a 3D QSHI with $\nu_3^{\pm} = \pm 2$, we can place translation-, $\mathcal{I}$-, and spinful $\mathcal{T}$-symmetric 2D layers with partial Chern numbers $C^{\pm}_{\gamma_1} = \pm 1$ in both the $r_3 = 0$ and $r_3 = 1/2$ planes within the primitive unit cell in $\mathbf{r}$-space. The left-hand side of (a) shows that the occupied energy bands in $\mathbf{k}$-space for such a layer construction in $\mathbf{r}$-space on the right-hand side of (a) are two spin-gapped insulators, each with partial Chern numbers $C^{\pm}_{\gamma_1} = \pm 1$, in both the $k_3 = 0$ and $k_3 = \pi$ planes. The right-hand side of (b) shows that to layer-construct a 3D T-DAXI, we place a 2D layer with partial Chern numbers $C^{\pm}_{\gamma_1} = \pm 1$ in the $r_3 = 0$ plane and another 2D layer with partial Chern numbers $C^{\pm}_{\gamma_1} = \mp 1$ in the $r_3 = 1/2$ plane within the primitive unit cell in the $\mathbf{r}$-space. The left-hand side of (b) shows that the occupied energy bands in $\mathbf{k}$-space for such a layer construction in $\mathbf{r}$-space on the right-hand side of (b) are two 2D spin-gapped layers in the $k_3 = 0$ plane, one with partial Chern numbers $C^{\pm}_{\gamma_1} = \pm 1$ and the other with $C^{\pm}_{\gamma_1} = \mp 1$, such that the total partial Chern numbers are zero in the $k_3 = 0$ plane. In the $k_3 = \pi$ plane of panel (b), the occupied spin bands carry trivial stable and fragile topology. The inversion eigenvalues at the eight TRIMs compatible with the layer constructions for (a) and (b) with four occupied electronic energy bands are also shown individually on the left-hand side of each panel. The $\mathbb{Z}_4 \times (\mathbb{Z}_2)^3$ partial SIs $(\tilde{z}_4^{\pm}, \tilde{z}_{2,1}^{\pm}, \tilde{z}_{2,2}^{\pm}, \tilde{z}_{2,3}^{\pm})$ are determined to be $(2, 0, 0, 0)$ for both (a) and (b). Notably, on the left-hand side of (b), the four occupied energy bands in the $k_3 = 0$ plane for a 3D T-DAXI have 2D fragile topology in the nonmagnetic space group $P\bar{1}1'$ (# 2.5), as will be numerically demonstrated in SN 4 E. However, as emphasized in SFig. 19, the four occupied bands taken throughout the 3D BZ in *both* (a) and (b) carry the *stable* topology of a helical HOTI. We additionally note that (a) and (b) correspond to the $L_1 \oplus L_2$ and $L_1 \ominus L_2$ spin-resolved layer constructions in Supplementary Table 3, respectively.

and the three weak $\mathbb{Z}_2$ indices for the nonmagnetic space group $P\bar{1}1'$ (# 2.5) are given by [142]

$$z_{2,i} \equiv \frac{1}{4} \sum_{\substack{\mathbf{k}_a \in \text{TRIMs} \\ \mathbf{k}_a \cdot \mathbf{a}_i = \pi}} \left(n_+^a - n_-^a \right) \mod 2, \tag{4.115}$$

where $i = 1, 2, 3$. Using SEqs. (4.111), (4.112), (4.114), and (4.115), together with the facts that $[\mathcal{I}, \mathcal{T}] = 0$, the positive and negative PsP eigenspaces in the occupied energy bands are orthogonal to each other, and that the numbers of positive and negative inversion eigenvalues are additive, we deduce that for topological crystalline phases in the nonmagnetic space group $P\bar{1}1'$ (# 2.5) with a spin gap we have

$$(z_4, z_{2,1}, z_{2,2}, z_{2,3}) = (\tilde{z}_4^+, \tilde{z}_{2,1}^+, \tilde{z}_{2,2}^+, \tilde{z}_{2,3}^+) = (\tilde{z}_4^-, \tilde{z}_{2,1}^-, \tilde{z}_{2,2}^-, \tilde{z}_{2,3}^-). \tag{4.116}$$

Therefore, we can tabulate the SIs and partial SIs of the elementary spin-resolved layer constructions L_1 and L_2 [SFig. 20(a,b)] for topological crystalline phases in nonmagnetic $P\bar{1}1'$ (# 2.5) with a spin gap and $\nu_1^{\pm} = \nu_2^{\pm} = 0$, as shown in Supplementary Table 4. The additivity of the symmetry indicators and of the $(C^+_{\gamma_2,in} \mod 2, \nu_3^+)$ $\mathbb{Z}_2 \times \mathbb{Z}$ invariant allows us to deduce that the SIs $(z_4, z_{2,1}, z_{2,2}, z_{2,3})$ [SEqs. (4.114) and (4.115)] for the topological crystalline phases in the nonmagnetic space group $P\bar{1}1'$ (# 2.5) with $\nu_1^{\pm} = \nu_2^{\pm} = 0$ and a spin gap are given by

$$z_4 = 2(C^+_{\gamma_2,in} \mod 2), \tag{4.117}$$

$$z_{2,1} = 0, \tag{4.118}$$

$$z_{2,2} = 0, \tag{4.119}$$

$$z_{2,3} = \nu_3^+ \mod 2. \tag{4.120}$$

Note that the existence of a spin gap requires that z_4 be even; this is because $z_4 = 1, 3$ correspond to 3D strong topological insulators, which are spin-Weyl semimetals as detailed in SN 2 E, 3 E, and 3 F. This is consistent with the fact that the partial symmetry indicators $\tilde{z}_4^{\pm}$ and the magnetic symmetry indicator $\tilde{z}_4$ cannot be odd for layer-constructable (*i.e.* spin-gapped or insulating) phases.

We emphasize that spin-resolved topology is a refinement of electronic (energy) band topology for systems with a spin gap. Systems with distinct spin-stable topology but equivalent electronic (energy) band topology can be deformed into each other without closing an energy gap provided a spin gap closes. We summarize this in Supplementary Table 5, where we show how the spin-stable invariants ($C^{+}_{\gamma_2,in} \mod 2, \nu_3^{+}$) characterizing spin-stable topological phases with $\nu_1^{\pm} = \nu_2^{\pm} = 0$ in space group $P\bar{1}1'$ (# 2.5) collapse onto the symmetry indicators of (energy) band topology [SEqs. (4.117)–(4.120)] when a spin gap is allowed to close. We emphasize that since $[\mathcal{I}, \mathcal{T}] = 0$, a spin band inversion without an energy band inversion cannot trivialize the partial SIs. This represents the 3D generalization of the statement that the spin Chern number $C^{s}_{\gamma_1}$ of a 2D TI can be changed without closing an energy gap, but cannot go to zero without closing an energy gap or breaking $\mathcal{T}$ symmetry (*i.e.* $(C^{s}_{\gamma_1}/2) \mod 2 = 1$ in a 2D TI state, see SRef. [1] and SN 3 C).

Let us now examine the $L_1 \oplus L_2$ $\nu_3^{\pm} = \pm 2$ QSHI and the $L_1 \ominus L_2$ T-DAXI in SFig. 22 in more detail. From Supplementary Table 4 we find that both the QSHI and T-DAXI have the SIs $(z_4, z_{2,1}, z_{2,2}, z_{2,3}) = (2, 0, 0, 0)$. This implies that the QSHI and T-DAXI represent distinct spin resolutions of the same symmetry-indicated stable topological crystalline phase in the nonmagnetic space group $P\bar{1}1'$ (# 2.5). Note that $(z_4, z_{2,1}, z_{2,2}, z_{2,3}) = (2, 0, 0, 0)$ corresponds to a symmetry-indicated helical HOTI. On the other hand, in terms of the $\mathbb{Z}_2 \times \mathbb{Z}$ spin-stable invariants $(C^{+}_{\gamma_2,in}, \nu_3^{+})$, the QSHI has $(C^{+}_{\gamma_2,in} \mod 2, \nu_3^{+}) = (1, 2)$ while the T-DAXI has $(C^{+}_{\gamma_2,in} \mod 2, \nu_3^{+}) = (1, 0)$ (Supplementary Tables 2 and 3). Hence, although the QSHI [SFig. 22(a)] and T-DAXI [SFig. 22(b)] collapse to the same symmetry-indicated stable topological crystalline phase in the nonmagnetic space group $P\bar{1}1'$ (# 2.5) without spin resolution, they realize distinct spin-stable phases. We will discuss how these differences manifest in physical observables in the remainder of this section below.

	(Partial) Symmetry Indicators and Spin-Stable Invariants		
	$(\tilde{z}_4^{\pm}, \tilde{z}_{2,1}^{\pm}, \tilde{z}_{2,2}^{\pm}, \tilde{z}_{2,3}^{\pm})\}$	$(z_4, z_{2,1}, z_{2,2}, z_{2,3})$	$(C^{+}_{\gamma_2,in} \mod 2, \nu_3^{+})$
L_1 [WTI in SFig. 20(a)]	$(2, 0, 0, 1)$	$(2, 0, 0, 1)$	$(1, 1)$
L_2 [oWTI in SFig. 20(b)]	$(0, 0, 0, 1)$	$(0, 0, 0, 1)$	$(0, 1)$
$2nL_1 \ominus 2nL_2$ $(n \in \mathbb{Z})$ (trivial spin-stable phase in SFig. 21)	$(0, 0, 0, 0)$	$(0, 0, 0, 0)$	$(0, 0)$
$L_1 \oplus L_2$ [QSHI in SFig. 22(a)]	$(2, 0, 0, 0)$	$(2, 0, 0, 0)$	$(1, 2)$
$L_1 \ominus L_2$ [T-DAXI in SFig. 22(b)]	$(2, 0, 0, 0)$	$(2, 0, 0, 0)$	$(1, 0)$

Supplementary Table 4. Partial SIs $(\tilde{z}_4^{\pm}, \tilde{z}_{2,1}^{\pm}, \tilde{z}_{2,2}^{\pm}, \tilde{z}_{2,3}^{\pm})$, SIs $(z_4, z_{2,1}, z_{2,2}, z_{2,3})$, and the $\mathbb{Z}_2 \times \mathbb{Z}$ spin-stable invariants $(C^{+}_{\gamma_2,in} \mod 2, \nu_3^{+})$ for different spin-resolved layer constructions of topological crystalline phases in the nonmagnetic space group $P\bar{1}1'$ (# 2.5) with a spin gap and partial weak Chern numbers $\nu_1^{\pm} = \nu_2^{\pm} = 0$. We see that $L_1 \oplus L_2$ and $L_1 \ominus L_2$ have distinct spin-resolved topology, as their invariants $(C^{+}_{\gamma_2,in} \mod 2, \nu_3^{+})$ do not satisfy SEq. (4.110). However, we also see that $L_1 \oplus L_2$ and $L_1 \ominus L_2$ have the same SIs $(z_4, z_{2,1}, z_{2,2}, z_{2,3}) = (2, 0, 0, 0)$. The corresponding symmetry-indicated electronic band topology for the spin-stable phases are given in Supplementary Table 5.

	$(C^{+}_{\gamma_2,in}, \nu_3^{+})$					
	$(1, 1)$	$(0, 1)$	$(0, 0)$	$(0, 2)$	$(1, 2)$	$(1, 0)$
$(z_4, z_{2,1}, z_{2,2}, z_{2,3})$	$(2, 0, 0, 1)$	$(0, 0, 0, 1)$	$(0, 0, 0, 0)$	$(0, 0, 0, 0)$	$(2, 0, 0, 0)$	$(2, 0, 0, 0)$
Symmetry-indicated topological phase	WTI	oWTI	trivial	trivial	helical HOTI	helical HOTI

Supplementary Table 5. SIs $(z_4, z_{2,1}, z_{2,2}, z_{2,3})$ [SEqs. (4.117), (4.118), (4.119), and (4.120)] and the corresponding stable symmetry-indicated topological crystalline phases in nonmagnetic space group $P\bar{1}1'$ (# 2.5) with a spin gap and $\nu_1^{\pm} = \nu_2^{\pm} = 0$ for the spin-stable phases with $(C^{+}_{\gamma_2,in}, \nu_3^{+})$ given in Supplementary Tables 2 and 3. The stable symmetry-indicated topological crystalline phases include a trivial insulator, weak topological insulator (WTI) [141, 142], obstructed weak topological insulator (oWTI) [92], and a helical higher-order topological insulator (HOTI). As we can see, although the spin-stable phases with $(C^{+}_{\gamma_2,in}, \nu_3^{+}) = (1, 2)$ and $(1, 0)$ are both symmetry-indicated helical HOTIs, they have distinct spin-resolved topology, as the difference between their $(C^{+}_{\gamma_2,in}, \nu_3^{+})$ violates SEq. (4.110). Similarly, although both $(C^{+}_{\gamma_2,in}, \nu_3^{+}) = (0, 0)$ and $(0, 2)$ are symmetry-indicated trivial insulators, they have distinct spin-resolved topology, as $(0, 2)$ corresponds to tiling 3D space with 2D layers carrying the partial Chern numbers $C^{\pm}_{\gamma_1} = \pm 2$ in the $r_3 = 1/2$ plane within the primitive unit cell.

Both the 3D QSHI and T-DAXI in SFig. 22(a,b) respectively are symmetry-indicated helical HOTIs, as they both have zero weak $\mathbb{Z}_2$ invariants ($z_{2,1} = z_{2,2} = z_{2,3} = 0$) and the strong $\mathbb{Z}_4$ invariant $z_4 = 2$ (Supplementary Table 5). In addition, both have odd nonzero winding numbers $C^{\pm}_{\gamma_2,in/out}$ in their nested partial Berry phases (Supplementary Table 5), indicating that we cannot form time-reversed pairs of inversion-symmetric exponentially localized Wannier functions [123]. However, the QSHI and T-DAXI in SFig. 22 differ from each other in their spin-resolved response to external fields. Let us again restrict to the spin-stable (spin-gapped) phases in the nonmagnetic space group $P\bar{1}1'$ (# 2.5) with partial weak Chern numbers $\nu_1^{\pm} = \nu_2^{\pm} = 0$. To see that their responses differ, let us first consider the limit in which the spin component $\hat{\mathbf{n}} \cdot \mathbf{s}$ is conserved. In this limit, the bulk spin Hall conductivity for the QSHI [SFig. 22(a)] and T-DAXI [SFig. 22(b)] per unit cell are given by, according to SEq. (4.73),

$$\sigma^s_{ij,\mathrm{top,QSHI}} = \frac{e}{4\pi}\epsilon_{ijk} \cdot \left(\nu_3^+ - \nu_3^-\right)(\mathbf{G}_3)_k = \frac{e}{4\pi} \cdot \left(2\nu_3^+\right)\epsilon_{ijk}(\mathbf{G}_3)_k = \frac{e}{4\pi} \cdot 4\epsilon_{ijk}(\mathbf{G}_3)_k, \tag{4.121}$$

$$\sigma^s_{ij,\mathrm{top,T-DAXI}} = 0, \tag{4.122}$$

where we have used SEq. (4.67) in the presence of spinful $\mathcal{T}$ symmetry. As noted in SEq. (3.36), in the presence of weak $\hat{\mathbf{n}} \cdot \mathbf{s}$ non-conserving SOC, we expect SEqs. (4.121) and (4.122) to give the leading-order bulk topological contributions to $\sigma^s_{ij,\mathrm{QSHI}}$ and $\sigma^s_{ij,\mathrm{T-DAXI}}$, respectively. As such, the spin Hall conductivity between QSHI and T-DAXI can be greatly different, even when spin is not conserved. We will later numerically demonstrate that a T-DAXI has a vanishing bulk topological contribution to the spin Hall conductivity by computing its layer-resolved partial Chern numbers in SN 5 E.

On the other hand, from the relation between the winding numbers of the nested Berry phases and the bulk axion angle introduced in SRef. [15], our nested spin-resolved Wilson loop formalism (SN 4 B) allows us to introduce *partial axion angles* $\theta^{\pm}$, which respectively correspond to the 3D bulk axion angle [77] within each of the positive and negative PsP eigenspaces. In analogy with the ordinary axion angle, we can use the layer constructions in Supplementary Table 3 to define the partial axion angle as $2\pi/|\mathbf{a}_3|$ times the partial Chern number polarization per unit cell (taken modulo 2π) [15, 16]. For the T-DAXI in SFig. 22(b), this gives

$$\theta^{\pm}_{\mathrm{T-DAXI}} = 2\pi[0(\pm 1) + \frac{1}{2}(\mp 1)] \mod 2\pi = \pi. \tag{4.123}$$

The bulk contribution to the isotropic magnetoelectric polarizability [77] *separately in the positive and negative eigenspace* of PsP is then given by

$$\alpha^{\pm} = \theta^{\pm}\frac{e^2}{2\pi h}, \tag{4.124}$$

where $\alpha^{\pm}$ is the *partial magnetoelectric polarizability*, which represents the 3D analog of the 1D partial polarization introduced by Fu and Kane in SRef. [2]. In the T-DAXI state [SFig. 22(b)], $\theta^{\pm} \mod 2\pi = \pi$, a result that is *origin-independent*, because the bulk partial weak Chern numbers all vanish [16, 36, 92]. Conversely in a 3D QSHI state [SFig. 22(a)], the partial Chern numbers per unit cell are nonvanishing, and the partial axion angles $\theta^{\pm}$ are hence origin-dependent.

We can combine this result with our arguments about flux insertion from SN 3 G to understand the response of QSHIs and T-DAXIs in SFig. 22(a,b) respectively to the insertion of a magnetic flux. Viewing a QSHI via the layer construction from Supplementary Table 3, we see that the spin Hall conductivity in SEq. (4.121) implies that the intrinsic spin Hall conductivity of a quasi-2D slab of a QSHI is proportional to the thickness of the slab. Inserting a π magnetic flux tube along the $\mathbf{a}_3$ axis into a $\nu_3^{\pm} \neq 0$ QSHI will thus bind an extensive number of mid-gap states localized at the flux tube. Conversely, the vanishing partial Chern number in each unit cell of a T-DAXI implies that a π-flux tube in a T-DAXI will not bind any states in the bulk. Instead, we expect the flux-insertion response of a T-DAXI to manifest on surfaces. Specifically by analogy to magnetic AXIs, the partial Chern number polarization in a T-DAXI implies that gapped surfaces with normal vectors parallel to $\pm\mathbf{G}_3$ have the response associated with a half-quantized partial Chern number [36, 92, 174, 220]. From the results of SRefs. [184, 185, 189], we hence expect a π-flux tube in a finite slab of a T-DAXI to bind one spinon between the top and bottom surface at half filling. This is consistent with the fact that, due to the presence of a sample-encircling helical hinge mode, a finite crystallite of a T-DAXI with global $\mathcal{I}$-symmetry, when viewed as a quasi-2D system, is a 2D strong TI. In SN 5 we will formalize the notion of a half-quantized surface partial Chern number by developing a layer-resolved marker for the partial Chern number in position space, which reveals that the gapped surfaces of helical HOTIs in the T-DAXI regime bind anomalous quantum spin Hall states with half-integral ($n + 1/2$ where $n \in \mathbb{Z}$) partial Chern numbers, which are

equivalent to anomalous halves of 2D TIs. Concurrent with the preparation of this work, a numerical investigation of π-flux insertion in helical HOTIs revealed that a π-flux tube, on the average, binds half a spinon per surface in a helical HOTI [174], which is consistent with the above argument and the results of this work. This suggests it would be interesting to explore the partial axion angles $\theta^{\pm}$ and the associated spin-magnetoelectric response from the perspective of topological quantum field theory, which we leave as an exciting direction for future investigation.

To summarize, our spin-resolved formalism of (nested) Wilson loops in SN 3 B and 4 B has allowed us to identify two distinct forms of spin-stable topology in symmetry-indicated helical HOTIs in the nonmagnetic space group $P\bar{1}1'$ (# 2.5) with a spin gap, $\nu_1^{\pm} = \nu_2^{\pm} = 0$, and spin-resolved Wannier band configurations described by $(C^{+}_{\gamma_2,in} \bmod 2, \nu_3^{+}) = (1,2)$ and $(1,0)$ [SFig. 22(a,b)]:

1. The spin-resolved Wilson loop formalism from SN 3 B allows us to compute the partial weak Chern numbers, which characterize distinct topological contributions to the bulk and surface spin Hall conductivity that distinguish between 3D QSHIs and T-DAXIs [SEqs. (4.121) and (4.122)].

2. The nested spin-resolved Wilson loop formalism from SN 4 B allows us to deduce that both 3D QSHIs [SFig. 22(a)] and T-DAXIs [SFig. 22(b)] with odd winding numbers $C^{\pm}_{\gamma_2,in/out}$ in their nested partial Berry phases (Supplementary Table 2) cannot form 3D inversion- and time-reversal-symmetric exponentially localized Wannier functions. Furthermore, since the nested $P_{\pm}$-Wilson loop winding provides information regarding the partial Chern numbers of the spin-resolved hybrid Wannier bands in position space, it also allows us to discover that beyond the spin Hall conductivity, there also exists a partial 3D magnetoelectric polarizability in spinful $\mathcal{T}$-invariant 3D insulators with vanishing weak $\mathbb{Z}_2$ indices, which can be considered the 3D generalization of the 1D partial polarization introduced by Fu and Kane in SRef. [2]. We can then deduce the spin-electromagnetic response of 3D QSHIs [SFig. 22(a)] and T-DAXIs [SFig. 22(b)] to external magnetic fields via the spin-resolved layer construction method introduced in this work (Supplementary Table 3, Supplementary Table 4, SFig. 20, and SFig. 22).

We summarize in Supplementary Table 6 the properties of 3D QSHIs [SFig. 22(a)] and 3D T-DAXIs [SFig. 22(b)] derived in this section. In SN 10 we will demonstrate that the candidate helical HOTI α-BiBr [50, 52, 53] realizes both 3D QSHI and T-DAXI states, depending on the spin resolution direction. We will specifically show that if α-BiBr is spin-resolved along s_z, it realizes a 3D QSHI state with a partial Chern vector $\boldsymbol{\nu}^{\pm} = \mp 2\mathbf{G}_3$ and the nested spin-resolved Wilson loop winding numbers (identified as the nested partial Chern numbers [SEq. (4.64)]) $C^{\pm}_{\gamma_2,in} = \mp 1$, and $C^{\pm}_{\gamma_2,out} = \mp 1$. Furthermore, we excitingly find that if α-BiBr is spin-resolved along s_x, it realizes a T-DAXI state with a partial Chern vector $\boldsymbol{\nu}^{\pm} = \mathbf{0}$ and the nested spin-resolved Wilson loop winding numbers (identified as the nested partial Chern numbers [SEq. (4.64)]) $C^{\pm}_{\gamma_2,in} = \mp 1$, $C^{\pm}_{\gamma_2,out} = \pm 1$, which are indicative of origin-independent nontrivial partial axion angles $\theta^{\pm} = \pi$.

Spin-Stable Phases	3D QSHI with $\nu_3^{\pm} = \pm 2$ [SFig. 22(a)]	3D T-DAXI [SFig. 22(b)]
$(C^{+}_{\gamma_2,in}, \nu_3^{+})$	$(1,2)$	$(1,0)$
$C^{\pm}_{\gamma_2,in}$	± 1	± 1
$C^{\pm}_{\gamma_2,out}$	± 1	∓ 1
Spin-Resolved Layer Construction	$C^{\pm}_{\gamma_1} = \pm 1$ in $r_3 = 0$, $C^{\pm}_{\gamma_1} = \pm 1$ in $r_3 = \frac{1}{2}$	$C^{\pm}_{\gamma_1} = \pm 1$ in $r_3 = 0$, $C^{\pm}_{\gamma_1} = \mp 1$ in $r_3 = \frac{1}{2}$
Partial Symmetry Indicators $(\tilde{z}_4^{\pm}, \tilde{z}_{2,1}^{\pm}, \tilde{z}_{2,2}^{\pm}, \tilde{z}_{2,3}^{\pm})$	$(2,0,0,0)$	$(2,0,0,0)$
Symmetry Indicators $(z_4, z_{2,1}, z_{2,2}, z_{2,3})$	$(2,0,0,0)$	$(2,0,0,0)$
Symmetry-Indicated Topological Phase	helical HOTI	helical HOTI
Topological Contribution to the Spin Hall Conductivity	$\frac{e}{4\pi} \cdot 4\epsilon_{ijk}(\mathbf{G}_3)_k$	0
Partial Axion Angles $\theta^{\pm}$ mod 2π	origin-dependent	π

Supplementary Table 6. Spin-resolved properties of helical HOTIs in nonmagnetic space group $P\bar{1}1'$ (# 2.5) with a spin gap, $\nu_1^{\pm} = \nu_2^{\pm} = 0$ (SFig. 22), and different spin-stable topology.

Finally, let us make two further remarks. First, since the 3D QSHI and 3D T-DAXI spin-stable phases discussed in this section (see also the summary in Supplementary Table 6) are both symmetry-indicated helical HOTIs in the nonmagnetic space group $P\bar{1}1'$ (# 2.5) with a spin gap and $\nu_1^{\pm} = \nu_2^{\pm} = 0$, they can be deformed into each other while keeping the energy gap open and preserving inversion and time-reversal symmetry. However, since their partial weak Chern numbers ν_3^{+} differ by 2, a gap must still close in the *spin* spectrum during such a deformation. In particular, the 3D QSHI [3D T-DAXI] considered in this section carries the partial Chern numbers $C^{\pm}_{\gamma_1}(k_3) = \pm 2$ [$C^{\pm}_{\gamma_1}(k_3) = 0$] in both the $k_3 = 0$ and $k_3 = \pi$ planes (see also SFig. 22). The intermediate spin-gapless phase during the deformation will hence have the partial Chern numbers $C^{\pm}_{\gamma_1}(k_3 = 0) = \pm 2$ and $C^{\pm}_{\gamma_1}(k_3 = \pi) = 0$, or vice-versa, but we expect it to have non-vanishing, nonquantized partial axion angles (by analogy to the nonquantized axion angles in Weyl

semimetals [221–223]). This implies that the intermediate phase is a spin-Weyl semimetal state with an even number of spin-Weyl nodes in each half of the BZ. We will show in SN 9 that the candidate helical HOTI β-MoTe$_2$ exhibits a spin band structure that lies within the spin-Weyl semimetal regime for all choices of $\hat{\mathbf{n}}\cdot\mathbf{s}$. We will also show in SN 10 that the candidate helical HOTI α-BiBr interpolates between 3D QSHI and T-DAXI regimes through an intermediate spin-Weyl state as the spin resolution direction is rotated from $s_z = \hat{\mathbf{z}}\cdot\mathbf{s}$ to $s_x = \hat{\mathbf{x}}\cdot\mathbf{s}$.

To understand the intermediate spin-Weyl semimetal phase that lies between 3D QSHI and T-DAXI states, recall from SN 3 E that a 3D strong topological insulator has an odd number of spin-Weyl nodes within each half of the 3D BZ. This implies that the intermediate spin-Weyl semimetal phase between a 3D QSHI and a 3D T-DAXI can be constructed by superposing two identical 3D strong topological insulators, in fact realizing the doubled strong topological insulator (DSTI) introduced in SRef. [17], which as discussed in the main text, represents one construction of a symmetry-indicated helical HOTI. Therefore, through the analysis in this section of spin-stable topology and deformations between distinct spin-stable phases, we have found that the minimal model of a helical HOTI in the nonmagnetic space group $P\bar{1}1'$ (# 2.5) with a spin gap and $\nu_1^{\pm} = \nu_2^{\pm} = 0$ realizes one of three spin-resolved phases with distinct spin-stable topology: a 3D QSHI with $\nu_3^{\pm} = \pm 2$ [SFig. 22(a)], a T-DAXI with $\theta^{\pm} \mod 2\pi = \pi$ [SFig. 22(b) and SEq. (4.123)], or a DSTI with an even number of spin-Weyl points per half BZ.

Second, although we have in this section considered a 3D QSHI with $(C^+_{\gamma_2,in} \mod 2, \nu_3^+) = (1,2)$, there can also exist 3D QSHIs with $(C^+_{\gamma_2,in}, \nu_3^+) = (0,2)$. Spin-resolved Wannier band configurations consistent with $(C^+_{\gamma_2,in} \mod 2, \nu_3^+) = (1,2)$ and $(0,2)$ have the same topological contribution to the spin Hall conductivity, as their partial weak Chern numbers $\nu_3^{\pm}$ are equal. However, although a 3D QSHI with $(C^+_{\gamma_2,in} \mod 2, \nu_3^+) = (1,2)$ is a symmetry-indicated helical HOTI, 3D QSHIs with $(C^+_{\gamma_2,in} \mod 2, \nu_3^+) = (0,2)$ are symmetry-indicated trivial insulators, as shown in Supplementary Table 5. This means that in the presence of s-nonconserving SOC, the $(1,2)$ QSHI remains topologically nontrivial even if a spin gap closes, while the $(0,2)$ state can be trivialized by deformations that close and reopen the spin gap (and include sufficient trivial bands to trivialize any fragile topology that may arise in few-band models).

In conclusion, we have shown that spin-resolved band topology provides a refinement of the classification of symmetry-indicated helical HOTIs and the layer construction method. Using the (nested) $P_{\pm}$-Wilson loop formalism developed in SN 3 B and 4 B, we have identified distinct spin-stable helical HOTI phases that cannot be deformed into each other without closing either an energy gap or a spin gap. We have used the method of spin-resolved layer constructions introduced in this work to demonstrate that insulators with distinct spin-stable topology, even if they share the same symmetry-indicated electronic band topology without spin resolution, exhibit distinct spin-electromagnetic responses. In the next section (SN 4 E), we will apply the theoretical techniques developed in this section to numerically analyze spin-resolved topology in a model of a helical HOTI.

E. Numerical Calculations for Nested $P_{\pm}$-Wilson Loops of an $\mathcal{I}$- and $\mathcal{T}$-Symmetric Helical HOTI

We now apply the formalism of nested P- and $P_{\pm}$-Wilson loop from SN 4 A and 4 B to analyze the 3D bulk topology of a helical HOTI with inversion ($\mathcal{I}$) and spinful time-reversal ($\mathcal{T}$) symmetry. Provided that the spin gap defined in SN 2 B is open, we may compute the bulk spin-resolved topology of the helical HOTI by considering either the positive (upper) or negative (lower) PsP eigenspace. Through the analysis of spin-resolved topology we will show that our model of the helical HOTI is in the T-DAXI regime.

As in the case of magnetic axion insulators (AXIs), whose bulk axion angles θ can be computed from the spectral flow of the *nested* P-Wilson loop eigenphases (see SN 4 A and SRef. [15]), we will here show how the nested $P_{\pm}$-Wilson loop allows us to compute the spin-resolved (partial) bulk topological invariants of time-reversal and inversion symmetric helical HOTIs. As demonstrated in SN 4 D and SRef. [174], the spin-resolved (partial) bulk topological invariants in helical HOTIs manifest as response coefficients of bulk spin-electromagnetic effects. Just like with AXIs, we will see that care must be taken to add extra trivial bands to our models in order to remove fragile winding in the P- and $P_{\pm}$-Wilson loops [15]. Specifically, although AXIs and helical HOTIs are stable topological crystalline insulators [15–19, 33, 43–45, 78, 106], minimal models of AXIs and helical HOTIs exhibit gapless Wilson loops, whereas models with larger number of (occupied) bands generally do not [15, 16]. We consider an eight-band helical HOTI with a 3D orthorhombic lattice, formed by placing two spinful s and two spinful ip orbitals at the $1a$ Wyckoff position $[(x,y,z) = (0,0,0)]$ of the primitive unit cell. Symmetry-breaking hopping terms are included to break accidental symmetries while preserving $\mathcal{I}$ and $\mathcal{T}$ symmetries [31]. Normalizing the lattice constants to one, we have that the primitive reciprocal lattice vectors are $\mathbf{G}_1 = 2\pi\hat{\mathbf{x}}$, $\mathbf{G}_2 = 2\pi\hat{\mathbf{y}}$, and $\mathbf{G}_3 = 2\pi\hat{\mathbf{z}}$. The corresponding eight-band

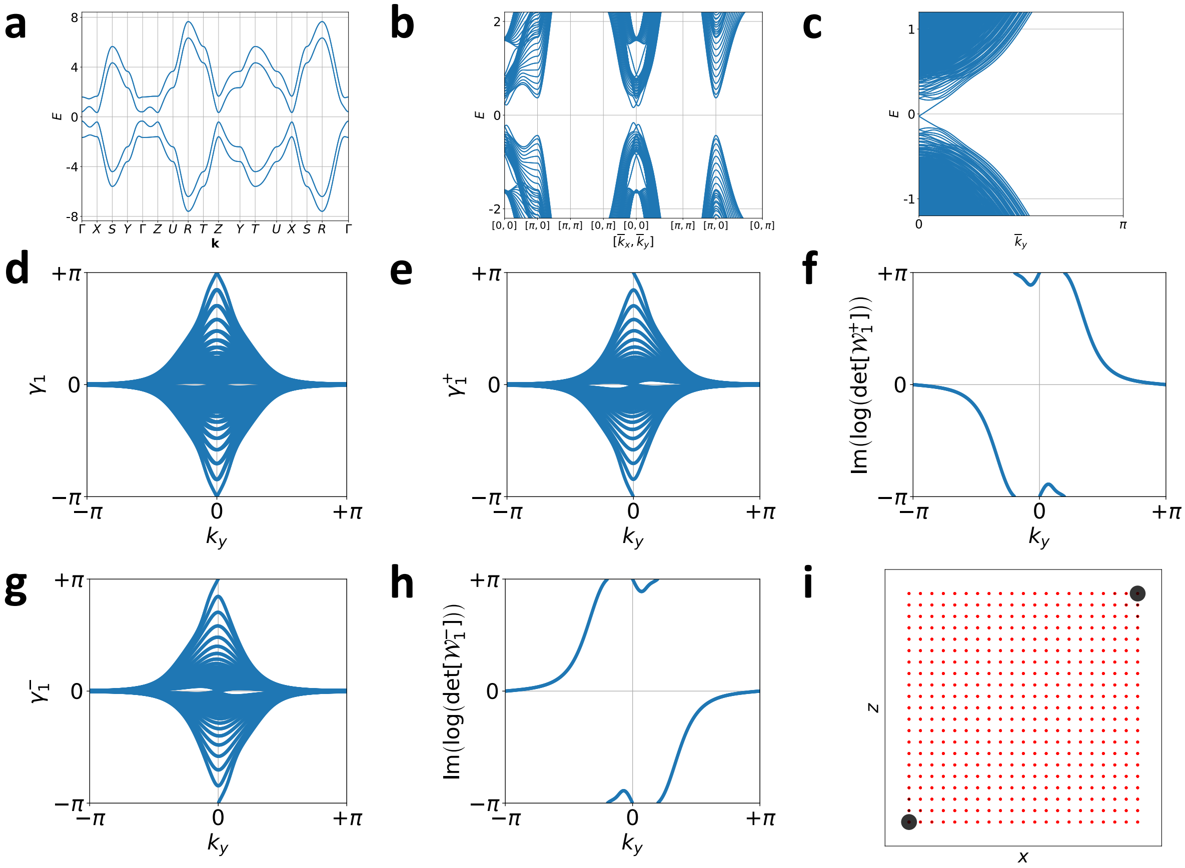

Supplementary Figure 23. Bulk and boundary spectrum of the eight-band helical HOTI tight-binding model introduced in SRef. [31], which has been reproduced in a slightly modified form in SEqs. (4.125), (4.128) and (4.129). (a) shows the 3D bulk band structure. The labels of the high-symmetry points are given in SFig. 4(a). (b) shows the 2D band structure of a slab infinite along x and y while finite along z with 21 unit cells. (c) shows the 1D band structure of a rod infinite along y while finite along x and z with size 21×21. Here "k_x-directed" means that we have chosen $\mathbf{G} = 2\pi\hat{\mathbf{x}}$ in the P- and $P_{\pm}$-Wilson loop matrices [SEqs. (3.2) and (3.17)]. (d) shows the k_x-directed P-Wilson loop eigenphases as a function of k_y for a 2D slab infinite along x and y while finite along z with 21 unit cells. There is an odd helical winding with band crossings at $k_y = 0$ and π protected by the spinful $\mathcal{T}$ symmetry in (d). (e) shows the k_x-directed P_+-Wilson loop eigenphases as a function of k_y for the same 2D slab model in (d). (f) shows the sum of the k_x-directed P_+-Wilson loop eigenphases in (e), which exhibit an overall -1 winding number. (g) shows the k_x-directed P_--Wilson loop eigenphases as a function of k_y for the same 2D slab in (d). (h) shows the sum of the k_x-directed P_--Wilson loop eigenphases of (g), which exhibit an overall $+1$ winding number. (d)–(h) collectively establish that the inversion-symmetric 2D slab of a helical HOTI is a time-reversal invariant strong topological insulator with nontrivial strong $\mathbb{Z}_2$ invariant $\nu_{2d} = 1$ [15, 92, 94]. (i) shows the averaged probability distribution of the four in-gap modes with $k_y = 0$ in (c). In particular, when the system is terminated in a $\hat{\mathbf{y}}$-directed rod geometry preserving both $\mathcal{I}$ and $\mathcal{T}$ symmetries as in (c), the 1D metallic states at the same hinge are related to each other by $\mathcal{T}$ symmetry; states at opposite hinges are related by $\mathcal{I}$ symmetry. These then demonstrate the existence of helical hinge modes. The calculations detailed in this figure were performed using the freely available Python package NESTED_AND_SPIN_RESOLVED_WILSON_LOOP [97], which represents an extension of the PythTB open-source Python tight-binding package [171] that was implemented and utilized for the preparation of SRefs. [8, 15] and the present work.

(momentum space) Bloch Hamiltonian is given by [31]

$$[H(\mathbf{k})] = \left(m_1 + \sum_{i=x,y,z} v_i \cos(k_i) \right) \tau_z \mu_0 \sigma_0 + m_2 \tau_z \mu_x \sigma_0 + m_3 \tau_z \mu_z \sigma_0 + u_x \sin(k_x) \tau_y \mu_y \sigma_0 + u_z \sin(k_z) \tau_x \mu_0 \sigma_0$$
$$+ m_{v_1} \tau_0 \mu_z \sigma_0 + m_{v_2} \tau_0 \mu_x \sigma_0 + v_H \sin(k_y) \tau_y \mu_z \sigma_z + A_{\text{spin-mixing}} \sin(k_z) \tau_y \mu_0 \sigma_x + f_{323} \tau_z \mu_y \sigma_z. \quad (4.125)$$

The Bloch Hamiltonian in SEq. (4.125) has both $\mathcal{I}$ and $\mathcal{T}$ symmetries such that

$$[\mathcal{I}][H(\mathbf{k})][\mathcal{I}]^{-1} = (\tau_z\mu_0\sigma_0)[H(\mathbf{k})](\tau_z\mu_0\sigma_0) = [H(-\mathbf{k})], \quad (4.126)$$
$$[\mathcal{T}][H(\mathbf{k})][\mathcal{T}]^{-1} = (\tau_z\mu_0\sigma_y)[H(\mathbf{k})]^*(\tau_z\mu_0\sigma_y) = [H(-\mathbf{k})], \quad (4.127)$$

respectively. We will be using the following set of parameters:

$$m_1 = -3.0, v_x = v_z = u_x = u_z = 1.0, v_y = 2.0, m_2 = 0.3, m_3 = 0.2, m_{v_1} = -0.4, m_{v_2} = 0.2, v_H = 1.2, f_{323} = 0.25. \quad (4.128)$$

When $A_{\text{spin}-\text{mixing}} \neq 0$ the Hamiltonian [SEq. (4.125)] does not conserve s_z. In this section we take

$$A_{\text{spin}-\text{mixing}} = 0.5. \quad (4.129)$$

Although s_z is not conserved, the gap in the $P(\mathbf{k})s_zP(\mathbf{k})$ spectrum remains open throughout the 3D BZ.

The 3D band structure [shown in SFig. 23(a)] consists of four doubly degenerate bands due to the coexistence of $\mathcal{I}$ and spinful $\mathcal{T}$ symmetries. The 2D surfaces of this helical HOTI are fully gapped [SFig. 23(b)]. In particular, the higher-order topology manifests in the existence of an odd number of gapless 1D hinge states in highly symmetric *finite-sized* model geometries (for any choice of Miller index, since the wallpaper group symmetries $p11'$ for any smooth surface termination cannot stabilize degeneracies between surface bands at generic momenta [18, 43, 44, 107]) that preserves $\mathcal{I}$ and spinful $\mathcal{T}$ symmetries. The gapless modes in SFig. 23(c) are helical hinge states [SFig. 23(i)] that are related to each other by $\mathcal{I}$ at $\mathcal{I}$-related hinges, and spinful $\mathcal{T}$ symmetry at the same hinges.

Employing the formalism developed in SN 3 A and 3 B, we next compute the k_x-directed P- and $P_\pm$-Wilson loop eigenphases for a highly-symmetric 2D slab of this helical HOTI infinite along x and y while finite along z at half-filling. Since the system has both $\mathcal{I}$ and spinful $\mathcal{T}$ symmetries, the set of PsP eigenvalues $\{(s_z)_n(\mathbf{k})\}$ with $n = 1, \ldots, N_{\text{occ}}$ at momentum $\mathbf{k}$ will be the same as the set $\{-(s_z)_n(\mathbf{k})\}$, as is discussed in SN 2 B. The spin gap in our model is open throughout the 3D BZ, such that we can define the $[P_\pm(\mathbf{k})]$ matrix projectors onto the positive and negative Ps_zP eigenspaces. In particular, we have $\text{rank}([P_+(\mathbf{k})]) = \text{rank}([P_-(\mathbf{k})]) = N_{\text{occ}}/2$ for all $\mathbf{k}$. As shown in SFig. 23(d), the k_x-directed P-Wilson loop eigenphases for the occupied energy bands of the 2D slab with half-filling exhibit a helical winding when $k_y \to k_y + 2\pi$. In addition, its k_x-directed $P_\pm$-Wilson loop eigenphases have winding numbers equal to ∓ 1 when $k_y \to k_y + 2\pi$ as shown in SFig. 23(e–h). According to the sign convention in SEq. (3.24) relating the winding number of the Wilson loop spectrum and the Chern number, we deduce that the partial Chern numbers of this 2D slab are $C^\pm_{\gamma_1} = \mp 1$. This confirms that the 2D slab of a helical HOTI is a 2D $\mathcal{T}$-invariant topological insulator with nontrivial $\mathbb{Z}_2$ invariant $\nu_{2d} = 1$ [1, 114].

Next, anticipating the possibility of fragile Wilson loop winding at TRIM planes in the 3D BZ (see SRefs. [8, 15]) with $\mathcal{I}$ and spinful $\mathcal{T}$ symmetries, we couple our helical HOTI model to eight additional trivial bands which also respect $\mathcal{I}$ and spinful $\mathcal{T}$ symmetries. We place two spinful s-like orbitals at a generic position $\mathbf{r}_3 = (r_{3,x}, r_{3,y}, r_{3,z}) = (0.35, 0.15, 0.31)$ in the unit cell (in reduced coordinates) and another two spinful s-like orbitals at $-\mathbf{r}_3$. We will use $d^\dagger_{\mathbf{R},\mu,\sigma}$ $[f^\dagger_{\mathbf{R},\mu,\sigma}]$ to denote the second-quantized creation operators for the μth ($\mu = 1, 2$) spin σ ($\sigma =\uparrow,\downarrow$) s-like orbital at $\mathbf{r}_3$ $[-\mathbf{r}_3]$ in unit cell $\mathbf{R}$. There are in total *eight* additional tight-binding basis orbitals. In order to induce spinful p-like orbitals at the $1a$ Wyckoff position, we couple these eight additional degrees of freedom through the following Hamiltonian:

$$H_{sp} = \sum_{\mathbf{R},\mu,\sigma}\left[t_a\left(d^\dagger_{\mathbf{R},\mu,\sigma}f_{\mathbf{R},\mu,\sigma} + \text{H.c.}\right)\right] + \sum_{\mathbf{R},\mu,\sigma}\left[t_b\left(d^\dagger_{\mathbf{R},\mu,\sigma}d_{\mathbf{R},\mu,\sigma} + f^\dagger_{\mathbf{R},\mu,\sigma}f_{\mathbf{R},\mu,\sigma}\right)\right]$$
$$+ \sum_{\mathbf{R},\sigma}\left[t_c\left(d^\dagger_{\mathbf{R},1,\sigma}d_{\mathbf{R},1,\sigma} - d^\dagger_{\mathbf{R},2,\sigma}d_{\mathbf{R},2,\sigma} + f^\dagger_{\mathbf{R},1,\sigma}f_{\mathbf{R},1,\sigma} - f^\dagger_{\mathbf{R},2,\sigma}f_{\mathbf{R},2,\sigma}\right)\right], \quad (4.130)$$

where t_a, t_b and t_c are all real numbers, and H.c. means the Hermitian conjugate. A nonzero value of t_a will hybridize the single-particle states $|d_{\mathbf{R},\mu,\sigma}\rangle = d^\dagger_{\mathbf{R},\mu,\sigma}|0\rangle$ and $|f_{\mathbf{R},\mu,\sigma}\rangle = f^\dagger_{\mathbf{R},\mu,\sigma}|0\rangle$. Next, we couple these additional orbitals to the original helical HOTI model [SEq. (4.125)]. The creation [annihilation] operators of the spinful s and ip orbitals in the original helical HOTI model will be denoted as $c^\dagger_{\mathbf{R},s,\mu,\sigma}$ $[c_{\mathbf{R},s,\mu,\sigma}]$ and $c^\dagger_{\mathbf{R},ip,\mu,\sigma}$ $[c_{\mathbf{R},ip,\mu,\sigma}]$. $c^\dagger_{\mathbf{R},s,\mu,\sigma}$ $[c^\dagger_{\mathbf{R},ip,\mu,\sigma}]$ creates an electron in the μth ($\mu = 1, 2$) s $[ip]$ orbital with spin σ ($\sigma =\uparrow,\downarrow$) at the origin of the unit cell $\mathbf{R}$. The Hamiltonian that couples the additional eight tight-binding basis states created by $d^\dagger_{\mathbf{R},\mu,\sigma}$ and $f^\dagger_{\mathbf{R},\mu,\sigma}$ to the original helical HOTI model [SEq. (4.125)] is given by

$$H_C = \sum_{\mathbf{R},\sigma}\sum_{\mu,\nu=1}^{2}\left[t_d\left(c^\dagger_{\mathbf{R},s,\mu,\sigma}\left\{d_{\mathbf{R},\nu,\sigma} + f_{\mathbf{R},\nu,\sigma}\right\} + \text{H.c.}\right)\right]. \quad (4.131)$$

Both H_{sp} in SEq. (4.130) and H_C in SEq. (4.131) are $\mathcal{I}$- and $\mathcal{T}$-symmetric. We take

$$t_a = 11.0,\ \ t_b = -0.5,\ \ t_c = 1.5, t_d = 1.0. \tag{4.132}$$

The 3D bulk band structure of the resulting sixteen-band model has eight pairs of doubly-degenerate bands and is shown in SFig. 24(a) where the middle four doubly degenerate bands (eight bands in total) are from the original helical HOTI model [SEq. (4.125)], as indicated in the figure. Our choice $|t_a| \gg |t_b|, |t_c|, |t_d|$ ensures that the lowest four bands in SFig. 24(a) are topologically trivial.

In order to explicitly see the fragile Wilson loop winding, we first compute the k_z-directed P- and $P_\pm$-Wilson loop eigenphases as a function of k_y for different constant-k_x planes for the four valence bands from the original helical HOTI model denoted by P_4 in SFig. 24(a). For our model with a 3D orthorhombic lattice, taking the P- and $P_\pm$-Wilson loops along the k_z direction ensures that our (spin-resolved) Wannier bands correspond to states localized in the $\hat{\mathbf{z}}$ direction, allowing us to make contact with the layer constructions of SN 4 D. Again, we construct the $[P_\pm(\mathbf{k})]$ matrix projectors such that they project to the positive and negative Ps_zP eigenspace, respectively. At constant-k_x planes, the P-Wannier bands [SFig. 24(b,e,h)] can be spin-resolved into P_+-Wannier bands [SFig. 24(c,f,i)] and P_--Wannier bands [SFig. 24(d,g,j)]. In particular, the number of P_+-Wannier bands is the same as the P_--Wannier bands, as $\text{rank}([P_+(\mathbf{k})]) = \text{rank}([P_-(\mathbf{k})])$. Notice that the P-Wannier bands are not a direct superposition of the corresponding $P_\pm$-Wannier bands, which would be the case in a system with s_z conservation. At the $k_x = 0$ plane, the P-Wannier bands [SFig. 24(b)] consist of two sets of helically winding bands. This is the pattern of Wilson loop winding that one would obtain from superposing two identical copies of a 3D spinful $\mathcal{T}$-invariant strong topological insulator (TIs) with $\mathcal{I}$ symmetry [17, 21, 33]. This doubled helical winding in the P-Wilson loop spectrum in SFig. 24(b), however, is fragile; below we will remove it by considering the projector P_6 indicated in SFig. 24(a).

Turning now to the spin-resolved topology, we can consider the $P_\pm$-Wilson loops constructed from the projector P_4. At the $k_x = 0$ plane, each of the $P_\pm$-Wannier bands [SFig. 24(c,d)] exhibits fragile winding (we will explicitly demonstrate the fragility shortly) that is protected by $\mathcal{I}$ symmetry, while at other constant-k_x planes there is no such fragile winding [SFig. 24(f,g,i,j)]. Notice that at the $k_x = 0$ and π planes, each of the positive and negative Ps_zP eigenspace has $\mathcal{I}$ symmetry while there is no spinful $\mathcal{T}$ symmetry—the spinful $\mathcal{T}$ operation maps between the positive and negative Ps_zP eigenspace. The $P_\pm$-Wannier band crossing in SFig. 24(c,d) is fragile, in the sense that they can be removed by adding additional trivial degrees of freedom to the occupied subspace. This implies that in 2D, there may also exist phases of matter with *fragile spin-resolved topology*. We leave the further exploration of this intriguing possibility for future work. By considering the bands in P_4 of SFig. 24(a) together with trivial bands induced from spinful p-orbitals at the $1a$ Wyckoff position, we may remove the fragile winding in both the P- and $P_\pm$-Wannier bands at the $k_x = 0$ planes.

To remove the fragile winding in SFig. 24(b–d) while—crucially—not changing the bulk stable (spin-resolved) topology, we add the doubly degenerate bands right below the image of P_4 to construct the projector P_6 onto the set of bands denoted in SFig. 24(a), which contains in total six bands. We then compute the k_z-directed P- and $P_\pm$-Wannier bands as a function of k_y at different constant-k_x planes for the six bands in the image of P_6. As shown in SFig. 25(a–c), there is no longer any winding of the P- and $P_\pm$-Wannier bands at the $k_x = 0$ plane. Moreover, for all constant-k_x constant planes, the P- and $P_\pm$-Wannier bands can be divided into two disjoint sets of *inner* and *outer* bands [SN 4 C 4] that are spectrally separated from each other by a *Wannier gap* of size $\approx 0.04\pi$, as indicated in SFig. 25(a–i). The inner and outer set of k_z-directed Wannier bands here represent hybrid Wannier states localized at the inversion-symmetric center ($z = 0$) and boundary ($z = 1/2$) of the unit cell respectively. Importantly, as discussed in SN 4 C 4, the inner and outer sets of Wannier bands both obey SEq. (4.83) due to bulk $\mathcal{I}$ symmetry. We refer the readers to SN 6 A and 6 E for the rigorous proof of the inversion symmetry constraint on the P- and $P_\pm$-Wannier bands. Together with the fact that the sixteen-band helical HOTI is $\mathcal{I}$- and $\mathcal{T}$-symmetric with gapped energy and Ps_zP spectrum, We can next apply the formalism of SN 4 A and 4 B to compute the nested P- and nested $P_\pm$-Wilson loops for the occupied bands in the image of the projector P_6.

Before we move on to compute the nested P- and $P_\pm$-Wilson loop eigenphases of our helical HOTI model, we here propose that a 3D helical HOTI can be viewed as a pump of a 2D $\mathcal{I}$- and $\mathcal{T}$-symmetric fragile (or obstructed atomic) phase, following the results of SRef. [15]. We emphasize first that since we use a k_z-directed Wilson loop to define our Wannier bands (to make contact with the layer constructions of SN 4 D), the nested Wilson loop spectra varies as a function of k_x. Note first that the fragile winding of the P-Wannier bands at the $k_x = 0$ plane can be removed by adding trivial degrees of freedom that respect $\mathcal{I}$ and $\mathcal{T}$ symmetries. In the sixteen-band helical HOTI model the trivial bands are induced from spinful p orbitals at the $1a$ Wyckoff position. Next, since there is a spin gap, we can decompose the occupied space into two parts—the positive and negative Ps_zP eigenspaces related to each other by $\mathcal{T}$. The fragile winding of the $P_\pm$-Wannier bands at the $k_x = 0$ plane can be removed by adding trivial degrees of freedom that respect $\mathcal{I}$ symmetry within each of the $P_\pm$-eigenspace. In our case such trivial bands within each of the $P_\pm$-eigenspaces are induced from spinless p orbitals at the $1a$ Wyckoff position. If we view k_x as a pumping parameter, we can say that a helical HOTI demonstrates a pump from a 2D fragile topological insulator with both $\mathcal{I}$

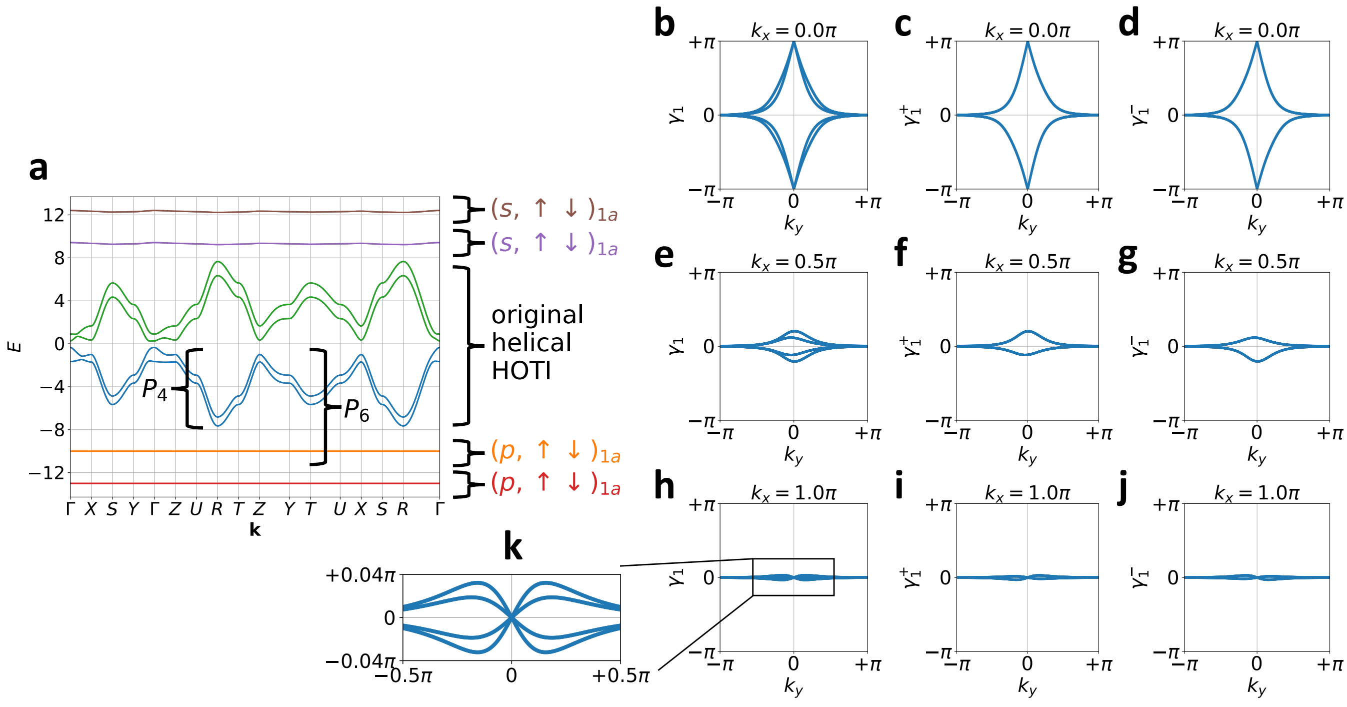

Supplementary Figure 24. P- and $P_\pm$-Wilson loops for four bands in the sixteen-band helical HOTI model. The model is obtained by coupling the eight-band helical HOTI model introduced in SRef. [31] [and described in SEqs. (4.125), (4.128), and (4.129)] to eight additional trivial bands, as described in SEqs. (4.130), (4.131) and (4.132). (a) shows the 3D bulk band structure where we have labeled the elementary band representation $(\rho, \uparrow\downarrow)_{\mathbf{q}}$ of the additional trivial bands induced from spinful ρ orbitals at Wyckoff position $\mathbf{q}$ [123, 224]. The middle four doubly-degenerate bands (eight bands in total) are also indicated as originating from the "original" eight band helical HOTI in SFig. 23. P_4 denotes the projector onto the four valence bands of the original helical HOTI and P_6 is the projector constructed from the direct sum of P_4 and the projector onto the two additional trivial bands induced from spinful p orbitals at the $1a$ Wyckoff position [whose bands are shown in orange in (a)]. (b), (c) and (d) show the k_z-directed P-, P_+- and P_--Wannier bands as a function of k_y for P_4 in the $k_x = 0$ plane. For our helical HOTI model with a 3D orthorhombic lattice, "k_z-directed" refers to our choice of $\mathbf{G} = 2\pi\hat{\mathbf{z}}$ in the P- and $P_\pm$-Wilson loop matrices in SEqs. (3.2) and (3.17). In (b) we see that the P-Wilson loop eigenphases evaluated with the projector P_4 have an even helical winding. On the other hand, in (c) and (d) we see that the $P_\pm$-Wilson loop eigenphases constructed from the projector P_4 have an odd helical winding. As explained in the text, such windings can be removed and are fragile, as demonstrated in SFig. 25. (e), (f) and (g) are the k_z-directed P-, P_+- and P_--Wannier bands as a function of k_y for P_4 in the $k_x = 0.5\pi$ plane. (h), (i) and (j) are the k_z-directed P-, P_+- and P_--Wannier bands as a function of k_y for P_4 in the $k_x = \pi$ plane. For (e)–(j), there is no spectral flow as opposed to (b)–(d). (k) is an enlarged plot of (h) showing that there are in total four Wannier bands in (h). The numbers of bands for the P- [panels (b), (e), and (h)], P_+- [panels (c), (f), and (i)], and P_- -Wannier bands [panels (d), (g), and (j)] are 4, 2 and 2 respectively. The calculations detailed in this figure were performed using the freely available Python package NESTED_AND_SPIN_RESOLVED_WILSON_LOOP [97], which represents an extension of the PythTB open-source Python tight-binding package [171] that was implemented and utilized for the preparation of SRefs. [8, 15] and the present work.

and $\mathcal{T}$ symmetries and (filling-) anomalous spin-charge-separated corner modes [15, 31, 174] at the $k_x = 0$ plane to a 2D trivial insulator at the $k_x = \pi$ plane. This is the spinful time-reversal-symmetric generalization of the concept that a magnetic AXI can be viewed as a pump from a 2D fragile topological insulator to a 2D trivial insulator with $\mathcal{I}$ symmetry [15]. In other words, from this pumping perspective, a helical HOTI can be viewed as superposing (*i.e.*, "stacking" in the language of SRef. [17]) two orbital (effectively spinless or spin-polarized) magnetic AXIs related to each other by spinful $\mathcal{T}$ symmetry. This picture is the momentum-space analogue of the position-space, spin-resolved layer construction of a helical HOTI in the T-DAXI regime introduced in SN 4 D.

We next compute the nested P- and nested $P_\pm$-Wilson loop eigenphases for our sixteen-band HOTI model [SEqs. (4.125), (4.130), and (4.131)]. After presenting the numerical results, we will then discuss how the coexistence of both $\mathcal{I}$ and spinful $\mathcal{T}$ symmetries can constrain the various nested (partial) Berry phases. For the P-Wannier bands in SFig. 25(a,d,g), the disjoint inner and outer set of bands contain two and four bands, respectively. For the $P_\pm$-Wannier bands in SFig. 25(b,c,e,f,h,i), the disjoint inner and outer set of bands contain one and two bands, respectively. We then construct the projectors onto the disjoint inner and outer set of P- [$P_\pm$-] Wannier bands,

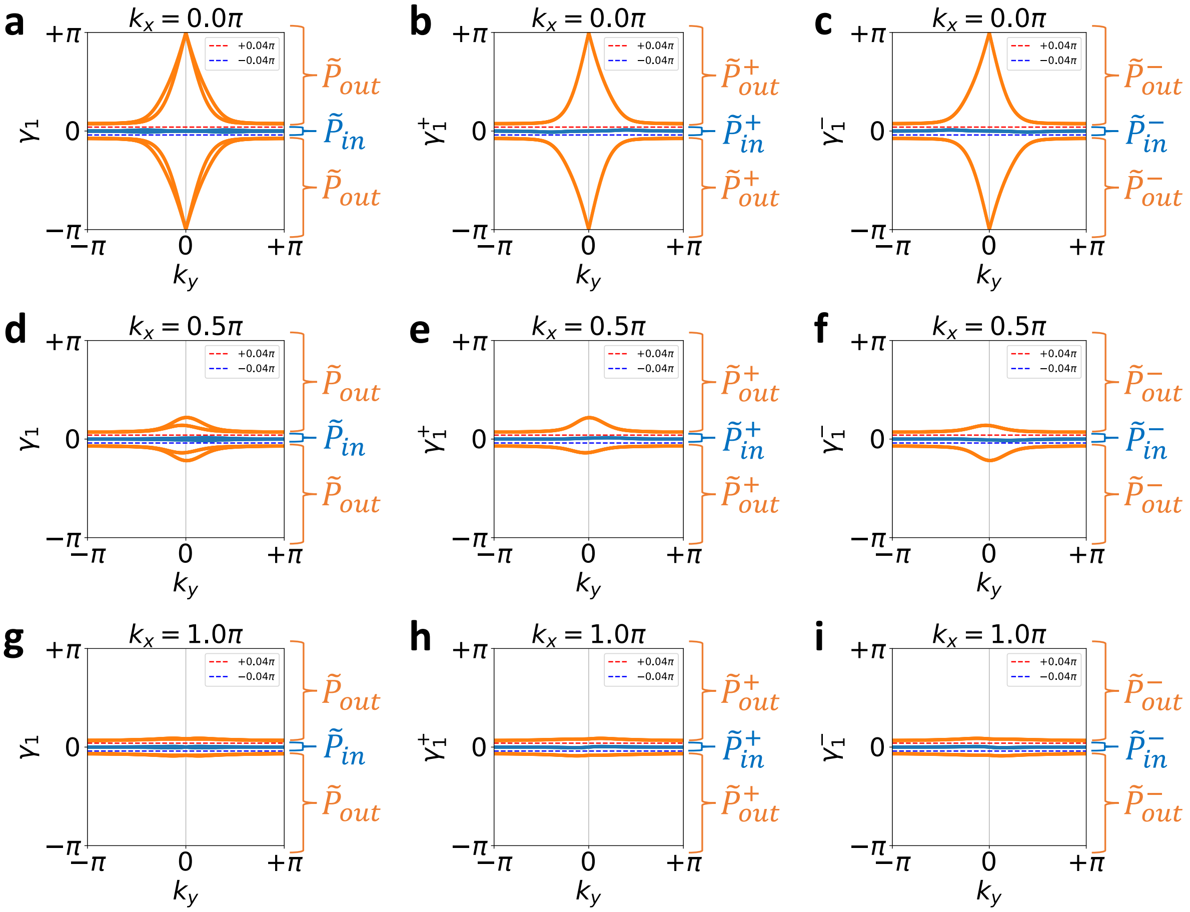

Supplementary Figure 25. P- and $P_\pm$-Wilson loops with $\mathbf{G} = 2\pi\hat{\mathbf{z}}$ [SEqs. (3.2) and (3.17)] of the sixteen-band helical HOTI model whose energy band structure is shown in SFig. 24(a). The model is obtained by coupling the eight-band helical HOTI model [SEqs. (4.125), (4.128), and (4.129)] to eight additional trivial bands described by SEqs. (4.130), (4.131) and (4.132). The occupied energy band projector considered here is P_6 in SFig. 24(a), which projects onto the valence bands of the original eight-band model of a helical HOTI together with two additional trivial bands induced from spinful p orbitals at the $1a$ Wyckoff position. (a), (b) and (c) are the k_z-directed P-, P_+- and P_--Wannier bands as functions of k_y for P_6 in the $k_x = 0$ plane. (d), (e) and (f) are the k_z-directed P-, P_+- and P_--Wannier bands as functions of k_y for P_6 in the $k_x = 0.5\pi$ plane. (g), (h) and (i) are the k_z-directed P-, P_+- and P_--Wannier bands as functions of k_y for P_6 in the $k_x = \pi$ plane. We can see that the spectral flow in SFig. 24(b–d) has been trivialized in panels (a)–(c). In particular, for each panel (a)–(i) of this figure, the P- and $P_\pm$-Wannier bands can be separated into inner and outer sets separated by a Wannier band gap size of at least $\approx 0.04\pi$. For each panel, we have also labeled the projectors onto the inner and outer set of Wannier bands by $\widetilde{P}_{in/out}$ and $\widetilde{P}^\pm_{in/out}$ explicitly, following the notation of SRef. [15]. We can view the inner and outer sets of Wannier bands here as representing hybrid Wannier states localized around the inversion-symmetric center ($z = 0$) and boundary ($z = 1/2$) of the unit cell [SN 4 C 4]. The numbers of bands for the P- [panels (a), (d), and (g)], P_+- [panels (b), (e), and (h)], and P_--Wannier bands [panels (c), (f), and (i)] in here are 6, 3 and 3, respectively. The calculations detailed in this figure were performed using the freely available Python package NESTED_AND_SPIN_RESOLVED_WILSON_LOOP [97], which represents an extension of the PythTB open-source Python tight-binding package [171] that was implemented and utilized for the preparation of SRefs. [8, 15] and the present work.

which we label as $\widetilde{P}_{in}$ [$\widetilde{P}^\pm_{in}$] and $\widetilde{P}_{out}$ [$\widetilde{P}^\pm_{out}$], respectively, as shown in SFig. 25(a–i). As the decomposition of the occupied band and positive/negative spin band projectors into $\widetilde{P}_{in/out}$ and $\widetilde{P}^\pm_{in/out}$ are well-defined and smooth over the full Brillouin zone, we can compute the corresponding *nested* P- and $P_\pm$-Wilson loop eigenphases. Following the formalism introduced in this work in SN 4 A and 4 B, we compute the nested P- and $P_\pm$-Wilson loops by choosing the primitive reciprocal lattice vectors $\mathbf{G} = 2\pi\hat{\mathbf{z}}$ and $\mathbf{G}' = 2\pi\hat{\mathbf{y}}$ [SEqs. (4.24) and (4.47)] as the first and second closed-loop holonomy, and the resulting eigenphases, which we also term nested Berry phases generally in this work, are plotted as functions of k_x. In SFig. 26(a,d), the nested P-Wilson loop eigenphases for $\widetilde{P}_{in}$ and $\widetilde{P}_{out}$ both have an odd *helical* winding. And as shown in SFig. 26(g), accidental crossing points in the nested P-Wilson loop eigenphases at generic momentum $k_x \neq 0$ and $k_x \neq \pi$ are not protected by symmetries or local topological invariants (like those

protecting Weyl points [173]), and are hence generically gapped. SFig. 26(a,d,g) therefore indicate that the 2D hybrid Wannier states localized at the $\mathcal{I}$-symmetric center ($z = 0$) and boundary ($z = 1/2$) planes of the unit cell both carry the same stable topology, and are specifically both topologically equivalent to $\mathcal{T}$-invariant 2D strong TIs. This can be compared with magnetic AXIs, whose spectral flow in the nested P-Wilson loop eigenphases has a nonzero odd chiral winding that is protected by bulk crystal symmetries, such as $\mathcal{I}$ [15]. By analogy, we hence establish that odd, helical winding in the nested P-Wilson loop spectrum is one bulk signature of a helical HOTI. As established in Supplementary Table 3, this is consistent with the recognition that a helical HOTI can be layer-constructed as an $\mathcal{I}$-symmetric stack of $\mathcal{T}$-invariant 2D strong TIs, with two parallel TI layers per unit cell separated by a half-lattice translation and pinned to $\mathcal{I}$-invariant real-space planes [33, 43, 44].

Due to the presence of a spin gap and a $P_{\pm}$-Wannier gap (see SFig. 25), we can further examine the spin-resolved topology of the sixteen-band helical HOTI model [SEqs. (4.125), (4.130), and (4.131)] by computing the nested $P_{\pm}$-Wilson loop eigenphases. Let us denote the nested partial Chern numbers computed from $\widetilde{P}^{\pm}_{in}$ and $\widetilde{P}^{\pm}_{out}$ as $C^{\pm}_{\gamma_2,in}$ and $C^{\pm}_{\gamma_2,out}$, respectively. We will here employ the sign convention used in SEq. (3.24) to relate the winding number of the (nested) Wilson loop spectrum to the Chern number (which relies on fixing a right-handed coordinate system) to determine the signs of $C^{\pm}_{\gamma_2,in}$ and $C^{\pm}_{\gamma_2,out}$ consistently. In this convention, $C^{\pm}_{\gamma_2,in}$ and $C^{\pm}_{\gamma_2,out}$ are also equal to the partial Chern numbers of the spin-resolved hybrid Wannier states localized at the center and the boundary of the unit cell. As discussed in SN 4 C 4, $C^{\pm}_{\gamma_2,in}$ and $C^{\pm}_{\gamma_2,out}$ are only $\mathbb{Z}_2$-stable after accounting for Wannier gap closures without accompanying closures of the spin gap or the energy gap. From SFig. 26(b,c,e,f,h,i), we observe that for the sixteen-band helical HOTI model, the nested partial Chern numbers are

$$C^{\pm}_{\gamma_2,in} = \pm 1, \tag{4.133}$$

$$C^{\pm}_{\gamma_2,out} = \mp 1. \tag{4.134}$$

We emphasize that $C^{\pm}_{\gamma_2,in}$ and $C^{\pm}_{\gamma_2,out}$ in SEq. (4.134) are the *negatives* of the winding numbers of the corresponding nested spin-resolved Wilson loop eigenphases in SFig. 26(b,c,e,f,h,i), owing to the sign convention in SEq. (3.24), in which the Chern number is equal to the negative [positive] winding number of the k_y-directed [k_x-directed] Wilson loop eigenphases as $k_x \to k_x + 2\pi$ [$k_y \to k_y + 2\pi$]. SEqs. (4.133) and (4.134) are consistent with the spin-stable topology of a T-DAXI characterized by the $\mathbb{Z}_2 \times \mathbb{Z}$ spin-stable topological indices

$$(C^{+}_{\gamma_2,in} \mod 2, \nu^{+}_{z}) = (1 \mod 2, 0) = (1, 0) \tag{4.135}$$

using the classification previously introduced in SN 4 D, in which ν^{+}_{z} is the partial weak Chern number in the xy plane, and where we have specialized to cases in which the other, unspecified $\mathbb{Z}$-valued partial weak Chern numbers vanish $\nu^{\pm}_{x} = \nu^{\pm}_{y} = 0$ (see also Supplementary Table 6).

Next, we recall that as established in SRef. [15], the nontrivial axion angle $\theta = \pi$ of a magnetic AXI can be inferred from the presence of an odd winding number in the nested P-Wilson loop eigenphases, provided that the weak Chern numbers ν_i all vanish (and that the bulk contains magnetic crystal symmetries that quantize θ, namely those that change the sign of $\mathbf{E} \cdot \mathbf{B}$). From this perspective, SFig. 26(b,c,e,f,h,i), together with SEqs. (4.133) and (4.134), therefore demonstrate that the positive and negative Ps_zP eigenspaces can each be viewed as orbital (effectively spinless or spin-polarized) AXIs. Hence our helical HOTI model lies in the T-DAXI regime. From the odd winding numbers $C^{\pm}_{\gamma_2,in/out}$ [SEqs. (4.133) and (4.134)], we specifically deduce that the partial axion angles [SEq. (4.123)] for the sixteen-band helical HOTI model are

$$\theta^{\pm} = \pi. \tag{4.136}$$

We further note that the winding numbers $C^{\pm}_{\gamma_2,in}$ and $C^{\pm}_{\gamma_2,out}$ are opposite to each other since $\nu^{\pm}_{z} = 0$ [SEq. (4.65)] in our helical HOTI model, which most clearly differentiates it from a spin-stable 3D QSHI as discussed in SN 4 D (see specifically Supplementary Table 6). For completeness, it is also useful to note that $\mathcal{T}$ symmetry enforces that the winding numbers $C^{+}_{\gamma_2,in}$ [$C^{+}_{\gamma_2,out}$] and $C^{-}_{\gamma_2,in}$ [$C^{-}_{\gamma_2,out}$] carry opposite signs [SN 4 C 4 and 6 H].

As discussed in SN 4 D, since our helical HOTI model is a spin-stable T-DAXI with an $s_z = \hat{\mathbf{z}} \cdot \mathbf{s}$ spin gap, we have that the bulk topological contribution to its s_z spin Hall conductivity in the xy-plane per unit cell along z vanishes $(\sigma^{s}_{\mathrm{T-DAXI}})_{\mathrm{topological}} = 0$ [SEq. (4.122)]. However, we importantly find that it instead carries a bulk topological contribution to the partial magnetoelectric polarizability $\alpha^{\pm} = \pi \cdot \frac{e^2}{2\pi h}$ [SEq. (4.124)].

To summarize, we have explicitly demonstrated the following bulk signatures of a symmetry-indicated, spin-gapped helical HOTI lying in the T-DAXI regime:

1. Independent of its spin resolution, a helical HOTI is characterized by an odd helical winding in the nested P-Wilson loop eigenphases as demonstrated in SFig. 26(a,d), whenever there is a Wannier gap as demonstrated in SFig. 25(a,d,g).

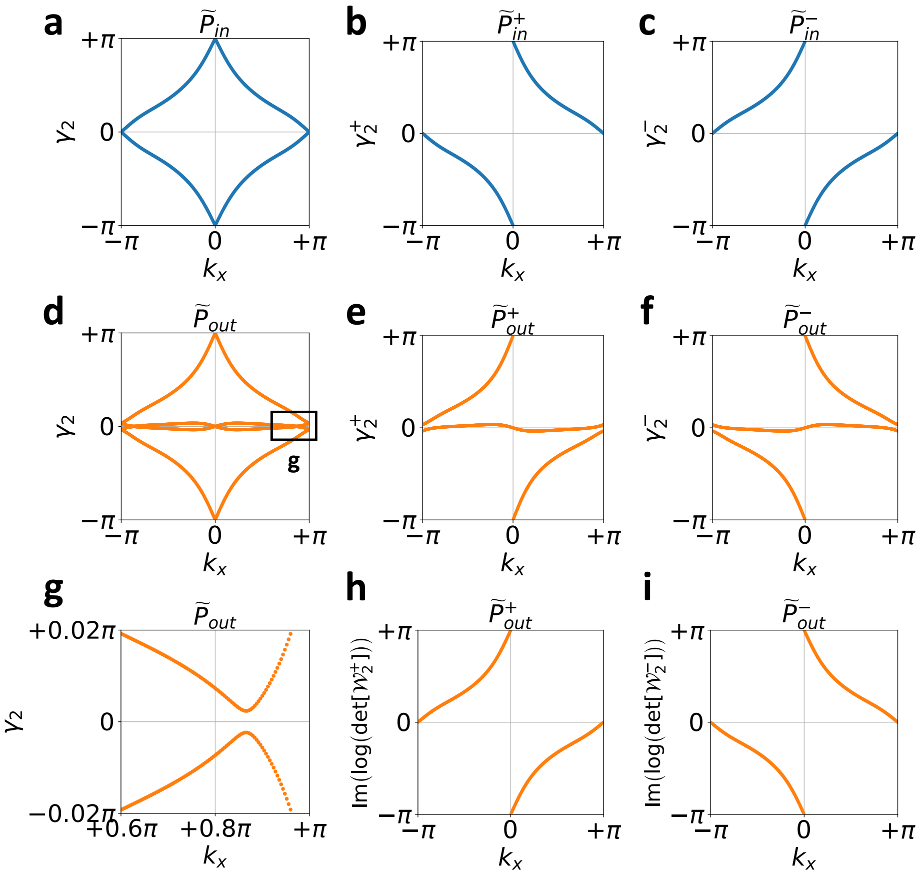

Supplementary Figure 26. Nested P- and $P_\pm$-Wilson loops with $\mathbf{G} = 2\pi\hat{\mathbf{z}}$ and $\mathbf{G}' = 2\pi\hat{\mathbf{y}}$ [SEqs. (4.24) and (4.47)] as the first and second closed-loop holonomy of the sixteen-band helical HOTI model whose energy band structure is shown in SFig. 24(a). The model is obtained by coupling the eight-band helical HOTI model [SEqs. (4.125), (4.128), and (4.129)] to eight additional trivial bands described by SEqs. (4.130), (4.131) and (4.132). We use the projector P_6 indicated in SFig. 24(a) to construct the nested Wilson loops. (a), (b) and (c) are the nested P-, P_+- and P_--Wilson loop eigenphases of the inner Wannier bands denoted by $\widetilde{P}_{in}$, $\widetilde{P}^{+}_{in}$ and $\widetilde{P}^{-}_{in}$ in SFig. 25, respectively. We observe an odd helical winding in the spectrum of the nested P-Wilson loop shown in (a). In the nested spin-resolved Wilson loop spectra shown in (b) and (c), we see a corresponding odd chiral winding with winding number -1 in the nested P_+-Wilson loop spectrum and winding number $+1$ in the nested P_--Wilson loop spectrum. (d), (e) and (f) are the nested P-, P_+- and P_--Wilson loop eigenphases of the outer Wannier bands denoted by $\widetilde{P}_{out}$, $\widetilde{P}^{+}_{out}$ and $\widetilde{P}^{-}_{out}$ in SFig. 25, respectively. (g) is an enlarged view of (d) which demonstrates that the band crossings at generic momentum $k_x \neq 0$ and $k_x \neq \pi$ are not protected and are gapped. There is also a helical winding in (d), similar to that shown in panel (a). Correspondingly, the nested spin-resolved Wilson loop spectra for the outer spin-resolved Wannier bands shown in (e) and (f) have odd chiral winding. (h) and (i) are the total nested partial Berry phases (each of (e) and (f) contains two bands) of the outer $P_\pm$-Wannier bands denoted by $\widetilde{P}^{+}_{out}$ and $\widetilde{P}^{-}_{out}$ in SFig. 25, respectively. From (h) and (i), we see that there is spectral flow with winding numbers $+1$ and -1 in (e) and (f), respectively. Together with (b) and (c) which carry opposite winding numbers of (h) and (i) respectively, this indicates that the helical HOTI in SFig. 24 lies in the T-DAXI regime and is hence characterized by the nontrivial *partial* axion angles $\theta^\pm = \pi$ [see SEq. (4.136) and the surrounding text]. The number of nested (spin-resolved) Wannier bands in (a), (b), and (c) is 2, 1, and 1, respectively, and the number of nested (spin-resolved) Wannier bands in (d), (e), and (f) is 4, 2, and 2, respectively. The calculations detailed in this figure were performed using the freely available Python package NESTED_AND_SPIN_RESOLVED_WILSON_LOOP [97], which represents an extension of the PythTB open-source Python tight-binding package [171] that was implemented and utilized for the preparation of SRefs. [8, 15] and the present work.

2. Independent of its spin-resolution, a helical HOTI can be viewed as a momentum-space pump from an $\mathcal{I}$- and spinful $\mathcal{T}$-symmetric 2D fragile topological insulator (or obstructed atomic insulator) to a 2D trivial insulator, building on the description of $\mathcal{I}$-symmetric magnetic AXIs established in SRef. [15].

3. A helical HOTI in the T-DAXI regime with vanishing partial weak Chern numbers $\nu_x^\pm = \nu_y^\pm = 0$ has a $\mathbb{Z}_2 \times \mathbb{Z}$ invariant $(C^{+}_{\gamma_2,in} \mod 2, \nu_i^+) = (1 \mod 2, 0) = (1, 0)$, which is indicated by odd winding numbers in the nested

Berry phases in the $P_{\pm}$-eigenspace, as demonstrated in SFig. 26(b,c,e,f,h,i).

4. A helical HOTI in the T-DAXI regime is characterized by the quantized nontrivial partial axion angles $\theta^{\pm} = \pi$, which give rise to a bulk topological contribution to the partial magnetoelectric polarizability, and represent a 3D generalization of the partial polarization introduced by Fu and Kane in SRef. [2].

5. POSITION-SPACE SIGNATURES OF SPIN-RESOLVED TOPOLOGY

In this section, we extend the formalism and numerical methods for computing layer-resolved Chern numbers [36, 77, 105] in position space to compute layer-resolved partial Chern numbers. This will allow us to quantitatively search for anomalies on the gapped 2D surfaces of 3D helical HOTIs in the T-DAXI regime, and to explore the implications of the partial axion angle introduced in SN 4 D.

To begin, in this section, we will consider d-dimensional translation-invariant systems with primitive Bravais lattice vectors $\{\mathbf{a}_1 \ldots \mathbf{a}_d\}$, where $d = 2$ or 3. Each lattice point $\mathbf{R}$ (*i.e.* the center of each unit cell indexed by $\mathbf{R}$), can be written as $\mathbf{R} = \sum_{i=1}^{d} n_i \mathbf{a}_i$ where $n_i \in \mathbb{Z}$ $(i = 1 \ldots d)$. Given $\{\mathbf{a}_1 \ldots \mathbf{a}_d\}$, we can construct a set of dual primitive reciprocal lattice vector $\{\mathbf{G}_1 \ldots \mathbf{G}_d\}$ satisfying $\mathbf{a}_i \cdot \mathbf{G}_j = 2\pi\delta_{ij}$ $(i = 1 \ldots d)$. We denote the number of (spinful) tight-binding basis orbitals within a primitive unit cell as N_{sta}. From a 3D translationally-invariant system we can form a 2D slab infinite along $\mathbf{a}_j$ and $\mathbf{a}_l$ while finite along $\mathbf{a}_i$ with N_i unit cells, where $\{\mathbf{a}_j, \mathbf{a}_l, \mathbf{a}_i\}$ is the linearly independent set of 3D real-space primitive lattice vectors. Throughout this section, we define a *layer* to consist of a 2D plane of the finite slab that is one *unit cell* thick in the finite direction of the slab [77, 105]. For example, if we use $N_i = 15$ *unit cells* of the original 3D translation-invariant system to form a slab that is finite along $\mathbf{a}_i$, we say this slab has 15 *layers* along $\mathbf{a}_i$. This is distinct from the usage of "layer" in the context of layer constructions (*e.g.* in SRefs. [33, 36, 42–44, 209] and SN 4 D), but is consistent with the terminology of SRefs. [77, 105]. For such a slab, we will see below that the layer-resolved partial Chern numbers tell us how the partial Chern numbers [SEq. (3.24)] of the 2D slab is distributed over the N_i unit cells, or equivalently over the N_i layers. We will argue that the layer-resolved partial Chern numbers are related to the response of a 2D slab to the insertion of a magnetic flux. For example, in SN 4 D we predicted that a $\Phi = \pi$ magnetic flux tube in a finite slab of a T-DAXI binds one spinon between the top and bottom surface [36, 92, 174, 220]. We will show that this is a consequence of the fact that in a T-DAXI the gapped surfaces have half-integer partial Chern numbers, while in the bulk the layer-resolved partial Chern numbers vanish when averaged over the degrees of freedom within each unit cell (layer). In this section we will first develop a formulation for the layer-resolved position-space marker [1, 36, 77, 98, 225] for the partial Chern number. We will then compute the layer-resolved partial Chern numbers for the model of a T-DAXI introduced in SN 4 E and numerically identify the *partial parity anomaly* (defined properly in SN 5 E) realized on its gapped surfaces.

In SN 5 A and 5 B, we will review the Chern marker [98, 99, 225] and partial Chern markers [1], which characterize for a 2D system how the Chern number and partial Chern numbers vary as functions of real-space position, respectively. As an extension of SN 5 A, in SN 5 C we will review the formalism for the layer-resolved Chern number, which describes how the Chern number of a 2D slab formed from a 3D translation-invariant system is distributed along the finite direction in position space. Based on SN 5 C, we then develop the formalism of layer-resolved partial Chern numbers in SN 5 D, which describes how the *partial* Chern numbers of a 2D slab formed from a 3D spinful translation-invariant system are distributed along the finite direction in position space. Finally, in SN 5 E we compute the layer-resolved partial Chern numbers of a T-DAXI, and show that a T-DAXI has vanishing partial Chern numbers in its bulk (when averaged over the degrees of freedom within each layer, or equivalently unit cell), and half-integer partial Chern numbers at its gapped surfaces—the hallmark of a *partial parity anomaly* arising from the bulk-quantized partial axion angle. We will also distinguish between the layer-resolved partial Chern numbers of a 3D quantum spin Hall insulator (QSHI) and a T-DAXI, which represent two physically distinct spin-stable topological regimes of spin-resolved helical HOTIs, as discussed in SN 4 D.

A. Chern Marker

In this section, we will review the construction of the position-space Chern marker $C(\mathbf{R})$, first introduced by Resta in SRef. [98]. This will allow us to develop intuition for topological markers in position space. We begin by considering a 2D tight-binding lattice Hamiltonian H with a finite size, and define the projector onto a set of occupied energy eigenstates as

$$P \equiv \sum_{n \in \text{occ}} |n\rangle \langle n| \,, \tag{5.1}$$

where $|n\rangle$ is the eigenstate of H satisfying $H|n\rangle = E_n|n\rangle$. The matrix representatives of the position operators $\widehat{x}$ and $\widehat{y}$ in the (spinful) tight-binding basis states have matrix elements

$$[\widehat{x}]_{\mathbf{R}\alpha,\mathbf{R}'\beta} = \langle \mathbf{R},\alpha|\widehat{x}|\mathbf{R}',\beta\rangle = \langle 0|c_{\mathbf{R},\alpha}\widehat{x}c^\dagger_{\mathbf{R}',\beta}|0\rangle = \delta_{\mathbf{R}\mathbf{R}'}\delta_{\alpha\beta}(\mathbf{R}+\mathbf{r}_\alpha)_x, \tag{5.2}$$

$$[\widehat{y}]_{\mathbf{R}\alpha,\mathbf{R}'\beta} = \langle \mathbf{R},\alpha|\widehat{y}|\mathbf{R}',\beta\rangle = \langle 0|c_{\mathbf{R},\alpha}\widehat{y}c^\dagger_{\mathbf{R}',\beta}|0\rangle = \delta_{\mathbf{R}\mathbf{R}'}\delta_{\alpha\beta}(\mathbf{R}+\mathbf{r}_\alpha)_y, \tag{5.3}$$

where $c^\dagger_{\mathbf{R},\alpha}$ and $c_{\mathbf{R},\alpha}$ represent creation and annihilation operators for the (spinful) orbital labeled by α in the unit cell $\mathbf{R}$, and $\mathbf{r}_\alpha$ is the position of the (spinful) orbital labeled by α within the unit cell $\mathbf{R}$. Using SEq. (5.1), we can then compute the position operators projected onto the occupied states as

$$\widehat{x}_P = P\widehat{x}P,\ \widehat{y}_P = P\widehat{y}P. \tag{5.4}$$

From SEq. (5.4) we can define the position-space-resolved Chern number termed the (local) Chern marker [98, 99, 225] in the unit cell $\mathbf{R}$ as

$$C(\mathbf{R}) = \frac{2\pi i}{A_{\text{cell}}}\sum_{\alpha=1}^{N_{\text{sta}}}\langle \mathbf{R},\alpha|\left[\widehat{x}_P,\widehat{y}_P\right]|\mathbf{R},\alpha\rangle\,, \tag{5.5}$$

where A_{cell} is the unit cell area of the 2D lattice, and N_{sta} is the number of tight-binding basis state within a unit cell of the 2D lattice. In SEq. (5.5), we have performed a trace of $[\widehat{x}_P,\widehat{y}_P]$ over the tight-binding basis states within the unit cell $\mathbf{R}$, and $|\mathbf{R},\alpha\rangle$ is the tight-binding basis state labeled by α in the unit cell $\mathbf{R}$. $C(\mathbf{R})$ represents the position-space resolution of the Chern number [98, 225] and physically corresponds to the local contribution to the Hall conductivity [105]. For a 2D gapped homogeneous system without disorder, the values of $C(\mathbf{R})$ for $\mathbf{R}$ within the bulk region of a finite-sized sample are equal to the Chern number of the system [98, 225]. In particular, in the thermodynamic limit, far from the (gapless) edges of a bulk-gapped system, $C(\mathbf{R})$ is a constant independent of $\mathbf{R}$ and equal to the Chern number. The local Chern marker can be used to study single-particle topology in the absence of translation invariance, such as for disordered systems, quasiperiodic systems and at interfaces between bulk topological phases [226–230]. In particular, formulated as a function of position, the Chern marker $C(\mathbf{R})$ allows one to compute how the Chern number varies as a function of position, either near the interface between two insulators with different Chern numbers [98], or in disordered systems [231]. By studying the scaling of the Chern marker as a function of the parameters inducing a band inversion, critical properties of (weakly-disordered) Chern insulators near topological quantum phase transition can be quantitatively computed [225, 232]. Furthermore, it is also possible to study the dynamics of the Chern marker $C(\mathbf{R},t)$ as a function of both position $\mathbf{R}$ and time t, from which a Chern marker current can be defined [225].

B. The Partial Chern Marker and the Spin Chern Marker

In this work, we are primarily focusing on $\mathcal{T}$-invariant insulators, in which the Chern number, and hence local Chern marker, vanish. However, this does not imply that $\mathcal{T}$-invariant insulators are featureless. Starting from the Chern marker formalism in SN 5 A, we will here formulate a partial Chern marker, which resolves partial Chern number [SEq. (3.24)] in position space. The partial Chern marker formalism that we will describe in this section builds off related constructions in SRefs. [1, 100, 101, 104, 233, 234]. Similar to SN 5 A, we begin by considering a 2D spinful tight-binding lattice Hamiltonian H with a finite size. We take the finite-sized system to have N unit cells with $N_{\text{sta}} = 2N_{\text{orb}}$ spinful orbitals in each unit cell, where the factor of 2 accounts for the spin-1/2 degree of freedom (where we recall from the discussion following SEq. (2.16) that the Hilbert space of a solid derives from pairs of orbitals at the same position with opposite spin, for systems both with and without $\mathcal{T}$-symmetry.) The spin operator along the direction $\hat{\mathbf{n}}$ in terms of the tight-binding basis states is defined in SEq. (2.13) as $s \equiv \hat{\mathbf{n}}\cdot\boldsymbol{\sigma}\otimes\mathbb{I}_{N_{\text{orb}}}\otimes\mathbb{I}_N$, where $\boldsymbol{\sigma}$ acts on the spin-1/2 degree of freedom, while $\mathbb{I}_{N_{\text{orb}}}$ and $\mathbb{I}_N$ are identity operators acting on the orbital and unit cell degrees of freedom, respectively. We can form the reduced spin operator in the occupied subspace as [see also SEq. (2.35)]

$$[s_{\text{reduced}}]_{m,n} = \langle m|\,s\,|n\rangle\,, \tag{5.6}$$

where $|n\rangle$ is the nth energy eigenstate of the Hamiltonian H, such that $H|n\rangle = E_n|n\rangle$, and $m,n = 1\ldots N_{\text{occ}}$ with N_{occ} representing the number of occupied energy eigenstates. Therefore, $[s_{\text{reduced}}]$ is an $N_{\text{occ}}\times N_{\text{occ}}$ matrix. The matrix elements of $[s_{\text{reduced}}]$ are the matrix elements of the projected spin operator PsP between occupied energy eigenstates, where P is given in SEq. (5.1). For a finite system with spinful $\mathcal{T}$ symmetry, recall from SN 2 B that the

eigenvalues λ_n of $[s_{\text{reduced}}]$ satisfy $\{\lambda_n|n = 1\ldots N_{\text{occ}}\} = \{-\lambda_n|n = 1\ldots N_{\text{occ}}\}$. We can then define the spin gap Δ_s for a spinful $\mathcal{T}$-invariant system with a finite size as twice the smallest magnitude among the $[s_{\text{reduced}}]$ eigenvalues such that $\Delta_s = 2|\lambda_{\text{min}}|$, where λ_{min} is the eigenvalue of $[s_{\text{reduced}}]$ with smallest absolute value. We remind the reader that here we are considering a finite-sized system, such that both the reduced spin matrix $[s_{\text{reduced}}]$ and the PsP eigenvalues λ_n are not functions of crystal momentum. In particular, when $\Delta_s > 0$, we say that the spin gap of this spinful $\mathcal{T}$-invariant finite-sized system is open.

When the spin gap is open, we can divide the occupied subspace into the positive and negative PsP eigenspace as follows. We first write the eigenvalue equation of $[s_{\text{reduced}}]$ as

$$[s_{\text{reduced}}] \left|\tilde{u}_n^{\pm}\right\rangle = \lambda_n^{\pm} \left|\tilde{u}_n^{\pm}\right\rangle, \tag{5.7}$$

where $\lambda_n^+ > 0$ and $\lambda_n^- < 0$, and $|\lambda_n^+| = |\lambda_n^-|$ due to $\mathcal{T}$ symmetry. We may re-express the N_{occ}-component eigenvectors $|\tilde{u}_n^{\pm}\rangle$ of $[s_{\text{reduced}}]$ in the spinful tight-binding orbital basis states using

$$\left|u_n^{\pm}\right\rangle = \sum_{m=1}^{N_{\text{occ}}} [\tilde{u}_n^{\pm}]_m \left|m\right\rangle, \tag{5.8}$$

where $|m\rangle$ are each $(2\times N_{\text{orb}}\times N)$-component occupied energy eigenvectors of H. We can then construct the projectors onto the positive and negative PsP eigenspaces as

$$P_{\pm} = \sum_{n=1}^{N_{\text{occ}}/2} \left|u_n^{\pm}\right\rangle \left\langle u_n^{\pm}\right|. \tag{5.9}$$

The position operators $\widehat{x}$ and $\widehat{y}$ [SEqs. (5.2) and (5.3)] projected onto the subspace of Image$(P_{\pm})$ are then given by

$$\widehat{x}_{P_{\pm}} = P_{\pm}\widehat{x}P_{\pm},\ \widehat{y}_{P_{\pm}} = P_{\pm}\widehat{y}P_{\pm}. \tag{5.10}$$

From this we can compute the position-space-resolved *partial Chern marker* [1] at the unit cell $\mathbf{R}$ using

$$C^{\pm}(\mathbf{R}) = \frac{2\pi i}{A_{\text{cell}}} \sum_{\alpha=1}^{N_{\text{sta}}} \langle \mathbf{R},\alpha| \left[\widehat{x}_{P_{\pm}}, \widehat{y}_{P_{\pm}}\right] |\mathbf{R},\alpha\rangle, \tag{5.11}$$

in analogy with SEq. (5.5). Note that the partial Chern marker in SEq. (5.11) is defined in terms of position operators projected onto the subspace of Image$(P_{\pm})$. We can then define the *spin Chern maker* in a similar manner as SEq. (3.26):

$$C^s(\mathbf{R}) = C^+(\mathbf{R}) - C^-(\mathbf{R}). \tag{5.12}$$

$C^s(\mathbf{R})$ is then the position-space resolution of the spin Chern number $C^s_{\gamma_1}$ [1, 7, 114] in SEq. (3.26). In particular, for a clean system with an energy gap and a spin gap in the bulk, in the thermodynamic limit the spin Chern marker $C^s(\mathbf{R})$ is independent of $\mathbf{R}$ and converges to the spin Chern number [SEq. (3.26)] when evaluated away from the boundaries. For a system with spinful $\mathcal{T}$ symmetry and a gapped spin spectrum, we have that $\frac{1}{2}\left(C^s_{\gamma_1} \bmod 4\right)$ is equal to the strong $\mathbb{Z}_2$ Kane-Mele invariant for 2D TIs [1, 6, 114, 160]. Crucially, we note for our later discussion that in a 2D non-interacting system in the thermodynamic limit with both an energy and a spin gap, the partial Chern markers $C^{\pm}(\mathbf{R})$ are integers in the bulk, and hence with spinful $\mathcal{T}$ symmetry the spin Chern marker $C^s(\mathbf{R})$ [SEq. (5.12)] is restricted to be an even integer in the bulk.

C. Layer-Resolved Chern Number

The formalism of resolving the Chern number in position space [SN 5 A] can be extended to semi-infinite systems to study how the Chern number of a 2D slab is distributed over the finite direction [36]. To do so, we consider a 2D slab finite along $\mathbf{a}_i$ with N_i unit cells and infinite along $\mathbf{a}_j$ and $\mathbf{a}_l$ where $\{\mathbf{a}_j, \mathbf{a}_l, \mathbf{a}_i\}$ are the primitive lattice vectors of a 3D Bravais lattice. Expressing the Chern number in terms of projection operators [78], we can write

$$C_{jl} = -\frac{i}{2\pi}\int_{-\pi}^{\pi} dk_j \int_{-\pi}^{\pi} dk_l\, \text{Tr}\left\{[P(k_j,k_l)]\left[\frac{\partial[P(k_j,k_l)]}{\partial k_j}, \frac{\partial[P(k_j,k_l)]}{\partial k_l}\right]\right\} \tag{5.13}$$

$$= -\frac{i}{2\pi}\int_{-\pi}^{\pi} dk_j \int_{-\pi}^{\pi} dk_l \sum_{n_i=1}^{N_i}\sum_{\alpha=1}^{N_{\text{sta}}} \langle n_i,\alpha,k_j,k_l| \left([P(k_j,k_l)]\left[\frac{\partial[P(k_j,k_l)]}{\partial k_j}, \frac{\partial[P(k_j,k_l)]}{\partial k_l}\right]\right) |n_i,\alpha,k_j,k_l\rangle, \tag{5.14}$$

where the integral is over the 2D BZ of the slab. In SEqs. (5.13) and (5.14), $[P(k_j,k_l)]$ is the projector

$$[P(k_j,k_l)] = \sum_{n\in\text{occ}} \left|u_{n,k_j,k_l}\right\rangle \left\langle u_{n,k_j,k_l}\right| \tag{5.15}$$

onto the subspace spanned by the N_{occ} occupied energy eigenvectors $|u_{n,k_j,k_l}\rangle$ of the $(N_i N_{\text{sta}} \times N_i N_{\text{sta}})$-dimensional Bloch Hamiltonian matrix $[H(k_j,k_l)]$ for the 2D slab. In SEq. (5.14), $|n_i,\alpha,k_j,k_l\rangle$ are the Fourier-transformed Bloch basis functions at the crystal momentum (k_j,k_l) for the Hilbert space of the system. The index α runs over the (spinful) tight-binding orbitals within each unit cell, and n_i indexes the unit cells along the finite $\mathbf{a}_i$ direction. By interchanging the order of the summation over n_i and the integration over the Brillouin Zone in SEq. (5.14), we can define the *layer-resolved Chern number*

$$C_{jl}(n_i) = -\frac{i}{2\pi}\int_{-\pi}^{\pi} dk_j \int_{-\pi}^{\pi} dk_l \sum_{\alpha=1}^{N_{\text{sta}}} \langle n_i,\alpha,k_j,k_l| \left([P(k_j,k_l)]\left[\frac{\partial[P(k_j,k_l)]}{\partial k_j},\frac{\partial[P(k_j,k_l)]}{\partial k_l}\right]\right)|n_i,\alpha,k_j,k_l\rangle\,, \tag{5.16}$$

such that

$$C_{jl} = \sum_{n_i=1}^{N_i} C_{jl}(n_i). \tag{5.17}$$

$C_{jl}(n_i)$ quantifies how the Chern number of the 2D slab is distributed over the N_i unit cells along the finite direction parallel to $\mathbf{a}_i$. Note that according to our sign convention in SEq. (3.24), to actually identify $C_{jl}(n_i)$ [SEq. (5.16)] as the Chern number of the 2D slab distributed over the finite direction $\mathbf{a}_i$, (jli) should be chosen to align with the right-handed coordinate system, namely a cyclic permutation of (123). Physically, $C_{jl}(n_i)$ tells us how each of the N_i unit cells along the finite direction parallel to $\mathbf{a}_i$ contribute to the Hall conductivity of the slab. It encodes the topology of layers in the bulk of the slab as well as at gapped surfaces. We remind the readers that throughout this section we define a *layer* to consist of a 2D plane of the finite slab that is one *unit cell* thick in the $\mathbf{a}_i$ direction [77, 105]. As such, for the slab we are considering here, we say that it has N_i layers along $\mathbf{a}_i$. Since the matrix projector $[P(k_j,k_l)]$ in SEq. (5.15) is invariant under any $U(N_{\text{occ}})$ gauge transformation of the occupied energy eigenvectors, together with SEq. (5.16) we deduce that the layer-resolved Chern number $C_{jl}(n_i)$ is also invariant under any $U(N_{\text{occ}})$ gauge transformation. In SRefs. [36, 77], $C_{jl}(n_i)$ was used to show that the gapped surfaces of magnetic axion insulators have half-integer Hall conductivity.

D. Layer-Resolved Partial Chern Numbers and Layer-Resolved Spin Chern Number

Building on the formalism for the partial and spin Chern markers in 2D developed in SN 5 B, and the formalism for the layer-resolved local Chern marker in 3D insulators developed in SN 5 C, we will next here formally introduce layer-resolved *partial* and *spin* Chern numbers.

We begin by again considering a 2D slab finite along $\mathbf{a}_i$ with N_i unit cells and infinite along $\mathbf{a}_j$ and $\mathbf{a}_l$ where $\{\mathbf{a}_j,\mathbf{a}_l,\mathbf{a}_i\}$ are the primitive lattice vectors for a 3D Bravais lattice. We take there to be $N_{\text{sta}} = 2N_{\text{orb}}$ tight-binding basis states per unit cell, where the factor of 2 accounts for the spin-1/2 degree of freedom. As in SN 5 B, the spin operator along $\hat{\mathbf{n}}$ is defined as $s \equiv \hat{\mathbf{n}}\cdot\boldsymbol{\sigma}\otimes\mathbb{I}_{N_{\text{orb}}}\otimes\mathbb{I}_{N_i}$ where $\boldsymbol{\sigma}$ acts on the spin-1/2 degree of freedom, and $\mathbb{I}_{N_{\text{orb}}}$ and $\mathbb{I}_{N_i}$ are identity matrices acting on the orbital and layer (unit cell) degrees of freedom, respectively. We suppose that the slab is insulating both in the bulk and on the surfaces with N_{occ} occupied energy bands. To spin-resolve the occupied energy eigenspace, we first construct the $N_{\text{occ}} \times N_{\text{occ}}$ reduced spin matrix [SEq. (3.5)] as a function of momentum in the 2D Brillouin zone,

$$[s_{\text{reduced}}(k_j,k_l)]_{m,n} = \left\langle u_{m,k_j,k_l}\right| s \left|u_{n,k_j,k_l}\right\rangle. \tag{5.18}$$

Here $m,n \in \text{occ}$ index the occupied single-particle eigenstates. From SN 2 B, if N_{occ} is an even number (as is required in insulators with spinful $\mathcal{T}$ symmetry), the spin gap is defined to be open when the $N_{\text{occ}}/2$ spin bands with the largest $[s_{\text{reduced}}(k_j,k_l)]$ eigenvalues (the upper spin bands) are disjoint from the $N_{\text{occ}}/2$ spin bands with the smallest $[s_{\text{reduced}}(k_j,k_l)]$ eigenvalues (the lower spin bands). We refer the readers to SFig. 1(b) for a schematic demonstration of the spin band structure of a spinful $\mathcal{T}$-symmetric system with a spin gap.

Let us assume that the N_{occ} is even and that the spin gap is open. The eigenvalue equation of $[s_{\text{reduced}}(k_j,k_l)]$ can then be written as

$$[s_{\text{reduced}}(k_j,k_l)]\left|\tilde{u}^{\pm}_{n,k_j,k_l}\right\rangle = \lambda^{\pm}_{n,k_j,k_l}\left|\tilde{u}^{\pm}_{n,k_j,k_l}\right\rangle, \tag{5.19}$$

where $n = 1 \ldots N_{\text{occ}}/2$ index the upper and lower spin bands, and $\lambda^{+}_{n,k_j,k_l} > \lambda^{-}_{m,k_j,k_l}$ for all n, m, k_j, and k_l. We may re-express the N_{occ}-component eigenvectors $\left|\tilde{u}^{\pm}_{n,k_j,k_l}\right\rangle$ of $[s_{\text{reduced}}(k_j, k_l)]$ in terms of the spinful tight-binding basis functions using

$$\left|u^{\pm}_{n,k_j,k_l}\right\rangle = \sum_{m=1}^{N_{\text{occ}}} [\tilde{u}^{\pm}_{n,k_j,k_l}]_m \left|u_{m,k_j,k_l}\right\rangle, \tag{5.20}$$

as in SEq. (3.7), where $\left|u_{m,k_j,k_l}\right\rangle$ are the $(2 \times N_{\text{orb}} \times N_i)$-component occupied energy eigenvectors of the Bloch Hamiltonian matrix of the slab. We can then construct the $(2N_{\text{orb}}N_i \times 2N_{\text{orb}}N_i)$-dimensional matrix projector $[P_{\pm}(k_j, k_l)]$ onto the upper $(+)$ and lower $(-)$ spin bands as

$$[P_{\pm}(k_j, k_l)] = \sum_{n=1}^{N_{\text{occ}}/2} \left|u^{\pm}_{n,k_j,k_l}\right\rangle \left\langle u^{\pm}_{n,k_j,k_l}\right|. \tag{5.21}$$

Extending SEq. (5.13) to compute the partial Chern numbers [SEq. (3.24)] within the upper and lower spin bands, the partial Chern numbers of the 2D slab are

$$C^{\pm}_{jl} = -\frac{i}{2\pi}\int_{-\pi}^{\pi} dk_j \int_{-\pi}^{\pi} dk_l \,\text{Tr}\left\{[P_{\pm}(k_j, k_l)]\left[\frac{\partial[P_{\pm}(k_j, k_l)]}{\partial k_j}, \frac{\partial[P_{\pm}(k_j, k_l)]}{\partial k_l}\right]\right\} \tag{5.22}$$

$$= -\frac{i}{2\pi}\int_{-\pi}^{\pi} dk_j \int_{-\pi}^{\pi} dk_l \sum_{n_i=1}^{N_i}\sum_{\alpha=1}^{2N_{\text{orb}}} \langle n_i, \alpha, k_j, k_l| \left([P_{\pm}(k_j, k_l)]\left[\frac{\partial[P_{\pm}(k_j, k_l)]}{\partial k_j}, \frac{\partial[P_{\pm}(k_j, k_l)]}{\partial k_l}\right]\right) |n_i, \alpha, k_j, k_l\rangle. \tag{5.23}$$

In analogy to our logic in SEqs. (5.14) and (5.16), we can interchange the summation over the layer index n_i with the Brillouin zone integration to define the *layer-resolved partial Chern numbers*

$$C^{\pm}_{jl}(n_i) = -\frac{i}{2\pi}\int_{-\pi}^{\pi} dk_j \int_{-\pi}^{\pi} dk_l \sum_{\alpha=1}^{2N_{\text{orb}}} \langle n_i, \alpha, k_j, k_l| \left([P_{\pm}(k_j, k_l)]\left[\frac{\partial[P_{\pm}(k_j, k_l)]}{\partial k_j}, \frac{\partial[P_{\pm}(k_j, k_l)]}{\partial k_l}\right]\right) |n_i, \alpha, k_j, k_l\rangle, \tag{5.24}$$

which satisfy

$$C^{\pm}_{jl} = \sum_{n_i=1}^{N_i} C^{\pm}_{jl}(n_i). \tag{5.25}$$

$C^{\pm}_{jl}(n_i)$ quantifies how the partial Chern numbers [SEq. (3.24)] of the 2D slab are distributed over the N_i unit cells along the finite direction parallel to $\mathbf{a}_i$. Similar to SEq. (3.26), we can define the *layer-resolved spin Chern number* as

$$C^{s}_{jl}(n_i) = C^{+}_{jl}(n_i) - C^{-}_{jl}(n_i). \tag{5.26}$$

We emphasize that it is important to re-express the N_{occ}-component eigenvectors $\left|\tilde{u}^{\pm}_{n,k_j,k_l}\right\rangle$ of $[s_{\text{reduced}}(k_j, k_l)]$ in terms of the spinful tight-binding basis functions using SEq. (5.20) in order to perform the layer resolution in SEq. (5.24). Since the matrix projector $[P_{\pm}(k_j, k_l)]$ [SEq. (5.21)] is invariant under any $U(N_{\text{occ}}/2)$ gauge transformation in the space of (upper or lower) spin bands, then together with SEq. (5.24) we deduce that the layer-resolved partial Chern number $C^{\pm}_{jl}(n_i)$ is also invariant under any $U(N_{\text{occ}}/2)$ gauge transformation within the upper or lower spin bands.

Physically, $C^{s}_{jl}(n_i)$ tells us how the topological contribution to the spin Hall conductivity in SEq. (3.36) of a 2D slab is distributed over the N_i unit cells along the finite direction parallel to $\mathbf{a}_i$. The layer-resolved partial and spin Chern numbers encode the topology of the layers in the bulk of the slab as well as at gapped surfaces, in analogy to the layer-resolved Chern number in magnetic insulators (see SN 5 C and SRefs. [36, 77]). In the next section, SN 5 E, we will numerically apply the layer-resolved partial and spin Chern numbers developed in this section to a model of a helical HOTI in the T-DAXI regime. We will find that the layer-resolved partial Chern numbers surprisingly do not vanish on the gapped surfaces of T-DAXIs, but instead saturate at an anomalous half-integer value, indicating that the gapped surfaces of T-DAXIs exhibit a novel *partial parity anomaly.*

E. Layer-Resolved Spin Chern Number of a Helical HOTI

In this section, we will apply the formalism developed in SN 5 D to compute the layer-resolved partial Chern numbers $C^{\pm}_{jl}(n_i)$ [defined in SEq. (5.24)] for the model of a symmetry-indicated helical higher-order topological insulator (HOTI) with inversion ($\mathcal{I}$) and time-reversal ($\mathcal{T}$) symmetries introduced in SRef. [15] and analyzed in SN 4 E. In SN 4 D and Supplementary Table 6 we demonstrated that this model has a spin gap and realizes the $\mathcal{T}$-doubled axion insulator (T-DAXI) spin-stable topological phase. Now we will explicitly compute the layer-resolved partial Chern numbers and will demonstrate that a semi-infinite 2D slab of a T-DAXI with gapped surfaces satisfies the following properties:

1. It is a 2D time-reversal-invariant strong topological insulator (TI) for highly symmetric surface terminations (*i.e.* in the case in which the entire slab has local $\mathcal{T}$ and global $\mathcal{I}$ symmetry).

2. The partial Chern numbers do not scale with the thickness of the slab, which physically distinguishes the T-DAXI from the 3D QSHI state discussed in SN 4 D. In particular, see SFig. 22 for their individual spin-resolved layer constructions. This implies that the topological contribution to the spin Hall conductivity of a T-DAXI vanishes in the bulk of the slab averaged over each unit cell (layer) along the finite direction.

3. The T-DAXI slab has half-quantized partial Chern numbers at the gapped surfaces, which cannot be realized for an isolated 2D system with energy and spin gaps. Hence the gapped surfaces of a T-DAXI exhibit a novel *partial parity anomaly*, which is the spinful time-reversal-symmetric generalization of the parity anomaly encountered on the gapped surfaces of magnetic axion insulators [79, 106, 220].

We begin by constructing a semi-infinite slab of a T-DAXI. Recall that our model has an orthorhombic lattice. Normalizing the lattice constants to 1, we take the three position-space primitive lattice vectors to be $\mathbf{a}_1 = \hat{x}$, $\mathbf{a}_2 = \hat{y}$, and $\mathbf{a}_3 = \hat{z}$. The dual primitive reciprocal lattice vectors are $\mathbf{G}_1 = 2\pi\hat{x}$, $\mathbf{G}_2 = 2\pi\hat{y}$, and $\mathbf{G}_3 = 2\pi\hat{z}$, satisfying $\mathbf{a}_i \cdot \mathbf{G}_j = 2\pi\delta_{ij}$ $(i, j = 1 \dots 3)$. Our model has eight bands, for which the matrix Bloch Hamiltonian is given explicitly in SEq. (4.125). The tight-binding parameters, specified in SEq. (4.128), together with spin-non-conserving SOC term [$A_{\text{spin}-\text{mixing}}$ in SEq. (4.125)] are chosen such that the energy spectrum of the surfaces with normal vectors $\pm\hat{x}$, $\pm\hat{y}$, and $\pm\hat{z}$ are all gapped. We construct 2D inversion- and time-reversal-symmetric helical HOTI slabs that are finite along $\mathbf{a}_i$ with 15 unit cells and infinite along $\mathbf{a}_j$ and $\mathbf{a}_l$ where (ijl) are cyclic permutations of (123). We below compute the spin spectrum and spin-resolved topology using Ps_zP [SEq. (2.13)], where the occupied energy bands used to form the projector in SEq. (5.15) are the lower half of all the energy bands of the slab. In other words, we consider the slab at half-filling. We will compare the layer-resolved partial Chern numbers of our model with both spin-s_z conservation ($A_{\text{spin}-\text{mixing}} = 0.0$) and with large spin-$s_z$-non-conserving SOC ($A_{\text{spin}-\text{mixing}} = 0.5$).

We next compute the layer-resolved partial Chern numbers $C^{\pm}_{jl}(n_i)$ for each (ijl) a cyclic permutation of (123) using SEq. (5.24). In order to numerically evaluate the integrand in SEq. (5.24), we discretize the 2D BZ of each slab into a 200×200 grid. We use a symmetric finite difference approximation to numerically evaluate derivatives; specifically for a function $f(x)$, we approximate $\frac{df(x)}{dx} \approx \frac{f(x+\frac{1}{2}\Delta x) - f(x-\frac{1}{2}\Delta x)}{\Delta x}$ where Δx is the grid spacing.

We will examine two quantities derived from the layer-resolved partial Chern number. First, we compute the partial Chern number $C^{\pm}_{jl}$ of the entire slab, which is given by summing $C^{\pm}_{jl}(n_i)$ over all of the layers (unit cells):

$$C^{\pm}_{jl} = \sum_{n_i \in \text{ all layers}} C^{\pm}_{jl}(n_i). \tag{5.27}$$

Second, we define the surface partial Chern number, which is given by summing $C^{\pm}_{jl}(n_i)$ over only the layers near the surface:

$$C^{\pm}_{jl,\text{surface}} = \sum_{n_i \in \text{ layers near a surface}} C^{\pm}_{jl}(n_i). \tag{5.28}$$

We will see shortly that for our T-DAXI model, $C^{\pm}_{jl,\text{surface}}$ is well-defined since $C^{\pm}_{jl}(n_i) \to 0$ for n_i in the bulk of the slab. In particular, if $C^{\pm}_{jl}(n_i) \approx 0$ for $N_{\text{bottom}} < n_i < N_{\text{top}}$, then the sum in SEq. (5.28) is over all $n_i < N_{\text{bottom}}$ for the bottom surface, and over all $n_i > N_{\text{top}}$ for the top surface.

In SFig. 27(a,c,e), we show the layer-resolved partial Chern numbers $C^{+}_{jl}(n_i)$ of our three T-DAXI slabs with normal vectors $\mathbf{a}_1$, $\mathbf{a}_2$, and $\mathbf{a}_3$, respectively. As discussed in SN 3 C, using the fact that time-reversal acts locally in the position space, we have in the presence of time-reversal symmetry that

$$C^{+}_{jl}(n_i) = -C^{-}_{jl}(n_i), \tag{5.29}$$

in analogy to SEq. (3.31). There are three main features of $C^{+}_{jl}(n_i)$ in SFig. 27(a,c,e). First, from $C^{\pm}_{jl}(n_i)$, we can compute the partial Chern numbers $C^{\pm}_{jl}$ using SEq. (5.27). As shown in Supplementary Table 7, all of the partial Chern numbers $C^{\pm}_{jl}$ of our helical HOTI slabs, with or without spin-s_z conservation, satisfy $C^{+}_{jl} = -C^{-}_{jl}$ and $C^{+}_{jl} = +1$ or -1. This implies that each slab is a 2D TI with a spin gap [1] [SN 3 C]. This can be viewed as a consequence of the global inversion symmetry of our semi-infinite slabs coupled with the spin-resolved layer construction description of the T-DAXI presented in SN 4 D. This result can also be compared to the analogous case of $\mathcal{I}$-symmetric finite slabs of $\mathcal{I}$-protected magnetic AXI phases, which have integer Chern numbers [15, 36, 92, 220]. However, the integer partial Chern number of the finite slab can be changed by adding a 2D topological insulator with a spin gap to the surface of the slab, and so a priori is not a robust invariant characterizing a T-DAXI phase, as real-material samples generically do not exhibit perfect global $\mathcal{I}$ symmetry.

	$A_{\text{spin}-\text{mixing}} = 0.0$	$A_{\text{spin}-\text{mixing}} = 0.5$
$C^{\pm}_{yz}$	$\pm 0.999404 \approx \pm 1$	$\pm 0.999401 \approx \pm 1$
$C^{\pm}_{zx}$	$\mp 0.999573 \approx \mp 1$	$\mp 0.999469 \approx \mp 1$
$C^{\pm}_{xy}$	$\mp 0.999373 \approx \mp 1$	$\mp 0.999399 \approx \mp 1$

Supplementary Table 7. Numerically computed partial Chern numbers $C^{\pm}_{jl}$ [SEq. (5.27)] for our T-DAXI slabs over different slab stacking directions. $C^{\pm}_{yz}$ are the partial Chern numbers of the slab finite along x, $C^{\pm}_{zx}$ are the partial Chern numbers of the slab finite along y, and $C^{\pm}_{xy}$ are the partial Chern numbers of the slab finite along z. $A_{\text{spin}-\text{mixing}} = 0.0$ and 0.5 correspond to the computations performed using the T-DAXI model with and without spin-s_z conservation, respectively. In each entry we provide the numerical value as well as the closest integer. We see that in all cases $|C^{\pm}_{jl}| \approx 1$, and the deviation from unity can be attributed to numerical error coming from the finite discretization of the BZ.

To contrast the T-DAXI with the 3D QSHI regime of a helical HOTI, we can examine how the partial Chern number is distributed over the layers of the slab. In SFig. 27(a,c,e), we see that the nonzero values of $C^{+}_{jl}(n_i)$ are concentrated around the top and bottom gapped surfaces of the slab. In other words, we have $C^{+}_{jl}(n_i) = 0$ for layers n_i far enough from the gapped surfaces. Therefore, the partial Chern numbers $C^{\pm}_{jl}$ of our helical HOTI slabs, which are in the T-DAXI regime, only have contributions from the gapped surfaces, and thus $|C^{\pm}_{jl}| = 1$ independent of the thickness of the slab. This should be contrasted with a slab obtained from a 3D QSHI state discussed in SN 4 D, which has a nonzero and quantized value of $C^{+}_{jl}(n_i)$ for layers n_i in the bulk region far from the gapped surfaces. For example, the layer-constructed 3D QSHI in Supplementary Table 6 [see also SFig. 22(a)] has $|C^{+}_{jl}(n_i)| = 2$ in the bulk. This shows that the T-DAXI is physically distinguishable from a QSHI state. In particular, this implies that the topological contribution to the spin Hall conductivity of a slab of a T-DAXI is constant and independent of the thickness of the slab, unlike in a QSHI. Furthermore, the topological contribution to the spin Hall conductivity is only nonzero near the boundary of the slab; in the limit in which $A_{\text{spin}-\text{mixing}}$ is zero, this implies that the spin current in response to an electric field only flows near the boundary of the system. Similarly, when $A_{\text{spin}-\text{mixing}} \neq 0$, the topological contribution to the spin current will still flow near the boundary of the system.

Furthermore, we find that the gapped surface of a slab of a T-DAXI is qualitatively distinct from an isolated 2D system. Since $C^{+}_{jl}(n_i)$ in SFig. 27(a,c,e) is concentrated within the vicinity of the top and bottom gapped surfaces of our slabs, then from global inversion symmetry we can obtain the surface partial Chern numbers $C^{\pm}_{jl,\text{surface}}$ [SEq. (5.28)] as

$$C^{\pm}_{jl,\text{surface}} = \frac{1}{2} C^{\pm}_{jl} \tag{5.30}$$

for both the top and bottom gapped surfaces. Therefore, from Supplementary Table 7, we deduce that our helical HOTI slabs have

$$C^{\pm}_{yz,\text{surface}} = \pm\frac{1}{2},\ C^{\pm}_{zx,\text{surface}} = \mp\frac{1}{2},\ C^{\pm}_{xy,\text{surface}} = \mp\frac{1}{2}. \tag{5.31}$$

SEq. (5.31) is also verified in SFig. 27(b,d,f), where we plot the cumulative layer-resolved partial Chern number

$$\sum_{n_i'=\text{bottom}}^{n_i} C^{+}_{jl}(n_i') \tag{5.32}$$

summed over layers beginning at the bottom of the slab. We see that the cumulative layer-resolved partial Chern number [SEq. (5.32)] quickly converges to $\pm\frac{1}{2}$ for n_i in the bulk region of the slab. It is then constant throughout the bulk, and finally converges to ± 1 when n_i reaches the top layer of the slab.

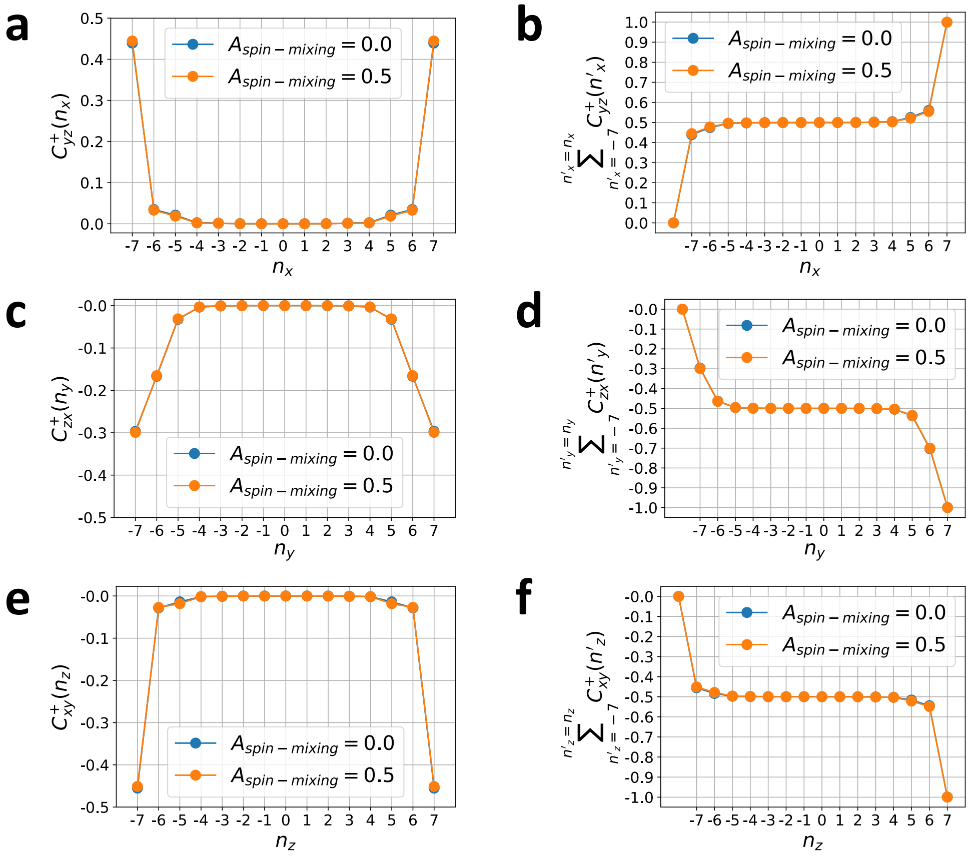

Supplementary Figure 27. Layer-resolved partial Chern numbers $C^+_{jl}(n_i)$ of 2D slabs formed from our model of an $\mathcal{I}$- and $\mathcal{T}$-symmetric helical HOTI [SEq. (4.125)] in the T-DAXI regime (see SN 4 E). We consider T-DAXIs both with ($A_{\text{spin-mixing}} = 0.0$) and without ($A_{\text{spin-mixing}} = 0.5$) spin-$s_z$ conservation. Due to time-reversal symmetry we have $C^+_{jl}(n_i) = -C^-_{jl}(n_i)$, hence we show only $C^+_{jl}(n_i)$ in this figure. (a) shows the layer-resolved partial Chern number $C^+_{yz}(n_x)$ for a 2D slab finite along x with 15 unit cells and infinite along y and z. (b) shows the cumulative partial Chern number $\sum_{n'_x=-7}^{n'_x=n_x} C^+_{yz}(n'_x)$ as a function of n_x beginning from the bottom layer in (a). (c) shows the layer-resolved partial Chern number $C^+_{zx}(n_y)$ for a 2D slab finite along y with 15 unit cells and infinite along z and x. (d) shows the cumulative partial Chern number $\sum_{n'_y=-7}^{n'_y=n_y} C^+_{zx}(n'_y)$ as a function of n_y beginning from the bottom layer in (c). (e) shows the layer-resolved partial Chern number $C^+_{xy}(n_z)$ for a 2D slab finite along z with 15 unit cells and infinite along x and y. (f) shows the cumulative partial Chern number $\sum_{n'_z=-7}^{n'_z=n_z} C^+_{xy}(n'_z)$ as a function of n_z beginning from the bottom layer in (e). As we can see in (a), (c) and (e), the nonzero values of $C^+_{jl}(n_i)$ are concentrated around the top and bottom layers, and this feature persists when spin-s_z conservation is broken due to a nonzero value of the SOC term $A_{\text{spin-mixing}}$ in SEq. (4.125). In addition, (b), (d), and (f) demonstrate the appearance of anomalous, half-quantized partial Chern numbers around the gapped surfaces of the T-DAXI, as the cumulative values of $C^+_{jl}(n_i)$ beginning from the bottom layer quickly converge to $\pm\frac{1}{2}$ and remain constant in the bulk. The numerical values of the partial Chern numbers obtained by summing $C^+_{jl}(n_i)$ over all the layers for (b), (d), and (f) are given in Supplementary Table 7. The calculations detailed in this figure were performed using the freely available Python package NESTED_AND_SPIN_RESOLVED_WILSON_LOOP [97], which represents an extension of the PythTB open-source Python tight-binding package [171] that was implemented and utilized for the preparation of SRefs. [8, 15] and the present work.

While the global inversion symmetry of our slabs allowed us to quickly deduce SEqs. (5.30) and (5.31), the fact that the gapped surface of a T-DAXI slab carries a half-integer partial Chern number is independent of the global inversion symmetry of the slab. To see this, first note that we can always break global inversion symmetry by "gluing" a layer of 2D TI to just one surface of our helical HOTI slab without breaking time-reversal symmetry. Recall from SN 3 C that a 2D TI with a spin gap has the partial Chern numbers $C^+_{jl} = -C^-_{jl}$ with C^+_{jl} an odd integer. Hence, if we add any number of 2D TIs to the top surface of our T-DAXI slab, the partial Chern numbers at the top surface

will satisfy

$$C^{+}_{jl,\text{surface}} = -C^{-}_{jl,\text{surface}}, \tag{5.33}$$

and

$$C^{+}_{jl,\text{surface}} = \frac{1}{2} + n \tag{5.34}$$

where $n \in \mathbb{Z}$. Furthermore, the fact that the entire slab of a T-DAXI must have integer partial Chern number implies that local perturbations to the top surface of the sample cannot modify SEq. (5.34) without closing either a spin gap or an energy gap. The half-integer surface partial Chern number hence provides a robust surface signature of the spin-stable T-DAXI phase, *even* when spin-conservation symmetry is broken by SOC.

The half-integer partial Chern number at the surface of a T-DAXI distinguishes the surface of a T-DAXI from an isolated 2D insulator with an energy and spin gap (which can only have an integer partial Chern number). In that sense, the gapped surface of a T-DAXI is an anomalous 2D system. There is a direct analogy between the anomaly at the surface of a T-DAXI and the parity anomaly. Recall that the parity anomaly in a magnetic axion insulator (AXI) is manifested through a half-quantized Chern number on gapped surfaces [15, 16, 36, 77]. In a magnetic AXI this is a direct consequence of the bulk-quantized electromagnetic theta (axion) angle $\theta = \pi$, which implies a half-integer Chern number on gapped surfaces, which represent "axion domain walls" [80] between the $\theta = \pi$ bulk and the $\theta = 0$ vacuum. The quantized value of θ in the bulk compensates for the parity anomaly on the surface. The half-quantized *partial* Chern numbers in SEq. (5.34) on the gapped surfaces of a T-DAXI similarly exhibit a *partial* parity anomaly, which is a spinful and time-reversal-symmetric generalization of the parity anomaly. In other words, when we examine the spin-stable topology of a T-DAXI, each of the (positive and negative) PsP eigenspaces exhibits a half-quantized partial Chern numbers on gapped surfaces. For an isolated, lattice-regularized 2D spinful system with time-reversal symmetry and a spin gap, the partial Chern numbers must be integral. Therefore, the half-quantized partial Chern numbers on the 2D surface of a T-DAXI are anomalous, exhibiting a *partial* parity anomaly in each of the positive and negative PsP eigenspaces. The anomaly is compensated by the bulk, which we showed in SN 4 D and SEq. (4.123) to have an $\mathcal{I}$-quantized *partial* axion angle $\theta^{\pm} = \pi$. We thus see that a T-DAXI is uniquely characterized by a bulk partial axion angle quantized by inversion symmetry to $\theta^{\pm} = \pi$ and corresponding anomalous surfaces with half-integer partial Chern numbers [SEq. (5.34)] satisfying SEq. (5.33) due to time-reversal symmetry. In particular, the $1/2$ in SEq. (5.34) implies that the anomalous surfaces of a T-DAXI have partial Chern numbers that are halves of the partial Chern numbers of lattice-regularized 2D TIs, which are always odd integers [1]. Going further, SEqs. (5.33) and (5.34) together imply that the surface spin Chern number of a T-DAXI satisfies

$$C^{s}_{jl,\text{surface}} = C^{+}_{jl,\text{surface}} - C^{-}_{jl,\text{surface}} = 2n + 1 \tag{5.35}$$

for some integer n. As discussed in SN 3 D, an isolated lattice-regularized 2D system with an energy gap and a spin gap cannot have an odd spin Chern number without interactions [235]. Thus SEq. (5.35) further demonstrates that the surface of a T-DAXI is anomalous.

We further note that SRef. [174] recently demonstrated that upon the adiabatic insertion of a U(1) magnetic flux, the gapped surface of a T-DAXI exhibits half of the response of an isolated 2D TI. The layer-resolved partial Chern numbers of our T-DAXI slabs in SFig. 27 are consistent with this result, since the half-quantized partial Chern numbers at the gapped surfaces imply that the topological contribution to the spin Hall conductivity [SEq. (3.36)] at the gapped surfaces will be

$$[\sigma^{s}_{H}]_{\text{surface,topological}} = \frac{e}{4\pi} C^{s}_{jl,\text{surface}} = \frac{e}{4\pi}(1 + 2n) \tag{5.36}$$

where we have used SEqs. (3.36) and (5.35) with $n \in \mathbb{Z}$. On the contrary, for an isolated, lattice regularized, and spin-gapped 2D TI the topological contribution to the spin Hall conductivity [SEq. (3.36)] must be

$$[\sigma^{s}_{H}]_{\text{2D TI,topological}} = \frac{e}{4\pi} \cdot 2 \cdot (1 + 2m) \tag{5.37}$$

where $m \in \mathbb{Z}$, and we have used the fact that an isolated, spinful $\mathcal{T}$-invariant, lattice-regularized 2D TI with an energy gap and a spin gap has $C^{+}_{\gamma_1} = -C^{-}_{\gamma_1}$ and $C^{s}_{\gamma_1} \mod 4 = 2$. In other words, the topological contribution $[\sigma^{s}_{H}]_{\text{surface,topological}}$ to the surface spin Hall conductivity of a T-DAXI is half-integer quantized in units of the spin Hall conductivity of an isolated 2D system. In particular, combining the results of Supplementary Table 7 with our analysis of flux response in SN 3 G, we deduce that in the presence of a π magnetic flux a slab of T-DAXI will bind one spinon between the top and bottom surface, with on average "half" a spinon per surface. From the point of view

of responses to external applied fields, we hence conclude that the gapped surfaces of a T-DAXI may be viewed in a precise sense as a half of an isolated 2D TI. We remark that it was previously predicted in SRef. [236] that the gapped top surfaces of weak TI states exhibit similar halves of 2D QSHIs—the position-space calculations performed in this section reveal the surface half-2D TI state to be more generic, appearing on all the gapped surfaces of helical HOTIs in the T-DAXI regime. As a final remark, note that in SFig. 27(a–f), we observe that the surface partial Chern numbers in a T-DAXI remain nearly quantized to half integers, even when s_z-conservation is broken by SOC, as long as the energy and the spin gap remains open both in the bulk and on the surfaces. Therefore, we see that the *partial* parity anomaly remains robust as long as the spin gap both in the bulk and on the gapped surfaces remains open.

6. SYMMETRY CONSTRAINTS ON WILSON LOOP SPECTRA

In this section we derive constraints that unitary inversion ($\mathcal{I}$) and antiunitary time-reversal ($\mathcal{T}$) symmetry place on the Wilson loop spectra and their nested and spin-resolved generalizations. Throughout this section, we will consider 3D translation-invariant systems with position-space primitive lattice vectors $\{\mathbf{a}_1, \mathbf{a}_2, \mathbf{a}_3\}$ and dual primitive reciprocal lattice vectors $\{\mathbf{G}_1, \mathbf{G}_2, \mathbf{G}_3\}$ such that $\mathbf{a}_i \cdot \mathbf{G}_j = 2\pi\delta_{ij}$ $(i, j = 1 \ldots 3)$. Any crystal momentum $\mathbf{k}$ can be expanded as $\sum_{j=1}^{3} \frac{k_j}{2\pi}\mathbf{G}_j$ with reduced $k_j = \mathbf{k} \cdot \mathbf{a}_j$. In the text below, the first BZ is taken to be the region defined by $k_j \in [-\pi, \pi)$ for all $j = 1, 2, 3$. We will denote $\mathbf{k}$ points by their coordinates as (k_1, k_2, k_3). We will denote the number of (spinful) tight-binding basis states in the primitive unit cell as N_{sta}. When the tight-binding model has spin-1/2 degree of freedom, we will assume $N_{\text{sta}} = 2N_{\text{orb}}$, where N_{orb} is the number of orbitals (such as s, p, and d orbitals) and the factor of 2 accounts for the spin-1/2 degree of freedom. In addition, we will assume that both the energy spectrum and PsP spectrum are gapped at every $\mathbf{k}$ in the BZ such that projectors onto the energy and spin bands are well-defined and are smooth functions of $\mathbf{k}$.

Throughout the derivation in this section, we will focus on the eigenstates and the eigenvalues of (nested) (spin-resolved) Wilson loop operators represented as products of $N_{\text{sta}} \times N_{\text{sta}}$ projection matrices acting in the tight-binding Hilbert space (see SEqs. (3.4), (3.19) and the surrounding text, and where we have included an additional $[V(\mathbf{G})]$ at the end of the products in order to close the loop in $\mathbf{k}$-space in a gauge-invariant manner [4, 107]). This will allow us to derive symmetry constraints on the Wilson loop spectra in terms of the matrix representatives of symmetries in the tight-binding basis states, which can be generated from the symmetry data of the crystal [224]. The spectrum of an $N_{\text{sta}} \times N_{\text{sta}}$ (nested) (spin-resolved) Wilson loop operator contains a set of unimodular eigenvalues that coincide with the eigenphases of the (nested) (spin-resolved) Wilson loop matrices defined in SEqs. (3.2), (3.17), (4.24), and (4.47). In addition, the spectrum of an $N_{\text{sta}} \times N_{\text{sta}}$ (nested) (spin-resolved) Wilson loop operator also contains a set of zero eigenvalues whose eigenvectors correspond to the unoccupied (energy, PsP, Wannier band, or spin-resolved Wannier band) eigenstates.

We will in this section construct P- and $P_{\pm}$-Wilson loops parallel to the $\mathbf{G}_1$ direction in the BZ (see SN 3 A and 3 B). Similarly, we will use $\mathbf{G}_1$ and $\mathbf{G}_2$ as the directions for the first and second closed loop needed to define nested $P-$ and nested $P_{\pm}$-Wilson loops (see SN 4 A and 4 B). We will introduce a simplified notation throughout the derivation (in SN 6 A–6 D) to make the calculation explicit, while we will generalize the relevant results for (nested) (spin-resolved) Wilson loops oriented along *arbitrary* primitive reciprocal lattice vectors using the general notation of SN 3 A, 3 B, 4 A, and 4 B.

The remainder of this section is organized as follows. In SN 6 A and 6 B, we will derive the constraints from $\mathcal{I}$—a unitary symmetry—on the P- and nested P-Wilson loop spectra, respectively. We will show that $\mathcal{I}$ places a quantization condition on the P- and nested P-Wilson loop eigenphases at the time-reversal invariant momenta. In SN 6 C and 6 D, we will derive the constraints from $\mathcal{T}$—an antiunitary symmetry—on the P- and nested P-Wilson loop spectra, respectively, and a set of general results that hold for both spinless $\mathcal{T}$ ($\mathcal{T}^2 = 1$, where 1 is shorthand for the identity operator) and spinful $\mathcal{T}$ ($\mathcal{T}^2 = -1$, where 1 is shorthand for the identity operator) will be presented. We will then show that if we specialize to the case with $\mathcal{T}^2 = -1$, there will be Kramers' degeneracies in the P- and nested P-Wilson loop spectra at TRIMs. In SN 6 E and 6 F, we will derive the constraints from $\mathcal{I}$ on the $P_{\pm}$- and nested $P_{\pm}$-Wilson loop spectra respectively for a spinful system, in which we also derive a quantization condition enforced by $\mathcal{I}$ at TRIM points. In SN 6 G and 6 H, we will derive the constraints from $\mathcal{T}$ on the $P_{\pm}$- and nested $P_{\pm}$-Wilson loop spectra respectively for a spinful system with $\mathcal{T}^2 = -1$, where $\mathcal{T}$ acts to flip the spin. In SN 6 I, we will present a short summary of the symmetry constraints on the (nested) (spin-resolved) Wilson loop spectra derived throughout this section. In SN 6 J, we will briefly review the relation between the eigenvalues and eigenvectors of Wilson loop operators and their Hermitian conjugates, which will be used throughout this section. Throughout this section, we will denote $[\mathcal{I}]$ and $[\mathcal{T}] = [U_{\mathcal{T}}]\mathcal{K}$ as the representatives of $\mathcal{I}$ and $\mathcal{T}$ ($\mathcal{T}^2$ can be either $+1$ or -1) in the tight-binding basis states. We will present the construction of the representatives for $\mathcal{I}$ and $\mathcal{T}$ in SN 6 K and 6 L respectively, in which we will also derive how the matrix $[V(\mathbf{G})]$ in SEq. (2.8) transforms under $\mathcal{I}$ and $\mathcal{T}$. Although the constraints

presented in SN 6 A, 6 B 6 C, 6 H, 6 K and 6 L are not new (see for example SRefs. [4, 13–15, 107, 237]), we will review them here for completeness, and to complement the results of SN 6 E, 6 F, 6 G, and 6 H, which are derived for the first time in the present work.

A. Unitary $\mathcal{I}$ Constraint on the P-Wilson Loop

We begin by constructing the P-Wilson loop associated to a projector P onto a set of spectrally isolated occupied bands. Recall from SN 3 A and SRefs. [3, 4, 148] that the $\widehat{\mathbf{G}}_1$-directed P-Wilson loop operator with base point $\mathbf{k}=(k_1,k_2,k_3)$ can be written as

$$\mathcal{W}_{1,\mathbf{k},\mathbf{G}_1}=[V(\mathbf{G}_1)]\lim_{N\to\infty}\left([P(k_1+2\pi,k_2,k_3)][P(k_1+\frac{2\pi(N-1)}{N},k_2,k_3)]\cdots[P(k_1+\frac{2\pi}{N},k_2,k_3)][P(k_1,k_2,k_3)]\right), \tag{6.1}$$

where the product of projectors $[P(\mathbf{k})]$ is taken along a straight path from $\mathbf{k}$ to $\mathbf{k}+\mathbf{G}_1$, or equivalently from (k_1,k_2,k_3) to $(k_1+2\pi,k_2,k_3)$ in reduced coordinates. In SEq. (6.1) the matrix $[V(\mathbf{G})]$ defined in SEq. (2.8) encodes the positions of the tight-binding basis orbitals, and $[P(\mathbf{k})]$ is the projector onto the occupied eigenvectors $|u_{n,\mathbf{k}}\rangle$ of the Bloch Hamiltonian as defined in SEq. (3.1). For completeness, we summarize the important properties of $[V(\mathbf{G})]$ and $[P(\mathbf{k})]$ below:

$$[V(\mathbf{G})]_{\alpha\beta}=e^{i\mathbf{G}\cdot\mathbf{r}_\alpha}\delta_{\alpha\beta}, \tag{6.2}$$

$$[P(\mathbf{k})]=\sum_{n=1}^{N_{\text{occ}}}|u_{n,\mathbf{k}}\rangle\langle u_{n,\mathbf{k}}|, \tag{6.3}$$

$$|u_{n,\mathbf{k}+\mathbf{G}}\rangle=[V(\mathbf{G})]^{-1}|u_{n,\mathbf{k}}\rangle=[V(\mathbf{G})]^{\dagger}|u_{n,\mathbf{k}}\rangle, \tag{6.4}$$

where $\alpha,\beta=1\ldots N_{\text{sta}}$ in SEq. (6.2) label the tight-binding basis states within the primitive unit cell, and N_{occ} in SEq. (6.3) is the number of occupied energy bands. From the definition in SEq. (6.3), the matrix projector $[P(\mathbf{k})]$ is Hermitian, such that

$$[P(\mathbf{k})]^{\dagger}=[P(\mathbf{k})]. \tag{6.5}$$

For a system with inversion symmetry, $[V(\mathbf{G})]$ and $[P(\mathbf{k})]$ transform under $\mathcal{I}$ according to

$$[\mathcal{I}][V(\mathbf{G})][\mathcal{I}]^{\dagger}=[V(-\mathbf{G})], \tag{6.6}$$

$$[\mathcal{I}][P(\mathbf{k})][\mathcal{I}]^{\dagger}=[P(-\mathbf{k})]. \tag{6.7}$$

For the proof of SEq. (6.6) and also the explicit construction of $[\mathcal{I}]$ as the unitary matrix representative of $\mathcal{I}$ in the tight-binding basis states, see SN 6 K and SRefs. [4, 107]. Note that $[P(\mathbf{k})]$ must project onto a set of energy bands that is mapped into itself under $\mathcal{I}$ in order for SEq. (6.7) to be satisfied. This is guaranteed if $[P(\mathbf{k})]$ projects onto a set of gapped energy bands for an $\mathcal{I}$-symmetric Hamiltonian. Using SEqs. (6.2), (6.3), (6.4), (6.5), (6.6), and (6.7), it follows that under the action of $\mathcal{I}$, the $\widehat{\mathbf{G}}_1$-directed P-Wilson loop operator $\mathcal{W}_{1,\mathbf{k},\mathbf{G}_1}$ transforms according to

$$\begin{aligned}
[\mathcal{I}]\mathcal{W}_{1,\mathbf{k},\mathbf{G}_1}[\mathcal{I}]^{\dagger}&=[\mathcal{I}][V(\mathbf{G}_1)][P(k_1+2\pi,k_2,k_3)]\cdots[P(k_1,k_2,k_3)][\mathcal{I}]^{\dagger}\\
&=[\mathcal{I}][V(\mathbf{G}_1)][\mathcal{I}]^{\dagger}[\mathcal{I}][P(k_1+2\pi,k_2,k_3)][\mathcal{I}]^{\dagger}\cdots[\mathcal{I}][P(k_1,k_2,k_3)][\mathcal{I}]^{\dagger}\\
&=[V(-\mathbf{G}_1)][P(-k_1-2\pi,-k_2,-k_3)]\cdots[P(-k_1,-k_2,-k_3)]\\
&=[V(-\mathbf{G}_1)][V(-\mathbf{G}_1)]^{-1}[P(-k_1,-k_2,-k_3)][V(-\mathbf{G}_1)]\cdots[V(-\mathbf{G}_1)]^{-1}[P(-k_1+2\pi,-k_2,-k_3)][V(-\mathbf{G}_1)]\\
&=[P(-k_1,-k_2,-k_3)]\cdots[P(-k_1+2\pi,-k_2,-k_3)][V(-\mathbf{G}_1)]\\
&=\left([V(-\mathbf{G}_1)]^{\dagger}[P(-k_1+2\pi,-k_2,-k_3)]\cdots[P(-k_1,-k_2,-k_3)]\right)^{\dagger}\\
&=\left([V(\mathbf{G}_1)][P(-k_1+2\pi,-k_2,-k_3)]\cdots[P(-k_1,-k_2,-k_3)]\right)^{\dagger}\\
&=\mathcal{W}^{\dagger}_{1,-\mathbf{k},\mathbf{G}_1},
\end{aligned} \tag{6.8}$$

where $[P(\mathbf{k}_f)]\cdots[P(\mathbf{k}_i)]$ is a shorthand notation for the product of projection matrices along a straight path in $\mathbf{k}$-space from $\mathbf{k}_i$ to $\mathbf{k}_f$. $\mathcal{W}_{1,\mathbf{k},\mathbf{G}_1}$ has $N_{\text{sta}}-N_{\text{occ}}$ zero eigenvalues corresponding to the number of unoccupied eigenstates. More importantly, $\mathcal{W}_{1,\mathbf{k},\mathbf{G}_1}$ has N_{occ} unimodular eigenvalues that are independent of k_1 [4, 78, 146]. For simplicity,

we denote the set of unimodular eigenvalues of $\mathcal{W}_{1,\mathbf{k},\mathbf{G}_1}$ as $\{e^{i\gamma_{1,j}(k_2,k_3)}|j=1\dots N_{\text{occ}}\}$ where j is the Wannier band index. SEq. (6.8) implies that $\mathcal{W}_{1,\mathbf{k},\mathbf{G}_1}$ and $\mathcal{W}^{\dagger}_{1,-\mathbf{k},\mathbf{G}_1}$ are isospectral since they are related to each other by a unitary transformation. According to SEq. (6.8), and using the fact that the eigenvalues of $\mathcal{W}^{\dagger}_{1,-\mathbf{k},\mathbf{G}_1}$ coincide with the complex conjugates of the eigenvalues of $\mathcal{W}_{1,-\mathbf{k},\mathbf{G}_1}$ (see SN 6 J for more details), we have that

$$\{e^{i\gamma_{1,j}(k_2,k_3)}|j=1\dots N_{\text{occ}}\}=\{e^{-i\gamma_{1,j}(-k_2,-k_3)}|j=1\dots N_{\text{occ}}\}. \tag{6.9}$$

In terms of the set of eigenphases $\gamma_{1,j}(k_2,k_3)$, SEq. (6.9) implies that

$$\{\gamma_{1,j}(k_2,k_3)|j=1\dots N_{\text{occ}}\}\bmod 2\pi=\{-\gamma_{1,j}(-k_2,-k_3)|j=1\dots N_{\text{occ}}\}\bmod 2\pi. \tag{6.10}$$

We refer to the $\mathcal{I}$ constraint in SEq. (6.10) as an effective "particle-hole" symmetry in the P-Wilson loop eigenphases. A more operational way to interpret SEq. (6.10) is that the P-Wannier spectrum $\{\gamma_{1,j}(k_2,k_3)|j=1\dots N_{\text{occ}}\}$ is invariant under a simultaneous sign-change of the momenta $(k_2,k_3)\to(-k_2,-k_3)$ and the phase $\gamma_1\to-\gamma_1$. Recall from SN 3 A that the eigenphases [SEq. (6.10)] of the P-Wilson loop operator [SEq. (6.1)] correspond to the localized positions of hybrid Wannier functions formed from the set of occupied states [4, 13, 14, 78, 146]. With this in mind, SEq. (6.10) then follows from the fact that inversion flips both position and momentum of a hybrid Wannier function.

We can also compute the sum over j of the $\gamma_{1,j}(k_2,k_3)$ which defines the total Berry phase [3, 4, 163, 238]

$$\gamma_1(k_2,k_3)\equiv\sum_{j=1}^{N_{\text{occ}}}\gamma_{1,j}(k_2,k_3)\bmod 2\pi, \tag{6.11}$$

where SEq. (6.10) indicates that

$$\gamma_1(k_2,k_3)\bmod 2\pi=-\gamma_1(-k_2,-k_3)\bmod 2\pi. \tag{6.12}$$

Recall that SEq. (4.17) implies that the eigenphases $\gamma_1(k_2,k_3)$ are invariant if we shift the base point by a reciprocal lattice vector. Using this fact, SEq. (6.12) implies that at the four time-reversal invariant momenta (TRIMs) $(k_2^{TRIM},k_3^{TRIM})=(0,0)$, $(\pi,0)$, $(0,\pi)$, and (π,π), we have

$$\left(\gamma_1(k_2^{TRIM},k_3^{TRIM})\mod\pi\right)=0. \tag{6.13}$$

Since inversion symmetry treats all momentum components k_i in the same way—it flips the sign of all the components k_i—the results of this section can be generalized to $\widehat{\mathbf{G}}_2$- and $\widehat{\mathbf{G}}_3$-directed P-Wilson loops. The results of SEqs. (6.9), (6.10), (6.12), and (6.13), written in the general notation in SN 3 A, therefore generalize to

$$\{e^{i(\gamma_1)_{j,\mathbf{k},\mathbf{G}}}|j=1\dots N_{\text{occ}}\}=\{e^{-i(\gamma_1)_{j,-\mathbf{k},\mathbf{G}}}|j=1\dots N_{\text{occ}}\}, \tag{6.14}$$
$$\{(\gamma_1)_{j,\mathbf{k},\mathbf{G}}|j=1\dots N_{\text{occ}}\}\bmod 2\pi=\{-(\gamma_1)_{j,-\mathbf{k},\mathbf{G}}|j=1\dots N_{\text{occ}}\}\bmod 2\pi, \tag{6.15}$$
$$(\gamma_1)_{\mathbf{k},\mathbf{G}}\bmod 2\pi=-(\gamma_1)_{-\mathbf{k},\mathbf{G}}\bmod 2\pi, \tag{6.16}$$
$$\left((\gamma_1)_{\mathbf{k}_{TRIM},\mathbf{G}}\mod\pi\right)=0, \tag{6.17}$$

where

$$(\gamma_1)_{\mathbf{k},\mathbf{G}}\equiv\sum_{j=1}^{N_{\text{occ}}}(\gamma_1)_{j,\mathbf{k},\mathbf{G}}\bmod 2\pi, \tag{6.18}$$

and $(\gamma_1)_{j,\mathbf{k},\mathbf{G}}$ are the eigenphases of the $\widehat{\mathbf{G}}$-directed P-Wilson loop operator $\mathcal{W}_{1,\mathbf{k},\mathbf{G}}$ satisfying

$$[\mathcal{I}]\mathcal{W}_{1,\mathbf{k},\mathbf{G}}[\mathcal{I}]^{\dagger}=\mathcal{W}^{\dagger}_{1,-\mathbf{k},\mathbf{G}}. \tag{6.19}$$

SEq. (6.19) is true provided that the matrix projector onto the occupied energy bands is inversion-symmetric as specified in SEq. (6.7). Finally, since $(\gamma_1)_{j,\mathbf{k},\mathbf{G}}$ is independent of the momentum component $\mathbf{k}\cdot\mathbf{a}$, where $\mathbf{a}$ is the real-space primitive lattice vector dual to the primitive reciprocal lattice vector $\mathbf{G}$, $\mathbf{k}_{TRIM}$ in SEq. (6.17) should be interpreted as a $\mathbf{k}$-vector with $(\mathbf{k}_{TRIM}\cdot\mathbf{a}')\mod\pi=0$ for each primitive lattice vector $\mathbf{a}'\neq\mathbf{a}$.

B. Unitary $\mathcal{I}$ Constraint on the Nested P-Wilson Loop

Recall that to construct the nested P-Wilson loop operator from SN 4 A, we must first specify two primitive reciprocal lattice vectors $\mathbf{G}_1$ and $\mathbf{G}_2$, respectively. We then solve the eigenvalue equation for the $\widehat{\mathbf{G}}_1$-directed P-Wilson loop operator [SEq. (6.1)],

$$\mathcal{W}_{1,\mathbf{k},\mathbf{G}_1}|w_j(\mathbf{k})\rangle = e^{i\gamma_{1,j}(k_2,k_3)}|w_j(\mathbf{k})\rangle, \tag{6.20}$$

where the eigenvectors $\{|w_j(\mathbf{k})\rangle | j = 1\ldots N_{\text{occ}}\}$ with unimodular eigenvalues form an orthonormal set such that

$$\langle w_j(\mathbf{k})|w_{j'}(\mathbf{k})\rangle = \delta_{jj'}. \tag{6.21}$$

In SEq. (6.20), the Wannier band basis vector $|w_j(\mathbf{k})\rangle$ lies in the image of $[P(\mathbf{k})]$ and is an eigenvector of $\mathcal{W}_{1,\mathbf{k},\mathbf{G}_1}$ with unimodular eigenvalue $e^{i\gamma_{1,j}(k_2,k_3)}$ [14]. Although the eigenvalue $e^{i\gamma_{1,j}(k_2,k_3)}$ of $\mathcal{W}_{1,\mathbf{k},\mathbf{G}_1}$ [SEq. (6.20)] is independent of k_1 (the component of the momentum along $\mathbf{G}_1$), the eigenvector $|w_j(\mathbf{k})\rangle$ depends on all momentum components k_1, k_2, and k_3 of $\mathbf{k}$. In particular, if we expand $|w_j(\mathbf{k})\rangle$ in terms of the occupied energy eigenvectors $\{|u_{m,\mathbf{k}}\rangle | m = 1\ldots N_{\text{occ}}\}$ as

$$|w_j(\mathbf{k})\rangle = \sum_{m=1}^{N_{\text{occ}}} [\nu_j(\mathbf{k})]_m |u_{m,\mathbf{k}}\rangle, \tag{6.22}$$

then as discussed in SN 4 A the N_{occ}-component vector $|\nu_j(\mathbf{k})\rangle$ satisfies the parallel transport condition in SEq. (4.5).

Let us consider an inversion-symmetric system such that the $\widehat{\mathbf{G}}_1$-directed P-Wilson loop operator satisfies SEq. (6.8). Acting with $[\mathcal{I}]$, the unitary matrix representative of $\mathcal{I}$ in the tight-binding basis states, on both sides of the eigenvalue equation in SEq. (6.20), we have

$$[\mathcal{I}]\mathcal{W}_{1,\mathbf{k},\mathbf{G}_1}[\mathcal{I}]^\dagger[\mathcal{I}]|w_j(\mathbf{k})\rangle = e^{i\gamma_{1,j}(k_2,k_3)}[\mathcal{I}]|w_j(\mathbf{k})\rangle, \tag{6.23}$$

where we have inserted the identity matrix $[\mathcal{I}]^\dagger[\mathcal{I}]$ since $[\mathcal{I}]$ is unitary. Combining SEqs. (6.8) and (6.23), we obtain

$$\mathcal{W}^\dagger_{1,-\mathbf{k},\mathbf{G}_1}[\mathcal{I}]|w_j(\mathbf{k})\rangle = e^{i\gamma_{1,j}(k_2,k_3)}[\mathcal{I}]|w_j(\mathbf{k})\rangle. \tag{6.24}$$

Recall that the eigenvector of $\mathcal{W}_{1,\mathbf{k},\mathbf{G}_1}$ with eigenvalue $e^{i\gamma_{1,j}(k_2,k_3)}$ is also the eigenvector of $\mathcal{W}^\dagger_{1,\mathbf{k},\mathbf{G}_1}$ with eigenvalue $e^{-i\gamma_{1,j}(k_2,k_3)}$ (see SN 6 J for more details). From SEq. (6.24), we then deduce that for a given eigenvector $|w_j(\mathbf{k})\rangle$ of $\mathcal{W}_{1,\mathbf{k},\mathbf{G}_1}$ with eigenvalue $e^{i\gamma_{1,j}(k_2,k_3)}$, $[\mathcal{I}]|w_j(\mathbf{k})\rangle$ is an eigenvector of $\mathcal{W}_{1,-\mathbf{k},\mathbf{G}_1}$ with eigenvalue $e^{-i\gamma_{1,j}(k_2,k_3)}$. In other words, we have

$$\mathcal{W}_{1,-\mathbf{k},\mathbf{G}_1}[\mathcal{I}]|w_j(\mathbf{k})\rangle = e^{-i\gamma_{1,j}(k_2,k_3)}[\mathcal{I}]|w_j(\mathbf{k})\rangle. \tag{6.25}$$

In addition to the effective particle-hole symmetry of the $\widehat{\mathbf{G}}_1$-directed P-Wannier bands that we have already established in SEq. (6.10), SEq. (6.25) implies that the eigenvectors $\{|w_j(\mathbf{k})\rangle\, | j = 1\ldots N_{\text{occ}}\}$ and $\{|w_j(-\mathbf{k})\rangle\, | j = 1\ldots N_{\text{occ}}\}$ of $\mathcal{W}_{1,\mathbf{k},\mathbf{G}_1}$ and $\mathcal{W}_{1,-\mathbf{k},\mathbf{G}_1}$, respectively, are related to each other by $[\mathcal{I}]$.

Suppose that the $\widehat{\mathbf{G}}_1$-directed P-Wannier bands can be separated into disjoint groupings and we choose a grouping of N_W bands described by

$$\{\gamma_{1,j}(k_2,k_3) | j = 1\ldots N_W\} \tag{6.26}$$

to form the projector

$$[\widetilde{P}(\mathbf{k})] = \sum_{j=1}^{N_W} |w_j(\mathbf{k})\rangle\langle w_j(\mathbf{k})|, \tag{6.27}$$

which projects onto the vector space spanned by the N_W Wannier band eigenstates $|w_j(\mathbf{k})\rangle$ from SEq. (6.20). By definition, the matrix projector $[\widetilde{P}(\mathbf{k})]$ in SEq. (6.27) is Hermitian, and therefore satisfies

$$[\widetilde{P}(\mathbf{k})]^\dagger = [\widetilde{P}(\mathbf{k})]. \tag{6.28}$$

For an inversion-symmetric system, an isolated grouping of Wannier bands [SEq. (6.26)] centered around an inversion-invariant eigenphase $\gamma_1 \mod \pi = 0$ can be chosen to satisfy

$$[\mathcal{I}][\widetilde{P}(\mathbf{k})][\mathcal{I}]^\dagger = [\widetilde{P}(-\mathbf{k})]. \tag{6.29}$$

Combining SEqs. (6.20), (6.25), and (6.29), such a grouping of N_W Wannier bands [SEq. (6.26)] must satisfy

$$\{\gamma_{1,j}(k_2,k_3)|j=1\ldots N_W\} \bmod 2\pi = \{-\gamma_{1,j}(-k_2,-k_3)|j=1\ldots N_W\} \bmod 2\pi. \tag{6.30}$$

In this work we will always choose $[\widetilde{P}(\mathbf{k})]$ for inversion-symmetric systems such that SEqs. (6.29) and (6.30) hold. Similar to the effective particle-hole symmetry of the entire $\widehat{\mathbf{G}}_1$-directed P-Wannier band structure described in SEq. (6.10), the grouping of N_W $\widehat{\mathbf{G}}_1$-directed P-Wannier bands chosen in a manner that satisfies SEq. (6.29) also has an effective particle-hole symmetry described by SEq. (6.30) [15].

Before we move on, we emphasize that the eigenvectors $|w_j(\mathbf{k})\rangle$ satisfy the boundary condition

$$|w_j(\mathbf{k}+\mathbf{G})\rangle = [V(\mathbf{G})]^{-1}|w_j(\mathbf{k})\rangle = [V(\mathbf{G})]^\dagger|w_j(\mathbf{k})\rangle, \tag{6.31}$$

where $\mathbf{G}$ is a reciprocal lattice vector and $[V(\mathbf{G})]$ is given in SEq. (6.2). To verify SEq. (6.31), we note that under a shift of the base point from $\mathbf{k}$ to $\mathbf{k}+\mathbf{G}$, the $\widehat{\mathbf{G}}_1$-directed P-Wilson loop operator $\mathcal{W}_{1,\mathbf{k},\mathbf{G}_1}$ [SEq. (6.1)] transforms according to

$$\begin{aligned}
\mathcal{W}_{1,\mathbf{k}+\mathbf{G},\mathbf{G}_1} &= [V(\mathbf{G}_1)][P(\mathbf{k}+\mathbf{G}+\mathbf{G}_1)]\cdots[P(\mathbf{k}+\mathbf{G})] \\
&= [V(\mathbf{G}_1)][V(\mathbf{G})]^{-1}[P(\mathbf{k}+\mathbf{G}_1)][V(\mathbf{G})]\cdots[V(\mathbf{G})]^{-1}[P(\mathbf{k})][V(\mathbf{G})] \\
&= [V(\mathbf{G})]^{-1}[V(\mathbf{G}_1)][P(\mathbf{k}+\mathbf{G}_1)]\cdots[P(\mathbf{k})][V(\mathbf{G})] \\
&= [V(\mathbf{G})]^{-1}\mathcal{W}_{1,\mathbf{k},\mathbf{G}_1}[V(\mathbf{G})],
\end{aligned} \tag{6.32}$$

where $[P(\mathbf{k}_f)]\cdots[P(\mathbf{k}_i)]$ is a shorthand notation for the product of projection matrices along a straight path in $\mathbf{k}$-space from $\mathbf{k}_i$ to $\mathbf{k}_f$. In simplifying SEq. (6.32) we have used the boundary condition SEq. (2.12) on the energy eigenvectors $|u_{n,\mathbf{k}}\rangle$, as well as the commutation relation SEq. (4.18) between the matrices $[V(\mathbf{G})]$ and $[V(\mathbf{G}_1)]$. SEq. (6.32) indicates that $\mathcal{W}_{1,\mathbf{k}+\mathbf{G},\mathbf{G}_1}$ and $\mathcal{W}_{1,\mathbf{k},\mathbf{G}_1}$ are related to each other by a similarity transformation, hence implies that their eigenvectors $|w_j(\mathbf{k}+\mathbf{G})\rangle$ and $|w_j(\mathbf{k})\rangle$ can be chosen to satisfy SEq. (6.31).

Using the definition in SEq. (6.27) of the matrix projector $[\widetilde{P}(\mathbf{k})]$ onto the N_W Wannier band eigenfunctions with the eigenphases in SEq. (6.26), we can construct the following $N_{\text{sta}}\times N_{\text{sta}}$ $\widetilde{P}$-Wilson loop operator along a closed loop parallel to $\mathbf{G}_2$,

$$\mathcal{W}_{2,\mathbf{k},\mathbf{G}_1,\mathbf{G}_2} = [V(\mathbf{G}_2)]\lim_{N\to\infty}\left([\widetilde{P}(k_1,k_2+2\pi,k_3)][\widetilde{P}(k_1,k_2+\frac{2\pi(N-1)}{N},k_3)]\cdots[\widetilde{P}(k_1,k_2+\frac{2\pi}{N},k_3)][\widetilde{P}(k_1,k_2,k_3)]\right). \tag{6.33}$$

$\mathcal{W}_{2,\mathbf{k},\mathbf{G}_1,\mathbf{G}_2}$ in SEq. (6.33) is then the nested P-Wilson loop operator constructed by first computing the holonomy along a closed loop parallel to $\mathbf{G}_1$, followed by computing the holonomy along a closed loop parallel to $\mathbf{G}_2$. $\mathcal{W}_{2,\mathbf{k},\mathbf{G}_1,\mathbf{G}_2}$ has $N_{\text{sta}}-N_W$ zero and N_W unimodular eigenvalues that are independent of k_2 [4, 78, 146]. For simplicity, we denote the set of unimodular eigenvalues of $\mathcal{W}_{2,\mathbf{k},\mathbf{G}_1,\mathbf{G}_2}$ as $\{e^{i\gamma_{2,j}(k_1,k_3)}|j=1\ldots N_W\}$ where j is the nested Wannier band index. Under an $\mathcal{I}$ transformation, $\mathcal{W}_{2,\mathbf{k},\mathbf{G}_1,\mathbf{G}_2}$ in SEq. (6.33) transforms according to

$$\begin{aligned}
[\mathcal{I}]\mathcal{W}_{2,\mathbf{k},\mathbf{G}_1,\mathbf{G}_2}[\mathcal{I}]^\dagger &= [\mathcal{I}][V(\mathbf{G}_2)][\widetilde{P}(k_1,k_2+2\pi,k_3)]\cdots[\widetilde{P}(k_1,k_2,k_3)][\mathcal{I}]^\dagger \\
&= [\mathcal{I}][V(\mathbf{G}_2)][\mathcal{I}]^\dagger[\mathcal{I}][\widetilde{P}(k_1,k_2+2\pi,k_3)][\mathcal{I}]^\dagger\cdots[\mathcal{I}][\widetilde{P}(k_1,k_2,k_3)][\mathcal{I}]^\dagger \\
&= [V(-\mathbf{G}_2)][\widetilde{P}(-k_1,-k_2-2\pi,-k_3)]\cdots[\widetilde{P}(-k_1,-k_2,-k_3)] \\
&= [V(-\mathbf{G}_2)][V(-\mathbf{G}_2)]^{-1}[\widetilde{P}(-k_1,-k_2,-k_3)][V(-\mathbf{G}_2)]\cdots[V(-\mathbf{G}_2)]^{-1}[\widetilde{P}(-k_1,-k_2+2\pi,-k_3)][V(-\mathbf{G}_2)] \\
&= [\widetilde{P}(-k_1,-k_2,-k_3)]\cdots[\widetilde{P}(-k_1,-k_2+2\pi,-k_3)][V(-\mathbf{G}_2)] \\
&= \left([V(-\mathbf{G}_2)]^\dagger[\widetilde{P}(-k_1,-k_2+2\pi,-k_3)]\cdots[\widetilde{P}(-k_1,-k_2,-k_3)]\right)^\dagger \\
&= \left([V(\mathbf{G}_2)][\widetilde{P}(-k_1,-k_2+2\pi,-k_3)]\cdots[\widetilde{P}(-k_1,-k_2,-k_3)]\right)^\dagger \\
&= \mathcal{W}^\dagger_{2,-\mathbf{k},\mathbf{G}_1,\mathbf{G}_2},
\end{aligned} \tag{6.34}$$

where $[\widetilde{P}(\mathbf{k}_f)]\cdots[\widetilde{P}(\mathbf{k}_i)]$ represents a product of Wannier band projectors [SEq. (6.27)] along a straight path in $\mathbf{k}$-space from $\mathbf{k}_i$ to $\mathbf{k}_f$. In deriving SEq. (6.34) we have also used SEqs. (6.2), (6.6), (6.27), (6.29), and (6.31), and the unitarity of the matrix $[\mathcal{I}]$. SEq. (6.34) implies that $\mathcal{W}_{2,\mathbf{k},\mathbf{G}_1,\mathbf{G}_2}$ and $\mathcal{W}^\dagger_{2,-\mathbf{k},\mathbf{G}_1,\mathbf{G}_2}$ are isospectral since they are related to each other by a unitary transformation. According to SEq. (6.34) and using the fact that the unimodular eigenvalues of $\mathcal{W}^\dagger_{2,-\mathbf{k},\mathbf{G}_1,\mathbf{G}_2}$ are the complex conjugates of the unimodular eigenvalues of $\mathcal{W}_{2,-\mathbf{k},\mathbf{G}_1,\mathbf{G}_2}$ (see SN 6 J for more details), we have

$$\{e^{i\gamma_{2,j}(k_1,k_3)}|j=1\dots N_W\}=\{e^{-i\gamma_{2,j}(-k_1,-k_3)}|j=1\dots N_W\}. \tag{6.35}$$

In terms of the set of eigenphases $\gamma_{2,j}(k_1,k_3)$, SEq. (6.35) implies that

$$\{\gamma_{2,j}(k_1,k_3)|j=1\dots N_W\} \bmod 2\pi=\{-\gamma_{2,j}(-k_1,-k_3)|j=1\dots N_W\} \bmod 2\pi. \tag{6.36}$$

From the $\mathcal{I}$ constraint in SEq. (6.36), we identify an effective particle-hole symmetry in the set of nested P-Wilson loop eigenphases. A more operational way to interpret SEq. (6.36) is that the nested P-Wannier spectrum $\{\gamma_{2,j}(k_1,k_3)|j=1\dots N_W\}$ is invariant under a simultaneous sign-change of the momenta $(k_1,k_3)\to(-k_1,-k_3)$ and the phase $\gamma_2\to-\gamma_2$. Recall from SN 4 A that the eigenphases [SEq. (6.36)] of the nested P-Wilson loop operator [SEq. (6.33)] correspond to the localized positions of the hybrid Wannier functions formed from a group of P-Wannier bands [SEq. (6.26)] [13, 14]. With this in mind, we can understand SEq. (6.36) as a consequence of the fact that inversion flips both position and momentum of a hybrid Wannier function.

We can also compute the sum over j of the $\gamma_{2,j}(k_1,k_3)$, which defines the nested Berry phase [15, 16, 31]

$$\gamma_2(k_1,k_3)\equiv\sum_{j=1}^{N_W}\gamma_{2,j}(k_1,k_3) \bmod 2\pi, \tag{6.37}$$

where SEq. (6.36) indicates that

$$\gamma_2(k_1,k_3) \bmod 2\pi=-\gamma_2(-k_1,-k_3) \bmod 2\pi. \tag{6.38}$$

Notice that $\mathcal{W}_{2,\mathbf{k},\mathbf{G}_1,\mathbf{G}_2}$ and $\mathcal{W}_{2,\mathbf{k}+\mathbf{G},\mathbf{G}_1,\mathbf{G}_2}$ are isospectral for any reciprocal lattice vector $\mathbf{G}$ since $\mathcal{W}_{2,\mathbf{k},\mathbf{G}_1,\mathbf{G}_2}$ and $\mathcal{W}_{2,\mathbf{k}+\mathbf{G},\mathbf{G}_1,\mathbf{G}_2}$ are related to each other by a similarity transformation:

$$\begin{aligned}\mathcal{W}_{2,\mathbf{k}+\mathbf{G},\mathbf{G}_1,\mathbf{G}_2}&=[V(\mathbf{G}_2)][\widetilde{P}(\mathbf{k}+\mathbf{G}+\mathbf{G}_2)]\cdots[\widetilde{P}(\mathbf{k}+\mathbf{G})]\\&=[V(\mathbf{G}_2)][V(\mathbf{G})]^{-1}[\widetilde{P}(\mathbf{k}+\mathbf{G}_2)]\cdots[\widetilde{P}(\mathbf{k}+\mathbf{G})][V(\mathbf{G})]\\&=[V(\mathbf{G})]^{-1}[V(\mathbf{G}_2)][\widetilde{P}(\mathbf{k}+\mathbf{G}_2)]\cdots[\widetilde{P}(\mathbf{k}+\mathbf{G})][V(\mathbf{G})]\\&=[V(\mathbf{G})]^{-1}\mathcal{W}_{2,\mathbf{k},\mathbf{G}_1,\mathbf{G}_2}[V(\mathbf{G})],\end{aligned} \tag{6.39}$$

where again $[\widetilde{P}(\mathbf{k}_f)]\cdots[\widetilde{P}(\mathbf{k}_i)]$ is a shorthand notation for a product of Wannier band projectors [SEq. (6.27)] along a straight path in $\mathbf{k}$-space from $\mathbf{k}_i$ to $\mathbf{k}_f$, and we have used SEqs. (6.2), (6.27), and (6.31). Therefore, SEq. (6.38) implies that at the four TRIMs $(k_1^{TRIM},k_3^{TRIM})=(0,0)$, $(\pi,0)$, $(0,\pi)$, and (π,π), we have

$$\left(\gamma_2(k_1^{TRIM},k_3^{TRIM}) \mod \pi\right)=0. \tag{6.40}$$

Since inversion symmetry treats all momentum components k_i in the same way—it flips the sign of all the components k_i—the results of this section can be generalized to the nested P-Wilson loop for any primitive reciprocal lattice vectors $\mathbf{G}$ for the direction of the first loop and $\mathbf{G}'$ for the direction of the second loop. The results of SEqs. (6.35), (6.36), (6.38), and (6.40), written in the general notation in SN 4 A, generalize to

$$\{e^{i(\gamma_2)_{j,\mathbf{k},\mathbf{G},\mathbf{G}'}}|j=1\dots N_W\}=\{e^{-i(\gamma_2)_{j,-\mathbf{k},\mathbf{G},\mathbf{G}'}}|j=1\dots N_W\}, \tag{6.41}$$

$$\{(\gamma_2)_{j,\mathbf{k},\mathbf{G},\mathbf{G}'}|j=1\dots N_W\} \bmod 2\pi=\{-(\gamma_2)_{j,-\mathbf{k},\mathbf{G},\mathbf{G}'}|j=1\dots N_W\} \bmod 2\pi, \tag{6.42}$$

$$(\gamma_2)_{\mathbf{k},\mathbf{G},\mathbf{G}'} \bmod 2\pi=-(\gamma_2)_{-\mathbf{k},\mathbf{G},\mathbf{G}'} \bmod 2\pi, \tag{6.43}$$

$$\left((\gamma_2)_{\mathbf{k}_{TRIM},\mathbf{G},\mathbf{G}'} \mod \pi\right)=0, \tag{6.44}$$

where

$$(\gamma_2)_{\mathbf{k},\mathbf{G},\mathbf{G}'}\equiv\sum_{j=1}^{N_W}(\gamma_2)_{j,\mathbf{k},\mathbf{G},\mathbf{G}'} \bmod 2\pi, \tag{6.45}$$

and $(\gamma_2)_{j,\mathbf{k},\mathbf{G},\mathbf{G}'}$ are the eigenphases of the nested P-Wilson loop operator $\mathcal{W}_{2,\mathbf{k},\mathbf{G},\mathbf{G}'}$ satisfying

$$[\mathcal{I}]\mathcal{W}_{2,\mathbf{k},\mathbf{G},\mathbf{G}'}[\mathcal{I}]^\dagger = \mathcal{W}^\dagger_{2,-\mathbf{k},\mathbf{G},\mathbf{G}'}. \tag{6.46}$$

There are two requirements in order for SEq. (6.46) to hold. First, the Hamiltonian must be inversion-symmetric such that SEq. (6.7) holds, which implies that the $\widehat{\mathbf{G}}$-directed P-Wilson loop operator satisfies SEq. (6.19). Second, we must choose an inversion-symmetric grouping of isolated N_W $\widehat{\mathbf{G}}$-directed P-Wannier bands in SEq. (4.23), namely

$$[\mathcal{I}][\widetilde{P}_{\mathbf{G}}(\mathbf{k})][\mathcal{I}]^\dagger = [\widetilde{P}_{\mathbf{G}}(-\mathbf{k})], \tag{6.47}$$

which implies that the chosen N_W $\widehat{\mathbf{G}}$-directed P-Wannier bands satisfy

$$\{(\gamma_1)_{j,\mathbf{k},\mathbf{G}}|j = 1\ldots N_W\} \bmod 2\pi = \{-(\gamma_1)_{j,-\mathbf{k},\mathbf{G}}|j = 1\ldots N_W\} \bmod 2\pi. \tag{6.48}$$

Finally, since $(\gamma_2)_{j,\mathbf{k},\mathbf{G},\mathbf{G}'}$ is independent of the momentum component $\mathbf{k}\cdot\mathbf{a}'$, where $\mathbf{a}'$ is the primitive lattice vector dual to the primitive reciprocal lattice vector $\mathbf{G}'$, $\mathbf{k}_{TRIM}$ in SEq. (6.44) should be interpreted as a $\mathbf{k}$-vector with $(\mathbf{k}_{TRIM}\cdot\mathbf{a}) \bmod \pi = 0$ for each primitive lattice vector $\mathbf{a}\neq\mathbf{a}'$.

C. Antiunitary $\mathcal{T}$ Constraint on the P-Wilson Loop

We will next examine the constraints that antiunitary $\mathcal{T}$ symmetry places on the P-Wilson loop. For concreteness, we will specifically analyze the $\widehat{\mathbf{G}}_1$-directed P-Wilson loop operator $\mathcal{W}_{1,\mathbf{k},\mathbf{G}_1}$ with base point (k_1,k_2,k_3) given in SEq. (6.1), although our results generalize straightforwardly to P-Wilson loops taken along any primitive reciprocal lattice direction. If the Hamiltonian has $\mathcal{T}$ symmetry, then the projection matrix $[P(\mathbf{k})]$ onto the occupied states defined in SEq. (6.3) satisfies

$$[\mathcal{T}][P(\mathbf{k})][\mathcal{T}]^{-1} = [P(-\mathbf{k})], \tag{6.49}$$

where $[\mathcal{T}]$ is the antiunitary representative of $\mathcal{T}$ explicitly constructed in SN 6 L. Note that $[P(\mathbf{k})]$ must project onto a set of energy bands that is mapped into itself under $\mathcal{T}$ in order for SEq. (6.49) to be satisfied. This is guaranteed if $[P(\mathbf{k})]$ projects onto a set of gapped energy bands for a $\mathcal{T}$-symmetric Hamiltonian. Acting with $\mathcal{T}$ on the definition of $\mathcal{W}_{1,\mathbf{k},\mathbf{G}_1}$ [SEq. (6.1)], we find

$$\begin{aligned}
[\mathcal{T}]\mathcal{W}_{1,\mathbf{k},\mathbf{G}_1}[\mathcal{T}]^{-1} &= [\mathcal{T}][V(\mathbf{G}_1)][P(k_1+2\pi,k_2,k_3)]\cdots[P(k_1,k_2,k_3)][\mathcal{T}]^{-1}\\
&= [\mathcal{T}][V(\mathbf{G}_1)][\mathcal{T}]^{-1}[\mathcal{T}][P(k_1+2\pi,k_2,k_3)][\mathcal{T}]^{-1}\cdots[\mathcal{T}][P(k_1,k_2,k_3)][\mathcal{T}]^{-1}\\
&= [V(-\mathbf{G}_1)][P(-k_1-2\pi,-k_2,-k_3)]\cdots[P(-k_1,-k_2,-k_3)]\\
&= [V(-\mathbf{G}_1)][V(-\mathbf{G}_1)]^{-1}[P(-k_1,-k_2,-k_3)][V(-\mathbf{G}_1)]\cdots[V(-\mathbf{G}_1)]^{-1}[P(-k_1+2\pi,-k_2,-k_3)][V(-\mathbf{G}_1)]\\
&= [P(-k_1,-k_2,-k_3)]\cdots[P(-k_1+2\pi,-k_2,-k_3)][V(-\mathbf{G}_1)]\\
&= \left([V(-\mathbf{G}_1)]^\dagger[P(-k_1+2\pi,-k_2,-k_3)]\cdots[P(-k_1,-k_2,-k_3)]\right)^\dagger\\
&= \left([V(\mathbf{G}_1)][P(-k_1+2\pi,-k_2,-k_3)]\cdots[P(-k_1,-k_2,-k_3)]\right)^\dagger\\
&= \mathcal{W}^\dagger_{1,-\mathbf{k},\mathbf{G}_1},
\end{aligned} \tag{6.50}$$

where we have made use of SEqs. (6.4), (6.49), and (6.136) (proved in SN 6 L). While this is formally equivalent to SEq. (6.8), the fact that $\mathcal{T}$ is antiunitary implies that SEq. (6.50) places distinct constraints on the eigenvalues of $\mathcal{W}_{1,\mathbf{k},\mathbf{G}_1}$. In particular, suppose that $|w_j(\mathbf{k})\rangle$ is an eigenvector of $\mathcal{W}_{1,\mathbf{k},\mathbf{G}_1}$ with unimodular eigenvalue $e^{i\gamma_{1,j}(k_2,k_3)}$, such that

$$\mathcal{W}_{1,\mathbf{k},\mathbf{G}_1}|w_j(\mathbf{k})\rangle = e^{i\gamma_{1,j}(k_2,k_3)}|w_j(\mathbf{k})\rangle. \tag{6.51}$$

Acting with $\mathcal{T}$ on both sides of SEq. (6.51) and using SEq. (6.50) we find that

$$[\mathcal{T}]\mathcal{W}_{1,\mathbf{k},\mathbf{G}_1}|w_j(\mathbf{k})\rangle = [\mathcal{T}]e^{i\gamma_{1,j}(k_2,k_3)}|w_j(\mathbf{k})\rangle, \tag{6.52}$$

$$[\mathcal{T}]\mathcal{W}_{1,\mathbf{k},\mathbf{G}_1}[\mathcal{T}]^{-1}[\mathcal{T}]|w_j(\mathbf{k})\rangle = e^{-i\gamma_{1,j}(k_2,k_3)}[\mathcal{T}]|w_j(\mathbf{k})\rangle, \tag{6.53}$$

$$\mathcal{W}^\dagger_{1,-\mathbf{k},\mathbf{G}_1}\left([\mathcal{T}]|w_j(\mathbf{k})\rangle\right) = e^{-i\gamma_{1,j}(k_2,k_3)}\left([\mathcal{T}]|w_j(\mathbf{k})\rangle\right), \tag{6.54}$$

where in going from SEq. (6.52) to SEq. (6.53) we made use of the antiunitarity of $[\mathcal{T}]$. SEq. (6.54) shows that if $|w_j(\mathbf{k})\rangle$ is an eigenvector of $\mathcal{W}_{1,\mathbf{k},\mathbf{G}_1}$ with unimodular eigenvalue $e^{i\gamma_{1,j}(k_2,k_3)}$, then $[\mathcal{T}]\,|w_j(\mathbf{k})\rangle$ is an eigenvector of $\mathcal{W}^\dagger_{1,-\mathbf{k},\mathbf{G}_1}$ with unimodular eigenvalue $e^{-i\gamma_{1,j}(k_2,k_3)}$. Using the fact that the unimodular eigenvalues of $\mathcal{W}^\dagger_{1,-\mathbf{k},\mathbf{G}_1}$ are the complex conjugates of the unimodular eigenvalues of $\mathcal{W}_{1,-\mathbf{k},\mathbf{G}_1}$ (see SN 6 J for more details), we have that the set of unimodular eigenvalues of $\mathcal{W}_{1,\mathbf{k},\mathbf{G}_1}$ satisfies

$$\{e^{i\gamma_{1,j}(k_2,k_3)}|j=1\ldots N_{\text{occ}}\}=\{e^{i\gamma_{1,j}(-k_2,-k_3)}|j=1\ldots N_{\text{occ}}\}. \tag{6.55}$$

In terms of the set of eigenphases $\gamma_{1,j}(k_2,k_3)$, SEq. (6.55) implies

$$\{\gamma_{1,j}(k_2,k_3)|j=1\ldots N_{\text{occ}}\}\bmod 2\pi=\{\gamma_{1,j}(-k_2,-k_3)|j=1\ldots N_{\text{occ}}\}\bmod 2\pi. \tag{6.56}$$

SEq. (6.56) indicates specifically that the P-Wilson loop eigenphase spectrum (*i.e.* the P-Wannier band structure) is invariant under a reversal of the sign of the crystal momentum $(k_2,k_3)\to(-k_2,-k_3)$. Recall from SN 3 A that the eigenphases [SEq. (6.56)] of the P-Wilson loop operator [SEq. (6.1)] correspond to the localized positions of hybrid Wannier functions formed from the set of occupied states [4, 13, 14, 78, 146]. With this in mind, SEq. (6.56) then follows from the fact that time-reversal flips the momentum, but not the position, of a hybrid Wannier function.

We can also examine the $\mathcal{T}$ constraint on the sum over j of the eigenphases $\gamma_{1,j}(k_2,k_3)$, as defined in SEq. (6.11). SEq. (6.56) indicates that

$$\gamma_1(k_2,k_3)\bmod 2\pi=\gamma_1(-k_2,-k_3)\bmod 2\pi. \tag{6.57}$$

Notice that SEq. (6.57) *does not* lead to the quantization of $\gamma_1(k_2,k_3)$ at the four TRIMs $(k_2^{TRIM},k_3^{TRIM})=(0,0)$, $(\pi,0)$, $(0,\pi)$, and (π,π).

As we mentioned at the start of this section (SN 6), the SEqs. (6.50), (6.55), (6.56), and (6.57) hold for both spinless and spinful systems. Specializing now to spinful systems with $\mathcal{T}^2=-1$, we have that SEqs. (6.51)–(6.54), together with the related SEq. (6.62) explained at the beginning of the next section (SN 6 D), imply that the eigenphases $\gamma_{1,j}(k_2,k_3)$ are twofold degenerate at the four TRIMs $(k_2^{TRIM},k_3^{TRIM})=(0,0)$, $(\pi,0)$, $(0,\pi)$, and (π,π). This follows from the periodicity [SEq. (6.32)] of the Wilson loop operator $\mathcal{W}_{1,\mathbf{k},\mathbf{G}_1}$ and the fact that since at TRIMs, namely at $\mathbf{k}=\mathbf{G}/2$ where $\mathbf{G}$ is a reciprocal lattice vector, $|w_j(\mathbf{k})\rangle$ and $[V(\mathbf{G})]^\dagger[\mathcal{T}]\,|w_j(\mathbf{k})\rangle$ are linear combinations of the Bloch states $|u_{n,\mathbf{k}}\rangle$ at the same momentum (where the base point k_1, of which $\gamma_{1,j}(k_2,k_3)$ is independent, is taken to be 0 or π). Additionally, by Kramers' theorem we find that $|w_j(\mathbf{k})\rangle$ and $[V(\mathbf{G})]^\dagger[\mathcal{T}]\,|w_j(\mathbf{k})\rangle$ are orthogonal. Note that we have also used the property that if $[\mathcal{T}]$ is antiunitary and satisfies $[\mathcal{T}]^2=-1$, then $[V(\mathbf{G})]^\dagger[\mathcal{T}]$ is also antiunitary and satisfies $\left([V(\mathbf{G})]^\dagger[\mathcal{T}]\right)^2=-1$, which can be deduced by using SEq. (6.136) proved in SN 6 L. Hence Kramers' theorem can be applied to deduce the orthogonality between $|w_j(\mathbf{k})\rangle$ and $[V(\mathbf{G})]^\dagger[\mathcal{T}]\,|w_j(\mathbf{k})\rangle$ at the BZ boundary TRIMs $\mathbf{k}=\mathbf{G}/2$.

Since time-reversal symmetry treats all momentum components k_i in the same way—it flips the sign of all the components k_i—the results of this section can be generalized to $\widehat{\mathbf{G}}_2$- and $\widehat{\mathbf{G}}_3$-directed P-Wilson loops. The results of SEqs. (6.55), (6.56), and (6.57), written in the general notation in SN 3 A, therefore generalize to

$$\{e^{i(\gamma_1)_{j,\mathbf{k},\mathbf{G}}}|j=1\ldots N_{\text{occ}}\}=\{e^{i(\gamma_1)_{j,-\mathbf{k},\mathbf{G}}}|j=1\ldots N_{\text{occ}}\}, \tag{6.58}$$

$$\{(\gamma_1)_{j,\mathbf{k},\mathbf{G}}|j=1\ldots N_{\text{occ}}\}\bmod 2\pi=\{(\gamma_1)_{j,-\mathbf{k},\mathbf{G}}|j=1\ldots N_{\text{occ}}\}\bmod 2\pi, \tag{6.59}$$

$$(\gamma_1)_{\mathbf{k},\mathbf{G}}\bmod 2\pi=(\gamma_1)_{-\mathbf{k},\mathbf{G}}\bmod 2\pi \tag{6.60}$$

where $(\gamma_1)_{\mathbf{k},\mathbf{G}}$ is defined in SEq. (6.18), and $(\gamma_1)_{j,\mathbf{k},\mathbf{G}}$ are the eigenphases of the $\widehat{\mathbf{G}}$-directed P-Wilson loop operator $\mathcal{W}_{1,\mathbf{k},\mathbf{G}}$ satisfying

$$[\mathcal{T}]\mathcal{W}_{1,\mathbf{k},\mathbf{G}}[\mathcal{T}]^{-1}=\mathcal{W}^\dagger_{1,-\mathbf{k},\mathbf{G}}. \tag{6.61}$$

SEq. (6.61) is true provided that the matrix projector onto the occupied energy bands is time-reversal-symmetric as specified in SEq. (6.49). Finally, since $(\gamma_1)_{j,\mathbf{k},\mathbf{G}}$ is independent of the momentum component $\mathbf{k}\cdot\mathbf{a}$ (where $\mathbf{a}$ is the primitive lattice vector dual to $\mathbf{G}$), Kramers' theorem implies that if $\mathcal{T}^2=-1$ then the eigenphases $(\gamma_1)_{j,\mathbf{k},\mathbf{G}}$ are twofold degenerate at TRIMs $\mathbf{k}_{TRIM}$ satisfying $(\mathbf{k}_{TRIM}\cdot\mathbf{a}')\bmod\pi=0$ for each primitive lattice vector $\mathbf{a}'\neq\mathbf{a}$.

D. Antiunitary $\mathcal{T}$ Constraint on the Nested P-Wilson Loop

We next investigate the constraints that antiunitary $\mathcal{T}$ symmetry places on the nested P-Wilson loop. To begin, we consider the eigenvector $|w_j(\mathbf{k})\rangle$ of the $\widehat{\mathbf{G}}_1$-directed P-Wilson loop operator $\mathcal{W}_{1,\mathbf{k},\mathbf{G}_1}$ with eigenvalue $e^{i\gamma_{1,j}(k_2,k_3)}$

as shown in SEq. (6.20). From SEqs. (6.52)–(6.54) and the surrounding discussion, we know that $[\mathcal{T}]|w_j(\mathbf{k})\rangle$ is an eigenvector of $\mathcal{W}_{1,-\mathbf{k},\mathbf{G}_1}$ with eigenvalue $e^{i\gamma_{1,j}(k_2,k_3)}$, namely

$$\mathcal{W}_{1,-\mathbf{k},\mathbf{G}_1}[\mathcal{T}]|w_j(\mathbf{k})\rangle = e^{i\gamma_{1,j}(k_2,k_3)}[\mathcal{T}]|w_j(\mathbf{k})\rangle. \tag{6.62}$$

According to SEqs. (6.51) and (6.62), there is then a one-to-one correspondence between the eigenvectors of $\mathcal{W}_{1,\mathbf{k},\mathbf{G}_1}$ and $\mathcal{W}_{1,-\mathbf{k},\mathbf{G}_1}$ with unimodular eigenvalues. In particular, if we choose a grouping of N_W P-Wannier bands [denoted as in SEq. (6.26)] that respects the time-reversal symmetry constraint

$$[\mathcal{T}][\widetilde{P}(\mathbf{k})][\mathcal{T}]^{-1} = [\widetilde{P}(-\mathbf{k})], \tag{6.63}$$

where $[\widetilde{P}(\mathbf{k})]$ [defined as in SEq. (6.27)] is the $N_{\text{sta}} \times N_{\text{sta}}$ matrix projector onto the N_W Wannier band eigenstates $|w_j(\mathbf{k})\rangle$, then such a grouping of N_W Wannier bands must satisfy

$$\{\gamma_{1,j}(k_2,k_3)|j = 1\ldots N_W\} \bmod 2\pi = \{\gamma_{1,j}(-k_2,-k_3)|j = 1\ldots N_W\} \bmod 2\pi. \tag{6.64}$$

In this work we will always choose $[\widetilde{P}(\mathbf{k})]$ for time-reversal-symmetric systems in a manner that satisfies SEqs. (6.63) and (6.64).

We now consider the $N_{\text{sta}} \times N_{\text{sta}}$ nested P-Wilson loop operator $\mathcal{W}_{2,\mathbf{k},\mathbf{G}_1,\mathbf{G}_2}$ given in SEq. (6.33). Acting with $\mathcal{T}$, we find that

$$\begin{aligned}
[\mathcal{T}]\mathcal{W}_{2,\mathbf{k},\mathbf{G}_1,\mathbf{G}_2}[\mathcal{T}]^{-1} &= [\mathcal{T}][V(\mathbf{G}_2)][\widetilde{P}(k_1,k_2+2\pi,k_3)]\cdots[\widetilde{P}(k_1,k_2,k_3)][\mathcal{T}]^{-1} \\
&= [\mathcal{T}][V(\mathbf{G}_2)][\mathcal{T}]^{-1}[\mathcal{T}][\widetilde{P}(k_1,k_2+2\pi,k_3)][\mathcal{T}]^{-1}\cdots[\mathcal{T}][\widetilde{P}(k_1,k_2,k_3)][\mathcal{T}]^{-1} \\
&= [V(-\mathbf{G}_2)][\widetilde{P}(-k_1,-k_2-2\pi,-k_3)]\cdots[\widetilde{P}(-k_1,-k_2,-k_3)] \\
&= [V(-\mathbf{G}_2)][V(-\mathbf{G}_2)]^{-1}[\widetilde{P}(-k_1,-k_2,-k_3)][V(-\mathbf{G}_2)]\cdots[V(-\mathbf{G}_2)]^{-1}[\widetilde{P}(-k_1,-k_2+2\pi,-k_3)][V(-\mathbf{G}_2)] \\
&= [\widetilde{P}(-k_1,-k_2,-k_3)]\cdots[\widetilde{P}(-k_1,-k_2+2\pi,-k_3)][V(-\mathbf{G}_2)] \\
&= \Big([V(-\mathbf{G}_2)]^\dagger[\widetilde{P}(-k_1,-k_2+2\pi,-k_3)]\cdots[\widetilde{P}(-k_1,-k_2,-k_3)]\Big)^\dagger \\
&= \Big([V(\mathbf{G}_2)][\widetilde{P}(-k_1,-k_2+2\pi,-k_3)]\cdots[\widetilde{P}(-k_1,-k_2,-k_3)]\Big)^\dagger \\
&= \mathcal{W}^\dagger_{2,-\mathbf{k},\mathbf{G}_1,\mathbf{G}_2},
\end{aligned} \tag{6.65}$$

where we have made use of SEqs. (6.2), (6.27), (6.31), (6.63), and (6.136) (proved in SN 6 L). Following the same logic we used in SEqs. (6.50)–(6.55), SEq. (6.65) implies that the set of unimodular eigenvalues $e^{i\gamma_{2,j}(k_1,k_3)}$ of $\mathcal{W}_{2,\mathbf{k},\mathbf{G}_1,\mathbf{G}_2}$ satisfies

$$\{e^{i\gamma_{2,j}(k_1,k_3)}|j = 1\cdots N_W\} = \{e^{i\gamma_{2,j}(-k_1,-k_3)}|j = 1\cdots N_W\} \tag{6.66}$$

due to $\mathcal{T}$ symmetry. In terms of the set of eigenphases $\gamma_{2,j}(k_1,k_3)$, SEq. (6.66) implies that

$$\{\gamma_{2,j}(k_1,k_3)|j = 1\cdots N_W\} \bmod 2\pi = \{\gamma_{2,j}(-k_1,-k_3)|j = 1\cdots N_W\} \bmod 2\pi. \tag{6.67}$$

SEq. (6.67) specifically implies that the nested P-Wilson loop eigenphase spectrum, *i.e.* the nested P-Wannier band structure, is invariant under a reversal of the sign of the crystal momentum $(k_1,k_3) \to (-k_1,-k_3)$. Recall from SN 4 A that the eigenphases in SEq. (6.67) of the nested P-Wilson loop operator [SEq. (6.33)] correspond to the localized positions of the hybrid Wannier functions formed from a group of P-Wannier bands [denoted as in SEq. (6.26)] [13, 14]. With this in mind, we can understand SEq. (6.67) as a consequence of the fact that time-reversal flips the momentum, but not the position, of a hybrid Wannier function.

We can also examine the $\mathcal{T}$ constraint on the sum over j of the eigenphases $\gamma_{2,j}(k_1,k_3)$, as defined in SEq. (6.37). SEq. (6.67) indicates that

$$\gamma_2(k_1,k_3) \bmod 2\pi = \gamma_2(-k_1,-k_3) \bmod 2\pi. \tag{6.68}$$

Notice that SEq. (6.68) *does not* lead to the quantization of $\gamma_2(k_1,k_3)$ at the four TRIMs $(k_1^{TRIM},k_3^{TRIM}) = (0,0)$, $(\pi,0)$, $(0,\pi)$, and (π,π).

As we mentioned at the start of this section (SN 6), the SEqs. (6.65), (6.66), (6.67), and (6.68) hold for both spinless and spinful systems. Specializing now to spinful systems with $\mathcal{T}^2 = -1$, using SEq. (6.65) and following the same logic as SEqs. (6.51)–(6.54) and (6.62), we have that

$$\mathcal{W}_{2,\mathbf{k},\mathbf{G}_1,\mathbf{G}_2}|w_{2,j}(\mathbf{k})\rangle = e^{i\gamma_{2,j}(k_1,k_3)}|w_{2,j}(\mathbf{k})\rangle, \tag{6.69}$$

$$\mathcal{W}_{2,-\mathbf{k},\mathbf{G}_1,\mathbf{G}_2}[\mathcal{T}]|w_{2,j}(\mathbf{k})\rangle = e^{i\gamma_{2,j}(k_1,k_3)}[\mathcal{T}]|w_{2,j}(\mathbf{k})\rangle, \tag{6.70}$$

where $|w_{2,j}(\mathbf{k})\rangle$ is the eigenstate of $\mathcal{W}_{2,\mathbf{k},\mathbf{G}_1,\mathbf{G}_2}$ with unimodular eigenvalue $e^{i\gamma_{2,j}(k_1,k_3)}$. As in SN 6 C, SEqs. (6.69) and (6.70) imply that the eigenphases $\gamma_{2,j}(k_1,k_3)$ are twofold degenerate at the four TRIMs $(k_1^{TRIM}, k_3^{TRIM}) = (0,0)$, $(\pi,0)$, $(0,\pi)$, and (π,π). This follows from the periodicity [SEq. (6.39)] of the nested Wilson loop and the fact that since at TRIMs, namely at $\mathbf{k} = \mathbf{G}/2$ where $\mathbf{G}$ is a reciprocal lattice vector, the eigenvectors $|w_{2,j}(\mathbf{k})\rangle$ and $[V(\mathbf{G})]^\dagger[\mathcal{T}]\,|w_{2,j}(\mathbf{k})\rangle$ of $\mathcal{W}_{2,\mathbf{k},\mathbf{G}_1,\mathbf{G}_2}$ are linear combinations of Bloch states $|u_{n,\mathbf{k}}\rangle$ at the same momentum (where the base point k_2, of which $\gamma_{2,j}(k_1,k_3)$ is independent, is taken to be 0 or π). Additionally, by Kramers' theorem we find that $|w_{2,j}(\mathbf{k})\rangle$ and $[V(\mathbf{G})]^\dagger[\mathcal{T}]\,|w_{2,j}(\mathbf{k})\rangle$ are orthogonal.

Since time-reversal symmetry treats all momentum components k_i in the same way—it flips the sign of all the components k_i—the results of this section can be generalized to the nested P-Wilson loop for any primitive reciprocal lattice vectors $\mathbf{G}$ for the direction of the first loop and $\mathbf{G}'$ for the direction of the second loop. The results of SEqs. (6.66), (6.67), and (6.68), written in the general notation in SN 4 A, therefore generalize to

$$\{e^{i(\gamma_2)_{j,\mathbf{k},\mathbf{G},\mathbf{G}'}}|j = 1\ldots N_W\} = \{e^{i(\gamma_2)_{j,-\mathbf{k},\mathbf{G},\mathbf{G}'}}|j = 1\ldots N_W\}, \tag{6.71}$$

$$\{(\gamma_2)_{j,\mathbf{k},\mathbf{G},\mathbf{G}'}|j = 1\ldots N_W\} \bmod 2\pi = \{(\gamma_2)_{j,-\mathbf{k},\mathbf{G},\mathbf{G}'}|j = 1\ldots N_W\} \bmod 2\pi, \tag{6.72}$$

$$(\gamma_2)_{\mathbf{k},\mathbf{G},\mathbf{G}'} \bmod 2\pi = (\gamma_2)_{-\mathbf{k},\mathbf{G},\mathbf{G}'} \bmod 2\pi, \tag{6.73}$$

where $(\gamma_2)_{\mathbf{k},\mathbf{G},\mathbf{G}'}$ is defined in SEq. (6.45), and $(\gamma_2)_{j,\mathbf{k},\mathbf{G},\mathbf{G}'}$ are the eigenphases of the nested P-Wilson loop operator $\mathcal{W}_{2,\mathbf{k},\mathbf{G},\mathbf{G}'}$ satisfying

$$[\mathcal{T}]\mathcal{W}_{2,\mathbf{k},\mathbf{G},\mathbf{G}'}[\mathcal{T}]^{-1} = \mathcal{W}^\dagger_{2,-\mathbf{k},\mathbf{G},\mathbf{G}'}. \tag{6.74}$$

SEq. (6.74) is true provided that the projector $[\widetilde{P}_{\mathbf{G}}(\mathbf{k})]$ [SEq. (4.23)] onto the isolated grouping of N_W $\widehat{\mathbf{G}}$-directed P-Wannier bands satisfies the time-reversal-symmetric constraint

$$[\mathcal{T}][\widetilde{P}_{\mathbf{G}}(\mathbf{k})][\mathcal{T}]^{-1} = [\widetilde{P}_{\mathbf{G}}(-\mathbf{k})]. \tag{6.75}$$

Finally, since $(\gamma_2)_{j,\mathbf{k},\mathbf{G},\mathbf{G}'}$ is independent of the momentum component $\mathbf{k}\cdot\mathbf{a}'$ (where $\mathbf{a}'$ is the primitive lattice vector dual to $\mathbf{G}'$), Kramers' theorem implies that if $\mathcal{T}^2 = -1$ then the eigenphases $(\gamma_2)_{j,\mathbf{k},\mathbf{G},\mathbf{G}'}$ are twofold degenerate at TRIMs $\mathbf{k}_{TRIM}$ satisfying $(\mathbf{k}_{TRIM}\cdot\mathbf{a}) \bmod \pi = 0$ for each primitive lattice vector $\mathbf{a} \neq \mathbf{a}'$.

E. Unitary $\mathcal{I}$ Constraint on the $P_\pm$-Wilson Loop

In this section, we will extend the analysis of SN 6 A to investigate the constraints that unitary $\mathcal{I}$ places on the $P_\pm$-Wilson loop (SN 3 B) for a system with spin-1/2 degrees of freedom. We will make the physically relevant assumption that since spin is a $\mathcal{T}$-odd pseudovector, inversion does not act on spin, implying that

$$[\mathcal{I}]s = s[\mathcal{I}], \tag{6.76}$$

where $s = \mathbf{s}\cdot\hat{\mathbf{n}}$ is the spin operator along the direction $\hat{\mathbf{n}}$ and $\mathbf{s} = (s_x, s_y, s_z)$ is given in SEq. (2.13). Acting with inversion on the projected spin operator $[s(\mathbf{k})] \equiv [P(\mathbf{k})]s[P(\mathbf{k})]$ [SEq. (2.14)], we have that for an inversion-symmetric system

$$\begin{aligned}[\mathcal{I}][s(\mathbf{k})][\mathcal{I}]^\dagger &= [\mathcal{I}][P(\mathbf{k})][\mathcal{I}]^\dagger[\mathcal{I}]s[\mathcal{I}]^\dagger[\mathcal{I}][P(\mathbf{k})][\mathcal{I}]^\dagger \\ &= [P(-\mathbf{k})][\mathcal{I}]s[\mathcal{I}]^\dagger[P(-\mathbf{k})] \\ &= [P(-\mathbf{k})]s[P(-\mathbf{k})] \\ &= [s(-\mathbf{k})],\end{aligned} \tag{6.77}$$

where we have used SEqs. (6.7), (6.76), and the unitarity of $[\mathcal{I}]$. Next, we assume that the spin bands, defined as the eigenvalues of $[s(\mathbf{k})]$ with eigenvectors in the image of $[P(\mathbf{k})]$, can be separated into disjoint upper (+) and lower (−) groupings. The corresponding eigenvalue equation for $[s(\mathbf{k})]$ is then

$$[s(\mathbf{k})]|u^\pm_{n,\mathbf{k}}\rangle = \lambda^\pm_{n,\mathbf{k}}|u^\pm_{n,\mathbf{k}}\rangle, \tag{6.78}$$

where the eigenstates $|u^{\pm}_{n,\mathbf{k}}\rangle \in \text{Image}([P(\mathbf{k})])$ of $[s(\mathbf{k})]$ satisfy the boundary condition in SEq. (3.23). We notice that, with only inversion symmetry, the numbers $N^{\pm}_{\text{occ}}$ of upper and lower spin bands are not necessarily the same. For instance, if we consider an inversion-symmetric magnetic system where the electronic ground state contains states with spins nearly aligned along $\hat{\mathbf{n}}$, then the upper and lower spin bands can both have $\lambda^{\pm}_{n\mathbf{k}} > 0$. However, we always have that $N^{+}_{\text{occ}} + N^{-}_{\text{occ}} = N_{\text{occ}}$, the number of occupied electronic energy bands. Using SEqs. (6.77), (6.78), and the assumption that the upper and lower spin bands are disjoint from each other, we can deduce that

$$[\mathcal{I}][P_{\pm}(\mathbf{k})][\mathcal{I}]^{\dagger} = [P_{\pm}(-\mathbf{k})], \tag{6.79}$$

where $[P_{\pm}(\mathbf{k})]$ [defined in SEq. (3.16)] is the projector onto the upper/lower spin bands. From SEq. (6.79), we see that the upper and lower spin bands transform under inversion independently of each other, which is a consequence of SEq. (6.76). Using the projector $[P_{\pm}(\mathbf{k})]$ onto the upper/lower spin bands, the $\widehat{\mathbf{G}}$-directed $P_{\pm}$-Wilson loop operator $\mathcal{W}^{\pm}_{1,\mathbf{k},\mathbf{G}}$ can be constructed as

$$\mathcal{W}^{\pm}_{1,\mathbf{k},\mathbf{G}} = [V(\mathbf{G})] \lim_{N\to\infty} \left([P_{\pm}(\mathbf{k}+\mathbf{G})][P_{\pm}(\mathbf{k}+\frac{N-1}{N}\mathbf{G})]\cdots[P_{\pm}(\mathbf{k}+\frac{1}{N}\mathbf{G})][P_{\pm}(\mathbf{k})] \right) \tag{6.80}$$

along the closed loop parallel to $\mathbf{G}$ analogous to SEq. (6.1). $\mathcal{W}^{\pm}_{1,\mathbf{k},\mathbf{G}}$ has $N_{\text{sta}} - N^{\pm}_{\text{occ}}$ zero eigenvalues corresponding to the number of unoccupied eigenstates. More importantly, $\mathcal{W}^{\pm}_{1,\mathbf{k},\mathbf{G}}$ has $N^{\pm}_{\text{occ}}$ unimodular eigenvalues that are independent of the momentum component $\mathbf{k}\cdot\mathbf{a}$ where $\mathbf{a}$ is the real-space primitive lattice vector dual to the primitive reciprocal lattice vector $\mathbf{G}$ [4, 78, 146]. Using SEqs. (6.79) and (6.80), and following the same logic as SEq. (6.8), we have that $\mathcal{W}^{\pm}_{1,\mathbf{k},\mathbf{G}}$ transforms under inversion according to

$$[\mathcal{I}]\mathcal{W}^{\pm}_{1,\mathbf{k},\mathbf{G}}[\mathcal{I}]^{\dagger} = (\mathcal{W}^{\pm}_{1,-\mathbf{k},\mathbf{G}})^{\dagger}. \tag{6.81}$$

As in SN 6 A, SEq. (6.81) implies the following $\mathcal{I}$-symmetry constraints on the $P_{\pm}$-Wilson loop eigenphases:

$$\{e^{i(\gamma^{\pm}_1)_{j,\mathbf{k},\mathbf{G}}}|j=1\dots N^{\pm}_{\text{occ}}\} = \{e^{-i(\gamma^{\pm}_1)_{j,-\mathbf{k},\mathbf{G}}}|j=1\dots N^{\pm}_{\text{occ}}\}, \tag{6.82}$$

$$\{(\gamma^{\pm}_1)_{j,\mathbf{k},\mathbf{G}}|j=1\dots N^{\pm}_{\text{occ}}\} \bmod 2\pi = \{-(\gamma^{\pm}_1)_{j,-\mathbf{k},\mathbf{G}}|j=1\dots N^{\pm}_{\text{occ}}\} \bmod 2\pi, \tag{6.83}$$

$$(\gamma^{\pm}_1)_{\mathbf{k},\mathbf{G}} \bmod 2\pi = -(\gamma^{\pm}_1)_{-\mathbf{k},\mathbf{G}} \bmod 2\pi, \tag{6.84}$$

$$\left((\gamma^{\pm}_1)_{\mathbf{k}_{TRIM},\mathbf{G}} \mod \pi\right) = 0, \tag{6.85}$$

where

$$(\gamma^{\pm}_1)_{\mathbf{k},\mathbf{G}} \equiv \sum_{j=1}^{N^{\pm}_{\text{occ}}} (\gamma^{\pm}_1)_{j,\mathbf{k},\mathbf{G}} \bmod 2\pi, \tag{6.86}$$

and $(\gamma^{\pm}_1)_{j,\mathbf{k},\mathbf{G}}$ are the eigenphases ($P_{\pm}$-Wannier bands) of the $\widehat{\mathbf{G}}$-directed $P_{\pm}$-Wilson loop operator $\mathcal{W}^{\pm}_{1,\mathbf{k},\mathbf{G}}$ satisfying SEq. (6.81). Recall from SN 3 B that the eigenphases $(\gamma^{\pm}_1)_{j,\mathbf{k},\mathbf{G}}$ of the $P_{\pm}$-Wilson loop operator [SEq. (6.80)] correspond to the localized positions of spin-resolved hybrid Wannier functions formed from the set of upper/lower spin bands [4, 13, 14, 78, 146]. SEq. (6.83) is then a consequence of the fact that inversion flips both the position and momentum, but does not flip the spin, of a spin-resolved hybrid Wannier function. Since $(\gamma^{\pm}_1)_{j,\mathbf{k},\mathbf{G}}$ is independent of the momentum component $\mathbf{k}\cdot\mathbf{a}$, where $\mathbf{a}$ is the real-space primitive lattice vector dual to the primitive reciprocal lattice vector $\mathbf{G}$, $\mathbf{k}_{TRIM}$ in SEq. (6.85) should be interpreted as a $\mathbf{k}$-vector with $(\mathbf{k}_{TRIM}\cdot\mathbf{a}') \mod \pi = 0$ for each primitive lattice vector $\mathbf{a}' \neq \mathbf{a}$.

F. Unitary $\mathcal{I}$ Constraint on the Nested $P_{\pm}$-Wilson Loop

Following SN 6 E, we will in this section extend the analysis in SN 6 B to derive the constraints that unitary $\mathcal{I}$ places on the nested $P_{\pm}$-Wilson loop (SN 4 B) for a system with spin-1/2 degrees of freedom. Since inversion does not act on spin [see SEq. (6.76) and the surrounding text], then the analysis for the nested P_+- and nested P_--Wilson loops are independent of each other. Suppose that the $\widehat{\mathbf{G}}$-directed $P_{\pm}$-Wannier bands ($\{(\gamma^{\pm}_1)_{j,\mathbf{k},\mathbf{G}}|j = 1\dots N^{\pm}_{\text{occ}}\}$) can be separated into disjoint groupings and that we choose an isolated grouping of $N^{\pm}_W$ bands described by $\{(\gamma^{\pm}_1)_{j,\mathbf{k},\mathbf{G}}|j=1\dots N^{\pm}_W\}$ (where $N^{\pm}_W \leq N^{\pm}_{\text{occ}}$) onto which the operator $[\widetilde{P}^{\pm}_{\mathbf{G}}(\mathbf{k})]$ constructed in SEq. (4.46) projects. We now follow the same logic as SN 6 B and focus on either the P_+- or P_--Wannier bands. For an inversion-symmetric

system, the projector $[\widetilde{P}^{\pm}_{\mathbf{G}}(\mathbf{k})]$ onto an isolated grouping of $N^{\pm}_{W}$ $\widehat{\mathbf{G}}$-directed $P_{\pm}$-Wannier bands centered around an inversion-invariant eigenphase $\gamma^{\pm}_{1} \mod \pi = 0$ can be chosen to satisfy

$$[\mathcal{I}][\widetilde{P}^{\pm}_{\mathbf{G}}(\mathbf{k})][\mathcal{I}]^{\dagger} = [\widetilde{P}^{\pm}_{\mathbf{G}}(-\mathbf{k})]. \tag{6.87}$$

SEqs. (6.81) and (6.87) imply that the isolated grouping of $N^{\pm}_{W}$ $\widehat{\mathbf{G}}$-directed $P_{\pm}$-Wilson loop eigenphases satisfies

$$\{(\gamma^{\pm}_{1})_{j,\mathbf{k},\mathbf{G}} | j = 1 \ldots N^{\pm}_{W}\} \mod 2\pi = \{-(\gamma^{\pm}_{1})_{j,-\mathbf{k},\mathbf{G}} | j = 1 \ldots N^{\pm}_{W}\} \mod 2\pi. \tag{6.88}$$

Similar to the effective particle-hole symmetry of the entire $P_{\pm}$-Wannier band structure described in SEq. (6.83), the isolated grouping of $N^{\pm}_{W}$ $P_{\pm}$-Wannier satisfying SEq. (6.87) also has an effective particle-hole symmetry described by SEq. (6.88). From the matrix projector $[\widetilde{P}^{\pm}_{\mathbf{G}}(\mathbf{k})]$, we can construct the $\widetilde{P}^{\pm}_{\mathbf{G}}$-Wilson loop operator along a closed loop parallel to $\mathbf{G}'$ as

$$\mathcal{W}^{\pm}_{2,\mathbf{k},\mathbf{G},\mathbf{G}'} = [V(\mathbf{G}')] \lim_{N\to\infty} \left([\widetilde{P}^{\pm}_{\mathbf{G}}(\mathbf{k}+\mathbf{G}')][\widetilde{P}^{\pm}_{\mathbf{G}}(\mathbf{k}+\frac{N-1}{N}\mathbf{G}')] \cdots [\widetilde{P}^{\pm}_{\mathbf{G}}(\mathbf{k}+\frac{1}{N}\mathbf{G}')][\widetilde{P}^{\pm}_{\mathbf{G}}(\mathbf{k})] \right). \tag{6.89}$$

$\mathcal{W}^{\pm}_{2,\mathbf{k},\mathbf{G},\mathbf{G}'}$ in SEq. (6.89) is the nested $P_{\pm}$-Wilson loop operator with the first and second closed loop parallel to $\mathbf{G}$ and $\mathbf{G}'$ respectively. $\mathcal{W}^{\pm}_{2,\mathbf{k},\mathbf{G},\mathbf{G}'}$ has $N_{\text{sta}} - N^{\pm}_{W}$ zero eigenvalues corresponding to the number of states annihilated by $\widetilde{P}^{\pm}_{\mathbf{G}}(\mathbf{k})$. More importantly, $\mathcal{W}^{\pm}_{2,\mathbf{k},\mathbf{G},\mathbf{G}'}$ has $N^{\pm}_{W}$ unimodular eigenvalues that are independent of the momentum component $\mathbf{k}\cdot\mathbf{a}'$ where $\mathbf{a}'$ is the real-space primitive lattice vector dual to the primitive reciprocal lattice vector $\mathbf{G}'$ [4, 78, 146].

Using SEqs. (6.87) and (6.89), and following the same logic as SEq. (6.34), we have that $\mathcal{W}^{\pm}_{2,\mathbf{k},\mathbf{G},\mathbf{G}'}$ transforms under inversion according to

$$[\mathcal{I}]\mathcal{W}^{\pm}_{2,\mathbf{k},\mathbf{G},\mathbf{G}'}[\mathcal{I}]^{\dagger} = (\mathcal{W}^{\pm}_{2,-\mathbf{k},\mathbf{G},\mathbf{G}'})^{\dagger}. \tag{6.90}$$

As in SN 6 B, SEq. (6.90) allows us to deduce the following constraints on the nested $P_{\pm}$-Wilson loop eigenphases:

$$\{e^{i(\gamma^{\pm}_{2})_{j,\mathbf{k},\mathbf{G},\mathbf{G}'}} | j = 1 \ldots N^{\pm}_{W}\} = \{e^{-i(\gamma^{\pm}_{2})_{j,-\mathbf{k},\mathbf{G},\mathbf{G}'}} | j = 1 \ldots N^{\pm}_{W}\}, \tag{6.91}$$

$$\{(\gamma^{\pm}_{2})_{j,\mathbf{k},\mathbf{G},\mathbf{G}'} | j = 1 \ldots N^{\pm}_{W}\} \mod 2\pi = \{-(\gamma^{\pm}_{2})_{j,-\mathbf{k},\mathbf{G},\mathbf{G}'} | j = 1 \ldots N^{\pm}_{W}\} \mod 2\pi, \tag{6.92}$$

$$(\gamma^{\pm}_{2})_{\mathbf{k},\mathbf{G},\mathbf{G}'} \mod 2\pi = -(\gamma^{\pm}_{2})_{-\mathbf{k},\mathbf{G},\mathbf{G}'} \mod 2\pi, \tag{6.93}$$

$$\left((\gamma^{\pm}_{2})_{\mathbf{k}_{TRIM},\mathbf{G},\mathbf{G}'} \mod \pi\right) = 0, \tag{6.94}$$

where

$$(\gamma^{\pm}_{2})_{\mathbf{k},\mathbf{G},\mathbf{G}'} \equiv \sum_{j=1}^{N^{\pm}_{W}} (\gamma^{\pm}_{2})_{j,\mathbf{k},\mathbf{G},\mathbf{G}'} \mod 2\pi, \tag{6.95}$$

and $(\gamma^{\pm}_{2})_{j,\mathbf{k},\mathbf{G},\mathbf{G}'}$ are the eigenphases (nested $P_{\pm}$-Wannier bands) of the nested $P_{\pm}$-Wilson loop operator $\mathcal{W}^{\pm}_{2,\mathbf{k},\mathbf{G},\mathbf{G}'}$ satisfying SEq. (6.90). Recall from SN 4 B that the eigenphases $(\gamma^{\pm}_{2})_{j,\mathbf{k},\mathbf{G},\mathbf{G}'}$ of the nested $P_{\pm}$-Wilson loop operator [SEq. (6.89)] correspond to the localized positions of the spin-resolved hybrid Wannier functions formed from a group of $P_{\pm}$-Wannier bands [13, 14]. SEq. (6.92) is then a consequence of the fact that inversion flips both position and momentum, but does not flip the spin of a spin-resolved hybrid Wannier function. Since $(\gamma^{\pm}_{2})_{j,\mathbf{k},\mathbf{G},\mathbf{G}'}$ is independent of the momentum component $\mathbf{k}\cdot\mathbf{a}'$, where $\mathbf{a}'$ is the primitive lattice vector dual to the primitive reciprocal lattice vector $\mathbf{G}'$, $\mathbf{k}_{TRIM}$ in SEq. (6.94) should be interpreted as a $\mathbf{k}$-vector with $(\mathbf{k}_{TRIM}\cdot\mathbf{a}) \mod \pi = 0$ for each primitive lattice vector $\mathbf{a} \neq \mathbf{a}'$.

G. Antiunitary $\mathcal{T}$ Constraint on the $P_{\pm}$-Wilson Loop

In this section, we will extend the analysis in SN 6 C to investigate the constraints that spinful antiunitary $\mathcal{T}$ places on the $P_{\pm}$-Wilson loop (SN 3 B) for a system with spin-1/2 degrees of freedom and with $\mathcal{T}^2 = -1$. In the presence of spinful $\mathcal{T}$ symmetry, we denote the $N_{\text{occ}}/2$ spin bands with largest PsP eigenvalue as the upper spin bands; we denote the $N_{\text{occ}}/2$ spin bands with smallest PsP eigenvalue as the lower spin bands. We assume that the upper and lower

spin bands are disjoint from each other [see also SFig. 1(b)]. The projectors $[P_\pm(\mathbf{k})]$ onto the upper (+)/lower (−) spin bands defined in SEq. (3.16) then transforms under spinful $\mathcal{T}$ according to

$$[\mathcal{T}][P_\pm(\mathbf{k})][\mathcal{T}]^{-1} = [P_\mp(-\mathbf{k})]. \tag{6.96}$$

Consequently, the upper and lower PsP eigenvalues [$\lambda^\pm_{n,\mathbf{k}}$ in SEq. (6.78)] are related by

$$\{\lambda^+_{n,\mathbf{k}}|j = 1\dots N^+_{\mathrm{occ}}\} = \{-\lambda^-_{n,-\mathbf{k}}|j = 1\dots N^-_{\mathrm{occ}}\}. \tag{6.97}$$

SEq. (6.96) follows from the eigenvalue equation for $[s(\mathbf{k})]$ in SEq. (6.78), and the fact that time-reversal flips spins, namely

$$[\mathcal{T}]s = -s[\mathcal{T}], \tag{6.98}$$

such that the projected spin operator $[s(\mathbf{k})]$ transforms under spinful $\mathcal{T}$ according to

$$\begin{aligned}[\mathcal{T}][s(\mathbf{k})][\mathcal{T}]^{-1} &= [\mathcal{T}][P(\mathbf{k})][\mathcal{T}]^{-1}[\mathcal{T}]s[\mathcal{T}]^{-1}[\mathcal{T}][P(\mathbf{k})][\mathcal{T}]^{-1} \\ &= [P(-\mathbf{k})][\mathcal{T}]s[\mathcal{T}]^{-1}[P(-\mathbf{k})] \\ &= -[P(-\mathbf{k})]s[P(-\mathbf{k})] \\ &= -[s(-\mathbf{k})],\end{aligned} \tag{6.99}$$

where we have also used SEq. (6.49). Different from the analysis in SN 6 E, we here specialize to the case in which $N^\pm_{\mathrm{occ}} = N_{\mathrm{occ}}/2$, which is required by $\mathcal{T}$ symmetry. Following the same logic as in SEq. (6.50) and using SEq. (6.96), the $P_\pm$-Wilson loop operator [SEq. (6.80)] transforms under spinful $\mathcal{T}$ according to

$$[\mathcal{T}]\mathcal{W}^\pm_{1,\mathbf{k},\mathbf{G}}[\mathcal{T}]^{-1} = (\mathcal{W}^\mp_{1,-\mathbf{k},\mathbf{G}})^\dagger. \tag{6.100}$$

As in SN 6 C, SEq. (6.100) allows us to deduce the following spinful time-reversal constraints on the $P_\pm$-Wilson loop eigenphases:

$$\{e^{i(\gamma_1^\pm)_{j,\mathbf{k},\mathbf{G}}}|j = 1\dots N^\pm_{\mathrm{occ}}\} = \{e^{i(\gamma_1^\mp)_{j,-\mathbf{k},\mathbf{G}}}|j = 1\dots N^\mp_{\mathrm{occ}}\}, \tag{6.101}$$

$$\{(\gamma_1^\pm)_{j,\mathbf{k},\mathbf{G}}|j = 1\dots N^\pm_{\mathrm{occ}}\} \bmod 2\pi = \{(\gamma_1^\mp)_{j,-\mathbf{k},\mathbf{G}}|j = 1\dots N^\mp_{\mathrm{occ}}\} \bmod 2\pi, \tag{6.102}$$

$$(\gamma_1^\pm)_{\mathbf{k},\mathbf{G}} \bmod 2\pi = (\gamma_1^\mp)_{-\mathbf{k},\mathbf{G}} \bmod 2\pi \tag{6.103}$$

where $N^+_{\mathrm{occ}} = N^-_{\mathrm{occ}} = N_{\mathrm{occ}}/2$, $(\gamma_1^\pm)_{\mathbf{k},\mathbf{G}}$ is defined in SEq. (6.86), and $(\gamma_1^\pm)_{j,\mathbf{k},\mathbf{G}}$ are the eigenphases ($P_\pm$-Wannier bands) of the $\hat{\mathbf{G}}$-directed $P_\pm$-Wilson loop operator $\mathcal{W}^\pm_{1,\mathbf{k},\mathbf{G}}$ satisfying SEq. (6.100). Recall from SN 3 B that the eigenphases $(\gamma_1^\pm)_{j,\mathbf{k},\mathbf{G}}$ of the $P_\pm$-Wilson loop operator [SEq. (6.80)] correspond to the localized positions of spin-resolved hybrid Wannier functions formed from the set of upper/lower spin bands [4, 13, 14, 78, 146]. SEq. (6.102) is then a consequence of the fact that spinful time-reversal flips both momentum and spin, but not the position, of a spin-resolved hybrid Wannier function.

H. Antiunitary $\mathcal{T}$ Constraint on the Nested $P_\pm$-Wilson Loop

Following SN 6 G, in this section, we will extend the analysis in SN 6 D to deduce the constraints that antiunitary spinful $\mathcal{T}$ places on the nested $P_\pm$-Wilson loop (SN 4 B) for a system with spin-1/2 degrees of freedom and for which $\mathcal{T}^2 = -1$. For a spinful $\mathcal{T}$-symmetric system, the projector $[\widetilde{P}^\pm_{\mathbf{G}}(\mathbf{k})]$ [SEq. (4.46)] onto an isolated grouping of $N^\pm_W$ $\hat{\mathbf{G}}$-directed $P_\pm$-Wannier bands can be chosen to satisfy

$$[\mathcal{T}][\widetilde{P}^\pm_{\mathbf{G}}(\mathbf{k})][\mathcal{T}]^{-1} = [\widetilde{P}^\mp_{\mathbf{G}}(-\mathbf{k})], \tag{6.104}$$

which combining with SEq. (6.100) implies that the isolated grouping of $N^\pm_W$ $\hat{\mathbf{G}}$-directed $P_\pm$-Wilson loop eigenphases satisfies

$$\{(\gamma_1^\pm)_{j,\mathbf{k},\mathbf{G}}|j = 1\dots N^\pm_W\} \bmod 2\pi = \{(\gamma_1^\mp)_{j,-\mathbf{k},\mathbf{G}}|j = 1\dots N^\mp_W\} \bmod 2\pi, \tag{6.105}$$

which is similar to the constraints imposed by spinful $\mathcal{T}$ on the entire $P_\pm$-Wannier band structure [SEq. (6.102)]. Notice that, as a consequence of enforcing SEqs. (6.104) and (6.105), we have that $N^+_W = N^-_W \le N_{\mathrm{occ}}/2$. Following

the same logic as in SEq. (6.65) and using SEq. (6.104), the nested $P_\pm$-Wilson loop operator [SEq. (6.89)] transforms under spinful $\mathcal{T}$ according to

$$[\mathcal{T}]\mathcal{W}^{\pm}_{2,\mathbf{k},\mathbf{G},\mathbf{G}'}[\mathcal{T}]^{-1} = (\mathcal{W}^{\mp}_{2,-\mathbf{k},\mathbf{G},\mathbf{G}'})^{\dagger}. \tag{6.106}$$

As in SN 6 D, SEq. (6.106) allows us to deduce the following spinful time-reversal constraints on the nested $P_\pm$-Wilson loop eigenphases:

$$\{e^{i(\gamma_2^{\pm})_{j,\mathbf{k},\mathbf{G},\mathbf{G}'}}|j = 1\ldots N_W^{\pm}\} = \{e^{i(\gamma_2^{\mp})_{j,-\mathbf{k},\mathbf{G},\mathbf{G}'}}|j = 1\ldots N_W^{\mp}\}, \tag{6.107}$$

$$\{(\gamma_2^{\pm})_{j,\mathbf{k},\mathbf{G},\mathbf{G}'}|j = 1\ldots N_W^{\pm}\} \bmod 2\pi = \{(\gamma_2^{\mp})_{j,-\mathbf{k},\mathbf{G},\mathbf{G}'}|j = 1\ldots N_W^{\mp}\} \bmod 2\pi, \tag{6.108}$$

$$(\gamma_2^{\pm})_{\mathbf{k},\mathbf{G},\mathbf{G}'} \bmod 2\pi = (\gamma_2^{\mp})_{-\mathbf{k},\mathbf{G},\mathbf{G}'} \bmod 2\pi, \tag{6.109}$$

where $(\gamma_2^{\pm})_{\mathbf{k},\mathbf{G},\mathbf{G}'}$ is defined in SEq. (6.95) and $(\gamma_2^{\pm})_{j,\mathbf{k},\mathbf{G},\mathbf{G}'}$ are the eigenphases (nested $P_\pm$-Wannier bands) of the nested P-Wilson loop operator $\mathcal{W}^{\pm}_{2,\mathbf{k},\mathbf{G},\mathbf{G}'}$ satisfying SEq. (6.106). Recall from SN 4 B that the eigenphases $(\gamma_2^{\pm})_{j,\mathbf{k},\mathbf{G},\mathbf{G}'}$ of the nested $P_\pm$-Wilson loop operator [SEq. (6.89)] correspond to the localized positions of the spin-resolved hybrid Wannier functions formed from a group of $P_\pm$-Wannier bands [13, 14]. SEq. (6.108) is then a consequence of the fact that spinful time-reversal flips both momentum and spin, but not the position, of a spin-resolved hybrid Wannier function.

I. A Summary of Symmetry Constraints on P-, $P_\pm$-, Nested P-, and Nested $P_\pm$-Wilson Loop

In this section, we present two tables [Supplementary Tables 8 and 9] summarizing the symmetry constraints derived in SN 6 A–6 H on the projectors and the eigenphases of the Wilson loops constructed from the product of these projectors along a closed loop. Supplementary Tables 8 and 9 summarize the symmetry constraints from unitary inversion ($\mathcal{I}$) and antiunitary time-reversal ($\mathcal{T}$) symmetries, respectively. The expressions in Supplementary Tables 8 and 9 use the general notation employed in SN 3 A, 3 B, 4 A, and 4 B.

Unitary Inversion ($\mathcal{I}$) Symmetry		
	Projector	Wilson Loop Eigenphases
P-Wilson Loop (SN 6 A)	$[\mathcal{I}][P(\mathbf{k})][\mathcal{I}]^\dagger = [P(-\mathbf{k})]$ [SEq. (6.7)]	$\{(\gamma_1)_{j,\mathbf{k},\mathbf{G}}\|j = 1\ldots N_{\text{occ}}\} \bmod 2\pi$ $= \{-(\gamma_1)_{j,-\mathbf{k},\mathbf{G}}\|j = 1\ldots N_{\text{occ}}\} \bmod 2\pi$ [SEq. (6.15)]
$P_\pm$-Wilson Loop (SN 6 E)	$[\mathcal{I}][P_\pm(\mathbf{k})][\mathcal{I}]^\dagger = [P_\pm(-\mathbf{k})]$ [SEq. (6.79)]	$\{(\gamma_1^{\pm})_{j,\mathbf{k},\mathbf{G}}\|j = 1\ldots N_{\text{occ}}^{\pm}\} \bmod 2\pi$ $= \{-(\gamma_1^{\pm})_{j,-\mathbf{k},\mathbf{G}}\|j = 1\ldots N_{\text{occ}}^{\pm}\} \bmod 2\pi$ [SEq. (6.83)]
Nested P-Wilson Loop (SN 6 B)	$[\mathcal{I}][\widetilde{P}_{\mathbf{G}}(\mathbf{k})][\mathcal{I}]^\dagger = [\widetilde{P}_{\mathbf{G}}(-\mathbf{k})]$ [SEq. (6.47)]	$\{(\gamma_2)_{j,\mathbf{k},\mathbf{G},\mathbf{G}'}\|j = 1\ldots N_W\} \bmod 2\pi$ $= \{-(\gamma_2)_{j,-\mathbf{k},\mathbf{G},\mathbf{G}'}\|j = 1\ldots N_W\} \bmod 2\pi$ [SEq. (6.42)]
Nested $P_\pm$-Wilson Loop (SN 6 F)	$[\mathcal{I}][\widetilde{P}^{\pm}_{\mathbf{G}}(\mathbf{k})][\mathcal{I}]^\dagger = [\widetilde{P}^{\pm}_{\mathbf{G}}(-\mathbf{k})]$ [SEq. (6.87)]	$\{(\gamma_2^{\pm})_{j,\mathbf{k},\mathbf{G},\mathbf{G}'}\|j = 1\ldots N_W^{\pm}\} \bmod 2\pi$ $= \{-(\gamma_2^{\pm})_{j,-\mathbf{k},\mathbf{G},\mathbf{G}'}\|j = 1\ldots N_W^{\pm}\} \bmod 2\pi$ [SEq. (6.92)]

Supplementary Table 8. Constraints from unitary inversion ($\mathcal{I}$) symmetry on the projectors and on the eigenphases of Wilson loops. $[P(\mathbf{k})]$ [SEq. (3.1)] is a projector onto N_{occ} occupied energy bands. $[P_\pm(\mathbf{k})]$ [SEq. (3.16)] is a projector onto $N_{\text{occ}}^{\pm}$ upper/lower spin bands. $[\widetilde{P}_{\mathbf{G}}(\mathbf{k})]$ [SEq. (4.23)] is a projector onto an isolated grouping of N_W $\widehat{\mathbf{G}}$-directed P-Wannier bands. $[\widetilde{P}^{\pm}_{\mathbf{G}}(\mathbf{k})]$ [SEq. (4.46)] is a projector onto an isolated grouping of $N_W^{\pm}$ $\widehat{\mathbf{G}}$-directed $P_\pm$-Wannier bands. $[\mathcal{I}]$ is the unitary matrix representative of $\mathcal{I}$.

Antiunitary Time-Reversal ($\mathcal{T}$) Symmetry		
	Projector	Wilson Loop Eigenphases
P-Wilson Loop (SN 6 C)	$[\mathcal{T}][P(\mathbf{k})][\mathcal{T}]^{-1} = [P(-\mathbf{k})]$ [SEq. (6.49)]	$\{(\gamma_1)_{j,\mathbf{k},\mathbf{G}} \lvert j = 1 \ldots N_{\text{occ}}\} \bmod 2\pi$ $= \{(\gamma_1)_{j,-\mathbf{k},\mathbf{G}} \lvert j = 1 \ldots N_{\text{occ}}\} \bmod 2\pi$ [SEq. (6.59)] (Kramers' degeneracy at TRIMs when $\mathcal{T}^2 = -1$)
$P_{\pm}$-Wilson Loop (SN 6 G)	$[\mathcal{T}][P_{\pm}(\mathbf{k})][\mathcal{T}]^{-1} = [P_{\mp}(-\mathbf{k})]$ [SEq. (6.96)]	$\{(\gamma_1^{\pm})_{j,\mathbf{k},\mathbf{G}} \lvert j = 1 \ldots N_{\text{occ}}^{\pm}\} \bmod 2\pi$ $= \{(\gamma_1^{\mp})_{j,-\mathbf{k},\mathbf{G}} \lvert j = 1 \ldots N_{\text{occ}}^{\mp}\} \bmod 2\pi$ [SEq. (6.102)] (assuming $\mathcal{T}^2 = -1$)
Nested P-Wilson Loop (SN 6 D)	$[\mathcal{T}][\widetilde{P}_{\mathbf{G}}(\mathbf{k})][\mathcal{T}]^{-1} = [\widetilde{P}_{\mathbf{G}}(-\mathbf{k})]$ [SEq. (6.75)]	$\{(\gamma_2)_{j,\mathbf{k},\mathbf{G},\mathbf{G}'} \lvert j = 1 \ldots N_W\} \bmod 2\pi$ $= \{(\gamma_2)_{j,-\mathbf{k},\mathbf{G},\mathbf{G}'} \lvert j = 1 \ldots N_W\} \bmod 2\pi$ [SEq. (6.72)] (Kramers' degeneracy at TRIMs when $\mathcal{T}^2 = -1$)
Nested $P_{\pm}$-Wilson Loop (SN 6 H)	$[\mathcal{T}][\widetilde{P}^{\pm}_{\mathbf{G}}(\mathbf{k})][\mathcal{T}]^{-1} = [\widetilde{P}^{\mp}_{\mathbf{G}}(-\mathbf{k})]$ [SEq. (6.104)]	$\{(\gamma_2^{\pm})_{j,\mathbf{k},\mathbf{G},\mathbf{G}'} \lvert j = 1 \ldots N_W^{\pm}\} \bmod 2\pi$ $= \{(\gamma_2^{\mp})_{j,-\mathbf{k},\mathbf{G},\mathbf{G}'} \lvert j = 1 \ldots N_W^{\mp}\} \bmod 2\pi$ [SEq. (6.108)] (assuming $\mathcal{T}^2 = -1$)

Supplementary Table 9. Constraints from antiunitary time-reversal ($\mathcal{T}$) symmetry on the projectors and on the eigenphases of the Wilson loops. $[P(\mathbf{k})]$ [SEq. (3.1)] is a projector onto N_{occ} occupied energy bands. $[P_{\pm}(\mathbf{k})]$ [SEq. (3.16)] is a projector onto $N_{\text{occ}}^{\pm}$ upper/lower spin bands. $[\widetilde{P}_{\mathbf{G}}(\mathbf{k})]$ [SEq. (4.23)] is a projector onto an isolated grouping of N_W $\widehat{\mathbf{G}}$-directed P-Wannier bands. $[\widetilde{P}^{\pm}_{\mathbf{G}}(\mathbf{k})]$ [SEq. (4.46)] is a projector onto an isolated grouping of $N_W^{\pm}$ $\widehat{\mathbf{G}}$-directed $P_{\pm}$-Wannier bands. $[\mathcal{T}]$ is the antiunitary representative of $\mathcal{T}$.

J. Eigenvalues and Eigenvectors of Wilson Loop Operators and Their Hermitian Conjugates

Suppose an operator $\mathcal{W}$ has spectral decomposition

$$\mathcal{W} = \sum_j e^{i\gamma_j} |w_j\rangle\langle w_j|, \tag{6.110}$$

where $\langle w_j| = (|w_j\rangle)^\dagger$, $\langle w_i|w_j\rangle = \delta_{ij}$, and $\gamma_j \in \mathbb{R}$. For example, all of the Wilson loop operators that we considered in this section (SN 6), including the P-Wilson loop operator [SEq. (6.1)], the nested P-Wilson loop operator [SEq. (6.33)], the $P_{\pm}$-Wilson loop operator [SEq. (6.80)], and the nested $P_{\pm}$-Wilson loop operator [SEq. (6.89)], satisfy SEq. (6.110). Taking the Hermitian conjugate of SEq. (6.110), we obtain

$$\mathcal{W}^\dagger = \sum_j e^{-i\gamma_j} |w_j\rangle\langle w_j|. \tag{6.111}$$

SEqs. (6.110) and (6.111) imply that the eigenvector $|w_j\rangle$ of $\mathcal{W}$ with unimodular eigenvalue $e^{i\gamma_j}$ is also an eigenvector of $\mathcal{W}^\dagger$ with eigenvalue $e^{-i\gamma_j}$. In other words, the sets of unimodular eigenvalues of $\mathcal{W}$ and $\mathcal{W}^\dagger$ are complex conjugates of each other; in addition, $\mathcal{W}$ and $\mathcal{W}^\dagger$ have the same set of orthonormal eigenvectors.

K. Transformation of the $[V(\mathbf{G})]$ Matrix Under Unitary $\mathcal{I}$

In this section, we will derive the representative $[\mathcal{I}]$ of $\mathcal{I}$ in the tight-binding basis states. We will denote by $\mathcal{I}$ the unitary inversion operator that acts on the second-quantized electron operators in SEqs. (2.2) and (2.3). We will use $[\mathcal{I}]$ to denote the $N_{\text{sta}} \times N_{\text{sta}}$ unitary representative of inversion in the tight-binding basis states.

In this section, we will specifically prove that

$$[\mathcal{I}][V(\mathbf{G})][\mathcal{I}]^\dagger = [V(-\mathbf{G})], \tag{6.112}$$

where $[V(\mathbf{G})]$ is the $N_{\text{sta}} \times N_{\text{sta}}$ matrix defined in SEq. (6.2) that encodes the positions of the tight-binding basis states.

Let us consider the creation operators [SEq. (2.2)] for the Bloch basis states

$$c^\dagger_{\mathbf{k},\alpha} = \frac{1}{\sqrt{N}} \sum_{\mathbf{R}} e^{i\mathbf{k}\cdot(\mathbf{R}+\mathbf{r}_\alpha)} c^\dagger_{\mathbf{R},\alpha}. \tag{6.113}$$

Upon a displacement $\mathbf{k} \to \mathbf{k} + \mathbf{G}$ where $\mathbf{G}$ is a reciprocal lattice vector, we have

$$c^\dagger_{\mathbf{k}+\mathbf{G},\alpha} = \frac{1}{\sqrt{N}} \sum_{\mathbf{R}} e^{i(\mathbf{k}+\mathbf{G})\cdot(\mathbf{R}+\mathbf{r}_\alpha)} c^\dagger_{\mathbf{R},\alpha} = e^{i\mathbf{G}\cdot\mathbf{r}_\alpha} \frac{1}{\sqrt{N}} \sum_{\mathbf{R}} e^{i\mathbf{k}\cdot(\mathbf{R}+\mathbf{r}_\alpha)} c^\dagger_{\mathbf{R},\alpha} = e^{i\mathbf{G}\cdot\mathbf{r}_\alpha} c^\dagger_{\mathbf{k},\alpha} \tag{6.114}$$

where we have made use of $e^{i\mathbf{G}\cdot\mathbf{R}} = 1$. Grouping the Bloch basis state creation operators into a creation-operator-valued row vector

$$\psi^\dagger_{\mathbf{k}} = [c^\dagger_{\mathbf{k},1}, \cdots, c^\dagger_{\mathbf{k},N_{\text{sta}}}], \tag{6.115}$$

we have that

$$\psi^\dagger_{\mathbf{k}+\mathbf{G}} = \psi^\dagger_{\mathbf{k}}[V(\mathbf{G})]. \tag{6.116}$$

Next we consider the inversion operation acting on the creation operators of the tight-binding basis states [107]

$$\mathcal{I} c^\dagger_{\mathbf{R},\alpha} \mathcal{I}^\dagger = c^\dagger_{\mathbf{R}',\beta} [\mathcal{I}]_{\beta\alpha} \tag{6.117}$$

where $\mathbf{R}' = -(\mathbf{R} + \mathbf{r}_\alpha) - \mathbf{r}_\beta$ and the β index is implicitly summed over. $[\mathcal{I}]$ in SEq. (6.117) is then the unitary matrix representative of inversion, which can be obtained from the position-space symmetry data of the tight-binding basis states of the system. Notice that if $-(\mathbf{R} + \mathbf{r}_\alpha) - \mathbf{r}_\beta$ is not a linear combination of the Bravais lattice vectors with integral coefficients, we automatically have that the corresponding matrix element $[\mathcal{I}]_{\beta\alpha} = 0$. Using SEqs. (2.2) and (6.117), we can obtain

$$\begin{aligned}
\mathcal{I} c^\dagger_{\mathbf{k},\alpha} \mathcal{I}^\dagger &= \frac{1}{\sqrt{N}} \sum_{\mathbf{R}} e^{i\mathbf{k}\cdot(\mathbf{R}+\mathbf{r}_\alpha)} \mathcal{I} c^\dagger_{\mathbf{R},\alpha} \mathcal{I}^\dagger = \frac{1}{\sqrt{N}} \sum_{\mathbf{R}} e^{i\mathbf{k}\cdot(\mathbf{R}+\mathbf{r}_\alpha)} c^\dagger_{\mathbf{R}',\beta} [\mathcal{I}]_{\beta\alpha} \\
&= \frac{1}{\sqrt{N}} \sum_{\mathbf{R}} e^{i\mathbf{k}\cdot(\mathbf{R}+\mathbf{r}_\alpha)} c^\dagger_{-(\mathbf{R}+\mathbf{r}_\alpha)-\mathbf{r}_\beta,\beta} [\mathcal{I}]_{\beta\alpha} \\
&= \frac{1}{\sqrt{N}} \sum_{\mathbf{R}} e^{i(-\mathbf{k})\cdot(-(\mathbf{R}+\mathbf{r}_\alpha)-\mathbf{r}_\beta+\mathbf{r}_\beta)} c^\dagger_{-(\mathbf{R}+\mathbf{r}_\alpha)-\mathbf{r}_\beta,\beta} [\mathcal{I}]_{\beta\alpha} = \frac{1}{\sqrt{N}} \sum_{\mathbf{R}'} e^{i(-\mathbf{k})\cdot(\mathbf{R}'+\mathbf{r}_\beta)} c^\dagger_{\mathbf{R}',\beta} [\mathcal{I}]_{\beta\alpha} \\
&= c^\dagger_{-\mathbf{k},\beta} [\mathcal{I}]_{\beta\alpha}.
\end{aligned} \tag{6.118}$$

In other words, under inversion symmetry, $\psi^\dagger_{\mathbf{k}}$ in SEq. (6.115) transforms according to

$$\mathcal{I} \psi^\dagger_{\mathbf{k}} \mathcal{I}^\dagger = \psi^\dagger_{-\mathbf{k}} [\mathcal{I}]. \tag{6.119}$$

Now, let us consider the expression

$$\mathcal{I} \psi^\dagger_{\mathbf{k}+\mathbf{G}} \mathcal{I}^\dagger. \tag{6.120}$$

There are two ways we can rewrite SEq. (6.120). First, using SEq. (6.116) and then SEq. (6.119) we can write

$$\begin{aligned}
\mathcal{I} \psi^\dagger_{\mathbf{k}+\mathbf{G}} \mathcal{I}^\dagger &= \mathcal{I} \psi^\dagger_{\mathbf{k}} [V(\mathbf{G})] \mathcal{I}^\dagger \\
&= \mathcal{I} \psi^\dagger_{\mathbf{k}} \mathcal{I}^\dagger [V(\mathbf{G})] \\
&= \psi^\dagger_{-\mathbf{k}} [\mathcal{I}][V(\mathbf{G})],
\end{aligned} \tag{6.121}$$

where, importantly, notice that $\mathcal{I}$ *does not* act on the numerical matrix $[V(\mathbf{G})]$. Second, using SEq. (6.119) and then SEq. (6.116), we can also write SEq. (6.120) as

$$\begin{aligned}
\mathcal{I} \psi^\dagger_{\mathbf{k}+\mathbf{G}} \mathcal{I}^\dagger &= \psi^\dagger_{-\mathbf{k}-\mathbf{G}} [\mathcal{I}] \\
&= \psi^\dagger_{-\mathbf{k}} [V(-\mathbf{G})][\mathcal{I}].
\end{aligned} \tag{6.122}$$

By comparing SEqs. (6.121) and (6.122), we have

$$\psi^\dagger_{-\mathbf{k}} [\mathcal{I}][V(\mathbf{G})] = \psi^\dagger_{-\mathbf{k}} [V(-\mathbf{G})][\mathcal{I}]. \tag{6.123}$$

We next assume that all of the tight-binding basis states are orthogonal to each other. Therefore, SEq. (6.123) can only be satisfied if

$$[\mathcal{I}][V(\mathbf{G})] = [V(-\mathbf{G})][\mathcal{I}]. \tag{6.124}$$

Since $[\mathcal{I}]$ is unitary, we hence conclude that

$$[\mathcal{I}][V(\mathbf{G})][\mathcal{I}]^\dagger = [V(-\mathbf{G})]. \tag{6.125}$$

L. Transformation of the $[V(\mathbf{G})]$ Matrix Under Antiunitary $\mathcal{T}$

In this section, we will derive the transformation of $[V(\mathbf{G})]$ under antiunitary $\mathcal{T}$. As in SN 6 K, we will first define the antiunitary representative $[\mathcal{T}]$ of $\mathcal{T}$. Let us consider the action of $\mathcal{T}$ on the creation operators of the tight-binding basis states [107]

$$\mathcal{T} c^\dagger_{\mathbf{R},\alpha} \mathcal{T}^{-1} = c^\dagger_{\mathbf{R}',\beta} [U_\mathcal{T}]_{\beta\alpha} \tag{6.126}$$

where $\mathbf{R}' = (\mathbf{R} + \mathbf{r}_\alpha) - \mathbf{r}_\beta$, and β is summed over. $[U_\mathcal{T}]$ in SEq. (6.126) is the unitary part of the representative of the antiunitary $\mathcal{T}$. Since $\mathcal{T}$ acts trivially in position space, $[U_\mathcal{T}]_{\beta\alpha}$ has nonzero matrix elements only when $\mathbf{r}_\beta = \mathbf{r}_\alpha$. With SEq. (6.126), we can deduce that the Bloch basis state creation operator [SEq. (2.2)] transforms under $\mathcal{T}$ according to

$$\begin{aligned}
\mathcal{T} c^\dagger_{\mathbf{k},\alpha} \mathcal{T}^{-1} &= \mathcal{T} \frac{1}{\sqrt{N}} \sum_{\mathbf{R}} e^{i\mathbf{k}\cdot(\mathbf{R}+\mathbf{r}_\alpha)} c^\dagger_{\mathbf{R},\alpha} \mathcal{T}^{-1} = \frac{1}{\sqrt{N}} \sum_{\mathbf{R}} e^{-i\mathbf{k}\cdot(\mathbf{R}+\mathbf{r}_\alpha)} \mathcal{T} c^\dagger_{\mathbf{R},\alpha} \mathcal{T}^{-1} = \frac{1}{\sqrt{N}} \sum_{\mathbf{R}} e^{-i\mathbf{k}\cdot(\mathbf{R}+\mathbf{r}_\alpha)} c^\dagger_{\mathbf{R}',\beta} [U_\mathcal{T}]_{\beta\alpha} \\
&= \frac{1}{\sqrt{N}} \sum_{\mathbf{R}} e^{-i\mathbf{k}\cdot(\mathbf{R}+\mathbf{r}_\alpha)} c^\dagger_{\mathbf{R}+\mathbf{r}_\alpha-\mathbf{r}_\beta,\beta} [U_\mathcal{T}]_{\beta\alpha} = \frac{1}{\sqrt{N}} \sum_{\mathbf{R}} e^{-i\mathbf{k}\cdot(\mathbf{R}+\mathbf{r}_\alpha+\mathbf{r}_\beta-\mathbf{r}_\beta)} c^\dagger_{\mathbf{R}+\mathbf{r}_\alpha-\mathbf{r}_\beta,\beta} [U_\mathcal{T}]_{\beta\alpha} \\
&= \frac{1}{\sqrt{N}} \sum_{\mathbf{R}'} e^{-i\mathbf{k}\cdot(\mathbf{R}'+\mathbf{r}_\beta)} c^\dagger_{\mathbf{R}',\beta} [U_\mathcal{T}]_{\beta\alpha} = c^\dagger_{-\mathbf{k},\beta} [U_\mathcal{T}]_{\beta\alpha},
\end{aligned} \tag{6.127}$$

where we have also used the fact that $\mathcal{T}$ is antiunitary. In terms of the creation-operator-valued row vector $\psi^\dagger_\mathbf{k}$ in SEq. (6.115), we then have

$$\mathcal{T} \psi^\dagger_\mathbf{k} \mathcal{T}^{-1} = \psi^\dagger_{-\mathbf{k}} [U_\mathcal{T}]. \tag{6.128}$$

As in SN 6 K, we then consider the expression

$$\mathcal{T} \psi^\dagger_{\mathbf{k}+\mathbf{G}} \mathcal{T}^{-1}. \tag{6.129}$$

First, using SEq. (6.116) and then SEq. (6.128), we can write SEq. (6.129) as

$$\begin{aligned}
\mathcal{T} \psi^\dagger_{\mathbf{k}+\mathbf{G}} \mathcal{T}^{-1} &= \mathcal{T} \psi^\dagger_\mathbf{k} [V(\mathbf{G})] \mathcal{T}^{-1} \\
&= \mathcal{T} \psi^\dagger_\mathbf{k} \mathcal{T}^{-1} \mathcal{T} [V(\mathbf{G})] \mathcal{T}^{-1} \\
&= \mathcal{T} \psi^\dagger_\mathbf{k} \mathcal{T}^{-1} [V(\mathbf{G})]^* \\
&= \psi^\dagger_{-\mathbf{k}} [U_\mathcal{T}] [V(\mathbf{G})]^*.
\end{aligned} \tag{6.130}$$

Notice that in SEq. (6.130) we have also used

$$\mathcal{T} [V(\mathbf{G})] \mathcal{T}^{-1} = [V(\mathbf{G})]^* \tag{6.131}$$

which is a consequence of the antiunitarity of $\mathcal{T}$.

Second, using SEq. (6.128) and then SEq. (6.116), we can also write SEq. (6.129) as

$$\begin{aligned}
\mathcal{T} \psi^\dagger_{\mathbf{k}+\mathbf{G}} \mathcal{T}^{-1} &= \psi^\dagger_{-\mathbf{k}-\mathbf{G}} [U_\mathcal{T}] \\
&= \psi^\dagger_{-\mathbf{k}} [V(-\mathbf{G})] [U_\mathcal{T}].
\end{aligned} \tag{6.132}$$

Comparing SEqs. (6.130) and (6.132), we find that

$$\psi^\dagger_{-\mathbf{k}} [U_\mathcal{T}] [V(\mathbf{G})]^* = \psi^\dagger_{-\mathbf{k}} [V(-\mathbf{G})] [U_\mathcal{T}]. \tag{6.133}$$

As in SN 6 K, we use the orthogonality of the tight-binding basis states to deduce that SEq. (6.133) can only be satisfied if

$$[U_\mathcal{T}] [V(\mathbf{G})]^* = [V(-\mathbf{G})] [U_\mathcal{T}]. \tag{6.134}$$

In the first-quantized matrix formalism, we can introduce the antiunitary representative

$$[\mathcal{T}] = [U_\mathcal{T}] \mathcal{K} \tag{6.135}$$

of $\mathcal{T}$, where $[U_\mathcal{T}]$ is the unitary matrix in SEq. (6.126) that can be obtained from the position-space symmetry data of the tight-binding basis orbitals, and $\mathcal{K}$ is the complex conjugation operator. SEq. (6.135) also implies that $[\mathcal{T}]^{-1} = \mathcal{K} [U_\mathcal{T}]^\dagger$. Using the antiunitary representative $[\mathcal{T}]$ of time-reversal, SEq. (6.134) can then be written as

$$[\mathcal{T}] [V(\mathbf{G})] [\mathcal{T}]^{-1} = [V(-\mathbf{G})]. \tag{6.136}$$

7. BULK SPIN HALL CONDUCTIVITY

In this section, we will use the Kubo formula of SRef. [108] and SEqs. (3.34)–(3.36) to compute the bulk contribution to the spin Hall conductivity in the clean limit for models and materials considered in this work. We start in SN 7 A by reviewing the computational techniques used to evaluate the Kubo formula. Next, in SN 7 B we compute the spin-s_z Hall conductivity for the two-dimensional spin-stable $C_s = 4$ quantum spin Hall insulator introduced in SN 3 G, where in here and SN 7 B we denote the spin Chern number as C_s instead of $C^s_{\gamma_1}$ in SN 3 G, for simplicity. We will pay special attention to how the strength of s_z-conservation-breaking SOC affects the spin Hall conductivity. Next, in SN 7 C we will introduce the *layer*-resolved spin Hall conductivity for three-dimensional systems. We will compute the layer-resolved spin Hall conductivity for the T-DAXI model introduced in SEq. (4.125), and compare it with the layer-resolved spin Chern number computed in SN 5 E.

A. Computational Details

The spin conductivity tensor $\sigma^{s,i}_{\mu\nu}$ parametrizes the linear response of the spin current $\mathbf{J}^{s,i}$ to an applied DC electric field $\mathbf{E}$, via

$$\langle J^{s,i}_{\mu}\rangle = \sum_{\nu} \sigma^{s,i}_{\mu\nu} E_{\nu}. \tag{7.1}$$

Here μ and ν index spatial coordinates (with respect to, *e.g.* a Cartesian basis, as we will elaborate on in Sec. 7 C), and $i = x, y, z$ indexes the spin direction. The spin conductivity can be evaluated using the standard Kubo formula

$$\sigma^{s,i}_{\mu\nu} = \lim_{\epsilon\to 0} \int_0^{\infty} dt \langle \left[J^{s,i}_{\mu}(t), X_{\nu}(0)\right]\rangle e^{-\epsilon t}, \tag{7.2}$$

where X_ν is the ν component of the position operator (which couples to the external electric field in the Hamiltonian), the time-dependence of operators is evaluated in the Heisenberg picture using the unperturbed ($\mathbf{E} = 0$) Hamiltonian H_0, and the average is with respect to the unperturbed ground state. There are two main obstacles to the direct evaluation of SEq. (7.2) for a tight-binding model of a system. First, we must identify the spin current operator $\mathbf{J}^{s,i}$. As proposed in SRefs. [108, 165, 167, 239], we adopt the definition

$$J^{s,i}_{\mu} = \frac{\partial}{\partial t}\left(X_\mu s^i\right) = i\left[H_0, X_\mu s^i\right]. \tag{7.3}$$

Defining the spin current via SEq. (7.3) ensures that the spin conductivity satisfies the Onsager reciprocity relations [167]. This is crucial for relating the spin conductivity to experimental observables, since Onsager reciprocity relates the spin conductivity [SEq. (7.2)] to the inverse spin conductivity that is measured by injecting a spin current into a sample and measuring the induced voltage. The definition [SEq. (7.3)] corresponds to adding to the "conventional spin current" $\mathbf{J}^{s,i}_{\text{conv}} = 1/2\{\mathbf{v}, s^i\}$ the curl-free contributions to the spin torque [165].

Having defined the spin current operator, we can attempt to evaluate SEq. (7.2). One difficulty that arises is that the Kubo formula involves the position operator, both explicitly in SEq. (7.2) and implicitly via the definition [SEq. (7.3)] of the spin current. We must take care then in applying SEq. (7.2) to infinite or periodic systems, where the position operator may not be well-defined. SRef. [108] gives a careful exposition of these issues. In particular, since $X_\nu(0)$ appears in the commutator in SEq. (7.2), only the off-diagonal matrix elements of X_ν, which are well-defined even in infinite or periodic system: the off-diagonal matrix elements of X_ν are expressible in terms of the Berry connection. Thus, when evaluating $\sigma^{s,i}_{\mu\nu}$ using SEq. (7.2) we are free to take our system to be infinite in the ν direction.

More subtle is the position operator appearing in SEq. (7.3) for the spin current. When the i-component of spin is conserved, then SEq. (7.3) only involves off-diagonal components of the position operator X_μ, and we have no issues. However, when s^i is not conserved, $J^{s,i}_{\mu}$ involves both diagonal and off-diagonal matrix elements of X_μ, and hence is not manifestly well-defined for periodic or infinite systems. Nevertheless, SRef. [108] showed that for insulators in the thermodynamic limit, the average in SEq. (7.2) is given by a constant times the number of unit cells of the system, and so can be evaluated by taking a trace over the degrees of freedom in a single unit cell. Thus, we can evaluate the Kubo formula for $\sigma^{s,i}_{\mu\nu}$ when $\mu \neq \nu$ by considering a system finite in the μ-direction (consisting of N_μ unit cells with periodic boundary conditions) and infinite in the ν direction. Provided we choose the origin for X_μ to lie at the center of the finite system, then we recover the bulk spin conductivity for N_μ sufficiently large compared to the inverse bulk energy gap.

Concretely, we will consider d-dimensional tight-binding Hamiltonians defined on a cylinder finite and periodic in the μ direction (with $d = 2$ or 3). The Bloch Hamiltonian for such a system can be written as $H(\mathbf{k})$, where $\mathbf{k}$ is a

$(d-1)$-dimensional vector. The basis states for the Hilbert space can be written as $|R_\mu \mathbf{k}\alpha s\rangle$ where R_μ indexes the unit cells in the X_μ direction, α indexes the orbital degrees of freedom, and s indexes the spin. In this basis the energy eigenstates are $|u_{n\mathbf{k}}\rangle = \sum_{R_\mu \alpha s} u_{R_\mu \alpha s n\mathbf{k}} |R_\mu \mathbf{k}\alpha s\rangle$ with energies $\epsilon_{n\mathbf{k}}$. We can then rewrite SEq. (7.2) for the off-diagonal components of the spin conductivity tensor as

$$\sigma^{s,i}_{\mu\neq\nu} = \frac{i}{(2\pi)^{d-1}} \int d^{d-1}k \sum_{\alpha s} \langle 0\mathbf{k}\alpha s| \left[H(\mathbf{k}), X_\mu s^i\right] \Pi_\nu(\mathbf{k}) |0\mathbf{k}\alpha s\rangle\,, \tag{7.4}$$

where $H(\mathbf{k})$ is the Bloch Hamiltonian and Π_ν is the first-order correction to the projection operator given by

$$\Pi_\nu(\mathbf{k}) = \sum_{\substack{n\in\text{occ}\\ m\in\text{unocc}}} \frac{i}{\epsilon_{m\mathbf{k}} - \epsilon_{n\mathbf{k}}} \left(|u_{n\mathbf{k}}\rangle \langle u_{n\mathbf{k}}|\partial_\nu u_{m\mathbf{k}}\rangle \langle u_{m\mathbf{k}}| + |u_{m\mathbf{k}}\rangle \langle u_{m\mathbf{k}}|\partial_\nu u_{n\mathbf{k}}\rangle \langle u_{n\mathbf{k}}|\right), \tag{7.5}$$

where $\partial_\nu \equiv \partial/\partial k_\nu$. To compute Π_ν numerically, we note that the numerator can be computed in a gauge invariant form,

$$\sum_{\substack{n\in\text{occ}\\ m\in\text{unocc}}} \left(|u_{n\mathbf{k}}\rangle \langle u_{n\mathbf{k}}|\partial_\nu u_{m\mathbf{k}}\rangle \langle u_{m\mathbf{k}}| + |u_{m\mathbf{k}}\rangle \langle u_{m\mathbf{k}}|\partial_\nu u_{n\mathbf{k}}\rangle \langle u_{n\mathbf{k}}|\right) = Q\partial_\nu P P - P\partial_\nu P Q. \tag{7.6}$$

where $P(\mathbf{k})$ is the projector onto the occupied states at $\mathbf{k}$, and $Q(\mathbf{k}) = 1 - P(\mathbf{k})$. SEq. (7.6) can be evaluated using a symmetric finite difference approximation to the derivative ∂_ν. We can then isolate the off-diagonal matrix elements and divide by the energy denominators in SEq. (7.5) to compute Π_ν.

In the subsequent sections, we will apply SEq. (7.4) to compute the spin Hall conductivity for models of spin-stable topological phases in two and three dimensions. We begin by computing the spin Hall conductivity $\sigma^{s,z}_{xy}$ for the fragile two-dimensional topological insulator analyzed in SN 3 G.

B. Spin Hall Conductivity of the 2D Spin-Stable Quantum Spin Hall Insulator

To begin, we consider the square lattice fragile TI with Hamiltonian in SEq. (3.55) and with additional trivial bands coupled via SEq. (3.59). As shown in SN 3 G, this model realizes a spin-stable topological phase with spin-s_z Chern number $C_s = 4$. When the spin-orbit coupling parameter v_{M_z} in SEq. (3.55) is zero, s_z is conserved and the model has two pairs of counterpropagating edge states and a bulk Wilson loop with nonzero helical winding [8]. When $v_{M_z} \neq 0$ however, we showed how the spin Chern number could be computed from the net winding of the spin-resolved Wilson loop (SFig. 10).

The fact that $C_s = 4$ even when $v_{M_z} \neq 0$ suggests that the fragile TI should have a large spin-Hall conductivity even in the absence of spin-s_z conservation. To justify this, we use SEq. (7.4) to compute the spin-s_z Hall conductivity $\sigma^{s,z}_{xy}$ as a function of v_{M_z}. Note that due to the $4mm$ symmetry of the Hamiltonian [SEqs. (3.55) and (3.59)], the spin conductivity tensor satisfies

$$\sigma^{s,z}_{xy} = -\sigma^{s,z}_{yx}. \tag{7.7}$$

This follow from the fact that m_{110} symmetry (which is an element of wallpaper group $p4mm$) maps $x \to -y, y \to -x$, and $s_z \to -s_z$. Applying m_{110} to the Kubo formula [SEq. (7.2)] yields the antisymmetry condition [SEq. (7.7)].

We show the computed spin Hall conductivity for the fragile TI model in SFig. 28. When $v_{M_z} = 0$, we see that the spin Hall conductivity is given by $\sigma^{s,z}_{xy} = e/(4\pi)C_s = 4e/(4\pi)$ as expected from SEq. (3.36). Away from $v_{M_z} = 0$, we see that the spin Hall conductivity increases quadratically as a function of v_{M_z}, consistent with the analysis of SRef. [108] on other two-dimensional topological systems. For weak spin-orbit coupling, we thus expect the spin Hall conductivity to be perturbatively close to the quantized value of $e/(4\pi)C_s$ for spin-stable topological phases in two dimensions.

C. Layer-Resolved Spin Hall Conductivity of T-DAXIs in 3D

Next, we examine the spin Hall conductivity of helical HOTIs in the T-DAXI regime. To do so, let us return to SEq. (7.4) for the off-diagonal spin conductivity tensor. In analogy with Secs. 5 C and 5 E, for a quasi-2D slab we can organize the sum over orbitals α, s in the unit cell in SEq. (7.4) to identify the *layer-resolved* spin conductivity.

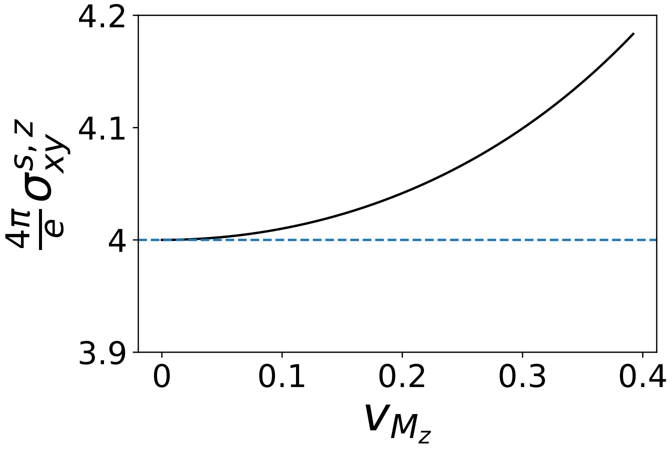

Supplementary Figure 28. Spin-s_z Hall conductivity $\sigma_{xy}^{s,z}$ for the fragile TI model given by SEqs. (3.55) and (3.59), plotted as a function of s_z-nonconserving SOC strength v_{M_z}.

Concretely, we consider a tight-binding model for a three-dimensional system with primitive Bravais lattice vectors $\mathbf{a}_1, \mathbf{a}_2$, and $\mathbf{a}_3$. We consider a two-dimensional slab finite along $\mathbf{a}_3$ with N_3 unit cells. We take there to be $N_{\text{sta}} = 2N_{\text{orb}}$ tight-binding basis states per unit cell. We will compute the spin conductivity in reduced coordinates, defined as

$$\sigma^{s,i}_{a\neq b} = \frac{i}{(2\pi)} \int dk_b \sum_{\alpha s} \langle 0k_b\alpha s| \, [H(k_b), \mathbf{G}_a \cdot \mathbf{X} s^i] \mathbf{G}_b \cdot \Pi(k_b) \, |0k_b\alpha s\rangle \,, \tag{7.8}$$

where $\{\mathbf{G}_a\}$ are the reciprocal lattice vectors dual to $\{\mathbf{a}_a\}$. The sum over α runs over the N_{orb} orbitals in each of the N_3 unit cells, allowing us to write

$$|0k_b\alpha s\rangle = |0k_b n_3 \beta s\rangle \,, \tag{7.9}$$

where $n_3 = 1, \dots N_3$ indexes the unit cell in the $\mathbf{a}_3$ direction of the slab, and $\beta = 1, \dots N_{\text{orb}}$ indexes the orbitals in each unit cell. Inserting this parametrization into SEq. (7.8), we have

$$\sigma^{s,i}_{a\neq b} = \sum_{n_3} \int dk_b \frac{i}{(2\pi)} \sum_{\beta} \langle 0k_b n_3\beta s| \, [H(k_b), \mathbf{G}_a \cdot \mathbf{X} s^i] \mathbf{G}_b \cdot \Pi(k_b) \, |0k_b n_3\beta s\rangle \tag{7.10}$$

$$= \sum_{n_3} \sigma^{s,i}_{a\neq b}(n_3), \tag{7.11}$$

where we have defined the layer-resolved spin conductivity

$$\sigma^{s,i}_{a\neq b}(n_3) = \int dk_b \frac{i}{(2\pi)} \sum_{\beta} \langle 0k_b n_3\beta s| \, [H(k_b), \mathbf{G}_a \cdot \mathbf{X} s^i] \mathbf{G}_b \cdot \Pi(k_b) \, |0k_b n_3\beta s\rangle \,. \tag{7.12}$$

We will focus in particular on the layer-resolved spin Hall conductivity

$$\sigma^{s,i}_{H}(n_3) = \frac{1}{2}\left(\sigma^{s,i}_{12}(n_3) - \sigma^{s,i}_{21}(n_3)\right), \tag{7.13}$$

which gives the contribution of layer n_3 to the spin Hall conductivity

$$\sigma^{s,i}_{H} = \sum_{n_3} \sigma^{s,i}_{H}(n_3) \tag{7.14}$$

of a 2D slab.

Let us now employ SEq. (7.12) to compute the layer-resolved spin Hall conductivity for the model of a symmetry-indicated helical HOTI with inversion and time-reversal symmetry introduced in SRef. [15] and analyzed in SN 4 E and 5 E. In SN 4 E we demonstrated that this model has a spin gap and realizes the T-DAXI spin stable topological phase. In SN 5 E and Supplementary Table 7 we further showed that the two-dimensional surfaces of the model realized an

anomalous odd integer spin Chern number, and we argued that this implied an anomalous topological contribution to the surface spin Hall conductivity. We can now substantiate this argument by computing the layer-resolved spin Hall conductivity for the T-DAXI slab.

We begin by constructing a semi-infinite slab of a T-DAXI. Recall that our model has an orthorhombic lattice. Normalizing the lattice constants to 1, we take the three position-space primitive lattice vectors to be $\mathbf{a}_1 = \hat{x}$, $\mathbf{a}_2 = \hat{y}$, and $\mathbf{a}_3 = \hat{z}$. The dual primitive reciprocal lattice vectors are $\mathbf{G}_1 = 2\pi\hat{x}$, $\mathbf{G}_2 = 2\pi\hat{y}$, and $\mathbf{G}_3 = 2\pi\hat{z}$, satisfying $\mathbf{a}_i \cdot \mathbf{G}_j = 2\pi\delta_{ij}$ $(i, j = 1 \ldots 3)$. Our model has eight bands, for which the matrix Bloch Hamiltonian is given explicitly in SEq. (4.125). The tight-binding parameters, specified in SEq. (4.128), together with spin-non-conserving SOC term [$A_{\text{spin-mixing}}$ in SEq. (4.125)] are chosen such that the energy spectrum of the surfaces with normal vectors $\pm\hat{x}$, $\pm\hat{y}$, and $\pm\hat{z}$ are all gapped. We construct a 2D inversion- and time-reversal-symmetric helical HOTI slab that is finite along $\mathbf{a}_3$ with 15 unit cells and infinite along $\mathbf{a}_1$ and $\mathbf{a}_2$. We then compute the layer-resolved spin-s_z Hall conductivity $\sigma_H^{s,z}(n_3)$ for the helical HOTI slab with both s_z conservation ($A_{\text{spin-mixing}} = 0$) and with large spin non-conserving SOC ($A_{\text{spin-mixing}} = 0.5$).

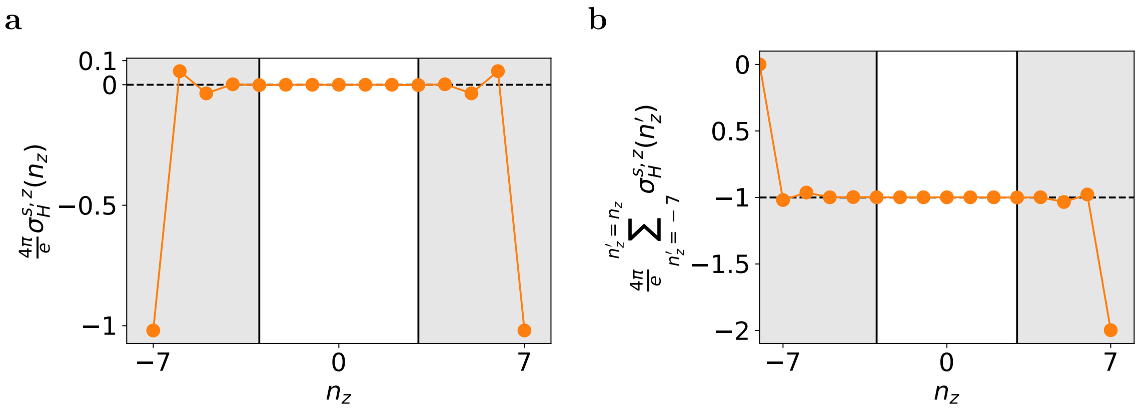

Supplementary Figure 29. Layer-resolved spin Hall conductivity $\sigma_H^{s,z}(n_z)$ of a 2D slab formed from our model of an $\mathcal{I}$- and $\mathcal{T}$-symmetric helical HOTI [SEq. (4.125)] in the T-DAXI regime (see SN 4 E) with spin s_z conservation ($A_{\text{spin-mixing}} = 0.0$). (a) shows the layer-resolved spin Hall conductivity $\sigma_H^{s,z}(n_z)$ for a 2D slab finite along z with 15 unit cells and infinite along x and y. (b) shows the cumulative spin Hall conductivity $\sum_{n_z'=-7}^{n_z'=n_z}\sigma_H^{s,z}(n_z')$ as a function of n_z beginning from the bottom layer in (a). As we can see in (a), the nonzero values of $\sigma_H^{s,z}(n_z)$ are concentrated around the top and bottom layers. In addition, (b) demonstrates the appearance of anomalous odd-integer spin Hall conductivity (in units of $e/(4\pi)$) around the gapped surfaces of the T-DAXI, as the cumulative values of $\sigma_H^{s,z}(n_z)$ beginning from the bottom layer quickly converge to $-e/4\pi$ and remain constant in the bulk.

SFig. 29 shows the layer-resolved spin Hall conductivity in the s_z-conserving limit $A_{\text{spin-mixing}} = 0$, in units of $e/(4\pi)$. In this limit, the spin Hall conductivity is given entirely by the topological contribution defined in SEq. (3.36). We see from SFig. 29(a) that the layer-resolved spin Hall conductivity is zero deep in the bulk of the slab and nonzero near the surfaces. This is consistent with the spin-resolved layer construction of the T-DAXI presented in SN 4 D 2. In SFig. 29(b) we show the cumulative spin Hall conductivity $\sum_{n_z'=-7}^{n_z'=n_z}\sigma_H^{s,z}(n_z)$. Summing over the five layers closest to the surface, we find that each surface contributes

$$\sigma_{H,\text{surface}}^{s,z} = \sum_{n_z'=-7}^{n_z'=-3}\sigma_H^{s,z}(n_z) = -0.999e/(4\pi) \approx (-1)e/(4\pi) \tag{7.15}$$

to the spin Hall conductivity. This is consistent with our computation in SN 5 E, where we showed that the surface spin-s_z Chern number for this helical HOTI slab is given by $C_{xy}^{+} - C_{xy}^{-} = -1$. We thus see that in the s_z-conserving limit, the odd-integer surface spin Chern number of the T-DAXI implies a quantized odd-integer (in units of $e/(4\pi)$) surface spin Hall conductivity. The total spin Hall conductivity $\sigma_H^{s,z} = \sum_{n_z'=-7}^{n_z'=7}\sigma_H^{s,z}(n_z) = (-2)e/(4\pi)$, consistent with the partial Chern number of the T-DAXI slab given in Supplementary Table 7. In comparing SFig. 29 for the layer-resolved spin Hall conductivity with SFig. 27 for the layer-resolved partial Chern number, we see that although the cumulative spin Hall conductivity for the top surface is proportional to the surface spin Chern number, the layer-resolved spin Hall conductivity is not proportional to the layer-resolved spin Chern number even in this spin-conserving limit. In particular, we see from SFig. 29 that the layer-resolved spin Hall conductivity oscillates between

positive and negative values near each surface, while the layer-resolved spin Chern number in SFig. 27 decreases monotonically to zero near each surface. To understand this discrepancy, we recall from SEqs. (3.34)–(3.36) that the spin Hall conductivity can be written as the sum of the spin Chern number and a correction term σ^s_{II}. Although σ^s_{II} vanishes in the s_z-conserving limit when summed over all layers, it still gives a non-vanishing contribution to the layer-resolved spin Hall conductivity. Physically, this is because each layer of the slab is not an isolated 2D system: s_z-per layer is not a good quantum number since electrons can hop into and out of each layer. Nevertheless, we see that the surface contribution to the spin Hall conductivity (summed over the layers near the surface) are given by $e/(4\pi)$ times the surface contribution to the spin Chern number [SEq. (5.28)].

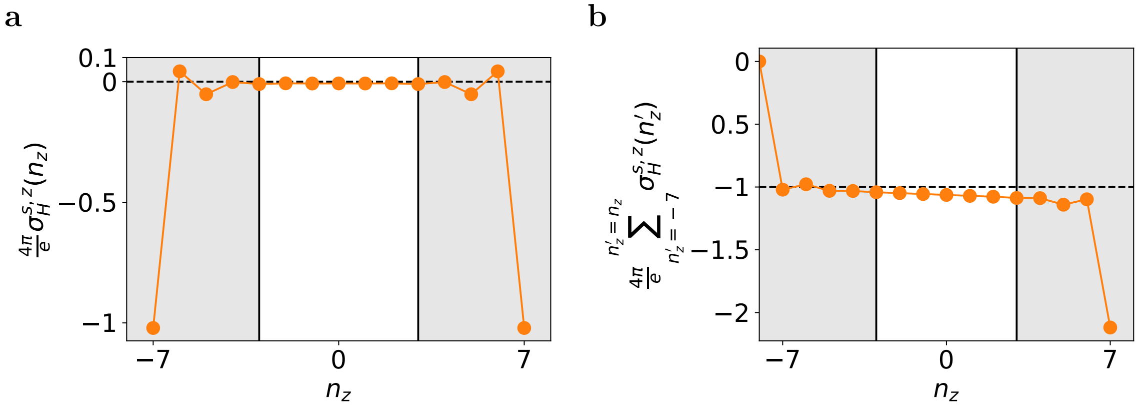

Supplementary Figure 30. Layer-resolved spin Hall conductivity $\sigma_H^{s,z}(n_z)$ of a 2D slab formed from our model of an $\mathcal{I}$- and $\mathcal{T}$-symmetric helical HOTI [SEq. (4.125)] in the T-DAXI regime (see SN 4 E) with strong s_z non-conserving SOC ($A_{\text{spin-mixing}} = 0.5$). (a) shows the layer-resolved spin Hall conductivity $\sigma_H^{s,z}(n_z)$ for a 2D slab finite along z with 15 unit cells and infinite along x and y. (b) shows the cumulative spin Hall conductivity $\sum_{n_z'=-7}^{n_z'=n_z}\sigma_H^{s,z}(n_z')$ as a function of n_z beginning from the bottom layer in (a). As we can see in (a), the nonzero values of $\sigma_H^{s,z}(n_z)$ are largely concentrated around the top and bottom layers, although the spin Hall conductivity remains small but nonzero in the bulk of the system. This becomes clearer in (b), where we see both large anomalous topological contributions to the spin Hall conductivity around the gapped surfaces of the T-DAXI, as well as a residual small nontopological contribution from the bulk of the system.

When $A_{\text{spin-mixing}} = 0.5$, s_z is no longer conserved, and so we expect the spin Hall conductivity to receive both topological and non-topological contributions per SEqs. (3.34)–(3.36). In SFig. 30 we show the layer-resolved spin Hall conductivity $\sigma_H^{s,z}(n_z)$ [panel (a)] and the layer-summed cumulative spin Hall conductivity $\sum_{n_z'=-7}^{n_z'=n_z}\sigma_H^{s,z}(n_z)$ [panel (b)] For the T-DAXI model with $A_{\text{spin-mixing}} = 0.5$. Since s_z is no longer conserved, the bulk spin Hall conductivity is no longer zero, but receives small corrections; we find that $\sigma_H^{s,z}(n_z = 0) \approx -0.007e/(4\pi)$ in the center of the slab. Similarly, the total spin Hall conductivity of the slab deviates from the spin Chern number, and is given by $\sigma_H^{s,z} = \sum_{n_z'=-7}^{n_z'=7}\sigma_H^{s,z}(n_z) \approx -2.11e/(4\pi)$. Nevertheless, we see from SFig. 30(a) that the largest contributions to the spin Hall conductivity come from the anomalous gapped surfaces. Summing over the five layers closest to the surface, we find that the surface contribution to the spin Hall conductivity is

$$\sigma^{s,z}_{H,\text{surface}} = \sum_{n_z'=-7}^{n_z'=-3}\sigma_H^{s,z}(n_z) = -1.042e/(4\pi). \tag{7.16}$$

8. SPIN-RESOLVED TOPOLOGY IN REAL MATERIALS

Importantly, the spin-resolved Wilson loop and nested spin-resolved Wilson loop formalisms developed in SN 3 and 4 are applicable beyond toy models, and can be applied without modification to deduce the spin-resolved topology of real materials. Additionally, when there is a spin gap, the spin-resolved topology can then be compared to the intrinsic bulk contribution to the spin Hall conductivity, which can be computed through the Kubo formula as shown in SN 7. Below, in SN 9 and 10, we will present detailed analyses of the spin-resolved topology and physical observables for two experimentally accessible candidate helical HOTI materials: β-MoTe$_2$ [ICSD 14349, space group (SG) $P2_1/m1'$ (#11.51) in magnetic (Shubnikov) notation, SG 11 ($P2_1/m$) in nonmagnetic notation] [31, 43, 50] and α-BiBr [ICSD 1560, SG $C2/m1'$ (#12.59) in magnetic notation, SG 12 ($C2/m$) in nonmagnetic notation] [50, 52, 53, 64, 68, 240].

In the remainder of this section, we will briefly summarize our application of spin-resolved topological analysis to real materials and numerical demonstration of the associated physical signatures of nontrivial spin-resolved topology; complete details are provided below in SN 9 and 10.

First, in SN 9 B, we will show that β-$MoTe_2$ realizes a spin-Weyl state (SN 4 D 3) for all choices of the spin resolution direction. We will then demonstrate in SN 9 C that β-$MoTe_2$, when subjected to a large external Zeeman field, exhibits (001)-surface topological Fermi arcs originating from bulk spin-Weyl points (see SN 2 G for the underlying theoretical details). Next, in SN 10 B, we will show that α-BiBr remarkably exhibits a large spin gap across a significant range of spin resolution directions, and specifically hosts both spin-stable 3D QSHI and T-DAXI states (SN 4 D 2 and 4 D 3). Lastly, in SN 10 C, we will numerically demonstrate that the spin-gapped 3D QSHI and T-DAXI states in α-BiBr respectively carry nearly quantized and nearly vanishing bulk spin Hall conductivities per unit cell, respectively (see SN 7).

In our topological analysis of real materials, we will work primarily with Wannier-based approximate tight-binding models. We will compute (spin-resolved) Wilson loops and transport coefficients in terms of the tight-binding eigenstates. We take care to note that in doing so, we will in our analysis ignore contributions to Wilson loops and transport coefficients arising from the finite extent and spatial profile of the Wannier functions themselves. In particular, Wilson loops computed using tight-binding eigenstates can have a different eigenspectrum than Wilson loops computed using exact Bloch eigenstates due to off-diagonal matrix elements of the position operator in the Wannier-function basis [4, 172]. Similarly, off-diagonal matrix elements of the position operator in the Wannier-function basis lead to modifications of the current and spin-current operators that can alter the numerical value of computed transport coefficients [241]. Since the discrepancy between tight-binding and ab-initio derived quantities originates from the spatial extent of the Wannier functions, we expect that the discrepancies will be exponentially small provided that the Wannier functions are well-localized. In particular, for a model with exponentially localized Wannier functions that are spin eigenstates, the (partial) Chern numbers computed from the tight-binding eigenstates will coincide with the (partial) Chern numbers computed from the full ab-initio wave functions. In our analysis of real materials detailed below, we have taken care to include sufficient bands in the tight-binding Hilbert space to ensure that the Wannier functions of our tight-binding model are symmetric, exponentially localized, and spin eigenstates. As such, we expect that the (partial) Chern numbers that we obtain from Wannier-based tight-binding calculations accurately reflect the band topology of the ab-initio wave functions, and we similarly expect our tight-binding-derived numerical calculation of the spin Hall conductivity to be a good approximation of the intrinsic spin Hall conductivity of real materials (here α-BiBr).

9. FIRST-PRINCIPLES ANALYSIS OF β-$MoTe_2$

In this section, we will compute the spin-resolved topology of 3D β-phase $MoTe_2$, which was identified in SRefs. [31, 50] as a helical HOTI with both inversion ($\mathcal{I}$) and time-reversal ($\mathcal{T}$) symmetries (specifically characterized by a nontrivial $\mathbb{Z}_4$-invariant $z_4 = 2$ and vanishing weak indices $z_{2i} = 0$).

Below, we will begin our spin-resolved topological analysis of 3D β-$MoTe_2$ by detailing in SN 9 A the density-functional-theory (DFT) calculations that we employed to obtain a symmetric, Wannier-based tight-binding model of β-$MoTe_2$. In SN 9 B, we will then show that 3D β-$MoTe_2$ generically lies in the spin-Weyl regime of a helical HOTI [SN 4 D 3] for all choices of spin direction by computing the spectrum of the projected spin operator [SEq. (2.14)] and spin-resolved Wilson loops [SN 3 B]. Finally in SN 9 C, we will demonstrate the appearance of (001)-surface topological Fermi arcs in β-$MoTe_2$ subjected to a strong (spin-) Zeeman field, which represent a physical signature of its bulk spin-Weyl points (see SN 2 G).

A. Details of Density Functional Theory Calculations on β-$MoTe_2$

The 3D β phase of $MoTe_2$ crystallizes a centrosymmetric structure that respects the symmetries of nonmagnetic SG $P2_1/m1'$ (#11.51). Each primitive (unit) cell of β-$MoTe_2$ contains four Mo atoms and eight Te atoms [SFig. 31(a)]. The primitive Bravais lattice vectors of β-$MoTe_2$ are given by

$$\begin{aligned}
\mathbf{a}_1 &= \mathbf{a} = (6.3299999\ \text{Å})\hat{\mathbf{x}} = a\hat{\mathbf{x}}, \\
\mathbf{a}_2 &= \mathbf{b} = (3.4690001\ \text{Å})\hat{\mathbf{y}} = b\hat{\mathbf{y}}, \\
\mathbf{a}_3 &= \mathbf{c} = -(0.9467946\ \text{Å})\hat{\mathbf{x}} + (13.8276235\ \text{Å})\hat{\mathbf{z}} = c\cos\beta\hat{\mathbf{x}} + c\sin\beta\hat{\mathbf{z}},
\end{aligned} \tag{9.1}$$

where a, b, and c denote the conventional-cell lattice parameters [242]

$$a = |\mathbf{a}| = 6.3299999\ \text{Å},\ \ b = |\mathbf{b}| = 3.4690001\ \text{Å},\ \ c = |\mathbf{c}| = 13.8599997\ \text{Å}, \tag{9.2}$$

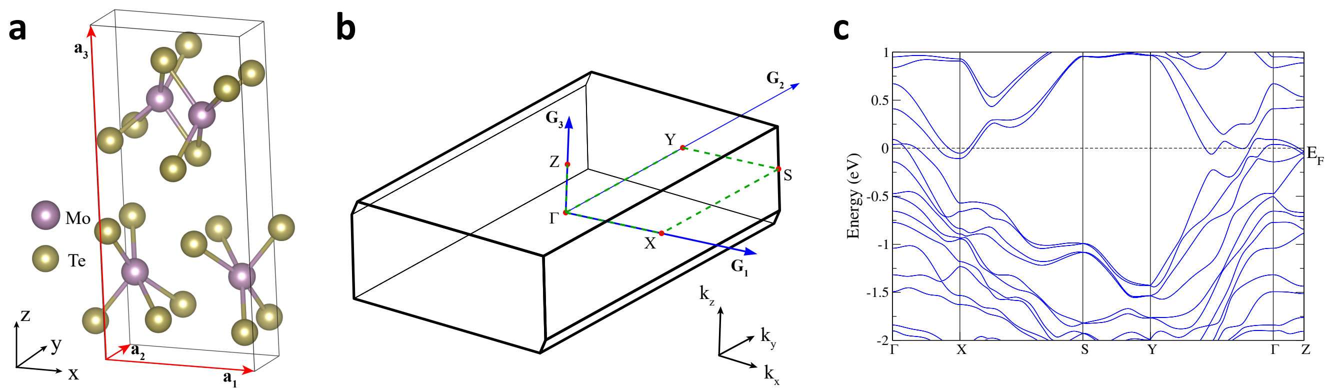

Supplementary Figure 31. First-principles electronic structure of β-$MoTe_2$. (a) The crystal structure of β-$MoTe_2$, which respects the symmetries of space group (SG) $P2_1/m1'$ (#11.51) [242] . There are four Mo atoms and eight Te atoms in each primitive (unit) cell. In (a), the red (black) arrows denote the primitive-cell lattice (Cartesian-unit) vectors [SEq. (9.1)]. (b) The bulk Brillouin zone (BZ) for β-$MoTe_2$; the primitive reciprocal lattice vectors $\mathbf{G}_i$ (blue arrows) are given in SEq. (9.3), and notably differ from the Cartesian reciprocal unit vectors (black arrows). In (b), the time-reversal-invariant $\mathbf{k}$ (TRIM) points are labeled for consistency using the convention previously employed in SRef. [31], which we note differs from the labeling convention for SG $P2_1/m1'$ (#11.51) on the Bilbao Crystallographic Server [243–245]. (c) The first-principles- (DFT-) obtained electronic structure of β-$MoTe_2$ plotted along the green dashed $\mathbf{k}$-path in (b). The Fermi energy is denoted as E_F.

such that for β-$MoTe_2$, a, b, and c respectively coincide with $|\mathbf{a}_1|$, $|\mathbf{a}_2|$, and $|\mathbf{a}_3|$, because nonmagnetic SG $P2_1/m1'$ (#11.51) is primitive (P) monoclinic. In SEq. (9.1), $\beta \approx 93.91699963419269°$ is the angle between $\mathbf{a}_1$ and $\mathbf{a}_3$. The primitive reciprocal lattice vectors [SFig. 31(b)] for β-$MoTe_2$ are correspondingly given by

$$\begin{aligned} \mathbf{G}_1 &= \frac{2\pi}{a}\left(\hat{\mathbf{x}} - \cot\beta\hat{\mathbf{z}}\right), \\ \mathbf{G}_2 &= \frac{2\pi}{b}\hat{\mathbf{y}}, \\ \mathbf{G}_3 &= \frac{2\pi}{c}\csc\beta\hat{\mathbf{z}}. \end{aligned} \tag{9.3}$$

We use ab-initio (DFT) calculations incorporating the effects of SOC to compute the electronic band structure of β-$MoTe_2$. Our first-principles calculations were specifically performed within the DFT framework using the projector-augmented wave (PAW) method [246, 247] as implemented in the Vienna ab-initio simulation package (VASP) [248, 249]. In our first-principles calculations, we adopted the Perdew-Burke-Ernzerhof (PBE) generalized gradient approximation exchange-correlation functional [250], and SOC was incorporated self-consistently. The cutoff energy for the plane-wave expansion was 400 eV, and $0.03 \times 2\pi$ Å^{-1} $\mathbf{k}$-point sampling grids were used in the self-consistent process. In SFig. 31(c) we show the ab-initio band structure for β-$MoTe_2$ computed along high-symmetry lines. We note that throughout this work, high-symmetry BZ (TRIM) points in the bulk electronic and spin spectra of β-$MoTe_2$ are labeled for consistency using the convention previously employed in SRef. [31]. The TRIM point labels for β-$MoTe_2$ in this work hence differ from the standard labels for SG $P2_1/m1'$ (#11.51) listed on the Bilbao Crystallographic Server [43, 243–245, 251–253].

Next, to analyze the spin-resolved band topology, we constructed a symmetric, Wannier-based tight-binding model fit to the electronic structure of β-$MoTe_2$ obtained from our DFT calculations. We constructed symmetric Wannier functions for the bands near the Fermi energy E_F in β-$MoTe_2$ by using the Wannier90 package [254] for the Mo $4d$ and the Te $5p$ orbitals, and then performing a subsequent SG symmetrization using WannierTools [255]. For the following discussion, we denote the Hamiltonian of the Wannier-based tight-binding model as $[H_{\mathrm{MoTe}_2}]$. The single-particle Hilbert space of $[H_{\mathrm{MoTe}_2}]$ consists of 44 spinful Wannier functions per unit cell; the Bloch Hamiltonian $[H_{\mathrm{MoTe}_2}(\mathbf{k})]$ is therefore an 88×88 matrix. To reduce the computational resources required for our spin-resolved and Wilson loop calculations, we next truncated $[H_{\mathrm{MoTe}_2}]$ to only contain hopping terms with an absolute magnitude greater than or equal to 0.001eV. We have confirmed that this truncation affects neither the band ordering nor the qualitative features of the band structure near the Fermi energy. Specifically in SFig. 32(a,b), we show the band structure of β-$MoTe_2$ computed from the Wannier-based tight-binding model $[H_{\mathrm{MoTe}_2}]$, which shows good agreement with the DFT-obtained band structure in SFig. 31(c), demonstrating that our truncated Wannier-based tight-binding model well-approximates the electronic structure of β-$MoTe_2$. In both the DFT-obtained and Wannier tight-binding band structures, there is an avoided crossing between the valence and conduction bands along the $\Gamma - Y$ line [enlarged view

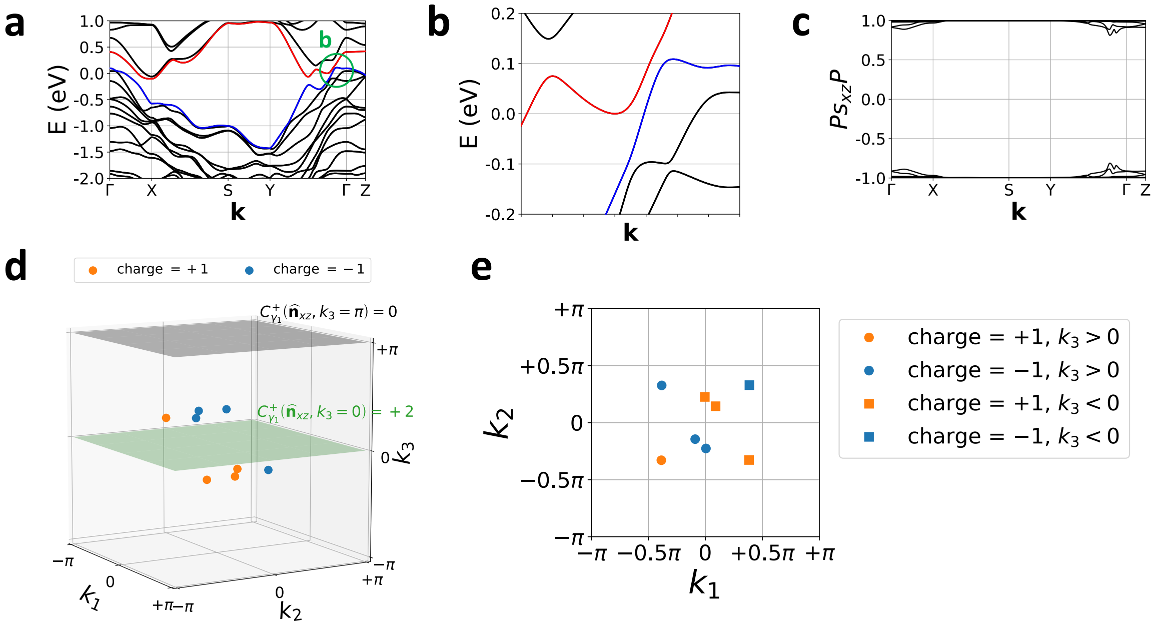

Supplementary Figure 32. Energy and spin spectrum for the ab-initio-derived symmetric Wannier-based tight-binding model of β-MoTe$_2$. (a) shows the band structure for our Wannier-based tight-binding model along high-symmetry lines in the BZ. The high-symmetry BZ (TRIM) points are labeled employing the convention in SFig. 31(c), which follows the notation employed in SRef. [31]. Panel (b) shows an enlarged view of the band structure along the $\Gamma - Y$ line highlighted with a green circle in (a). In (b), there is an avoided crossing between the highest valence (blue) and lowest conduction (red) bands, which is a remnant of a monopole-charged Dirac nodal line that is gapped by SOC [31]. The energy bands in (a,b) are doubly degenerate due to the presence of both inversion and spinful time-reversal symmetries [178]. (c) The spin spectrum of $P(s_x + s_z)P/\sqrt{2} \equiv Ps_{xz}P$ plotted along the same high-symmetry BZ path as in (a) [see SFig. 31(b)]. Despite being gapped along the high-symmetry $\mathbf{k}$-path in (c), the $Ps_{xz}P$ spectrum is in fact gapless, specifically exhibiting eight spin-Weyl nodes at $Ps_{xz}P$ eigenvalue 0. (d) A 3D plot of the locations of the spin-Weyl nodes in the $Ps_{xz}P$ spectrum of β-MoTe$_2$. Each node in (d) is a monopole source of partial Chern number $C^+_{\gamma_1}$ for the positive spin bands [see SN 3 F for the definition of the partial monopole (chiral) charge of a spin-Weyl node]. In (d), spin-Weyl nodes with a positive (negative) partial chiral charge $C^+_{\gamma_1} = 1$ ($C^+_{\gamma_1} = -1$) are shown in orange (blue). (e) shows the projection of the spin-Weyl nodes from (d) onto the $k_1 - k_2$ BZ plane. In (e), spin-Weyl nodes in the $k_3 > 0$ half of the BZ are labeled with circles, and spin-Weyl nodes in the $k_3 < 0$ half of the BZ are labeled with squares. In the $k_3 > 0$ half of the BZ in (d,e), there are three spin-Weyl nodes with partial chiral charge -1 and one spin-Weyl node with partial chiral charge $+1$. Due to time-reversal symmetry, which flips the sign of the chiral charge of spin-Weyl points (see SN 2 E), there are therefore in the $k_3 < 0$ half of the BZ three spin-Weyl nodes with partial chiral charge $+1$ and one spin-Weyl node with partial chiral charge -1. The calculations detailed in this figure were performed using the freely available Python package NESTED_AND_SPIN_RESOLVED_WILSON_LOOP [97], which represents an extension of the PythTB open-source Python tight-binding package [171] that was implemented and utilized for the preparation of SRefs. [8, 15] and the present work.

in SFig. 32(b)], which is a remnant of a monopole-charged Dirac nodal line that is gapped by SOC [31].

In 3D β-MoTe$_2$, there are 56 valence electrons per unit cell. To analyze the (spin-resolved) topology of the valence electrons in β-MoTe$_2$, we will hence in the discussion below take the lowest 56 electronic states in energy of $[H_{\text{MoTe}_2}]$ to be separately occupied at each $\mathbf{k}$ point. Though β-MoTe$_2$ is a (semi)metal [*i.e.* valence and conduction bands cross E_F (set to zero) in SFig. 32(a,b)], the topology of the 56 valence bands of β-MoTe$_2$ is nevertheless a gauge-invariant quantity. Specifically, the 56 occupied (valence) bands in β-MoTe$_2$ are separated from the unoccupied (conduction) bands by an energy gap at each $\mathbf{k}$ point. Because there is an energy gap between the 56th and 57th Bloch states at each $\mathbf{k}$ point [SFig. 32(a)], we can therefore uniquely and consistently define a projector onto the valence bands at each $\mathbf{k}$ point to characterize their topology across the 3D BZ [107, 256]. Hence throughout this work, we will analyze β-MoTe$_2$—despite the existence of electron and hole pockets at E_F—as if it is an insulator with 56 occupied valence bands. Lastly, by computing the SIs of our Wannier-based tight-binding model, we find that β-MoTe$_2$ is specifically a helical HOTI with $(z_4, z_{21}, z_{22}, z_{23}) = (2, 0, 0, 0)$, consistent with the findings of previous works [31, 50]. Below in SN 9 B, we will next analyze the spin-resolved topology of the 56 occupied valence bands in β-MoTe$_2$.

B. Spin-Resolved Topology of β-MoTe$_2$

In this section, we will show that the helical HOTI phase of β-MoTe$_2$ carries the spin-resolved topology of a spin-Weyl semimetal state (SN 4 D 3) with an even number of spin-Weyl nodes in each half of the BZ for every choice of spin resolution direction.

To begin, we recall that the $P_\pm$-Wilson loops defined in SN 3 B depend on the choice of spin operator $s = \hat{\mathbf{n}} \cdot \mathbf{s}$ used to separate occupied states into positive and negative PsP eigenspaces. To analyze the spin-resolved topology of β-MoTe$_2$, we first compute the partial Chern numbers $C^\pm_{\gamma_1}(\hat{\mathbf{n}}, k_i)$ [SEq. (3.24)] through the winding numbers of the $P_\pm$-Wilson loops of the occupied states in constant-k_i BZ planes ($i = 1, 2, 3$). For $\hat{\mathbf{n}}$ sampled on a uniform (in spherical coordinates) mesh of 451 points in the upper hemisphere of the unit sphere (to exclude spin directions related by $\mathcal{T}$), we show in SFig. 33(a,b) the winding numbers of the $P_\pm$-Wilson loop spectra for the positive spin bands in the $\mathcal{T}$-invariant $k_i = 0$ and $k_i = \pi$ planes. We first find that for all choices of spin direction $\hat{\mathbf{n}}$ and for all $i = 1, 2, 3$, the partial Chern numbers of the occupied bands in the $k_i = \pi$ planes vanish [$C^\pm_{\gamma_1}(\hat{\mathbf{n}}, k_i = \pi) = 0$]. This implies that the occupied bands of the Hamiltonian [H_{MoTe_2}] restricted to each $k_i = \pi$ plane are equivalent to a 2D $\mathcal{I}$- and $\mathcal{T}$-symmetric Hamiltonian with trivial spin-resolved stable topology. Next, in SFig. 33(a–c) we show the partial Chern number $C^+_{\gamma_1}(\hat{\mathbf{n}}, k_i = 0)$ for $i = 1, 2, 3$ computed as a function of $\hat{\mathbf{n}}$. We see that $C^\pm_{\gamma_1}(\hat{\mathbf{n}}, k_i = 0)$ takes the values 0 or ± 2 for different choices of spin direction when $i \neq 2$. Recalling from SN 2 E and 3 E that the partial Chern number can only change as a function of k_i due to the presence of spin-Weyl nodes (chirally-charged 3D spin-gap closing points), we deduce that for the spin resolution directions $\hat{\mathbf{n}}$ for which $C^\pm_{\gamma_1}(\hat{\mathbf{n}}, k_i = 0) = \pm 2$, β-MoTe$_2$ must host an even number of spin-Weyl nodes per half BZ.

If we focus in particular on constant-k_3 planes, we see from SFig. 33(c) that there is a set of $\hat{\mathbf{n}}$ for which the partial Chern numbers $C^\pm_{\gamma_1}(\hat{\mathbf{n}}, k_3 = 0) = C^\pm_{\gamma_1}(\hat{\mathbf{n}}, k_3 = \pi) = 0$. From this, one might assume that there are no spin-Weyl nodes for these $\hat{\mathbf{n}}$, for example $\hat{\mathbf{n}} = \hat{\mathbf{y}}$. However comparing with SFig. 33(a), we see that when $C^\pm_{\gamma_1}(\hat{\mathbf{n}}, k_3 = 0) = 0$, the partial Chern numbers $C^\pm_{\gamma_1}(\hat{\mathbf{n}}, k_1 = 0)$ are poorly conditioned: if the spin gap were open near the $k_1 = 0$ plane, we would expect the partial Chern number $C^\pm_{\gamma_1}(\hat{\mathbf{n}}, k_1 = 0)$ to be constant in $\hat{\mathbf{n}}$ taken over continuous patches of the upper spin hemisphere. However in SFig. 33(a), we see that the partial Chern number oscillates rapidly between -2, 0, and 2 as $\hat{\mathbf{n}}$ is varied. Through an explicit numerical computation of the PsP spectrum, we verify that this rapid oscillation of $C^+_{\gamma_1}$ is due to the presence of spin-Weyl nodes lying close to the $k_1 = 0$ plane. Specifically, as done previously in SN 3 E, we determine the $\mathbf{k}$-space locations of spin-Weyl nodes by minimizing the absolute value of the determinant of the reduced spin matrix [s_{reduced}] [SEq. (3.5)]. This provides a reasonable indicator of the locations of the spin-Weyl nodes in 3D systems like β-MoTe$_2$ with $\mathcal{I}$ and $\mathcal{T}$ symmetries, which together restrict spin-Weyl nodes to only occur when two eigenvalues of [s_{reduced}] go to zero (see SN 3 E). We show a representative configuration of spin-Weyl points in β-MoTe$_2$ for $\hat{\mathbf{n}} = \hat{\mathbf{y}}$ in SFig. 33(d), in which we find that indeed numerous spin-Weyl nodes lie within the small BZ region $|k_1| < 0.05\pi$. Below in SN 9 B 1, we will further perform an extensive numerical sampling and minimization of the spin gap for all spin directions $\hat{\mathbf{n}}$, the results of which demonstrate the absence of a discernible numerical spin gap in any spin resolution direction in β-MoTe$_2$. This calculation allows us to conclude that even for the values of $\hat{\mathbf{n}}$ in SFig. 33(c) for which $C^\pm_{\gamma_1}(\hat{\mathbf{n}}, k_3 = 0) = C^\pm_{\gamma_1}(\hat{\mathbf{n}}, k_3 = \pi) = 0$, β-MoTe$_2$ realizes a spin-Weyl semimetal state with an even number of spin-Weyl nodes per half BZ. To summarize, through spin-resolved Wilson loop calculations (supported by direct spin gap calculations that will be detailed in SN 9 B 1), we have demonstrated that for all choices of $\hat{\mathbf{n}}$, β-MoTe$_2$ carries an even number of spin-Weyl nodes per half BZ whose positions continuously evolve as a function of the choice of spin direction $\hat{\mathbf{n}}$, but not in a manner in which all spin-Weyl points are annihilated for any particular $\hat{\mathbf{n}}$. As shown in SRef. [17] and discussed in the main text, simply doubling—or "stacking"—a model of a 3D strong TI is one way of constructing a model of a helical HOTI, a construction that the authors of SRef. [17] specifically termed a "doubled strong TI" (DSTI). Because a 3D TI hosts an odd number of spin-Weyl nodes per half BZ (SN 3 E), then a helical HOTI in the DSTI regime necessarily hosts an even number of spin-Weyl points per half BZ. As shown in SN 4 D 3, the DSTI regime of a helical HOTI with $\mathcal{I}$ and $\mathcal{T}$ symmetries can be understood as an intermediate critical spin-stable (spin-gapless) phase separating spin-gapped $\mathcal{T}$-doubled axion insulator [SFig. 22(b)] and 3D quantum spin Hall insulator [SFig. 22(a)] states (see the main text and SN 4 D). From the above analysis, we determine β-MoTe$_2$ to be a helical HOTI that lies in the DSTI regime for all choices of spin resolution direction $\hat{\mathbf{n}}$.

We will next fix a choice of $\hat{\mathbf{n}}$ and investigate the spin spectrum in more detail. As we will show below, the spin spectrum (spin-Weyl point distribution) in β-MoTe$_2$ is particularly simple for $\hat{\mathbf{n}} = \hat{\mathbf{n}}_{xz} = (\hat{\mathbf{x}} + \hat{\mathbf{z}})/\sqrt{2}$, to which we will hence specialize in the analysis below. This specialization to $\hat{\mathbf{n}}_{xz}$ is further justified because $\hat{\mathbf{n}}_{xz}$ is invariant under the $m_y \times \mathcal{T}$ (magnetic [43]) reflection symmetry of β-MoTe$_2$ [nonmagnetic SG $P2_1/m1'$ (#11.51)], and recent studies have observed preferential spin-electromagnetic responses for reflection-invariant spin directions in devices based on transition metal dichalcogenides like MoTe$_2$ [137–139, 257, 258].

First, from our analysis in SFig. 33(c), we see that the partial Chern numbers $C^\pm_{\gamma_1}(\hat{\mathbf{n}}_{xz}, k_3 = 0) = \pm 2$, while $C^\pm_{\gamma_1}(\hat{\mathbf{n}}_{xz}, k_3 = \pi) = 0$. This is precisely what one would expect from a naive doubling of the partial Chern numbers of

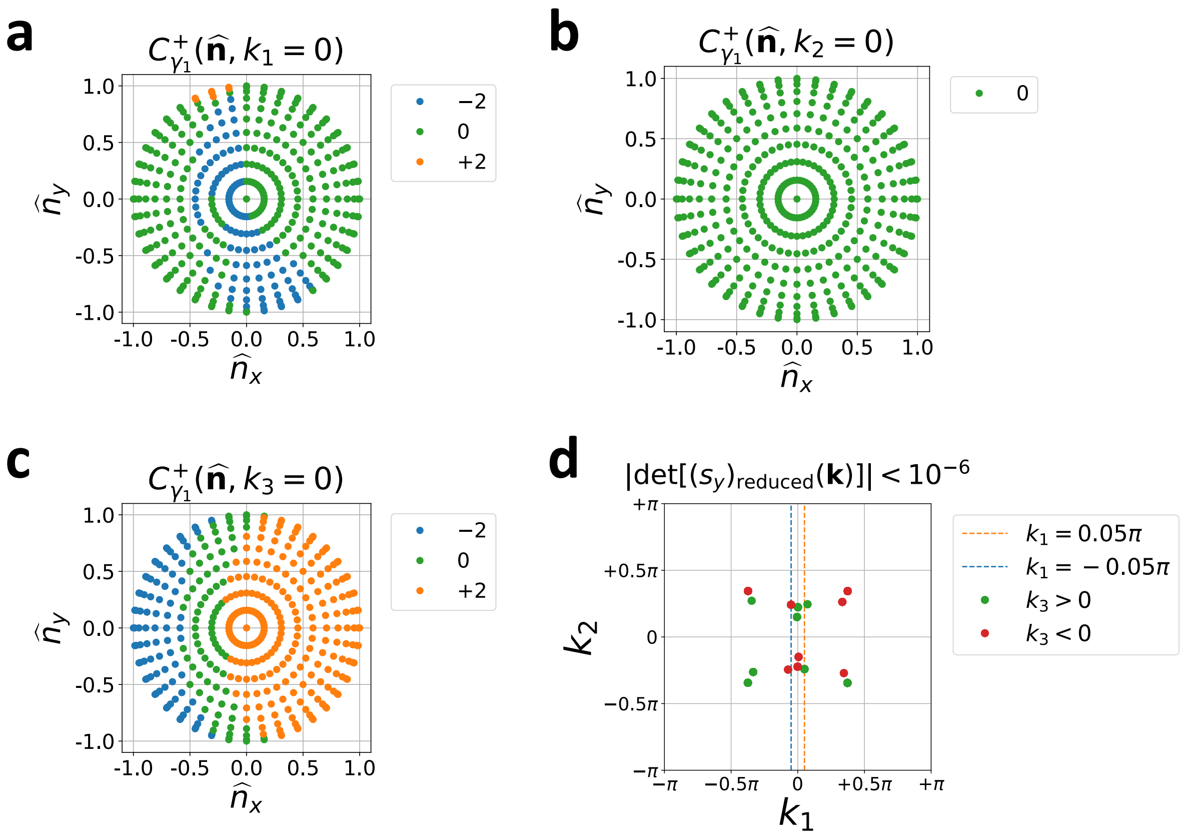

Supplementary Figure 33. Partial Chern numbers for the occupied bands of β-MoTe$_2$ as a function of the spin resolution direction $\hat{\mathbf{n}}$ in $P\hat{\mathbf{n}}\cdot\mathbf{s}P$. The direction $\hat{\mathbf{n}}$ of the spin operator is taken to lie in the upper hemisphere $\hat{n}_z \geq 0$ without loss of generality, which is sufficient to determine the spin-resolved topology (here partial Chern numbers) for all $\hat{\mathbf{n}}$ in time-reversal- ($\mathcal{T}$-) invariant insulators, because $\mathcal{T}$ symmetry relates the $\pm\hat{\mathbf{n}}$ spin resolution directions. The x- and y-axes in panels (a-c) respectively correspond to the $\hat{n}_x$ and $\hat{n}_y$ components of the spin-resolution unit vector $\hat{\mathbf{n}}$. First, we find that $C^+_{\gamma_1}(\hat{\mathbf{n}}, k_i = \pi) = 0$ for all $\hat{\mathbf{n}}$; we hence do not show in this figure the partial Chern numbers in the $k_i = \pi$ planes. (a) shows the partial Chern number $C^+_{\gamma_1}(\hat{\mathbf{n}}, k_1 = 0)$ in the $k_1 = 0$ plane as a function of $\hat{\mathbf{n}}$. In (a), $C^+_{\gamma_1}(\hat{\mathbf{n}}, k_1 = 0)$ oscillates between $-2, 0$ and 2 as $\hat{\mathbf{n}}$ is varied. In particular, when $\hat{n}_x < 0$ we find that the regions of constant $C^+_{\gamma_1}(\hat{\mathbf{n}}, k_1 = 0)$ are discontinuous. This is a signature of spin-Weyl nodes lying very close to the $k_1 = 0$ plane, for which the representative example of $\hat{\mathbf{n}} = \hat{\mathbf{y}}$ is shown in (d). (b) shows the partial Chern number $C^+_{\gamma_1}(\hat{\mathbf{n}}, k_2 = 0)$ in the $k_2 = 0$ plane as a function of $\hat{\mathbf{n}}$. We see that $C^+_{\gamma_1}(\hat{\mathbf{n}}, k_2 = 0) = 0$ for all spin directions. (c) shows the partial Chern number $C^+_{\gamma_1}(\hat{\mathbf{n}}, k_3 = 0)$ in the $k_3 = 0$ plane as a function of $\hat{\mathbf{n}}$. We see that there are well-defined (smooth and continuous) regions in spin-resolution parameter ($\hat{\mathbf{n}}$-) space for which $C^+_{\gamma_1}(\hat{\mathbf{n}}, k_3 = 0) = 0, \pm 2$. In (d), we plot the 2D projection onto the $k_1 - k_2$ plane of the $\mathbf{k}$ points at which the absolute values of the determinant of the reduced spin s_y matrix [SEq. (3.5) for $\hat{\mathbf{n}} = \hat{\mathbf{y}}$] is smaller than 10^{-6}, which numerically defines the locations of the spin-Weyl nodes for the spin resolution direction $\hat{\mathbf{n}} = \hat{\mathbf{y}}$. We find numerous (8) additional spin-Weyl points lying close to $k_1 = 0$ for $\hat{\mathbf{n}} = \hat{\mathbf{y}}$ in (d), consistent with the numerical oscillations of $C^+_{\gamma_1}(\hat{\mathbf{n}}, k_1 = 0)$ in (a). The calculations detailed in this figure were performed using the freely available Python package NESTED_AND_SPIN_RESOLVED_WILSON_LOOP [97], which represents an extension of the PythTB open-source Python tight-binding package [171] that was implemented and utilized for the preparation of SRefs. [8, 15] and the present work.

the adapted BHZ model of a 3D strong TI (SN 3 E), consistent with our above determination of β-MoTe$_2$ as a DSTI.

We next explicitly compute the configuration of spin-Weyl nodes in β-MoTe$_2$ for $\hat{\mathbf{n}} = \hat{\mathbf{n}}_{xz}$. We find that there are a total of four spin-Weyl nodes in each half of the BZ. We plot the locations of the spin-Weyl nodes in the 3D BZ for $\hat{\mathbf{n}} = \hat{\mathbf{n}}_{xz}$ in SFig. 32(d,e). Focusing on the upper half of the BZ ($k_3 \geq 0$), we see that there are three spin-Weyl nodes with partial Chern number (or partial monopole chiral charge) -1, and one spin-Weyl node with partial Chern number $+1$. From this, we deduce that the change in partial Chern number $C^+_{\gamma_1}(\hat{\mathbf{n}}_{xz}, k_3)$ between $k_3 = 0$ and $k_3 = \pi$

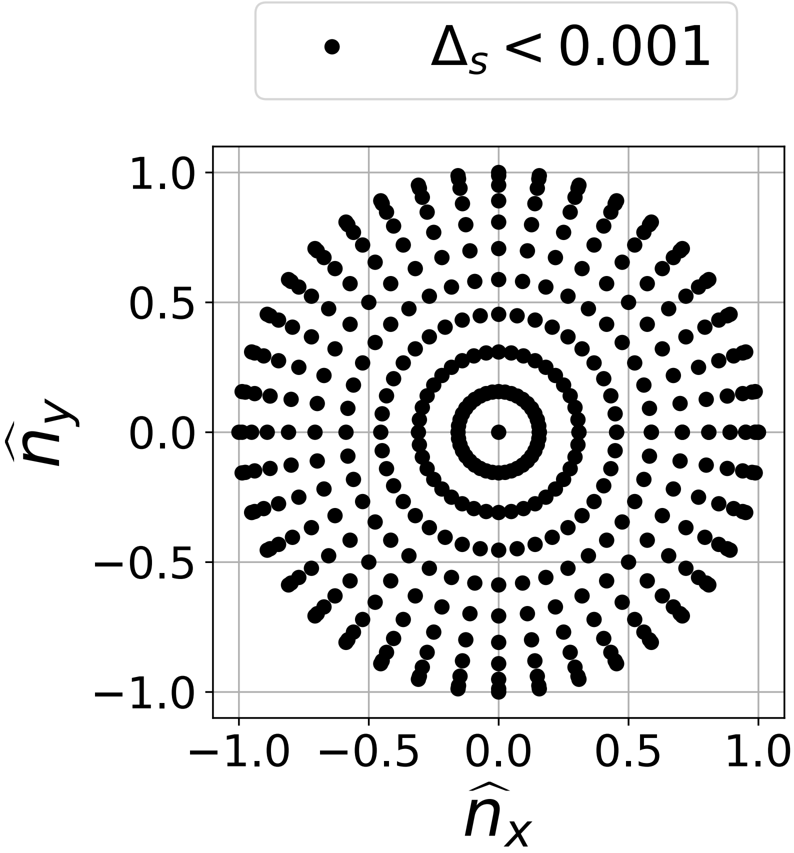

Supplementary Figure 34. Numerical spin gap $\Delta_{s=\hat{\mathbf{n}}\cdot\mathbf{s}}$ as a function of the spin resolution direction $\hat{\mathbf{n}}$ for 3D β-MoTe$_2$. The angular parameterization of $\hat{\mathbf{n}}$ is defined in SEq. (9.5), and our calculations were performed over the spin-direction hemisphere of $\hat{\mathbf{n}}$ defined by $\vartheta \in [0, 0.5\pi]$ and $\phi \in [0, 2\pi]$. For our calculations, the angular variables (ϑ, ϕ) were respectively sampled using the numerical resolution of $\Delta\vartheta = 0.05\pi$ and $\Delta\phi = 0.05\pi$. By performing a Nelder-Mead minimization [259] on the spin gap function $\Delta_s(\mathbf{k})$ individually for 100 $\mathbf{k}$ points randomly sampled from the 3D Brillouin zone as the initial points, we define for each $\hat{\mathbf{n}}$ the *numerical spin gap* $\Delta_{s=\hat{\mathbf{n}}\cdot\mathbf{s}}$ as the minimal value (in the units of $\hbar/2$) of the 100 minimization results for the fixed value of $\hat{\mathbf{n}}$. In this figure, we use black dots to indicate the values of $\hat{\mathbf{n}}$ for which the numerical spin gap $\Delta_{s=\hat{\mathbf{n}}\cdot\mathbf{s}}$ in β-MoTe$_2$ is less than $10^{-3} = 0.001$. From the uniform (projected) spacing of the black dots in this figure, we conclude that β-MoTe$_2$ is in fact spin-gapless (within numerical precision) for *all* values of $\hat{\mathbf{n}}$. For completeness, we note that within the resolution of the angular variables (ϑ, ϕ) sampled over the spin hemisphere considered in our calculations, the maximum numerical spin gap $\Delta_s \approx 1.401393465896699 \times 10^{-4}$ lies at $(\vartheta, \phi) = (0.25\pi, 0.05\pi)$, and the minimal numerical spin gap $\Delta_s \approx 2.4070658354675936 \times 10^{-6}$ lies at $(\vartheta, \phi) = (0.05\pi, 1.75\pi)$. The calculations detailed in this figure were performed using the freely available Python package NESTED_AND_SPIN_RESOLVED_WILSON_LOOP [97], which represents an extension of the PythTB open-source Python tight-binding package [171] that was implemented and utilized for the preparation of SRefs. [8, 15] and the present work.

is given by

$$C^+_{\gamma_1}(\hat{\mathbf{n}}_{xz}, k_3 = \pi) - C^+_{\gamma_1}(\hat{\mathbf{n}}_{xz}, k_3 = 0) = -1 - 1 - 1 + 1 = -2. \tag{9.4}$$

This is consistent with our calculation in SFig. 33(c) showing that $C^+_{\gamma_1}(\hat{\mathbf{n}}_{xz}, k_3 = \pi) = 0$ and $C^+_{\gamma_1}(\hat{\mathbf{n}}_{xz}, k_3 = 0) = +2$. We therefore conclude that while β-MoTe$_2$ realizes a DSTI for all choices of $\hat{\mathbf{n}}$, β-MoTe$_2$ is not a *minimal* DSTI for $\hat{\mathbf{n}} = \hat{\mathbf{n}}_{xz}$. Specifically, in a helical HOTI lying in the minimal DSTI regime, there are only two spin-Weyl nodes of the same partial chiral charge in each half of the 3D BZ. Instead, for $\hat{\mathbf{n}} = \hat{\mathbf{n}}_{xz}$ in β-MoTe$_2$, an additional dipole of spin-Weyl nodes of opposite partial Chern numbers is present in each half of the BZ.

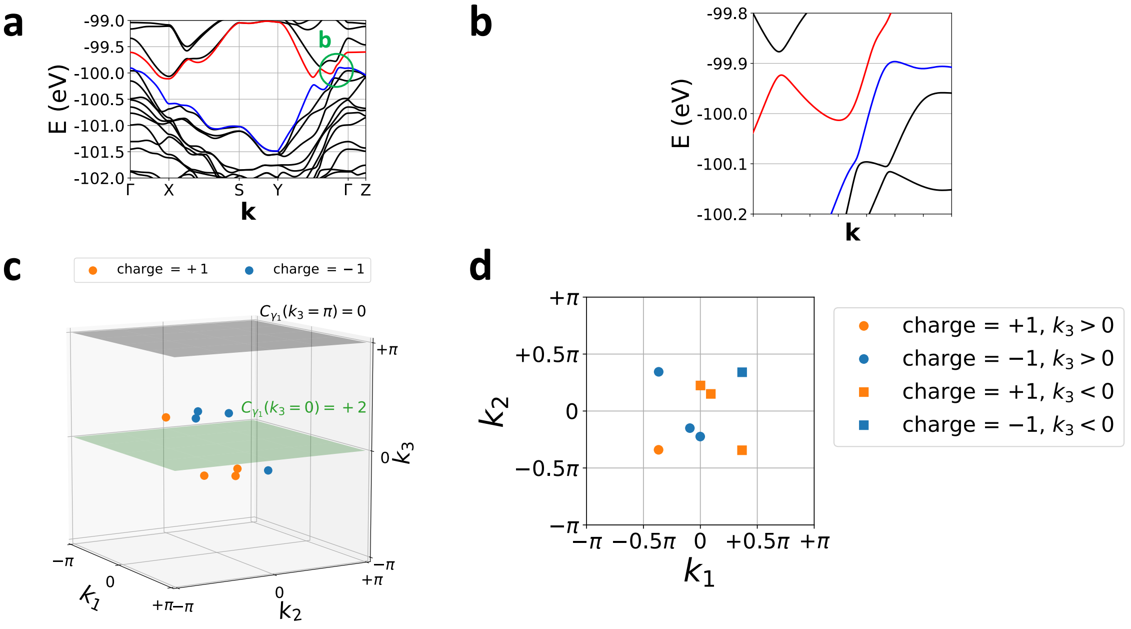

Supplementary Figure 35. Energy spectrum and Weyl nodes for β-MoTe$_2$ in a strong $(\hat{\mathbf{x}}+\hat{\mathbf{z}})$-directed Zeeman field. (a) The band structure of $[H_Z]$ in SEq. (9.8), which was obtained by applying a strong $(\hat{\mathbf{x}}+\hat{\mathbf{z}})$-directed (spin-) Zeeman field to a Wannier-based tight-binding model of β-MoTe$_2$ obtained from DFT calculations (SN 9 A). Near $E \approx -B = -100$eV in (a), the electronic band structure appears qualitatively similar to the zero-field bulk band structure in SFig. 32(a), albeit with half the number of bands [the remaining bands have energies $E \approx +B$, and so do not appear (a)]. Because the product of inversion and time-reversal symmetries ($\mathcal{I} \times \mathcal{T}$) is broken by the large Zeeman field, the energy bands in (a) are singly degenerate at generic $\mathbf{k}$ points, as opposed to the doubly-degenerate bulk bands in β-MoTe$_2$ in the absence of an external field [SFig. 32(a)]. (b) shows an enlarged view of the energy band structure along the $\Gamma - Y$ line near the green circle in (a). In (b), there is an avoided crossing between the red and blue bands. Nevertheless, upon closer examination, we find that the red and blue bands in (a,b) do indeed cross and form Weyl points at lower-symmetry $\mathbf{k}$ points throughout the 3D BZ. (c) shows the positions and monopole chiral charges of the eight Weyl nodes in the energy spectrum of $[H_Z]$. The locations and chiral charges of the Weyl points in (c) are nearly identical to the locations and chiral charges of the $\hat{\mathbf{n}}_{xz}$ spin-Weyl nodes in β-MoTe$_2$ [SFig. 32(d,e)]. (d) shows a projection of the Weyl nodes in (c) onto the $k_1 - k_2$ plane; Weyl nodes with positive (negative) chiral charges are shown in orange (blue). In the $k_3 > 0$ half of the BZ in (c,d), there are three Weyl nodes with charge -1 and one Weyl node with charge $+1$. As required by inversion symmetry, which is preserved by an external Zeeman field, there are three Weyl nodes with charge $+1$ and one Weyl node with charge -1 in the $k_3 < 0$ half of the BZ. The calculations detailed in this figure were performed using the freely available Python package NESTED_AND_SPIN_RESOLVED_WILSON_LOOP [97], which represents an extension of the PythTB open-source Python tight-binding package [171] that was implemented and utilized for the preparation of SRefs. [8, 15] and the present work.

1. *Searching for a Spin Gap in β-MoTe$_2$ at Generic Spin Resolution Directions*

As shown in SFigs. 32 and 33, by calculating the partial Chern numbers of occupied states in high-symmetry ($\mathcal{T}$-invariant) BZ planes, 3D β-MoTe$_2$ can be determined to lie in the DSTI regime of a helical HOTI [17, 21, 31, 50] for a large range of spin resolution directions $\hat{\mathbf{n}}$. However, as shown in SFig. 33(a,d), there exist several regions of $\hat{\mathbf{n}}$-parameter space (such as in the vicinity of $\hat{\mathbf{n}} = \hat{\mathbf{y}}$) in which the partial Chern number is poorly conditioned (*i.e.* unstably oscillating). To further characterize the spin-resolved topology of β-MoTe$_2$ in the spin directions for which high-symmetry-plane ($k_1 = 0$) partial Chern numbers cannot be computed, we in this section directly numerically investigate the presence (or absence) of a spin gap throughout the full 3D BZ for each spin direction $\hat{\mathbf{n}}$ in the $P(\hat{\mathbf{n}} \cdot \mathbf{s})P$ spectrum of β-MoTe$_2$. As shown below, our calculations show that β-MoTe$_2$ realizes a spin-Weyl semimetal (spin-gapless) state for *all* choices of spin resolution direction $\hat{\mathbf{n}}$.

To begin, we parameterize the spin direction $\hat{\mathbf{n}}$ as a 3D unit vector described by

$$\hat{\mathbf{n}} = (\sin\vartheta\cos\phi, \sin\vartheta\sin\phi, \cos\vartheta). \tag{9.5}$$

The parameterization in SEq. (9.5) is chosen such that the angular variables $(\vartheta, \phi) = (0, 0)$, $(0.5\pi, 0)$, and $(0.5\pi, 0.5\pi)$

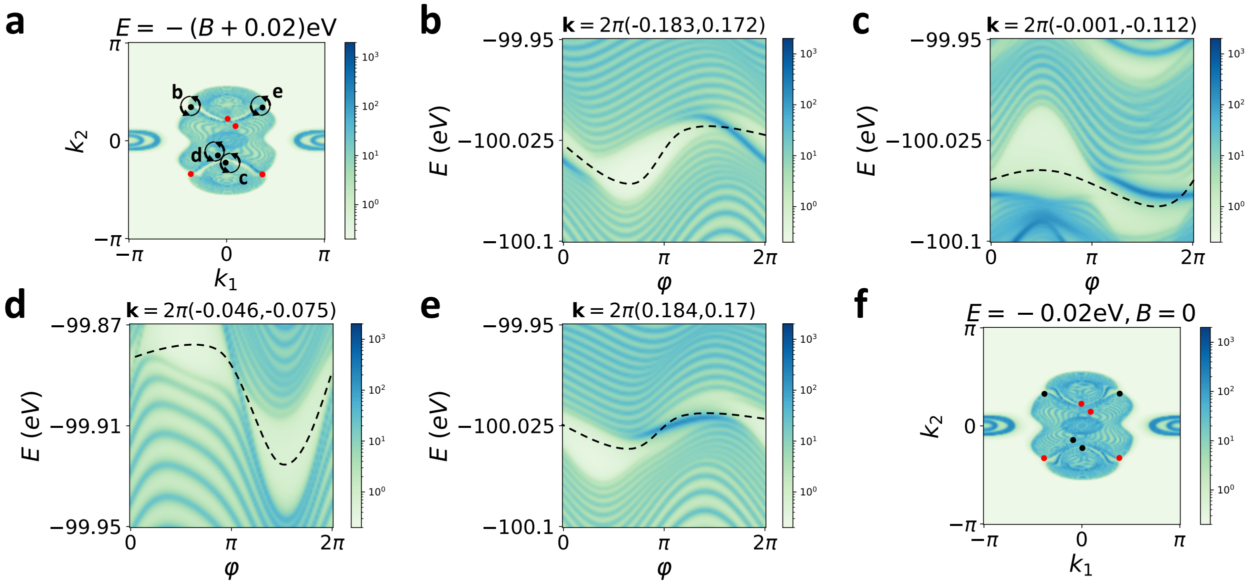

Supplementary Figure 36. Spectral function for the (001)-surface of β-MoTe$_2$ in a strong $(\hat{\mathbf{x}}+\hat{\mathbf{z}})$-directed Zeeman field. (a) shows the surface spectral function at $E=(-B-0.02)$eV as a function of k_1 and k_2 for an (001)-oriented slab of the tight-binding model $[H_Z]$ in SEq. (9.8), which consists of a DFT-obtained, Wannier-based tight-binding model of β-MoTe$_2$ (SN 9 A) subjected to a $B=100$eV (spin-) Zeeman field. In (a), red and black dots mark the positions of the Weyl nodes in the energy spectrum with positive and negative chiral charges, respectively. Arc-like surface features can be seen emanating from the surface projections of the bulk Weyl points. (b–e) show the surface spectral function computed on circles surrounding the negatively-charged Weyl nodes [indicated in panel (a)] plotted as functions of E and the polar angle φ of the circles in (a). $\varphi=0$ corresponds to the positive k_1 axis, and the circles are traversed counterclockwise in (b–e) as indicated by the black arrows in (a). In (b–e), gap-traversing chiral modes indicative of topological surface Fermi arcs can be seen emanating towards the center of the surface BZ. In panel (b), we see that the Fermi-arc chiral mode crosses the dashed black line near $\varphi\approx 3\pi/2$, indicating that the Fermi arc is directed towards the origin in (a). In (b), there is specifically one chiral surface state with a negative slope crossing the dashed line, in correspondence with the -1 charge of the encircled Weyl node in (a). (c) and (d) show surface spectral functions surrounding the surface projections of the two Weyl nodes closest to the origin in the $k_2<0$ half of (a). The Weyl points in (c,d) are embedded into the continuum of bulk states at lower energies. In both (c,d), a single chiral surface state with negative slope crosses the dashed line, corresponding again to the -1 charge of the encircled Weyl nodes in (a). (e) shows the surface spectral function surrounding the Weyl node in the upper right quadrant of (a). In (e), there is a Fermi-arc surface state near $\varphi=\pi$, which corresponds to an arc emanating towards the center of the surface BZ in (a). A single chiral surface state with negative slope crosses the dashed line in (e), corresponding to the -1 charge of the encircled Weyl-node projection in (a). To draw comparison with the Zeeman-field energy spectrum in (a), in (f), we plot the zero-field (001)-surface spectral function of β-MoTe$_2$ at $E=-0.02$eV and indicate the surface projections and partial chiral charges of the bulk $\hat{\mathbf{n}}_{xz}$ spin-Weyl nodes. The zero-field spin-Weyl points in (f) lie at almost identical $\mathbf{k}$ positions and carry the same chiral charges as the Zeeman-induced Weyl points in (a).

correspond to the Cartesian unit vectors $\hat{\mathbf{z}}$, $\hat{\mathbf{x}}$, and $\hat{\mathbf{y}}$, respectively. Because 3D β-MoTe$_2$ has both inversion ($\mathcal{I}$) and spinful time-reversal ($\mathcal{T}$) symmetries, its PsP spectrum exhibits an antiunitary chiral symmetry due to the relation

$$[\mathcal{I}][\mathcal{T}][P(\mathbf{k})]s[P(\mathbf{k})]([\mathcal{I}][\mathcal{T}])^{-1}=-[P(\mathbf{k})]s[P(\mathbf{k})], \tag{9.6}$$

where $[\mathcal{I}]$ and $[\mathcal{T}]$ are the unitary and antiunitary matrix representatives of $\mathcal{I}$ and spinful $\mathcal{T}$ acting on the 88×88 Wannier-based tight-binding Bloch Hamiltonian matrix $[H_{\mathrm{MoTe}_2}(\mathbf{k})]$ that we obtained from DFT calculations of the electronic structure of 3D β-MoTe$_2$ (SN 9 A). In SEq. (9.6), $[P(\mathbf{k})]$ denotes the matrix projector $[P(\mathbf{k})]=\sum_{n=1}^{56}|u_{n,\mathbf{k}}\rangle\langle u_{n,\mathbf{k}}|$ onto the 56 occupied (valence) bands of β-MoTe$_2$ (see SN 9 A), where $|u_{n,\mathbf{k}}\rangle$ is the nth eigenvector of $[H_{\mathrm{MoTe}_2}(\mathbf{k})]$. As discussed in SN 2 B, the presence of $\mathcal{I}\times\mathcal{T}$ symmetry [SEq. (9.6)] further implies that

$$\mathrm{Spec}\left([P(\mathbf{k})]s[P(\mathbf{k})]\right)=-\mathrm{Spec}\left([P(\mathbf{k})]s[P(\mathbf{k})]\right), \tag{9.7}$$

in which Spec($\mathcal{O}$) denotes the spectrum of the operator $\mathcal{O}$. From SEq. (9.7), we can then define the spin gap function $\Delta_s(\mathbf{k})$ as the difference between the smallest positive and largest negative PsP eigenvalues at each crystal momentum $\mathbf{k}$. From the values of $\Delta_s(\mathbf{k})$ taken across the BZ for a given spin operator $s=\hat{\mathbf{n}}\cdot\mathbf{s}$, we then define the spin gap Δ_s as $\Delta_s\equiv\min_{\mathbf{k}\in\mathrm{BZ}}[\Delta_s(\mathbf{k})]$, namely the minimal value of $\Delta_s(\mathbf{k})$ over the entire BZ.

Having established the spin-direction parameterization in SEq. (9.5) as a function of the angular variables (ϑ, ϕ) and defined the spin gap $\Delta_s \equiv \min_{\mathbf{k}\in \mathrm{BZ}}[\Delta_s(\mathbf{k})]$ in the text following SEq. (9.7), we next compute $\Delta_{\hat{\mathbf{n}}\cdot\mathbf{s}}$ as a function of the spin direction $\hat{\mathbf{n}}$ through numerical minimization. Practically, for each spin direction $\hat{\mathbf{n}}$, we perform a Nelder-Mead minimization [259] on the spin gap function $\Delta_s(\mathbf{k})$ for 100 $\mathbf{k}$ points randomly sampled across the 3D BZ as the initial points. We then define the *numerical spin gap* Δ_s as the minimal value of the 100 minimization results for each spin direction $\hat{\mathbf{n}}$.

In SFig. 34, we show the numerical spin gap Δ_s (in the units of $\hbar/2$) for $\hat{\mathbf{n}}$ (projected onto the xy plane) sampled over the spin hemisphere defined by $\vartheta \in [0, 0.5\pi]$ and $\phi \in [0, 2\pi]$ using the angular resolutions $\Delta\vartheta = 0.05\pi$ and $\Delta\phi = 0.05\pi$. Because $\Delta_s = \Delta_{-s}$ due to $\mathcal{T}$ symmetry, this sampling of the angular variables (ϑ, ϕ) contains all independent values of the numerical spin gap Δ_s over the full sphere of spin resolution directions $\hat{\mathbf{n}}$. In SFig. 34, we use black dots to indicate the spin directions $\hat{\mathbf{n}}$ for which Δ_s is smaller than 10^{-3}=0.001. From the regular (projected) spacing of black dots in SFig. 34, we conclude that the numerical spin gap Δ_s of 3D β-$MoTe_2$ is smaller than 10^{-3} for all $\hat{\mathbf{n}}$, which implies that within numerical precision, β-$MoTe_2$ is spin-gapless for *all* choices of $\hat{\mathbf{n}}$. Within the spin spectrum, the only nodal degeneracies that can appear in 3D insulators with only bulk $\mathcal{I}$ and $\mathcal{T}$ symmetry are 3D spin-Weyl points (SN 4 D 3); other degeneracies, such as spin-nodal-lines, would require higher crystal symmetries. In addition to $\mathcal{I}$ and $\mathcal{T}$ symmetries, the crystal structure of β-$MoTe_2$ [SG $P2_1/m1'$ (#11.51)], respects m_y mirror and s_{2y} screw symmetries, which are both broken at generic spin resolution directions. Specifically, the spin spectrum of β-$MoTe_2$ is only constrained by symmetries other than $\mathcal{I}$ and $\mathcal{T}$ for $\hat{\mathbf{n}} = \hat{\mathbf{y}}$ (m_y-preserving) and $\hat{\mathbf{n}}$ lying in the xz-plane ($m_y \times \mathcal{T}$-preserving). Together with our previous observation of numerically stable partial Chern numbers for $\hat{\mathbf{n}}$ lying in the xz-plane [SFig. 33(a,b,c)], and our direct computation of the spin spectrum for $\hat{\mathbf{n}} = \hat{\mathbf{y}}$ [the spin-Weyl state shown in SFig. 33(d)], the spin-gaplessness of β-$MoTe_2$ in SFig. 34 hence specifically indicates that 3D β-$MoTe_2$ realizes a spin-Weyl state for all choices of spin resolution direction $\hat{\mathbf{n}}$.

C. Physical Observables of the Spin-Weyl State in β-$MoTe_2$: Surface Fermi Arcs in a Strong Zeeman Field

To demonstrate physical signatures of the bulk spin-Weyl points β-$MoTe_2$, we begin with the relationship, previously established in SN 2 G, between the PsP spectrum and the spectrum of the Hamiltonian in a strong Zeeman field. Specifically, as shown in SN 2 G, if the bulk of an insulator hosts a spin-Weyl semimetal state for a particular spin resolution direction $\hat{\mathbf{n}}$, then under the application of a large Zeeman field parallel to $\hat{\mathbf{n}}$, the *energy* spectrum of the system will develop Weyl points whose positions in $\mathbf{k}$ and chiral charges lie close to those of the spin-Weyl points in the original zero-field insulator. For $\hat{\mathbf{n}} = \hat{\mathbf{n}}_{xz} = (\hat{\mathbf{x}} + \hat{\mathbf{z}})/\sqrt{2}$ in β-$MoTe_2$ [SFig. 32(c,d,e)], we specifically expect that the low-energy ($E \sim -B$) bands of

$$\begin{aligned}[H_Z] &= [H_{\mathrm{MoTe}_2}] - \frac{B}{\sqrt{2}}([s_x] + [s_z]) \\ &= [H_{\mathrm{MoTe}_2}] - B s_{xz} \end{aligned} \tag{9.8}$$

will host Weyl nodes that are adiabatically connected to the spin-Weyl nodes in the $Ps_{xz}P$ spectrum. To verify this, in SFig. 35(a,b) we compute the energy band structure for $[H_Z]$ in SEq. (9.8) with the external Zeeman field strength set to $B = 100$eV. Along high-symmetry BZ lines, we see that the electronic band structure at $E \approx -B \approx -100$eV in the presence of a strong $(\hat{\mathbf{x}} + \hat{\mathbf{z}})$-directed Zeeman field structure strongly resembles that of the original field-free Hamiltonian [shown in SFig. 32(a,b)], albeit with half the number of bands [the remaining bands lie at energies $E \approx +B$, and hence do not appear in SFig. 35(a)]. As was done previously in SN 9 B 1, we next perform a numerical minimization using the Nelder-Mead method [259] to search for degeneracies between the red and blue bands near $E = -100$eV in SFig. 35(a,b). We find that, like the $\hat{\mathbf{n}}_{xz}$ spin spectrum of β-$MoTe_2$ [SFig. 32(d,e)], the *energy* spectrum of $[H_Z]$ contains four Weyl nodes per half-BZ. In SFig. 35(c,d) we plot the locations and chiral charges of the Weyl nodes in the energy spectrum of $[H_Z]$ [SEq. (9.8)] in the vicinity of $E = -100$eV. We find that as expected from the analytic analysis in SN 2 G, the energy Weyl nodes in SFig. 35(c,d) lie at very similar positions to the $\hat{\mathbf{n}}_{xz}$ spin-Weyl nodes in β-$MoTe_2$ [SFig. 32(d,e)].

Finally, as established in SN 2 G and 3 H, the presence of Weyl nodes in the spectrum of $[H_Z]$ allows us to infer the existence of topological surface Fermi arcs on surfaces of β-$MoTe_2$ subjected to a strong (spin-) Zeeman field. To verify this intuition, we next construct an $L = 21$ unit-cell-thick slab of the tight-binding model $[H_Z]$ with a surface normal vector pointing in the experimentally-accessible (001)-direction of β-$MoTe_2$ [260–267]. To remove dangling-bond surface states, we have added a constant chemical potential offset for atoms in the quarter of the unit cell closest to each surface to passivate the outermost (fractional) van der Waals layer of the tight-binding model $[H_Z]$. In SFig. 36, we plot top- [(001)-] surface spectrum of $[H_Z]$ obtained from surface Green's functions. SFig. 36(b–e) specifically show the (001)-surface spectral function as a function of energy and position plotted along circles surrounding the surface

projections of the four energy Weyl points with -1 chiral charges. In all four panels [SFig. 36(b–e)], we observe a chiral surface mode traversing the (direct) gap between the bands surrounding $E = -100$eV, confirming the presence of topological surface Fermi arcs. In SFig. 36(a), we show the (001)-surface spectral function of $[H_Z]$ for a fixed energy $(-B - 0.02)$eV, plotted as a function of k_1 and k_2. The Fermi arcs in SFig. 36(b–e) can be seen extending towards the center of the BZ. To draw comparison with the energy spectrum of $[H_Z]$, in SFig. 36(f), we show the surface spectral function of $[H_{\text{MoTe}_2}]$ in the absence of a Zeeman field and label the (001)-surface projections and partial chiral charges of the $\hat{\mathbf{n}}_{xz}$ spin-Weyl points. The zero-field spin-Weyl points in SFig. 36(f) lie at almost identical $\mathbf{k}$ positions and carry the same chiral charges as the Zeeman-induced Weyl points in SFig. 36(a) [see also SFigs. 32(d,e) and 35(c,d)]).

To conclude, we have hence crucially demonstrated that the spin-resolved topological analysis techniques developed in this work can be applied to ab-initio calculations of the electronic structure of real materials, here specifically β-MoTe_2. Below, in SN 10, we will next apply the machinery of spin-resolved topology to the candidate helical HOTI α-BiBr [50, 52, 53], which unlike β-MoTe_2 exhibits a bulk topological spin gap for large regions of $\hat{\mathbf{n}}$ spin-resolution parameter space.

10. FIRST-PRINCIPLES ANALYSIS OF α-BiBr

In this section, we will compute the spin-resolved topology of 3D α-BiBr, which was theoretically identified in SRefs. [50, 52, 53] as a candidate helical higher-order topological insulator (HOTI) with both inversion ($\mathcal{I}$) and time-reversal ($\mathcal{T}$) symmetries. As previously with β-MoTe_2 in SN 9, the helical HOTI phase predicted in α-BiBr is characterized by a nontrivial $\mathbb{Z}_4$-invariant $z_4 = 2$ and vanishing weak indices $z_{2i} = 0$. Recent experimental studies have also revealed evidence of helical higher-order topology in α-BiBr, including signatures of 1D helical hinge states in laser angle-resolved photoemission experiments [64], and scanning tunneling microscopy signatures of hinge-localized, $\mathcal{T}$-protected 1D gapless (helical) conducting channels that persist up to room temperature [68]. Further experiments have also reported spectroscopic and transport signatures of helical hinge modes in α-BiBr [65, 66, 72, 109–113].

We will begin our spin-resolved topological analysis of α-BiBr below by first in SN 10 A detailing the DFT calculations that we performed to obtain a symmetric, Wannier-based tight-binding model of α-BiBr. We then in SN 10 B compute the PsP spin spectrum for α-BiBr over the full range of spin-resolution directions $\hat{\mathbf{n}}$. In SN 10 B, we will specifically use (nested) spin-resolved Wilson loops to show that unlike previously for β-MoTe_2 (SN 9), α-BiBr exhibits a bulk topological spin gap over a large range of spin resolution directions $\hat{\mathbf{n}}$, and hosts both spin-stable 3D quantum spin Hall insulator (QSHI) states as well as, remarkably, the $\mathcal{T}$-doubled axion insulating (T-DAXI) state introduced in this work. Lastly, in SN 10 C, we will numerically compute the bulk intrinsic contribution to the spin Hall conductivity (per layered unit cell) in α-BiBr, which we find to be nearly quantized in the 3D QSHI regime, and nearly vanishing for the spin-stable T-DAXI state.

A. Details of Density Functional Theory Calculations on α-BiBr

α-BiBr crystallizes in a centrosymmetric structure that respects the symmetries of nonmagnetic SG $C2/m1'$ (#12.59). Each primitive (unit) cell of α-BiBr contains eight Bi atoms and eight Br atoms [SFig. 37(a)]. The primitive lattice vectors of α-BiBr are given by

$$\begin{aligned}
\mathbf{a}_1 &= (6.5320001\ \text{Å})\hat{\mathbf{x}} - (2.1689999\ \text{Å})\hat{\mathbf{y}}, \\
\mathbf{a}_2 &= (6.5320001\ \text{Å})\hat{\mathbf{x}} + (2.1689999\ \text{Å})\hat{\mathbf{y}}, \\
\mathbf{a}_3 &= -(6.0057388\ \text{Å})\hat{\mathbf{x}} + (19.1409210\ \text{Å})\hat{\mathbf{z}},
\end{aligned} \tag{10.1}$$

SG $C2/m1'$ (#12.59) is generated by C-face-centered 3D lattice translations [SEq. (10.1)], as well as C_{2y} (180° rotation about the Cartesian y axis), $\mathcal{I}$, and $\mathcal{T}$ symmetries. In particular, C_{2y} and $\mathcal{I}$ act on the lattice vectors $\mathbf{a}_i$ as

$$C_{2y} : (\mathbf{a}_1, \mathbf{a}_2, \mathbf{a}_3) \to (-\mathbf{a}_2, -\mathbf{a}_1, -\mathbf{a}_3), \tag{10.2}$$

$$\mathcal{I} : (\mathbf{a}_1, \mathbf{a}_2, \mathbf{a}_3) \to (-\mathbf{a}_1, -\mathbf{a}_2, -\mathbf{a}_3). \tag{10.3}$$

The primitive reciprocal lattice vectors $\mathbf{G}_i$ [SFig. 37(b)] dual to the lattice vectors $\mathbf{a}_i$ in SEq. (10.1) satisfy $\mathbf{a}_i \cdot \mathbf{G}_j = 2\pi\delta_{ij}$, and are specifically given by

$$\begin{aligned}
\mathbf{G}_1 &= \frac{2\pi\left(\mathbf{a}_2 \times \mathbf{a}_3\right)}{\mathbf{a}_1 \cdot \left(\mathbf{a}_2 \times \mathbf{a}_3\right)} = 2\pi\left[\left(0.07654623\ \text{Å}^{-1}\right)\hat{\mathbf{x}} - \left(0.23052099\ \text{Å}^{-1}\right)\hat{\mathbf{y}} + \left(0.02401748\ \text{Å}^{-1}\right)\hat{\mathbf{z}}\right], \\
\mathbf{G}_2 &= \frac{2\pi\left(\mathbf{a}_3 \times \mathbf{a}_1\right)}{\mathbf{a}_2 \cdot \left(\mathbf{a}_3 \times \mathbf{a}_1\right)} = 2\pi\left[\left(0.07654623\ \text{Å}^{-1}\right)\hat{\mathbf{x}} + \left(0.23052099\ \text{Å}^{-1}\right)\hat{\mathbf{y}} + \left(0.02401748\ \text{Å}^{-1}\right)\hat{\mathbf{z}}\right], \\
\mathbf{G}_3 &= \frac{2\pi\left(\mathbf{a}_1 \times \mathbf{a}_2\right)}{\mathbf{a}_3 \cdot \left(\mathbf{a}_1 \times \mathbf{a}_2\right)} = 2\pi\left[0.05224409\ \text{Å}^{-1}\right]\hat{\mathbf{z}}.
\end{aligned} \tag{10.4}$$

To provide symmetry intuition for the $\mathbf{k}$-space electronic structure and spin-orbital texture of α-BiBr, we note that C_{2y} and $\mathcal{I}$ act on the primitive reciprocal lattice vectors $\{\mathbf{G}_1, \mathbf{G}_2, \mathbf{G}_3\}$ in SEq. (10.4) as

$$\begin{aligned}
C_{2y} &: (\mathbf{G}_1, \mathbf{G}_2, \mathbf{G}_3) \to (-\mathbf{G}_2, -\mathbf{G}_1, -\mathbf{G}_3), \\
\mathcal{I} &: (\mathbf{G}_1, \mathbf{G}_2, \mathbf{G}_3) \to (-\mathbf{G}_1, -\mathbf{G}_2, -\mathbf{G}_3).
\end{aligned} \tag{10.5}$$

Because SG $C2/m1'$ (#12.59) is C-face centered, and not primitive monoclinic, α-BiBr is sometimes instead characterized by its conventional (primitive supercell) lattice vectors [SFig. 37(a)]

$$\begin{aligned}
\mathbf{a} &= \mathbf{a}_1 + \mathbf{a}_2 = a\hat{\mathbf{x}}, \\
\mathbf{b} &= -\mathbf{a}_1 + \mathbf{a}_2 = b\hat{\mathbf{y}}, \\
\mathbf{c} &= \mathbf{a}_3 = c\cos\beta\hat{\mathbf{x}} + c\sin\beta\hat{\mathbf{z}},
\end{aligned} \tag{10.6}$$

where a, b, and c denote the conventional-cell lattice parameters [268]

$$a = |\mathbf{a}| = 13.0640002\ \text{Å},\ b = |\mathbf{b}| = 4.3379998\ \text{Å},\ c = |\mathbf{c}| = 20.0610009\ \text{Å}. \tag{10.7}$$

In SEq. (10.6), $\beta \approx 107.41999821484573°$ is the angle between $\mathbf{a}$ and $\mathbf{c}$ [268]. The conventional reciprocal lattice vectors of α-BiBr are correspondingly given by [52]

$$\begin{aligned}
\mathbf{a}^* &= \frac{2\pi}{a}\left(\hat{\mathbf{x}} - \cot\beta\hat{\mathbf{z}}\right), \\
\mathbf{b}^* &= \frac{2\pi}{b}\hat{\mathbf{y}}, \\
\mathbf{c}^* &= \frac{2\pi}{c}\csc\beta\hat{\mathbf{z}}.
\end{aligned} \tag{10.8}$$

In order to analyze the spin-resolved bulk topology, we first use DFT incorporating the effects of SOC to compute the electronic band structure of α-BiBr. We specifically performed first-principles calculations within the DFT framework using the PAW method [246, 247] as implemented in VASP [248, 249]. In our DFT calculations, we adopted the PBE generalized gradient approximation exchange-correlations functional [250], and SOC was incorporated self-consistently. The cutoff energy for the plane-wave expansion was 400 eV, and $0.03 \times 2\pi\ \text{Å}^{-1}$ $\mathbf{k}$-point sampling grids were used in the self-consistent process. In SFig. 37(c), we show the ab-initio band structure (labeled as "DFT") for α-BiBr computed along high-symmetry BZ lines connecting TRIM points labeled using the Bilbao Crystallographic Server convention for SG $C2/m1'$ (#12.59) (see SFig. 37(b) and SRefs. [43, 243–245, 251–253]). In our topological (Wilson-loop, see SN 4 C) analysis of α-BiBr below, we will used reduced $\mathbf{k}$ coordinates (k_1, k_2, k_3) defined by $k_i = \mathbf{k} \cdot \mathbf{a}_i$, such that:

$$\mathbf{k} = \frac{1}{2\pi}\left(k_1\mathbf{G}_1 + k_2\mathbf{G}_2 + k_3\mathbf{G}_3\right), \tag{10.9}$$

where each $\mathbf{G}_i$ is defined in SEq. (10.4). In the reduced $\mathbf{k} = (k_1, k_2, k_3)$ coordinates of SEq. (10.9), the TRIM points in α-BiBr lie at the positions [SFig. 37(b)]:

$$V = (\pi, 0, 0),\ L = (\pi, 0, -\pi),\ A = (0, 0, \pi),\ \Gamma = (0, 0, 0),\ M = (\pi, \pi, -\pi),\ Y = (\pi, \pi, 0). \tag{10.10}$$

To analyze the spin-resolved band topology of α-BiBr, we next constructed a symmetric, Wannier-based tight-binding model fit to the electronic structure of α-BiBr obtained from our DFT calculations. We specifically constructed symmetric Wannier functions for the bands near E_F in α-BiBr by using the Wannier90 package [254] for the Bi $6p$ and the Br $4p$ orbitals, and then performing a subsequent SG symmetrization using WannierTools [255]. Here and below,

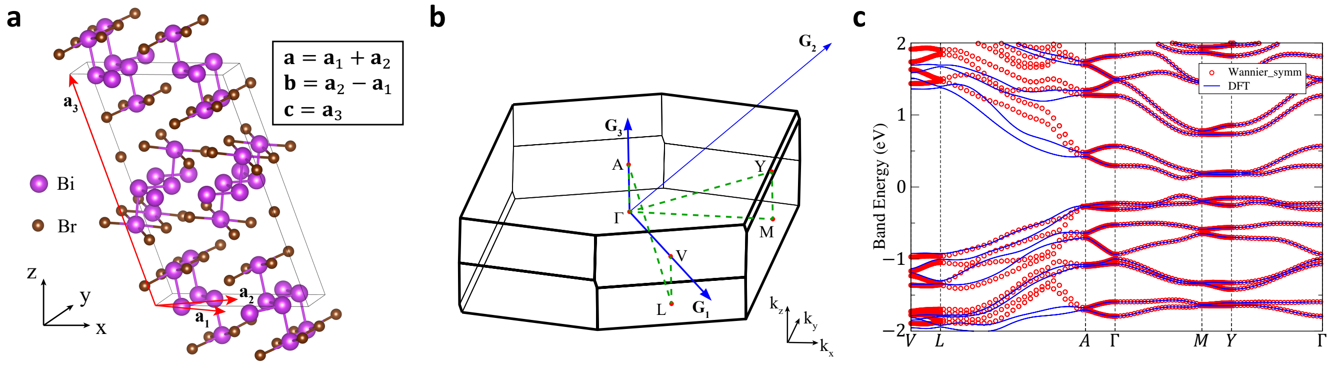

Supplementary Figure 37. First-principles and Wannier-based tight-binding electronic structure of α-BiBr. (a) The crystal structure of α-BiBr, which respects the symmetries of SG $C2/m1'$ (#12.59). There are eight Bi atoms and eight Br atoms per primitive (unit) cell. However, because α-BiBr is C-face-centered, it is also frequently characterized by its larger conventional primitive monoclinic supercell. In (a), the red (black) arrows denote the primitive-cell lattice (Cartesian-unit) vectors [SEq. (10.1)] and the inset box provides the relationship between the primitive lattice vectors $\mathbf{a}_{1,2,3}$ and the conventional supercell lattice vectors $\mathbf{a}$, $\mathbf{b}$, and $\mathbf{c}$ [SEq. (10.6)]. (b) The bulk BZ for α-BiBr; the primitive reciprocal lattice vectors $\mathbf{G}_i$ (blue) arrows are given in SEq. (10.4), and importantly differ from the Cartesian reciprocal unit vectors (black arrows). In (b), the TRIM points are labeled using the convention of the Bilbao Crystallographic Server [43, 243–245, 251–253], and their specific coordinates in reduced $\mathbf{k}$ units are provided in SEq. (10.10). (c) The DFT-obtained ("DFT," blue lines) and symmetric Wannier-based ("Wannier_symm," red circles) electronic band structure of α-BiBr plotted along the green dashed $\mathbf{k}$-path in (b). Although there are some qualitative discrepancies between the DFT and Wannier-based tight-binding band structures along $\overline{LA}$ in (c), the two band structures are otherwise in quite close agreement, and we have confirmed that α-BiBr exhibits the same band ordering and bulk stable topology at E_F (set to zero) in both calculations. We will hence employ the Wannier-based tight-binding model shown with red circles in (c) to below compute the spin-resolved topology (SN 10 B) and spin-electromagnetic response (SN 10 C) of α-BiBr.

we denote the tight-binding Hamiltonian of the Wannier-based tight-binding model as H_{BiBr}. The single-particle Hilbert space of H_{BiBr} consists of 48 spinful Wannier functions per primitive (unit) cell; the Bloch Hamiltonian $[H_{\text{BiBr}}(\mathbf{k})]$ is therefore a 96×96 matrix, To reduce the computational resources required for our spin-resolved and Wilson loop tight-binding calculations, we have truncated $[H_{\text{BiBr}}(\mathbf{k})]$ to only contain hopping terms with an absolute magnitude greater than or equal to 0.001 eV. In SFig. 37(c) we show the band structure of $[H_{\text{BiBr}}(\mathbf{k})]$ in the vicinity of E_F using red circles (denoted as "Wannier_symm") overlaying the DFT-obtained electronic band structure (blue lines, denoted as "DFT"). We have confirmed that the truncated Wannier-based tight-binding model exhibits the same band ordering and qualitative features as the first-principles electronic structure.

In our DFT calculations, α-BiBr is an insulator with 64 occupied spinful valence bands, which appear in doubly-degenerate pairs due to bulk $\mathcal{I} \times \mathcal{T}$ symmetry [178]. In SFig. 38(a), we again plot the band structure of the Wannier-based tight-binding model $[H_{\text{BiBr}}(\mathbf{k})]$, coloring the highest occupied (lowest unoccupied) pair of bands in blue (red). To confirm that the truncated form of $[H_{\text{BiBr}}(\mathbf{k})]$ exhibits a band gap at E_F at all $\mathbf{k}$ points, we have computed the energy gap $\Delta(\mathbf{k})$ between the 64th (highest occupied valence) and 65th (lowest unoccupied conduction) bands of $[H_{\text{BiBr}}(\mathbf{k})]$ using a $100 \times 100 \times 100$ grid uniformly spaced over the 3D BZ in the reduced $\mathbf{k}$ coordinates (k_1, k_2, k_3) defined in SEq. (10.9) and the surrounding text. As shown in SFig. 38(d), we find that the band gap at E_F at all $\mathbf{k}$ points is nonzero, and takes values greater than $\Delta(\mathbf{k}) \approx 0.24196355481095755$ eV. As a final test that $[H_{\text{BiBr}}(\mathbf{k})]$ is gapped at E_F, we further performed a numerical Nelder-Mead minimization [259] on the direct energy band gap $\Delta(\mathbf{k})$ using uniformly sampled $20 \times 20 \times 20$ $\mathbf{k}$ grids (again in reduced $\mathbf{k}$ coordinates) as initial points of minimization. For the truncated form of $[H_{\text{BiBr}}(\mathbf{k})]$, we found after 8000 Nelder-Mead minimizations that $\Delta(\mathbf{k}) \geq 0.24181320342543444$eV, essentially identical to the value obtained from uniform BZ sampling [SFig. 38(d)]. Hence for our calculations below of the spin-resolved topology (SN 10 B) and spin-electromagnetic response (SN 10 C) of α-BiBr, we have established that the projector onto the occupied (valence) bands, $[P(\mathbf{k})] = \sum_{n=1}^{64} |u_{n,\mathbf{k}}\rangle\langle u_{n,\mathbf{k}}|$ where $|u_{n,\mathbf{k}}\rangle$ is the nth eigenvector of $[H_{\text{BiBr}}(\mathbf{k})]$, is numerically well-defined over the full 3D BZ.

B. Spin-Resolved Topology of α-BiBr

In this section we will analyze the spin-resolved topology of the candidate helical HOTI α-BiBr. As we will show below, we find that α-BiBr hosts a bulk spin gap over a large range of spin resolution directions $\hat{\mathbf{n}}$, and specifically

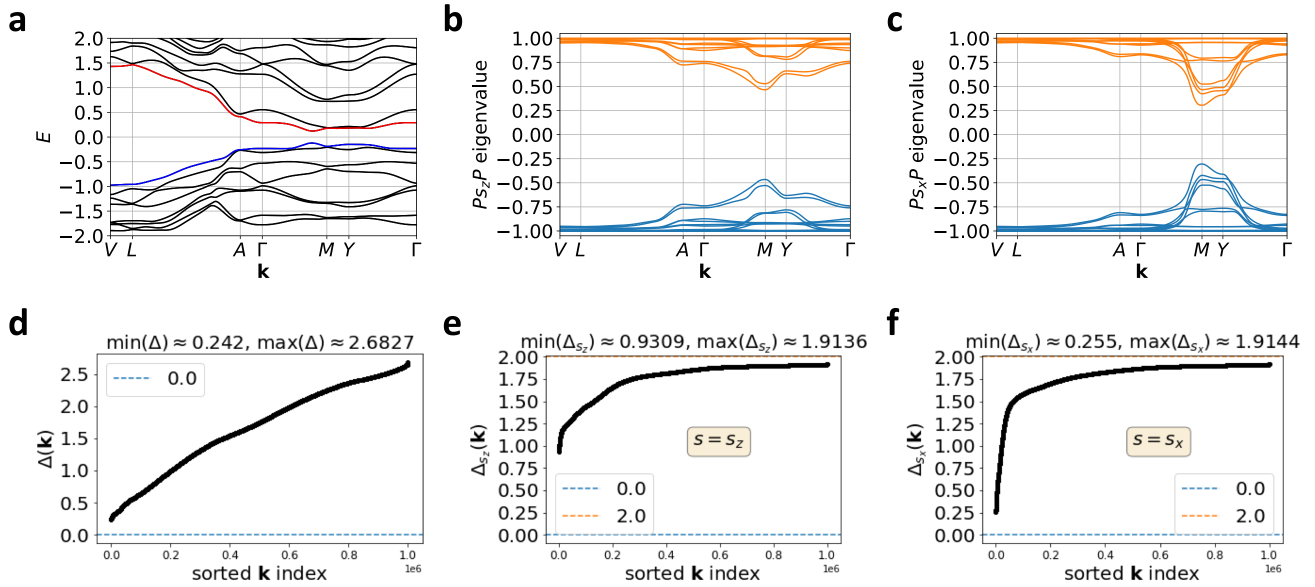

Supplementary Figure 38. Energy bands, energy gaps, PsP spin bands, and PsP spin gaps for the Wannier-based tight-binding model of α-BiBr. (a) Tight-binding energy band structure along high-symmetry BZ lines in α-BiBr [dashed green lines in SFig. 37(b)]. In (a), the bands are doubly degenerate due to bulk $\mathcal{I}\times\mathcal{T}$ symmetry [178], and the highest valence (lowest conduction) bands are colored in blue (red). (b,c) Respectively the Ps_zP and Ps_xP band structures computed along the same high-symmetry BZ lines as the electronic band structure in (a), with the positive (negative) spin bands colored in orange (blue). (d) Direct energy gaps $\Delta(\mathbf{k})$ of the Wannier-based tight-binding model in (a) at $\mathbf{k}$ points sampled from a uniformly spaced [in the reduced $\mathbf{k}$ coordinates defined in SEq. (10.9) and the surrounding text] $100\times 100\times 100$ grid over the 3D BZ of α-BiBr. We find that the direct energy gap takes the minimal value $\Delta(\mathbf{k})\approx 0.24196355481095755$ eV. (e,f) Respectively the direct Ps_zP and Ps_xP spin gaps $\Delta_s(\mathbf{k})$ (in the units of $\hbar/2$) computed over the uniform (in reduced coordinates) $\mathbf{k}$-grid employed in (d). For the spin direction $\hat{\mathbf{n}}=\hat{\mathbf{z}}$ in panel (e) [$\hat{\mathbf{n}}=\hat{\mathbf{x}}$ in panel (f)], we find that the direct spin gap takes the minimal value $\Delta_{s_z}(\mathbf{k})\approx 0.9309028798325673$ [$\Delta_{s_x}(\mathbf{k})\approx 0.2550432063802285$]. The calculations detailed in this figure were performed using the freely available Python package NESTED_AND_SPIN_RESOLVED_WILSON_LOOP [97], which represents an extension of the PythTB open-source Python tight-binding package [171] that was implemented and utilized for the preparation of SRefs. [8, 15] and the present work.

hosts both 3D QSHI (*e.g.* for $\hat{\mathbf{n}}=\hat{\mathbf{z}}$) and T-DAXI (*e.g.* for $\hat{\mathbf{n}}=\hat{\mathbf{x}}$) spin-stable states.

Spin Spectrum of α-BiBr. We will begin by computing the spin gap of α-BiBr over the full range of spin resolution directions $\hat{\mathbf{n}}$. The spin band structure is defined as the eigenspectrum of $PsP\equiv[P(\mathbf{k})]s[P(\mathbf{k})]$ computed as a function of $\mathbf{k}$. As previously with β-$MoTe_2$ in SN 9 B, α-BiBr has both $\mathcal{I}$ and spinful $\mathcal{T}$ symmetries. Hence, the PsP spectrum of α-BiBr exhibits an antiunitary chiral symmetry due to the relation

$$[\mathcal{I}][\mathcal{T}][P(\mathbf{k})]s[P(\mathbf{k})]([\mathcal{I}][\mathcal{T}])^{-1}=-[P(\mathbf{k})]s[P(\mathbf{k})], \tag{10.11}$$

where $[\mathcal{I}]$ and $[\mathcal{T}]$ are the matrix representatives of $\mathcal{I}$ and spinful $\mathcal{T}$ symmetries acting on the 96×96 Wannier-based tight-binding Bloch Hamiltonian matrix $[H_{\mathrm{BiBr}}(\mathbf{k})]$ that we obtained from DFT calculations of the electronic structure of α-BiBr (SN 10 A), and where $[P(\mathbf{k})]$ denotes the matrix projector $[P(\mathbf{k})]=\sum_{n=1}^{64}|u_{n,\mathbf{k}}\rangle\langle u_{n,\mathbf{k}}|$ onto the 64 occupied (valence) bands of α-BiBr. As discussed in SN 2 B, the presence of bulk $\mathcal{I}\times\mathcal{T}$ symmetry [SEq. (10.11)] further implies that

$$\mathrm{Spec}\left([P(\mathbf{k})]s[P(\mathbf{k})]\right)=-\mathrm{Spec}\left([P(\mathbf{k})]s[P(\mathbf{k})]\right), \tag{10.12}$$

where $\mathrm{Spec}(\mathcal{O})$ denotes the spectrum of the operator $\mathcal{O}$. From SEq. (10.12), we then again define the spin gap function $\Delta_s(\mathbf{k})$ as the difference between the smallest positive and the largest negative PsP eigenvalues at each $\mathbf{k}$ point.

Before performing the more intensive calculation of the global (direct) spin gap for each spin resolution direction $\hat{\mathbf{n}}$, we have first computed the full spin spectrum (band structure) of α-BiBr for the high-symmetry $\hat{\mathbf{n}}=\hat{\mathbf{z}}$ and $\hat{\mathbf{n}}=\hat{\mathbf{x}}$ spin directions. As shown in SFig. 38(b,c), we find that the Ps_zP and Ps_xP spin spectra of α-BiBr are gapped along

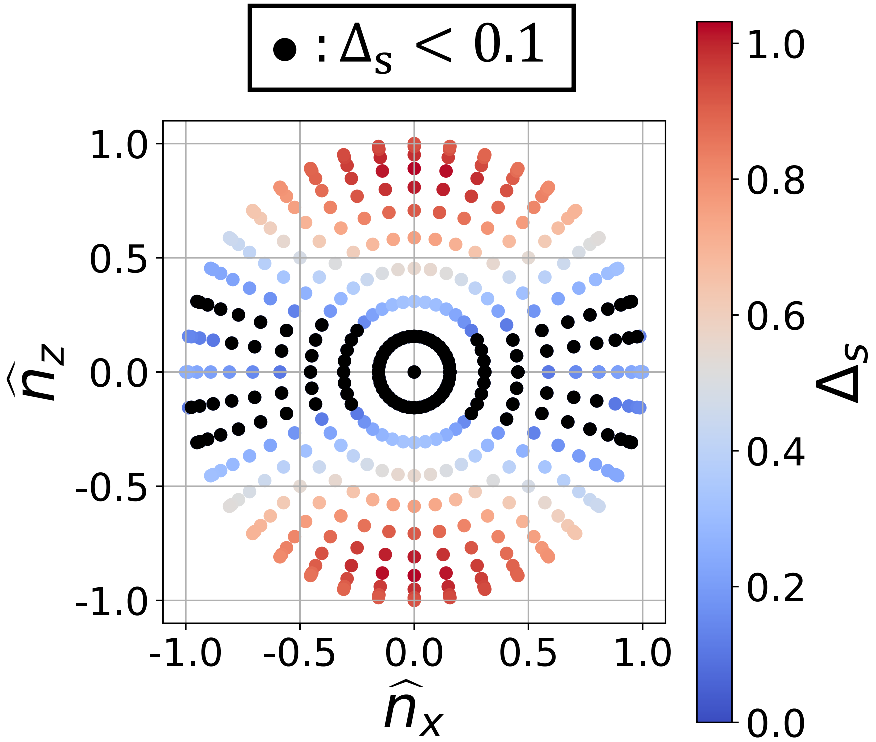

Supplementary Figure 39. Numerical spin gap $\Delta_{s=\hat{\mathbf{n}}\cdot\mathbf{s}}$ as a function of the spin resolution direction $\hat{\mathbf{n}}$ for α-BiBr. The angular parameterization of $\hat{\mathbf{n}}$ is defined in SEq. (10.13), and our calculations were performed over the spin-direction hemisphere of $\hat{\mathbf{n}}$ defined by $\vartheta \in [0, 0.5\pi]$ and $\phi \in [0, 2\pi]$. For our calculations, the angular variables (ϑ, ϕ) were respectively sampled using the numerical resolution of $\Delta\vartheta = 0.05\pi$ and $\Delta\phi = 0.05\pi$. By performing a Nelder-Mead minimization [259] on the spin gap function $\Delta_s(\mathbf{k})$ taking as the initial points 100 $\mathbf{k}$ points randomly sampled from the 3D BZ in the reduced $\mathbf{k}$ coordinates (k_1, k_2, k_3) defined in SEq. (10.9), we define for each $\hat{\mathbf{n}}$ the numerical spin gap $\Delta_{s=\hat{\mathbf{n}}\cdot\mathbf{s}}$ as the minimal value (in the units of $\hbar/2$) of the 100 minimization results for the fixed value of $\hat{\mathbf{n}}$. Unlike previously for β-$MoTe_2$ [SFig. 34], we find that α-BiBr exhibits a significant spin gap ($\Delta_s > 0.1$) for most values of $\hat{\mathbf{n}}$, and becomes larger than 0.8 (40% of the maximum possible value $\Delta_s = 2$) over a significant range of spin resolution directions roughly centered around $\hat{\mathbf{n}} = \pm\hat{\mathbf{z}}$ (light and dark red circles). We find specifically that the global spin gap in α-BiBr peaks at a large value [$\Delta_s \approx 0.9479813926905263$] and lies at $\hat{\mathbf{n}} = (\hat{n}_x, \hat{n}_y, \hat{n}_z) = \pm(-0.2486898871648553, 0, 0.968583161128631)$, which is surprisingly oriented within $\approx 3.019998214845685$ degrees of the $\mathbf{a}_3 \parallel \mathbf{c}$ lattice vector [see SEqs. (10.1) and (10.6) and SFig. 37(a)]. This indicates that the bulk spin-orbital texture in α-BiBr is dominated by contributions that are almost entirely polarized along the $\mathbf{a}_3 \parallel \mathbf{c}$ axis. We note that there also exists a narrower pair of spin-gapped region with smaller values of Δ_s centered around $\hat{\mathbf{n}} = \pm\hat{\mathbf{x}}$ (light blue circles), and that in particular $\Delta_s(\mathbf{k}) \geq 0.2550432063802285$ for $\hat{\mathbf{n}} = \pm\hat{\mathbf{x}}$ [see SFig. 38(f)]. As we will show later in this section, all four spin-gapped regions of α-BiBr exhibit nontrivial spin-resolved topology, with the $\pm\hat{\mathbf{z}}$- ($\pm\hat{\mathbf{x}}$-) centered regions specifically hosting 3D QSHI (T-DAXI) spin-stable states. The calculations detailed in this figure were performed using the freely available Python package NESTED_AND_SPIN_RESOLVED_WILSON_LOOP [97], which represents an extension of the PythTB open-source Python tight-binding package [171] that was implemented and utilized for the preparation of SRefs. [8, 15] and the present work.

high-symmetry BZ lines. Next, to search for the existence of spin gap closures at lower-symmetry $\mathbf{k}$ points in the 3D BZ interior (*e.g.* spin-Weyl points), we computed the local spin gap $\Delta_s(\mathbf{k})$ over a $100 \times 100 \times 100$ grid uniformly spaced over the 3D BZ in the reduced $\mathbf{k}$ coordinates (k_1, k_2, k_3) defined in SEq. (10.9). For both $\hat{\mathbf{n}} = \hat{\mathbf{z}}$ and $\hat{\mathbf{n}} = \hat{\mathbf{x}}$, we observe a nonzero $\Delta_s(\mathbf{k})$ at all sampled $\mathbf{k}$ points, with the specific local spin gap values of $\Delta_{s_z}(\mathbf{k}) \geq 0.9309028798325673$ for $\hat{\mathbf{n}} = \hat{\mathbf{z}}$ [SFig. 38(e)] and $\Delta_{s_x}(\mathbf{k}) \geq 0.2550432063802285$ for $\hat{\mathbf{n}} = \hat{\mathbf{x}}$ [SFig. 38(f)].

To gain a more comprehensive understanding of the spin gap structure in α-BiBr, we next computed the global (minimum) spin gap $\Delta_s \equiv \min_{\mathbf{k}\in\mathrm{BZ}}[\Delta_s(\mathbf{k})]$ for each spin direction $\hat{\mathbf{n}}$ in the $P(\hat{\mathbf{n}}\cdot\mathbf{s})P$ spectrum of α-BiBr. As we will show below, we find that unlike β-$MoTe_2$ (SN 9 B), α-BiBr in fact hosts a topological spin gap over a large range of $\hat{\mathbf{n}}$. We begin by re-expressing the spin direction $\hat{\mathbf{n}}$ as a 3D unit vector parameterized in rotated spherical coordinates as

$$\hat{\mathbf{n}} = (\sin\vartheta\cos\phi, \cos\vartheta, -\sin\vartheta\sin\phi). \tag{10.13}$$

The parameterization in SEq. (10.13) is chosen such that $(\vartheta, \phi) = (0, 0)$, $(0.5\pi, 0)$, and $(0.5\pi, -0.5\pi)$ respectively

correspond to the Cartesian unit vectors $\hat{\mathbf{y}}$, $\hat{\mathbf{x}}$, and $\hat{\mathbf{z}}$, a choice that is motivated by the C_{2y} rotation symmetry of α-BiBr [SEq. (10.5)]. For each (ϑ,ϕ), we then performed a Nelder-Mead minimization [259] on the spin gap function $\Delta_s(\mathbf{k})$ taking for the initial points 100 $\mathbf{k}$ points randomly sampled from the 3D BZ in the reduced $\mathbf{k}$ coordinates (k_1,k_2,k_3) defined in SEq. (10.9). We next define the *numerical spin gap* Δ_s as the minimal value of the 100 minimization results for each spin direction $\hat{\mathbf{n}}$. In SFig. 39, we show the numerical spin gap Δ_s (in the units of $\hbar/2$) for $\hat{\mathbf{n}}$ (projected into the xz plane) sampled over the spin hemisphere defined by $\vartheta \in [0,0.5\pi]$ and $\phi \in [0,2\pi]$ [SEq. (10.13)] using the angular resolutions $\Delta\vartheta = 0.05\pi$ and $\Delta\phi = 0.05\pi$. Such a sampling of (ϑ,ϕ) specifically corresponds to the upper hemisphere of a unit sphere with the north pole is identified as $\hat{\mathbf{y}}$. Because $\Delta_s = \Delta_{-s}$ due to $\mathcal{T}$ symmetry, this sampling of the angular variables (ϑ,ϕ) contains all independent values of the numerical spin gap Δ_s over the full sphere of spin resolution directions $\hat{\mathbf{n}}$.

As shown in SFig. 39, the numerical spin gap $\Delta_s > 0.1$ for most values of $\hat{\mathbf{n}}$, and is larger than 0.8 (40% of the maximum possible value $\Delta_s = 2$) over a significant range of spin resolution directions roughly centered around $\hat{\mathbf{n}} = \pm\hat{\mathbf{z}}$ (light and dark red circles in SFig. 39). For our subsequent calculations, it is important to note that there also exists a narrower pair of spin-gapped regions in α-BiBr in the vicinity of $\hat{\mathbf{n}} = \pm\hat{\mathbf{x}}$ with smaller values of Δ_s (light blue circles in SFig. 39) relative to those in the vicinity of $\hat{\mathbf{n}} = \pm\hat{\mathbf{z}}$. The $\pm s_z$ spin gap in α-BiBr is hence large ($\Delta_{s_z} \approx 0.9309028798325673$), and is much larger than the $\pm s_x$ spin gap ($\Delta_{s_x} \approx 0.2550432063802285$). This is consistent with earlier first-principles investigations of α-BiBr, which found the spin-electromagnetic (Rashba-Edelstein) response of its (010)-surface states to be strongly polarized in the z-direction relative to the x-direction [52]. We further crucially observe in SFig. 39 a pair of effectively spin-gapless ($\Delta_s < 0.1$) lines separating the four spin-gapped regions, suggesting the possibility that the $\hat{\mathbf{n}} = \pm\hat{\mathbf{z}}$ and $\hat{\mathbf{n}} = \pm\hat{\mathbf{x}}$ spin-gapped regions of α-BiBr are separated by spin-resolved topological phase transitions. Below, we will shortly confirm this intuition, finding specifically through (nested) spin-resolved Wilson-loop calculations that α-BiBr is a 3D QSHI [T-DAXI] for $\hat{\mathbf{n}} = \pm\hat{\mathbf{z}}$ [$\hat{\mathbf{n}} = \pm\hat{\mathbf{x}}$].

Overall, we find that the global spin gap in α-BiBr peaks at a large value ($\Delta_s \approx 0.9479813926905263$) and lies at $\hat{\mathbf{n}} = (\hat{n}_x,\hat{n}_y,\hat{n}_z) = \pm(-0.2486898871648553, 0, 0.968583161128631)$, which is surprisingly oriented within $\approx 3.019998214845685°$ of the lattice vector $\mathbf{a}_3$ [SEq. (10.1)]. This indicates that the bulk spin-orbital texture in α-BiBr is dominated by contributions that are almost entirely polarized along the $\mathbf{a}_3 \parallel \mathbf{c}$ lattice vector [SFig. 37(a)]. In 2D materials, such as superconducting transition-metal dichalcogenides, analogous spin-orbital textures polarized along high-symmetry crystallographic axes have also been observed, and have been termed "Ising SOC" [118, 269–272]. The appearance of a large spin gap nearly locked to a crystallographic axis in SFig. 39 suggests that it would be intriguing to investigate the microscopic mechanism of the SOC in α-BiBr in future theoretical studies, and to study the spin-resolved response of α-BiBr in future photoemission and transport experiments, which may exhibit an unusually high degree of spin polarization relative to other strongly spin-orbit-coupled 3D materials.

To conclude our analysis of the spin spectrum of α-BiBr, we last performed a Nelder-Mead minimization [259] for $\hat{\mathbf{n}} = \hat{\mathbf{y}}$ on the spin gap function $\Delta_s(\mathbf{k})$, taking for the initial points uniformly sampled $\mathbf{k}$ points drawn from $10\times10\times10$ grids in the 3D BZ in the reduced $\mathbf{k}$ coordinates (k_1,k_2,k_3) defined in SEq. (10.9). We find that the numerical spin gap $\Delta_{s_y} \approx 7.981038733049717\times10^{-6}$, such that the Ps_yP spectrum of α-BiBr can be identified as spin-gapless within numerical precision. By direct computation, we have confirmed that like β-$MoTe_2$ for all spin-resolution directions (SN 9 B), α-BiBr for $\hat{\mathbf{n}} = \hat{\mathbf{y}}$ is specifically a spin-Weyl semimetal with two spin-Weyl points within each half of the 3D BZ, as expected for a helical HOTI lying in the critical DSTI (spin-Weyl) regime that separates 3D QSHI and T-DAXI spin-stable states (see SN 4 D 3).

Spin-Resolved Topology of α-BiBr: Spin-Resolved Wilson Loop. Having established that α-BiBr hosts a bulk spin gap over a large range of spin resolution directions $\hat{\mathbf{n}}$, we will next focus on analyzing the bulk (spin-resolved) topology of α-BiBr from several perspectives. We begin by first investigating the partial Chern numbers $C^{\pm}_{\gamma_1}(\hat{\mathbf{n}},k_i)$ of the occupied 64 occupied valence bands in constant-k_i BZ planes ($i=1,2,3$). We first recall that if a 3D insulator hosts a global spin gap for a spin direction $\hat{\mathbf{n}}$, then $C^{\pm}_{\gamma_1}(\hat{\mathbf{n}},k_i)$ evaluated in *any* BZ plane of constant k_i will take the same values in all other BZ planes of constant k_i (keeping i fixed). This can be seen by recognizing that $C^{\pm}_{\gamma_1}(\hat{\mathbf{n}},k_i)$ can only change via the closing and reopening of a spin gap that manifests as a nodal degeneracy in the 3D spin spectrum with a nonvanishing partial chiral charge, such as a spin-Weyl point [see SN 3 E and 4 C 3]. In this section and below in SN 10 C we will, for simplicity, express the partial Chern vector $\boldsymbol{\nu}^{\pm}$ in the reduced coordinate of the reciprocal lattice vectors $\{\mathbf{G}_1,\mathbf{G}_2,\mathbf{G}_3\}$. As discussed in SN 3 C and 4 C 3, in spin-gapped 3D insulators, the partial Chern vectors $\boldsymbol{\nu}^{\pm}$ indicate the topological contributions $\sigma^H_{s=\hat{\mathbf{n}}\cdot\mathbf{s},\mathrm{top},ij}$ to the bulk *non-quantized* spin Hall conductivity $\sigma^H_{s=\hat{\mathbf{n}}\cdot\mathbf{s},ij}$ ($i\neq j$ and $i,j\in\{1,2,3\}$). As shown in SEq. (4.73), $\sigma^H_{s=\hat{\mathbf{n}}\cdot\mathbf{s},ij}$ specifically satisfies the relation

$$\sigma^H_{s=\hat{\mathbf{n}}\cdot\mathbf{s},\mathrm{top},ij} = \frac{e}{4\pi}\sum_{k=1}^{3}\epsilon_{ijk}\left[\sum_{l=1}^{3}(\nu_l^+ - \nu_l^-)\mathbf{G}_l\right]_k . \tag{10.14}$$

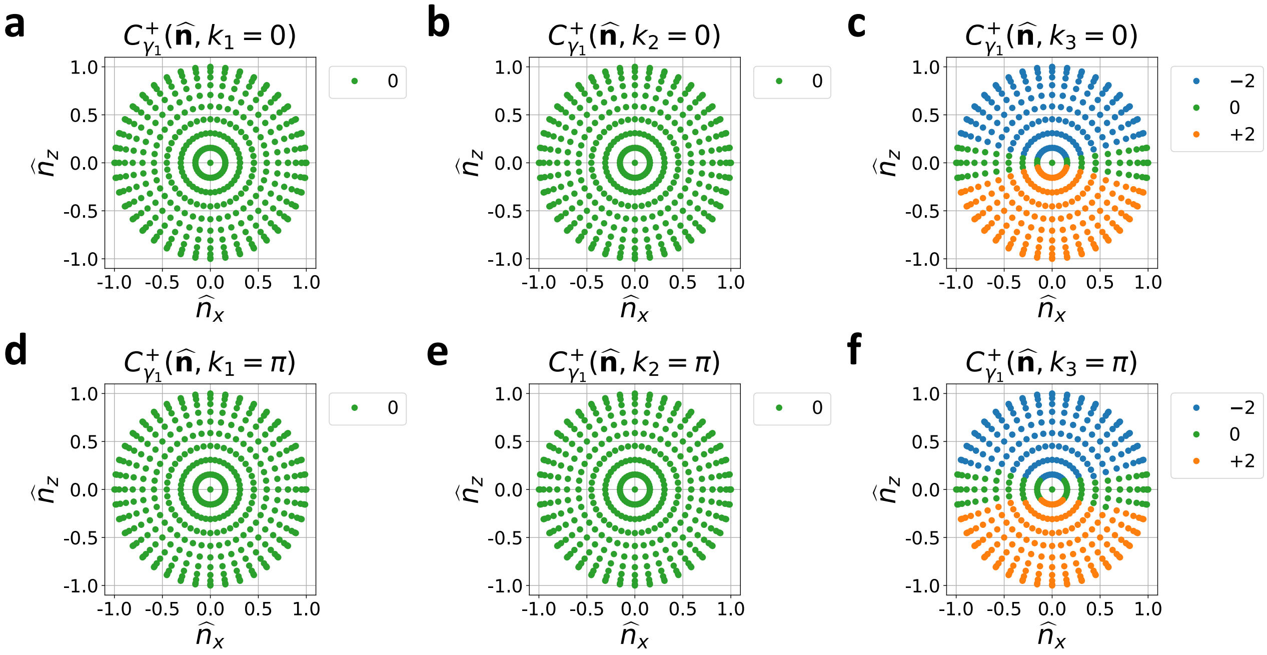

Supplementary Figure 40. Partial Chern numbers of the occupied bands in α-BiBr. In each panel, we show the partial Chern numbers $C^+_{\gamma_1}(\hat{\mathbf{n}}, k_i)$ in the high-symmetry ($\mathcal{T}$-invariant) $k_i = 0, \pi$ ($i = 1, 2, 3$) BZ planes. Because $\mathcal{T}$ symmetry enforces that $C^+_{\gamma_1}(-\hat{\mathbf{n}}, k_i) = -C^+_{\gamma_1}(\hat{\mathbf{n}}, k_i)$, and because $C^-_{\gamma_1}(\hat{\mathbf{n}}, k_i) = -C^+_{\gamma_1}(\hat{\mathbf{n}}, k_i)$ at $k_i = 0, \pi$, then we in this figure only show $C^+_{\gamma_1}(\hat{\mathbf{n}}, k_i)$ sampled over the positive spin hemisphere defined in SEq. (10.13) for which $\hat{\mathbf{n}} = \hat{\mathbf{y}}$ is taken to be the north pole. At each point in each panel in this figure, we show the value of the partial Chern number $C^+_{\gamma_1}(\hat{\mathbf{n}}, k_i)$ plotted at the position of $\hat{\mathbf{n}}$ projected onto the xz-plane. As shown in (a,b,d,e), $C^+_{\gamma_1}(\hat{\mathbf{n}}, k_i) = 0$ at all $\hat{\mathbf{n}}$ for $i = 1, 2$ and $k_i = 0, \pi$. Conversely for $k_3 = 0, \pi$, there are large regions of $\hat{\mathbf{n}}$ parameter space (centered around $\hat{\mathbf{n}} = \pm\hat{\mathbf{z}}$) in which the partial Chern numbers are nontrivial $C^+_{\gamma_1}(\hat{\mathbf{n}}, k_3) = \pm 2$. Though the regions with $C^+_{\gamma_1}(\hat{\mathbf{n}}, k_3) = \pm 2$ in (c,f) [blue and orange circles] appear identical, we note that there exist small numerical differences in the shape and range of the intermediate $C^+_{\gamma_1}(\hat{\mathbf{n}}, k_3) = 0$ regions in (c,f) [green circles]. Along with the nonvanishing global spin gap for most $\hat{\mathbf{n}}$ in α-BiBr established in SFig. 39, and the nontrivial (partial) SIs of α-BiBr (see SN 4 D 2 and SRefs. [50, 52, 53]), the data in this figure indicate that α-BiBr is a $\boldsymbol{\nu}^{\pm} = (\nu_1^{\pm}, \nu_2^{\pm}, \nu_3^{\pm}) = (0, 0, \mp 2)$ or $(0, 0, \pm 2)$ 3D QSHI over the large spin-gapped regions in (c,f) with $C^+_{\gamma_1}(\hat{\mathbf{n}}, k_3) = -2$ or $+2$, and is remarkably a T-DAXI for the smaller spin-gapped region in (c,f) with $C^+_{\gamma_1}(\hat{\mathbf{n}}, k_3) = 0$ [see SFig. 39 for comparison]. We will shortly confirm this conclusion by direct computation of the nested partial Wilson loop spectrum of α-BiBr. The calculations detailed in this figure were performed using the freely available Python package NESTED_AND_SPIN_RESOLVED_WILSON_LOOP [97], which represents an extension of the PythTB open-source Python tight-binding package [171] that was implemented and utilized for the preparation of SRefs. [8, 15] and the present work.

In $\mathcal{T}$-invariant (nonmagnetic) insulators like α-BiBr, $\nu_i^- = -\nu_i^+$, such that

$$\sigma^H_{s=\hat{\mathbf{n}}\cdot\mathbf{s},\mathrm{top},ij} = \frac{2e}{4\pi}\sum_{k=1}^{3}\epsilon_{ijk}\left[\sum_{l=1}^{3}\nu_l^+\mathbf{G}_l\right]_k. \tag{10.15}$$

As introduced in this work, the partial Chern numbers $C^{\pm}_{\gamma_1}(\hat{\mathbf{n}}, k_i)$ can be numerically obtained via the winding numbers of the spin-resolved Wilson loop spectra of the occupied bands in constant-k_i BZ planes, here specifically $k_i = 0, \pi$ (see SN 3 C). As discussed above, if $C^{\pm}_{\gamma_1}(\hat{\mathbf{n}}, k_i)$ in the $k_i = 0, \pi$ planes take different values, then there must exist spin gap closing points with nonvanishing partial chiral charges (such as spin-Weyl points) between $k_i = 0, \pi$. Lastly as seen in SN 9 B, diagnosing the bulk spin-resolved topology via high-symmetry ($k_i = 0, \pi$) BZ-plane partial Chern numbers is only a numerically stable calculation when spin-gap closing points do not lie close to the $k_i = 0, \pi$ BZ planes. However, as shown earlier in this section [SFig. 39], α-BiBr is in fact spin-gapped for most values of the spin resolution direction $\hat{\mathbf{n}}$.

To compute the high-symmetry $k_i = 0, \pi$ partial Chern numbers of α-BiBr, we begin by again parameterizing $\hat{\mathbf{n}}$ using SEq. (10.13) with ϑ and ϕ sampled over the angular range $\vartheta \in [0, 0.5\pi]$ and $\phi \in [0, 2\pi]$ using the angular resolution $\Delta\vartheta = 0.05\pi$ and $\Delta\phi = 0.05\pi$. In SFig. 40, we show the partial Chern numbers for the occupied bands in α-BiBr $C^+_{\gamma_1}(\hat{\mathbf{n}}, k_i)$ computed over all $\hat{\mathbf{n}}$ for $k_i = 0, \pi$. We specifically in SFig. 40 only show the upper spin hemisphere

parameterized by (ϑ, ϕ) in SEq. (10.13), relying on the result that in the $\mathcal{T}$-invariant BZ planes $k_i = 0, \pi$,

$$C^{\pm}_{\gamma_1}(\hat{\mathbf{n}}, k_i) = -C^{\mp}_{\gamma_1}(\hat{\mathbf{n}}, k_i), \tag{10.16}$$

which implies that $C^{\pm}_{\gamma_1}(\hat{\mathbf{n}}, k_i) = -C^{\pm}_{\gamma_1}(-\hat{\mathbf{n}}, k_i)$ at $k_i = 0, \pi$, due the general property that $C^{\pm}_{\gamma_1}(\hat{\mathbf{n}}, k_i) = C^{\mp}_{\gamma_1}(-\hat{\mathbf{n}}, k_i)$.

As shown in SFig. 40(a,b,d,e), we find that

$$C^{\pm}_{\gamma_1}(\hat{\mathbf{n}}, k_1 = 0) = C^{\pm}_{\gamma_1}(\hat{\mathbf{n}}, k_1 = \pi) = C^{\pm}_{\gamma_1}(\hat{\mathbf{n}}, k_2 = 0) = C^{\pm}_{\gamma_1}(\hat{\mathbf{n}}, k_2 = \pi) = 0, \tag{10.17}$$

for all $\hat{\mathbf{n}}$ in α-BiBr. Conversely in the $k_3 = 0, \pi$ BZ planes, $C^{\pm}_{\gamma_1}(\hat{\mathbf{n}}, k_3 = 0)$ and $C^{\pm}_{\gamma_1}(\hat{\mathbf{n}}, k_3 = \pi)$ take nonvanishing values (± 2) over a large range of $\hat{\mathbf{n}}$ centered around $\hat{\mathbf{n}} = \hat{\mathbf{z}}$, and vanish (while remaining numerically stable) over a smaller range of $\hat{\mathbf{n}}$ centered around $\hat{\mathbf{n}} = \hat{\mathbf{x}}$, such that

$$C^{\pm}_{\gamma_1}(\hat{\mathbf{z}}, k_3 = 0) = C^{\pm}_{\gamma_1}(\hat{\mathbf{z}}, k_3 = \pi) = \mp 2, \tag{10.18}$$

$$C^{\pm}_{\gamma_1}(\hat{\mathbf{x}}, k_3 = 0) = C^{\pm}_{\gamma_1}(\hat{\mathbf{x}}, k_3 = \pi) = 0. \tag{10.19}$$

Along with the finite numerical spin gap Δ_s established in SFig. 39 for the spin resolution directions $\hat{\mathbf{n}}$ centered around $\hat{\mathbf{n}} = \hat{\mathbf{z}}$ and $\hat{\mathbf{n}} = \hat{\mathbf{x}}$, we conclude from SEqs. (10.17), (10.18), and (10.19) that α-BiBr hosts the partial weak Chern vectors

$$\begin{aligned} \boldsymbol{\nu}^{\pm} &= (0, 0, \mp 2) \text{ for } s_z \text{ (and surrounding } \hat{\mathbf{n}}), \\ \boldsymbol{\nu}^{\pm} &= (0, 0, 0) \text{ for } s_x \text{ (and surrounding } \hat{\mathbf{n}}). \end{aligned} \tag{10.20}$$

Because α-BiBr is a symmetry-indicated helical HOTI [50, 52, 53], SEq. (10.20), along with the s_z and s_x numerical spin gaps, imply that α-BiBr realizes a 3D QSHI (T-DAXI) state for $\hat{\mathbf{n}} = \hat{\mathbf{z}}$ ($\hat{\mathbf{n}} = \hat{\mathbf{x}}$), due to its bulk spin-resolved (partial) SIs (SN 4 D 2). We will below in this section shortly confirm this conclusion via the direct computation of the nested spin-resolved Wilson loop spectrum of α-BiBr. In SN 10 C, we will also compare the partial weak indices in SEq. (10.20) to the bulk intrinsic spin Hall conductivity in α-BiBr for s_z and s_x spins, which we will show provides a physically measurable signature of its spin-gapped bulk topology.

Higher-Order Spectral Flow in α-BiBr: Nested Wilson Loop Spectrum. We will next pause from analyzing spin-resolved quantities to compute the ordinary (nested) Wilson loop of α-BiBr. This calculation will provide us with a reference hybrid Wannier (nested Wilson) spectrum for the nested spin-resolved Wilson loop of α-BiBr, which will be computed later in this section. Furthermore as we will discuss below, confirming helical nested Wilson loop flow in the ab-initio-derived electronic structure of a candidate helical HOTI itself represents a significant result; such calculations remain exceedingly rare, with a noteworthy previous example being the nested Wilson loop identification of a non-symmetry-indicated helical HOTI state in γ-$MoTe_2$ [31]. Here and throughout this work, our (spin-resolved) nested Wilson loop calculations have been performed using the freely available Python package NESTED_AND_SPIN_RESOLVED_WILSON_LOOP [97], which represents an extension of the PythTB open-source Python tight-binding package [171] that was implemented and utilized for the preparation of SRefs. [8, 15], and was then greatly expanded for the present work.

To investigate the hybrid Wannier spectrum of α-BiBr, we first compute the k_3-directed Wilson loop eigenphases [Wannier band energies] $\gamma_1(k_1, k_2)$ for the 64 occupied (valence) bands [SFig. 41(a)]. The Wilson loop spectrum in [SFig. 41(a)] exhibits clear gaps at $\gamma_1(k_1, k_2) = \pm\pi/2 = \pm 0.5\pi$, reminiscent of the Wilson loop spectrum of the candidate helical HOTI β-$MoTe_2$ [31], whose spin-resolved topology was previously analyzed in SN 9 B. Following the procedure introduced in SRefs. [15, 31] and discussed in SN 4 E, we then divide the Wannier (Wilson) bands in SFig. 41(a) into two $\mathcal{I}$- and $\mathcal{T}$-symmetric groupings: an inner set centered around $\gamma_1 = 0$ and an outer set centered around $\gamma_1 = \pi$. We crucially further find that for all perpendicular momenta k_2, the k_3-directed Wannier spectrum of α-BiBr remains gapped in the vicinity of $\gamma_1 = \pm\pi/2$.

We may hence compute the $\mathcal{I}$- and $\mathcal{T}$-invariant *nested* Wilson spectrum of α-BiBr (see SRefs. [15, 31, 174] and SN 6). To perform the nested Wilson loop calculation, we first define the inner and outer Wannier- (Wilson-) band projectors as P_{in} and P_{out} [SFig. 41(a)]. We then separately compute the k_2-directed nested Wilson loop eigenvalues $\gamma_2(k_1)$ for the inner [SFig. 41(b)] and outer [SFig. 41(c)] Wannier bands. In both the inner and outer nested Wilson loop spectra in SFig. 41(b,c), we observe the odd helical winding characteristic of an $\mathcal{I}$- and $\mathcal{T}$-protected helical HOTI (see SRef. [31] and SN 4 E). Specifically, due to the correspondence between the Wilson spectrum and the hybrid Wannier spectrum (see SN 2 A and 3 A, and SRefs. [4, 14]), the nested Wilson spectra in SFig. 41(b,c) indicate that the hybrid Wannier spectrum of α-BiBr can be deformed without breaking $\mathcal{I}$ or $\mathcal{T}$ symmetry (or closing a bulk energy gap) to the hybrid Wannier spectrum of the minimal layer construction of a helical HOTI [33]. More specifically, the k_3-directed hybrid Wannier spectrum of α-BiBr can be symmetrically deformed without closing an energy gap to a hybrid Wannier spectrum consisting of 2D TI layers with normal vectors parallel to $\mathbf{a}_3$ pinned by $\mathcal{I}$ symmetry to

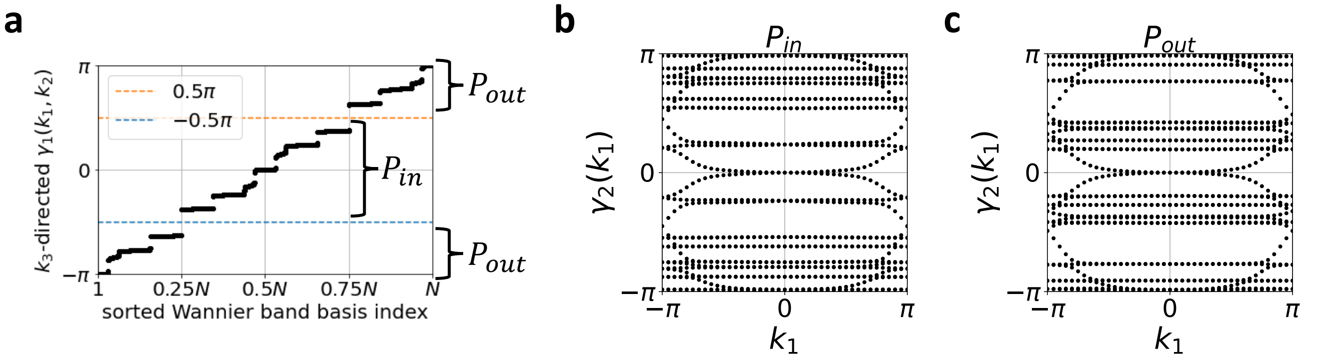

Supplementary Figure 41. Nested Wilson loop spectrum of α-BiBr. In this figure, we compute the Wilson loop (a) and nested Wilson loop (b,c) spectra of α-BiBr. The calculations shown in this figure were performed using a $41 \times 41 \times 41$ $\mathbf{k}$-grid uniformly spaced over the 3D BZ in the reduced $\mathbf{k}$ coordinates defined in SEq. (10.9). (a) The eigenphases of the k_3-directed [see SEq. (10.9)] P-Wilson loop spectrum of α-BiBr. Like that of the candidate helical HOTI β-MoTe$_2$ [31], the Wilson loop spectrum in (a) exhibits clear gaps at $\gamma_1 = \pm\pi/2 = 0.5\pi$. We have also confirmed that the gaps in the k_3-directed P-Wilson loop spectrum remain present for all values of k_2. We may hence define in (a) the $\mathcal{I}$- and $\mathcal{T}$-invariant (see SRefs. [15, 31, 174] and SN 6) nested Wilson projectors P_{in} and P_{out} [SEq. (4.23)]. (b,c) The k_2-directed nested P-Wilson loop spectrum (SN 4 A) of α-BiBr respectively computed over the inner (P_{in}) and outer (P_{out}) Wilson band projectors in (a). In both (b,c), the nested Wilson spectrum exhibits the characteristic odd helical winding of a $\mathcal{I}$- and $\mathcal{T}$-protected helical HOTI (see SRef. [31] and SN 4 E). The calculations detailed in this figure were performed using the freely available Python package NESTED_AND_SPIN_RESOLVED_WILSON_LOOP [97], which represents an extension of the PythTB open-source Python tight-binding package [171] that was implemented and utilized for the preparation of SRefs. [8, 15] and the present work.

the origin and $\mathbf{a}_3/2$, equivalent to that of the minimal layer construction of a $\mathcal{I}$- and $\mathcal{T}$-protected helical HOTI (see SN 4 D). We have hence shown, for the first time, that the hybrid Wannier spectrum of the candidate helical HOTI α-BiBr [50, 52, 53, 64, 68] exhibits higher-order helical spectral flow.

Spin-Resolved Topology of α-BiBr: Nested Spin-Resolved Wilson Loop. Having confirmed that α-BiBr hosts large spin-gapped regions in spin-direction ($\hat{\mathbf{n}}$) parameter space (SFig. 39), and that its occupied bands exhibit the characteristic nested Wilson spectrum of an $\mathcal{I}$- and $\mathcal{T}$-protected helical HOTI (SFig. 41), we will next directly apply the (nested) spin-resolved Wilson loop method developed in this work (SN 3 B and 4 B) to extract the spin-resolved topology of α-BiBr. First, earlier in this section, we showed that the spin spectrum of α-BiBr can be divided into four spin-gapped regions respectively centered around $\hat{\mathbf{n}} = \pm\hat{\mathbf{z}}$ and $\hat{\mathbf{n}} = \pm\hat{\mathbf{x}}$ (SFig. 39). For this reason, we will here focus on computing the spin-resolved topology of α-BiBr in just the $\hat{\mathbf{n}} = \hat{\mathbf{z}}$ and $\hat{\mathbf{n}} = \hat{\mathbf{x}}$ spin resolution directions, noting that the $\mathcal{I}$-protected spin-resolved topology of α-BiBr will be the same for other values of $\hat{\mathbf{n}}$ that are related to $\hat{\mathbf{n}} = \hat{\mathbf{z}}, \hat{\mathbf{x}}$ by (adiabatic) paths in $\hat{\mathbf{n}}$ parameter space along which the spin gap does not close (see SN 2 C and SFig. 39). As noted earlier in this section, the (nested) spin-resolved Wilson loop calculations detailed below were performed using the freely available Python package NESTED_AND_SPIN_RESOLVED_WILSON_LOOP [97], which was developed for SRefs. [8, 15] and the present work.

We begin by reconsidering the $\hat{\mathbf{n}} = \hat{\mathbf{z}}$ $P(\hat{\mathbf{n}} \cdot \mathbf{s})P$ $[Ps_zP]$ spectrum of α-BiBr. Because there is a spin gap at all $\mathbf{k}$ points for $\hat{\mathbf{n}} = \hat{\mathbf{z}}$ [SFig. 38(b,e)], then we may compute the spin-resolved $P_\pm$-Wilson loop on the positive and negative spin bands (SN 3 B). In SFig. 42(a) [SFig. 42(d)], we show the k_3-directed P_+- [P_--] Wilson loop spectrum for the positive [negative] s_z spin bands of α-BiBr. As with the ordinary k_3-directed P-Wilson loop in α-BiBr [SFig. 41(a)], the spin-resolved k_3-directed $P_\pm$-Wilson bands are gapped, and form $\mathcal{I}$-symmetric groupings (see SN 6 E) centered around $\gamma_1^\pm(k_1, k_2) = 0, \pi$ that are separated by pronounced spin-resolved Wilson gaps at $\gamma_1^\pm(k_1, k_2) = \pm\pi/2 = \pm 0.5\pi$. We have also numerically confirmed that the k_3-directed $P_\pm$-Wilson bands in α-BiBr remain gapped at $\gamma_1^\pm(k_1, k_2) = \pm\pi/2$ for all values of k_2, allowing us to consistently define $\mathcal{I}$-symmetric inner [$P_{in}^\pm$] and outer [$P_{out}^\pm$] s_z nested spin-resolved Wilson projectors [SFig. 42(a,d)]. Using $P_{in}^\pm$ and $P_{out}^\pm$, we then respectively compute the inner and outer k_2-directed nested spin-resolved Wilson loop (SN 4 B) of α-BiBr for both the positive and negative s_z spin bands, whose determinants $\text{Im}\left(\log\left(\det[\mathcal{W}_2^\pm(k_1)]\right)\right)$ are plotted in SFig. 42(b,c,e,f) as functions of the remaining crystal momentum k_1. For both the positive and negative spin bands, the inner and outer nested s_z spin-resolved Wilson bands exhibit the same odd winding numbers [SEq. (4.64)]. Specifically, we respectively find that $C^\pm_{\gamma_2,in} = \mp 1$ [$C^\pm_{\gamma_2,out} = \mp 1$] for the inner [outer] s_z $P_\pm$-Wilson bands in α-BiBr, employing the sign convention established in SEq. (3.24) in which the *negative* winding number of the k_2-directed nested spin-resolved Wilson loop as a function of k_1 gives the nested partial Chern

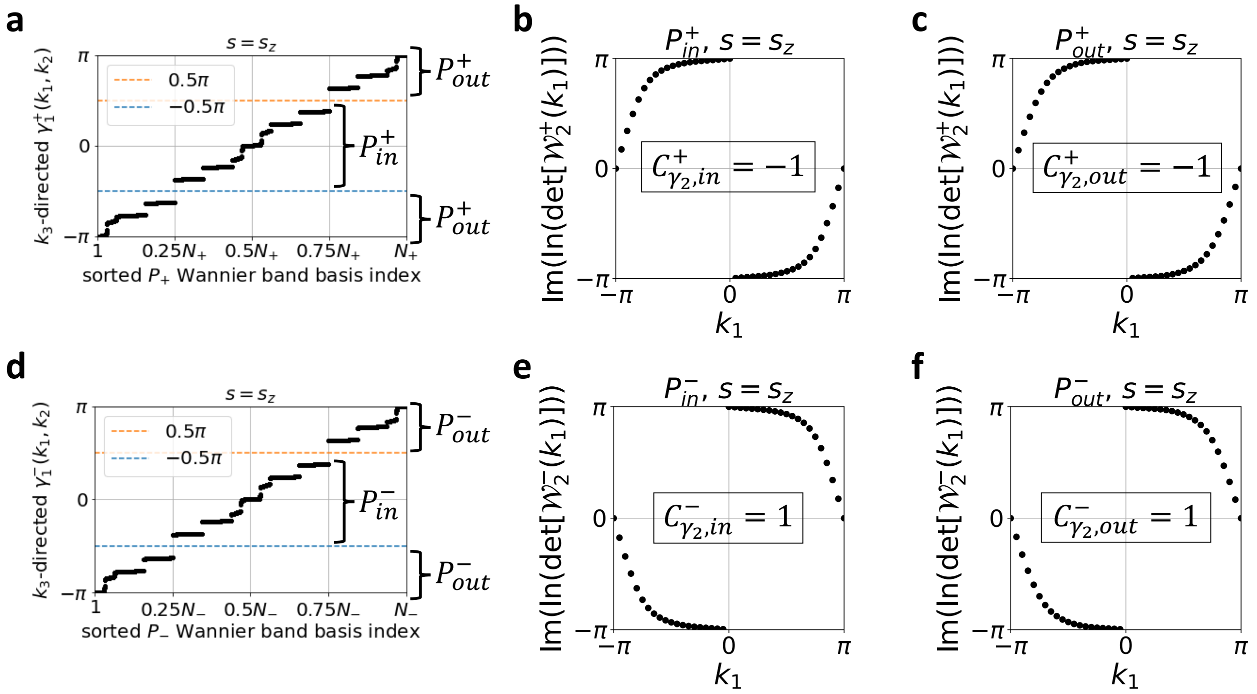

Supplementary Figure 42. $\hat{\mathbf{n}} = \hat{\mathbf{z}}$ nested spin-resolved Wilson loop spectrum of α-BiBr. The calculations shown in this figure were performed using a $41 \times 41 \times 41$ $\mathbf{k}$-grid uniformly spaced over the 3D BZ in the reduced $\mathbf{k}$ coordinates defined in SEq. (10.9). (a,d) Respectively the eigenphases of the k_3-directed [see SEq. (10.9)] s_z spin-resolved P_+- and P_--Wilson loop spectra of α-BiBr. The s_z spin-resolved Wilson loop spectra in (a,d) exhibit clear gaps at $\gamma_1^\pm = \pi/2 = 0.5\pi$. We have also confirmed that the gaps in the k_3-directed s_z spin-resolved Wilson loop spectra remain present for all values of k_2. We may hence define in (a) the $\mathcal{I}$-invariant (see SRef. [15] and SN 6 E) nested Wilson projectors P^+_{in} and P^+_{out} [SEq. (4.46)], and similarly define in (d) the $\mathcal{I}$-invariant nested Wilson projectors P^-_{in} and P^-_{out} [SEq. (4.46)]. (b,c) Respectively the determinants of the k_2-directed nested P_+-Wilson loop matrix $[\mathcal{W}_2^+(k_1)]$ for the inner and outer s_z spin-resolved P_+-Wilson bands in (a), plotted as functions of the remaining crystal momentum k_1. Following the sign convention established in SEq. (3.24), the nested s_z spin-resolved P_+-Wilson loop winding numbers in (b,c) respectively indicate the nested partial Chern numbers [SEq. (4.64)] $C^+_{\gamma_2,in} = -1$ and $C^+_{\gamma_2,out} = -1$. (e,f) Respectively the determinants of the k_2-directed nested P_--Wilson loop matrix $[\mathcal{W}_2^-(k_1)]$ for the inner and outer s_z spin-resolved P_--Wilson bands in (d), plotted as functions of the remaining crystal momentum k_1. Following the sign convention established in SEq. (3.24), the nested s_z spin-resolved P_--Wilson loop winding numbers in (e,f) respectively indicate the nested partial Chern numbers [SEq. (4.64)] $C^-_{\gamma_2,in} = 1$ and $C^-_{\gamma_2,out} = 1$, which carry opposite signs from those in (b,c) due to the action of $\mathcal{T}$ symmetry on the nested spin-resolved Wilson spectrum (SN 6 H). Because the nested s_z spin-resolved partial Chern numbers in (b,c) are the same [and opposite to those in (e,f)], this implies that for $\hat{\mathbf{n}} = \hat{\mathbf{z}}$, α-BiBr has nonvanishing $\mathbf{G}_3$ partial weak Chern numbers [SEq. (4.71)] $\nu_3^\pm$. This is consistent with our earlier $P_\pm$-Wilson loop calculations demonstrating that for $\hat{\mathbf{n}} = \hat{\mathbf{z}}$, α-BiBr realizes a 3D QSHI state with $\nu_3^\pm = \mp 2$ [see SN 4 D 2 and SEq. (10.20) and the surrounding text]. The calculations detailed in this figure were performed using the freely available Python package NESTED_AND_SPIN_RESOLVED_WILSON_LOOP [97], which represents an extension of the PythTB open-source Python tight-binding package [171] that was implemented and utilized for the preparation of SRefs. [8, 15] and the present work.

number. Our observation that the s_z spin-resolved k_3-directed hybrid Wannier sheets (Wilson bands) in α-BiBr have the same Chern numbers $C^\pm_{\gamma_2,in} = C^\pm_{\gamma_2,out} = \mp 1$ implies that α-BiBr overall has nonvanishing $\mathbf{G}_3$ partial weak Chern numbers $\nu_3^\pm$ [SEq. (4.71)], consistent with our earlier $P_\pm$-Wilson loop calculations demonstrating that for $\hat{\mathbf{n}} = \hat{\mathbf{z}}$, α-BiBr realizes a 3D QSHI state with $\nu_3^\pm = \mp 2$ [see SN 4 D 2 and SEq. (10.20) and the surrounding text].

We next revisit the Ps_xP spectrum of α-BiBr. Because there is a spin gap at all $\mathbf{k}$ points for $\hat{\mathbf{n}} = \hat{\mathbf{x}}$ [SFig. 38(c,f)], then we may compute the spin-resolved $P_\pm$-Wilson loop on the positive and negative spin bands (SN 3 B). In SFig. 43(a) [SFig. 43(d)], we show the k_3-directed P_+- [P_--] Wilson loop spectrum for the positive [negative] s_x spin bands of α-BiBr. As with the ordinary k_3-directed P-Wilson loop [SFig. 41(a)] and the s_z spin-resolved k_3-directed $P_\pm$-Wilson bands [SFig. 42(a,d)], the s_x spin-resolved k_3-directed $P_\pm$-Wilson bands are gapped, and form $\mathcal{I}$-symmetric groupings

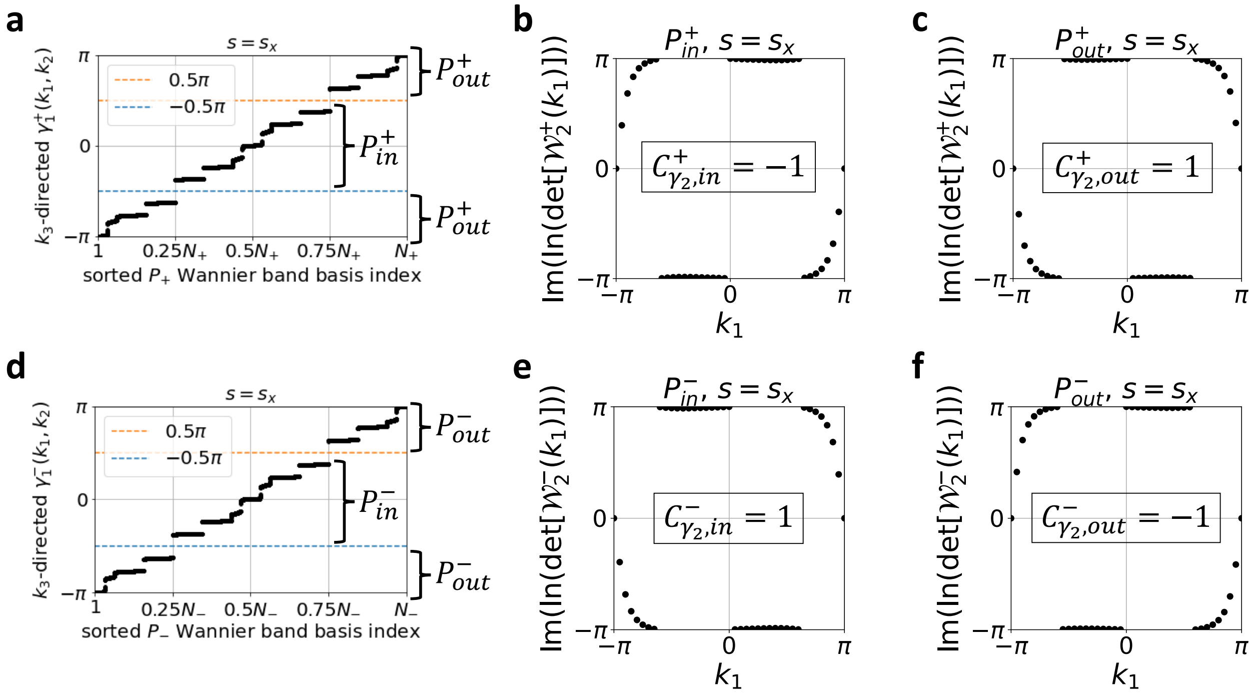

Supplementary Figure 43. $\hat{\mathbf{n}} = \hat{\mathbf{x}}$ nested spin-resolved Wilson loop spectrum of α-BiBr. The calculations shown in this figure were performed using a $41 \times 41 \times 41$ $\mathbf{k}$-grid uniformly spaced over the 3D BZ in the reduced $\mathbf{k}$ coordinates defined in SEq. (10.9). (a,d) Respectively the eigenphases of the k_3-directed [see SEq. (10.9)] s_x spin-resolved P_+- and P_--Wilson loop spectra of α-BiBr. The s_x spin-resolved Wilson loop spectra in (a,d) exhibit clear gaps at $\gamma_1^{\pm} = \pi/2 = 0.5\pi$. We have also confirmed that the gaps in the k_3-directed s_x spin-resolved Wilson loop spectra remain present for all values of k_2. We may hence define in (a) the $\mathcal{I}$-invariant (see SRef. [15] and SN 6 E) nested Wilson projectors P^+_{in} and P^+_{out} [SEq. (4.46)], and similarly define in (d) the $\mathcal{I}$-invariant nested Wilson projectors P^-_{in} and P^-_{out} [SEq. (4.46)]. (b,c) Respectively the determinants of the k_2-directed nested P_+-Wilson loop matrix $[\mathcal{W}_2^+(k_1)]$ for the inner and outer s_x spin-resolved P_+-Wilson bands in (a), plotted as functions of the remaining crystal momentum k_1. Following the sign convention established in SEq. (3.24), the nested s_x spin-resolved P_+-Wilson loop winding numbers in (b,c) respectively indicate the nested partial Chern numbers [SEq. (4.64)] $C^+_{\gamma_2,in} = -1$ and $C^+_{\gamma_2,out} = 1$. (e,f) Respectively the determinants of the k_2-directed nested P_--Wilson loop matrix $[\mathcal{W}_2^-(k_1)]$ for the inner and outer s_x spin-resolved P_--Wilson bands in (d), plotted as functions of the remaining crystal momentum k_1. Following the sign convention established in SEq. (3.24), the nested s_x spin-resolved P_--Wilson loop winding numbers in (e,f) respectively indicate the nested partial Chern numbers [SEq. (4.64)] $C^-_{\gamma_2,in} = 1$ and $C^-_{\gamma_2,out} = -1$, which carry opposite signs from those in (b,c) due to the action of $\mathcal{T}$ symmetry on the nested spin-resolved Wilson spectrum (SN 6 H). Because the nested s_x spin-resolved partial Chern numbers in (b,c) are the opposite [and opposite to those in (e,f)], this implies that for $\hat{\mathbf{n}} = \hat{\mathbf{x}}$, α-BiBr has vanishing $\mathbf{G}_3$ partial weak Chern numbers [SEq. (4.71)] $\nu_3^{\pm} = 0$. Instead, the opposite, odd nested partial Chern numbers in (b,c) [and in (e,f)] are identical to those of an $\mathcal{I}$-protected AXI [15], indicating that for $\hat{\mathbf{n}} = \hat{\mathbf{x}}$, α-BiBr carries nontrivial, origin-independent, $\mathcal{I}$-quantized partial axion angles $\theta^{\pm} = \pi$ (SN 4 D 2). α-BiBr for $\hat{\mathbf{n}} = \hat{\mathbf{x}}$ hence represents a real-material realization of the spin-stable T-DAXI state introduced in this work. The calculations detailed in this figure were performed using the freely available Python package NESTED_AND_SPIN_RESOLVED_WILSON_LOOP [97], which represents an extension of the PythTB open-source Python tight-binding package [171] that was implemented and utilized for the preparation of SRefs. [8, 15] and the present work.

(see SN 6 E) centered around $\gamma_1^{\pm}(k_1,k_2) = 0, \pi$ that are separated by spin-resolved Wilson gaps at $\gamma_1^{\pm}(k_1,k_2) = \pm\pi/2 = \pm 0.5\pi$. We have also numerically confirmed that the k_3-directed $P_{\pm}$-Wilson bands in α-BiBr remain gapped at $\gamma_1^{\pm}(k_1,k_2) = \pm\pi/2$ for all values of k_2, allowing us to consistently define $\mathcal{I}$-symmetric inner $[P^{\pm}_{in}]$ and outer $[P^{\pm}_{out}]$ nested s_x spin-resolved Wilson projectors [SFig. 43(a,d)]. Using $P^{\pm}_{in}$ and $P^{\pm}_{out}$, we then respectively compute the inner and outer k_2-directed nested spin-resolved Wilson loop (SN 4 B) of α-BiBr for both the positive and negative s_x spin bands, whose determinants $\mathrm{Im}\left(\log\left(\det[\mathcal{W}_2^{\pm}(k_1)]\right)\right)$ are plotted in SFig. 43(b,c,e,f) as functions of the remaining crystal momentum k_1. For both the positive and negative spin bands, the inner and outer nested s_x spin-resolved Wilson bands exhibit *opposite* winding numbers [SEq. (4.64)]. Specifically, we respectively find that $C^{\pm}_{\gamma_2,in} = \mp 1$

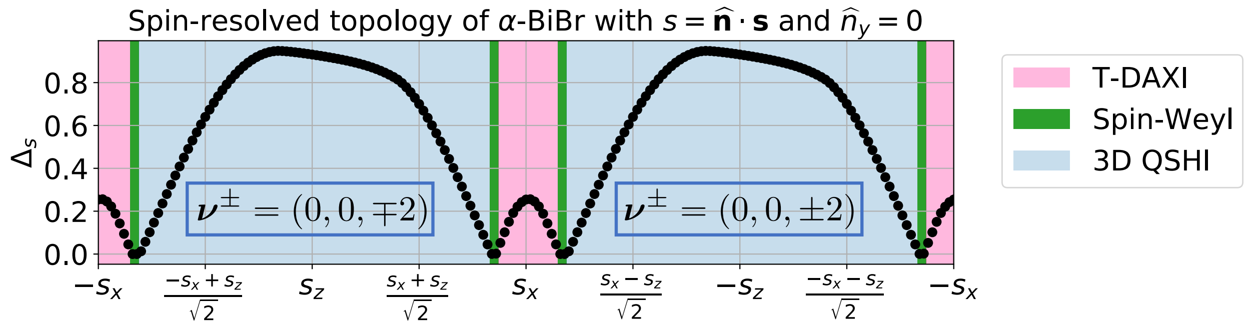

Supplementary Figure 44. High-resolution numerical spin gap $\Delta_{s=\hat{\mathbf{n}}\cdot\mathbf{s}}$ in α-BiBr computed as a function of the spin resolution direction $\hat{\mathbf{n}}$. We take $\hat{\mathbf{n}}$ to lie in the xz-plane and then angular parameterize $\hat{\mathbf{n}}$ using SEq. (10.13) with fixed $\vartheta = 0.5\pi$ and varying $\phi \in [-\pi, \pi]$. Unlike the previous numerical (global) spin gap calculation in SFig. 39, we here take a finer angular resolution $\Delta\phi = 0.01\pi$ in order to determine the spin-resolved topological phase boundaries in α-BiBr. By performing a Nelder-Mead minimization [259] on the direct spin gap function $\Delta_s(\mathbf{k})$ taking as the initial points 100 $\mathbf{k}$ points randomly sampled from the 3D BZ in the reduced $\mathbf{k}$ coordinates defined in SEq. (10.9), we define for each $\hat{\mathbf{n}}$ the numerical spin gap $\Delta_{s=\hat{\mathbf{n}}\cdot\mathbf{s}}$ as the minimal value (in the units of $\hbar/2$) of the 100 minimization results for the fixed value of $\hat{\mathbf{n}}$. We note that the right half of this figure was generated directly from the left half for numerical expediency by relying on the fact that $\Delta_s = \Delta_{-s}$, which follows from the definition of the projected spin operator PsP (see SN 2 B). We identify four narrow regions (green background) that are spin-gapless to within numerical precision. By directly computing the spin spectrum for selected $\hat{\mathbf{n}}$ within the green regions, we confirm that they represent spin-Weyl states that separate four spin-gapped regions in $\hat{\mathbf{n}}$ parameter space (blue and pink regions). By comparing the regions in this plot to the (nested) spin-resolved Wilson loop calculations performed earlier in this section [SFigs. 40, 42, and 43], as well as by performing additional nested spin-resolved Wilson loop calculations for selected additional ϕ, we confirm that the blue (pink) regions represent spin-stable 3D QSHI (T-DAXI) regimes of α-BiBr. Specifically, the blue 3D QSHI region centered around $\hat{\mathbf{n}} = \hat{\mathbf{z}}$ [$\hat{\mathbf{n}} = -\hat{\mathbf{z}}$] is characterized by the partial weak Chern numbers $\boldsymbol{\nu}^{\pm} = (0, 0, \mp 2)$ [$\boldsymbol{\nu}^{\pm} = (0, 0, \pm 2)$], and the two pink T-DAXI regions centered around $\hat{\mathbf{n}} = \pm\hat{\mathbf{x}}$ are characterized by nontrivial, origin-independent, $\mathcal{I}$-quantized partial axion angles $\theta^{\pm} = \pi$ (see SN 4 D). The calculations detailed in this figure were performed using the freely available Python package NESTED_AND_SPIN_RESOLVED_WILSON_LOOP [97], which represents an extension of the PythTB open-source Python tight-binding package [171] that was implemented and utilized for the preparation of SRefs. [8, 15] and the present work.

[$C^{\pm}_{\gamma_2,out} = \pm 1$] for the inner [outer] s_x $P_{\pm}$-Wilson bands in α-BiBr, employing the sign convention established in SEq. (3.24) in which the *negative* winding number of the k_2-directed nested spin-resolved Wilson loop as a function of k_1 gives the nested partial Chern number. Our observation that the s_x spin-resolved k_3-directed hybrid Wannier sheets (Wilson bands) in α-BiBr have opposite Chern numbers $C^{\pm}_{\gamma_2,in} = -C^{\pm}_{\gamma_2,out} = \mp 1$ implies that α-BiBr overall has vanishing $\mathbf{G}_3$ partial weak Chern numbers $\nu_3^{\pm}$ [SEq. (4.71)], consistent with our earlier $P_{\pm}$-Wilson loop calculations demonstrating that $\nu_3^{\pm} = 0$ for $\hat{\mathbf{n}} = \hat{\mathbf{x}}$ in α-BiBr [see SEq. (10.20) and the surrounding text]. Unlike the 3D QSHI state realized for $\hat{\mathbf{n}} = \hat{\mathbf{z}}$ in α-BiBr [SFig. 42(b,c,e,f)], the oppositely signed nested partial Chern numbers of the inner and outer Wilson bands in SFig. 43(b,c,e,f) indicate that for $\hat{\mathbf{n}} = \hat{\mathbf{x}}$, α-BiBr instead has nontrivial, origin-independent, $\mathcal{I}$-quantized partial axion angles $\theta^{\pm} = \pi$ (see SRef. [15] and SN 4 D 2), and hence realizes the spin-stable T-DAXI state introduced in this work.

Spin-Resolved Topology of α-BiBr: Phase Diagram. We have in this section performed extensive spin gap and (nested, partial) Wilson loop calculations to demonstrate that α-BiBr exhibits a bulk spin gap over a large range of $\hat{\mathbf{n}}$, realizes a 3D QSHI state for $\hat{\mathbf{n}} = \hat{\mathbf{z}}$, and realizes a T-DAXI state for $\hat{\mathbf{n}} = \hat{\mathbf{x}}$. As established in SN 2 C, the spin spectrum varies adiabatically under small changes in the system Hamiltonian, which can be treated as equivalent to small changes in the spin resolution direction $\hat{\mathbf{n}}$. This indicates that for small deviations in $\hat{\mathbf{n}}$ from $\hat{\mathbf{n}} = \hat{\mathbf{z}}$ [$\hat{\mathbf{n}} = \hat{\mathbf{x}}$], α-BiBr will still also realize a spin-stable 3D QSHI [T-DAXI] state. However we have not yet determined the exact phase boundaries in $\hat{\mathbf{n}}$ parameter space of the 3D QSHI and T-DAXI spin-stable states in α-BiBr. To create a *spin-resolved phase diagram* for α-BiBr, we begin by taking $\hat{\mathbf{n}}$ to lie in the xz-plane, such that $\hat{\mathbf{n}} = (\hat{n}_x, 0, \hat{n}_z)$. We next again parameterize $\hat{\mathbf{n}}$ using the (ϑ, ϕ) angular parameterization in SEq. (10.13), but here fixing $\vartheta = \pi/2$ while varying ϕ with an angular resolution of $\Delta\phi = 0.01\pi$ [a finer resolution than the full spin (hemi-) sphere Δ_s calculations for α-BiBr in SFig. 39]. For each ϕ, we then perform a Nelder-Mead minimization [259] on the direct spin gap function $\Delta_s(\mathbf{k})$ taking 100 $\mathbf{k}$ points randomly sampled from the 3D BZ as the initial points. Lastly, we define the *numerical*

spin gap $\Delta_{s=\hat{\mathbf{n}}\cdot\mathbf{s}}$ as the minimal value of the 100 minimization results for each spin direction $\hat{\mathbf{n}}$.

In SFig. 44 we plot the numerical (global) spin gap in α-BiBr for $\hat{n}_y = 0$ [$\vartheta = \pi/2$ in SEq. (10.13)]. Over the spin resolution circle parameterized by $\vartheta = \pi/2$ in α-BiBr [SEq. (10.13)], there are only four very narrow regions in which the global spin gap closes within numerical precision [green regions in SFig. 44]. By computing the full spin spectrum of α-BiBr for representative and continuous values of $\hat{\mathbf{n}}$ within the green (spin-gapless) regions in SFig. 44, we have confirmed that the spin-gapless regions correspond to spin-Weyl semimetal states. Combined with the extensive (nested) spin-resolved Wilson loops performed in this section, we hence conclude that for $\vartheta = \pi/2$, α-BiBr hosts four spin-gapped regions: a $\boldsymbol{\nu}^{\pm} = (0, 0, \mp 2)$ 3D QSHI region centered around $\hat{\mathbf{n}} = \hat{\mathbf{z}}$, a $\boldsymbol{\nu}^{\pm} = (0, 0, \pm 2)$ 3D QSHI region centered around $\hat{\mathbf{n}} = -\hat{\mathbf{z}}$, and two T-DAXI regions centered around $\hat{\mathbf{n}} = \pm\hat{\mathbf{x}}$. α-BiBr hence realizes for varying $\hat{\mathbf{n}}$ *all three* of the spin resolutions of a helical HOTI uncovered in this work: 3D QSHI, spin-Weyl semimetal, and T-DAXI states.

C. Physical Signatures of Spin-Gapped States in α-BiBr: Bulk Spin Hall Conductivity

To demonstrate physical signatures of the spin-gapped states in α-BiBr, we will in this section compute the intrinsic bulk contribution to the (non-quantized) spin Hall conductivity. We will specifically compute the non-quantized bulk spin Hall conductivity (per unit cell) for s_z and s_x, and will then compare the results to the *quantized* bulk spin-resolved topology. Previously in SN 7, we used the Kubo formula to derive the spin Hall conductivity tensor $\sigma^{s,i}_{\mu\nu}$, which we emphasize is generically nonquantized due to s-nonconserving SOC. As discussed in SEq. (4.73) and the surrounding text, in spin-gapped states, the quantized partial weak Chern vector $\boldsymbol{\nu}^{\pm} = (\nu_1^{\pm}, \nu_2^{\pm}, \nu_3^{\pm})$ indicates the bulk topological contribution to the intrinsic spin Hall conductivity per layered unit cell. However in real materials like α-BiBr—even those with bulk spin gap—one might expect that SOC destroys any discernible relationship between $\boldsymbol{\nu}^{\pm}$ and $\sigma^{s,i}_{\mu\nu}$. However as we will show below, we find remarkable agreement between $\boldsymbol{\nu}^{\pm}$ and $\sigma^{s,i}_{\mu\nu}$ in α-BiBr for s_z and s_x spins.

To compute the spin Hall conductivity per unit cell of α-BiBr, we use our implementation [SEq. (7.8)] of the Kubo formula and focus on the s_z and s_x spin Hall conductivities. Previously in SN 10 B, we found that α-BiBr is a $\boldsymbol{\nu}^{\pm} = (0, 0, \mp 2)$ 3D QSHI [$\boldsymbol{\nu}^{\pm} = \mathbf{0}$ T-DAXI] for $\hat{\mathbf{n}} = \hat{\mathbf{z}}$ [$\hat{\mathbf{n}} = \hat{\mathbf{x}}$] spin resolution direction. By direct numerical computation, we first find that the s_z spin Hall conductivity (per unit cell) in α-BiBr—crucially allowing for spin-nonconserving SOC—is given by

$$\begin{aligned} \sigma^{s,z}_{12} &= \frac{e}{4\pi}(-3.62), \\ \sigma^{s,z}_{31} &= \frac{e}{4\pi}(-0.0001), \\ \sigma^{s,z}_{23} &= \frac{e}{4\pi}(-0.009). \end{aligned} \tag{10.21}$$

SEq. (10.21) shows remarkable agreement with the partial weak Chern numbers of α-BiBr. Specifically as discussed in SEq. (4.73) and the surrounding text, an s-conserving, $\mathcal{T}$-invariant quantum spin Hall state will carry a bulk spin Hall conductivity of $[e/(4\pi)] \times 2\nu^{+}$ (noting that $\nu^{+} = -\nu^{-}$ due to $\mathcal{T}$ symmetry). This implies that if α-BiBr had perfect s_z spin symmetry, it would carry the quantized spin Hall conductivities (per cell) of $\sigma^{s,z}_{12} = [e/(4\pi)] \times -4$, $\sigma^{s,z}_{31} = \sigma^{s,z}_{23} = 0$ (*i.e.* the spin Hall conductivity would be entirely given by the topological contribution). Hence even though the s_z spin gap in α-BiBr $\Delta_{s_z} \approx 0.9309028798325673$ is only $\approx 46\%$ of the maximum possible value $\Delta_s = 2$ [see SFig. 38(e)], indicating the presence of non-negligible s_z-nonconserving SOC, the bulk intrinsic spin Hall conductivity (per unit cell) in SEq. (10.21) only deviates slightly from the quantized topological contribution that originates from its nontrivial spin-resolved topology.

We next perform the analogous computation of the s_x spin Hall conductivity per unit cell. We find that for α-BiBr

$$\begin{aligned} \sigma^{s,x}_{12} &= \frac{e}{4\pi}(-0.026), \\ \sigma^{s,x}_{31} &= \frac{e}{4\pi}(-0.00002), \\ \sigma^{s,x}_{23} &= \frac{e}{4\pi}(-0.023). \end{aligned} \tag{10.22}$$

Previously in SN 10 B, we found that α-BiBr realizes a $\boldsymbol{\nu}^{\pm} = \mathbf{0}$ T-DAXI for $\hat{\mathbf{n}} = \hat{\mathbf{x}}$. In a T-DAXI state, the bulk topological contribution to the spin Hall conductivity vanishes (though the bulk nontrivial 3D partial axion angles $\theta^{\pm} = \pi$ give rise to an anomalous 2D surface spin Hall conductivity, see SN 4 D 3, 5, and 7 C). Even though the s_x spin gap in α-BiBr is even smaller than its s_z spin gap [$\Delta_{s_x} \approx 0.2550432063802285$, $\approx 12\%$ of the maximum possible

value $\Delta_s = 2$, see SFig. 38(f)], we find that the s_x spin Hall conductivity nevertheless nearly matches the vanishing value expected for an idealized s_x-conserving T-DAXI state.

We have hence demonstrated that for s_z and s_x spins, the nonquantized bulk intrinsic spin Hall conductivity in α-BiBr lies close to the quantized topological contribution from its nontrivial spin-resolved bulk topology. Practically, the calculations performed in this section suggest a highly anisotropic spin Hall response in α-BiBr that interpolates between a large, extensive bulk contribution for s_z spin transport to a small, surface-dominated contribution for s_x spin transport. Given that α-BiBr is readily synthesizable [64–66, 68, 72, 109–113, 268], the anisotropic spin-electromagnetic response predicted in this work should be accessible through straightforward (inverse) spin Hall measurements that are achievable within a short timeframe.

SUPPLEMENTARY REFERENCES

[1] Prodan, E. Robustness of the spin-Chern number. *Phys. Rev. B* **80**, 125327 (2009). URL https://link.aps.org/doi/10.1103/PhysRevB.80.125327.

[2] Fu, L. & Kane, C. L. Time reversal polarization and a Z_2 adiabatic spin pump. *Phys. Rev. B* **74**, 195312 (2006). URL https://link.aps.org/doi/10.1103/PhysRevB.74.195312.

[3] Yu, R., Qi, X. L., Bernevig, A., Fang, Z. & Dai, X. Equivalent expression of $\mathbb{Z}_2$ topological invariant for band insulators using the non-Abelian Berry connection. *Phys. Rev. B* **84**, 075119 (2011). URL https://link.aps.org/doi/10.1103/PhysRevB.84.075119.

[4] Alexandradinata, A., Dai, X. & Bernevig, B. A. Wilson-loop characterization of inversion-symmetric topological insulators. *Phys. Rev. B* **89**, 155114 (2014). URL https://link.aps.org/doi/10.1103/PhysRevB.89.155114.

[5] Taherinejad, M., Garrity, K. F. & Vanderbilt, D. Wannier center sheets in topological insulators. *Phys. Rev. B* **89**, 115102 (2014). URL https://link.aps.org/doi/10.1103/PhysRevB.89.115102.

[6] Kane, C. L. & Mele, E. J. Quantum Spin Hall Effect in Graphene. *Phys. Rev. Lett.* **95**, 226801 (2005). URL https://link.aps.org/doi/10.1103/PhysRevLett.95.226801.

[7] Sheng, D. N., Weng, Z. Y., Sheng, L. & Haldane, F. D. M. Quantum Spin-Hall Effect and Topologically Invariant Chern Numbers. *Phys. Rev. Lett.* **97**, 036808 (2006). URL https://link.aps.org/doi/10.1103/PhysRevLett.97.036808.

[8] Wieder, B. J. *et al.* Strong and fragile topological Dirac semimetals with higher-order Fermi arcs. *Nature Communications* **11**, 627 (2020). URL https://doi.org/10.1038/s41467-020-14443-5.

[9] Schüler, M. *et al.* Local Berry curvature signatures in dichroic angle-resolved photoelectron spectroscopy from two-dimensional materials. *Science Advances* **6**, eaay2730 (2020). URL https://www.science.org/doi/abs/10.1126/sciadv.aay2730.

[10] Ünzelmann, M. *et al.* Momentum-space signatures of Berry flux monopoles in the Weyl semimetal TaAs. *Nature Communications* **12**, 3650 (2021). URL https://doi.org/10.1038/s41467-021-23727-3.

[11] Cho, S. *et al.* Studying local Berry curvature in 2H-WSe_2 by circular dichroism photoemission utilizing crystal mirror plane. *Scientific Reports* **11**, 1684 (2021). URL https://doi.org/10.1038/s41598-020-79672-6.

[12] Di Sante, D. *et al.* Flat band separation and robust spin Berry curvature in bilayer kagome metals. *Nature Physics* **19**, 1135–1142 (2023). URL https://doi.org/10.1038/s41567-023-02053-z.

[13] Benalcazar, W. A., Bernevig, B. A. & Hughes, T. L. Quantized electric multipole insulators. *Science* **357**, 61–66 (2017). URL https://www.science.org/doi/abs/10.1126/science.aah6442.

[14] Benalcazar, W. A., Bernevig, B. A. & Hughes, T. L. Electric multipole moments, topological multipole moment pumping, and chiral hinge states in crystalline insulators. *Phys. Rev. B* **96**, 245115 (2017). URL https://link.aps.org/doi/10.1103/PhysRevB.96.245115.

[15] Wieder, B. J. & Bernevig, B. A. The Axion Insulator as a Pump of Fragile Topology. *arXiv e-prints* arXiv:1810.02373 (2018). URL https://arxiv.org/abs/1810.02373.

[16] Varnava, N., Souza, I. & Vanderbilt, D. Axion coupling in the hybrid Wannier representation. *Phys. Rev. B* **101**, 155130 (2020). URL https://link.aps.org/doi/10.1103/PhysRevB.101.155130.

[17] Po, H. C., Vishwanath, A. & Watanabe, H. Symmetry-based indicators of band topology in the 230 space groups. *Nature Communications* **8**, 50 (2017). URL https://doi.org/10.1038/s41467-017-00133-2.

[18] Schindler, F. *et al.* Higher-order topological insulators. *Science Advances* **4**, eaat0346 (2018). URL https://www.science.org/doi/abs/10.1126/sciadv.aat0346.

[19] Schindler, F. *et al.* Higher-order topology in bismuth. *Nature Physics* **14**, 918–924 (2018). URL https://doi.org/10.1038/s41567-018-0224-7.

[20] Fang, C. & Fu, L. New classes of topological crystalline insulators having surface rotation anomaly. *Science Advances* **5**, eaat2374 (2019). URL https://www.science.org/doi/abs/10.1126/sciadv.aat2374.

[21] Khalaf, E., Po, H. C., Vishwanath, A. & Watanabe, H. Symmetry Indicators and Anomalous Surface States of Topological Crystalline Insulators. *Phys. Rev. X* **8**, 031070 (2018). URL https://link.aps.org/doi/10.1103/PhysRevX.8.031070.

[22] Song, Z., Fang, Z. & Fang, C. $(d-2)$-Dimensional Edge States of Rotation Symmetry Protected Topological States. *Phys. Rev. Lett.* **119**, 246402 (2017). URL https://link.aps.org/doi/10.1103/PhysRevLett.119.246402.

[23] Khalaf, E. Higher-order topological insulators and superconductors protected by inversion symmetry. *Phys. Rev. B* **97**, 205136 (2018). URL https://link.aps.org/doi/10.1103/PhysRevB.97.205136.

[24] Langbehn, J., Peng, Y., Trifunovic, L., von Oppen, F. & Brouwer, P. W. Reflection-Symmetric Second-Order Topological Insulators and Superconductors. *Phys. Rev. Lett.* **119**, 246401 (2017). URL https://link.aps.org/doi/10.1103/PhysRevLett.119.246401.

[25] Trifunovic, L. & Brouwer, P. W. Higher-Order Bulk-Boundary Correspondence for Topological Crystalline Phases. *Phys. Rev. X* **9**, 011012 (2019). URL https://link.aps.org/doi/10.1103/PhysRevX.9.011012.

[26] Tyner, A. C. & Goswami, P. Part I: Staggered index and 3D winding number of Kramers-degenerate bands. *arXiv e-prints* arXiv:2109.06871 (2021). URL https://arxiv.org/abs/2109.06871.

[27] Zhang, F., Kane, C. L. & Mele, E. J. Surface State Magnetization and Chiral Edge States on Topological Insulators. *Phys. Rev. Lett.* **110**, 046404 (2013). URL https://link.aps.org/doi/10.1103/PhysRevLett.110.046404.

[28] Ezawa, M. Magnetic second-order topological insulators and semimetals. *Phys. Rev. B* **97**, 155305 (2018). URL https://link.aps.org/doi/10.1103/PhysRevB.97.155305.

[29] Liu, F. & Wakabayashi, K. Novel Topological Phase with a Zero Berry Curvature. *Phys. Rev. Lett.* **118**, 076803 (2017). URL https://link.aps.org/doi/10.1103/PhysRevLett.118.076803.

[30] Franca, S., van den Brink, J. & Fulga, I. C. An anomalous higher-order topological insulator. *Phys. Rev. B* **98**, 201114 (2018). URL https://link.aps.org/doi/10.1103/PhysRevB.98.201114.

[31] Wang, Z., Wieder, B. J., Li, J., Yan, B. & Bernevig, B. A. Higher-Order Topology, Monopole Nodal Lines, and the Origin of Large Fermi Arcs in Transition Metal Dichalcogenides $X\mathrm{Te}_2$ (X = Mo, W). *Phys. Rev. Lett.* **123**, 186401 (2019). URL https://link.aps.org/doi/10.1103/PhysRevLett.123.186401.

[32] Matsugatani, A. & Watanabe, H. Connecting higher-order topological insulators to lower-dimensional topological insulators. *Phys. Rev. B* **98**, 205129 (2018). URL https://link.aps.org/doi/10.1103/PhysRevB.98.205129.

[33] Song, Z., Zhang, T., Fang, Z. & Fang, C. Quantitative mappings between symmetry and topology in solids. *Nature Communications* **9**, 3530 (2018). URL https://doi.org/10.1038/s41467-018-06010-w.

[34] van Miert, G. & Ortix, C. Higher-order topological insulators protected by inversion and rotoinversion symmetries. *Phys. Rev. B* **98**, 081110 (2018). URL https://link.aps.org/doi/10.1103/PhysRevB.98.081110.

[35] You, Y., Devakul, T., Burnell, F. J. & Neupert, T. Higher-order symmetry-protected topological states for interacting bosons and fermions. *Phys. Rev. B* **98**, 235102 (2018). URL https://link.aps.org/doi/10.1103/PhysRevB.98.235102.

[36] Varnava, N. & Vanderbilt, D. Surfaces of axion insulators. *Phys. Rev. B* **98**, 245117 (2018). URL https://link.aps.org/doi/10.1103/PhysRevB.98.245117.

[37] Ahn, J. & Yang, B.-J. Symmetry Representation Approach to Topological Invariants in $C_{2z}T$-Symmetric Systems. *Phys. Rev. B* **99**, 235125 (2019). URL https://link.aps.org/doi/10.1103/PhysRevB.99.235125.

[38] Olsen, T., Taherinejad, M., Vanderbilt, D. & Souza, I. Surface theorem for the Chern-Simons axion coupling. *Phys. Rev. B* **95**, 075137 (2017). URL https://link.aps.org/doi/10.1103/PhysRevB.95.075137.

[39] Olsen, T., Rauch, T., Vanderbilt, D. & Souza, I. Gapless hinge states from adiabatic pumping of axion coupling. *Phys. Rev. B* **102**, 035166 (2020). URL https://link.aps.org/doi/10.1103/PhysRevB.102.035166.

[40] Ghorashi, S. A. A., Hu, X., Hughes, T. L. & Rossi, E. Second-order Dirac superconductors and magnetic field induced Majorana hinge modes. *Phys. Rev. B* **100**, 020509 (2019). URL https://link.aps.org/doi/10.1103/PhysRevB.100.020509.

[41] Yang, Y.-B., Wang, J.-H., Li, K. & Xu, Y. Higher-order topological phases in crystalline and non-crystalline systems: a review. *arXiv e-prints* arXiv:2309.03688 (2023). URL https://arxiv.org/abs/2309.03688.

[42] Song, Z., Huang, S.-J., Qi, Y., Fang, C. & Hermele, M. Topological states from topological crystals. *Science Advances* **5**, eaax2007 (2019). URL https://www.science.org/doi/abs/10.1126/sciadv.aax2007.

[43] Elcoro, L. *et al.* Magnetic topological quantum chemistry. *Nature Communications* **12**, 5965 (2021). URL https://doi.org/10.1038/s41467-021-26241-8.

[44] Gao, J., Guo, Z., Weng, H. & Wang, Z. Magnetic band representations, Fu-Kane-like symmetry indicators, and magnetic topological materials. *Phys. Rev. B* **106**, 035150 (2022). URL https://link.aps.org/doi/10.1103/PhysRevB.106.035150.

[45] Watanabe, H., Po, H. C. & Vishwanath, A. Structure and topology of band structures in the 1651 magnetic space groups. *Science Advances* **4**, eaat8685 (2018). URL https://www.science.org/doi/abs/10.1126/sciadv.aat8685.

[46] Peng, B., Jiang, Y., Fang, Z., Weng, H. & Fang, C. Topological classification and diagnosis in magnetically ordered electronic materials. *Phys. Rev. B* **105**, 235138 (2022). URL https://link.aps.org/doi/10.1103/PhysRevB.105.235138.

[47] Teo, J. C. Y., Fu, L. & Kane, C. L. Surface States and Topological Invariants in Three-Dimensional Topological Insulators: Application to $\mathrm{Bi}_{1-x}\mathrm{Sb}_x$. *Phys. Rev. B* **78**, 045426 (2008). URL https://link.aps.org/doi/10.1103/PhysRevB.78.045426.

[48] Slager, R.-J., Mesaros, A., Juričić, V. & Zaanen, J. The space group classification of topological band-insulators. *Nature Physics* **9**, 98–102 (2013). URL https://doi.org/10.1038/nphys2513.

[49] Khalaf, E., Benalcazar, W. A., Hughes, T. L. & Queiroz, R. Boundary-obstructed topological phases. *Phys. Rev. Res.* **3**, 013239 (2021). URL https://link.aps.org/doi/10.1103/PhysRevResearch.3.013239.

[50] Tang, F., Po, H. C., Vishwanath, A. & Wan, X. Efficient topological materials discovery using symmetry indicators. *Nature Physics* **15**, 470–476 (2019). URL https://doi.org/10.1038/s41567-019-0418-7.

[51] Nayak, A. K. *et al.* Resolving the topological classification of bismuth with topological defects. *Science Advances* **5**, eaax6996 (2019). URL https://www.science.org/doi/abs/10.1126/sciadv.aax6996.

[52] Hsu, C.-H. *et al.* Purely rotational symmetry-protected topological crystalline insulator α-Bi_4Br_4. *2D Materials* (2019). URL https://iopscience.iop.org/article/10.1088/2053-1583/ab1607.

[53] Yoon, C., Liu, C.-C., Min, H. & Zhang, F. Quasi-One-Dimensional Higher-Order Topological Insulators. *arXiv e-prints* arXiv:2005.14710 (2020). URL https://arxiv.org/abs/2005.14710.

[54] Drozdov, I. K. *et al.* One-dimensional topological edge states of bismuth bilayers. *Nature Physics* **10**, 664–669 (2014). URL https://doi.org/10.1038/nphys3048.

[55] Murani, A. *et al.* Ballistic edge states in Bismuth nanowires revealed by SQUID interferometry. *Nature Communications* **8**, 15941 (2017). URL https://doi.org/10.1038/ncomms15941.

[56] Bernard, A. *et al.* Long-lived Andreev states as evidence for protected hinge modes in a bismuth nanoring Josephson junction. *Nature Physics* **19**, 358–364 (2023). URL https://doi.org/10.1038/s41567-022-01858-8.

[57] Liang, S. *et al.* A gap-protected zero-Hall effect state in the quantum limit of the non-symmorphic metal KHgSb. *Nature Materials* **18**, 443–447 (2019). URL https://doi.org/10.1038/s41563-019-0303-x.

[58] Huang, F.-T. *et al.* Polar and phase domain walls with conducting interfacial states in a Weyl semimetal $MoTe_2$. *Nature Communications* **10**, 4211 (2019). URL https://doi.org/10.1038/s41467-019-11949-5.

[59] Wang, W. *et al.* Evidence for an edge supercurrent in the Weyl superconductor $MoTe_2$. *Science* **368**, 534–537 (2020). URL https://science.sciencemag.org/content/368/6490/534.

[60] Choi, Y.-B. *et al.* Evidence of higher-order topology in multilayer WTe_2 from Josephson coupling through anisotropic hinge states. *Nature Materials* **19**, 974–979 (2020). URL https://doi.org/10.1038/s41563-020-0721-9.

[61] Kononov, A. *et al.* One-Dimensional Edge Transport in Few-Layer WTe_2. *Nano Letters* **20**, 4228–4233 (2020). URL https://doi.org/10.1021/acs.nanolett.0c00658.

[62] Huang, C. *et al.* Edge superconductivity in multilayer WTe_2 Josephson junction. *National Science Review* **7**, 1468–1475 (2020). URL https://doi.org/10.1093/nsr/nwaa114.

[63] Wang, Y., Lee, G.-H. & Ali, M. N. Topology and superconductivity on the edge. *Nature Physics* **17**, 542–546 (2021). URL https://doi.org/10.1038/s41567-021-01190-7.

[64] Noguchi, R. *et al.* Evidence for a higher-order topological insulator in a three-dimensional material built from van der Waals stacking of bismuth-halide chains. *Nature Materials* **20**, 473–479 (2021). URL https://doi.org/10.1038/s41563-020-00871-7.

[65] Mao, P. *et al.* Observation of the Topologically Originated Edge States in large-gap Quasi-One-Dimensional a-Bi_4Br_4. *arXiv e-prints* arXiv:2007.00223 (2020). URL https://arxiv.org/abs/2007.00223.

[66] Mao, P. *et al.* Ultralong carrier lifetime of topological edge states in a-Bi4Br4. *arXiv e-prints* arXiv:2007.00264 (2020). URL https://arxiv.org/abs/2007.00264.

[67] Huang, J. *et al.* Room-Temperature Topological Phase Transition in Quasi-One-Dimensional Material Bi_4I_4. *Phys. Rev. X* **11**, 031042 (2021). URL https://link.aps.org/doi/10.1103/PhysRevX.11.031042.

[68] Shumiya, N. *et al.* Evidence of a room-temperature quantum spin Hall edge state in a higher-order topological insulator. *Nature Materials* **21**, 1111–1115 (2022). URL https://doi.org/10.1038/s41563-022-01304-3.

[69] Zhong, J. *et al.* Towards layer-selective quantum spin hall channels in weak topological insulator $Bi_4Br_2I_2$. *Nature Communications* **14**, 4964 (2023). URL https://doi.org/10.1038/s41467-023-40735-7.

[70] Kang, W., Spathelf, F., Fauqué, B., Fuseya, Y. & Behnia, K. Boundary conductance in macroscopic bismuth crystals. *Nature Communications* **13**, 189 (2022). URL https://doi.org/10.1038/s41467-021-27721-7.

[71] Liu, Y. *et al.* Gate-Tunable Transport in Quasi-One-Dimensional α-Bi_4I_4 Field Effect Transistors. *Nano Letters* **22**, 1151–1158 (2022). URL https://doi.org/10.1021/acs.nanolett.1c04264.

[72] Zhong, J. *et al.* Facet-dependent Electronic Quantum Diffusion in the High-Order Topological Insulator Bi_4Br_4. *Phys. Rev. Appl.* **17**, 064017 (2022). URL https://link.aps.org/doi/10.1103/PhysRevApplied.17.064017.

[73] Lee, J. *et al.* Spinful hinge states in the higher-order topological insulators WTe_2. *Nature Communications* **14**, 1801 (2023). URL https://doi.org/10.1038/s41467-023-37482-0.

[74] Zhang, T. *et al.* Scanning tunneling microscopy observation of the hinge states of bismuth nanocrystals. *Phys. Rev. B* **108**, 085422 (2023). URL https://link.aps.org/doi/10.1103/PhysRevB.108.085422.

[75] Deng, J. *et al.* Twisted nodal wires and three-dimensional quantum spin Hall effect in distorted square-net compounds. *Phys. Rev. B* **105**, 224103 (2022). URL https://link.aps.org/doi/10.1103/PhysRevB.105.224103.

[76] Wang, Z., Alexandradinata, A., Cava, R. J. & Bernevig, B. A. Hourglass fermions. *Nature* **532**, 189–194 (2016). URL https://doi.org/10.1038/nature17410.

[77] Essin, A. M., Moore, J. E. & Vanderbilt, D. Magnetoelectric polarizability and axion electrodynamics in crystalline insulators. *Phys. Rev. Lett.* **102**, 146805 (2009). URL https://link.aps.org/doi/10.1103/PhysRevLett.102.146805.

[78] Vanderbilt, D. *Berry Phases in Electronic Structure Theory: Electric Polarization, Orbital Magnetization and Topological Insulators* (Cambridge University Press, 2018). URL https://books.google.com/books?id=485FtgEACAAJ.

[79] Qi, X.-L., Hughes, T. L. & Zhang, S.-C. Topological field theory of time-reversal invariant insulators. *Phys. Rev. B* **78**, 195424 (2008). URL https://link.aps.org/doi/10.1103/PhysRevB.78.195424.

[80] Wilczek, F. Two applications of axion electrodynamics. *Phys. Rev. Lett.* **58**, 1799–1802 (1987). URL https://link.aps.org/doi/10.1103/PhysRevLett.58.1799.

[81] Hughes, T. L., Prodan, E. & Bernevig, B. A. Inversion-symmetric topological insulators. *Phys. Rev. B* **83**, 245132 (2011). URL https://link.aps.org/doi/10.1103/PhysRevB.83.245132.

[82] Turner, A. M., Zhang, Y., Mong, R. S. K. & Vishwanath, A. Quantized response and topology of magnetic insulators with inversion symmetry. *Phys. Rev. B* **85**, 165120 (2012). URL https://link.aps.org/doi/10.1103/PhysRevB.85.165120.

[83] Xu, Y., Song, Z., Wang, Z., Weng, H. & Dai, X. Higher-Order Topology of the Axion Insulator $EuIn_2As_2$. *Phys. Rev.*

Lett. **122**, 256402 (2019). URL https://link.aps.org/doi/10.1103/PhysRevLett.122.256402.

[84] Coh, S. & Vanderbilt, D. Canonical magnetic insulators with isotropic magnetoelectric coupling. *Phys. Rev. B* **88**, 121106 (2013). URL https://link.aps.org/doi/10.1103/PhysRevB.88.121106.

[85] Alexandradinata, A., Nelson, A. & Soluyanov, A. A. Teleportation of Berry curvature on the surface of a Hopf insulator. *Phys. Rev. B* **103**, 045107 (2021). URL https://link.aps.org/doi/10.1103/PhysRevB.103.045107.

[86] Klett, M. *et al.* Topology and magnetism in the Kondo insulator phase diagram. *Phys. Rev. B* **101**, 161112 (2020). URL https://link.aps.org/doi/10.1103/PhysRevB.101.161112.

[87] Ahn, J., Kim, D., Kim, Y. & Yang, B.-J. Band Topology and Linking Structure of Nodal Line Semimetals with Z_2 Monopole Charges. *Phys. Rev. Lett.* **121**, 106403 (2018). URL https://link.aps.org/doi/10.1103/PhysRevLett.121.106403.

[88] Kim, H., Shiozaki, K. & Murakami, S. Glide-symmetric magnetic topological crystalline insulators with inversion symmetry. *Phys. Rev. B* **100**, 165202 (2019). URL https://link.aps.org/doi/10.1103/PhysRevB.100.165202.

[89] Takahashi, R., Tanaka, Y. & Murakami, S. Bulk-edge and bulk-hinge correspondence in inversion-symmetric insulators. *Phys. Rev. Research* **2**, 013300 (2020). URL https://link.aps.org/doi/10.1103/PhysRevResearch.2.013300.

[90] Okuma, N., Sato, M. & Shiozaki, K. Topological classification under nonmagnetic and magnetic point group symmetry: Application of real-space Atiyah-Hirzebruch spectral sequence to higher-order topology. *Phys. Rev. B* **99**, 085127 (2019). URL https://link.aps.org/doi/10.1103/PhysRevB.99.085127.

[91] Lee, E., Furusaki, A. & Yang, B.-J. Fractional charge bound to a vortex in two-dimensional topological crystalline insulators. *Phys. Rev. B* **101**, 241109 (2020). URL https://link.aps.org/doi/10.1103/PhysRevB.101.241109.

[92] Wieder, B. J., Lin, K.-S. & Bradlyn, B. Axionic band topology in inversion-symmetric Weyl-charge-density waves. *Phys. Rev. Res.* **2**, 042010 (2020). URL https://link.aps.org/doi/10.1103/PhysRevResearch.2.042010.

[93] Mulligan, M. & Burnell, F. J. Topological insulators avoid the parity anomaly. *Phys. Rev. B* **88**, 085104 (2013). URL https://link.aps.org/doi/10.1103/PhysRevB.88.085104.

[94] Yu, J., Wieder, B. J. & Liu, C.-X. Dynamical piezomagnetic effect in time-reversal-invariant Weyl semimetals with axionic charge density waves. *Phys. Rev. B* **104**, 174406 (2021). URL https://link.aps.org/doi/10.1103/PhysRevB.104.174406.

[95] Ahn, J., Xu, S.-Y. & Vishwanath, A. Theory of optical axion electrodynamics and application to the Kerr effect in topological antiferromagnets. *Nature Communications* **13**, 7615 (2022). URL https://doi.org/10.1038/s41467-022-35248-8.

[96] Qiu, J.-X. *et al.* Axion optical induction of antiferromagnetic order. *Nature Materials* (2023). URL https://doi.org/10.1038/s41563-023-01493-5.

[97] Lin, K.-S., Wieder, B. J. & Bradlyn, B. *PythTB-based package for nested Wilson loop and spin-resolved tight-binding calculations* (2023). URL https://github.com/kuansenlin/nested_and_spin_resolved_Wilson_loop. https://github.com/kuansenlin/nested_and_spin_resolved_Wilson_loop.

[98] Bianco, R. & Resta, R. Mapping topological order in coordinate space. *Phys. Rev. B* **84**, 241106 (2011). URL https://link.aps.org/doi/10.1103/PhysRevB.84.241106.

[99] Kitaev, A. Anyons in an exactly solved model and beyond. *Annals of Physics* **321**, 2–111 (2006). URL https://www.sciencedirect.com/science/article/pii/S0003491605002381.

[100] Chen, W. Optical absorption measurement of spin Berry curvature and spin Chern marker. *Journal of Physics: Condensed Matter* **35**, 155601 (2023). URL https://dx.doi.org/10.1088/1361-648X/acba72.

[101] Monaco, D. & Moscolari, M. Středa formula for charge and spin currents. *Reviews in Mathematical Physics* **33**, 2060003 (2021). URL https://doi.org/10.1142/S0129055X2060003X.

[102] Hannukainen, J. D., Martínez, M. F., Bardarson, J. H. & Kvorning, T. K. Local Topological Markers in Odd Spatial Dimensions and Their Application to Amorphous Topological Matter. *Phys. Rev. Lett.* **129**, 277601 (2022). URL https://link.aps.org/doi/10.1103/PhysRevLett.129.277601.

[103] Prodan, E. & Schulz-Baldes, H. *Bulk and Boundary Invariants for Complex Topological Insulators* (Springer, 2016). URL https://link.springer.com/book/10.1007/978-3-319-29351-6.

[104] Gilardoni, I., Becca, F., Marrazzo, A. & Parola, A. Real-space many-body marker for correlated $\mathbb{Z}_2$ topological insulators. *Phys. Rev. B* **106**, L161106 (2022). URL https://link.aps.org/doi/10.1103/PhysRevB.106.L161106.

[105] Pozo, O., Repellin, C. & Grushin, A. G. Quantization in Chiral Higher Order Topological Insulators: Circular Dichroism and Local Chern Marker. *Phys. Rev. Lett.* **123**, 247401 (2019). URL https://link.aps.org/doi/10.1103/PhysRevLett.123.247401.

[106] Witten, E. Fermion path integrals and topological phases. *Rev. Mod. Phys.* **88**, 035001 (2016). URL https://link.aps.org/doi/10.1103/RevModPhys.88.035001.

[107] Wieder, B. J. *et al.* Wallpaper fermions and the nonsymmorphic Dirac insulator. *Science* **361**, 246–251 (2018). URL https://www.science.org/doi/abs/10.1126/science.aan2802.

[108] Monaco, D. & Ulčakar, L. Spin Hall conductivity in insulators with nonconserved spin. *Phys. Rev. B* **102**, 125138 (2020). URL https://link.aps.org/doi/10.1103/PhysRevB.102.125138.

[109] Yang, M. *et al.* Large-Gap Quantum Spin Hall State and Temperature-Induced Lifshitz Transition in Bi_4Br_4. *ACS Nano* **16**, 3036–3044 (2022). URL https://doi.org/10.1021/acsnano.1c10539.

[110] Peng, X. *et al.* Observation of Topological Edge States on α-Bi_4Br_4 Nanowires Grown on $TiSe_2$ Substrates. *The Journal of Physical Chemistry Letters* **12**, 10465–10471 (2021). URL https://doi.org/10.1021/acs.jpclett.1c02586.

[111] Qiao, L. *et al.* Ultralong Single-Crystal α-Bi_4Br_4 Nanobelts with a High Current-Carrying Capacity by Mechanical Exfoliation. *The Journal of Physical Chemistry C* **125**, 22312–22317 (2021). URL https://doi.org/10.1021/acs.jpcc.1c06702.

[112] Chen, D.-Y. *et al.* Quantum transport evidence of boundary states and Lifshitz transition in Bi_4Br_4. *Phys. Rev. B* **106**, 075206 (2022). URL https://link.aps.org/doi/10.1103/PhysRevB.106.075206.

[113] Han, J. *et al.* Optical bulk-boundary dichotomy in a quantum spin Hall insulator. *Science Bulletin* **68**, 417–423 (2023). URL https://www.sciencedirect.com/science/article/pii/S2095927323000749.

[114] Prodan, E. Three-dimensional phase diagram of disordered HgTe/CdTe quantum spin-Hall wells. *Phys. Rev. B* **83**, 195119 (2011). URL https://link.aps.org/doi/10.1103/PhysRevB.83.195119.

[115] Stone, M. & Goldbart, P. *Mathematics for Physics: A Guided Tour for Graduate Students* (Cambridge University Press, 2009). URL https://books.google.com/books?id=Z6p71mL5Z9AC.

[116] Zhang, X., Liu, Q., Luo, J.-W., Freeman, A. J. & Zunger, A. Hidden spin polarization in inversion-symmetric bulk crystals. *Nature Physics* **10**, 387–393 (2014). URL https://doi.org/10.1038/nphys2933.

[117] Yuan, L.-D., Zhang, X., Mera, C. & Zunger, A. Uncovering hidden spin polarization of energy bands in antiferromagnets. *arXiv e-prints* arXiv:2211.09921 (2022). URL https://arxiv.org/abs/2211.09921.

[118] Wang, C., Xu, Y. & Duan, W. Ising Superconductivity and Its Hidden Variants. *Accounts of Materials Research* **2**, 526–533 (2021). URL https://doi.org/10.1021/accountsmr.1c00068.

[119] Semenoff, G. W. Condensed-matter simulation of a three-dimensional anomaly. *Phys. Rev. Lett.* **53**, 2449–2452 (1984). URL https://link.aps.org/doi/10.1103/PhysRevLett.53.2449.

[120] Niemi, A. J. & Semenoff, G. W. Fermion number fractionization in quantum field theory. *Physics Reports* **135**, 99–193 (1986). URL https://www.sciencedirect.com/science/article/pii/0370157386901675.

[121] Bistritzer, R. & MacDonald, A. H. Moiré butterflies in twisted bilayer graphene. *Phys. Rev. B* **84**, 035440 (2011). URL https://link.aps.org/doi/10.1103/PhysRevB.84.035440.

[122] Wallace, P. R. The Band Theory of Graphite. *Phys. Rev.* **71**, 622–634 (1947). URL https://link.aps.org/doi/10.1103/PhysRev.71.622.

[123] Bradlyn, B. *et al.* Topological quantum chemistry. *Nature* **547**, 298–305 (2017). URL https://doi.org/10.1038/nature23268.

[124] Călugăru, D. *et al.* General construction and topological classification of crystalline flat bands. *Nature Physics* **18**, 185–189 (2022). URL https://doi.org/10.1038/s41567-021-01445-3.

[125] Regnault, N. *et al.* Catalogue of flat-band stoichiometric materials. *Nature* **603**, 824–828 (2022). URL https://doi.org/10.1038/s41586-022-04519-1.

[126] Chiu, C. S., Ma, D.-S., Song, Z.-D., Bernevig, B. A. & Houck, A. A. Fragile topology in line-graph lattices with two, three, or four gapped flat bands. *Phys. Rev. Res.* **2**, 043414 (2020). URL https://link.aps.org/doi/10.1103/PhysRevResearch.2.043414.

[127] Ogata, T., Kawamura, M. & Ozaki, T. Methods for constructing parameter-dependent flat-band lattices. *Phys. Rev. B* **103**, 205119 (2021). URL https://link.aps.org/doi/10.1103/PhysRevB.103.205119.

[128] Rhim, J.-W. & Yang, B.-J. Classification of flat bands according to the band-crossing singularity of bloch wave functions. *Phys. Rev. B* **99**, 045107 (2019). URL https://link.aps.org/doi/10.1103/PhysRevB.99.045107.

[129] Leykam, D., Andreanov, A. & Flach, S. Artificial flat band systems: from lattice models to experiments. *Advances in Physics: X* **3**, 1473052 (2018). URL https://doi.org/10.1080/23746149.2018.1473052.

[130] Addison, Z. & Trivedi, N. Flat bands arising from spin-orbit assisted orbital frustration. *Phys. Rev. B* **106**, 235144 (2022). URL https://link.aps.org/doi/10.1103/PhysRevB.106.235144.

[131] Fukui, T. & Hatsugai, Y. Entanglement Chern Number for an Extensive Partition of a Topological Ground State. *Journal of the Physical Society of Japan* **83**, 113705 (2014). URL https://doi.org/10.7566/JPSJ.83.113705.

[132] Araki, H., Kariyado, T., Fukui, T. & Hatsugai, Y. Entanglement Chern Number of the Kane–Mele Model with Ferromagnetism. *Journal of the Physical Society of Japan* **85**, 043706 (2016). URL https://doi.org/10.7566/JPSJ.85.043706.

[133] Araki, H., Fukui, T. & Hatsugai, Y. Entanglement Chern number for three-dimensional topological insulators: Characterization by Weyl points of entanglement Hamiltonians. *Phys. Rev. B* **96**, 165139 (2017). URL https://link.aps.org/doi/10.1103/PhysRevB.96.165139.

[134] Schrieffer, J. R. & Wolff, P. A. Relation between the Anderson and Kondo Hamiltonians. *Phys. Rev.* **149**, 491–492 (1966). URL https://link.aps.org/doi/10.1103/PhysRev.149.491.

[135] Winkler, R. *Spin-Orbit Coupling Effects in Two-Dimensional Electron and Hole Systems.* Springer Tracts in Modern Physics (Springer Berlin Heidelberg, 2003). URL https://books.google.com/books?id=w4B8CwAAQBAJ.

[136] Bravyi, S., DiVincenzo, D. P. & Loss, D. Schrieffer–Wolff transformation for quantum many-body systems. *Annals of Physics* **326**, 2793–2826 (2011). URL https://www.sciencedirect.com/science/article/pii/S0003491611001059.

[137] Zhao, W. *et al.* Determination of the Spin Axis in Quantum Spin Hall Insulator Candidate Monolayer WTe_2. *Phys. Rev. X* **11**, 041034 (2021). URL https://link.aps.org/doi/10.1103/PhysRevX.11.041034.

[138] Garcia, J. H. *et al.* Canted Persistent Spin Texture and Quantum Spin Hall Effect in WTe_2. *Phys. Rev. Lett.* **125**, 256603 (2020). URL https://link.aps.org/doi/10.1103/PhysRevLett.125.256603.

[139] Kurebayashi, H., Garcia, J. H., Khan, S., Sinova, J. & Roche, S. Magnetism, symmetry and spin transport in van der Waals layered systems. *Nature Reviews Physics* **4**, 150–166 (2022). URL https://doi.org/10.1038/s42254-021-00403-5.

[140] Bernevig, B. A., Hughes, T. L. & Zhang, S.-C. Quantum Spin Hall Effect and Topological Phase Transition in HgTe Quantum Wells. *Science* **314**, 1757–1761 (2006). URL https://www.science.org/doi/abs/10.1126/science.1133734.

[141] Fu, L., Kane, C. L. & Mele, E. J. Topological Insulators in Three Dimensions. *Phys. Rev. Lett.* **98**, 106803 (2007). URL https://link.aps.org/doi/10.1103/PhysRevLett.98.106803.

[142] Fu, L. & Kane, C. L. Topological insulators with inversion symmetry. *Phys. Rev. B* **76**, 045302 (2007). URL https://link.aps.org/doi/10.1103/PhysRevB.76.045302.

[143] Xu, Y. *et al.* High-throughput calculations of magnetic topological materials. *Nature* **586**, 702–707 (2020). URL https://doi.org/10.1038/s41586-020-2837-0.

[144] Mostofi, A. A. *et al.* wannier90: A tool for obtaining maximally-localised Wannier functions. *Computer Physics Communications* **178**, 685–699 (2008). URL https://www.sciencedirect.com/science/article/pii/S0010465507004936.

[145] Fidkowski, L., Jackson, T. S. & Klich, I. Model Characterization of Gapless Edge Modes of Topological Insulators Using Intermediate Brillouin-Zone Functions. *Phys. Rev. Lett.* **107**, 036601 (2011). URL https://link.aps.org/doi/10.1103/PhysRevLett.107.036601.

[146] Bradlyn, B. & Iraola, M. Lecture Notes on Berry Phases and Topology. *SciPost Phys. Lect. Notes* 51 (2022). URL https://scipost.org/10.21468/SciPostPhysLectNotes.51.

[147] Soluyanov, A. A. & Vanderbilt, D. Wannier Representation of $\mathbb{Z}_2$ Topological Insulators. *Phys. Rev. B* **83**, 035108 (2011). URL https://link.aps.org/doi/10.1103/PhysRevB.83.035108.

[148] Gresch, D. *et al.* Z2Pack: Numerical implementation of hybrid Wannier centers for identifying topological materials. *Phys. Rev. B* **95**, 075146 (2017). URL https://link.aps.org/doi/10.1103/PhysRevB.95.075146.

[149] Cano, J. & Bradlyn, B. Band Representations and Topological Quantum Chemistry. *Annual Review of Condensed Matter Physics* **12**, 225–246 (2021). URL https://doi.org/10.1146/annurev-conmatphys-041720-124134.

[150] Po, H. C., Watanabe, H. & Vishwanath, A. Fragile Topology and Wannier Obstructions. *Phys. Rev. Lett.* **121**, 126402 (2018). URL https://link.aps.org/doi/10.1103/PhysRevLett.121.126402.

[151] Bouhon, A., Black-Schaffer, A. M. & Slager, R.-J. Wilson loop approach to fragile topology of split elementary band representations and topological crystalline insulators with time-reversal symmetry. *Phys. Rev. B* **100**, 195135 (2019). URL https://link.aps.org/doi/10.1103/PhysRevB.100.195135.

[152] Cano, J. *et al.* Topology of Disconnected Elementary Band Representations. *Phys. Rev. Lett.* **120**, 266401 (2018). URL https://link.aps.org/doi/10.1103/PhysRevLett.120.266401.

[153] Bradlyn, B., Wang, Z., Cano, J. & Bernevig, B. A. Disconnected elementary band representations, fragile topology, and Wilson loops as topological indices: An example on the triangular lattice. *Phys. Rev. B* **99**, 045140 (2019). URL https://link.aps.org/doi/10.1103/PhysRevB.99.045140.

[154] Avron, J. E. Adiabatic quantum transport. In *Quantum Symmetries/Symetries Quantiques: Proceedings of the Les Houches Summer School, Session Lxiv, Les Houches, France, 1 August - 8 September, 1995 (Les Houches Summer School Proceedings)* (Elsevier, Amsterdam, 1998). URL http://phsites.technion.ac.il/avron/wp-content/uploads/sites/3/2013/08/leshouches.pdf.

[155] König, M. *et al.* The Quantum Spin Hall Effect: Theory and Experiment. *Journal of the Physical Society of Japan* **77**, 031007 (2008). URL https://doi.org/10.1143/JPSJ.77.031007.

[156] Murakami, S., Nagaosa, N. & Zhang, S.-C. Spin-Hall Insulator. *Phys. Rev. Lett.* **93**, 156804 (2004). URL https://link.aps.org/doi/10.1103/PhysRevLett.93.156804.

[157] Sinova, J. *et al.* Universal Intrinsic Spin Hall Effect. *Phys. Rev. Lett.* **92**, 126603 (2004). URL https://link.aps.org/doi/10.1103/PhysRevLett.92.126603.

[158] Sinova, J., Valenzuela, S. O., Wunderlich, J., Back, C. H. & Jungwirth, T. Spin Hall effects. *Rev. Mod. Phys.* **87**, 1213–1260 (2015). URL https://link.aps.org/doi/10.1103/RevModPhys.87.1213.

[159] Fukui, T. & Hatsugai, Y. Topological aspects of the quantum spin-Hall effect in graphene: Z_2 topological order and spin Chern number. *Phys. Rev. B* **75**, 121403 (2007). URL https://link.aps.org/doi/10.1103/PhysRevB.75.121403.

[160] Kane, C. L. & Mele, E. J. Z_2 Topological Order and the Quantum Spin Hall Effect. *Phys. Rev. Lett.* **95**, 146802 (2005). URL https://link.aps.org/doi/10.1103/PhysRevLett.95.146802.

[161] Fang, C., Gilbert, M. J., Dai, X. & Bernevig, B. A. Multi-Weyl Topological Semimetals Stabilized by Point Group Symmetry. *Phys. Rev. Lett.* **108**, 266802 (2012). URL https://link.aps.org/doi/10.1103/PhysRevLett.108.266802.

[162] Alexandradinata, A., Höller, J., Wang, C., Cheng, H. & Lu, L. Crystallographic splitting theorem for band representations and fragile topological photonic crystals. *Phys. Rev. B* **102**, 115117 (2020). URL https://link.aps.org/doi/10.1103/PhysRevB.102.115117.

[163] Soluyanov, A. A. & Vanderbilt, D. Smooth gauge for topological insulators. *Phys. Rev. B* **85**, 115415 (2012). URL https://link.aps.org/doi/10.1103/PhysRevB.85.115415.

[164] Thouless, D. J., Kohmoto, M., Nightingale, M. P. & den Nijs, M. Quantized Hall Conductance in a Two-Dimensional Periodic Potential. *Phys. Rev. Lett.* **49**, 405–408 (1982). URL https://link.aps.org/doi/10.1103/PhysRevLett.49.405.

[165] Shi, J., Zhang, P., Xiao, D. & Niu, Q. Proper Definition of Spin Current in Spin-Orbit Coupled Systems. *Phys. Rev. Lett.* **96**, 076604 (2006). URL https://link.aps.org/doi/10.1103/PhysRevLett.96.076604.

[166] Tokatly, I. V. Equilibrium Spin Currents: Non-Abelian Gauge Invariance and Color Diamagnetism in Condensed Matter. *Phys. Rev. Lett.* **101**, 106601 (2008). URL https://link.aps.org/doi/10.1103/PhysRevLett.101.106601.

[167] Gorini, C., Raimondi, R. & Schwab, P. Onsager Relations in a Two-Dimensional Electron Gas with Spin-Orbit Coupling. *Phys. Rev. Lett.* **109**, 246604 (2012). URL https://link.aps.org/doi/10.1103/PhysRevLett.109.246604.

[168] Yang, M.-F. & Chang, M.-C. Středa-like formula in the spin Hall effect. *Phys. Rev. B* **73**, 073304 (2006). URL https://link.aps.org/doi/10.1103/PhysRevB.73.073304.

[169] Roy, R. Characterization of three-dimensional topological insulators by two-dimensional invariants. *New Journal of Physics* **12**, 065009 (2010). URL https://dx.doi.org/10.1088/1367-2630/12/6/065009.

[170] Herring, C. Accidental Degeneracy in the Energy Bands of Crystals. *Phys. Rev.* **52**, 365–373 (1937). URL https://link.aps.org/doi/10.1103/PhysRev.52.365.

[171] *Python Tight-Binding (PythTB) Package* (2022). URL https://www.physics.rutgers.edu/pythtb/.

[172] Bradlyn, B. & Iraola, M. Lecture notes on Berry phases and topology. *SciPost Phys. Lect. Notes* 51 (2022). URL https://scipost.org/10.21468/SciPostPhysLectNotes.51.

[173] Armitage, N. P., Mele, E. J. & Vishwanath, A. Weyl and Dirac semimetals in three-dimensional solids. *Rev. Mod. Phys.* **90**, 015001 (2018). URL https://link.aps.org/doi/10.1103/RevModPhys.90.015001.

[174] Schindler, F., Tsirkin, S. S., Neupert, T., Andrei Bernevig, B. & Wieder, B. J. Topological zero-dimensional defect and flux states in three-dimensional insulators. *Nature Communications* **13**, 5791 (2022). URL https://doi.org/10.1038/s41467-022-33471-x.

[175] Ahn, J., Park, S. & Yang, B.-J. Failure of Nielsen-Ninomiya Theorem and Fragile Topology in Two-Dimensional Systems with Space-Time Inversion Symmetry: Application to Twisted Bilayer Graphene at Magic Angle. *Phys. Rev. X* **9**, 021013 (2019). URL https://link.aps.org/doi/10.1103/PhysRevX.9.021013.

[176] Hwang, Y., Ahn, J. & Yang, B.-J. Fragile topology protected by inversion symmetry: Diagnosis, bulk-boundary correspondence, and Wilson loop. *Phys. Rev. B* **100**, 205126 (2019). URL https://link.aps.org/doi/10.1103/PhysRevB.100.205126.

[177] Lee, E., Kim, R., Ahn, J. & Yang, B.-J. Two-dimensional higher-order topology in monolayer graphdiyne. *npj Quantum Materials* **5**, 1 (2020). URL https://doi.org/10.1038/s41535-019-0206-8.

[178] Wieder, B. J. & Kane, C. L. Spin-orbit semimetals in the layer groups. *Phys. Rev. B* **94**, 155108 (2016). URL https://link.aps.org/doi/10.1103/PhysRevB.94.155108.

[179] Litvin, D. B. *Magnetic Group Tables* (International Union of Crystallography, 2013). URL https://www.iucr.org/publ/978-0-9553602-2-0.

[180] Kopsky, V. & Litvin, D. *International Tables for Crystallography, Volume E: Subperiodic Groups*. International Tables for Crystallography (Springer Netherlands, 2002). URL https://onlinelibrary.wiley.com/iucr/itc/E/.

[181] Young, S. M. & Wieder, B. J. Filling-Enforced Magnetic Dirac Semimetals in Two Dimensions. *Phys. Rev. Lett.* **118**, 186401 (2017). URL https://link.aps.org/doi/10.1103/PhysRevLett.118.186401.

[182] Song, Z. *et al.* All Magic Angles in Twisted Bilayer Graphene are Topological. *Phys. Rev. Lett.* **123**, 036401 (2019). URL https://link.aps.org/doi/10.1103/PhysRevLett.123.036401.

[183] Song, Z.-D., Lian, B., Regnault, N. & Bernevig, B. A. Twisted bilayer graphene. II. Stable symmetry anomaly. *Phys. Rev. B* **103**, 205412 (2021). URL https://link.aps.org/doi/10.1103/PhysRevB.103.205412.

[184] Qi, X.-L. & Zhang, S.-C. Spin-Charge Separation in the Quantum Spin Hall State. *Phys. Rev. Lett.* **101**, 086802 (2008). URL https://link.aps.org/doi/10.1103/PhysRevLett.101.086802.

[185] Ran, Y., Vishwanath, A. & Lee, D.-H. Spin-Charge Separated Solitons in a Topological Band Insulator. *Phys. Rev. Lett.* **101**, 086801 (2008). URL https://link.aps.org/doi/10.1103/PhysRevLett.101.086801.

[186] Ringel, Z. & Stern, A. $\mathbb{Z}_2$ anomaly and boundaries of topological insulators. *Phys. Rev. B* **88**, 115307 (2013). URL https://link.aps.org/doi/10.1103/PhysRevB.88.115307.

[187] Rosenberg, G., Guo, H.-M. & Franz, M. Wormhole effect in a strong topological insulator. *Phys. Rev. B* **82**, 041104 (2010). URL https://link.aps.org/doi/10.1103/PhysRevB.82.041104.

[188] Gong, M. *et al.* Transport study of the wormhole effect in three-dimensional topological insulators. *Phys. Rev. B* **102**, 165425 (2020). URL https://link.aps.org/doi/10.1103/PhysRevB.102.165425.

[189] Ostrovsky, P. M., Gornyi, I. V. & Mirlin, A. D. Interaction-Induced Criticality in $\mathbb{Z}_2$ Topological Insulators. *Phys. Rev. Lett.* **105**, 036803 (2010). URL https://link.aps.org/doi/10.1103/PhysRevLett.105.036803.

[190] Assaad, F. F., Bercx, M. & Hohenadler, M. Topological Invariant and Quantum Spin Models from Magnetic π Fluxes in Correlated Topological Insulators. *Phys. Rev. X* **3**, 011015 (2013). URL https://link.aps.org/doi/10.1103/PhysRevX.3.011015.

[191] Juričić, V., Mesaros, A., Slager, R.-J. & Zaanen, J. Universal Probes of Two-Dimensional Topological Insulators: Dislocation and π Flux. *Phys. Rev. Lett.* **108**, 106403 (2012). URL https://link.aps.org/doi/10.1103/PhysRevLett.108.106403.

[192] Tyner, A. C., Sur, S., Puggioni, D., Rondinelli, J. M. & Goswami, P. Topology of three-dimensional Dirac semimetals and generalized quantum spin Hall systems without gapless edge modes. *arXiv e-prints* arXiv:2012.12906 (2020). URL https://arxiv.org/abs/2012.12906.

[193] Tyner, A. C. & Goswami, P. Part II: Witten effect and $\mathbb{Z}$-classification of axion angle $\theta = n\pi$. *arXiv e-prints* arXiv:2206.10636 (2022). URL https://arxiv.org/abs/2206.10636.

[194] Tyner, A. C. & Goswami, P. Solitons and real-space screening of bulk topology of quantum materials. *arXiv e-prints* arXiv:2304.05424 (2023). URL https://arxiv.org/abs/2304.05424.

[195] Landau, L. D. Zur Theorie der Energieubertragung II. *Zeitschrift fur Physik (Sowjetunion)* **2**, 46–51 (1932). URL https://www.sciencedirect.com/science/article/pii/B9780080105864500146.

[196] Zener, C. Non-Adiabatic Crossing of Energy Levels. *Proceedings of the Royal Society of London. Series A, Containing Papers of a Mathematical and Physical Character* **137**, 696–702 (1932). URL http://www.jstor.org/stable/96038.

[197] Rubbmark, J. R., Kash, M. M., Littman, M. G. & Kleppner, D. Dynamical effects at avoided level crossings: A study of the Landau-Zener effect using Rydberg atoms. *Phys. Rev. A* **23**, 3107–3117 (1981). URL https://link.aps.org/doi/10.1103/PhysRevA.23.3107.

[198] Qi, X.-L., Wu, Y.-S. & Zhang, S.-C. Topological quantization of the spin Hall effect in two-dimensional paramagnetic semiconductors. *Phys. Rev. B* **74**, 085308 (2006). URL https://link.aps.org/doi/10.1103/PhysRevB.74.085308.

[199] Laflorencie, N. & Rachel, S. Spin-resolved entanglement spectroscopy of critical spin chains and Luttinger liquids. *Journal of Statistical Mechanics: Theory and Experiment* **2014**, P11013 (2014). URL https://dx.doi.org/10.1088/1742-5468/2014/11/P11013.

[200] Goldstein, M. & Sela, E. Symmetry-Resolved Entanglement in Many-Body Systems. *Phys. Rev. Lett.* **120**, 200602 (2018). URL https://link.aps.org/doi/10.1103/PhysRevLett.120.200602.

[201] Azses, D. & Sela, E. Symmetry-resolved entanglement in symmetry-protected topological phases. *Phys. Rev. B* **102**, 235157 (2020). URL https://link.aps.org/doi/10.1103/PhysRevB.102.235157.

[202] Zeng, H., Duan, W. & Huang, H. Generalization of the nested Wilson loop formalism in topological Dirac semimetals with higher-order Fermi arcs. *Phys. Rev. Res.* **5**, L042003 (2023). URL https://link.aps.org/doi/10.1103/PhysRevResearch.5.L042003.

[203] Horn, R. & Johnson, C. *Matrix Analysis* (Cambridge University Press, 2012). URL https://www.cambridge.org/core/books/matrix-analysis/9CF2CB491C9E97948B15FAD835EF9A8B.

[204] Ran, Y., Zhang, Y. & Vishwanath, A. One-dimensional topologically protected modes in topological insulators with lattice dislocations. *Nature Physics* **5**, 298–303 (2009). URL https://doi.org/10.1038/nphys1220.

[205] Teo, J. C. Y. & Kane, C. L. Topological defects and gapless modes in insulators and superconductors. *Phys. Rev. B* **82**, 115120 (2010). URL https://link.aps.org/doi/10.1103/PhysRevB.82.115120.

[206] Halperin, B. I. Possible States for a Three-Dimensional Electron Gas in a Strong Magnetic Field. *Japanese Journal of Applied Physics* **26**, 1913 (1987). URL https://doi.org/10.7567%2Fjjaps.26s3.1913.

[207] Kohmoto, M., Halperin, B. I. & Wu, Y.-S. Diophantine equation for the three-dimensional quantum Hall effect. *Phys. Rev. B* **45**, 13488–13493 (1992). URL https://link.aps.org/doi/10.1103/PhysRevB.45.13488.

[208] Haldane, F. D. M. Berry Curvature on the Fermi Surface: Anomalous Hall Effect as a Topological Fermi-Liquid Property. *Phys. Rev. Lett.* **93**, 206602 (2004). URL https://link.aps.org/doi/10.1103/PhysRevLett.93.206602.

[209] Huang, S.-J., Song, H., Huang, Y.-P. & Hermele, M. Building crystalline topological phases from lower-dimensional states. *Phys. Rev. B* **96**, 205106 (2017). URL https://link.aps.org/doi/10.1103/PhysRevB.96.205106.

[210] Song, Z., Fang, C. & Qi, Y. Real-space recipes for general topological crystalline states. *Nature Communications* **11**, 4197 (2020). URL https://doi.org/10.1038/s41467-020-17685-5.

[211] Brinkman, W. F. & Elliott, R. J. Theory of spin-space groups. *Proceedings of the Royal Society of London. Series A. Mathematical and Physical Sciences* **294**, 343–358 (1966). URL https://royalsocietypublishing.org/doi/abs/10.1098/rspa.1966.0211.

[212] Yang, J., Fang, C. & Liu, Z.-X. Symmetry-protected nodal points and nodal lines in magnetic materials. *Phys. Rev. B* **103**, 245141 (2021). URL https://link.aps.org/doi/10.1103/PhysRevB.103.245141.

[213] Corticelli, A., Moessner, R. & McClarty, P. A. Spin-space groups and magnon band topology. *Phys. Rev. B* **105**, 064430 (2022). URL https://link.aps.org/doi/10.1103/PhysRevB.105.064430.

[214] Liu, P., Li, J., Han, J., Wan, X. & Liu, Q. Spin-Group Symmetry in Magnetic Materials with Negligible Spin-Orbit Coupling. *Phys. Rev. X* **12**, 021016 (2022). URL https://link.aps.org/doi/10.1103/PhysRevX.12.021016.

[215] Xiao, Z., Zhao, J., Li, Y., Shindou, R. & Song, Z.-D. Spin Space Groups: Full Classification and Applications. *arXiv e-prints* arXiv:2307.10364 (2023). URL https://arxiv.org/abs/2307.10364.

[216] Jiang, Y. *et al.* Enumeration of spin-space groups: Towards a complete description of symmetries of magnetic orders. *arXiv e-prints* arXiv:2307.10371 (2023). URL https://arxiv.org/abs/2307.10371.

[217] Ren, J. *et al.* Enumeration and representation of spin space groups. *arXiv e-prints* arXiv:2307.10369 (2023). URL https://arxiv.org/abs/2307.10369.

[218] Chen, X., Ren, J., Li, J., Liu, Y. & Liu, Q. Spin Space Group Theory and Unconventional Magnons in Collinear Magnets. *arXiv e-prints* arXiv:2307.12366 (2023). URL https://arxiv.org/abs/2307.12366.

[219] Yang, J., Liu, Z.-X. & Fang, C. Symmetry invariants in magnetically ordered systems having weak spin-orbit coupling. *arXiv e-prints* arXiv:2105.12738 (2021). URL https://arxiv.org/abs/2105.12738.

[220] Armitage, N. P. & Wu, L. On the matter of topological insulators as magnetoelectrics. *SciPost Phys.* **6**, 046 (2019). URL https://scipost.org/10.21468/SciPostPhys.6.4.046.

[221] Burkov, A. A. Negative longitudinal magnetoresistance in Dirac and Weyl metals. *Phys. Rev. B* **91**, 245157 (2015). URL https://link.aps.org/doi/10.1103/PhysRevB.91.245157.

[222] Wang, Z. & Zhang, S.-C. Chiral anomaly, charge density waves, and axion strings from Weyl semimetals. *Phys. Rev. B* **87**, 161107 (2013). URL https://link.aps.org/doi/10.1103/PhysRevB.87.161107.

[223] You, Y., Cho, G. Y. & Hughes, T. L. Response properties of axion insulators and Weyl semimetals driven by screw dislocations and dynamical axion strings. *Phys. Rev. B* **94**, 085102 (2016). URL https://link.aps.org/doi/10.1103/PhysRevB.94.085102.

[224] Cano, J. *et al.* Building blocks of topological quantum chemistry: Elementary band representations. *Phys. Rev. B* **97**, 035139 (2018). URL https://link.aps.org/doi/10.1103/PhysRevB.97.035139.

[225] Caio, M. D., Möller, G., Cooper, N. R. & Bhaseen, M. J. Topological marker currents in Chern insulators. *Nature Physics* **15**, 257–261 (2019). URL https://doi.org/10.1038/s41567-018-0390-7.

[226] Tran, D.-T., Dauphin, A., Goldman, N. & Gaspard, P. Topological Hofstadter insulators in a two-dimensional quasicrystal. *Phys. Rev. B* **91**, 085125 (2015). URL https://link.aps.org/doi/10.1103/PhysRevB.91.085125.

[227] Tran, D. T., Dauphin, A., Grushin, A. G., Zoller, P. & Goldman, N. Probing topology by "heating": Quantized circular dichroism in ultracold atoms. *Science Advances* **3**, e1701207 (2017). URL https://www.science.org/doi/abs/10.1126/sciadv.1701207.

[228] Loring, T. A. Bulk spectrum and K-theory for infinite-area topological quasicrystals. *Journal of Mathematical Physics* **60**, 081903 (2019). URL https://doi.org/10.1063/1.5083051.

[229] Marra, P. & Nitta, M. Topologically quantized current in quasiperiodic Thouless pumps. *Phys. Rev. Research* **2**, 042035 (2020). URL https://link.aps.org/doi/10.1103/PhysRevResearch.2.042035.

[230] Privitera, L. & Santoro, G. E. Quantum annealing and nonequilibrium dynamics of Floquet Chern insulators. *Phys. Rev. B* **93**, 241406 (2016). URL https://link.aps.org/doi/10.1103/PhysRevB.93.241406.

[231] Marsal, Q., Varjas, D. & Grushin, A. G. Topological Weaire–Thorpe models of amorphous matter. *Proceedings of the National Academy of Sciences* **117**, 30260–30265 (2020). URL https://www.pnas.org/doi/abs/10.1073/pnas.2007384117.

[232] Ulčakar, L., Mravlje, J. & Rejec, T. Kibble-Zurek Behavior in Disordered Chern Insulators. *Phys. Rev. Lett.* **125**, 216601 (2020). URL https://link.aps.org/doi/10.1103/PhysRevLett.125.216601.

[233] Huang, H. & Liu, F. Quantum Spin Hall Effect and Spin Bott Index in a Quasicrystal Lattice. *Phys. Rev. Lett.* **121**, 126401 (2018). URL https://link.aps.org/doi/10.1103/PhysRevLett.121.126401.

[234] Huang, H. & Liu, F. Theory of spin Bott index for quantum spin Hall states in nonperiodic systems. *Phys. Rev. B* **98**, 125130 (2018). URL https://link.aps.org/doi/10.1103/PhysRevB.98.125130.

[235] Levin, M. & Stern, A. Fractional Topological Insulators. *Phys. Rev. Lett.* **103**, 196803 (2009). URL https://link.aps.org/doi/10.1103/PhysRevLett.103.196803.

[236] Liu, C.-X., Qi, X.-L. & Zhang, S.-C. Half quantum spin Hall effect on the surface of weak topological insulators. *Physica E: Low-dimensional Systems and Nanostructures* **44**, 906 – 911 (2012). URL http://www.sciencedirect.com/science/article/pii/S1386947711004000.

[237] Alexandradinata, A., Wang, Z. & Bernevig, B. A. Topological Insulators from Group Cohomology. *Phys. Rev. X* **6**, 021008 (2016). URL http://link.aps.org/doi/10.1103/PhysRevX.6.021008.

[238] Marzari, N. & Vanderbilt, D. Maximally localized generalized Wannier functions for composite energy bands. *Phys. Rev. B* **56**, 12847–12865 (1997). URL https://link.aps.org/doi/10.1103/PhysRevB.56.12847.

[239] Tokatly, I. V. Magnetoelasticity theory of incompressible quantum Hall liquids. *Phys Rev B* **73**, 205340 (2006). URL http://prb.aps.org/abstract/PRB/v73/i20/e205340.

[240] Liu, C.-C., Zhou, J.-J., Yao, Y. & Zhang, F. Weak Topological Insulators and Composite Weyl Semimetals: $\beta{-}\mathrm{Bi}_4X_4$ ($X = \mathrm{Br}, \mathrm{I}$). *Phys. Rev. Lett.* **116**, 066801 (2016). URL https://link.aps.org/doi/10.1103/PhysRevLett.116.066801.

[241] Parker, D. E., Morimoto, T., Orenstein, J. & Moore, J. E. Diagrammatic approach to nonlinear optical response with application to Weyl semimetals. *Phys. Rev. B* **99**, 045121 (2019). URL https://link.aps.org/doi/10.1103/PhysRevB.99.045121.

[242] Brown, B. E. The crystal structures of WTe_2 and high-temperature $MoTe_2$. *Acta Crystallographica* **20**, 268–274 (1966). URL https://onlinelibrary.wiley.com/doi/abs/10.1107/S0365110X66000513.

[243] Aroyo, M. I. *et al.* Bilbao Crystallographic Server: I. Databases and crystallographic computing programs. *Zeitschrift für Kristallographie - Crystalline Materials* **221**, 15–27 (2006). URL https://www.degruyter.com/view/journals/zkri/221/1/article-p15.xml.

[244] Aroyo, M. I., Kirov, A., Capillas, C., Perez-Mato, J. M. & Wondratschek, H. Bilbao Crystallographic Server. II. Representations of crystallographic point groups and space groups. *Acta Crystallographica Section A* **62**, 115–128 (2006). URL https://doi.org/10.1107/S0108767305040286.

[245] Aroyo, M. I. *et al.* Crystallography Online: Bilbao Crystallographic Server. *Bulg. Chem. Commun* **43**, 183–197 (2011). URL http://bcc.bas.bg/BCC_Volumes/Volume_43_Number_2_2011/Volume_43_Number_2_2011_PDF/2011_43_2_1.pdf.

[246] Blöchl, P. E., Jepsen, O. & Andersen, O. K. Improved tetrahedron method for Brillouin-zone integrations. *Phys. Rev. B* **49**, 16223–16233 (1994). URL https://link.aps.org/doi/10.1103/PhysRevB.49.16223.

[247] Kresse, G. & Hafner, J. Norm-conserving and ultrasoft pseudopotentials for first-row and transition elements. *Journal of Physics: Condensed Matter* **6**, 8245 (1994). URL https://dx.doi.org/10.1088/0953-8984/6/40/015.

[248] Kresse, G. & Furthmüller, J. Efficiency of ab-initio total energy calculations for metals and semiconductors using a plane-wave basis set. *Computational Materials Science* **6**, 15–50 (1996). URL https://www.sciencedirect.com/science/article/pii/0927025696000080.

[249] Kresse, G. & Furthmüller, J. Efficient iterative schemes for *ab initio* total-energy calculations using a plane-wave basis set. *Phys. Rev. B* **54**, 11169–11186 (1996). URL https://link.aps.org/doi/10.1103/PhysRevB.54.11169.

[250] Perdew, J. P., Burke, K. & Ernzerhof, M. Generalized gradient approximation made simple. *Phys. Rev. Lett.* **77**, 3865–3868 (1996). URL https://link.aps.org/doi/10.1103/PhysRevLett.77.3865.

[251] Bilbao Crystallographic Server. *BANDREP: Band Representations of the Double Space Groups* (2017). URL http://www.cryst.ehu.es/cgi-bin/cryst/programs/bandrep.pl.

[252] Bilbao Crystallographic Server. *Check Topological Mat* (2018). URL https://www.cryst.ehu.es/cryst/checktopologicalmat.

[253] Elcoro, L. *et al.* Double crystallographic groups and their representations on the Bilbao Crystallographic Server. *Journal of Applied Crystallography* **50**, 1457–1477 (2017). URL https://doi.org/10.1107/S1600576717011712.

[254] Pizzi, G. *et al.* Wannier90 as a community code: new features and applications. *Journal of Physics: Condensed Matter* **32**, 165902 (2020). URL https://dx.doi.org/10.1088/1361-648X/ab51ff.

[255] Wu, Q., Zhang, S., Song, H.-F., Troyer, M. & Soluyanov, A. A. WannierTools: An open-source software package for novel topological materials. *Computer Physics Communications* **224**, 405–416 (2018). URL https://www.sciencedirect.com/science/article/pii/S0010465517303442.

[256] Wieder, B. J. *et al.* Topological materials discovery from crystal symmetry. *Nature Reviews Materials* **7**, 196–216 (2022). URL https://doi.org/10.1038/s41578-021-00380-2.

[257] Safeer, C. K. *et al.* Large Multidirectional Spin-to-Charge Conversion in Low-Symmetry Semimetal $MoTe_2$ at Room Temperature. *Nano Letters* **19**, 8758–8766 (2019). URL https://doi.org/10.1021/acs.nanolett.9b03485.

[258] Weber, A. P. *et al.* Spin-Resolved Electronic Response to the Phase Transition in $MoTe_2$. *Phys. Rev. Lett.* **121**, 156401 (2018). URL https://link.aps.org/doi/10.1103/PhysRevLett.121.156401.

[259] Pandey, V. & Pandey, S. K. *PY-Nodes*: An *ab-initio* python code for searching nodes in a material using *Nelder-Mead*'s simplex approach. *Computer Physics Communications* **283**, 108570 (2023). URL https://www.sciencedirect.com/science/article/pii/S0010465522002892.

[260] Wu, Y. *et al.* Observation of Fermi arcs in the type-II Weyl semimetal candidate WTe_2. *Phys. Rev. B* **94**, 121113 (2016). URL https://link.aps.org/doi/10.1103/PhysRevB.94.121113.

[261] Bruno, F. Y. *et al.* Observation of large topologically trivial Fermi arcs in the candidate type-II Weyl semimetal WTe_2. *Phys. Rev. B* **94**, 121112 (2016). URL https://link.aps.org/doi/10.1103/PhysRevB.94.121112.

[262] Wang, C. *et al.* Observation of Fermi arc and its connection with bulk states in the candidate type-II Weyl semimetal WTe_2. *Phys. Rev. B* **94**, 241119 (2016). URL https://link.aps.org/doi/10.1103/PhysRevB.94.241119.

[263] Jiang, J. *et al.* Signature of type-II Weyl semimetal phase in $MoTe_2$. *Nature Communications* **8**, 13973 (2017). URL https://doi.org/10.1038/ncomms13973.

[264] Liang, A. *et al.* Electronic Evidence for Type II Weyl Semimetal State in MoTe2. *ArXiv e-prints* (2016). URL https://arxiv.org/abs/1604.01706.

[265] Kawahara, K. *et al.* Surface structure of novel semimetal WTe_2. *Applied Physics Express* **10**, 045702 (2017). URL https://dx.doi.org/10.7567/APEX.10.045702.

[266] Yuan, Y. *et al.* Quasiparticle interference of Fermi arc states in the type-II Weyl semimetal candidate WTe_2. *Phys. Rev. B* **97**, 165435 (2018). URL https://link.aps.org/doi/10.1103/PhysRevB.97.165435.

[267] Berger, A. N. *et al.* Temperature-driven topological transition in 1T'-$MoTe_2$. *npj Quantum Materials* **3**, 2 (2018). URL https://doi.org/10.1038/s41535-017-0075-y.

[268] von Benda, H., Simon, A. & Bauhofer, W. Zur Kenntnis von BiBr und $BiBr_{1,167}$. *Zeitschrift für anorganische und allgemeine Chemie* **438**, 53–67 (1978). URL https://onlinelibrary.wiley.com/doi/abs/10.1002/zaac.19784380105.

[269] Saito, Y. *et al.* Superconductivity protected by spin–valley locking in ion-gated MoS_2. *Nature Physics* **12**, 144–149 (2016). URL https://doi.org/10.1038/nphys3580.

[270] Lu, J. M. *et al.* Evidence for two-dimensional Ising superconductivity in gated MoS_2. *Science* **350**, 1353–1357 (2015). URL https://www.science.org/doi/abs/10.1126/science.aab2277.

[271] de la Barrera, S. C. *et al.* Tuning Ising superconductivity with layer and spin–orbit coupling in two-dimensional transition-metal dichalcogenides. *Nature Communications* **9**, 1427 (2018). URL https://doi.org/10.1038/s41467-018-03888-4.

[272] Wang, C. *et al.* Type-II Ising Superconductivity in Two-Dimensional Materials with Spin-Orbit Coupling. *Phys. Rev. Lett.* **123**, 126402 (2019). URL https://link.aps.org/doi/10.1103/PhysRevLett.123.126402.
